# Supplementary material for: Stereoselective polar radical crossover for the functionalization of strained-ring systems
Source: Commun Chem. 2024 Jun 19;7:139. doi: 10.1038/s42004-024-01221-3 (PMC11187220; doi:10.1038/s42004-024-01221-3)
Supplement: Supplementary file 2 — Supplementary Information [file 42004_2024_1221_MOESM2_ESM.pdf]

Stereoselective Polar Radical Crossover for the Functionalization of Strained-Ring Systems

Florian Trauner,[a,b] Rahma Ghazali,[a,b] Jan Rettig,[a] Christina M. Thiele,[a] and Dorian Didier[a,b]

[a] F. Trauner, D. Didier, J. Rettig, C.M. Thiele; Clemens-Schöpf-Institut für Organische Chemie und Biochemie; Peter-Grünberg-Straße 4; Technische Universität Darmstadt; 64287 Darmstadt [b] Ludwig-Maximilians-Universität, Department Chemie, Butenandtstrasse 5-13, 81377 München,

Supporting Information

Supplementary Methods

1.	**General Considerations**	**2**
	**1.1 Photochemical Setup**	**3**
2.	**Optimizations**	**5**
3.	**General Procedures**	**9**
4.	**Limitations**	**16**
5.	**Experimental Section**	**17**
6.	**NMR Investigations**	**115**
7.	**References**	**127**

Supplementary Methods

1. General Considerations

All reactions were carried out under dry N_2 atmosphere in flame-dried glassware unless otherwise stated. Syringes, which were used to transfer anhydrous solvents or reagents, were purged with nitrogen three times prior to use. THF (stabilized) was purchased in 99.5 % purity from Acros Organics. Inhibitor-free THF was purchased by Sigma-Aldrich in 99.7 % purity. 2-Methyltetrafuran (inhibitor-free) was purchased in >99 % purity from Sigma-Aldrich. Organolithiums (*n*BuLi, *s*BuLi, *t*BuLi,) were purchased from Rockwood Lithium and the concentration was determined by titration against *i*PrOH using 1,10-phenantroline as indicator. Gringard reagents were prepared in THF, the used magnesium was activated by addition of 1,2-dibromoethane and subsequent heating to reflux. Titration of Gringard reagents was performed with benzoic acid and 4-phenylazodiphenylamin as indicator. Chromatographic purifications were performed using silica gel (SiO_2, 0.040-0.063 mm, 230- 400 mesh ASTM) from Merck or Alumina (Al_2O_3, 32-63 µm) from MP EcoChrom™. The spots were visualized under UV (254 nm) or by staining the TLC plate with either $KMnO_4$ solution (K_2CO_3, 10 g – $KMnO_4$, 1.5 g – H_2O, 150 mL – NaOH 10% in H_2O, 1.25 mL) or Curcumin solution (Curcumin, 0.4 g – EtOH, 400 mL – 2 M HCl, 20 mL). Yields refer to isolated yields of compounds estimated to be >95% pure as determined by ^{1}H NMR and GC-analysis. The ^{13}C and ^{1}H NMR spectra were recorded on VARIAN Mercury 200, BRUKER ARX 300, VARIAN VXR 400 S and BRUKER AMX 600 instruments. Chemical shifts are reported as δ values in ppm relative to the residual solvent peak (^{1}H-NMR, ^{13}C-NMR) in deuterated chloroform ($CDCl_3$: δ 7.26 ppm for ^{1}H-NMR and δ 77.16 ppm for ^{13}C-NMR) and deuterated acetone (Acetone-D_6: δ 2.05 ppm for ^{1}H-NMR and δ 29.84 ppm for ^{13}C-NMR). Abbreviations for signal coupling are as follows: s (singlet), d (doublet), t (triplet), q (quartet), quint (quintet), m (multiplet) and br (broad). Reaction endpoints were determined by GC monitoring of the reactions with *n*dodecane as an internal standard. Gas chromatography was performed with machines of Agilent Technologies 7890, using a column of type HP 5 (Agilent 5% phenylmethylpolysiloxane; length: 15 m; diameter: 0.25 mm; film thickness: 0.25 µm) or Hewlett-Packard 6890 or 5890 series II, using a column of type HP 5 (Hewlett-Packard, 5% phenylmethylpolysiloxane; length: 15 m; diameter: 0.25 mm; film thickness: 0.25 µm). High resolution mass spectra (HRMS) and low-resolution mass spectra (LRMS) were recorded on Finnigan MAT 95Q, Finnigan MAT 90 instrument or JEOL JMS-700. Infrared spectra were recorded on a Perkin 281 IR spectrometer and samples were measured neat (ATR, Smiths Detection DuraSample IR II Diamond ATR). The absorption bands were reported in wave numbers (cm^{-1}) and abbreviations for intensity are as follows: vs (very strong; maximum intensity), s (strong; above 75% of max. intensity), m (medium; from

50% to 75% of max. intensity), w (weak; below 50% of max. intensity) and br (broad). Melting points were determined on a Büchi B-540 apparatus and are uncorrected.

1.1 Photochemical Setup

Photochemical Reactions at ambient temperature were performed using the EvoluChem PhotoRedOx Box Setup by Hepatochem. *Via* built in cooling fan the temperature could be adjusted to 15°C. The employed LED's were 365 nm (18 W, 60 °, EvoluChem 365PF) and/or 450 nm (18 W, 25 °, EvoluChem 450PF). Reactions at or below 0°C were performed using a Huber TC-100e immersion cooler to chill a dewar filled with EtOH (cutoff wavelength 210 nm).

Supplementary Figure 1: Photochemical Setup for low-temperature Reactions

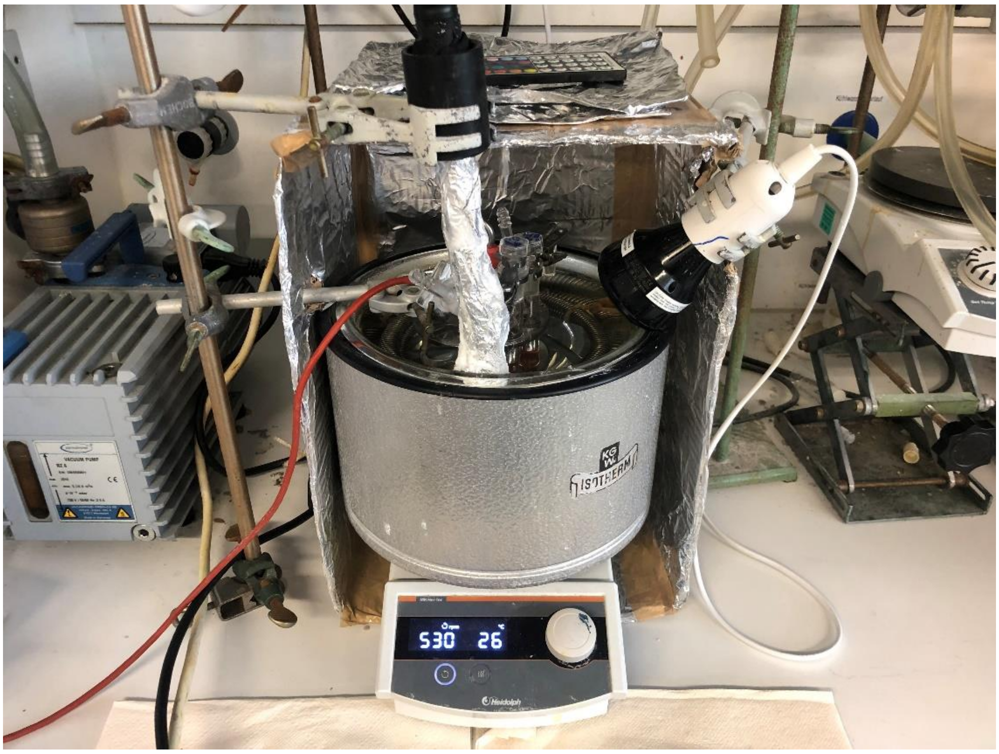

Supplementary Figure 2 *:* UV-Vis emission spectrum of employed EvoluChem 450PF LED*.*

HepatoChem

Reinventing Chemistry for Life Sciences

LIGHT MEASUREMENT

Description	Part number	LED	Batch Number	S/N	Performed By	Date
EvoluChem 450PF	HCK1012-01-002	CREE XPE	[redacted]	[redacted]	[redacted]	[redacted]

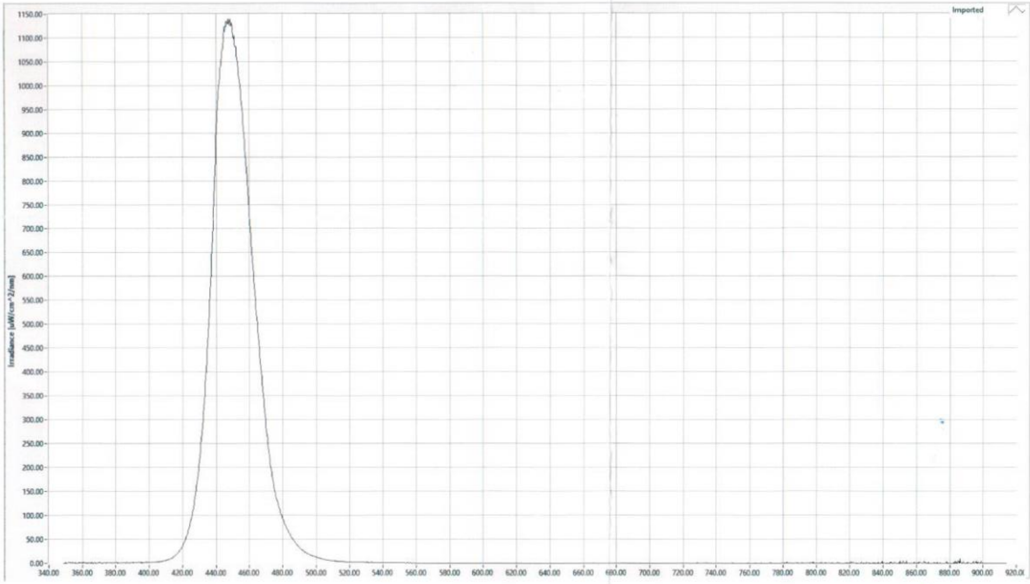

Graph Irradiance Power Radiance Peak

Color Data

CCT	5328	K	Duv	NaN
u	0.2042		u'	0.2042
v	0.05077		v'	0.07616
X	603.19		x	0.1531
Y	100		y	0.02537
Z	3237.9		z	0.8216

CRI Data

Ra -62.59

R1	-61.63	R5	-44.11
R2	-18.57	R6	-54.86
R3	-93.35	R7	-34.22
R4	-113.5	R8	-80.45

Total Band Output

Total Irradiance	34219.4	uW/cm^2
Selected Irradiance	0	uW/cm^2
Total Illuminance	11576.6	lux
PAR	1286.12	µmol/m^2/s
Luminous Intensity	11576.6	cd

VIS - UV Band

VIS [380-780]	341.24	W/m^2
UVA [321-390]	43.0749	uW/cm^2
UVB [281-320]	0	uW/cm^2
UVC [<280]	0	uW/cm^2

Dominant - Complementary Wavelength

DWL	455.0	CWL	572.0
Purity	0.9908	Purity	1.474

HepatoChem, Inc. 100 Cummings Center, Suite 451C; Beverly, MA 01915 http://www.hepatochem.com ; Tel: (617)-500-5285; Fax: (617)-274-0827

2. Optimization of Reaction Parameters

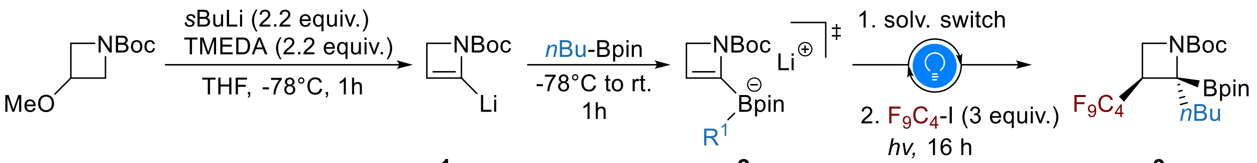

1 2 3

Supplementary **Table 1:** Reaction parameter optimization

**Solvent**	**Hv [nm]**	**Eq. Bpin**	**Photocatalyst**	***T* [°C]**	***dr* [A]**	**Yield[A,B]**
MeCN	365	1	-----	15	2:1	51 (45)
MeCN	365	1	-----	Rt.	1:1	47 (41)
Et_2O	365	1	-----	15	2:1	40 (34)
THF	365	1	$Ru(bpy)_3(PF_6)_2$	Rt.	3:1	50
THF	365+450	1	$Ru(bpy)_3(PF_6)_2$	-20	3:1	42 (39)
THF	365+450	1	$Ru(bpy)_3(PF_6)_2$	-40	4:1	55 (53)
THF	365+450	1	-----	-40	4:1	44
THF	365	2	-----	15	3:1	68 (66)
THF	365+450	2	-----	-40	4:1	77 (75)
THF	365+450	1.5	-----	-40	4:1	78 (77)
THF	450	1.5	-----	-20	4:1	74
THF	450	1.5	$Ru(bpy)_3(PF_6)_2$	-20	4:1	66 (64)
THF	450	1.3	-----	-20	4:1	62 (59)
DMI	450	1.5	-----	-20	1.5:1	67
EtCN	450	1.5	-----	-40	1:1	49
DCM	450	1.5	-----	-40	4:1	75 (74)
m-THF	450	1.5	-----	-40	5:1	73 (71)
m-THF	450	1.3	-----	-40	5:1	74 (71)
DCE	450	1.5	-----	-20	3:1	64
Dioxane/ THF (1:1)	450	1.5	-----	Rt.	4:1	nd
DME	450	1.5	-----	-20	2:1	41
BTF	450	1.5	-----	-20	3:1	51
m-THF	-----	1.3	-----	-40	5:1	nd

[A] The *dr* was determined by ^{19}F NMR analysis of the crude reaction mixture before chromatographical purification. Yields were determined by GC-analysis using Dodecane as internal standard.[B] Yields in parentheses refer to isolated yields after FCC.

Concentration Optimization

***Supplementary* Table 2:** *Concentration optimization*.

Concentration A [M][A]	Concentration B [M][B]	*dr* [C]	Yield [D]
0.15	0.1	5:1	74
0.3	0.1	5:1	67
0.3	0.3	4:1	66
0.15	0.05	5:1	69

[A] referring to the respective molarity in the lithiation-elemination step. [B] referring to the respective molarity after the solvent switch.[C] The *dr* was determined by ^{19}F NMR analysis of the crude reaction mixture before chromatographical purification. [D] isolated yields.

Boron Source Optimization

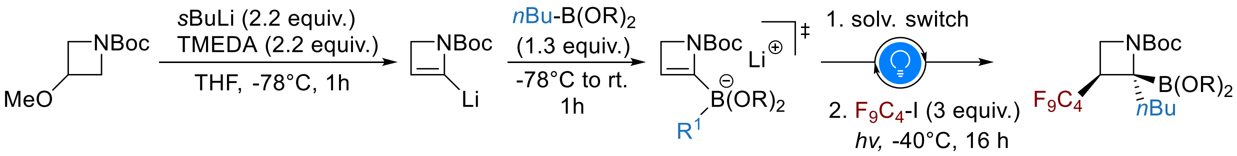

***Supplementary* Table 3:** Boron source optimization.

Boron Source	*T* [°C]	*dr* [A]	Yield [%][A,B]
L1 = B(pin)	-40	5:1	73 (71)
L2 = B(Epin)	-40	7:1	92 (92)
L3	-40	3:1	63
L4 = B(neo)	-40	13:1	<5
L4 = B(neo)	-20	>20:1	<5
L4 = B(neo)	15	>20:1	<5
L5	-40	8:1	<5
L6	-40	5:1	<5
L7 = B(mac)	-40	3:1	32
L7 = B(mac)	-60	3:1	37
L8	-40	1.5:1	39
L9	-40	nd	<5
L10	-40	2:1	70
L11	-40	nd	nd
L12	-40	2:1	44

[A] The *dr* was determined by ^{19}F NMR analysis of the crude reaction mixture before chromatographical purification. Yields were determined by GC-analysis using Dodecane as internal standard.[B] Yields in parentheses refer to isolated yields after FCC.

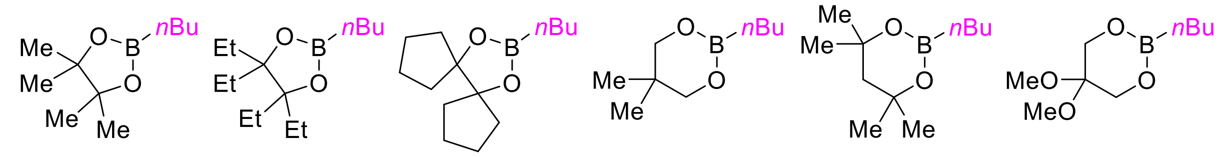

L1 B(pin) | L2 B(Epin) | L3 | L4 B(neo) | L5 | L6

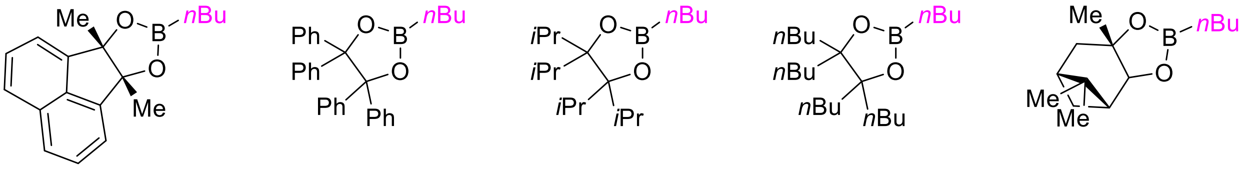

L7 B(mac) | L8 | L9 | L10 | L12

Observations:

- Partial protodeboronation of obtained azetidines on silica gel was observed when using L1 (Bpin). Hereby protodeboronation was especially dominant with aromatic residues as R[1].
- More sterically demanding ligands suppressed protodeboronation.

Additives/ Cosolvents

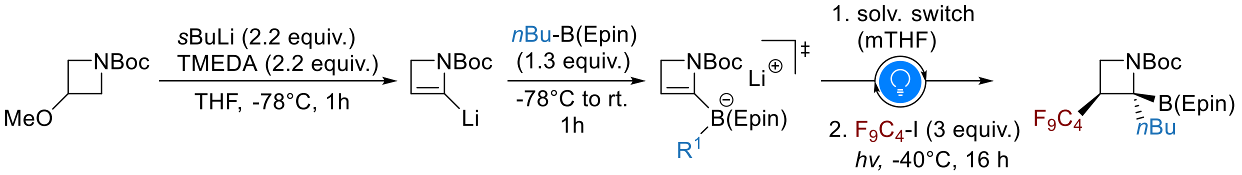

1 2 3

***Supplementary* Table 4:** Additives/ Cosolvents

Additive/ Cosolvent		*dr* [crude][A]	Yield [%][A,B]
None	-40	6:1	93
Dodecane 1Vol%	-40	7:1	92
Dodecane 5Vol%	-40	8:1	94 (93)
Dodecane 10Vol%	-40	8:1	88 (89)
$ZnCl_2$ (1 equiv.)	-40	4:1	67
BTF (2 equiv.)	-40	5:1	nd
18-crown-6	-40	4:1	nd

[A] The *dr* was determined by ^{19}F NMR analysis of the crude reaction mixture before chromatographical purification. Yields were determined by GC-analysis using Dodecane as internal standard.[B] Yields in parentheses refer to isolated yields after FCC.

Observations:

- when dodecane (1 Vol%.) was omitted the *dr* decreased to 6:1
- increasing the amount of dodecane to 5 Vol%. the *dr* rose to 8:1. 10 Vol%. dodecane led to a decreased yield, while not affecting anymore the *dr*.

Addition temperature for Cyclobutanes

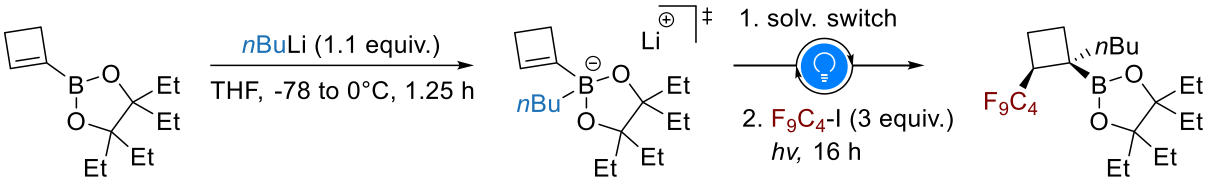

4 **5** **6a**

***Supplementary* Table 5:** Optimization of addition temperature for cyclobutanes

Addition Temperature[A]	Warming to[B]	*dr* [crude][C]	Yield [%][D]
0°C	25°C	>20:1	31
-20°C	0°C	>20:1	63
-40°C	0°C	>20:1	77
-78°C	0°C	>20:1	82

[A] referring to the reaction temperature when adding *n*BuLi. [B] referring to the reaction temperature to which the reaction is allowed to warm 30 min after addition of *n*BuLi. [C] The *dr* was determined by ^{19}F NMR analysis of the crude reaction mixture before chromatographical purification. [C] isolated yields.

Observations:

- Addition of the Lithium Species at 0°C and stirring at this temperature for 30min, followed by warming to rt. leads to a visible (brown) degradation/ insufficient formation of the boronate complex.
- The optimum addition temperature was found to be -78°C with stirring at that temperature for 30min, followed by warming to 0°C for 45min.
- For azetidines, warming to rt. is essential as only minor ate complex formation is observed at 0°C.

3. General Procedures

General Procedure A: Synthesis of trisubstituted azetidines

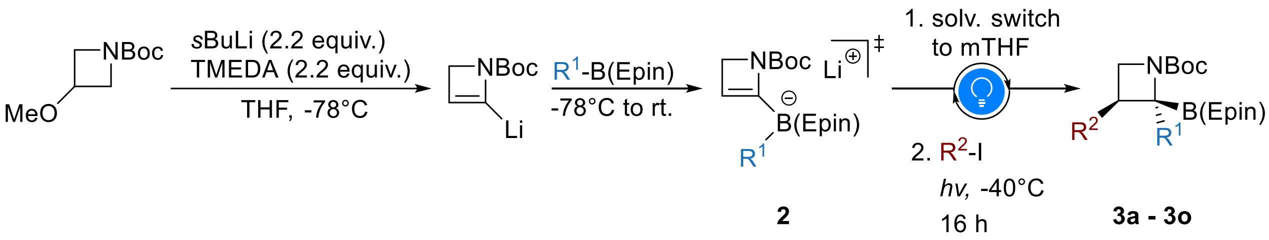

2 **3a - 3o**

A flame-dried Schlenk flask was charged with *tert*-butyl 3-methoxyazetidine-1-carboxylate (56 mg, 0.3 mmol, 1.0 equiv.) followed by the addition of THF (2 mL). The solution was cooled to -78°C and TMEDA (99 µL, 0.66 mmol, 2.2 equiv.) was added. *s*BuLi (0.66 mmol, 2.2 equiv.) was added dropwise over a period of 4 minutes (0.12 mL/ min) and the pale-red solution was allowed to stir for 1h at -78°C. Subsequently, the respective boronate ester (0.39 mmol, 1.3 equiv.) was dissolved in THF (0.5 mL) and added dropwise to the solution. Following this, the mixture was stirred at -78°C for 30 minutes, and was then allowed to warm to ambient temperature. After stirring at this temperature for a further 30 minutes, the solvents were carefully removed *in vacuo*. The residual oil was dissolved in 2-methyltetrahydrofuran (2.5 mL), dry dodecane (0.15 mL, 5 Vol%.) was added and the flask was transferred to the photoreactor precooled to -40 °C. The respective radical precursor (0.9 mmol, 3.0 equiv. or 0.6 mmol, 2.0 equiv.) was dissolved in 2-methyltetrahydrofuran (0.5 mL) and added dropwise to the solution under irradiation with a blue LED (450 nm, 18 W). The yellow solution was stirred at the indicated temperature under LED irradiation for 16 h. The crude mixture was pushed through a Silica-Plug (4 cm), eluting with Et_2O (50 mL) and was concentrated *in vacuo*. Flash column chromatography (SiO_2 or Al_2O_3; pentane – EtOAc) afforded the respective Azetidines (**3a - 3o**).

Note: TMEDA was distilled from CaH_2 and stored under inert atmosphere prior to use. *s*BuLi was filtered *via* Syringe filter prior to use.

General Procedure B: Synthesis of trisubstituted Cyclobutanes

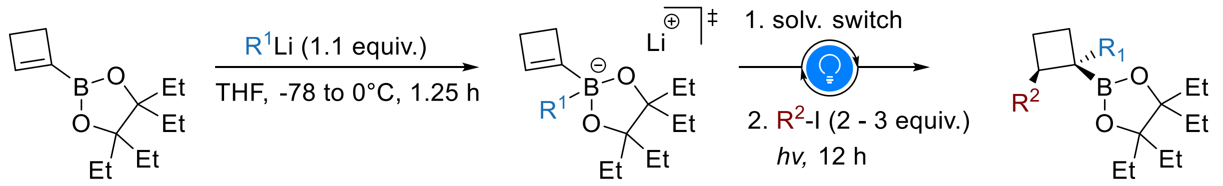

4 **5** **6a - 6h**

A flame-dried Schlenk flask was charged with the respective Aryl/ Alkyl halide for Halogen-Lithium exchange (0.22 mmol, 1.1 equiv.), THF (1 mL) was added and the solution was cooled to -78°C. *t*BuLi/ *n*BuLi (0.22 mmol, 1.1 equiv.) was slowly added dropwise and the mixture was stirred for the indicated time at -78°C, then warming to rt. 2-(Cyclobut-1-en-1-yl)-4,4,5,5-tetraethyl-1,3,2-dioxaborolane (**4**) (47 mg, 0.2 mmol, 1.0 equiv.) was dissolved in THF (0.3 mL) and added slowly dropwise to the mixture at -78°C. Commercially available lithium species (1.1 equiv.) were also added at -78°C. Stirring was continued at this temperature for 30 min, before the mixture was allowed to warm to 0°C for 45 min. Following this, the solvents were carefully removed *in vacuo*. The residue was dissolved in 2-methyltetrahydrofuran (1.5 mL), dry dodecane (0.1 mL, 5 Vol%.) was added and the flask was transferred to the photoreactor precooled to -40 °C. The respective radical precursor (0.6 mmol, 3.0 equiv. or 0.4 mmol, 2.0 equiv.) was dissolved in 0.5 mL 2-methyltetrahydrofuran and added dropwise to the solution under irradiation with a blue LED (450 nm, 18 W). The colorless solution was stirred at the indicated temperature under LED irradiation for 12 h. The crude mixture was pushed through a Silica-Plug (4 cm), eluting with Et_2O (50 mL) and was concentrated *in vacuo*. Flash column chromatography (SiO_2; pentane – EtOAc) afforded the respective Cyclobutanes (**6a – 6h**).

General Procedure C: Synthesis of trisubstituted Cyclopentanes

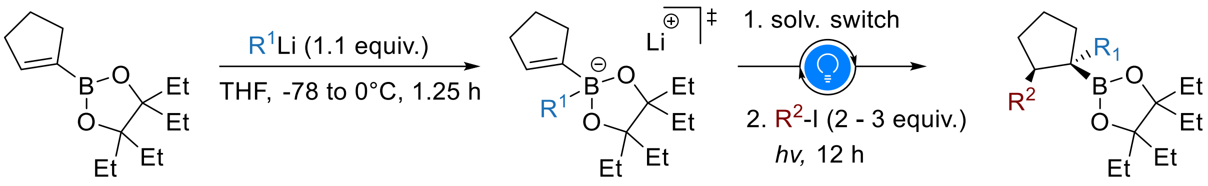

7 **8** **9a - 9h**

A flame-dried Schlenk flask was charged with the respective Aryl/ Alkyl halide for Halogen-Lithium exchange (0.22 mmol, 1.1 equiv.), THF (1 mL) was added and the solution was cooled to -78°C. *t*BuLi/ *n*BuLi (0.22 mmol, 1.1 equiv.) was slowly added dropwise and the mixture was stirred for the indicated time at -78°C, then warming to rt. Commercially available lithium species (1.1 equiv.) were also added at -78°C. 2-(Cyclopent-1-en-1-yl)-4,4,5,5-tetraethyl-1,3,2-dioxaborolane (**7**) (50 mg, 0.2 mmol, 1.0 equiv.) was dissolved in THF (0.3 mL) and added slowly dropwise to the mixture at -78°C. Stirring was continued at this temperature for 30 min, before the mixture was allowed to warm to 0°C for 45 min. Following this, the solvents were carefully removed *in vacuo*. The residue was dissolved in 2-methyltetrahydrofuran (1.5 mL), dry dodecane (0.1 mL, 5 Vol%.) was added and the flask was transferred to the photoreactor precooled to -40 °C. The respective radical precursor (0.6 mmol, 3.0 equiv. or 0.4 mmol, 2.0 equiv.) was dissolved in 0.5 mL 2-methyltetrahydrofuran and added dropwise to the solution under irradiation with a blue LED (450 nm, 18 W). The colorless solution was stirred at the indicated temperature under LED irradiation for 12 h. The crude mixture was pushed through a Silica-Plug (4 cm), eluting with Et_2O (50 mL) and was concentrated *in vacuo*. Flash column chromatography (SiO_2; pentane – EtOAc) afforded the respective Cyclopentanes (**9a - 9h**).

General Procedure D: Synthesis of trisubstituted Tetrahydrofuranes

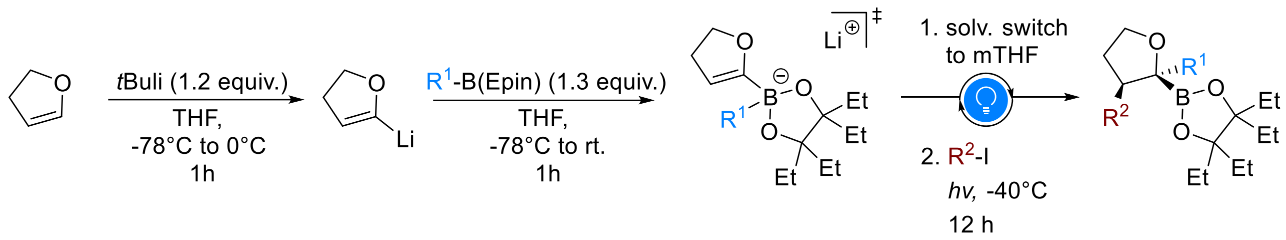

10 **11** **12b; 12c**

A flame-dried Schlenk flask was charged with 2,3-dihydrofuran (15 µL, 0.2 mmol, 1.0 equiv.) followed by the addition of THF (1 mL). The solution was cooled to -78°C and *t*BuLi (0.24 mmol, 1.2 equiv.) was added dropwise over a period of 3 minutes. The reaction mixture stirred at that temperature for a further 10 min. and was then allowed to warm to 0°C for 45 min. Subsequently the mixture was recooled to -78°C and the respective Boronate ester (0.26 mmol, 1.3 equiv.) was added in 0.5 mL THF. The mixture was allowed to stir at -78°C for 30 min before warming to rt. for a further 30 min. Following this, the solvents were carefully removed *in vacuo*. The residue was dissolved in 2-methyltetrahydrofuran (1.5 mL), dry dodecane (0.1 mL, 5 Vol%.) was added and the flask was transferred to the photoreactor precooled to -40 °C. The respective radical precursor (0.6 mmol, 3.0 equiv. or 0.4 mmol, 2.0 equiv.) was dissolved in 2-methyltetrahydrofuran (0.5 mL) and added dropwise to the solution under irradiation with a blue LED (450 nm, 18 W). The colorless solution was stirred at the indicated temperature under LED irradiation for 12 h. The crude mixture was pushed through a Silica-Plug (4 cm), eluting with Et_2O (50 mL) and was concentrated *in vacuo*. Flash column chromatography (SiO_2; pentane – EtOAc) afforded the respective tetrahydrofuranes (**12b; 12c**).

General Procedure E: Synthesis of symmetric Diols by Pinacol Coupling using $TiCl_4$ and Zn

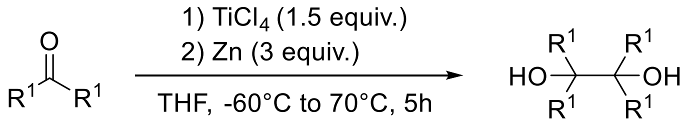

In analogy to a literature procedure[1], a flame-dried three-necked flask was charged with anhydrous THF (100 mL) and the respective ketone (40 mmol, 1.0 equiv.) was added. The

solution was cooled to -60°C and $TiCl_4$ (6.6 mL, 60 mmol, 1.5 equiv.) was added dropwise over a period of 30 minutes via a dropping funnel. The yellow suspension was stirred for a further 30 minutes at the aforementioned temperature and was then allowed to warm to ambient temperature. Zn dust (7.86 g, 120 mmol, 3 equiv.) was added in one portion and the green suspension was heated to 70°C for 3 h. Then, the mixture was cooled to 0°C and a saturated aqueous solution of K_2CO_3 was added carefully, upon which stirring was continued for a further 30 minutes. The suspension was filtered through a plug of Celite, and the solid residues were washed with EtOAc (120 mL). Following this, the organic phase was separated and the aqueous phase was extracted with EtOAc (3 × 50 mL). All organic fractions were combined, washed with Brine and dried over anhydrous magnesium sulfate. Evaporation of the solvents *in vacuo* and flash column chromatography (*i*hexane/ EtOAc) afforded the respective diols.

Note: When filtering the dark suspension over Celite, only the organic phase should be decanted.

General Procedure F: Synthesis of symmetric Diols by Pinacol Coupling using Lithium powder

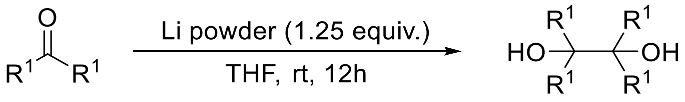

A pre-weighted and flame-dried Schlenk-flask was charged with Lithium powder (Suspension in Heptane) and the solvent was removed *in vacuo*. After determining the amount of Lithium (usually around 0.5 g, 70 mmol, 1.0 equiv.) anhydrous THF (200 mL) was added. The respective Ketone (56 mmol, 0.8 equiv.) was added dropwise and the grey suspension was allowed to stir overnight. Then, the reaction mixture was poured into ice-water (150 mL) and the aqueous phase was extracted with EtOAc (4 × 100 mL). The combined organic fractions were washed with brine, dried over anhydrous magnesium sulfate and concentrated *in vacuo*. Flash column chromatography (SiO_2, *i*hexane; EtOAc) afforded the desired diols.

General Procedure G: Synthesis of Boronic Esters

Method A – $FeCl_3$ mediated esterification

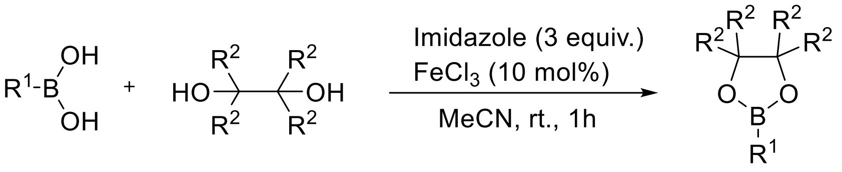

According to a modified literature procedure[2]: to an Erlenmeyer flask equipped with a stirring bar was added in the following order: MeCN (20 mL), Boronic acid (4 mmol, 1.0 equiv.), the respective diol (4 mmol, 1.0 equiv.), Imidazole (0.81 g, 3.0 equiv.), and $FeCl_3$ (32 mg, 0.2 mmol, 5 mol%.) The mixture was stirred at ambient temperature for 1h, then filtered and concentrated *in vacuo*. The crude product was purified *via* flash column chromatography (SiO_2 plug, *i*hexane/ EtOAc) to yield the desired Boronate esters.

Note: Method selected for insensitive substrates

Method B – classical esterification

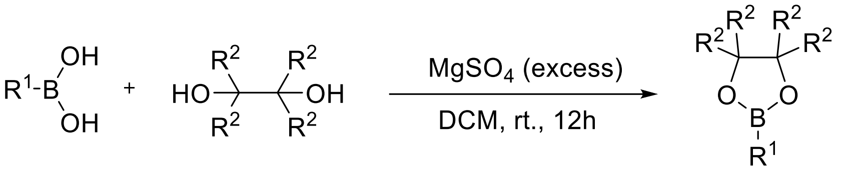

A flame-dried flask was charged with the respective Boronic acid (4 mmol, 1.0 equiv.), the respective Diol (4 mmol, 1.0 equiv.) and anhydrous magnesium sulfate (1 g). Anhydrous DCM (15 mL) was added and the suspension was allowed to stir overnight. After filtration and concentration *in vacuo*, the crude boronic acid esters were purified by flash-column chromatography (SiO_2, *i*hexane; EtOAc).

Note: This method is typically selected for base sensitive substrates.

Method C – *via* respective methoxyboronic ester

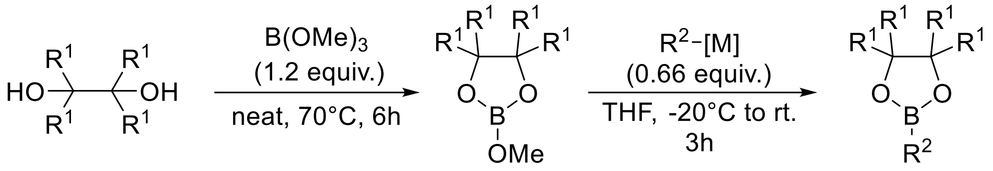

A flame-dried flask equipped with a Dean-Stark apparatus was charged with the respective diol (20 mmol, 1.0 equiv.) and $B(OMe)_3$ (2.2 mL, 20 mmol, 1.2 equiv.) was added *via* syringe and the mixture was heated to 70 °C for 6 h. Residual $B(OMe)_3$ was removed under high-vacuum to afford the respective crude methoxy boronic esters, which were employed in the next step without further purification.

Hereby, the respective methoxy boronic ester (2 mmol, 1.5 equiv.) was dissolved in dry THF (10 mL) and the reaction mixture was cooled to -20°C. The respective Grignard or Lithium species (1.3 mmol, 1.0 equiv.) was added dropwise and the solution was allowed to stir for 1h at the aforementioned temperature. After warming to rt. and stirring at that temperature for a further 2h, the reaction was quenched by addition of HCl (1M, 2 mL). The solution was extracted with Et_2O (3 × 15 mL), the combined organic fractions were washed with Brine, dried over anhyd. $MgSO_4$ and concentrated *in vacuo.* Flash column chromatography (SiO_2; pentane – EtOAc) afforded the desired Boronic esters.

Note: This method is preferentially selected for Boronic esters containing bulky diol ligands. Method A or B only afford insufficient yields for these substrates.

4. Limitations

For Azetidines:

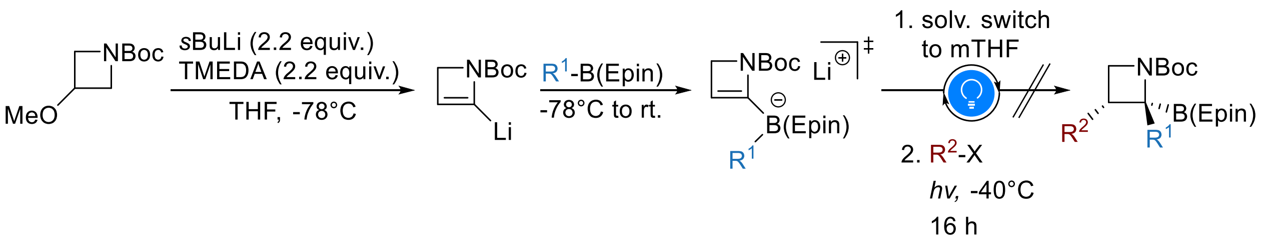

incompatible with R2-X =

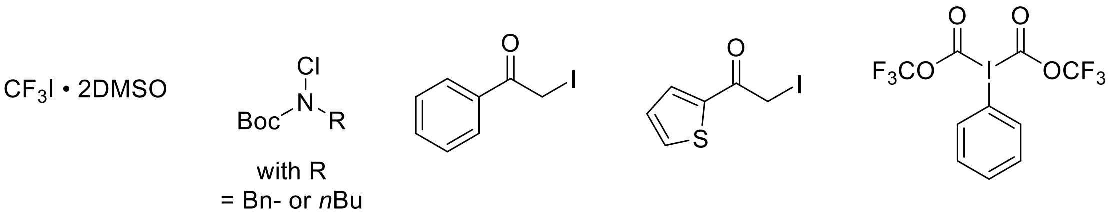

incompatible with R1-B(Epin) =

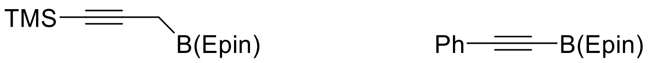

- Radical precursors Ritter trifluoroiodomethane, *N*-chlorocarbamates and α-Iodoketones shown above did not furnish the desired products.
- Alkynes as R[1] proved incompatible; tertiary alkyl moieties as well.

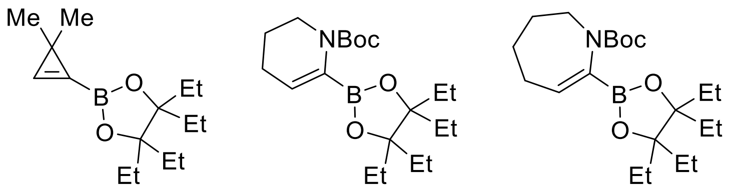

- Ate complex formation observed for depicted cyclopropene, *N*-Boc-tetrahydropyridine and *N*-Boc-tetrahydroazepine but no desired product detected on GC-MS.

5. Experimental Data

tert-Butyl 3-methoxyazetidine-1-carboxylate (<u>Si-1</u>)

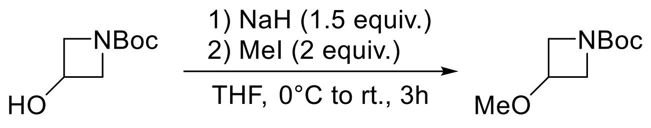

In analogy to a modified literature procedure[3], a flame-dried Schlenk-Flask was charged with *tert*-butyl 3-hydroxyazetidine-1-carboxylate (5.2 g, 30 mmol, 1.0 equiv.) and dry THF (50 mL). The solution was cooled to 0°C and NaH in mineral oil (1.8 g, 45 mmol, 1.5 equiv.) was added portionwise. The ice-bath was removed after 10 min and the suspension was allowed to warm to rt. and stir for 50 min. Once again the mixture was cooled to 0°C and MeI (60 mmol, 2.0 equiv.) was added dropwise. The ice-bath was removed and the mixture stirred for 3 hours before it was carefully quenched by addition of MeOH (20 mL). The solvents were removed *in vacuo*, and the mixture was redissolved in Et_2O and filtered. After removal of residual solvents *in vacuo*, and flash-column chromatography (SiO_2; pentane – EtOAc: 85:15) the title compound was obtained as a colorless oil (5.3 g, 28 mmol, 94%). If a yellowish oil was obtained, the product was pushed through a short plug of activated charcoal eluting with Et_2O.

^{1}H NMR (400 MHz, $CDCl_3$): δ (ppm) = 4.10 – 4.02 (m, 1H), 4.01 – 3.97 (m, 2H), 3.78 – 3.72 (m, 2H), 3.21 (s, 3H), 1.37 (s, 9H).
^{13}C NMR (100 MHz, $CDCl_3$): δ (ppm) = 156.48, 79.63, 69.15, 56.12, 28.48.

3,4-Diethylhexane-3,4-diol – Epin (L-2)

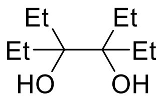

According to GP-E, using 3-pentanone (38.4 mL, 350 mmol, 1.00 equiv.) in THF (900 mL) and $TiCl_4$ (57.6 mL, 0.525 mol, 1.50 equiv.), as well as Zn powder (68.7 g, 1.05 mol, 3.00 equiv.). Final flash-column chromatography (SiO_2; pentane – EtOAc: 9:1 to 85:15) afforded the title compound as a slightly yellow oil, which was eluted through a plug of activated charcoal, to obtain 3,4-diethylhexane-3,4-diol as a colorless oil (24 g, 0.14 mol, 79%).[1]

Note: Running the synthesis on this scale requires slow and portionwise addition of the Zn powder.

^{1}H NMR (400 MHz, $CDCl_3$): δ (ppm) = 1.99 (s, 2H), 1.61 (qd, J = 7.5, 2.7 Hz, 8H), 0.94 (t, J = 7.5 Hz, 12H).

^{13}C NMR (100 MHz, $CDCl_3$): δ (ppm) = 78.96, 27.43, 9.17.

[1,1'-Bi(cyclopentane)]-1,1'-diol (L-3)

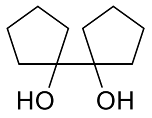

According to GP-E, using cyclopentanone (3.5 mL, 40 mmol, 1.0 equiv.) and final flash-column chromatography (SiO_2; pentane – EtOAc: 89:11) afforded the title compound as a colorless solid (2.3 g, 13 mmol, 67%).

^{1}H NMR (400 MHz, $CDCl_3$): δ (ppm) 1.94 (s, 2H), 1.90 – 1.78 (m, 4H), 1.79 – 1.67 (m, 4H), 1.67 – 1.57 (m, 8H).
^{13}C NMR (100 MHz, $CDCl_3$): 87.30, 36.54, 24.98.

2,4-Dimethylpentane-2,4-diol (L-5)

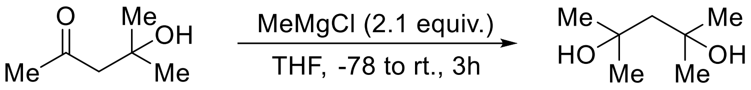

A flame-dried flask was charged with 4-hydroxy-4-methylpentan-2-one (20 mmol, 1.0 equiv.) and dissolved in anhydrous THF (50 mL). A solution of MeMgCl in THF (42 mmol, 2.1 equiv.) was added dropwise *via* syringe-pump at -78°C and the solution was allowed to stir at that temperature for 1 h before warming to rt. After stirring at that temperature for another 2 h, the reaction was quenched by addition of saturated aqueous NH_4Cl (5 mL). The organic phase was separated and the aqueous fraction was extracted with EtOAc (3 × 50 mL). The combined organic fractions were washed with Brine, dried over anhydrous magnesium sulfate and concentrated *in vacuo*. Flash column chromatography (SiO_2; pentane – EtOAc: 87:13) afforded the title compound as a colorless oil (1.7 g, 13 mmol, 63%)

^{1}H NMR (400 MHz, $CDCl_3$): δ (ppm) = 1.75 (s, 2H), 1.34 (s, 12H).
^{13}C NMR (100 MHz, $CDCl_3$): δ (ppm) = 72.36, 52.31, 32.19.

2,2-Dimethoxypropane-1,3-diol (L-6)

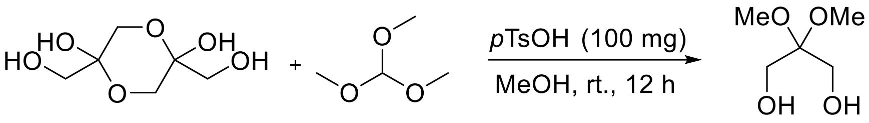

According to a literature procedure[4], 2,5-dihydroxydioxane-2,5-dimethanol (25 g, 0.14 mol, 1.0 equiv.), *p*-toluenesulfonic acid (100 mg), and trimethyl orthoformate (30 mL, 0.27 mol, 1.3 equiv.) were suspended in MeOH (300 mL) and stirred at ambient temperature overnight. Following this, Na_2CO_3 (300 mg) was added and the solvent was removed *in vacuo*. The solid residue was purified *via* flash column chromatography (SiO_2; DCM – MeOH: 93:7) to yield the title compound as a light yellow solid (13.8 g, 101 mmol, 73%).

^{1}H NMR (400 MHz, $CDCl_3$): δ (ppm) = 3.69 (d, J = 6.1 Hz, 4H), 3.30 (s, 6H).
^{13}C NMR (100 MHz, $CDCl_3$): δ (ppm) = 100.11, 61.88, 48.89.

***syn*-1,2-Dimethyl-1,2-dihydroacenaphthylene-1,2-diol – Mac (L-7)**

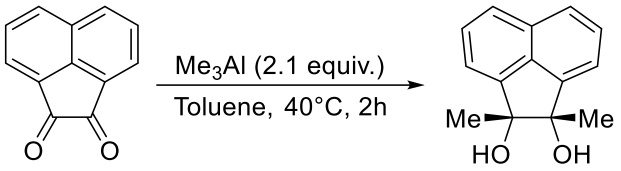

According to a literature procedure[5], a flame-dried 1 L three-necked flask was charged with Acenaphthoquinone (18.2 g, 100 mmol, 1.00 equiv.). Dry toluene was added and the resulting yellow suspension was heated to 40 ºC. A 2 M solution of Trimethylaluminum in hexane (105 mL, 210 mmol, 2.10 equiv.) was added dropwise *via* syringe pump. Upon completion of addition, the reaction was allowed to stir for 1 hour at 40 ºC, cooled to 0 ºC and quenched carefully by addition of H_2O (50 mL) and 2 M HCl (30 mL). EtOAc (200 mL) was added and the mixture was filtered over a plug of Celite, washing with EtOAc (50 mL). The filtrate was transferred in a separatory funnel and washed with water (200 mL). The aqueous fraction was extracted with EtOAc (3 × 150 mL) and the combined organic layers were washed with B rine, dried over anhydrous magnesium sulfate and concentrated *in vacuo*. The crude product was obtained in a *syn*/*anti* ratio of 3.9:1 as determined by crude NMR. Recrystallization from EtOAc (500 mL) afforded pure *syn*-1,2-dimethyl-1,2-dihydroacenaphthylene-1,2-diol as off-white crystals (10.3 g, 48.3 mmol, 48% yield).

^{1}H NMR (400 MHz, $CDCl_3$): δ (ppm) = 7.75 (d, J = 8.2 Hz, 2H), 7.60 – 7.53 (m, 2H), 7.48 (d, J = 6.9 Hz, 2H), 3.01 (s, 2H), 1.62 (s, 6H).
^{13}C NMR (100 MHz, $CDCl_3$): δ (ppm) = 146.27, 134.42, 131.25, 128.61, 125.00, 119.26, 82.29, 23.45.

3,4-Diisopropyl-2,5-dimethylhexane-3,4-diol (L-9)

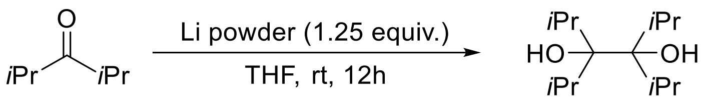

Following GP-F, with freshly distilled 2,4-dimethylpentan-3-on (7.5 mL, 53 mmol, 0.8 equiv.) and Lithium powder (0.46 g, 66 mmol, 1.0 equiv.) in dry THF (200 mL). Flash column chromatography (SiO_2; pentane – EtOAc: 9:1) afforded the title compound as a colorless solid (2.6 g, 11 mmol, 43%).

^{1}H NMR (400 MHz, CDCl$_3$): δ (ppm) = 2.43 – 2.28 (m, 4H), 1.04 (s, 24H).
^{13}C NMR (100 MHz, CDCl$_3$): δ (ppm) = 83.03, 20.59.

5,6-Dibutyldecane-5,6-diol (<u>L-10</u>) and 4,4,5,5-tetrabutyl-2-methoxy-1,3,2-dioxaborolane

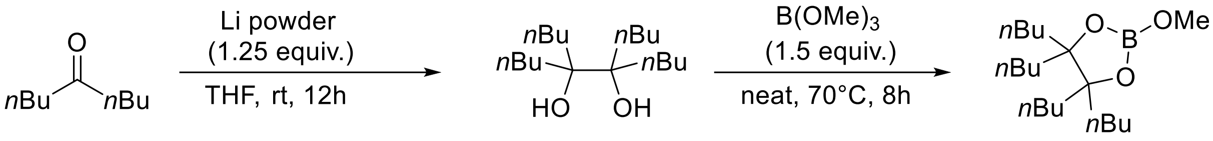

Following GP-F with 5-nonanone (8.4 mL, 49 mmol, 0.8 equiv.) and Lithium powder (0.43 g, 61 mmol, 1.0 equiv.) in dry THF (200 mL). Flash column chromatography (SiO_2; pentane – EtOAc; 95:5 to 9:1) afforded the title compound as a colorless solid (2.17 g, 7.6 mmol, 31%). The full amount of the title compound was directly employed to obtain 4,4,5,5-tetrabutyl-2-methoxy-1,3,2-dioxaborolane as follows: A dry 25 mL flask equipped with reflux condenser was charged with diol (2.17 g, 7.6 mmol, 1.0 equiv.) and $B(OMe)_3$ (1.3 mL, 11 mmol, 1.5 equiv.) and the reaction mixture was heated to 70°C for 8 h according to TLC. The resulting viscous oil was dried under high-vacuum and directly used to prepare 2,4,4,5,5-Pentabutyl-1,3,2-dioxaborolane without further purification.

<u>Note:</u> with increasing steric hindrance of the diol ligands Method C of General Procedure C is preferred over Method A or B.

Overview: Boronic Esters employed for Optimization

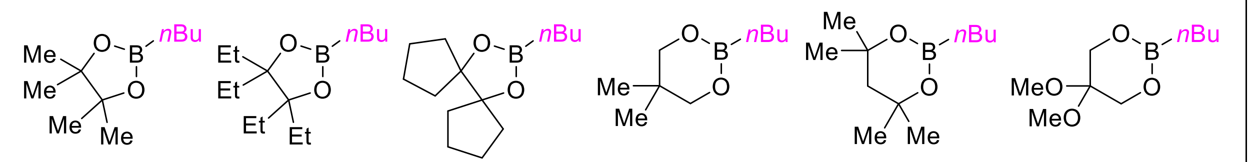

Si-2 B(pin) | **Si-3** B(Epin) | **Si-4** | **Si-5** B(neo) | **Si-6** | **Si-7**

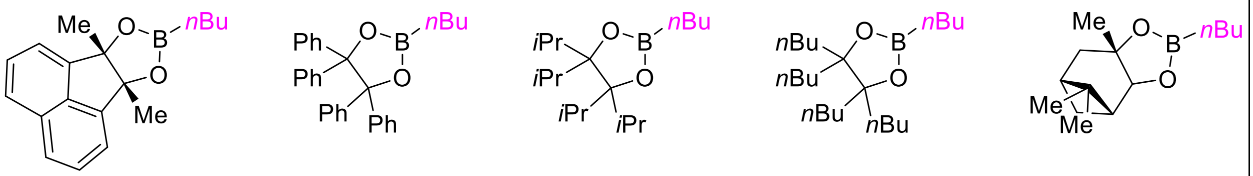

Si-8 B(mac) | **Si-9** | **Si-10** | **Si-11** | **Si-12**

2-Butyl-4,4,5,5-tetramethyl-1,3,2-dioxaborolane (Si-2)

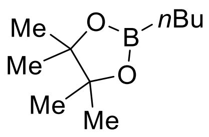

According to GP-G (Method A), using *n*butylboronic acid (0.41 g, 4.0 mmol, 1.0 equiv.) and pinacol (0.47 g, 4 mmol, 1.0 equiv.). Final flash column chromatography (SiO_2; pentane – EtOAc; 97:3) afforded the title compound as a colorless oil (0.47 g, 2.6 mmol, 65 %).

Note: *n*butylboronic acid was prepared by treating $B(OiPr)_3$ (1.5 equiv.) in THF (0.15 M) with *n*BuLi (1.0 equiv.) at -20°C and final recrystallization from water.

^{1}H NMR (400 MHz, $CDCl_3$): δ (ppm) = 1.42 – 1.26 (m, 4H), 1.23 (s, 12H), 0.87 (t, J = 7.5 Hz, 3H), 0.76 (t, J = 7.5 Hz, 2H).
^{13}C NMR (100 MHz, $CDCl_3$): δ (ppm) = 82.96, 26.35, 25.56, 24.95, 14.04.

2-Butyl-4,4,5,5-tetraethyl-1,3,2-dioxaborolane (Si-3)

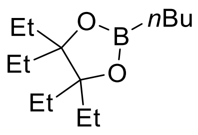

According to GP-G (Method A), using *n*butylboronic acid (0.70 g, 6.9 mmol, 1.0 equiv.) and 3,4-diethylhexane-3,4-diol (**L-2**) (0.99 g, 6.9 mmol, 1.0 equiv.). Final flash column chromatography (SiO_2; pentane – EtOAc; 98:2) afforded the title compound as a colorless oil (1.22 g, 5.1 mmol, 74%).

Note: *n*butylboronic acid was prepared by treating $B(OiPr)_3$ (1.5 equiv.) in THF (0.15 M) with *n*BuLi (1.0 equiv.) at -20°C and final recrystallization from water.

^{1}H NMR (400 MHz, $CDCl_3$): δ (ppm) = 1.65 (qd, *J* = 7.5, 5.8 Hz, 8H), 1.43 – 1.23 (m, 4H), 0.95 – 0.85 (m, 15H), 0.77 (t, *J* = 7.7 Hz, 2H).
^{13}C NMR (100 MHz, $CDCl_3$): δ (ppm) = 87.97, 26.58, 26.49, 25.62, 14.35, 14.07, 8.95.

12-Butyl-11,13-dioxa-12-boradispiro[4.0.46.35]tridecane (<u>Si-4</u>)

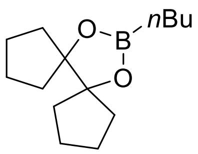

According to GP-G (Method A), using *n*butylboronic acid (0.41 g, 4.0 mmol, 1.0 equiv.) and [1,1'-bi(cyclopentane)]-1,1'-diol (**<u>L-3</u>**) (0.68 g, 4.0 mmol, 1.0 equiv.). Final flash column chromatography (SiO_2; pentane – EtOAc; 98:2) afforded the title compound as a colorless oil (0.44 g, 1.9 mmol, 48%).

<u>Note:</u> *n*butylboronic acid was prepared by treating $B(OiPr)_3$ (1.5 equiv.) in THF (0.15 M) with *n*BuLi (1.0 equiv.) at -20°C and final recrystallization from water.

^{1}H NMR (400 MHz, $CDCl_3$): δ (ppm) = 1.92 – 1.78 (m, 4H), 1.76 – 1.54 (m, 12H), 1.44 – 1.23 (m, 4H), 0.87 (t, J = 7.5 Hz, 3H), 0.76 (t, J = 7.5 Hz, 2H).
^{13}C NMR (100 MHz, $CDCl_3$): δ (ppm) = 92.97, 35.83, 26.49, 25.55, 23.16, 14.08.

2-Butyl-5,5-dimethyl-1,3,2-dioxaborinane (SI-5)

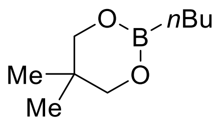

According to GP-G (Method A), using *n*butylboronic acid (0.41 g, 4.0 mmol, 1.0 equiv.) and neopentyl glycol (0.42 g, 4.0 mmol, 1.0 equiv.). Final flash column chromatography (SiO_2; pentane – EtOAc; 98:2) afforded the title compound as a colorless oil (0.45 g, 2.6 mmol, 66 %).

Note: *n*butylboronic acid was prepared by treating $B(OiPr)_3$ (1.5 equiv.) in THF (0.15 M) with *n*BuLi (1.0 equiv.) at -20°C and final recrystallization from water.

^{1}H NMR (400 MHz, $CDCl_3$): δ (ppm) = 3.58 (s, 4H), 1.39 – 1.18 (m, 4H), 0.94 (s, 6H), 0.87 (t, J = 7.1 Hz, 3H), 0.70 (t, J = 7.1 Hz, 2H).
^{13}C NMR (100 MHz, $CDCl_3$): δ (ppm) = 72.10, 35.01, 31.74, 26.51, 25.65, 21.96, 14.11.

2-Butyl-4,4,6,6-tetramethyl-1,3,2-dioxaborinane (Si-6)

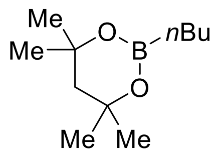

According to GP-G (Method A), using *n*butylboronic acid (0.41 g, 4.0 mmol, 1.0 equiv.) and 2,4-dimethylpentane-2,4-diol (**L-5**) (0.53 g, 4.0 mmol, 1.0 equiv.). Final flash column chromatography (SiO_2; pentane – EtOAc; 95:5) afforded the title compound as a colorless oil (0.48 g, 2.4 mmol, 59 %).

Note: *n*butylboronic acid was prepared by treating $B(OiPr)_3$ (1.5 equiv.) in THF (0.15 M) with *n*BuLi (1.0 equiv.) at -20°C and final recrystallization from water.

^{1}H NMR (400 MHz, $CDCl_3$): δ (ppm) = 1.78 (s, 2H), 1.32 (s, 12H), 1.30 – 1.23 (m, 4H), 0.86 (t, J = 7.1 Hz, 3H), 0.64 (t, J = 7.6 Hz, 2H).
^{13}C NMR (100 MHz, $CDCl_3$): δ (ppm) = 70.22, 48.94, 31.96, 26.77, 25.61, 14.19.

2-Butyl-5,5-dimethoxy-1,3,2-dioxaborinane (Si-7)

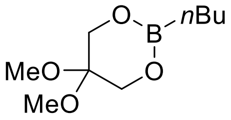

According to GP-G (Method B), using *n*butylboronic acid (0.41 g, 4.0 mmol, 1.0 equiv.) and 2,2-dimethoxypropane-1,3-diol (**L-6**) (0.54 g, 4.0 mmol, 1.0 equiv.). Final flash column chromatography (SiO_2; pentane – EtOAc; 97:3) afforded the title compound as a colorless oil (0.36 g, 1.8 mmol, 45%).

Note: *n*butylboronic acid was prepared by treating $B(OiPr)_3$ (1.5 equiv.) in THF (0.15 M) with *n*BuLi (1.0 equiv.) at -20°C and final recrystallization from water.

^{1}H NMR (400 MHz, $CDCl_3$): δ (ppm) = 3.87 (s, 4H), 3.28 (s, 6H), 1.39 – 1.22 (m, 4H), 0.87 (t, J = 7.1 Hz, 3H), 0.71 (t, J = 7.1 Hz, 2H).
^{13}C NMR (100 MHz, $CDCl_3$): δ (ppm) = 95.61, 64.60, 48.79, 26.42, 25.48, 14.10.

(6bR,9aS)-8-butyl-6b,9a-dimethyl-6b,9a-dihydroacenaphtho[1,2-d][1,3,2]dioxaborole (Si-8)

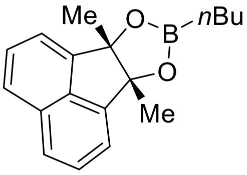

According to GP-G (Method A), using *n*butylboronic acid (0.41 g, 4.0 mmol, 1.0 equiv.) and *syn*-1,2-dimethyl-1,2-dihydroacenaphthylene-1,2-diol (**L-7**) (0.86 g, 4.0 mmol, 1.0 equiv.). Final flash column chromatography (SiO_2; pentane – EtOAc; 98:2) afforded the title compound as a colorless solid (0.71 g, 2.5 mmol, 63 %).

Note: *n*butylboronic acid was prepared by treating $B(OiPr)_3$ (1.5 equiv.) in THF (0.15 M) with *n*BuLi (1.0 equiv.) at -20°C and final recrystallization from water.

^{1}H NMR (400 MHz, $CDCl_3$): δ (ppm) = 7.79 (d, J = 8.6 Hz, 2H), 7.65 – 7.48 (m, 4H), 1.77 (s, 6H), 1.37 – 1.27 (m, 2H), 1.27 – 1.15 (m, 2H), 0.81 (t, J = 7.2 Hz, 3H), 0.72 (d, J = 7.9 Hz, 2H).
^{13}C NMR (100 MHz, $CDCl_3$): δ (ppm) = 145.02, 134.77, 131.47, 128.61, 125.35, 119.54, 91.71, 26.22, 25.49, 22.28, 13.95.

2-Butyl-4,4,5,5-tetraphenyl-1,3,2-dioxaborolane (Si-9)

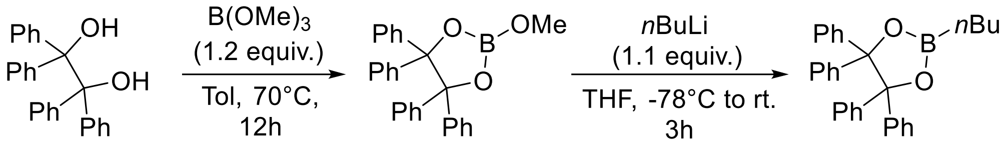

According to GP-G (Method C), using benzopinacol (1.83 g, 5.0 mmol, 1.0 equiv.) and $B(OMe)_3$ (0.67 mL, 6 mmol, 1.2 equiv.) in toluene (10 mL). After removal of excess $B(OMe)_3$ and toluene *in vacuo*, the crude methoxyester was dissolved in THF (30 mL) and cooled to -78°C. A solution of *n*BuLi in hexane (5.5 mmol, 1.1 equiv.) was added dropwise and the mixture was allowed to stir for 1h at that temperature. After warming to rt. for a further 2h, the reaction was quenched and extracted. Flash column chromatography (SiO_2; pentane – EtOAc; 95:5) afforded the title compound as a colorless viscous oil (1.4 g, 3.3 mmol, 65 %).

^{1}H NMR (400 MHz, $CDCl_2$): δ (ppm) = 7.19 – 7.12 (m, 8H), 7.10 – 7.05 (m, 12H), 1.70 – 1.62 (m, 2H), 1.51 – 1.45 (m, 2H), 1.27 – 1.23 (m, 2H), 0.97 (t, J = 7.3 Hz, 3H).
^{13}C NMR (100 MHz, $CDCl_2$): δ (ppm) = 143.28, 129.03, 127.72, 127.38, 96.18, 26.76, 26.08, 14.30.

2-Butyl-4,4,5,5-tetraisopropyl-1,3,2-dioxaborolane (<u>Si-10</u>)

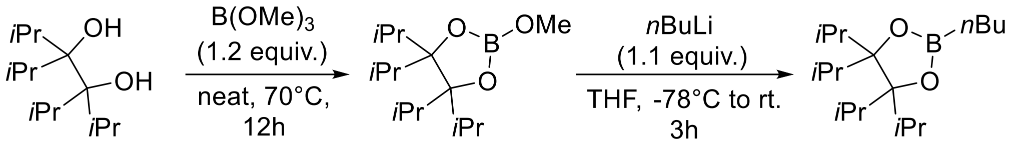

According to GP-G (Method C), using 3,4-diisopropyl-2,5-dimethylhexane-3,4-diol (**<u>L-9</u>**) (1.2 g, 5.0 mmol, 1.0 equiv.) and $B(OMe)_3$ (0.67 mL, 6 mmol, 1.2 equiv.) in toluene (10 mL). After removal of excess $B(OMe)_3$ and toluene *in vacuo*, the crude methoxyester was dissolved in THF (30 mL) and cooled to -78°C. A solution of *n*BuLi in hexane (5.5 mmol, 1.1 equiv.) was added dropwise and the mixture was allowed to stir for 1h at that temperature. After warming to rt. for a further 2h, the reaction was quenched and extracted. Flash column chromatography (SiO_2; pentane – EtOAc; 97:3) afforded the title compound as a colorless oil (0.5 g, 1.7 mmol, 34 %).

^{1}H NMR (400 MHz, $CDCl_3$): δ (ppm) = 2.19 (hept, J = 7.0 Hz, 4H), 1.41 – 1.24 (m, 4H), 1.01 – 0.87 (m, 24H), 0.83 (t, J = 7.1 Hz, 3H), 0.78 (t, J = 7.6 Hz, 2H).
^{13}C NMR (100 MHz, $CDCl_3$): δ (ppm) = 93.10, 31.73, 26.84, 25.63, 20.52, 19.56, 14.15.

2,4,4,5,5-Pentabutyl-1,3,2-dioxaborolane (Si-11)

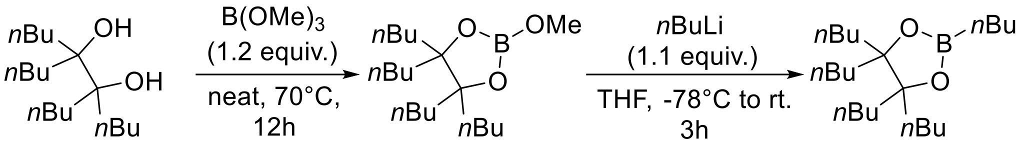

According to GP-G (Method C), using 5,6-dibutyldecane-5,6-diol (**L-10**) (0.9 g, 3.1 mmol, 1.0 equiv.) and $B(OMe)_3$ (0.41 mL, 3.7 mmol, 1.2 equiv.) in toluene (5 mL). After removal of excess $B(OMe)_3$ and toluene *in vacuo*, the crude methoxyester was dissolved in THF (30 mL) and cooled to -78°C. A solution of *n*BuLi in hexane (3.4 mmol, 1.1 equiv.) was added dropwise and the mixture was allowed to stir for 1h at that temperature. After warming to rt. for a further 2h, the reaction was quenched and extracted. Flash column chromatography (SiO_2; pentane – EtOAc; 99:1) afforded the title compound as a colorless oil (0.6 g, 1.6 mmol, 51 %).

^{1}H NMR (400 MHz, $CDCl_3$): δ (ppm) = 1.61 – 1.52 (m, 8H), 1.42 – 1.19 (m, 20H), 0.95 – 0.85 (m, 15H), 0.76 (t, J = 7.6 Hz, 2H).
^{13}C NMR (100 MHz, $CDCl_3$): δ (ppm) = 87.68, 34.50, 26.58, 25.58, 23.58, 14.19.

(4*R*,6*R*,7a*S*)-2-Butyl-5,5,7a-trimethylhexahydro-4,6-methanobenzo[d][1,3,2]dioxaborole (Si-12)

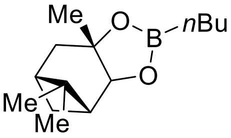

According to GP-G (Method A), using *n*butylboronic acid (0.2 g, 2 mmol, 1.0 equiv.) and (1*R*,2*R*,3*S*,5*R*)-(−)-Pinanediol (0.34 g, 2 mmol, 1.0 equiv.). Final flash column chromatography (SiO_2; pentane/ EtOAc – 96:4) afforded the title compound as a colorless oil (0.32 g, 1.3 mmol, 67 %).

Note: *n*butylboronic acid was prepared by treating $B(OiPr)_3$ (1.5 equiv.) in THF (0.15 M) with *n*BuLi (1.0 equiv.) at -20°C and final recrystallization from water.

^{1}H NMR (400 MHz, $CDCl_3$): δ (ppm) = 4.24 (dd, J = 8.6, 1.8 Hz, 1H), 2.37 – 2.29 (m, 1H), 2.24 – 2.16 (m, 1H), 2.04 (t, J = 5.6 Hz, 1H), 1.93 – 1.80 (m, 2H), 1.43 – 1.26 (m, 10H), 1.11 (d, J = 10.9 Hz, 1H), 0.91 – 0.77 (m, 8H).
^{13}C NMR (100 MHz, $CDCl_3$): δ (ppm) = 85.40, 51.40, 39.66, 38.25, 35.71, 28.84, 27.22, 26.51, 25.61, 24.15, 14.06.

Ritter's trifluoroiodomethane-DMSO complex (Si-13)

$$CF_3I \xrightarrow[\text{neat, -78°C to rt. 30 min}]{\text{DMSO (2 equiv.)}} CF_3I \cdot 2DMSO$$

According to a literature procedure[6], Trifluoromethyl iodide was condensed in a pre-weighted and dry Schlenk-flask at -78°C. After determining the amount of CF_3I, dry DMSO (3.3 mL, 46 mmol, 2.0 equiv.) was added dropwise at -78°C. The reaction mixture was allowed to warm to rt. and the flask was sealed, wrapped with aluminum foil and stored at -18°C.

^{1}H NMR (600 MHz, $CDCl_3$): δ (ppm) = 2.50 (s, 12H).
^{13}C NMR (150 MHz, $CDCl_3$): δ (ppm) = 40.74.
^{19}F NMR (400 MHz, $CDCl_3$): δ (ppm) = -5.68.

2-Iodo-1-phenylethan-1-one (Si-14)

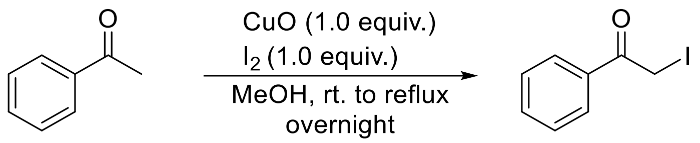

According to a literature procedure[7], Acetophenone (2.3 mL, 20 mmol, 1.0 equiv.) was dissolved in dry MeOH (50 mL) followed by addition of CuO (1.6 g, 20 mmol, 1.0 equiv.) and I_2 (5.1 g, 20 mmol, 1.0 equiv.). After stirring for 5 min, the solution was heated to reflux overnight. The next day, the solvent was removed *in vacuo*, and sat. aq. $Na_2S_2O_3$ (100 mL) was added. The suspension was extracted with EtOAc (3 × 100 mL), the combined organic fractions were washed with Brine and concentrated *in vacuo*. Flash column chromatography (SiO_2; pentane – EtOAc; 9:1) afforded the title compound as red crystals (4.3, 18 mmol, 88%).

^{1}H NMR (400 MHz, $CDCl_3$): δ (ppm) = 8.01 – 7.97 (m, 2H), 7.63 – 7.58 (m, 1H), 7.52 – 7.46 (m, 2H), 4.36 (s, 2H).
^{13}C NMR (100 MHz, $CDCl_3$): δ (ppm) = 192.96, 133.96, 129.16, 128.96, 1.85.

Boronic Esters:

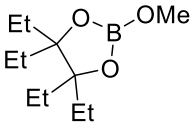

Si-15

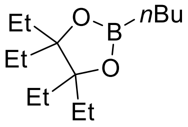

Si-3

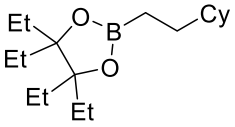

Si-16

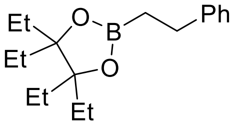

Si-17

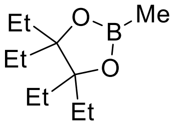

Si-18

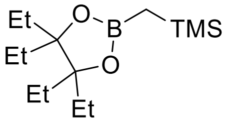

Si-19

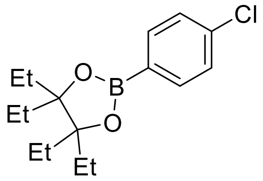

Si-20

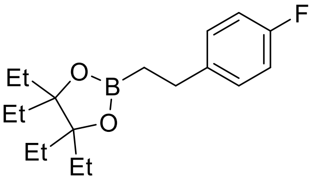

Si-21

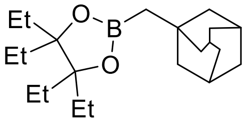

Si-22

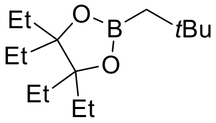

Si-23

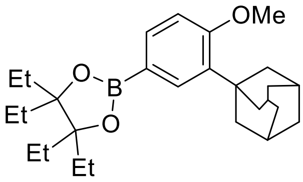

Si-24

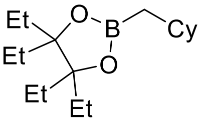

Si-25

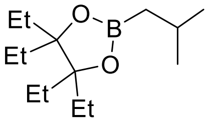

Si-26

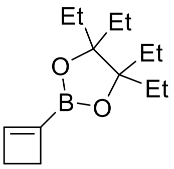

4

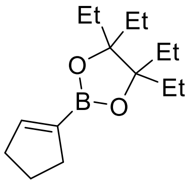

7

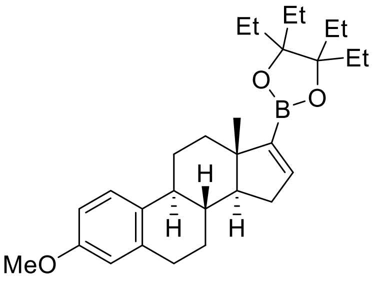

Si-27

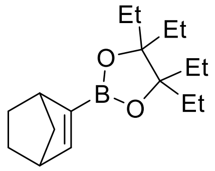

13

4,4,5,5-Tetraethyl-2-methoxy-1,3,2-dioxaborolane (MeO-B(Epin)) (Si-15)

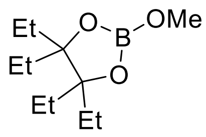

According to GP-G (Method C) 3,4-diethylhexane-3,4-diol (8.7 g, 50 mmol, 1.0 equiv.) was reacted with $B(OMe)_3$ (6.7 mL, 60 mmol, 1.2 equiv.). Distillation of the crude mixture afforded the title compound as a colorless oil (10.3 g, 48 mmol, 96%). Boiling Point (74°C | 4 × 10^{-1} mbar).

^{1}H NMR (400 MHz, $CDCl_3$): δ (ppm) = 3.60 (s, 3H), 1.77 – 1.57 (m, 8H), 0.91 (t, *J* = 7.5 Hz, 12H).

^{13}C NMR (100 MHz, $CDCl_3$): δ (ppm) = 87.87, 52.76, 26.38, 8.82.

2-(2-Cyclohexylethyl)-4,4,5,5-tetraethyl-1,3,2-dioxaborolane (Si-16)

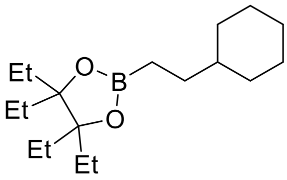

According to GP-G (Method A), using (2-cyclohexylethyl)boronic acid (0.62 g, 4.0 mmol, 1.0 equiv.) and subsequent flash column chromatography (pentane/ EtOAc – 97:3) afforded the title compound as a colorless oil (0.95 g, 3.2 mmol, 81 %).

Note: (2-cyclohexylethyl)boronic acid was prepared by treating $B(OiPr)_3$ (1.5 equiv.) 0.15 M in THF with freshly prepared (2-cyclohexylethyl)magnesium bromide with at -20°C and final recrystallization from water.

^{1}H NMR (600 MHz, $CDCl_3$): δ (ppm) = 1.74 – 1.59 (m, 13H), 1.31 – 1.26 (m, 2H), 1.22 – 1.09 (m, 4H), 0.90 (t, J = 7.5 Hz, 12H), 0.83 (td, J = 12.0, 3.2 Hz, 2H), 0.78 – 0.74 (m, 2H).
^{13}C NMR (150 MHz, $CDCl_3$): δ (ppm) = 87.97, 40.09, 33.17, 31.76, 26.94, 26.63, 26.47, 8.96.

4,4,5,5-Tetraethyl-2-phenethyl-1,3,2-dioxaborolane (Si-17)

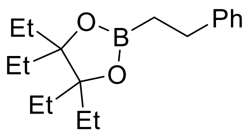

According to GP-G (Method A), using phenethylboronic acid (0.75 g, 5.0 mmol, 1.0 equiv.) and subsequent flash column chromatography (pentane/ EtOAc – 96:4) afforded the title compound as a colorless oil (0.98 g, 3.4 mmol, 68 %).

Note: phenethylboronic acid was prepared by treating freshly prepared phenethyl magnesium bromide with $B(OMe)_3$ (1.5 equiv.) in THF (0.15 M) at -20°C.

^{1}H NMR (600 MHz, $CDCl_3$): δ (ppm) = δ 7.26 – 7.23 (m, 2H), 7.23 – 7.20 (m, 2H), 7.16 – 7.13 (m, 1H), 2.77 – 2.73 (m, 2H), 1.63 (qd, J = 7.4, 4.5 Hz, 8H), 1.17 – 1.13 (m, 2H), 0.88 (t, J = 7.5 Hz, 12H).
^{13}C NMR (150 MHz, $CDCl_3$): δ (ppm) = 144.68, 128.27, 125.55, 88.25, 30.21, 26.44, 8.93.

4,4,5,5-Tetraethyl-2-methyl-1,3,2-dioxaborolane (Si-18)

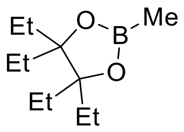

According to GP-G (Method A), using commercially available methylboronic acid (0.24 g, 4.0 mmol, 1.0 equiv.) and subsequent flash column chromatography (pentane/ EtOAc – 98:2) afforded the title compound as a colorless oil (0.98 g, 3.4 mmol, 54 %).

^{1}H NMR (400 MHz, $CDCl_3$): δ (ppm) = 1.65 (qd, J = 7.5, 5.9 Hz, 8H), 0.91 (t, J = 7.5 Hz, 12H), 0.25 (s, 3H).
^{13}C NMR (100 MHz, $CDCl_3$): δ (ppm) = 88.19, 26.56, 8.95.

Trimethyl((4,4,5,5-tetraethyl-1,3,2-dioxaborolan-2-yl)methyl)silane (Si-19)

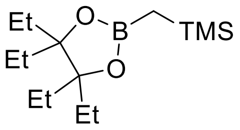

According to GP-G (Method C), by quenching commercially available (trimethylsilyl)methyl magnesium chloride (3 mmol, 1.0 equiv.) in THF with MeO-B(Epin) (0.96 g, 4.5 mmol, 1.5 equiv.). Subsequent flash column chromatography (SiO_2; pentane/ EtOAc – 98:2) afforded the title compound as a colorless oil (0.65 g, 2.5 mmol, 82%).

^{1}H NMR (400 MHz, $CDCl_3$): δ (ppm) = 1.64 (qd, J = 7.4, 3.1 Hz, 8H), 0.90 (t, J = 7.5 Hz, 21H), 0.08 (s, 2H), 0.04 (s, 9H).
^{13}C NMR (100 MHz, $CDCl_3$): δ (ppm) = 87.92, 32.02, 29.73, 26.41, 8.96.

2-(4-chlorophenyl)-4,4,5,5-tetraethyl-1,3,2-dioxaborolane (Si-20)

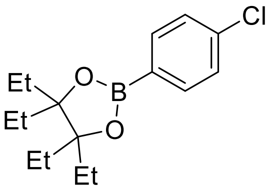

According to GP-G (Method A), using commercially available (4-chlorophenyl)boronic acid (0.63 g, 4.0 mmol, 1.0 equiv.) and subsequent flash column chromatography (pentane/ EtOAc – 96:4) afforded the title compound as a colorless solid (0.99 g, 3.4 mmol, 84 %).

^{1}H NMR (400 MHz, $CDCl_3$): δ (ppm) = 7.77 – 7.72 (m, 2H), 7.37 – 7.31 (m, 2H), 1.84 – 1.66 (m, 8H), 0.96 (t, J = 7.5 Hz, 12H).
^{13}C NMR (100 MHz, $CDCl_3$): δ (ppm) = 137.48, 136.31, 128.11, 89.14, 26.56, 8.98.

4,4,5,5-Tetraethyl-2-(4-fluorophenethyl)-1,3,2-dioxaborolane (Si-21)

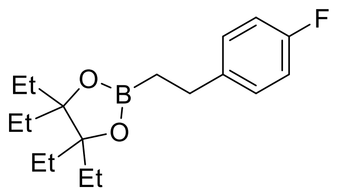

According to GP-G (Method A), using (4-fluorophenethyl)boronic acid (0.67 g, 4.0 mmol, 1.0 equiv.) and subsequent flash column chromatography (pentane/ EtOAc – 98:2) afforded the title compound as a colorless oil (0.53 g, 1.7 mmol, 43 %).

Note: (4-fluorophenethyl)boronic acid was prepared by treating freshly prepared (4-fluorophenethyl) magnesium bromide with $B(OMe)_3$ (1.5 equiv.) in THF (0.15 M) at -20°C.

^{1}H NMR (400 MHz, $CDCl_3$): δ (ppm) = 7.20 – 7.12 (m, 2H), 6.98 – 6.88 (m, 2H), 2.72 (t, J = 8.1 Hz, 2H), 1.68 – 1.56 (m, 8H), 1.15 – 1.10 (m, 2H), 0.88 (t, J = 7.5 Hz, 12H).
^{13}C NMR (100 MHz, $CDCl_3$): δ (ppm) = 129.46, 129.39, 115.00, 114.79, 88.32, 29.43, 26.47, 8.92.

2-(Adamantan-1-yl)methyl)-4,4,5,5-tetraethyl-1,3,2-dioxaborolane (Si-22)

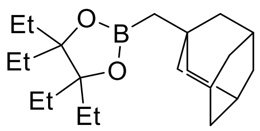

According to GP-G (Method C), by quenching (adamantan-1-yl)methyl)magnesium chloride (3 mmol, 1.0 equiv.) in THF with MeO-B(Epin) (0.96 g, 4.5 mmol, 1.5 equiv.). Subsequent flash column chromatography (pentane/ EtOAc – 98:2) afforded the title compound as a colorless solid (0.70 g, 2.1 mmol, 71 %).

Note: (adamantan-1-yl)methyl)magnesium chloride was prepared in THF (2 M) at 60°C. Activation of Mg-turnings with 1,2-dibromoethane.

^{1}H NMR (400 MHz, $CDCl_3$): δ (ppm) = 1.94 – 1.87 (m, 3H), 1.72 – 1.61 (m, 12H), 1.57 (d, *J* = 2.9 Hz, 6H), 0.91 (t, *J* = 7.5 Hz, 12H), 0.65 (s, 2H).

^{13}C NMR (100 MHz, $CDCl_3$): δ (ppm) = 87.91, 44.82, 37.14, 31.79, 29.21, 26.37, 8.99.

4,4,5,5-Tetraethyl-2-neopentyl-1,3,2-dioxaborolane (Si-23)

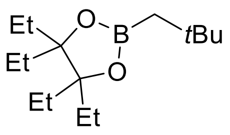

According to GP-G (Method C), by quenching neopentylmagnesium bromide (3 mmol, 1.0 equiv.) in THF with MeO-B(Epin) (0.96 g, 4.5 mmol, 1.5 equiv.). Subsequent flash column chromatography (SiO_2; pentane/ EtOAc – 99:1) afforded the title compound as a colorless oil (0.67 g, 2.6 mmol, 88%).

Note: neopentylmagnesium bromide was prepared in THF (1 M) at 25°C. Activation of Mg-turnings with 1,2-dibromoethane.

^{1}H NMR (400 MHz, $CDCl_3$): δ (ppm) = 1.65 (qd, J = 7.5, 5.9 Hz, 8H), 0.98 (s, 9H), 0.91 (t, J = 7.5 Hz, 12H), 0.81 (s, 2H).
^{13}C NMR (100 MHz, $CDCl_3$): δ (ppm) = 87.92, 32.02, 29.73, 26.41, 8.96.

2-(3-(Adamantan-1-yl)-4-methoxyphenyl)-4,4,5,5-tetraethyl-1,3,2-dioxaborolane (Si-24)

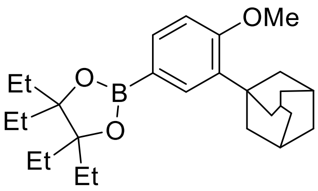

According to GP-G (Method C), by quenching (3-(adamantan-1-yl)-4-methoxyphenyl)magnesium bromide (1.9 mmol, 1.0 equiv.) in THF with MeO-B(Epin) (0.6 g, 2.9 mmol, 1.5 equiv.). Subsequent flash column chromatography (SiO_2; pentane/ EtOAc – 95:5) afforded the title compound as a colorless solid (0.77 g, 1.8 mmol, 96%).

Note: 3-(adamantan-1-yl)-4-methoxyphenyl)magnesium bromide was prepared in THF (1 M) at 25°C. Activation of Mg-turnings with 1,2-dibromoethane.

^{1}H NMR (400 MHz, $CDCl_3$): δ (ppm) = 7.70 – 7.64 (m, 2H), 6.89 – 6.84 (m, 1H), 3.85 (s, 3H), 2.17 – 2.04 (m, 10H), 1.84 – 1.68 (m, 13H), 0.97 (t, J = 7.5 Hz, 12H).
^{13}C NMR (100 MHz, $CDCl_3$): δ (ppm) = 137.65, 134.43, 133.15, 111.09, 88.50, 54.97, 40.60, 37.32, 29.28, 26.55, 9.05.

2-(Cyclohexylmethyl)-4,4,5,5-tetraethyl-1,3,2-dioxaborolane (Si-25)

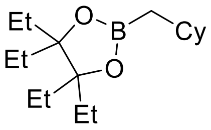

According to GP-G (Method C), by quenching (cyclohexylmethyl)magnesium bromide (4 mmol, 1.0 equiv.) in THF with MeO-B(Epin) (1.3 g, 6 mmol, 1.5 equiv.). Subsequent flash column chromatography (SiO_2; pentane/ EtOAc – 98:2) afforded the title compound as a colorless oil (0.63 g, 2.2 mmol, 56%).

Note: (cyclohexylmethyl)magnesium bromide was prepared in THF (1 M) at 25°C. Activation of Mg-turnings with 1,2-dibromoethane.

^{1}H NMR (400 MHz, $CDCl_3$): δ (ppm) = 1.73 – 1.58 (m, 14H), 1.52 – 1.44 (m, 1H), 1.29 – 1.05 (m, 4H), 0.90 (t, J = 7.5 Hz, 12H), 0.71 (d, J = 7.1 Hz, 2H).
^{13}C NMR (100 MHz, $CDCl_3$): δ (ppm) = 87.98, 36.03, 34.47, 33.64, 26.94, 26.75, 26.62, 26.48, 26.38, 8.95.

4,4,5,5-Tetraethyl-2-isobutyl-1,3,2-dioxaborolane (Si-26)

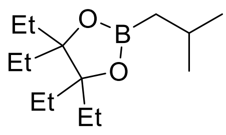

According to GP-G (Method A), using commercially available *i*butylboronic acid (0.41 g, 4.0 mmol, 1.0 equiv.) and subsequent flash column chromatography (pentane/ EtOAc – 98:2) afforded the title compound as a colorless oil (0.74 g, 3.1 mmol, 77 %).

^{1}H NMR (400 MHz, $CDCl_3$): δ (ppm) = 1.85 (dt, J = 13.5, 6.7 Hz, 1H), 1.74 – 1.57 (m, 8H), 0.98 – 0.84 (m, 18H), 0.73 (d, J = 7.1 Hz, 2H).

^{13}C NMR (100 MHz, $CDCl_3$): δ (ppm) = 87.98, 26.40, 25.36, 25.03, 8.95.

2-(Cyclobut-1-en-1-yl)-4,4,5,5-tetraethyl-1,3,2-dioxaborolane (4)

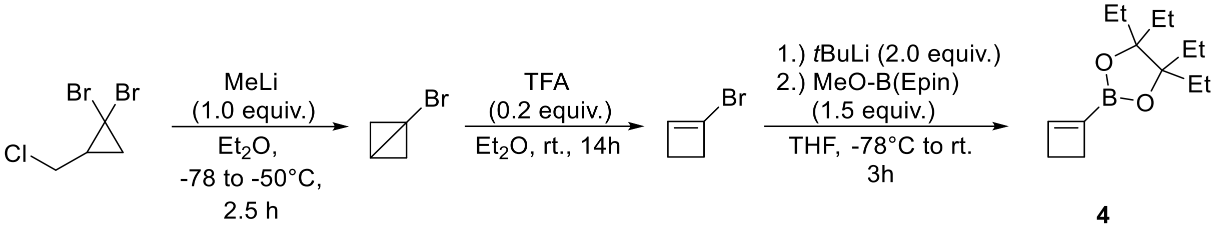

According to a literature procedure[8], a flame-dried Schlenk flask was charged with 1,1-dibromo-2-(chloromethyl)cyclopropane (4.83 g, 19.4 mmol, 1.00 equiv.) and dry Et_2O (15 mL). A solution of Methyllithium in Et_2O (19.4 mmol, 1.00 equiv.) was added dropwise at -78°C and the dark solution was allowed to stir for 30 min at that temperature. The dry-ice acetone bath was allowed to warm to -50°C and the solvent was removed *in vacuo* using a cooling trap cooled to -78°C. To the colorless solution was added TFA (0.3 mL, 4 mmol, 0.2 equiv.) and the mixture was allowed to stir overnight under nitrogen. The next day, the organic phase was washed with sat. aq. $NaHCO_3$ (30 mL), sat. aq. $NaHSO_3$ (2 × 30 mL), Brine (50 mL) and dried over anhydrous $MgSO_4$. After concentration of the solution, the molarity was determined by NMR-titration using Mesitylene as standard.

The degassed stock solution of 1-bromocyclobut-1-ene (4.9 mmol, 1.0 equiv.) was transferred in a flame-dried flask and dry THF (30 mL) was added. After cooling the solution to -78°C *t*BuLi in pentane (9.8 mmol, 2.0 equiv.) was added dropwise *via* syringe-pump (0.3 mL/min) and the mixture was allowed to stir at that temperature for 1h. Subsequently 4,4,5,5-tetraethyl-2-methoxy-1,3,2-dioxaborolane (2.1 g, 9.8 mmol, 2.0 equiv.) was added dropwise and the solution was allowed to stir at -78°C for 1h before warming to rt. After hydrolysis with 2 M HCl (5 mL) the mixture was extracted with EtOAc (3 × 30 mL) and the combined organic fractions were washed with Brine and dried over anhydrous $MgSO_4$. Evaporation of the solvent *in vacuo* and subsequent flash column chromatography (pentane/ EtOAc – 99:1 to 97: 3 afforded the title compound as a colorless oil (0.83 g, 3.5 mmol, 72 %).

^{1}H NMR (400 MHz, $CDCl_3$): δ (ppm) = 6.81 (t, J = 1.0 Hz, 1H), 2.65 – 2.59 (m, 4H), 1.76 – 1.58 (m, 8H), 0.91 (t, J = 7.5 Hz, 12H).

^{13}C NMR (100 MHz, $CDCl_3$): δ (ppm) = 153.28, 88.33, 32.19, 31.39, 26.48, 8.97.

4,4,5,5-Tetraethyl-2-((8*S*,9*S*,13*S*,14*S*)-3-methoxy-13-methyl-7,8,9,11,12,13,14,15-octahydro-6H-cyclopenta[a]phenanthren-17-yl)-1,3,2-dioxaborolane (Si-27)

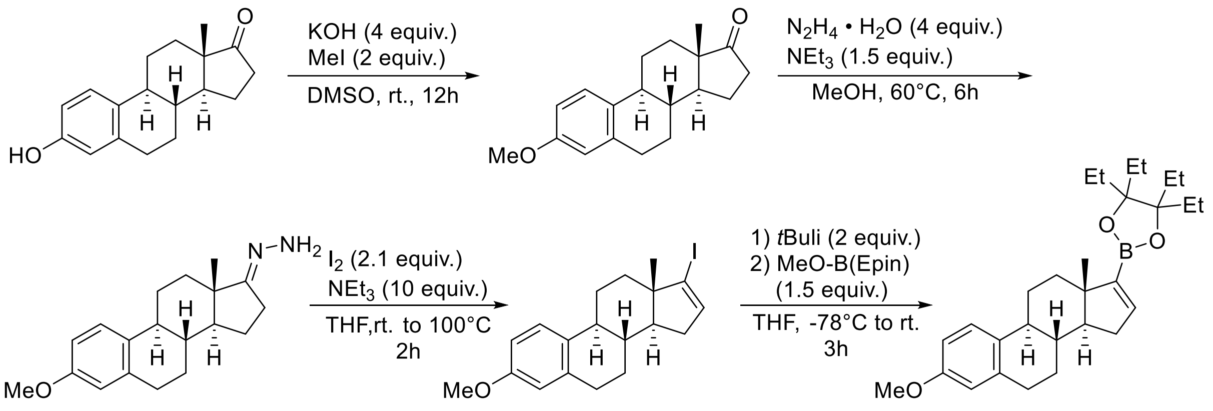

Estrone (2.0 g, 7.4 mmol, 1.0 equiv.) was charged to a solution of KOH (1.7 g, 30 mmol, 4 equiv.) in DMSO (20 mL). MeI (0.92 mL,15 mmol, 2.0 equiv.) was added at room temperature and the reaction mixture was stirred overnight. The colorless precipitate was collected by filtration, washed with water (50 mL) and dried *in vacuo,* to afford (8*R*,9*S*,13*S*,14*S*)-3-methoxy-13-methyl-6,7,8,9,11,12,13,14,15,16-decahydro-17H-cyclopenta[a]phenanthren-17-one as a colorless solid (1.7 g, 6.1 mmol, 83 %).[9]

The full amount of the obtained compound was directly employed in further synthesis. Hydrazine monohydrate (1.2 mL, 24 mmol, 4.0 equiv.) was added to a solution of X in MeOH (30 mL), followed by Triethylamine (1.3 mL, 9.2 mmol, 1.5 equiv.). The reaction mixture was heated to reflux for 6h, cooled to rt. and the volatiles were removed *in vacuo* in a well ventilated hood. Crude (E)-((8R,9S,13S,14S)-3-methoxy-13-methyl-6,7,8,9,11,12,13,14,15,16-decahydro-17H-cyclopenta[a]phenanthren-17-ylidene)hydrazine was obtained as a light yellow solid (1.78 g, 6.0 mmol, 98%).[9]

The full amount of the crude Hydrazone was dissolved in THF (60 mL) and NEt_3 (8.4 mL, 60 mmol, 10 equiv.) was added, followed by dropwise addition of a solution of I_2 (3.3 g, 13 mmol, 2.1 equiv.) in THF (30 mL). Subsequently, the solvent was removed *in vacuo* and Pyridine (25 mL) was added. The reaction mixture was refluxed for 1h, the cooled to rt. and dissolved in EtOAc (100 mL). The organic fraction was washed with HCl (2 M, 50 mL), Na_2SO_3 (50 mL), Water, Brine and was dried over anhydrous $MgSO_4$. After removal of the solvents *in vacuo* the residue was purified via flash column chromatography (SiO_2; Pentane – EtOAc: 99/1 to 97/3). (8R,9S,13S,14S)-17-Iodo-3-methoxy-13-methyl-7,8,9,11,12,13,14,15-octahydro-6H-cyclopenta[a]phenanthrene was obtained as colorless crystals (1.3 g, 3.2 mmol, 54%).[10]

^{1}H NMR (400 MHz, $CDCl_3$): δ (ppm) = 7.20 (dd, J = 8.7, 1.1 Hz, 1H), 6.72 (dd, J = 8.7, 2.8 Hz, 1H), 6.65 (t, J = 1.8 Hz, 1H), 6.17 (dd, J = 3.3, 1.7 Hz, 1H), 3.78 (s, 3H), 2.97 – 2.83 (m, 2H), 2.45 – 2.36 (m, 1H), 2.32 – 2.21 (m, 2H), 2.11 – 1.98 (m, 1H), 1.98 – 1.89 (m, 1H), 1.80 – 1.37 (m, 6H), 0.77 (s, 3H).

^{13}C NMR (100 MHz, $CDCl_3$): δ (ppm) = 157.62, 137.94, 137.54, 132.59, 126.20, 113.94, 112.83, 111.57, 55.35, 54.15, 50.42, 44.28, 37.93, 36.40, 33.59, 29.81, 27.59, 26.52, 15.42.

The Vinyl iodide (0.78 g, 2.0 mmol, 1.0 equiv.) was dissolved in dry THF (40 mL) and cooled to -78°C. *t*BuLi in pentane (4.0 mmol, 2.0 equiv.) was added dropwise over 15 min and the reaction mixture was allowed to stir at that temperature for 45 min. MeO-B(Epin) (0.64 g, 3.0 mmol, 1.5 equiv.) was added dropwise to the solution and stirring at -78°C was continued for 1h before warming to rt. The reaction was quenched by addition of HCl (1 M, 2 mL) and stirred for 30 min. before being extracted with EtOAc (3 × 40 mL). The combined organic fractions were washed with Brine, dried over anhydrous $MgSO_4$ and concentrated *in vacuo*. Flash-column chromatography (SiO_2; Pentane – EtOAc: 99/1 to 97/3; long column) afforded the title compound as a light yellow oil (0.65 g, 1.4 mmol, 72%)

^{1}H NMR (400 MHz, $CDCl_3$): δ (ppm) = 7.24 – 7.18 (m, 1H), 6.72 (dt, J = 8.6, 2.6 Hz, 1H), 6.64 (t, J = 3.0 Hz, 1H), 6.53 (dd, J = 3.1, 1.6 Hz, 1H), 3.78 (d, J = 0.9 Hz, 3H), 2.99 – 2.80 (m, 2H), 2.43 – 2.18 (m, 4H), 2.11 – 2.01 (m, 2H), 1.98 – 1.88 (m, 1H), 1.78 – 1.38 (m, 12H), 1.00 – 0.89 (m, 8H), 0.85 – 0.74 (m, 3H).

^{13}C NMR (100 MHz, $CDCl_3$): δ (ppm) = 157.45, 145.72, 144.11, 138.16, 137.94, 137.54, 133.43, 133.24, 132.58, 129.41, 126.23, 113.90, 112.83, 111.47, 87.84, 60.55, 56.03, 55.33, 54.16, 50.42, 48.18, 45.92, 44.60, 44.29, 37.94, 37.71, 37.47, 36.41, 36.07, 33.44, 29.97, 28.41, 28.12, 27.59, 26.92, 26.49, 21.22, 17.22, 17.04, 15.42, 14.35, 9.00.

2-(bicyclo[2.2.1]hept-2-en-2-yl)-4,4,5,5-tetraethyl-1,3,2-dioxaborolane (13)

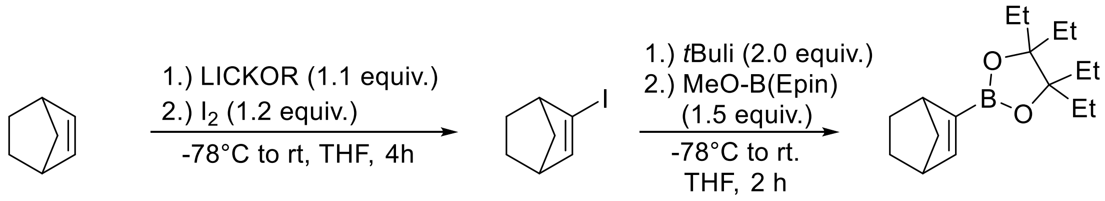

According to a modified literature procedure[11], a flame-dried flask was charged with norbornene (1.9 g, 20 mmol, 1.0 equiv.), KO*t*Bu (2.5 g, 1.1 equiv.) and dry THF (20 mL) was added. After cooling to -78°C, *n*BuLi (22 mmol, 1.1 equiv.) was added *via* syringe-pump (0.3 mL/min). The reaction mixture was allowed to stir at the aforementioned temperature for 1h and was then allowed to warm to -50°C for 30 min. After cooling back to -78°C, a solution of I_2 (6.1 g, 24 mmol, 1.2 equiv.) in THF (10 mL) was added via syringe-pump (0.9 mL/min). After warming to rt. for 1h, the reaction was quenched with sat. aq. NH_4Cl (10 mL). EtOAc (50 mL) was added and the organic phase was seperated and washed with sat. aq. $Na_2S_2O_3$ (2 × 50 mL). Organic extracts were washed with Brine, dried over $MgSO_4$ and concentrated *in vacuo*. Flash column chromatography (SiO_2; pentane) afforded 2-iodobicyclo[2.2.1]hept-2-ene as a colorless oil (1.9 g, 8.6 mmol, 43%).

^{1}H NMR (400 MHz, $CDCl_3$): δ (ppm) = 6.36 (d, J = 3.2 Hz, 1H), 2.96 – 2.93 (m, 1H), 2.81 – 2.77 (m, 1H), 1.64 – 1.52 (m, 3H), 1.14 – 1.04 (m, 3H).
^{13}C NMR (100 MHz, $CDCl_3$): δ (ppm) = 144.28, 96.78, 53.54, 48.24, 44.90, 25.86, 24.30.

A flame-dried schlenk flask was charged with 2-iodobicyclo[2.2.1]hept-2-ene (0.65 g, 2.9 mmol, 1.0 equiv.) and dry THF (30 mL) was added. The solution was cooled to -78°C and *t*BuLi (5.8 mmol, 2.0 equiv.) was added dropwise. The yellow reaction mixture was allowed to stir for 30min at -78°C before MeO-B(Epin) (0.93 g, 4.4 mmol, 1.5 equiv.) was added dropwise. After stirring for a further 30min at -78°C the now colorless solution was allowed to warm to rt. for 1h before quenching with sat. aq. NH_4Cl (5 mL). The organic phase was seperated and the aqeous fraction extracted with EtOAc (3 × 30 mL). The combined organic fractions were washed with Brine, dried over $MgSO_4$ and concentrated *in vacuo*. Final flash column chromatography (SiO_2; pentane – EtOAc: 98:2) afforded the title compound as a colorless oil. (0.64 g, 2.3 mmol, 79%)

^{1}H NMR (400 MHz, $CDCl_3$): δ (ppm) = 6.77 (d, J = 2.9 Hz, 1H), 3.07 – 3.04 (m, 1H), 2.90 – 2.87 (m, 1H), 1.72 – 1.60 (m, 11H), 0.93 – 0.89 (m, 15H).
^{13}C NMR (100 MHz, $CDCl_3$): δ (ppm) = δ 151.24, 88.01, 48.54, 43.48, 27.33, 26.57, 26.47, 24.75, 24.22, 9.06, 8.94.

Overview: trisubstituted Azetidines:

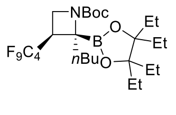

3a (NOESY)
dr = 8:1

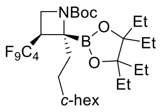

3b
dr = 8:1

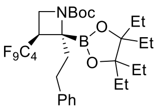

3c
dr = 10:1

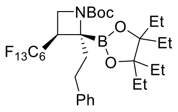

3d
dr = >20:1

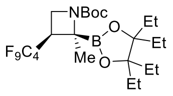

3e
dr = 1,5:1

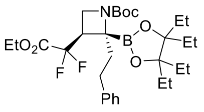

3f
dr = >20:1

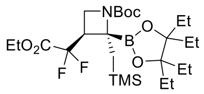

3g
dr = >20:1

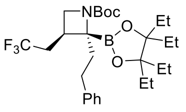

3h
dr = 7:1

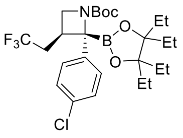

3i
dr = 4:1

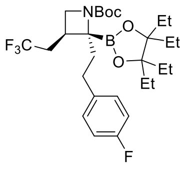

3j
dr = 7:1

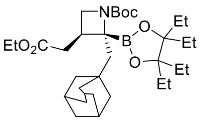

3k
dr = >20:1

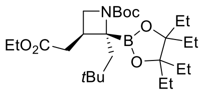

3l
dr = >20:1

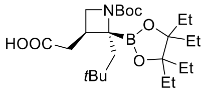

3l'
dr = >20:1

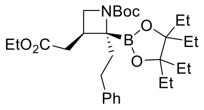

3m
dr = >20:1

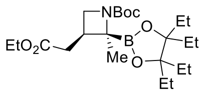

3n
dr = 2:1

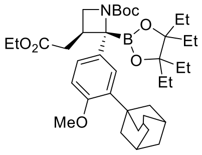

3o
dr = 2:1

tert-Butyl (2_R_,3_S_)-2-butyl-3-(perfluorobutyl)-2-(4,4,5,5-tetraethyl-1,3,2-dioxaborolan-2-yl)azetidine-1-carboxylate (3a)

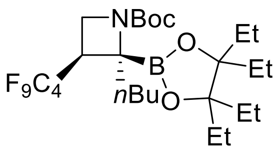

According to GP-A, using 2-butyl-4,4,5,5-tetraethyl-1,3,2-dioxaborolane **Si-3** (94 mg, 0.39 mmol, 1.3 equiv.) and nonafluoro-1-iodobutane (0.15 mL, 0.9 mmol, 3.0 equiv.). Purification *via* flash-column chromatography (SiO_2; Pentane – EtOAc: 99:1 to 98:2) yielded the title compound as a colorless oil (0.17 g, 0.28 mmol, 93 %). ^{19}F NMR analysis of the crude reaction mixture indicated a diastereomeric ratio of 8:1. The title compound was obtained in a mixture of *N*-Boc Rotamers in a ratio of 1:1, as indicated by ^{1}H NMR.

^{1}H NMR (600 MHz, Acetone-D$_6$): δ (ppm) = 3.97 – 3.89 (m, 1H), 3.85 (d, J = 8.6 Hz, 1H), 3.40 – 3.27 (m, 1H), 1.92 – 1.84 (m, 2H), 1.82 – 1.68 (m, 8H), 1.67 – 1.55 (m, 2H), 1.43 (d, J = 6.0 Hz, 9H), 1.39 – 1.33 (m, 2H), 0.97 – 0.87 (m, 15H).
^{13}C NMR (150 MHz, Acetone-D$_6$): δ (ppm) = 155.51, 154.75, 118.31, 117.04, 116.40, 89.56, 89.50, 78.88, 78.65, 46.43, 46.40, 46.37, 44.95, 44.91, 44.88, 38.04, 37.67, 37.51, 37.36, 37.33, 37.17, 37.02, 36.90, 27.76, 27.72, 27.63, 25.68, 25.67, 25.35, 25.25, 25.14, 25.00, 22.89, 22.88, 13.51, 13.47, 8.44, 8.24, 7.89, 7.63.
^{19}F NMR (400 MHz, Acetone-D$_6$): δ (ppm) = -81.88, -81.89, -81.91, -81.93, -81.94, -81.95, -81.96, -81.97, -112.98, -113.16, -113.70, -113.89, -115.77, -115.80, -115.84, -115.88, -115.91, -115.96, -116.46, -116.49, -116.53, -116.56, -116.60, -116.64, -117.22, -117.25, -117.29, -117.32, -117.35, -123.25, -123.56, -124.04, -124.31, -124.33, -124.34, -124.35, -124.37, -124.38, -124.72.

HRMS (ESI) m/z: $[H]^+$ calcd for $C_{26}H_{41}BF_9NO_4H^+$: 614.3065 found: 614.3072
IR (Diamond-ATR, neat) $\tilde{\nu}_{max}$: 2934 (m), 2900 (w), 1732 (w), 1702 (s), 1458 (m), 1388 (s), 1362 (s), 1314 (m), 1290 (m), 1232 (vs), 1158 (s), 1134 (vs), 1112 (s), 1094 (m), 1066 (m), 1052 (m), 1026 (m), 966 (w), 922 (s), 890 (w), 854 (m), 800 (w), 776 (w), 744 (w), 726 (m), 680 (vw).

***tert*-Butyl (2*R*,3*S*)-2-(2-cyclohexylethyl)-3-(perfluorobutyl)-2-(4,4,5,5-tetraethyl-1,3,2-dioxaborolan-2-yl)azetidine-1-carboxylate (<u>3b</u>)**

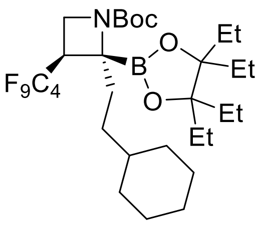

According to GP-A, using 2-(2-cyclohexylethyl)-4,4,5,5-tetraethyl-1,3,2-dioxaborolane **<u>Si-16</u>** (115 mg, 0.39 mmol, 1.3 equiv.) and nonafluoro-1-iodobutane (0.15 mL, 0.9 mmol, 3.0 equiv.). Purification *via* flash-column chromatography (SiO_2; Pentane – EtOAc: 99:1 to 97:3) yielded the title compound as a colorless oil (0.12 g, 0.18 mmol, 60 %). ^{19}F NMR analysis of the crude reaction mixture indicated a diastereomeric ratio of <u>8:1</u>. The title compound was obtained in a mixture of *N*-Boc Rotamers in a ratio of 1:1, as indicated by ^{1}H NMR.

^{1}H NMR (600 MHz, Acetone-D$_6$): δ (ppm) = 3.93 (dd, J = 17.6, 8.7 Hz, 1H), 3.85 (d, J = 8.7 Hz, 1H), 3.40 – 3.21 (m, 1H), 1.95 – 1.83 (m, 2H), 1.80 – 1.67 (m, 12H), 1.66 – 1.61 (m, 1H), 1.43 (d, J = 8.3 Hz, 9H), 1.31 – 1.13 (m, 6H), 0.97 – 0.87 (m, 14H).

^{13}C NMR (150 MHz, Acetone-D$_6$): δ (ppm) = 155.45, 154.79, 118.31, 116.40, 89.58, 89.48, 87.53, 78.90, 78.64, 46.44, 44.89, 38.09, 37.63, 37.47, 37.32, 37.15, 36.99, 35.66, 34.64, 33.39, 33.37, 33.31, 33.16, 32.91, 30.85, 30.60, 27.78, 27.72, 26.46, 26.44, 26.12, 25.71, 25.35, 25.16, 24.99, 8.46, 8.25, 7.93, 7.66.

^{19}F NMR (400 MHz, Acetone-D$_6$): δ (ppm) = -81.87, -81.88, -81.90, -81.92, -81.95, -81.96, -112.80, -113.17, -113.52, -113.90, -115.81, -116.40, -117.17, -123.23, -123.59, -124.02, -124.36, -124.69, -124.95, -125.48, -125.76, -126.72, -126.76, -126.79, -126.82, -126.86, -126.87.

HRMS (ESI) m/z: [Na]$^+$ calcd for $C_{30}H_{47}BF_9NO_4Na^+$: 690.3351; found: 690.3365.

IR (Diamond-ATR, neat) $\tilde{\nu}_{max}$: 2926 (m), 2854 (w), 1704 (m), 1478 (w), 1456 (w), 1388 (m), 1362 (m), 1316 (m), 1292 (w), 1232 (vs), 1202 (s), 1182 (s), 1160 (s), 1132 (vs), 1068 (w), 1028 (w), 958 (w), 922 (m), 876 (w), 854 (w), 810 (w), 794 (w), 772 (w), 744 (m), 726 (m).

***tert*-Butyl (2*R*,3*S*)-3-(perfluorobutyl)-2-phenethyl-2-(4,4,5,5-tetraethyl-1,3,2-dioxaborolan-2-yl)azetidine-1-carboxylate (<u>3c</u>)**

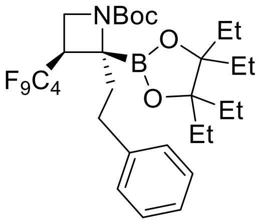

According to GP-A, using 4,4,5,5-tetraethyl-2-phenethyl-1,3,2-dioxaborolane **<u>Si-17</u>** (112 mg, 0.39 mmol, 1.3 equiv.) and nonafluoro-1-iodobutane (0.15 mL, 0.9 mmol, 3.0 equiv.). Purification *via* flash-column chromatography (SiO_2; Pentane – EtOAc: 98:2) yielded the title compound as a colorless oil (0.15 g, 0.23 mmol, 76 %). ^{19}F NMR analysis of the crude reaction mixture indicated a diastereomeric ratio of <u>10:1</u>. The title compound was obtained in a mixture of *N*-Boc Rotamers in a ratio of 1:1, as indicated by ^{1}H NMR.

^{1}H NMR (600 MHz, Acetone-D$_6$): δ (ppm) = 7.32 – 7.23 (m, 4H), 7.20 – 7.15 (m, 1H), 3.99 (dt, J = 22.1, 8.2 Hz, 1H), 3.92 (d, J = 8.2 Hz, 1H), 3.62 – 3.41 (m, 1H), 3.03 – 2.93 (m, 1H), 2.74 – 2.65 (m, 1H), 2.23 – 2.12 (m, 2H), 1.87 – 1.69 (m, 8H), 1.45 (d, J = 9.0 Hz, 9H), 0.97 – 0.89 (m, 12H).
^{13}C NMR (150 MHz, Acetone-D$_6$): δ (ppm) = 155.64, 154.81, 142.61, 142.29, 128.45, 128.34, 128.26, 128.25, 125.81, 125.64, 110.00, 89.78, 89.72, 79.16, 78.88, 41.13, 39.75, 37.92, 37.75, 37.60, 37.35, 37.20, 37.04, 30.41, 29.79, 27.81, 27.71, 25.75, 25.35, 25.22, 25.01, 8.47, 8.24, 7.99, 7.69.
^{19}F NMR (400 MHz, Acetone-D$_6$): δ (ppm) = -81.85, -81.86, -81.88, -81.89, -81.90, -81.91, -81.92, -81.94, -81.95, -112.90, -113.01, -113.64, -113.75, -115.87, -116.26, -116.60, -117.00, -123.18, -123.49, -123.98, -124.27, -124.64, -124.88, -125.43, -125.69, -125.98, -126.75, -126.77, -126.79, -126.81, -126.82, -126.84.

HRMS (ESI) m/z: [H]$^+$ calcd for $C_{30}H_{41}BF_9NO_4H^+$: 661.3065; found: 661.3075.
IR (Diamond-ATR, neat) $\tilde{\nu}_{max}$: 2978 (w), 2890 (w), 1704 (s), 1496 (vw), 1478 (w), 1456 (w), 1388 (m), 1362 (s), 1342 (m), 1292 (w), 1232 (vs), 1184 (s), 1162 (s), 1132 (vs), 1070 (m), 1028 (m), 990 (w), 956 (w), 922 (m), 898 (w), 870 (w), 854 (m), 794 (m), 770 (w), 744 (m), 726 (m).

tert-Butyl (2*R*,3*S*)-3-(perfluorohexyl)-2-phenethyl-2-(4,4,5,5-tetraethyl-1,3,2-dioxaborolan-2-yl)azetidine-1-carboxylate (3d)

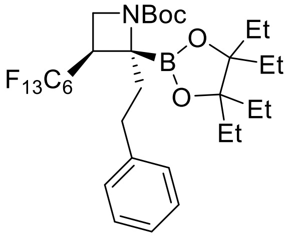

According to GP-A, using 4,4,5,5-tetraethyl-2-phenethyl-1,3,2-dioxaborolane **Si-17** (112 mg, 0.39 mmol, 1.3 equiv.) and perfluorohexyl iodide (0.19 mL, 0.9 mmol, 3.0 equiv.). Purification *via* flash-column chromatography (SiO_2; Pentane – EtOAc: 99:1 to 98:2) yielded the title compound as a colorless oil (0.17 g, 0.22 mmol, 74 %). ^{19}F NMR analysis of the crude reaction mixture indicated a diastereomeric ratio of >20:1. The title compound was obtained in a mixture of *N*-Boc Rotamers in a ratio of 1:1, as indicated by ^{1}H NMR.

^{1}H NMR (600 MHz, Acetone-D$_6$): δ (ppm) = 7.32 – 7.22 (m, 4H), 7.21 – 7.15 (m, 1H), 4.02 – 3.90 (m, 2H), 3.62 – 3.42 (m, 1H), 3.04 – 2.92 (m, 1H), 2.75 – 2.64 (m, 1H), 2.24 – 2.12 (m, 2H), 1.86 – 1.71 (m, 8H), 1.46 (d, J = 9.1 Hz, 9H), 0.98 – 0.89 (m, 12H).
^{13}C NMR (150 MHz, Acetone-D$_6$): δ (ppm) = 156.52, 155.70, 143.51, 143.18, 129.23, 129.14, 126.69, 126.53, 90.67, 90.61, 80.05, 79.77, 42.01, 40.63, 31.30, 28.70, 28.60, 26.64, 26.24, 25.91, 9.36, 9.12, 8.88, 8.58.
^{19}F NMR (400 MHz, Acetone-D$_6$): δ (ppm) = -81.64, -81.67, -81.69, -112.55, -112.71, -113.28, -113.43, -115.68, -116.04, -116.39, -116.77, -122.29, -122.56, -123.07, -123.39, -123.75, -124.34, -124.59, -126.71.

HRMS (ESI) m/z: $[H]^+$ calcd for $C_{32}H_{41}BF_{13}NO_4H^+$:762.3001; found: 762.3019.
IR (Diamond-ATR, neat) $\tilde{\nu}_{max}$: 2956 (w), 2894 (vw), 1704 (m), 1690 (m), 1496 (vw), 1476 (w), 1456 (w), 1416 (m), 1390 (m), 1362 (m), 1316 (w), 1296 (w), 1274 (w), 1230 (s), 1206 (s), 1168 (s), 1142 (vs), 1124 (s), 1068 (w), 1028 (w), 978 (w), 968 (w), 914 (m), 882 (w), 846 (vw), 792 (w), 770 (w), 746 (w), 716 (m).

***tert*-butyl (2*R*,3*S*)-2-methyl-3-(perfluorobutyl)-2-(4,4,5,5-tetraethyl-1,3,2-dioxaborolan-2-yl)azetidine-1-carboxylate (<u>3e</u>)**

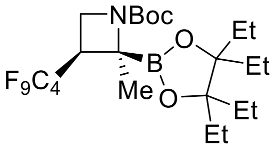

According to GP-A, using 4,4,5,5-tetraethyl-2-methyl-1,3,2-dioxaborolane **<u>Si-18</u>** (77 mg, 0.39 mmol, 1.3 equiv.) and nonafluoro-1-iodobutane (0.15 mL, 0.9 mmol, 3.0 equiv.). Purification *via* flash-column chromatography (SiO_2; Pentane – EtOAc: 99:1 to 98:2) yielded the title compound as a colorless oil (0.13 g, 0.23 mmol, 78 %). ^{19}F NMR analysis of the crude reaction mixture indicated a diastereomeric ratio of <u>1:1</u>. The title compound was obtained in a mixture of *N*-Boc Rotamers in a ratio of 1:1, as indicated by ^{1}H NMR.

^{1}H NMR (600 MHz, Acetone-D_6): δ (ppm) = 4.18 – 3.83 (m, 2H), 3.48 – 3.15 (m, 1H), 1.84 – 1.66 (m, 8H), 1.60 – 1.51 (m, 3H), 1.43 (d, J = 3.2 Hz, 9H), 0.99 – 0.86 (m, 12H).
^{13}C NMR (150 MHz, Acetone-D_6): δ (ppm) = 155.90, 155.71, 154.82, 89.68, 79.18, 78.81, 27.76, 27.72, 27.66, 25.86, 25.75, 25.70, 25.38, 25.13, 25.02, 24.95, 24.81, 8.41, 8.24, 8.20, 8.08, 7.88, 7.65.

HRMS (ESI) m/z: $[Na]^+$ calcd for $C_{23}H_{35}BF_9NO_4Na^+$: 594.2413; found: 594.2418.
IR (Diamond-ATR, neat) $\tilde{\nu}_{max}$: 2890 (w), 1704 (m), 1460 (w), 1384 (m), 1362 (m), 1316 (w), 1290 (w), 1236 (s), 1198 (s), 1162 (s), 1142 (vs), 1058 (m), 1026 (w), 972 (w), 956 (vw), 924 (m), 888 (w), 874 (w), 860 (w), 800 (w), 776 (w), 734 (w).

tert-Butyl (2_R_,3_S_)-3-(2-ethoxy-1,1-difluoro-2-oxoethyl)-2-phenethyl-2-(4,4,5,5-tetraethyl-1,3,2-dioxaborolan-2-yl)azetidine-1-carboxylate (3f)

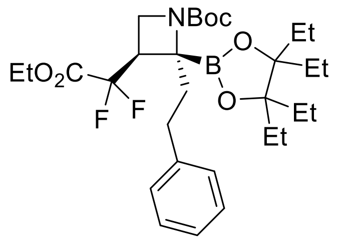

According to GP-A, using 4,4,5,5-tetraethyl-2-phenethyl-1,3,2-dioxaborolane **Si-17** (112 mg, 0.39 mmol, 1.3 equiv.) and ethyl iododifluoroacetate (145 mg, 0.6 mmol, 2.0 equiv.). Purification *via* flash-column chromatography (SiO_2; Pentane – EtOAc: 93:7) yielded the title compound as a colorless oil (82 mg, 0.14 mmol, 48 %). ^{19}F NMR analysis of the crude reaction mixture indicated a diastereomeric ratio of >20:1. The title compound was obtained in a mixture of *N*-Boc Rotamers in a ratio of 1:1, as indicated by ^{1}H NMR.

^{1}H NMR (600 MHz, Acetone-D$_6$): δ (ppm) = 7.32 – 7.14 (m, 5H), 4.41 – 4.28 (m, 2H), 3.93 – 3.82 (m, 2H), 3.49 – 3.39 (m, 1H), 2.97 (tdd, J = 13.4, 10.5, 6.0 Hz, 1H), 2.72 – 2.65 (m, 1H), 2.20 – 2.08 (m, 2H), 1.85 – 1.67 (m, 8H), 1.45 (d, J = 10.9 Hz, 9H), 1.37 – 1.33 (m, 3H), 0.95 – 0.88 (m, 12H).
^{13}C NMR (150 MHz, Acetone-D$_6$): δ (ppm) = 156.19, 155.73, 143.71, 143.36, 129.33, 129.20, 129.15, 126.64, 126.46, 90.57, 90.37, 79.46, 79.26, 63.87, 41.46, 40.16, 31.28, 30.72, 28.70, 28.64, 26.72, 26.28, 26.07, 25.65, 14.15, 9.57, 9.19, 8.89, 8.41.
^{19}F NMR (400 MHz, Acetone-D$_6$): δ (ppm) = -105.26, -105.30, -105.98, -106.03, -106.59, -106.64, -107.30, -107.35, -108.41, -108.45, -109.12, -109.16, -110.27, -110.32, -111.00, -111.05.

HRMS (ESI) m/z: $[Na]^+$ calcd for $C_{30}H_{46}BF_2NO_6Na^+$: 588.3284; found: 588.3295.
IR (Diamond-ATR, neat) $\tilde{\nu}_{max}$: 2976 (m), 2888 (w), 1772 (m), 1698 (s), 1604 (vw), 1496 (w), 1476 (w), 1456 (m), 1410 (m), 1388 (s), 1362 (s), 1342 (m), 1306 (s), 1258 (m), 1240 (m), 1220 (m), 1156 (s), 1100 (vs), 1072 (m), 1044 (m), 1018 (m), 982 (w), 950 (w), 920 (m), 864 (w), 826 (w), 768 (m), 752 (m).

***tert*-Butyl (2*S*,3*S*)-3-(2-ethoxy-1,1-difluoro-2-oxoethyl)-2-(4,4,5,5-tetraethyl-1,3,2-dioxaborolan-2-yl)-2-((trimethylsilyl)methyl)azetidine-1-carboxylate (<u>3g</u>)**

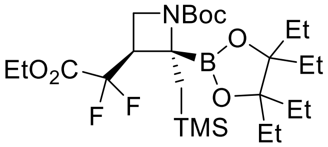

According to GP-A, using trimethyl((4,4,5,5-tetraethyl-1,3,2-dioxaborolan-2-yl)methyl)silane **<u>Si-19</u>** (0.11 g, 0.39 mmol, 1.3 equiv.) and ethyl iododifluoroacetate (145 mg, 0.6 mmol, 2.0 equiv.). Purification *via* flash-column chromatography (SiO_2; Pentane – EtOAc: 92:8) yielded the title compound as a colorless oil (103 mg, 0.19 mmol, 63 %). ^{19}F NMR analysis of the crude reaction mixture indicated a diastereomeric ratio of ><u>20:1</u>. The title compound was obtained in a mixture of N-Boc Rotamers in a ratio of 1:1, as indicated by ^{1}H NMR.

^{1}H NMR (400 MHz, Acetone-D$_6$): δ (ppm) = δ 4.43 – 4.27 (m, 2H), 3.95 – 3.74 (m, 2H), 3.35 – 3.10 (m, 1H), 1.86 – 1.63 (m, 8H), 1.43 (d, J = 8.6 Hz, 9H), 1.40 – 1.31 (m, 4H), 1.28 – 1.19 (m, 1H), 0.97 – 0.85 (m, 12H), 0.13 (d, J = 21.6 Hz, 9H).
^{13}C NMR (100 MHz, Acetone-D$_6$): δ (ppm) = 156.26, 155.96, 90.59, 90.39, 79.36, 79.18, 66.11, 63.86, 47.17, 45.20, 43.89, 43.65, 42.29, 42.06, 41.82, 28.73, 28.70, 28.00, 26.74, 26.21, 26.15, 25.64, 15.61, 14.18, 9.67, 9.14, 8.30, 0.91, 0.68, 0.44.
^{19}F NMR (**400 MHz, Acetone-D$_6$**): δ (ppm) = -104.58, -104.62, -105.31, -105.35, -106.75, -106.81, -107.46, -107.52, -108.68, -108.71, -109.39, -109.43, -110.71, -110.77, -111.45, -111.50.

HRMS (ESI) m/z: $[Na]^+$ calcd for $C_{26}H_{48}BF_2NO_6SiNa^+$: 570.3201; found: 570.3207.
IR (Diamond-ATR, neat) $\tilde{\nu}_{max}$: 2952 (m), 2888 (w), 1774 (m), 1762 (m), 1702 (s), 1478 (w), 1458 (m), 1390 (s), 1364 (s), 1334 (m), 1306 (s), 1244 (s), 1160 (s), 1092 (vs), 1066 (m), 1028 (m), 994 (w), 924 (m), 912 (m), 856 (vs), 836 (vs), 790 (w), 768 (m), 726 (w).

tert-Butyl (2_R_,3_S_)-2-phenethyl-2-(4,4,5,5-tetraethyl-1,3,2-dioxaborolan-2-yl)-3-(2,2,2-trifluoroethyl)azetidine-1-carboxylate (3h)

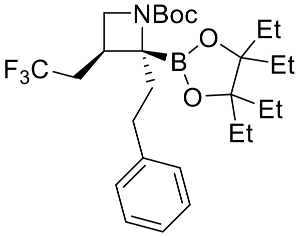

According to GP-A, using 4,4,5,5-tetraethyl-2-phenethyl-1,3,2-dioxaborolane **Si-17** (112 mg, 0.39 mmol, 1.3 equiv.) and 1,1,1-trifluoro-2-iodoethane (89 µL, 0.9 mmol, 3.0 equiv.). Purification *via* flash-column chromatography (SiO_2; Pentane – EtOAc: 98:2 to 95:5) yielded the title compound as a colorless oil (62 mg, 0.12 mmol, 39 %). ^{19}F NMR analysis of the crude reaction mixture indicated a diastereomeric ratio of 7:1. The title compound was obtained in a mixture of *N*-Boc Rotamers in a ratio of 1:1, as indicated by ^{1}H NMR.

^{1}H NMR (600 MHz, Acetone-D$_6$): δ (ppm) = 7.31 – 7.21 (m, 4H), 7.20 – 7.12 (m, 1H), 4.01 – 3.86 (m, 1H), 3.75 – 3.62 (m, 1H), 2.93 – 2.65 (m, 3H), 2.63 – 2.48 (m, 2H), 2.19 – 2.07 (m, 2H), 1.90 – 1.68 (m, 8H), 1.44 (s, 9H), 1.03 – 0.89 (m, 12H).
^{13}C NMR (150 MHz, Acetone-D$_6$): δ (ppm) = 143.99, 143.69, 129.15, 126.41, 90.52, 90.09, 79.36, 53.33, 51.86, 41.58, 40.06, 36.30, 36.02, 35.75, 35.47, 32.22, 31.47, 28.73, 28.66, 26.58, 26.37, 14.36, 13.93, 9.27, 9.02.
^{19}F NMR (400 MHz, Acetone-D$_6$): -65.83, -65.86, -65.89, -65.92, -65.98, -66.00, -66.03, -66.06.

HRMS (ESI) m/z: $[Na]^+$ calcd for $C_{28}H_{43}BF_3NO_4Na^+$: 548.3129; found: 548.3141.
IR (Diamond-ATR, neat) $\tilde{\nu}_{max}$: 2976 (m), 2886 (w), 1696 (s), 1604 (vw), 1496 (w), 1478 (w), 1456 (m), 1390 (s), 1362 (s), 1330 (m), 1272 (m), 1254 (s), 1232 (m), 1140 (vs), 1110 (m), 1050 (m), 1028 (w), 1004 (m), 980 (w), 954 (w), 920 (m), 854 (w), 836 (w), 782 (w), 752 (w).

***tert*-Butyl (2*S*,3*S*)-2-(4-chlorophenyl)-2-(4,4,5,5-tetraethyl-1,3,2-dioxaborolan-2-yl)-3-(2,2,2-trifluoroethyl)azetidine-1-carboxylate (<u>3i</u>)**

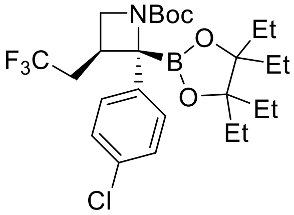

According to GP-A, using 2-(4-chlorophenyl)-4,4,5,5-tetraethyl-1,3,2-dioxaborolane **<u>Si-20</u>** (0.12 g, 0.39 mmol, 1.3 equiv.) and 1,1,1-trifluoro-2-iodoethane (89 µL, 0.9 mmol, 3.0 equiv.). Purification *via* flash-column chromatography (SiO_2; Pentane – EtOAc: 97/3) yielded the title compound as a colorless oil (65 mg, 0.12 mmol, 41 %).^{19}F NMR analysis of the crude reaction mixture indicated a diastereomeric ratio of <u>4:1</u>. The title compound was obtained in a mixture of *N*-Boc Rotamers in a ratio of 0,75:1, as indicated by ^{1}H NMR.

^{1}H NMR (800 MHz, Acetone-D$_6$): δ (ppm) = 7.51 – 7.26 (m, 4H), 4.40 – 4.20 (m, 1H), 3.81 – 3.63 (m, 1H), 3.10 – 2.99 (m, 1H), 1.87 – 1.74 (m, 10H), 1.50 (d, J = 7.4 Hz, 9H), 0.97 – 0.90 (m, 12H).
^{13}C NMR (200 MHz, Acetone-D$_6$): δ (ppm) = 129.31, 128.52, 91.15, 90.72, 90.30, 80.00, 78.88, 54.96, 53.60, 52.04, 28.61, 28.50, 27.87, 27.83, 26.56, 26.52, 9.45, 9.23, 8.98, 8.91.
^{19}F NMR (400 MHz, Acetone-D$_6$): δ (ppm) = -65.23, -65.25, -65.28, -65.85, -65.88, -65.91.

HRMS (ESI) m/z: [Na]$^+$ calcd for $C_{26}H_{38}BClF_3NO_4^+$: 554.2433; found: 554.2428.
IR (Diamond-ATR, neat) $\tilde{\nu}_{max}$: 2946 (w), 2888 (w), 1700 (vs), 1490 (w), 1458 (w), 1436 (w), 1388 (s), 1362 (s), 1340 (s), 1292 (m), 1274 (m), 1246 (vs), 1144 (vs), 1092 (s), 1030 (m), 1014 (m), 998 (m), 968 (m), 922 (m), 906 (m), 852 (w), 826 (w), 782 (m), 722 (vw), 704 (vw).

***tert*-Butyl (2*R*,3*S*)-2-(4-fluorophenethyl)-2-(4,4,5,5-tetraethyl-1,3,2-dioxaborolan-2-yl)-3-(2,2,2-trifluoroethyl)azetidine-1-carboxylate (3j)**

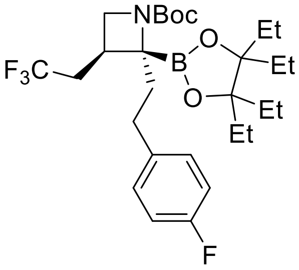

According to GP-A, using 4,4,5,5-tetraethyl-2-(4-fluorophenethyl)-1,3,2-dioxaborolane **Si-21** (0.12 g, 0.39 mmol, 1.3 equiv.) and 1,1,1-trifluoro-2-iodoethane (89 µL, 0.9 mmol, 3.0 equiv.). Purification *via* flash-column chromatography (SiO_2; Pentane – EtOAc: 96:4) yielded the title compound as a colorless oil (29 mg, 0.05 mmol, 18 %). ^{19}F NMR analysis of the crude reaction mixture indicated a diastereomeric ratio of 7:1. The title compound was obtained in a mixture of *N*-Boc Rotamers in a ratio of 1:1, as indicated by ^{1}H NMR.

^{1}H NMR (400 MHz, Acetone-D_6): δ (ppm) = 7.30 – 7.20 (m, 2H), 7.08 – 6.97 (m, 2H), 3.99 – 3.86 (m, 1H), 3.73 – 3.65 (m, 1H), 2.88 – 2.64 (m, 4H), 2.61 – 2.51 (m, 2H), 2.11 – 2.07 (m, 1H), 1.86 – 1.70 (m, 8H), 1.42 (d, J = 4.3 Hz, 9H), 0.99 – 0.89 (m, 12H).
^{13}C NMR (100 MHz, Acetone-D_6): δ (ppm) = 129.84, 129.76, 128.33, 125.58, 114.67, 112.00, 89.64, 89.64, 79.25, 78.47, 52.39, 50.93, 40.67, 39.14, 35.38, 35.10, 34.83, 34.55, 27.73, 27.49, 27.47, 25.65, 25.45, 19.88, 13.57, 8.34, 8.14, 8.08.
^{19}F NMR (400 MHz, Acetone-D_6): δ (ppm) = -65.77, -65.80, -65.83, -65.87, -65.90, -65.92, -65.98, -66.01, -66.04, -66.07, -119.41, -119.42, -119.43, -119.44, -119.47, -119.62, -119.63, -119.64, -119.66, -119.67, -119.68, -119.69.

HRMS (ESI) m/z: $[Na]^+$ calcd for $C_{28}H_{42}BF_4NO_4^+$: 566.3041; found: 566.3046.
IR (Diamond-ATR, neat) $\tilde{\nu}_{max}$: 2886 (w), 1740 (w), 1696 (s), 1602 (vw), 1510 (m), 1478 (w), 1456 (w), 1390 (s), 1362 (s), 1330 (m), 1274 (m), 1254 (s), 1224 (m), 1142 (vs), 1112 (m), 1100 (m), 1054 (m), 1026 (m), 1006 (m), 982 (w), 956 (w), 922 (m), 854 (w), 832 (m), 776 (m).

tert-Butyl (2_R_,3_S_)-2-(adamantan-1-ylmethyl)-3-(2-ethoxy-2-oxoethyl)-2-(4,4,5,5-tetraethyl-1,3,2-dioxaborolan-2-yl)azetidine-1-carboxylate (3k)

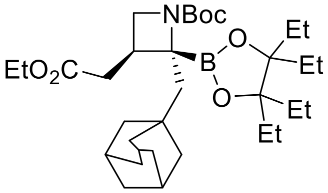

According to GP-A, using 2-(adamantan-1-yl)methyl)-4,4,5,5-tetraethyl-1,3,2-dioxaborolane **Si- 22** (0.13 g, 0.39 mmol, 1.3 equiv.) and ethyl iodoacetate (71 µL, 0.6 mmol, 2.0 equiv.). Purification *via* flash-column chromatography (SiO_2; Pentane – EtOAc: 91:9) yielded the title compound as a colorless oil (0.11 g, 0.2 mmol, 66 %). ^{1}H NMR and GC analysis of the crude reaction mixture indicated a diastereomeric ratio of >20:1. The title compound was obtained in a mixture of *N*-Boc Rotamers in a ratio of 0.75:1, as indicated by ^{1}H NMR.

^{1}H NMR (800 MHz, Acetone-D_6): δ (ppm) = 4.12 – 4.04 (m, 2H), 3.89 (dt, J = 57.9, 8.5 Hz, 1H), 3.53 (dt, J = 57.9, 8.5 Hz, 1H), 2.69 – 2.59 (m, 2H), 2.09 – 2.06 (m, 2H), 1.96 – 1.88 (m, 3H), 1.82 – 1.67 (m, 22H), 1.42 (d, J = 23.4 Hz, 9H), 1.24 – 1.20 (m, 2H), 0.97 – 0.90 (m, 12H).
^{13}C NMR (200 MHz, Acetone-D_6): δ (ppm) = 172.18, 156.82, 156.11, 90.19, 79.03, 78.64, 60.79, 53.82, 52.20, 51.81, 49.61, 44.82, 44.30, 37.77, 36.95, 36.82, 35.86, 34.83, 34.59, 34.54, 28.80, 28.75, 26.77, 26.42, 26.34, 14.55, 9.27, 9.21, 9.07, 8.88.

HRMS (ESI) m/z: $[Na]^+$ calcd for $C_{33}H_{56}BNO_6Na^+$: 596.4098; found: 596.4092.
IR (Diamond-ATR, neat) $\tilde{\nu}_{max}$: 2900 (s), 2844 (m), 1734 (s), 1692 (s), 1454 (m), 1416 (m), 1386 (s), 1354 (s), 1320 (s), 1308 (s), 1232 (m), 1214 (m), 1176 (s), 1152 (vs), 1110 (s), 1094 (s), 1050 (m), 1026 (s), 994 (w), 976 (w), 958 (w), 920 (s), 892 (w), 862 (m), 820 (w), 800 (w), 778 (m), 746 (w), 706 (w), 672 (w).

tert-Butyl (2_R_,3_S_)-3-(2-ethoxy-2-oxoethyl)-2-neopentyl-2-(4,4,5,5-tetraethyl-1,3,2-dioxaborolan-2-yl)azetidine-1-carboxylate (3l)

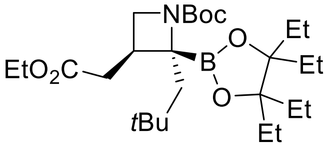

According to GP-A, using 4,4,5,5-tetraethyl-2-neopentyl-1,3,2-dioxaborolane **Si-23** (0.10 g, 0.39 mmol, 1.3 equiv.) and ethyl iodoacetate (71 µL, 0.6 mmol, 2.0 equiv.). Purification *via* flash-column chromatography (SiO_2; Pentane – EtOAc: 91:9) yielded the title compound as a colorless oil (0.12 g, 0.23 mmol, 78 %). ^{1}H NMR and GC analysis of the crude reaction mixture indicated a diastereomeric ratio of >20:1. The title compound was obtained in a mixture of *N*-Boc Rotamers in a ratio of 1:1, as indicated by ^{1}H NMR.

^{1}H NMR (600 MHz, Acetone-D_6): δ (ppm) = 4.08 (q, J = 7.2 Hz, 2H), 3.96 – 3.78 (m, 1H), 3.62 – 3.43 (m, 1H), 3.21 – 2.89 (m, 1H), 2.68 – 2.54 (m, 2H), 1.96 – 1.86 (m, 2H), 1.84 – 1.67 (m, 8H), 1.42 (d, J = 9.5 Hz, 9H), 1.22 (t, J = 7.1 Hz, 3H), 1.06 (d, J = 11.3 Hz, 9H), 0.99 – 0.88 (m, 12H).
^{13}C NMR (150 MHz, Acetone-D_6): δ (ppm) = 172.15, 157.10, 156.32, 90.34, 90.28, 79.12, 78.72, 60.82, 53.72, 52.16, 51.71, 49.69, 47.91, 37.01, 36.85, 35.12, 33.88, 32.35, 32.28, 32.02, 31.73, 28.79, 26.87, 26.48, 26.43, 26.29, 9.32, 9.19, 9.08, 8.80.

HRMS (ESI) m/z: $[Na]^+$ calcd for $C_{27}H_{50}BNO_6^+$: 518.3629; found: 518.3623.
IR (Diamond-ATR, neat) $\tilde{\nu}_{max}$: 2948 (m), 2884 (w), 1728 (m), 1694 (s), 1478 (w), 1458 (m), 1422 (m), 1384 (s), 1364 (s), 1350 (s), 1320 (s), 1304 (m), 1250 (m), 1222 (m), 1180 (s), 1156 (vs), 1112 (m), 1092 (s), 1062 (m), 1026 (m), 1000 (w), 966 (w), 950 (w), 916 (s), 876 (w), 856 (w), 814 (w), 782 (m), 760 (w), 712 (w), 674 (w).

2-((2*R*,3*S*)-1-(*tert*-Butoxycarbonyl)-2-neopentyl-2-(4,4,5,5-tetraethyl-1,3,2-dioxaborolan-2-yl)azetidin-3-yl)acetic acid (3l')

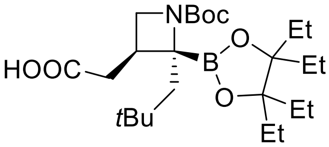

tert-Butyl (2*S*,3*S*)-3-(2-ethoxy-2-oxoethyl)-2-neopentyl-2-(4,4,5,5-tetraethyl-1,3,2-dioxaborolan-2-yl)azetidine-1-carboxylate (49 mg, 0.1 mmol, 1.0 equiv.) was dissolved in a mixture of H_2O (0.5 mL), THF (1 mL) and MeOH (1 mL) and treated with LiOH (0.06 g, 2.5 mmol, 25 equiv.). After stirring at rt. overnight, the solvents were removed *in vacuo*, and the residue was dissolved in Et_2O (10 mL) and filtered. Further concentration *in vacuo* and flash column chromatography (SiO_2; DCM – MeOH: 99:1) afforded the title compound as a viscous colorless oil (35 mg, 0.08 mmol, 76%). ^{1}H NMR and GC analysis of the crude reaction mixture indicated a diastereomeric ratio of >20:1.

^{1}H NMR (600 MHz, Acetone-D$_6$): δ (ppm) = 3.35 – 3.28 (m, 1H), 3.15 – 3.05 (m, 1H), 2.61 (dd, J = 17.0, 7.8 Hz, 1H), 2.49 – 2.40 (m, 1H), 2.27 (dd, J = 17.0, 8.5 Hz, 1H), 1.94 (d, J = 14.5 Hz, 1H), 1.86 – 1.71 (m, 8H), 1.64 (d, J = 14.5 Hz, 1H), 1.40 (s, 9H), 1.00 (s, 9H), 0.93 (td, J = 7.5, 3.4 Hz, 12H).

^{13}C NMR (150 MHz, Acetone-D$_6$): δ (ppm) = 176.27, 156.62, 91.32, 78.94, 51.68, 49.26, 42.03, 33.41, 31.89, 31.28, 28.55, 26.26, 26.17, 8.88, 8.83.

HRMS (ESI) m/z: $[H]^+$ calcd for $C_{25}H_{45}O_6BN^+$: 466.3340; found: 466.3348.

***tert*-Butyl (2*R*,3*S*)-3-(2-ethoxy-2-oxoethyl)-2-phenethyl-2-(4,4,5,5-tetraethyl-1,3,2-dioxaborolan-2-yl)azetidine-1-carboxylate (3m)**

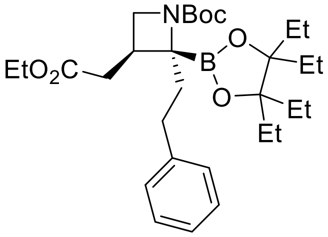

According to GP-A, using 4,4,5,5-tetraethyl-2-phenethyl-1,3,2-dioxaborolane **Si-17** (112 mg, 0.39 mmol, 1.3 equiv.) and Ethyl iodoacetate (71 µL, 0.6 mmol, 2.0 equiv.). Purification *via* flash-column chromatography (SiO_2; Pentane – EtOAc: 92:8) yielded the title compound as a colorless oil (0.99 g, 0.19 mmol, 62 %). ^{1}H NMR and GC analysis of the crude reaction mixture indicated a diastereomeric ratio of >20:1. The title compound was obtained in a mixture of *N*-Boc Rotamers in a ratio of 1:1, as indicated by ^{1}H NMR.

^{1}H NMR (600 MHz, Acetone-D$_6$): δ (ppm) = δ 7.32 – 7.23 (m, 4H), 7.20 – 7.14 (m, 1H), 4.13 – 4.05 (m, 2H), 3.94 (dt, J = 55.1, 8.4 Hz, 1H), 3.58 (dt, J = 55.1, 8.4 Hz, 1H), 2.94 – 2.85 (m, 1H), 2.79 – 2.63 (m, 3H), 2.17 – 2.07 (m, 2H), 1.89 – 1.71 (m, 8H), 1.45 (d, J = 12.4 Hz, 9H), 1.26 – 1.21 (m, 3H), 1.01 – 0.91 (m, 12H).
^{13}C NMR (150 MHz, Acetone-D$_6$): δ (ppm) = 172.21, 172.10, 157.45, 156.07, 144.35, 144.07, 129.23, 129.16, 129.12, 126.43, 126.28, 90.29, 90.20, 79.13, 78.94, 66.11, 60.82, 53.62, 52.02, 41.59, 40.11, 36.92, 36.85, 34.45, 33.60, 31.64, 31.09, 28.78, 28.69, 26.77, 26.53, 26.43, 26.40, 15.61, 14.54, 9.27, 9.20, 9.06, 8.95.

HRMS (ESI) m/z: $[H]^+$ calcd for $C_{30}H_{48}BNO_6H^+$:530.3655; found: 530.3661.
IR (Diamond-ATR, neat) $\tilde{\nu}_{max}$: 2976 (m), 2940 (m), 2884 (w), 1734 (s), 1694 (vs), 1604 (vw), 1496 (w), 1476 (w), 1456 (m), 1388 (s), 1362 (s), 1310 (m), 1288 (m), 1254 (m), 1178 (s), 1154 (vs), 1110 (s), 1064 (m), 1028 (m), 970 (w), 952 (w), 922 (m), 866 (w), 782 (w), 752 (m), 700 (s).

***tert*-Butyl (2*R*,3*S*)-3-(2-ethoxy-2-oxoethyl)-2-methyl-2-(4,4,5,5-tetraethyl-1,3,2-dioxaborolan-2-yl)azetidine-1-carboxylate (3n)**

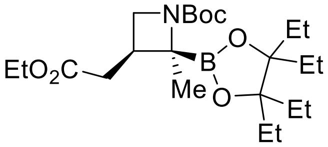

According to GP-A, using 4,4,5,5-tetraethyl-2-methyl-1,3,2-dioxaborolane **Si-18** (77 mg, 0.39 mmol, 1.3 equiv.) and ethyl iodoacetate (71 µL, 0.6 mmol, 2.0 equiv.). Purification *via* flash-column chromatography (SiO_2; Pentane – EtOAc: 9:1) yielded the title compound as a colorless oil (76 mg, 0.17 mmol, 58 %). ^{1}H NMR and GC analysis of the crude reaction mixture indicated a diastereomeric ratio of 2:1. The title compound was obtained in a mixture of *N*-Boc Rotamers in a ratio of 1:1, as indicated by ^{1}H NMR.

^{1}H NMR (600 MHz, Acetone-D$_6$): δ (ppm) = 4.12 – 4.07 (m, 2H), 3.57 – 3.44 (m, 1H), 2.84 (s, 3H), 2.70 – 2.58 (m, 2H), 1.84 – 1.67 (m, 10H), 1.45 – 1.40 (m, 9H), 1.25 – 1.20 (m, 3H), 1.00 – 0.87 (m, 12H).
^{13}C NMR (150 MHz, Acetone-D$_6$): δ (ppm) = 172.51, 172.43, 172.19, 172.09, 157.38, 157.23, 156.31, 155.99, 90.17, 90.09, 89.75, 78.85, 60.78, 60.76, 60.67, 53.92, 53.18, 52.33, 51.56, 37.74, 37.41, 36.66, 36.50, 34.64, 34.54, 31.61, 31.50, 28.71, 28.69, 27.87, 26.85, 26.71, 26.65, 26.51, 26.44, 26.37, 24.97, 24.69, 17.28, 17.07, 14.51, 9.45, 9.20, 9.13, 9.04, 9.00, 8.96, 8.94.

HRMS (ESI) m/z: $[Na]^+$ calcd for $C_{23}H_{42}BNO_6Na^+$: 462.2997; found: 462.2997.
IR (Diamond-ATR, neat) $\tilde{\nu}_{max}$: 2942 (m), 2884 (w), 1736 (s), 1694 (vs), 1458 (m), 1388 (s), 1362 (s), 1310 (s), 1250 (m), 1178 (s), 1154 (vs), 1110 (s), 1054 (m), 1026 (s), 970 (w), 924 (s), 886 (w), 862 (w), 776 (m).

***tert*-Butyl (2*S*,3*S*)-2-(3-(adamantan-1-yl)-4-methoxyphenyl)-3-(2-ethoxy-2-oxoethyl)-2-(4,4,5,5-tetraethyl-1,3,2-dioxaborolan-2-yl)azetidine-1-carboxylate (3o)**

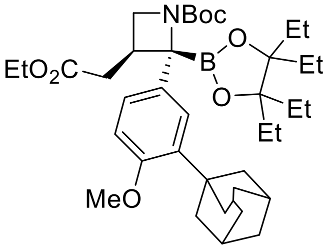

According to GP-A, using 2-(3-(adamantan-1-yl)-4-methoxyphenyl)-4,4,5,5-tetraethyl-1,3,2-dioxaborolane **Si-24** (0.17 g, 0.39 mmol, 1.3 equiv.) and ethyl iodoacetate (71 µL, 0.6 mmol, 2.0 equiv.). Purification *via* flash-column chromatography (SiO_2; Pentane – EtOAc: 92/8) yielded the title compound as a colorless oil (0.13 g, 0.19 mmol, 63 %). ^{1}H NMR and GC analysis of the crude reaction mixture indicated a diastereomeric ratio of 2:1. The title compound was obtained in a mixture of *N*-Boc Rotamers in a ratio of 1:1, as indicated by ^{1}H NMR.

^{1}H NMR (600 MHz, Acetone-D$_6$): δ (ppm) = 7.32 – 7.07 (m, 2H), 6.94 – 6.82 (m, 1H), 4.26 – 4.17 (m, 1H), 4.15 – 4.08 (m, 1H), 4.02 – 3.94 (m, 2H), 3.83 (d, J = 4.6 Hz, 3H), 3.13 – 3.02 (m, 1H), 2.13 (s, 6H), 1.84 – 1.73 (m, 15H), 1.47 (d, J = 4.6 Hz, 5H), 1.37 – 1.31 (m, 5H), 1.25 – 1.18 (m, 1H), 1.13 (t, J = 7.1 Hz, 2H), 1.00 – 0.87 (m, 15H).
^{13}C NMR (150 MHz, Acetone-D$_6$): δ (ppm) = 172.21, 158.04, 137.50, 90.55, 90.12, 79.18, 60.91, 60.67, 55.32, 41.59, 37.92, 37.72, 36.44, 28.71, 28.69, 26.57, 26.30, 14.53, 14.44, 9.31, 9.18.

HRMS (ESI) m/z: $[Na]^+$ calcd for $C_{39}H_{60}BNO_7Na^+$: 688.4361; found: 688.4351.
IR (Diamond-ATR, neat) $\tilde{\nu}_{max}$: 2904 (s), 2850 (m), 1734 (s), 1698 (s), 1606 (vw), 1496 (m), 1456 (m), 1386 (s), 1364 (s), 1312 (m), 1288 (m), 1258 (m), 1234 (vs), 1154 (vs), 1028 (s), 994 (m), 974 (w), 912 (s), 808 (m), 776 (m), 700 (vw).

tert-Butyl (2_R_,3_S_)-2-isobutyl-3-(perfluorobutyl)-2-(4,4,5,5-tetraethyl-1,3,2-dioxaborolan-2-yl)azetidine-1-carboxylate

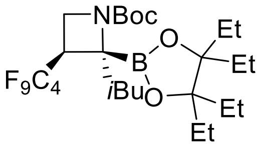

According to GP-A on a 2 mmol scale, using 4,4,5,5-tetraethyl-2-isobutyl-1,3,2-dioxaborolane **Si-26** (0.58 g, 2.6 mmol, 1.3 equiv.) and nonafluoro-1-iodobutane (1.0 mL, 6.0 mmol, 3.0 equiv.). Purification *via* flash-column chromatography (SiO_2; Pentane – EtOAc: 99/1 -> 97:3) yielded the title compound as a colorless oil (1.0 g, 1.7 mmol, 84 %). GC analysis of the crude reaction mixture indicated a diastereomeric ratio of 8:1. The title compound was directly employed in a deprotection – reprotection sequence towards compound **17b**. No additional analytics were determined.

tert-Butyl (2*R*,3S)-3-(2-ethoxy-2-oxoethyl)-2-isobutyl-2-(4,4,5,5-tetraethyl-1,3,2-dioxaborolan-2-yl)azetidine-1-carboxylate (3p)

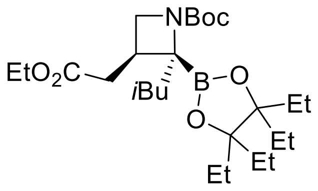

According to GP-A on a 2 mmol scale, using 4,4,5,5-tetraethyl-2-isobutyl-1,3,2-dioxaborolane **Si-26** (0.58 g, 2.6 mmol, 1.3 equiv.) and ethyl iodoacetate (0.47 mL, 4 mmol, 2.0 equiv.). Purification *via* flash-column chromatography (SiO_2; Pentane – EtOAc: 9/1) yielded the title compound as a colorless oil (0.53 g, 1.1 mmol, 55 %). GC analysis of the crude reaction mixture indicated a diastereomeric ratio of 9:1. The title compound was directly employed in a deprotection – reprotection sequence towards compound **18**. No additional analytics were determined.

Overview: trisubstituted Cyclobutanes

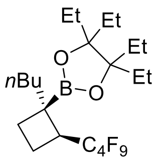

6a
dr = >20:1

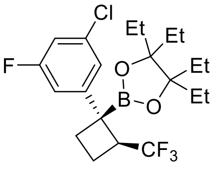

6b
dr = >20:1

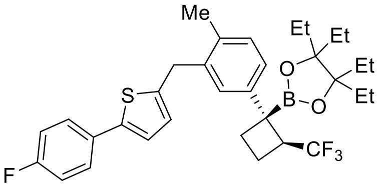

6c
dr = >20:1

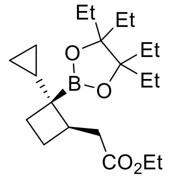

6d
dr = 6:1

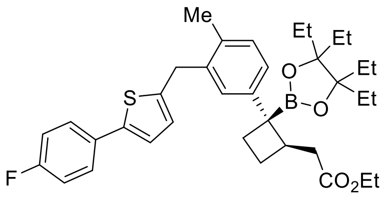

6e
dr = >20:1

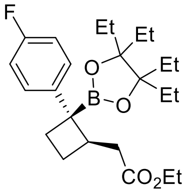

6f
dr = >20:1

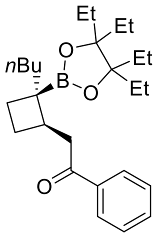

6g
dr = 18:1

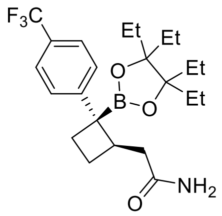

6h
dr = 7:1

2-((1*S*,2*S*)-1-Butyl-2-(perfluorobutyl)cyclobutyl)-4,4,5,5-tetraethyl-1,3,2-dioxaborolane (6a)

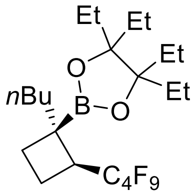

According to GP-B, using *n*BuLi (0.22 mmol, 1.1 equiv.) and nonafluoro-1-iodobutane (0.1 mL, 0.6 mmol, 3.0 equiv.). Purification *via* flash-column chromatography (SiO_2; Pentane – EtOAc: 99,5:0,5 to 98:2) yielded the title compound as a colorless oil (84 mg, 0.16 mmol, 82 %). ^{1}H NMR analysis of the crude reaction mixture indicated a diastereomeric ratio of >20:1.

^{1}H NMR (800 MHz, $CDCl_3$): δ (ppm) = 2.67 (dq, J = 26.6, 8.8, 8.0 Hz, 1H), 2.18 – 2.11 (m, 2H), 1.89 (q, J = 9.5 Hz, 1H), 1.83 (td, J = 12.3, 4.4 Hz, 1H), 1.76 – 1.62 (m, 8H), 1.55 – 1.52 (m, 1H), 1.34 – 1.25 (m, 3H), 1.20 – 1.15 (m, 1H), 1.11 – 1.06 (m, 1H), 0.91 – 0.86 (m, 15H).
^{13}C NMR (200 MHz, $CDCl_3$): δ (ppm) = 88.81, 45.62, 45.50, 45.38, 40.99, 30.46, 28.11, 27.79, 25.71, 25.65, 23.39, 19.28, 19.24, 14.22, 8.75, 8.71.
^{19}F NMR (400 MHz, $CDCl_3$): δ (ppm) = -81.04, -81.05, -81.06, -81.07, -81.08, -81.10, -81.11, -114.42, -115.13, -116.57, -116.60, -117.30, -117.34, -122.77, -122.80, -122.82, -123.56, -123.58, -123.61, -123.75, -123.77, -123.79, -124.54, -124.56, -124.58, -126.19, -126.23, -126.26, -126.29.

HRMS (EI-orbitrap): *m/z*: [M] calc. for [$C_{20}H_{29}BO_2F_9$]: 483.2117; found: 483.2110.
IR (Diamond-ATR, neat) $\tilde{\nu}_{max}$: 2960 (w), 2936 (w), 2886 (w), 1460 (w), 1410 (w), 1388 (m), 1352 (m), 1304 (w), 1288 (w), 1224 (vs), 1158 (m), 1132 (s), 1114 (s), 1084 (w), 1068 (w), 1024 (m), 988 (w), 968 (w), 924 (s), 868 (w), 850 (w), 830 (w), 810 (w), 774 (w), 732 (m), 694 (vw).

2-((1S,2S)-1-(3-Chloro-5-fluorophenyl)-2-(trifluoromethyl)cyclobutyl)-4,4,5,5-tetraethyl-1,3,2-dioxaborolane (6b)

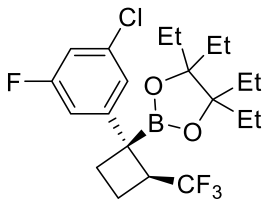

According to GP-B, using 1-bromo-3-chloro-5-fluorobenzene (27 µL, 0.22 mmol, 1.1 equiv.) and *n*BuLi (0.22 mmol, 1.1 equiv.) for Halogen-Lithium exchange at -78°C for 30min. Radical precursor Ritter-trifluoromethyl iodide (0.28 g, 0.8 mmol, 4.0 equiv.) was finally added to the reaction mixture in methyl-THF (0.5 mL). Purification *via* flash-column chromatography (SiO_2; Pentane – EtOAc: 99.05:0.5 to 99:1) yielded the title compound as a colorless oil (63 mg, 0.14 mmol, 72 %). ^{1}H NMR analysis of the crude reaction mixture indicated a diastereomeric ratio of >20:1.

^{1}H NMR (800 MHz, DCM-D$_2$): δ (ppm) = 6.92 (t, J = 1.7 Hz, 1H), 6.89 (dt, J = 8.5, 2.1 Hz, 1H), 6.79 (ddd, J = 9.9, 2.1, 1.7 Hz, 1H), 3.16 (h, J = 9.4 Hz, 1H), 2.65 – 2.62 (m, 1H), 2.27 (p, J = 9.8 Hz, 1H), 2.14 – 2.05 (m, 2H), 1.68 – 1.55 (m, 8H), 0.85 (t, J = 7.5 Hz, 6H), 0.76 (t, J = 7.5 Hz, 6H).
^{13}C NMR (200 MHz, DCM-D$_2$): δ (ppm) = 163.79, 162.55, 152.45, 135.05, 134.99, 129.12, 127.75, 126.37, 124.99, 122.31, 113.49, 113.37, 111.89, 111.78, 90.15, 47.34, 47.19, 47.04, 46.89, 28.52, 26.94, 26.27, 25.94, 19.10, 9.01, 8.59.
^{19}F NMR (400 MHz, DCM-D$_2$): δ (ppm) = -69.34, -69.36, -112.19, -112.21, -112.23.

HRMS (EI-orbitrap): *m/z*: [M] calc. for [$C_{21}H_{28}BO_2ClF_4$]: 434.1807; found: 434.1797.
IR (Diamond-ATR, neat) $\tilde{\nu}_{max}$: 2948 (w), 2886 (w), 1606 (w), 1584 (w), 1458 (w), 1432 (w), 1412 (w), 1384 (m), 1356 (m), 1340 (m), 1312 (w), 1292 (w), 1272 (m), 1248 (w), 1224 (vw), 1200 (vw), 1160 (m), 1134 (m), 1110 (m), 1088 (m), 1046 (w), 1026 (w), 968 (w), 908 (s), 870 (m), 848 (w), 804 (w), 796 (w), 732 (vs).

Ethyl 2-((1*R*,2*R*)-2-cyclopropyl-2-(4,4,5,5-tetraethyl-1,3,2-dioxaborolan-2-yl)cyclobutyl)acetate (6c)

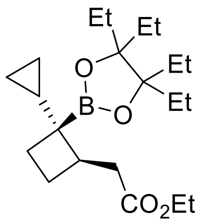

According to GP-B, using bromocyclopropane (18 µL, 0.22 mmol, 1.1 equiv.) and *t*BuLi (0.22 mmol, 1.1 equiv.) for Halogen-Lithium exchange at -78°C for 1h. Radical precursor ethyl iodoacetate (47 µL, 0.4 mmol, 2.0 equiv.) was finally added to the reaction mixture in methyl-THF (0.5 mL). Purification *via* flash-column chromatography (SiO_2; Pentane – EtOAc: 97/3) yielded the title compound as a colorless oil (69 mg, 0.17 mmol, 83 %). ^1H NMR analysis of the crude reaction mixture indicated a diastereomeric ratio of 6:1.

^1H NMR (600 MHz, $CDCl_3$): δ (ppm) = 4.17 – 4.06 (m, 2H), 2.60 – 2.51 (m, 1H), 2.46 – 2.39 (m, 1H), 1.98 – 1.89 (m, 1H), 1.81 – 1.60 (m, 10H), 1.37 (td, J = 10.0, 8.8 Hz, 1H), 1.26 (t, J = 7.1 Hz, 3H), 0.99 – 0.87 (m, 14H).
^{13}C NMR (150 MHz, $CDCl_3$): δ (ppm) = 173.48, 88.28, 88.09, 60.12, 39.42, 39.40, 26.46, 26.40, 26.23, 26.17, 24.88, 23.33, 16.77, 14.42, 9.04, 8.98, 8.92, 8.83, 1.31, 1.12, 0.78.

HRMS (ESI) m/z: $[Na]^+$ calcd for $C_{21}H_{37}BO_4Na^+$: 387.2683; found: 387.2679.
IR (Diamond-ATR, neat) $\tilde{v}_{max}$: 2974 (m), 2942 (m), 2884 (w), 1734 (vs), 1460 (m), 1412 (m), 1386 (s), 1366 (m), 1344 (s), 1294 (s), 1240 (m), 1162 (s), 1102 (s), 1032 (s), 970 (w), 956 (w), 922 (vs), 848 (w), 820 (w), 772 (w), 706 (vw), 686 (w).

Ethyl 2-((1*R*,2*S*)-2-(4-fluorophenyl)-2-(4,4,5,5-tetraethyl-1,3,2-dioxaborolan-2-yl)cyclobutyl)acetate (6d)

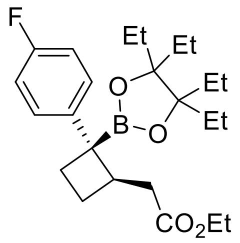

According to GP-B, using 4-bromofluorobenzene (24 µL, 0.22 mmol, 1.1 equiv.) and *n*BuLi (0.22 mmol, 1.1 equiv.) for Halogen-Lithium exchange at -78°C for 30 min. Radical precursor ethyl iodoacetate (47 µL, 0.4 mmol, 2.0 equiv.) was finally added dropwise to the reaction mixture in methyl-THF (0.5 mL). Purification *via* flash-column chromatography (SiO_2; Pentane – EtOAc: 96:4) yielded the title compound as a colorless oil (66 mg, 0.16 mmol, 81 %). ^1H NMR analysis of the crude reaction mixture indicated a diastereomeric ratio of >20:1.

^1H NMR (600 MHz, $CDCl_3$): δ (ppm) = 7.07 – 7.03 (m, 2H), 6.94 – 6.89 (m, 2H), 4.11 (q, J = 7.1 Hz, 2H), 2.88 – 2.82 (m, 2H), 2.68 – 2.60 (m, 1H), 2.50 – 2.45 (m, 1H), 2.15 – 2.02 (m, 2H), 1.85 – 1.78 (m, 1H), 1.70 – 1.53 (m, 10H), 1.24 (t, J = 7.2 Hz, 3H), 0.97 – 0.90 (m, 2H), 0.89 – 0.78 (m, 12H).

^{13}C NMR (150 MHz, $CDCl_3$): δ (ppm) = 173.12, 161.41, 159.80, 144.85, 126.97, 114.72, 114.58, 88.77, 60.33, 42.22, 40.25, 29.27, 26.32, 26.02, 14.41, 8.94, 8.66.

^{19}F NMR (400 MHz, $CDCl_3$): δ (ppm) = -118.98, -119.00, -119.00, -119.02, -119.03, -119.04, -119.06, -119.31, -119.33, -119.33, -119.34, -119.35, -119.36, -119.36, -119.37, -119.39.

HRMS (ESI) m/z: $[K]^+$ calcd for $C_{24}H_{36}BFO_4K^+$: 457.2322; found: 457.2330.

IR (Diamond-ATR, neat) $\tilde{\nu}_{max}$: 2942 (m), 2884 (w), 1734 (vs), 1602 (vw), 1508 (s), 1460 (m), 1366 (s), 1346 (s), 1296 (s), 1224 (s), 1160 (s), 1102 (vs), 1030 (s), 968 (w), 952 (w), 922 (vs), 858 (m), 832 (m), 810 (m), 772 (w), 736 (w), 680 (vw).

2-((1*R*,2*S*)-2-Butyl-2-(4,4,5,5-tetraethyl-1,3,2-dioxaborolan-2-yl)cyclobutyl)-1-phenylethan-1-one (6e)

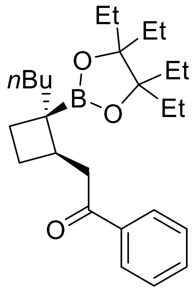

According to GP-B, using *n*BuLi (0.22 mmol, 1.1 equiv.) and 2-iodo-1-phenylethan-1-one (98 mg, 0.4 mmol, 2.0 equiv.). The radical precursor was added dropwise dissolved in 2-methylTHF (0.5 mL). Purification *via* flash-column chromatography (SiO_2; Pentane – EtOAc: 96:4) yielded the title compound as a colorless oil (58 mg, 0.14 mmol, 70 %). ^{1}H NMR analysis of the crude reaction mixture indicated a diastereomeric ratio of 18:1.

^{1}H NMR (600 MHz, $CDCl_3$): δ (ppm) = 7.94 – 7.92 (m, 2H), 7.55 – 7.52 (m, 1H), 7.47 – 7.42 (m, 2H), 3.23 – 3.10 (m, 2H), 2.59 – 2.46 (m, 1H), 2.08 – 1.99 (m, 2H), 1.74 – 1.60 (m, 10H), 1.53 – 1.45 (m, 1H), 1.30 – 1.21 (m, 3H), 1.21 – 1.12 (m, 2H), 0.95 – 0.84 (m, 15H).
^{13}C NMR (150 MHz, $CDCl_3$): δ (ppm) = 200.35, 137.57, 132.81, 128.58, 128.18, 88.25, 43.88, 40.92, 40.66, 29.03, 28.61, 26.32, 26.13, 25.71, 23.60, 14.31, 8.99, 8.87.

HRMS (ESI) m/z: $[K]^+$ calcd for $C_{26}H_{41}BO_3K^+$: 451.2780; found: 451.2788.
IR (Diamond-ATR, neat) $\tilde{\nu}_{max}$: 3366 (vw), 3086 (vw), 2966 (s), 2954 (s), 2930 (s), 2884 (m), 2860 (m), 1686 (s), 1598 (w), 1582 (w), 1512 (vw), 1454 (s), 1408 (m), 1388 (s), 1352 (s), 1284 (s), 1238 (m), 1216 (m), 1182 (m), 1144 (m), 1112 (s), 1076 (w), 1060 (w), 1044 (w), 1026 (w), 1000 (m), 988 (m), 956 (w), 924 (vs), 888 (w), 862 (w), 796 (vw), 750 (s).

2-((1*R*,2*S*)-2-(4,4,5,5-Tetraethyl-1,3,2-dioxaborolan-2-yl)-2-(4-(trifluoromethyl)-phenyl)cyclobutyl)acetamide (6f)

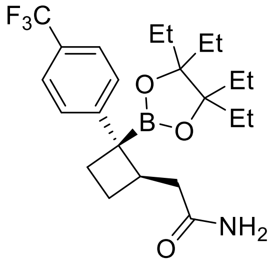

According to GP-B, using 4-bromobenzotrifluoride (31 µL, 0.22 mmol, 1.1 equiv.) and *n*BuLi (0.22 mmol, 1.1 equiv.) for Halogen-Lithium exchange at -78°C for 30 min. Radical precursor iodoacetamide (74 mg, 0.4 mmol, 2.0 equiv.) was finally added to the reaction mixture in one portion under nitrogen flow. Purification *via* flash-column chromatography (SiO_2; Pentane – EtOAc: 55:45) yielded the title compound as a colorless oil (52 mg, 0.12 mmol, 59 %). ^{1}H NMR analysis of the crude reaction mixture indicated a diastereomeric ratio of 7:1.

^{1}H NMR (600 MHz, Acetone-D$_6$): δ (ppm) = 7.56 (d, J = 8.0 Hz, 2H), 7.34 (d, J = 8.0 Hz, 2H), 6.76 (s, 1H), 6.12 (s, 1H), 3.02 – 2.92 (m, 1H), 2.84 – 2.75 (m, 1H), 2.60 – 2.50 (m, 2H), 2.16 – 2.07 (m, 2H), 1.97 – 1.86 (m, 1H), 1.73 – 1.58 (m, 8H), 0.91 – 0.79 (m, 12H).
^{13}C NMR (150 MHz, Acetone-D$_6$): δ (ppm) = 173.95, 155.57, 128.53, 127.08, 127.03, 125.53, 125.49, 125.41, 89.70, 43.74, 42.25, 42.22, 26.84, 26.78, 26.62, 8.87.
^{19}F NMR (100 MHz, Acetone-D$_6$): δ (ppm) = -62.52, -62.53.

HRMS (ESI) m/z: [Na]$^+$ calcd for $C_{23}H_{33}BF_3NO_3Na^+$: 462.2403; found: 462.2399.
IR (Diamond-ATR, neat) $\tilde{\nu}_{max}$: 3192 (vw), 2976 (w), 2942 (w), 2886 (w), 1740 (w), 1666 (m), 1616 (m), 1460 (w), 1444 (w), 1386 (m), 1366 (m), 1350 (m), 1324 (vs), 1288 (m), 1244 (m), 1190 (w), 1162 (m), 1112 (s), 1068 (s), 1046 (m), 1016 (m), 968 (vw), 954 (vw), 922 (m), 858 (w), 836 (w), 784 (vw), 768 (vw), 756 (vw).

Ethyl 2-((1*R*,2*S*)-2-(3-((5-(4-fluorophenyl)thiophen-2-yl)methyl)-4-methylphenyl)-2-(4,4,5,5-tetraethyl-1,3,2-dioxaborolan-2-yl)cyclobutyl)acetate (6g)

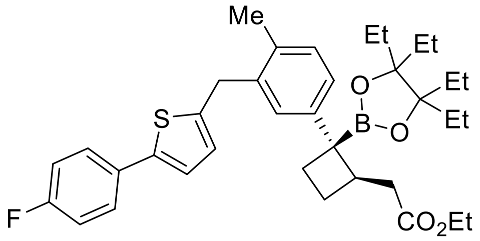

According to GP-B, using 2-(4-fluorophenyl)-5-(5-iodo-2-methylbenzyl)thiophene (99 mg, 0.22 mmol, 1.1 equiv.) and *n*BuLi (0.22 mmol, 1.1 equiv.) for Halogen-Lithium exchange at -78°C for 30min. Radical precursor ethyl iodoacetate (47 µL, 0.4 mmol, 2.0 equiv.) was finally added to the reaction mixture in methyl-THF (0.5 mL). Purification *via* flash-column chromatography (SiO_2; Pentane – EtOAc: 96:4) yielded the title compound as a colorless oil (74 mg, 0.12 mmol, 61 %). ^{1}H NMR analysis of the crude reaction mixture indicated a diastereomeric ratio of >20:1.

^{1}H NMR (800 MHz, DCM-D_2): δ (ppm) = 7.51 – 7.48 (m, 2H), 7.07 – 7.02 (m, 4H), 6.95 (d, J = 2.0 Hz, 1H), 6.92 (dd, J = 7.7, 2.1 Hz, 1H), 6.67 (dt, J = 3.7, 1.2 Hz, 1H), 4.10 – 4.05 (m, 4H), 2.87 – 2.79 (m, 2H), 2.64 (dd, J = 15.2, 10.7 Hz, 1H), 2.46 – 2.41 (m, 1H), 2.26 (s, 3H), 2.11 – 2.04 (m, 2H), 1.86 – 1.80 (m, 1H), 1.67 – 1.57 (m, 8H), 1.21 (t, J = 7.1 Hz, 3H), 0.86 – 0.81 (m, 12H).

^{13}C NMR (200 MHz, DCM-D_2): δ (ppm) = 173.20, 163.18, 161.96, 147.79, 144.82, 141.67, 138.24, 133.00, 131.62, 130.50, 127.58, 126.37, 124.42, 123.21, 116.18, 116.08, 89.20, 60.56, 42.82, 40.49, 34.70, 29.67, 26.65, 26.52, 19.24, 14.63, 9.13, 9.01.

^{19}F NMR (400 MHz, DCM-D_2): δ (ppm) = -116.11, -116.13, -116.14, -116.15, -116.16, -116.17, -116.17, -116.18, -116.19, -116.20.

HRMS (ESI) m/z: $[Na]^+$ calcd for $C_{36}H_{46}BFO_4SNa^+$:627.3092; found: 627.3084.

IR (Diamond-ATR, neat) $\tilde{\nu}_{max}$: 2940 (m), 2884 (w), 1732 (vs), 1606 (w), 1548 (vw), 1508 (s), 1460 (m), 1444 (m), 1410 (w), 1366 (s), 1344 (s), 1296 (s), 1258 (s), 1230 (vs), 1172 (s), 1160 (s), 1100 (s), 1030 (m), 970 (w), 954 (w), 922 (s), 894 (w), 858 (vw), 834 (s), 800 (s), 770 (w), 728 (w).

4,4,5,5-Tetraethyl-2-((1*S*,2*S*)-1-(3-((5-(4-fluorophenyl)thiophen-2-yl)methyl)-4-methylphenyl)-2-(trifluoromethyl)cyclobutyl)-1,3,2-dioxaborolane (6h)

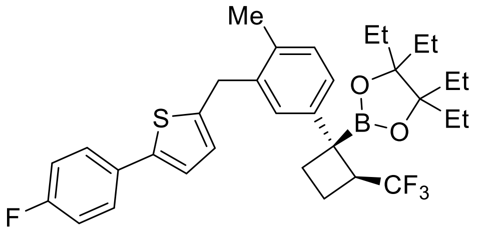

According to GP-B, using 2-(4-fluorophenyl)-5-(5-iodo-2-methylbenzyl)thiophene (99 mg, 0.22 mmol, 1.1 equiv.) and *n*BuLi (0.22 mmol, 1.1 equiv.) for Halogen-Lithium exchange at -78°C for 30min. Radical precursor Ritter-trifluoromethyl iodide (0.28 g, 0.8 mmol, 4.0 equiv.) was finally added to the reaction mixture in methyl-THF (0.5 mL). Purification *via* column chromatography (SiO_2; Pentane – EtOAc: 99.05:0.5 to 99:1; long column) yielded the title compound as a colorless oil (50 mg, 0.86 mmol, 43 %). ^{1}H NMR analysis of the crude reaction mixture indicated a diastereomeric ratio of >20:1.

^{1}H NMR (600 MHz, $CDCl_3$): δ (ppm) = 7.48 – 7.44 (m, 2H), 7.08 (d, J = 7.7 Hz, 1H), 7.04 – 6.97 (m, 5H), 6.63 (dd, J = 3.6, 1.1 Hz, 1H), 4.08 (s, 2H), 3.14 (h, J = 9.6 Hz, 1H), 2.65 (td, J = 9.6, 2.4 Hz, 1H), 2.27 (s, 3H), 2.22 (q, J = 9.9 Hz, 1H), 2.13 (q, J = 9.6 Hz, 1H), 2.05 (dtd, J = 11.0, 8.8, 2.4 Hz, 1H), 1.65 – 1.46 (m, 8H), 0.81 (t, J = 7.5 Hz, 6H), 0.72 (t, J = 7.5 Hz, 6H).
^{13}C NMR (150 MHz, $CDCl_3$): δ (ppm) = 162.96, 161.33, 145.70, 144.07, 144.06, 141.40, 137.82, 133.08, 131.13, 131.11, 130.37, 127.86, 127.21, 127.15, 126.02, 125.82, 124.15, 122.69, 122.68, 115.87, 115.72, 110.15, 89.07, 47.31, 47.12, 46.92, 46.72, 34.40, 32.08, 29.86, 29.52, 29.48, 28.11, 25.80, 25.66, 22.85, 19.13, 18.86, 18.83, 14.29, 8.76, 8.60, 1.17.
^{19}F NMR (400 MHz, $CDCl_3$): δ (ppm) = -69.17, -69.19, -115.36, -115.38, -115.39, -115.39, -115.40, -115.41, -115.41, -115.42, -115.44.

HRMS (ESI) m/z: $[Na]^+$ calcd for $C_{33}H_{39}BF_4O_2SNa^+$: 609.2598; found: 609.2596.

Five Membered Rings: Overview

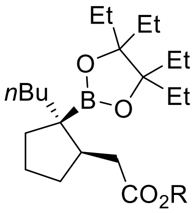

9a (R = Et)
dr = 19:1
9a' (R = *t*Bu)
dr = 13:1

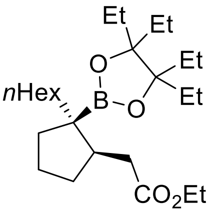

9b (R = Et)
dr = >20:1
9b' (R = H)
dr = >20:1
9b'' (R = *t*Bu)
dr = >20:1

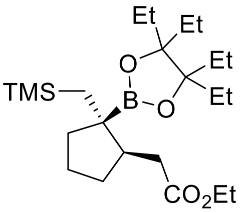

9c
dr = >20:1

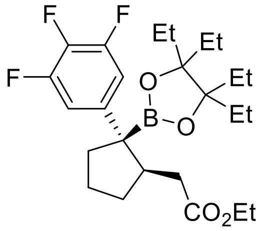

9d
dr = 2:1

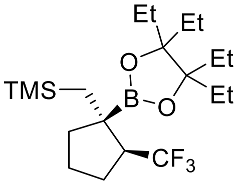

9e
dr = 7:1

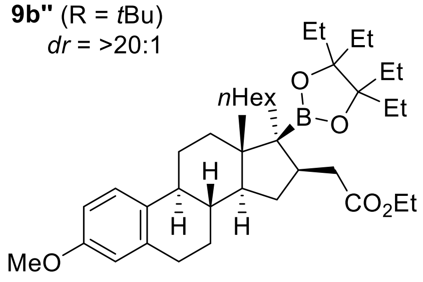

9f
dr = 3:1

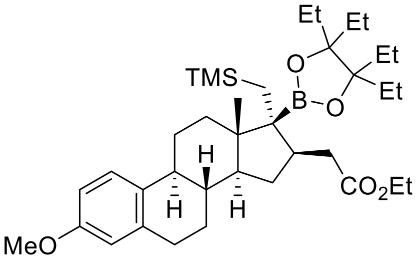

9g
dr = 2,5:1

Ethyl 2-((1*R*,2*S*)-2-butyl-2-(4,4,5,5-tetraethyl-1,3,2-dioxaborolan-2-yl)cyclopentyl)acetate (9a)

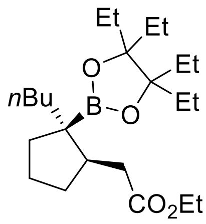

According to GP-C, using *n*BuLi (0.22 mmol, 1.1 equiv.) and ethyl iodoacetate (47 µL, 0.4 mmol, 2.0 equiv.). Purification *via* flash-column chromatography (SiO_2; Pentane – EtOAc: 97:3) yielded the title compound as a colorless oil (76 mg, 0.19 mmol, 96 %). ^1H NMR analysis of the crude reaction mixture indicated a diastereomeric ratio of 19:1.

^1H NMR (600 MHz, $CDCl_3$): δ (ppm) = 4.11 (q, J = 7.1 Hz, 2H), 2.60 (dd, J = 15.0, 3.5 Hz, 1H), 2.19 (dd, J = 15.0, 11.3 Hz, 1H), 1.97 – 1.85 (m, 2H), 1.84 – 1.78 (m, 1H), 1.72 – 1.53 (m, 11H), 1.31 – 1.14 (m, 9H), 1.04 (td, J = 12.3, 4.1 Hz, 1H), 0.92 – 0.84 (m, 15H).
^{13}C NMR (150 MHz, $CDCl_3$): δ (ppm) = 174.47, 88.18, 60.14, 46.87, 38.22, 37.76, 35.03, 32.04, 29.82, 26.37, 25.77, 23.96, 22.53, 14.45, 14.27, 9.07, 8.62.

HRMS (EI-orbitrap): *m/z*: [M] calc. for [$C_{21}H_{38}BO_4$]: 365.2863; found: 365.2841.
IR (Diamond-ATR, neat) $\tilde{\nu}_{max}$: 2934 (m), 2882 (m), 1736 (vs), 1458 (m), 1410 (m), 1388 (s), 1348 (m), 1288 (s), 1258 (s), 1222 (m), 1184 (s), 1152 (s), 1112 (s), 1030 (m), 994 (w), 956 (w), 922 (s), 856 (w), 796 (w), 774 (w), 728 (vw), 692 (w).

***tert*-Butyl 2-((1*R*,2*S*)-2-butyl-2-(4,4,5,5-tetraethyl-1,3,2-dioxaborolan-2-yl)cyclopentyl)acetate (9a')**

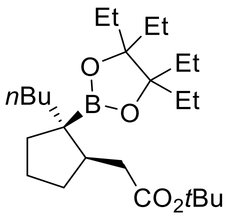

According to GP-C, using 2-(cyclopent-1-en-1-yl)-4,4,5,5-tetraethyl-1,3,2-dioxaborolane (0.8 g, 3.2 mmol, 1.0 equiv.) in THF (16 mL). *n*Butylllithium (3.5 mmol, 1.1 equiv.) was added over 30 min at -78°C and after 30 min at the same temperature the mixture was allowed to warm to 0°C for 1h. After solvent switch to methylTHF (33 mL) and addition of dodecane (1.5 mL, 5 Vol%.) *tert*-butyl iodoacetate (1.6 g, 6.4 mmol, 2.0 equiv.) was added dropwise to the mixture under irradiation at -40°C. Purification *via* flash-column chromatography (SiO_2; Pentane – EtOAc: 98:2) yielded the title compound as a colorless oil (0.92 g, 2.2 mmol, 68 %). ^{1}H NMR analysis of the crude reaction mixture indicated a diastereomeric ratio of 13:1.

^{1}H NMR (600 MHz, CD_2Cl_2): δ (ppm) = 2.47 (dd, J = 14.6, 3.6 Hz, 1H), 2.42 – 2.36 (m, 1H), 2.05 (dd, J = 14.6, 11.2 Hz, 1H), 1.97 – 1.72 (m, 3H), 1.69 – 1.60 (m, 10H), 1.42 (s, 9H), 1.31 – 1.14 (m, 6H), 1.07 – 0.98 (m, 1H), 0.93 – 0.86 (m, 15H).
^{13}C NMR (150 MHz, CD_2Cl_2): δ (ppm) = 173.24, 88.09, 79.35, 54.00, 47.05, 38.84, 38.17, 34.78, 31.69, 29.72, 27.81, 27.33, 26.32, 26.17, 25.58, 23.87, 22.31, 13.90, 8.70, 8.59, 8.26.

HRMS (EI-orbitrap): *m/z*: [M] calc. for [$C_{21}H_{38}BO_4$]: 365.2863; found: 365.2868.

Ethyl 2-((1*R*,2*S*)-2-hexyl-2-(4,4,5,5-tetraethyl-1,3,2-dioxaborolan-2-yl)cyclopentyl)acetate (<u>9b</u>)

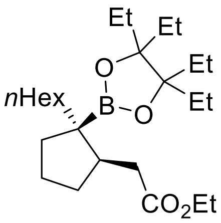

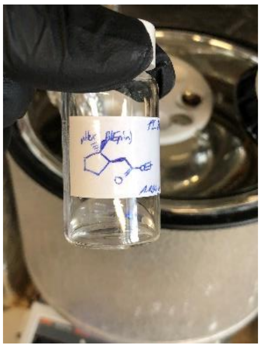

<u>0.2 mmol Scale:</u> According to GP-C, using hexyllithium (0.22 mmol, 1.1 equiv.) and ethyl iodoacetate (47 µL, 0.4 mmol, 2.0 equiv.). Purification *via* flash-column chromatography (SiO_2; Pentane – EtOAc: 96:4) yielded the title compound as a colorless oil (82 mg, 0.19 mmol, 97 %). ^{1}H NMR analysis of the crude reaction mixture indicated a diastereomeric ratio of <u>>20:1</u>.

<u>3.0 mmol Scale:</u> According to GP-C, using 2-(cyclopent-1-en-1-yl)-4,4,5,5-tetraethyl-1,3,2-dioxaborolane (**<u>7</u>**) (0.75 g, 3.0 mmol, 1.0 equiv.) in THF (15 mL). Hexyllithium (3.3 mmol, 1.1 equiv.) was added over 30 min at -78°C and after 30 min at the same temperature the mixture was allowed to warm to 0°C for 1h. After solvent switch to methylTHF (30 mL) and addition of dodecane (1.5 mL, 5 Vol%.) ethyl iodoacetate (0.71 mL, 6.0 mmol, 2.0 equiv.) was added dropwise to the mixture under irradiation at -40°C. Purification *via* flash-column chromatography (SiO_2; Pentane – EtOAc: 95:5) yielded the title compound as a colorless oil (1.2 g, 2.8 mmol, 95 %). ^{1}H NMR analysis of the crude reaction mixture indicated a diastereomeric ratio of <u>>20:1</u>.

^{1}H NMR (600 MHz, $CDCl_3$): δ (ppm) = 4.11 (q, J = 7.1 Hz, 2H), 2.59 (dd, J = 15.0, 3.5 Hz, 1H), 2.19 (dd, J = 15.0, 11.2 Hz, 1H), 1.98 – 1.85 (m, 2H), 1.84 – 1.77 (m, 1H), 1.72 – 1.53 (m, 11H), 1.32 – 1.13 (m, 13H), 1.04 (td, J = 12.8, 12.3, 4.0 Hz, 1H), 0.92 – 0.83 (m, 14H).
^{13}C NMR (150 MHz, $CDCl_3$): δ (ppm) = 174.47, 88.18, 60.14, 46.90, 38.55, 37.76, 35.03, 32.03, 32.00, 30.58, 27.52, 26.37, 25.79, 22.79, 22.52, 14.44, 14.27, 9.06, 8.64.

HRMS (EI-orbitrap): *m/z*: [M] calc. for [$C_{23}H_{43}BO_4$]: 394.3254; found: 394.3205.

IR (Diamond-ATR, neat) $\tilde{\nu}_{max}$: 2928 (s), 2884 (m), 2856 (m), 1736 (vs), 1458 (m), 1388 (s), 1348 (m), 1290 (s), 1258 (s), 1180 (s), 1150 (s), 1112 (s), 1032 (m), 976 (w), 956 (w), 922 (vs), 856 (w), 796 (w), 772 (w), 724 (w), 692 (w).

2-((1*R*,2*S*)-2-Hexyl-2-(4,4,5,5-tetraethyl-1,3,2-dioxaborolan-2-yl)cyclopentyl)acetic acid (9b')

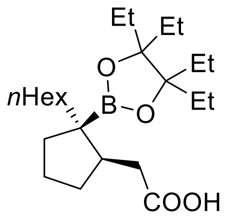

Ethyl 2-((1*R*,2*S*)-2-hexyl-2-(4,4,5,5-tetraethyl-1,3,2-dioxaborolan-2-yl)cyclopentyl)acetate **9b** (85 mg, 0.2 mmol, 1.0 equiv.) was dissolved in a mixture of H_2O (1 mL), THF (2 mL) and MeOH (2 mL) and treated with LiOH (0.12 g, 5 mmol, 25 equiv.). After stirring at rt. overnight, the solvents were removed *in vacuo*, and the residue was dissolved in Et_2O (10 mL) and filtered. Further concentration *in vacuo* and flash column chromatography (SiO_2; DCM – MeOH: 98:2) afforded the title compound as a colorless oil (56 mg, 0.18 mmol, 91%). ^{1}H NMR analysis of the crude reaction mixture indicated a diastereomeric ratio of ≥20:1.

^{1}H NMR (600 MHz, $CDCl_3$): δ (ppm) = 2.65 (dd, J = 15.3, 3.7 Hz, 1H), 2.28 (dd, J = 15.3, 10.9 Hz, 1H), 1.99 – 1.93 (m, 2H), 1.84 – 1.80 (m, 1H), 1.65 (s, 11H), 1.30 – 1.19 (m, 10H), 1.09 – 1.03 (m, 1H), 0.91 – 0.86 (m, 15H).
^{13}C NMR (150 MHz, $CDCl_3$): δ (ppm) = 179.38, 88.36, 46.54, 38.52, 35.09, 32.18, 31.99, 30.55, 27.51, 26.39, 25.81, 22.79, 22.49, 14.26, 9.05, 8.64.

HRMS (ESI) m/z: $[Na]^+$ calcd for $C_{23}H_{42}O_4B^+$: 393.3174; found: 393.3184.
IR (Diamond-ATR, neat) $\tilde{\nu}_{max}$: 2954 (m), 2924 (m), 2882 (m), 2858 (m), 2680 (w), 1700 (vs), 1454 (m), 1394 (m), 1364 (w), 1348 (m), 1320 (m), 1296 (s), 1250 (m), 1226 (w), 1210 (m), 1184 (w), 1158 (m), 1142 (w), 1112 (m), 1086 (w), 1058 (vw), 1028 (w), 974 (m), 954 (m), 922 (s), 854 (w), 818 (vw), 796 (vw), 774 (w), 724 (vw).

tert-Butyl 2-((1R,2S)-2-hexyl-2-(4,4,5,5-tetraethyl-1,3,2-dioxaborolan-2-yl)cyclopentyl)acetate (9b'')

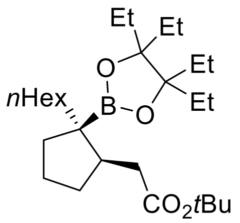

According to GP-C, using 2-(cyclopent-1-en-1-yl)-4,4,5,5-tetraethyl-1,3,2-dioxaborolane (**7**) (0.52 g, 2.1 mmol, 1.0 equiv.) in THF (11 mL). Hexyllithium (2.3 mmol, 1.1 equiv.) was added over 30 min at -78°C and after 30 min at the same temperature the mixture was allowed to warm to 0°C for 1h. After solvent switch to methylTHF (20 mL) and addition of dodecane (1 mL, 5 Vol%.) *tert*-butyl iodoacetate (1.0 g, 4.2 mmol, 2.0 equiv.) was added dropwise to the mixture under irradiation at -40°C. Purification *via* flash-column chromatography (SiO_2; Pentane – EtOAc: 97:3) yielded the title compound as a colorless oil (0.8 g, 1.8 mmol, 84 %). ^{1}H NMR analysis of the crude reaction mixture indicated a diastereomeric ratio of >20:1.

^{1}H NMR (600 MHz, CD_2Cl_2): δ (ppm) = 2.39 (dd, J = 14.5, 3.7 Hz, 1H), 2.34 – 2.28 (m, 1H), 1.97 (dd, J = 14.6, 11.2 Hz, 1H), 1.87 – 1.65 (m, 3H), 1.62 – 1.52 (m, 10H), 1.34 (s, 9H), 1.22 – 1.15 (m, 8H), 1.12 – 1.04 (m, 2H), 0.97 – 0.90 (m, 1H), 0.86 – 0.76 (m, 15H).
^{13}C NMR (150 MHz, CD_2Cl_2): δ (ppm) = 173.24, 88.09, 79.34, 47.07, 38.84, 38.50, 34.79, 31.87, 31.69, 30.47, 27.82, 27.42, 26.32, 26.17, 25.60, 22.65, 22.31, 13.90, 8.70, 8.59, 8.28.

HRMS (EI-orbitrap): *m/z*: [M] calc. for [$C_{23}H_{42}BO_4$]: 393.3176; found: 393.3168.

Ethyl 2-((1*R*,2*S*)-2-(4,4,5,5-tetraethyl-1,3,2-dioxaborolan-2-yl)-2-((trimethylsilyl)methyl)cyclopentyl)acetate (9c)

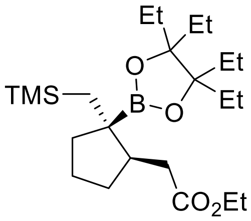

According to GP-C, using (trimethylsilyl)methyllithium (0.22 mmol, 1.1 equiv.) and ethyl iodoacetate (47 µL, 0.4 mmol, 2.0 equiv.). Purification *via* flash-column chromatography (SiO_2; Pentane – EtOAc: 96:4) yielded the title compound as a colorless oil (66 mg, 0.16 mmol, 78 %). ^{1}H NMR analysis of the crude reaction mixture indicated a diastereomeric ratio of ≥20:1.

^{1}H NMR (600 MHz, $CDCl_3$): δ (ppm) = 4.11 (qd, J = 7.1, 1.1 Hz, 2H), 2.59 (dd, J = 14.8, 3.3 Hz, 1H), 2.17 (dd, J = 14.8, 11.4 Hz, 1H), 1.97 – 1.92 (m, 1H), 1.89 – 1.83 (m, 1H), 1.76 – 1.56 (m, 13H), 1.29 – 1.20 (m, 5H), 0.92 – 0.85 (m, 12H), 0.01 (s, 9H).
^{13}C NMR (150 MHz, $CDCl_3$): δ (ppm) = 174.56, 88.30, 60.15, 51.36, 37.07, 36.78, 30.96, 26.38, 26.07, 25.17, 22.32, 14.45, 8.86, 0.94.

HRMS (EI-orbitrap): *m/z*: [M] calc. for [$C_{21}H_{40}BO_4Si$]: 395.2789; found: 395.2769.
IR (Diamond-ATR, neat) $\tilde{\nu}_{max}$: 2950 (m), 2884 (w), 1736 (s), 1458 (w), 1410 (w), 1384 (m), 1348 (m), 1290 (m), 1246 (s), 1202 (w), 1172 (m), 1140 (m), 1112 (m), 1074 (w), 1030 (m), 990 (w), 958 (vw), 920 (m), 856 (s), 836 (vs), 798 (m), 762 (w).

Ethyl 2-((1*R*,2*S*)-2-(4,4,5,5-tetraethyl-1,3,2-dioxaborolan-2-yl)-2-(3,4,5-trifluorophenyl)cyclopentyl)acetate (9d)

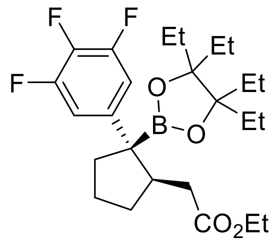

According to GP-C, using 5-bromo-1,2,3-trifluorobenzene (26 µL, 0.22 mmol, 1.1 equiv.) and *n*BuLi (0.22 mmol, 1.1 equiv.) for Halogen-Lithium exchange at -78°C for 30min. Radical precursor ethyl iodoacetate (47 µL, 0.4 mmol, 2.0 equiv.) was finally added to the reaction mixture in methyl-THF (0.5 mL). Purification *via* column chromatography (SiO_2; Pentane – EtOAc: 96:4) yielded the title compound as a colorless oil (88 mg, 0.19 mmol, 93%). ^{1}H NMR and GC analysis of the crude reaction mixture indicated a diastereomeric ratio of 2:1. Separation of Diastereomers is possible *via* column chromatography.

^{1}H NMR (400 MHz, $CDCl_3$): δ (ppm) = 6.97 – 6.91 (m, 2H), 4.13 (q, J = 7.2 Hz, 2H), 2.44 – 2.40 (m, 1H), 2.25 – 2.17 (m, 1H), 2.04 – 1.93 (m, 1H), 1.77 – 1.54 (m, 12H), 1.49 – 1.41 (m, 1H), 1.26 (t, J = 7.1 Hz, 4H), 0.92 – 0.82 (m, 12H).

^{13}C NMR (100 MHz, $CDCl_3$): δ (ppm) = 173.63, 111.34, 111.12, 89.18, 60.45, 45.27, 37.60, 36.87, 31.34, 26.15, 25.88, 22.25, 14.40, 8.68.

^{19}F NMR (400 MHz, $CDCl_3$): δ (ppm) = -135.47, -135.49, -135.52, -135.55, -135.62, -135.64, -135.67, -135.70, -165.20, -165.22, -165.23, -165.25, -165.27, -165.30, -165.31, -165.33, -165.35, -165.37, -165.39, -165.41, -165.42, -165.44.

HRMS (ESI) m/z: $[HCO_2]^+$ calcd for $C_{25}H_{36}BF_3O_4HCO_2^+$: 513.2635; found: 513.2644.

IR (Diamond-ATR, neat) $\tilde{\nu}_{max}$: 2948 (m), 2884 (w), 1734 (s), 1614 (m), 1590 (vw), 1528 (vs), 1458 (m), 1430 (m), 1368 (m), 1338 (s), 1300 (s), 1266 (s), 1240 (s), 1186 (s), 1152 (s), 1112 (m), 1036 (vs), 1000 (w), 954 (w), 916 (s), 852 (m), 802 (vw), 772 (w), 706 (w).

Trimethyl(((1*S*,2*S*)-1-(4,4,5,5-tetraethyl-1,3,2-dioxaborolan-2-yl)-2-(trifluoromethyl)cyclopentyl)methyl)silane (9e)

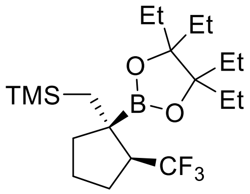

According to GP-C, using (trimethylsilyl)methyllithium (0.22 mmol, 1.1 equiv.) and Ritter trifluoroiodomethane (0.28 g, 0.8 mmol, 4.0 equiv.). Purification *via* flash-column chromatography (SiO_2; Pentane – EtOAc: 99:1; long column) yielded the title compound as a colorless oil (44 mg, 0.11 mmol, 54 %). ^{1}H NMR analysis of the crude reaction mixture indicated a diastereomeric ratio of 7:1.

^{1}H NMR (600 MHz, CD_2Cl_2): δ (ppm) = 2.26 – 2.15 (m, 1H), 2.01 – 1.80 (m, 4H), 1.74 – 1.61 (m, 8H), 1.42 – 1.26 (m, 3H), 0.88 (dt, J = 8.6, 7.5 Hz, 12H), 0.57 (d, J = 14.5 Hz, 1H), 0.03 (s, 9H).
^{13}C NMR (150 MHz, CD_2Cl_2): δ (ppm) = 89.19, 38.51, 27.32, 26.40, 25.65, 22.77, 9.42, 8.64, 1.23.
^{19}F NMR (400 MHz, CD_2Cl_2): δ (ppm) = -66.19, -66.22.

HRMS (ESI) m/z: $[Na]^+$ calcd for $C_{20}H_{38}BF_3O_2SiNa^+$: 429.2584; found: 429.2592.

Ethyl 2-((8*S*,9*S*,13*S*,14*S*,16*R*,17*R*)-17-hexyl-3-methoxy-13-methyl-17-(4,4,5,5-tetraethyl-1,3,2-dioxaborolan-2-yl)-7,8,9,11,12,13,14,15,16,17-decahydro-6H-cyclopenta[a]phenanthren-16-yl)acetate (9f)

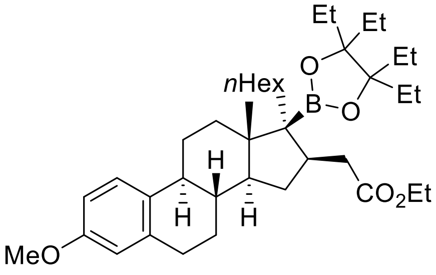

A flame-dried Schlenk flask was charged with 4,4,5,5-tetraethyl-2-((8*S*,9*S*,13*S*,14*S*)-3-methoxy-13-methyl-7,8,9,11,12,13,14,15-octahydro-6H-cyclopenta[a]phenanthren-17-yl)-1,3,2-dioxaborolane (**Si-27**) (45 mg, 0.1 mmol, 1.0 equiv.) and THF (0.5 mL) was added. The solution was cooled to -78°C and (trimethylsilyl)methyllithium in pentane (0.11 mmol, 1.1 equiv.) was added dropwise. Stirring was continued at this temperature for 30 min, before the mixture was allowed to warm to 0°C for 1 h. Following this, the solvents were carefully removed *in vacuo*. The residue was dissolved in 2-methyltetrahydrofuran (1 mL), dodecane (50 µL, 5 Vol%.) was added and the flask was transferred to the photoreactor precooled to -40 °C. Ethyl iodoacetate (0.71 mL, 6.0 mmol, 2.0 equiv.) was added dropwise to the mixture under irradiation at -40°C). The colorless solution was stirred at the indicated temperature under LED irradiation for 14 h. Purification *via* column chromatography (SiO_2; Pentane – EtOAc: 98:2 to 96:4) yielded the title compound as a colorless oil (48 mg, 0.09 mmol, 89%). ^{1}H NMR analysis of the crude reaction mixture indicated a diastereomeric ratio of 3:1.

^{1}H NMR (800 MHz, CD_2Cl_2): δ (ppm) = 7.19 – 7.15 (m, 1H), 6.65 (dd, J = 8.6, 2.8 Hz, 1H), 6.59 (d, J = 2.8 Hz, 1H), 4.17 (q, J = 7.1 Hz, 2H), 4.12 – 4.06 (m, 2H), 3.73 (s, 3H), 3.69 (s, 2H), 2.86 – 2.77 (m, 2H), 2.23 – 2.12 (m, 2H), 1.81 – 1.60 (m, 11H), 1.49 – 1.23 (m, 20H), 0.93 – 0.86 (m, 16H).

^{13}C NMR (200 MHz, CD_2Cl_2): δ (ppm) = 174.60, 169.22, 157.93, 138.73, 138.69, 133.68, 133.66, 126.62, 114.10, 111.71, 88.89, 88.67, 88.47, 62.64, 60.42, 55.61, 50.78, 49.31, 47.73, 46.93, 44.38, 44.26, 43.95, 40.36, 39.88, 39.66, 39.24, 38.91, 37.05, 36.04, 33.81, 33.60, 32.45, 32.41, 32.15, 31.73, 31.57, 30.63, 30.49, 30.44, 29.53, 28.89, 28.67, 28.36, 27.12, 27.01, 26.42, 26.10, 25.92, 23.31, 23.26, 18.99, 14.69, 14.47, 14.21, 9.43, 9.17, 8.92, 8.83.

HRMS (EI-orbitrap): *m/z*: [M] calc. for [$C_{39}H_{63}BO_5$]: 622.4769; found: 622.4790.

IR (Diamond-ATR, neat) $\tilde{\nu}_{max}$: 2930 (m), 1734 (s), 1610 (w), 1576 (vw), 1500 (m), 1458 (m), 1418 (w), 1384 (m), 1368 (m), 1348 (m), 1254 (vs), 1176 (s), 1158 (m), 1140 (m), 1112 (s), 1032 (s), 980 (w), 958 (w), 922 (s), 858 (w), 816 (w), 786 (w), 724 (vw).

Ethyl 2-((8*S*,9*S*,13*S*,14*S*,16*R*,17*R*)-3-methoxy-13-methyl-17-(4,4,5,5-tetraethyl-1,3,2-dioxaborolan-2-yl)-17-((trimethylsilyl)methyl)-7,8,9,11,12,13,14,15,16,17-decahydro-6H-cyclopenta[a]phenanthren-16-yl)acetate (9g)

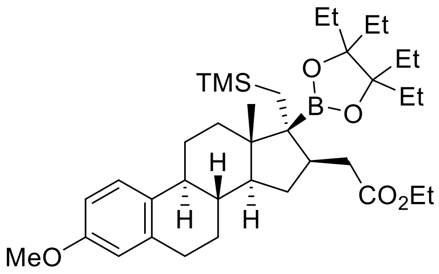

A flame-dried Schlenk flask was charged with 4,4,5,5-Tetraethyl-2-((8*S*,9*S*,13*S*,14*S*)-3-methoxy-13-methyl-7,8,9,11,12,13,14,15-octahydro-6H-cyclopenta[a]phenanthren-17-yl)-1,3,2-dioxaborolane (**Si-27**) (45 mg, 0.1 mmol, 1.0 equiv.) and THF (0.5 mL) was added. The solution was cooled to -78°C and (trimethylsilyl)methyllithium in pentane (0.11 mmol, 1.1 equiv.) was added dropwise. Stirring was continued at this temperature for 30 min, before the mixture was allowed to warm to 0°C for 1 h. Following this, the solvents were carefully removed *in vacuo*. The residue was dissolved in 2-methyltetrahydrofuran (1 mL), dodecane (50 µL, 5 Vol%.) was added and the flask was transferred to the photoreactor precooled to -40 °C. Ethyl iodoacetate (0.71 mL, 6.0 mmol, 2.0 equiv.) was added dropwise to the mixture under irradiation at -40°C). The colorless solution was stirred at the indicated temperature under LED irradiation for 14 h. Purification *via* column chromatography (SiO_2; Pentane – EtOAc: 98:2 to 95:5) yielded the title compound as a colorless oil that solidified in the freezer (36 mg, 0.06 mmol, 57%). ^{1}H NMR analysis of the crude reaction mixture indicated a diastereomeric ratio of 2,5:1.

^{1}H NMR (600 MHz, CD_2Cl_2): δ (ppm) = 7.18 – 7.16 (m, 1H), 6.67 (td, J = 8.1, 2.7 Hz, 1H), 6.61 (dd, J = 6.8, 2.7 Hz, 1H), 3.74 (d, J = 1.5 Hz, 3H), 2.91 – 2.86 (m, 2H), 2.29 – 2.25 (m, 4H), 2.08 – 2.03 (m, 2H), 1.95 – 1.91 (m, 2H), 1.71 – 1.59 (m, 13H), 1.45 – 1.42 (m, 2H), 1.41 (s, 3H), 1.26 (t, J = 3.5 Hz, 1H), 0.95 – 0.87 (m, 15H), 0.78 (d, J = 24.5 Hz, 9H), 0.08 (s, 2H).
^{13}C NMR (150 MHz, CD_2Cl_2): δ (ppm) = 158.09, 157.94, 145.97, 144.50, 138.59, 138.44, 138.18, 133.82, 133.68, 133.06, 129.77, 126.51, 126.45, 125.96, 114.19, 113.16, 111.81, 111.73, 111.72, 88.25, 56.57, 55.99, 55.62, 50.81, 48.58, 46.31, 45.15, 44.71, 38.44, 38.25, 38.00, 36.92, 33.94, 33.79, 32.26, 30.63, 30.34, 30.19, 28.83, 28.55, 27.99, 27.40, 27.22, 26.92, 26.74, 17.42, 17.26, 15.66, 9.24, 9.14, 1.32, 0.52.

HRMS (EI-orbitrap): *m/z*: [M] calc. for [$C_{37}H_{61}BO_5Si$]: 624.4381; found: 624.4396.

IR (Diamond-ATR, neat) $\tilde{\nu}_{max}$: 2922 (s), 2880 (m), 2850 (m), 2812 (w), 1736 (w), 1608 (m), 1578 (w), 1500 (s), 1452 (m), 1406 (w), 1370 (m), 1358 (m), 1312 (m), 1284 (s), 1246 (vs), 1236 (vs), 1198 (m), 1176 (m), 1158 (m), 1130 (m), 1112 (m), 1094 (s), 1068 (m), 1042 (vs), 1032 (vs), 992 (m), 976 (m), 954 (m), 926 (m), 902 (m), 878 (w), 856 (s), 842 (m), 822 (s), 788 (m), 706 (m), 694 (m).

Others:

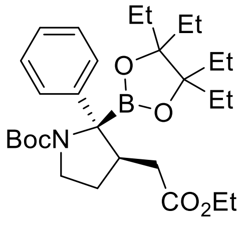

12a
dr = 2:1

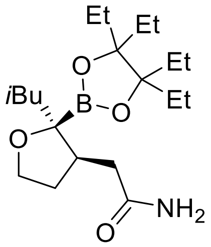

12b
dr = 3:1

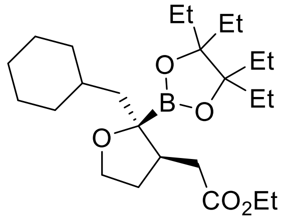

12c
dr = 3:1

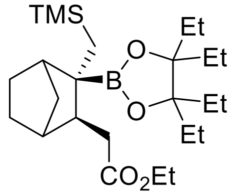

15a
dr = 4:1

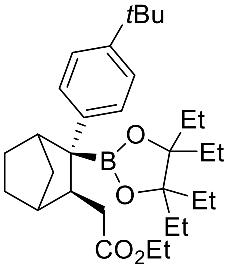

15b
dr = >20:1

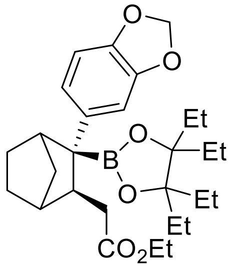

15c
dr = >20:1

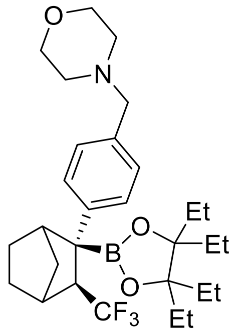

15d
dr = >20:1

tert-Butyl (2_S_,3_R_)-3-(2-ethoxy-2-oxoethyl)-2-phenyl-2-(4,4,5,5-tetraethyl-1,3,2-dioxaborolan-2-yl)pyrrolidine-1-carboxylate (<u>12a</u>)

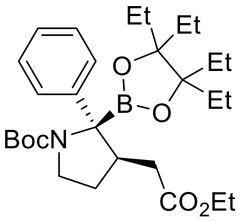

A flame-dried Schlenk flask was charged with *tert*-butyl 5-(4,4,5,5-tetraethyl-1,3,2-dioxaborolan-2-yl)-2,3-dihydro-1H-pyrrole-1-carboxylate (70 mg, 0.2 mmol, 1.0 equiv.) and THF (0.5 mL) was added. The solution was cooled to -78°C and phenyllithium (0.22 mmol, 1.1 equiv.) was added dropwise. Stirring was continued at this temperature for 30 min, before the mixture was allowed to warm to 0°C for 45 min. Following this, the solvents were carefully removed *in vacuo*. The residue was dissolved in 2-methyltetrahydrofuran (1.5 mL), dodecane (0.1 mL, 5 Vol%.) was added and the flask was transferred to the photoreactor precooled to -40 °C. Ethyl iodoacetate (47 µL, 0.4 mmol, 2.0 equiv.) was dissolved in 2-methylTHF (0.5 mL) and added dropwise to the mixture under irradiation at -40°C. The colorless solution was stirred at the indicated temperature under LED irradiation for 12 h. The crude mixture was pushed through a Silica-Plug (4 cm), eluting with Et_2O (50 mL) and was concentrated *in vacuo*. Flash column chromatography (SiO_2; pentane – EtOAc: 93:7) afforded the title compound as a viscous colorless oil (75 mg, 0.15 mmol, 73 %). ^{1}H NMR and GC analysis of the crude reaction mixture indicated a diastereomeric ratio of <u>2:1</u>. The title compound was obtained in a mixture of *N*-Boc Rotamers in a ratio of 3:1, as indicated by ^{1}H NMR.

^{1}H NMR (800 MHz, Acetone-D$_6$): δ (ppm) = 7.37 – 7.34 (m, 2H), 7.33 – 7.30 (m, 1H), 7.28 – 7.26 (m, 2H), 4.13 – 4.01 (m, 2H), 3.83 – 3.49 (m, 2H), 2.91 (dd, J = 16.1, 5.5 Hz, 1H), 2.58 – 2.51 (m, 1H), 2.36 (ddd, J = 26.4, 15.7, 10.3 Hz, 1H), 1.90 – 1.73 (m, 10H), 1.47 (d, J = 24.5 Hz, 9H), 1.22 (t, J = 7.1 Hz, 3H), 0.93 (t, J = 7.4 Hz, 6H), 0.87 (t, J = 7.5 Hz, 6H).
^{13}C NMR (200 MHz, Acetone-D$_6$): δ (ppm) = 172.99, 172.37, 172.08, 155.58, 155.02, 154.26, 147.74, 146.84, 128.59, 128.43, 128.29, 128.06, 126.74, 126.59, 126.49, 126.25, 90.31, 89.90, 89.75, 79.62, 79.21, 60.84, 60.77, 60.72, 52.57, 49.20, 47.73, 47.15, 46.37, 43.74, 36.71, 36.54, 35.57, 31.20, 30.52, 28.79, 28.36, 28.26, 26.66, 26.54, 26.40, 26.15, 25.88, 14.50, 14.43, 9.35, 9.21, 9.17, 9.05.

HRMS (ESI) m/z: $[Na]^+$ calcd for $C_{29}H_{46}BNO_6Na^+$: 538.3316; found: 538.3323.

IR (Diamond-ATR, neat) $\tilde{\nu}_{max}$: 2976 (m), 2942 (m), 2884 (w), 1734 (s), 1686 (vs), 1602 (vw), 1492 (w), 1478 (w), 1456 (m), 1394 (vs), 1364 (s), 1332 (m), 1296 (m), 1268 (m), 1244 (s), 1164 (vs), 1114 (s), 1084 (m), 1060 (w), 1030 (m), 976 (m), 958 (w), 922 (s), 856 (w), 776 (m), 766 (m), 740 (m).

2-((2*R*,3*R*)-2-Isobutyl-2-(4,4,5,5-tetraethyl-1,3,2-dioxaborolan-2-yl)tetrahydrofuran-3-yl)acetamide (12b)

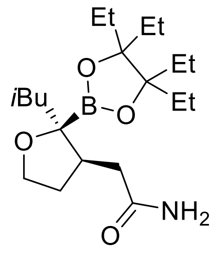

According to GP-D, using 4,4,5,5-tetraethyl-2-isobutyl-1,3,2-dioxaborolane (**Si-26**) (62 mg, 0.26 mmol, 1.3 equiv.) and iodoacetamide (74 mg, 0.4 mmol, 2.0 equiv.). Flash column chromatography (SiO_2; pentane – EtOAc: 1:1; 3% TEA) afforded the title compound as a light orange oil (46 mg, 0.13 mmol, 63 %). ^{1}H NMR analysis of the crude reaction mixture indicated a diastereomeric ratio of 3:1.

^{1}H NMR (800 MHz, CD_2Cl_2): δ (ppm) = 5.50 (t, 1H), 3.88 – 3.73 (m, 2H), 2.43 – 2.37 (m, 1H), 2.21 – 2.14 (m, 1H), 2.14 – 2.10 (m, 1H), 2.03 – 1.98 (m, 1H), 1.79 – 1.55 (m, 11H), 1.31 – 1.25 (m, 2H), 0.95 – 0.86 (m, 18H).
^{13}C NMR (200 MHz, CD_2Cl_2): δ (ppm) = 174.83, 89.92, 89.57, 66.29, 65.77, 46.46, 46.29, 43.20, 40.04, 38.60, 36.19, 33.03, 31.72, 27.06, 26.73, 26.51, 26.28, 26.01, 24.85, 24.20, 24.02, 23.98, 9.26, 9.03, 8.76.

HRMS (ESI) m/z: $[Na]^+$ calcd for $C_{20}H_{38}BNO_4Na^+$: 390.2792; found: 390.2792.
IR (Diamond-ATR, neat) $\tilde{\nu}_{max}$: 3198 (w), 2972 (m), 2950 (m), 2884 (m), 1740 (w), 1670 (vs), 1620 (m), 1456 (m), 1432 (m), 1396 (s), 1352 (s), 1306 (m), 1288 (m), 1240 (m), 1182 (w), 1146 (m), 1110 (s), 1040 (s), 988 (w), 958 (w), 916 (vs), 854 (w), 792 (w), 770 (w).

Ethyl 2-((2*R*,3*R*)-2-(cyclohexylmethyl)-2-(4,4,5,5-tetraethyl-1,3,2-dioxaborolan-2-yl)tetrahydrofuran-3-yl)acetate (12c)

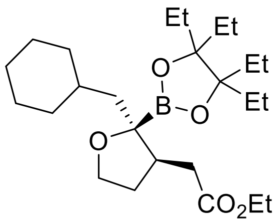

According to GP-D, using 4,4,5,5-tetraethyl-2-isobutyl-1,3,2-dioxaborolane (**Si-25**) (62 mg, 0.26 mmol, 1.3 equiv.) and ethyl iodoacetate (47 µL, 0.4 mmol, 2.0 equiv.). Flash column chromatography (SiO_2; pentane – EtOAc: 98:2 to 93:7) afforded the title compound as a colorless oil (84 mg, 0.19 mmol, 96 %). ^{1}H NMR and GC analysis of the crude reaction mixture indicated a diastereomeric ratio of 3:1. The two diastereomers were conveniently separated *via* column chromatography. Analytics are reported for the depicted major diastereomer.

^{1}H NMR (800 MHz, $CDCl_3$): δ (ppm) = 4.12 (q, J = 7.1 Hz, 2H), 3.88 – 3.81 (m, 2H), 2.66 (dd, J = 15.2, 3.2 Hz, 1H), 2.46 – 2.41 (m, 1H), 2.16 – 2.09 (m, 2H), 1.87 – 1.82 (m, 1H), 1.74 – 1.61 (m, 13H), 1.40 – 1.35 (m, 1H), 1.25 (t, J = 7.1 Hz, 4H), 1.22 – 1.18 (m, 2H), 1.12 (dd, J = 13.5, 6.0 Hz, 1H), 0.91 – 0.88 (m, 15H).
^{13}C NMR (200 MHz, $CDCl_3$): δ (ppm) = 173.34, 89.20, 66.09, 60.39, 42.47, 34.81, 34.69, 34.34, 31.18, 26.61, 25.92, 25.82, 14.37, 8.85, 8.78.

HRMS (ESI) m/z: $[Na]^+$ calcd for $C_{25}H_{45}BO_5Na^+$: 459.3258; found: 459.3261.
IR (Diamond-ATR, neat) $\tilde{\nu}_{max}$: 2922 (s), 2850 (m), 1736 (vs), 1450 (m), 1396 (m), 1368 (m), 1352 (m), 1302 (s), 1288 (s), 1252 (m), 1208 (m), 1170 (s), 1144 (s), 1110 (s), 1030 (s), 994 (w), 970 (w), 956 (w), 922 (s), 892 (m), 854 (w), 792 (w), 770 (w), 698 (w).

Ethyl 2-((2*S*,3*S*)-3-(4,4,5,5-tetraethyl-1,3,2-dioxaborolan-2-yl)-3-((trimethylsilyl)methyl)bicyclo[2.2.1]heptan-2-yl)acetate (15a)

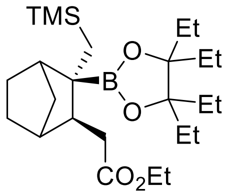

A flame-dried Schlenk flask was charged with 2-(bicyclo[2.2.1]hept-2-en-2-yl)-4,4,5,5-tetraethyl-1,3,2-dioxaborolane (**13**) (55 mg, 0.2 mmol, 1.0 equiv.) and THF (1 mL) was added. The solution was cooled to -78°C and (trimethylsilyl)methyllithium (0.22 mmol, 1.1 equiv.) was added dropwise. Stirring was continued at this temperature for 30 min, before the mixture was allowed to warm to 0°C for 45 min. Following this, the solvents were carefully removed *in vacuo*. The residue was dissolved in 2-methyltetrahydrofuran (1.5 mL), dodecane (0.1 mL, 5 Vol%.) was added and the flask was transferred to the photoreactor precooled to -40 °C. Ethyl iodoacetate (47 µL, 0.4 mmol, 2.0 equiv.) was dissolved in 2-methylTHF (0.5 mL) and added dropwise to the mixture under irradiation at -40°C. The colorless solution was stirred at the indicated temperature under LED irradiation for 12 h. The crude mixture was pushed through a Silica-Plug (4 cm), eluting with Et_2O (50 mL) and was concentrated *in vacuo*. Flash column chromatography (SiO_2; pentane – EtOAc: 97:3) afforded the title compound as a viscous colorless oil (71 mg, 0.16 mmol, 79 %). ^{1}H NMR analysis of the crude reaction mixture indicated a diastereomeric ratio of 4:1.

^{1}H NMR (600 MHz, CD_2Cl_2): δ (ppm) = 4.13 – 4.01 (m, 2H), 2.32 – 2.03 (m, 4H), 1.91 – 1.78 (m, 2H), 1.73 – 1.58 (m, 10H), 1.42 – 1.29 (m, 2H), 1.22 (t, J = 7.1 Hz, 2H), 1.03 – 0.95 (m, 1H), 0.93 – 0.84 (m, 15H), 0.09 – 0.01 (m, 9H).
^{13}C NMR (150 MHz, CD_2Cl_2): δ (ppm) = 174.54, 88.55, 88.23, 60.63, 60.39, 47.94, 46.06, 43.27, 36.89, 35.16, 30.65, 27.46, 27.23, 26.71, 26.40, 26.21, 20.54, 14.61, 9.20, 9.13, 9.07, 9.03, 1.18, 0.51.

HRMS (ESI) m/z: $[Na]^+$ calcd for $C_{25}H_{47}BO_4SiNa^+$: 473.3234; found: 473.3241
IR (Diamond-ATR, neat) $\tilde{\nu}_{max}$: 2948 (m), 2884 (w), 1736 (m), 1458 (w), 1382 (m), 1366 (m), 1346 (m), 1284 (m), 1246 (m), 1224 (w), 1170 (m), 1142 (m), 1112 (m), 1030 (m), 990 (w), 958 (vw), 922 (m), 856 (s), 836 (vs), 798 (w), 770 (w), 752 (w), 688 (w).

Ethyl 2-((2*S*,3*S*)-3-(4-(*tert*-butyl)phenyl)-3-(4,4,5,5-tetraethyl-1,3,2-dioxaborolan-2-yl)bicyclo[2.2.1]heptan-2-yl)acetate (15b)

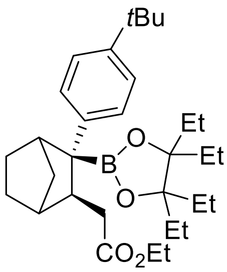

A flame-dried Schlenk flask was charged with 1-*tert*-butyl-4-iodobenzene (39 µL, 0.22 mmol, 1.0 equiv.) and THF (1 mL) was added. After cooling to -78°C, *n*BuLi (0.22 mmol, 1.1 equiv.) was added dropwise and the mixture was allowed to stir for 30min. 2-(Bicyclo[2.2.1]hept-2-en-2-yl)-4,4,5,5-tetraethyl-1,3,2-dioxaborolane (**13**) (55 mg, 0.2 mmol, 1.0 equiv.) was dissolved in THF (0.2 mL) and added dropwise. Stirring was continued at this temperature for 30min, before the mixture was allowed to warm to 0°C for 45 min. Following this, the solvents were carefully removed *in vacuo*. The residue was dissolved in 2-methyltetrahydrofuran (1.5 mL), dodecane (0.1 mL, 5 Vol%.) was added and the flask was transferred to the photoreactor precooled to -40 °C. Ethyl iodoacetate (47 µL, 0.4 mmol, 2.0 equiv.) was dissolved in 2-methylTHF (0.5 mL) and added dropwise to the mixture under irradiation at -40°C. The colorless solution was stirred at the indicated temperature under LED irradiation for 12 h. The crude mixture was pushed through a Silica-Plug (4 cm), eluting with Et_2O (50 mL) and was concentrated *in vacuo*. Flash column chromatography (SiO_2; pentane – EtOAc: 96:4) afforded the title compound as a viscous colorless oil (91 mg, 0.18 mmol, 92 %). ^{1}H NMR analysis of the crude reaction mixture indicated a diastereomeric ratio of >20:1

^{1}H NMR (400 MHz, DCM-D_2): δ (ppm) = 7.29 – 7.24 (m, 2H), 7.13 – 7.08 (m, 2H), 4.14 (q, J = 7.1 Hz, 2H), 3.43 (q, J = 7.0 Hz, 1H), 2.99 (dd, J = 15.2, 3.2 Hz, 1H), 2.92 – 2.89 (m, 1H), 2.33 – 2.28 (m, 1H), 2.16 (dd, J = 15.2, 12.3 Hz, 1H), 2.00 – 1.97 (m, 1H), 1.63 – 1.57 (m, 1H), 1.56 – 1.49 (m, 3H), 1.47 – 1.40 (m, 3H), 1.36 – 1.23 (m, 17H), 1.09 – 1.02 (m, 1H), 0.79 (t, J = 7.6 Hz, 6H), 0.63 (t, J = 7.6 Hz, 6H).

^{13}C NMR (100 MHz, DCM-D_2): δ (ppm) = 174.10, 147.87, 143.06, 128.08, 125.18, 88.86, 66.22, 60.56, 48.56, 43.91, 42.90, 40.80, 37.47, 34.61, 31.68, 30.92, 26.51, 25.63, 24.39, 15.65, 14.68, 9.32, 8.34.

HRMS (ESI) m/z: $[Na]^+$ calcd for $C_{31}H_{49}BO_4Na^+$: 519.3622 found: 519.3630.

IR (Diamond-ATR, neat) $\tilde{\nu}_{max}$: 2962 (s), 2872 (m), 1734 (vs), 1512 (w), 1458 (m), 1412 (w), 1362 (s), 1348 (m), 1326 (s), 1280 (vs), 1250 (s), 1206 (m), 1160 (s), 1144 (s), 1114 (s), 1032 (s), 998 (w), 956 (w), 924 (vs), 890 (w), 856 (w), 830 (m), 798 (w), 774 (w), 744 (vw), 722 (vw).

Ethyl 2-((2*S*,3*S*)-3-(benzo[d][1,3]dioxol-5-yl)-3-(4,4,5,5-tetraethyl-1,3,2-dioxaborolan-2-yl)bicyclo[2.2.1]heptan-2-yl)acetate (<u>15c</u>)

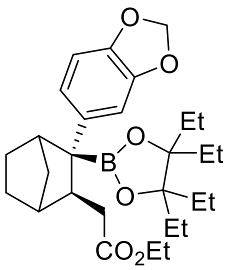

A flame-dried Schlenk flask was charged with 4-bromo-1,2-(methylenedioxy)benzene (26 µL, 0.22 mmol, 1.0 equiv.) and THF (1 mL) was added. After cooling to -78°C, *n*BuLi (0.22 mmol, 1.1 equiv.) was added dropwise and the mixture was allowed to stir for 20min. 2-(Bicyclo[2.2.1]hept-2-en-2-yl)-4,4,5,5-tetraethyl-1,3,2-dioxaborolane (**13**) (55 mg, 0.2 mmol, 1.0 equiv.) was dissolved in THF (0.2 mL) and added dropwise. Stirring was continued at this temperature for 30min, before the mixture was allowed to warm to 0°C for 45 min. Following this, the solvents were carefully removed *in vacuo*. The residue was dissolved in 2-methyltetrahydrofuran (1.5 mL), dodecane (0.1 mL, 5 Vol%.) was added and the flask was transferred to the photoreactor precooled to -40 °C. Ethyl iodoacetate (47 µL, 0.4 mmol, 2.0 equiv.) was dissolved in 2-methylTHF (0.5 mL) and added dropwise to the mixture under irradiation at -40°C. The colorless solution was stirred at the indicated temperature under LED irradiation for 12 h. The crude mixture was pushed through a Silica-Plug (4 cm), eluting with Et_2O (50 mL) and was concentrated *in vacuo*. Flash column chromatography (SiO_2; pentane – EtOAc: 95:5) afforded the title compound as a viscous colorless oil (70 mg, 0.14 mmol, 72 %). ^{1}H NMR of the crude reaction mixture indicated a diastereomeric ratio of <u>>20:1.</u>

^{1}H NMR (400 MHz, DCM-D_2): δ (ppm) = 6.73 – 6.69 (m, 2H), 6.64 (dd, J = 8.2, 1.8 Hz, 1H), 5.89 (q, J = 1.4 Hz, 2H), 4.20 – 4.09 (m, 2H), 2.96 (dd, J = 13.9, 1.8 Hz, 1H), 2.84 (dd, J = 3.9, 1.6 Hz, 1H), 2.27 – 2.10 (m, 2H), 1.97 (dd, J = 3.9, 1.6 Hz, 1H), 1.61 – 1.35 (m, 11H), 1.33 – 1.23 (m, 5H), 1.11 – 1.01 (m, 1H), 0.82 (t, J = 7.5 Hz, 6H), 0.70 (t, J = 7.5 Hz, 6H).
^{13}C NMR (100 MHz, DCM-D_2): δ (ppm) = 174.01, 147.79, 145.17, 140.39, 121.31, 109.21, 108.02, 101.30, 88.94, 62.63, 60.58, 48.94, 44.35, 43.00, 40.72, 37.44, 30.93, 26.40, 25.80, 24.24, 14.67, 14.20, 9.22, 8.54.

HRMS (ESI) m/z: $[Na]^+$ calcd for $C_{28}H_{41}BO_6Na^+$: 507.2894; found: 507.2901.

4-(4-((2S,3S)-2-(4,4,5,5-tetraethyl-1,3,2-dioxaborolan-2-yl)-3-(trifluoromethyl)bicyclo[2.2.1]heptan-2-yl)benzyl)morpholine (15d)

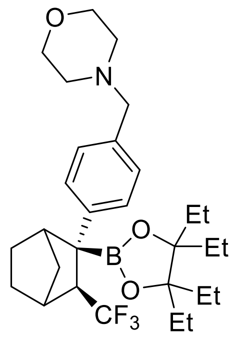

A flame-dried Schlenk flask was charged with 4-(4-bromobenzyl)morpholine (56 mg, 0.22 mmol, 1.0 equiv.) and THF (1 mL) was added. After cooling to -78°C, *n*BuLi (0.22 mmol, 1.1 equiv.) was added dropwise and the mixture was allowed to stir for 30min. 2-(Bicyclo[2.2.1]hept-2-en-2-yl)-4,4,5,5-tetraethyl-1,3,2-dioxaborolane (**13**) (55 mg, 0.2 mmol, 1.0 equiv.) was dissolved in THF (0.2 mL) and added dropwise. Stirring was continued at this temperature for 30min, before the mixture was allowed to warm to 0°C for 45 min. Following this, the solvents were carefully removed *in vacuo*. The residue was dissolved in 2-methyltetrahydrofuran (1.5 mL), dodecane (0.1 mL, 5 Vol%.) was added and the flask was transferred to the photoreactor precooled to -40 °C. Ritter trifluoroiodomethane (0.28 g, 0.8 mmol, 4.0 equiv.) was dissolved in 2-methylTHF (0.5 mL) and added dropwise to the mixture under irradiation at -40°C. The colorless solution was stirred at the indicated temperature under LED irradiation for 12 h. The crude mixture was pushed through a Silica-Plug (4 cm), eluting with Et_2O (50 mL) and was concentrated *in vacuo*. Flash column chromatography (SiO_2 deactivated with TEA; pentane – EtOAc: 9:1; 1% TEA) afforded the title compound as a viscous colorless oil (48 mg, 0.09 mmol, 46 %). ^{1}H NMR of the crude reaction mixture indicated a diastereomeric ratio of >20:1.

^{1}H NMR (400 MHz, DCM-D_2): δ (ppm) = 7.24 – 7.20 (m, 2H), 7.15 – 7.11 (m, 2H), 3.64 (q, J = 4.7, 4.2 Hz, 4H), 3.43 (d, J = 1.7 Hz, 2H), 3.04 (dd, J = 4.2, 2.2 Hz, 1H), 2.39 (q, J = 5.5, 4.9 Hz, 4H), 1.89 (d, J = 10.2 Hz, 1H), 1.81 – 1.70 (m, 1H), 1.58 – 1.49 (m, 5H), 1.45 – 1.36 (m, 5H), 1.26 – 1.20 (m, 2H), 1.12 – 1.03 (m, 1H), 0.95 (t, J = 7.5 Hz, 2H), 0.79 (t, J = 7.6 Hz, 6H), 0.60 (t, J = 7.6 Hz, 6H).

^{13}C NMR (100 MHz, DCM-D_2): δ (ppm) = 144.09, 135.29, 129.49, 128.98, 128.15, 89.06, 67.48, 63.46, 44.35, 39.58, 38.33, 31.00, 26.92, 26.07, 25.40, 24.27, 9.16, 8.32.

^{19}F NMR (400 MHz, DCM-D_2): δ (ppm) = -64.92, -64.95.

HRMS (ESI) m/z: $[Na]^+$ calcd for $C_{29}H_{43}BF_3NO_3Na^+$: 544.3186; found: 544.3182.

(6aR)-6a-hexylhexahydro-2H-cyclopenta[b]furan-2-one (16)

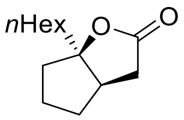

Ethyl 2-((1*R*,2*S*)-2-hexyl-2-(4,4,5,5-tetraethyl-1,3,2-dioxaborolan-2-yl)cyclopentyl)acetate **9b** (47 mg, 0.1 mmol, 1.0 equiv.) was dissolved in THF (3 mL) and cooled to 0°C. NaOH (2 M, 2 mL) was added, followed by dropwise addition of aq. H_2O_2 (30w%., 1 mL). The reaction mixture was allowed to warm to rt. under vigorous stirring. After 7h sat. aq. $Na_2S_2O_3$ (3 mL) was added and the mixture was extracted with Et_2O (3 × 15 mL). The combined organic fractions were washed with Brine, dried over $MgSO_4$ and concentrated *in vacuo*. Flash column chromatography (SiO_2; Pentane – EtOAc: 95:5) afforded the title compound as a colorless oil (19 mg, 0.09 mmol, 89%).

^{1}H NMR (600 MHz, $CDCl_3$): δ (ppm) = 2.85 (dd, J = 18.5, 10.2 Hz, 1H), 2.50 (ddt, J = 10.2, 8.9, 3.1 Hz, 1H), 2.29 (dd, J = 18.5, 3.1 Hz, 1H), 2.07 – 2.02 (m, 1H), 1.93 – 1.86 (m, 1H), 1.76 – 1.64 (m, 4H), 1.62 – 1.57 (m, 1H), 1.56 – 1.50 (m, 1H), 1.42 – 1.35 (m, 2H), 1.34 – 1.24 (m, 6H), 0.88 (t, J = 6.9 Hz, 3H).
^{13}C NMR (150 MHz, $CDCl_3$): δ (ppm) = 177.65, 98.40, 42.22, 39.50, 38.22, 37.22, 34.56, 31.82, 29.65, 24.34, 24.09, 22.70, 14.20.

HRMS (EI-orbitrap): *m/z*: [M] calc. for [$C_{12}H_{22}O_2$]: 210.1620; found: 210.1613.
IR (Diamond-ATR, neat) $\tilde{\nu}_{max}$: 2930 (m), 2860 (w), 1764 (vs), 1456 (w), 1420 (w), 1378 (vw), 1328 (w), 1304 (w), 1270 (w), 1252 (w), 1218 (m), 1188 (s), 1150 (m), 1120 (w), 1104 (w), 1090 (w), 1050 (w), 960 (m), 918 (m), 888 (vw), 846 (vw), 816 (vw), 726 (w), 708 (vw).

(2*R*,3*S*)-1-benzyl-2-butyl-3-(perfluorobutyl)-2-(4,4,5,5-tetraethyl-1,3,2-dioxaborolan-2-yl)azetidine (17a)

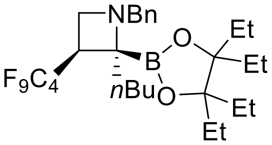

tert-Butyl (2*S*,3*S*)-2-butyl-3-(perfluorobutyl)-2-(4,4,5,5-tetraethyl-1,3,2-dioxaborolan-2-yl)azetidine-1-carboxylate **3a** (61 mg, 0.1 mmol, 1.0 equiv.) was dissolved in DCM (1 mL) and treated with TFA (0.5 mL). After stirring at rt. for 4h, the solvents were removed *in vacuo* and the residue was transferred in a pressure tube containing BnBr (36 µL, 0.3 mmol, 3.0 equiv.), K_2CO_3 (41 mg, 0.3 mmol, 3.0 equiv.) in MeOH (3 mL). The reaction mixture was then heated to 70°C overnight. After removing the solvents *in vacuo*, water (5 mL) was added and the aqueous phase was extracted with EtOAc (3 × 15 mL). The combined organic fractions were washed with Brine, dried over $MgSO_4$ and concentrated *in vacuo*. Flash column chromatography (SiO_2; pentane – EtOAc; 99:1) afforded the title compound as a colorless oil (40 mg, 0.067 mmol, 67%). ^{19}F NMR analysis of the crude reaction mixture indicated a diastereomeric ratio of 8:1.

^{1}H NMR (600 MHz, Acetone-D$_6$): δ (ppm) = 7.36 – 7.27 (m, 4H), 7.24 – 7.19 (m, 1H), 4.00 – 3.79 (m, 2H), 3.34 – 3.09 (m, 2H), 3.11 – 2.96 (m, 1H), 1.84 – 1.75 (m, 8H), 1.54 – 1.44 (m, 1H), 1.40 – 1.27 (m, 3H), 0.97 (td, J = 7.5, 1.8 Hz, 12H), 0.93 – 0.86 (m, 5H).

^{13}C NMR (150 MHz, Acetone-D$_6$): δ (ppm) = 140.58, 129.20, 128.99, 127.62, 90.10, 60.17, 51.26, 42.64, 42.28, 42.05, 27.75, 27.08, 26.37, 24.11, 14.33, 14.20, 9.10, 9.06.

^{19}F NMR (400 MHz, Acetone-D$_6$): δ (ppm) = -81.92, -81.93, -81.94, -81.95, -81.96, -81.98, -81.99, -113.46, -113.49, -113.58, -113.62, -123.45, -124.23, -124.77, -125.54, -126.76, -126.79, -126.83.

HRMS (ESI) m/z: [H]$^+$ calcd for $C_{28}H_{40}BF_9NO_2^+$: 604.3003; found: 604.3021.

IR (Diamond-ATR, neat) $\tilde{\nu}_{max}$: 2936 (w), 2886 (w), 2860 (w), 1494 (vw), 1458 (w), 1386 (w), 1352 (m), 1306 (w), 1288 (w), 1232 (vs), 1168 (m), 1134 (vs), 1112 (s), 1074 (m), 1026 (m), 968 (w), 922 (m), 888 (w), 854 (w), 834 (w), 800 (w), 778 (w), 742 (m), 728 (m).

Ethyl 2-((1*R*,2*S*)-2-(trifluoro-l4-boraneyl)-2-((trimethylsilyl)methyl)cyclopentyl)acetate, potassium salt (17b)

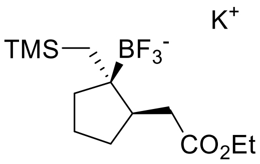

A flame-dried flask was charged with ethyl 2-((1*R*,2*S*)-2-(4,4,5,5-tetraethyl-1,3,2-dioxaborolan-2-yl)-2-((trimethylsilyl)methyl)cyclopentyl)acetate (**9c**) (42 mg, 0.1 mmol, 1.0 equiv.), dry DCM (3 mL) was added and the mixture was cooled to -78°C. A solution of BCl_3 in Heptane (1 mmol, 10 equiv.) was added dropwise to the mixture and stirring was continued at the aforementioned temperature for 30 min. Next, the dry-ice bath was removed and the red solution was allowed to warm to rt, stirring at this temperature for a further 4 h. Dry MeOH (2 mL) was added dropwise at 0°C and the mixture was allowed to warm to rt. for 1 h. After that, the solvents were removed *in vacuo* and the residue was dissolved in MeOH (1 mL). Saturated aqueous KHF_2 (0.42 mmol, 4.2 equiv.) was added dropwise and the mixture was allowed to stir overnight at ambient temperature. After addition of MeOH (2 mL) the solvents were removed *in vacuo* and the residual solid was washed and sonicated with pentane (3 × 2 mL) to obtain the title compound as an off-white solid (30 mg, 0.09 mmol, 85 %).

^{1}H NMR (400 MHz, CD_3OD): δ (ppm) = 3.61 (s, 2H), 2.61 (d, J = 14.7 Hz, 1H), 2.28 (t, J = 12.9 Hz, 1H), 1.97 – 1.35 (m, 7H), 1.18 – 0.78 (m, 3H), 0.33 – 0.13 (m, 2H), -0.02 (s, 9H).

^{13}C NMR (100 MHz, CD_3OD): δ (ppm) = 178.70, 51.84, 51.60, 38.70, 38.17, 32.51, 29.08, 24.08, 1.32.

^{11}B NMR (128 MHz, $CDCl_3$): δ (ppm) = 5.55.

^{19}F NMR (400 MHz, CD_3OD): δ (ppm) = -142.96, -154.89, -155.65, -155.74, -156.38.

HRMS (ESI) m/z: $[H]^+$ calcd for $C_{12}H_{23}BF_3O_2Si^+$: 295.1517; found: 295.1516.

(4a*R*,7a*S*)-7a-Butyl-1-hydroxyhexahydrocyclopenta[c][1,2]oxaborinin-3(1H)-one (17c)

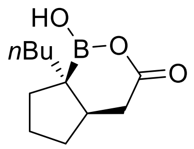

According to a modified literature procedure, a flame-dried flask was charged with *tert*-Butyl 2-((1*R*,2*S*)-2-butyl-2-(4,4,5,5-tetraethyl-1,3,2-dioxaborolan-2-yl)cyclopentyl)acetate (**9a'**) (85 mg, 0.2 mmol, 1.0 equiv.) and dry DCM (5 mL). The mixture was cooled to -78°C and a solution of BCl_3 in Heptane (2 mmol, 10 equiv.) was added dropwise. After 30 min, the yellow solution was allowed to warm to rt. and stirred at this temperature overnight. The next day, the solvents were removed *in vacuo* and the residue was dissolved in Et_2O (10 mL). After washing with water (10 mL), the aqueous phase was extracted with Et_2O (2 × 10 mL) and the combined organic fractions were washed with Brine, dried over anhydr. $MgSO_4$ and concentrated *in vacuo.* The residual solid was carefully washed with ice-cold pentane (10 mL) to afford the title compound as a colorless solid (18 mg, 0.086 mmol, 43 %)

^{1}H NMR (400 MHz, $CDCl_3$): δ (ppm) = 3.19 (dd, J = 14.2, 8.1 Hz, 1H), 2.32 (d, J = 14.2 Hz, 1H), 2.06 – 1.92 (m, 2H), 1.68 – 1.43 (m, 5H), 1.36 – 1.05 (m, 5H), 0.92 – 0.82 (m, 3H).

^{13}C NMR (150 MHz, $CDCl_3$): δ (ppm) = 186.82, 43.14, 40.18, 37.67, 35.48, 32.55, 29.05, 25.26, 24.11, 14.52.

^{11}B NMR (128 MHz, $CDCl_3$): δ (ppm) = 34.46.

HRMS (ESI) m/z: $[H]^+$ calcd for $C_{11}H_{18}BO_3^+$: 209.1349; found: 209.1354.

(2*R*,3*S*)-1-Benzyl-2-isobutyl-3-(perfluorobutyl)-2-(4,4,5,5-tetraethyl-1,3,2-dioxaborolan-2-yl)azetidine (17b)

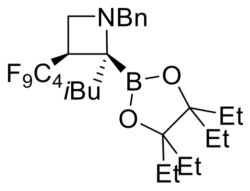

The *N*-Boc protected azetidine (1.0 g, 1.7 mmol, 1.0 equiv.) was charged to a flame-dried flask and dissolved in dry DCM (8 mL). TFA (1.5 mL, 20 mmol, 12 equiv.) was added dropwise and the reaction mixture was allowed to stir for 3 h at ambient temperature. After that, the volatiles were removed *in vacuo*, and the dark red mixture was dissolved in EtOAc (50 mL) and washed with sat. aq. Na_2CO_3 (2 × 30 mL) and Brine (30 mL). The solvent was removed *in vacuo*, and the deprotected azetidine was transferred to an Ace pressure vial containing benzyl bromide (0.5 mL, 4 mmol, 2.3 equiv.), DIPEA (1.7 mL, 10 mmol, 6 equiv.) and MeCN (3 mL). The vial was closed and the mixture was allowed to stir at 100 °C overnight. The next day, all volatiles were removed *in vacuo*, and the crude product was purified by flash column chromatography ((SiO_2; pentane – EtOAc; 99:1 to 96:4) to obtain the title compound as a yellow oil (0.58 g, 1 mmol, 56 %).

^{1}H NMR (500 MHz, $CDCl_3$): δ (ppm) = 7.34 – 7.28 (m, 4H), 7.24 – 7.20 (m, 1H), 4.01 (d, J = 13.2 Hz, 1H), 3.77 (d, J = 13.2 Hz, 1H), 3.23 (t, J = 7.1 Hz, 1H), 3.11 (dd, J = 10.0, 6.2 Hz, 1H), 3.04 – 2.90 (m, 1H), 1.90 – 1.62 (m, 11H), 1.02 (d, J = 6.6 Hz, 3H), 0.99 – 0.91 (m, 15H).

^{13}C NMR (126 MHz, $CDCl_3$): δ (ppm) = 139.65, 128.65, 128.43, 126.97, 89.40, 59.23, 50.80, 42.68, 42.50, 42.32, 25.99, 25.49, 25.19, 24.86, 24.76, 9.09, 8.63.

^{19}F NMR (471 MHz, $CDCl_3$) δ (ppm) = -81.07, -81.08, -81.09, -81.10, -81.10, -81.12, -81.12, -113.44, -113.48, -113.51, -123.14, -123.77, -124.43, -125.06, -126.14, -126.17, -126.20.

HRMS (ESI) m/z: $[H]^+$ calcd for $C_{28}H_{40}BF_9NO_2^+$: 604.3010; found: 604.3013.

Ethyl 2-((2*R*,3*S*)-1-benzyl-2-isobutyl-2-(4,4,5,5-tetraethyl-1,3,2-dioxaborolan-2-yl)azetidin-3-yl)acetate (18)

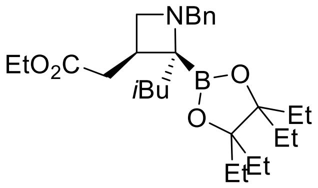

Compound **3p** (0.5 g, 1 mmol, 1.0 equiv.) was charged to a flame-dried flask and dissolved in dry DCM (6 mL). TFA (1.7 mL, 20 mmol, 20 equiv.) was added dropwise and the reaction mixture was allowed to stir for 3 h at ambient temperature. After that, the volatiles were removed *in vacuo*, and the dark red mixture was dissolved in EtOAc (50 mL) and washed with sat. aq. Na_2CO_3 (2 × 30 mL) and Brine (30 mL). The solvent was removed *in vacuo*, and the deprotected azetidine was transferred to an Ace pressure vial containing benzyl bromide (0.2 mL, 1.5 mmol, 1.5 equiv.), DIPEA (0.96 mL, 1.5 mmol, 1.5 equiv.) and MeCN (1 mL). The vial was closed and the mixture was allowed to stir at 100 °C overnight. The next day, all volatiles were removed *in vacuo*, and the crude product was purified by flash column chromatography ((SiO_2; pentane – EtOAc; 8:2 to 7:3) to obtain the title compound as a colorless oil (0.2 g, 0.4 mmol, 38 %).

^{1}H NMR (400 MHz, $CDCl_3$): δ (ppm) = 7.33 – 7.27 (m, 5H), 4.58 (d, *J* = 14.8 Hz, 1H), 4.47 (d, *J* = 14.9 Hz, 1H), 4.11 – 4.04 (m, 2H), 3.26 (dd, *J* = 11.7, 5.2 Hz, 1H), 2.87 (t, *J* = 10.8 Hz, 1H), 2.54 – 2.48 (m, 1H), 2.42 (dq, *J* = 9.0, 5.6 Hz, 1H), 2.23 (dd, *J* = 16.2, 10.4 Hz, 1H), 1.92 (dt, *J* = 13.3, 6.7 Hz, 1H), 1.66 – 1.59 (m, 10H), 1.19 – 1.16 (m, 3H), 1.00 (d, *J* = 6.6 Hz, 3H), 0.95 (d, *J* = 6.7 Hz, 3H), 0.88 (t, *J* = 7.4 Hz, 12H).
^{13}C NMR (150 MHz, $CDCl_3$): δ (ppm) = 171.75, 154.23, 137.02, 128.64, 128.38, 127.58, 90.34, 60.90, 52.68, 48.55, 44.18, 36.25, 34.06, 26.02, 25.89, 24.84, 24.54, 24.27, 14.24, 8.81, 8.71.

HRMS (ESI) m/z: $[H]^+$ calcd for $C_{28}H_{47}BNO_4^+$: 472.3600; found: 472.3606.

(1*R*,5*R*)-7-Benzyl-1-isobutyl-2-oxa-7-azabicyclo[3.2.0]heptan-3-one (20)

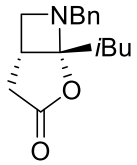

Compound **18** (24 mg, 0.05 mmol, 1.0 equiv.) was charged to a flame-dried flask and dissolved in dry DCM (1 mL) and cooled to -78°C. A solution of BCl_3 in heptane (0.5 mmol, 10 equiv.) was added dropwise, and the reaction mixture was allowed to stir at the aforementioned temperature for 1 h. After that, the pale yellow solution was allowed to warm to rt., stirring at this temperature for 1 h. Dry MeOH (0.5 mL) was added dropwise and stirring was continued for 30 min. Following this, the solvents were removed by high-vacuum, and residue was dissolved in THF (1 mL). A 1:1 mixture of 2 M NaOH (0.5 mL) and H_2O_2 (30 %, 0.5 mL) was added dropwise to the mixture at 0°C, and the reaction was allowed to warm to rt. After 1 h stirring at rt. sat. aq. NH_4Cl (2 mL) was added. H_2O (3 mL) was added and the aq. fraction was extracted with EtOAc (3 × 10 mL). The combined organic fractions were washed with Brine (5 mL), dried over anhydr. $MgSO_4$ and the solvents were removed *in vacuo.* The crude compound was purified by flash column chromatograophy (SiO_2, pentane – EtOAc: 9:1 to 3:7) to obtain the title compound as a colorless oil (11 mg, 0.04 mmol, 84 %)

^{1}H NMR (500 MHz, $CDCl_3$): δ (ppm) = 7.37 – 7.27 (m, 3H), 7.25 – 7.20 (m, 2H), 4.52 (d, *J* = 14.7 Hz, 1H), 4.38 (d, *J* = 14.7 Hz, 1H), 3.45 (dd, *J* = 9.7, 6.4 Hz, 1H), 3.36 (t, *J* = 9.1 Hz, 1H), 3.26 (q, *J* = 8.0 Hz, 1H), 2.66 (d, *J* = 8.5 Hz, 2H), 2.31 (d, *J* = 2.2 Hz, 1H), 2.29 (d, *J* = 1.2 Hz, 1H), 2.14 (dt, *J* = 13.4, 6.7 Hz, 1H), 0.88 (dd, *J* = 6.6, 3.9 Hz, 6H).

^{13}C NMR (126 MHz, $CDCl_3$): δ (ppm) = 208.07, 172.33, 136.10, 128.92, 128.31, 127.89, 50.60, 47.41, 46.75, 43.00, 33.42, 24.36, 22.64.

HRMS (ESI) m/z: $[H]^+$ calcd for $C_{16}H_{22}NO_2^+$: 260.1652; found: 260.1644.

(2*R*,3*S*)-1-Benzyl-2-isobutyl-3-(perfluorobutyl)-2-(trifluoro-l4-boraneyl)azetidine, potassium salt (19)

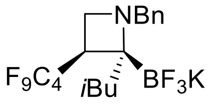

Compound **17b** (60 mg, 0.1 mmol, 1.0 equiv.) was charged to a flame-dried flask and dissolved in dry DCM (1 mL) and cooled to -78°C. A solution of BCl_3 in heptane (1 mmol, 10 equiv.) was added dropwise, and the reaction mixture was allowed to stir at the aforementioned temperature for 1 h. After that, the red solution was allowed to warm to rt., stirring at this temperature for 2 h. Dry MeOH (0.5 mL) was added dropwise and stirring was continued for 30 min. Following this, the solvents were removed by high-vacuum, and residue was dissolved in MeOH (1 mL). A 4.2 M aq. solution of KHF_2 (0.4 mmol, 4 equiv.) was added, and the mixture was allowed to stir at rt. for 30 min. The solvents were removed *in vacuo*, and dry acetone (2 mL) was added. After sonication, the acetone was carefully decanted and removed *in vacuo*. The solid residue was dissolved in MeOH (0.1 mL) and dropped in hexane. The colorless solid was collected by filtration to obtain the title compound as a colorless amorphous solid (40 mg, 0.08 mmol, 75 %).

^{1}H NMR (500 MHz, CD_3OD): δ (ppm) = 7.45 (s, 5H), 4.48 – 4.39 (m, 2H), 4.14 (ddd, *J* = 10.9, 8.8, 1.5 Hz, 1H), 3.56 (t, *J* = 8.7 Hz, 1H), 3.23 – 3.11 (m, 1H), 2.12 (dt, *J* = 12.6, 6.3 Hz, 1H), 1.99 (dd, *J* = 13.9, 5.7 Hz, 1H), 1.75 (dd, *J* = 13.9, 5.3 Hz, 1H), 1.02 (d, *J* = 6.6 Hz, 3H), 0.95 (d, *J* = 6.7 Hz, 3H).
^{13}C NMR (126 MHz, CD_3OD): δ (ppm) = 132.18, 131.50, 130.78, 130.39, 56.11, 49.77, 42.38, 42.17, 41.97, 25.04, 25.02, 24.96, 24.70.
^{19}F NMR (471 MHz, CD_3OD): δ (ppm) = -82.63, -82.63, -82.65, -82.66, -82.66, -82.68, -82.68, -114.23, -114.25, -114.26, -114.27, -114.28, -114.29, -114.30, -114.33, -114.83, -114.85, -114.86, -114.87, -114.88, -114.89, -114.90, -114.91, -114.93, -119.82, -119.88, -120.46, -123.60, -123.62, -123.64, -123.65, -123.67, -123.69, -123.71, -123.73, -124.23, -124.25, -124.27, -124.29, -124.30, -124.32, -124.34, -124.34, -124.36, -125.35, -125.36, -125.38, -125.40, -125.42, -125.44, -125.46, -125.98, -126.00, -126.01, -126.03, -126.05, -126.07, -126.09, -126.45, -126.47, -126.49, -126.51, -126.53, -127.08, -127.09, -127.11, -127.13, -127.14, -127.15, -127.25, -127.26, -127.27, -127.28, -127.30, -127.31, -127.32, -127.33, -127.35, -127.36, -127.37, -127.39, -127.87, -127.89, -127.90, -127.91, -127.92, -127.93, -127.95, -141.16.

HRMS (ESI) m/z: [H]$^+$ calcd for $C_{18}H_{19}BF_{12}N^+$: 489.1558; found: 489.1563.

6. NMR-spectroscopic determination of the relative configuration

General strategy and nomenclature

The determination of the respective relative configurations was done using a combination of the (^{1}H-^{1}H) nuclear Overhauser effect (NOE) and the (^{1}H-^{19}F) heteronuclear Overhauser effect (HOE). The structures of the compounds **3d** and **3e** investigated in detail are shown in figure SF-3.

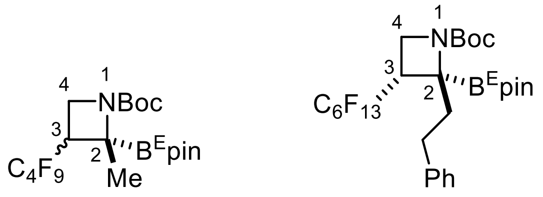

3e (74%)
(dr = 1:1)

3d (74%)
(dr > 20:1)

Supplementary Figure 3: Structures of the investigated compounds **3e** and **3d** including the numbering used for the combined NOE/HOE analysis. For both compounds the anti (referring to the relation of the perfluorinated and the alkylic side chain) configuration is depicted for consistency with the main text (despite the fact that the determination of the configuration is the subject of this chapter).

Compound **3e** is formed in a diastereomeric ratio of ca. 1:1, which allows the investigation and comparison of the relative configuration of both diastereomers **3e**$_{\mathbf{syn}}$ and **3e**$_{\mathbf{anti}}$, with *syn* meaning that the methyl group at position 2 and the perfluorinated side chain at position 3 are located on the same side of the four-membered heterocycle, whereas *anti* refers to both being on opposite sides. For the *anti* diastereoisomer we would expect to see a strong ^{1}H,^{1}H-NOE between the methyl group at position 2 and proton 3 accompanied by a weak ^{1}H,^{19}F-HOE between the methyl group and the perfluorinated side chain at position 3. For the *syn* diastereomer we would expect to see the opposite behavior of a weak NOE and a strong HOE (see scheme 6 in the main text). Compound **3d** was synthesized with such a high diastereomeric purity (dr = 20:1) that no minor diastereomer could be observed *via* NMR spectroscopy. For the assignment we use the fact that (only) one of the patterns described above can be observed for the *major* (sole) diastereomer, allowing to unambiguously assign its relative configuration to be *anti*. It should be noted that both compounds exhibit two signal sets as they exist as ca. 1:1 mixtures of Boc-rotamers in slow chemical exchange. This can be seen in the 2D NOESYs (figures SF-6 (**3e**) and SF-12 (**3d**)), where exchange peaks of the same sign as the diagonal peaks can be observed close to the diagonal. In figure SF-11 the

appearance of chemical exchange in NOE spectra is explained in more detail, using one-dimensional spectra.

Experimental details

NMR spectra were recorded on a 400 MHz (^{1}H resonance frequency, Bruker AVANCE III HD, BBFO probe) and a 700 MHz (^{1}H resonance frequency, Bruker AVANCE III HD, QCI cryo ^{1}H/^{19}F-^{31}P/^{13}C/^{15}N-^{2}H probe) spectrometer. Acquisition and analysis of all spectra was accomplished using the software TopSpin (versions 3.6.2 and 3.5.7) from Bruker. The samples were prepared in standard 5 mm NMR tubes using acetone-d_6 (Sigma Aldrich ampoule, 99.9 atom % D) as solvent. Compounds **3e** (8.32 mg) and **3d** (8.25 mg) were each weighed into an NMR tube directly, acetone-d_6 was added and the tubes were flame-sealed under atmosphere resulting in samples with a concentration of 33.7 µmol/L for **3e** (377.39 mg acetone-d_6) and 28.8 µmol/L for **3d** (327.59 mg acetone-d_6). All measurements were carried out at 300 K. For assignment of resonances ^{1}H, {^{1}H}^{13}C, ^{19}F, ^{1}H,^{13}C-HSQC, ^{1}H,^{13}C-HMBC, ^{1}H,^{1}H-COSY, ^{1}H-^{1}H-TOCSY and ^{1}H,^{1}H-NOESY spectra were acquired at 700 MHz using standard Bruker pulse sequences.

1D selective ^{1}H,^{1}H-NOE spectra were recorded at 700 MHz using a standard Bruker pulse sequence (*selnogpzs.2*[12,13]). Spectra were acquired with 64k points using 8 dummy scans (DS) and 32 or 64 scans (NS), zero-filled to 128k points and processed using an exponential apodization with a line broadening factor of 2 Hz. For the selective refocusing *RSnob* shaped pulses (for durations and bandwidths see table SF-6) were used which were calibrated according to the Bruker *Shapetool*.

Supplementary Table 6: Durations, offsets and bandwidths of selective RSnob pulses used for this 1D NOE analysis.

compound	proton	diastereomer		rotamer			offset / ppm	duration / ms	bandwidth / Hz
		anti	syn	1	2	1+2			
**3d**	H3	x		x			3.47	58	40
**3d**	H3	x			x		3.58	58	40
**3d**	H3	x				x	3.53	19	120
**3e**	H3		x			x	3.41	39	60
**3e**	H3	x				x	3.25	31	75

The sufficient selectivity of the shaped pulses was checked by acquisition of 1D selective spin echo spectra (Bruker pulse sequence *selgpse*) before the NOE measurements. NOE mixing time series with mixing times (D8) of 50, 100, 150, 200, 250, 300, 350, 400 and 500 ms were acquired. To ensure quantifiability of all spectra the relaxation delay D1 was set to at least five

times the longest T_1 (D1 = 23 s). Longitudinal relaxation times were determined *via* the inversion recovery method (Bruker pulse sequence *t1ir*).

2D ^{1}H,^{1}H-NOESY spectra (Bruker pulse sequence *noesygpphzs*[13,14]) were recorded at 400 MHz with 2k points (DS = 32, NS = 4 or 8, D1 = 2 s) in the direct dimension F2, spectral widths (SW) of 5 ppm (**3e**) and 8 ppm (**3d**) and offsets of 2.5 ppm (**3e**) and 4.2 ppm (**3d**). In the indirect dimension F1 256 (**3e**) or 512 (**3d**) points were acquired. The raw data was processed to 4k points in F2 and 1k (**3e**) or 2k (**3d**) points in F1 using a sine-bell apodization (SSB = 2) in both dimensions. The mixing time D8 was set to 400 (**3e**) or 500 (**3d**) ms.

2D ^{1}H,^{19}F-HOESY spectra (Bruker pulse sequence *hoesygpph*[15]) were recorded at 400 MHz with 2k points (DS = 32, NS = 32, D1 = 2 s) in the direct proton dimension F2, spectral widths (SW2) of 5 ppm (offset (O2P) 2.5 ppm, **3e**) and 8 ppm (offset (O2P) 4.2 ppm, **3d**). In the indirect fluorine dimension F1 128 points with spectral widths (SW1) of 20 ppm and an offset (O1P) of -121 ppm were acquired. The raw data was processed to 4k points in F2 and 1k points in F1 using a sine-bell apodization (SSB = 2) in both dimensions. The mixing time D8 was set to 500 ms.

Investigations of compound 3e

The ^{1}H NMR spectrum of **3e** shows baseline separated signals for the protons 3_{syn} and 3_{anti}, allowing for selective refocusing of the resonances and therefore a straightforward NOE analysis via 1D NOE spectra. Every diastereomer is represented by two signal sets due to the mixture of Boc rotamers. Therefore, both rotamer signals of one diastereomer are refocused simultaneously by one selective pulse (see table SF-6). An assignment of all ^{1}H resonances relevant to this NOE analysis is given in figure SF-4.

^{1}H chemical shifts (ppm)<br>700 MHz,<br>300K, acetone-d_6	diastereomer 1<br>**3e$_{anti}$**	diastereomer 2<br>**3e$_{syn}$**
rotamer 1	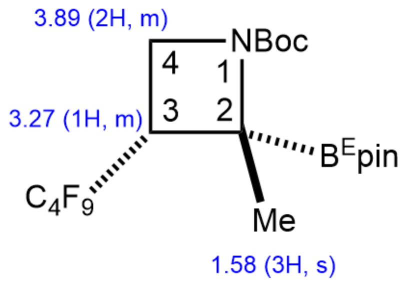	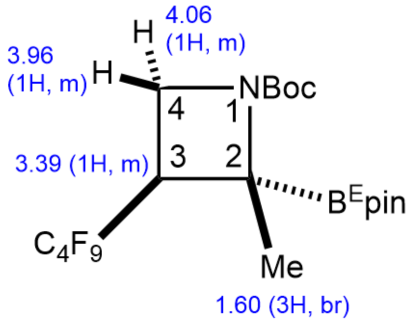
rotamer 2	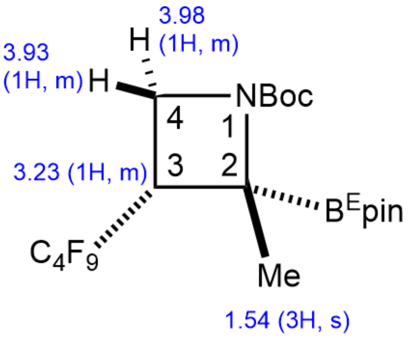	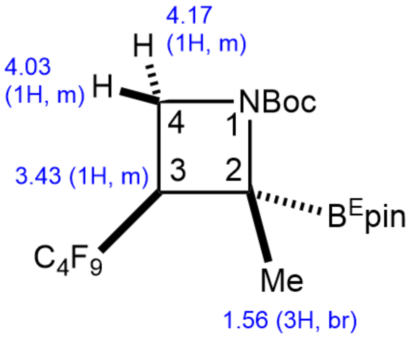

Supplementary Figure 4: Assignment of ^{1}H resonances to the different diastereomers and rotamers. Only the chemical shifts (blue) relevant to this analysis are visualized.

Interestingly, already in the normal ^{1}H NMR spectra (see figure SF-5) the methyl groups of the two diastereomers show a completely different signal shape with the *anti* methyl groups being sharp singlets and the *syn* methyl groups being substantially more broadened, indicating significantly different rotamer inversion barriers. This is also seen to a lesser extent in the respective signals of H3.

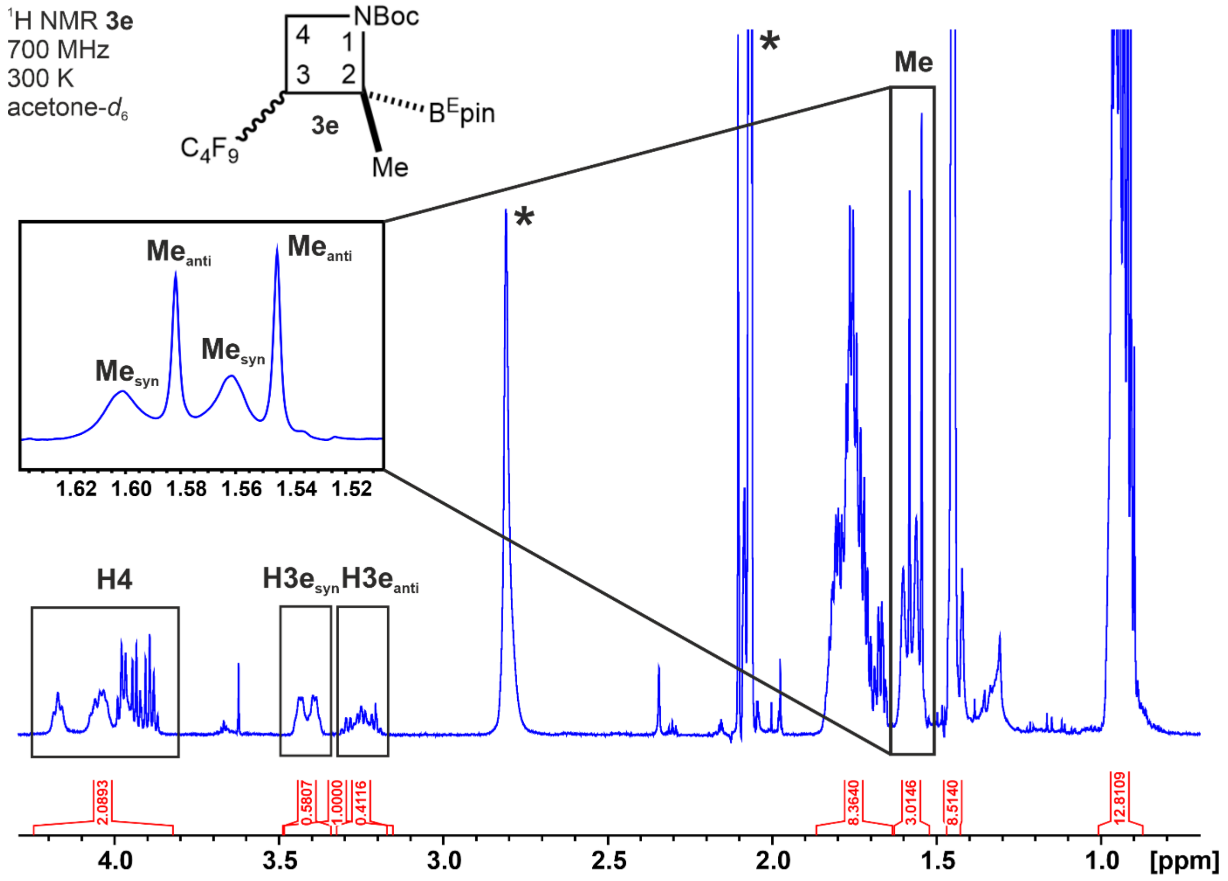

Supplementary Figure 5: ^{1}H NMR spectrum (relevant part shown) of compound **3e** in acetone-d_6 at 700 MHz and 300 K. Water and acetone are marked with an asterisk.

A 2D NOESY spectrum (figure SF-6) was acquired to gain insights into the stereochemical relation of the protons $H3_{syn}$/$H3_{anti}$ and the methyl groups.

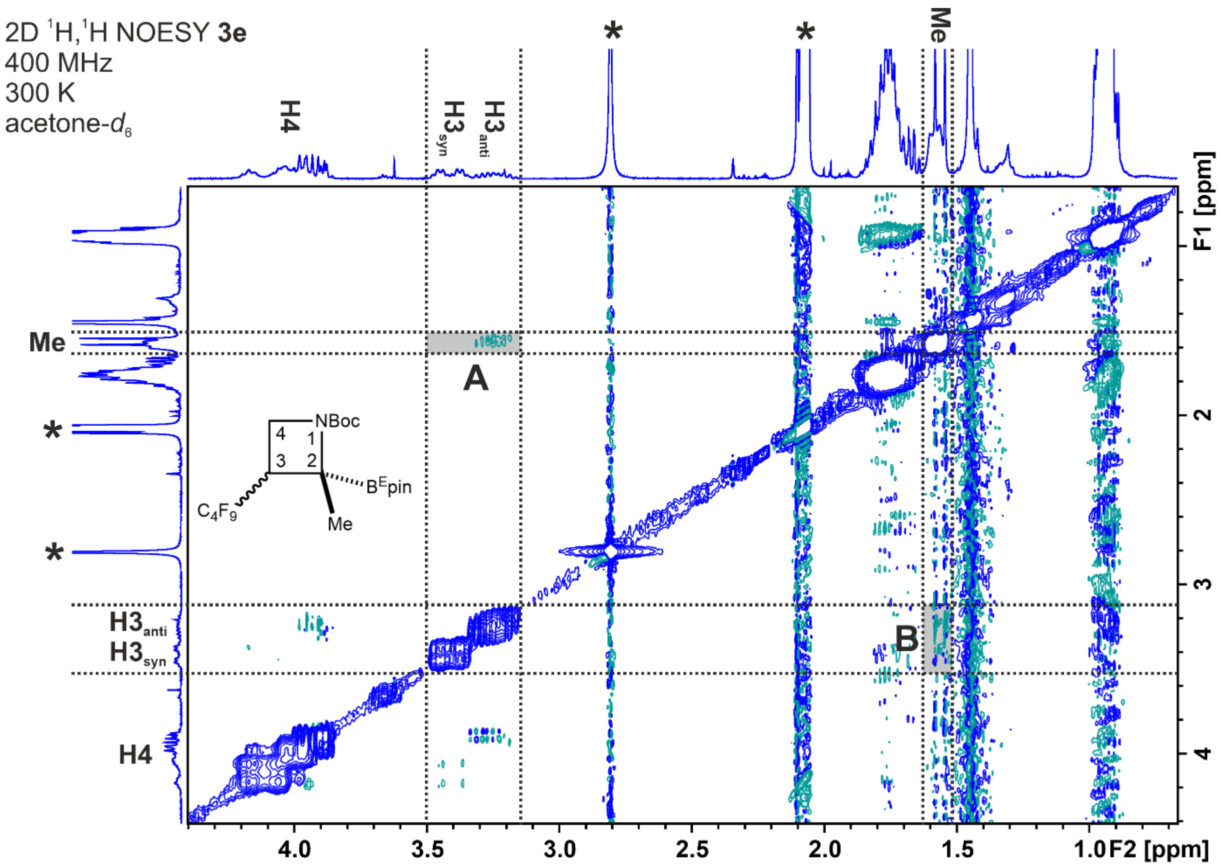

Supplementary Figure 6: 2D NOESY of compound **3e** in acetone-d_6 at 400 MHz and 300 K. The regions A and B where NOE cross peaks between $H3_{anti}$ and the methyl groups are observed (and not observed for $H3_{syn}$ and the methyl groups) are highlighted with grey boxes. Water and acetone are marked with asterisks.

The 2D NOESY in figure SF-6 shows NOE cross peaks between $H3_{anti}$ and the methyl groups and no NOE cross peak between $H3_{syn}$ and the methyl groups. The four methyl groups are poorly separated in F1 because of their similar chemical shifts and the limited resolution in the indirect dimension (see region A in figure SF-6). Additionally, spectrometer instability (also known as t1 noise), reduces spectral quality in region B drastically. Because of these findings, 1D NOE spectra (figure SF-7) were acquired, avoiding both drawbacks mentioned. These were recorded by selectively refocusing $H3_{syn}$ (red spectrum, in the middle) and $H3_{anti}$ (black spectrum, at the bottom) respectively. The ^{1}H NMR spectrum (blue, at the top) is also shown in figure SF-7 for easier understanding.

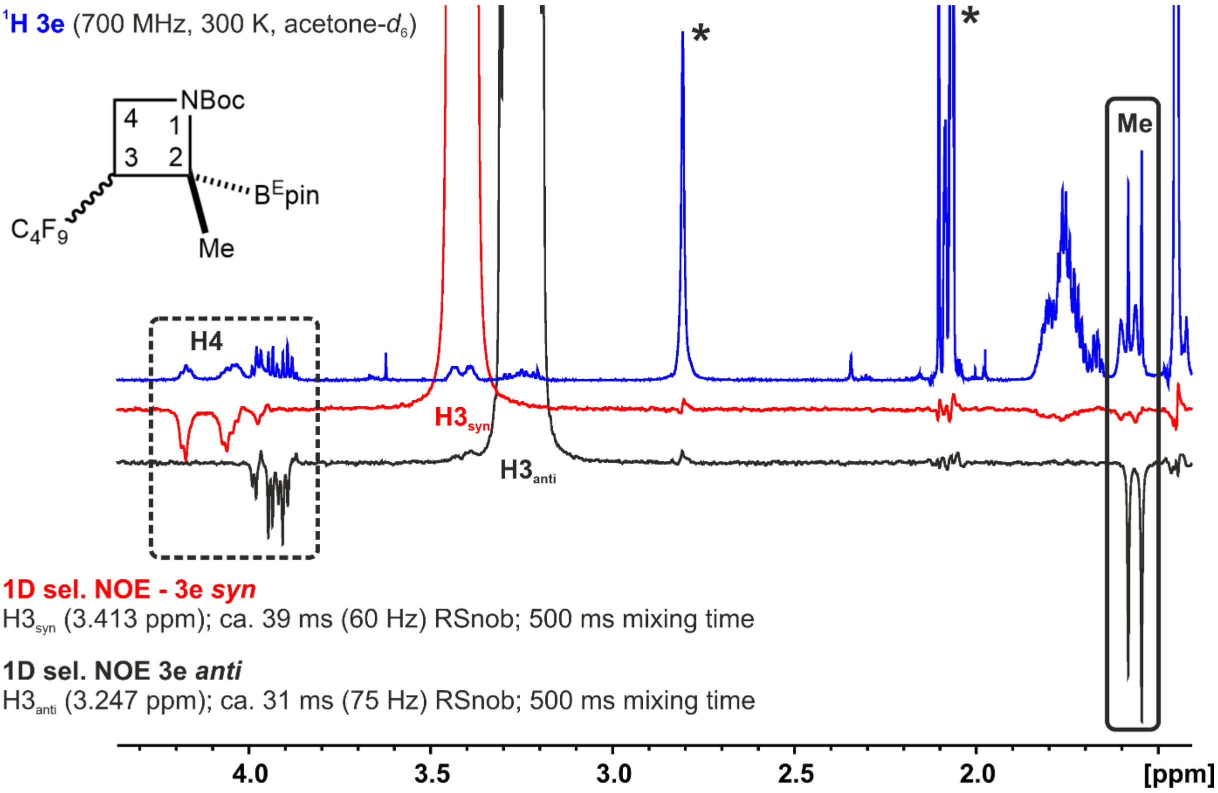

Supplementary Figure 7: Selective 1D NOE spectra and ^{1}H spectrum (blue, spectrum at the top) of compound **3e** in acetone-d_6 at 700 MHz and 300 K. H3_{syn} (red, spectrum in the middle) and H3_{anti} (black, spectrum at the bottom) were selectively refocused using RSnob pulses (see also table SF-6) of ca. 39 ms (60 Hz bandwidth, H3_{syn}) and ca. 31 ms (75 Hz bandwidth, H3_{anti}). The mixing time was set to 500 ms in both NOE experiments. Water and acetone are marked with asterisks.

For the *anti* diastereomer (black spectrum at the bottom) an intense NOE signal between H3_{anti} and the sharp methyl groups Me_{anti} can be observed (black box with solid lines), whereas only a very weak NOE signal between H3_{syn} and the broad methyl groups Me_{syn} can be observed. This indicates that in the *anti* diastereomer H3_{anti} and the methyl group are on the same side of the heterocycle. Therefore, the methyl group and the perfluorinated side chain have to be on opposite sides (and *vice versa* for the *syn* diastereomer). As expected, for both the *syn* and the *anti* diastereomer intense NOEs can be observed between H3 and the neighboring protons H4 (black box with dotted line), which allows assignment of the diastereotopic protons. To further support this interpretation a 2D ^{1}H,^{19}F-HOESY spectrum (figure SF-8) was acquired to determine the stereochemical relation of the perfluorinated side chain with respect to the methyl group.

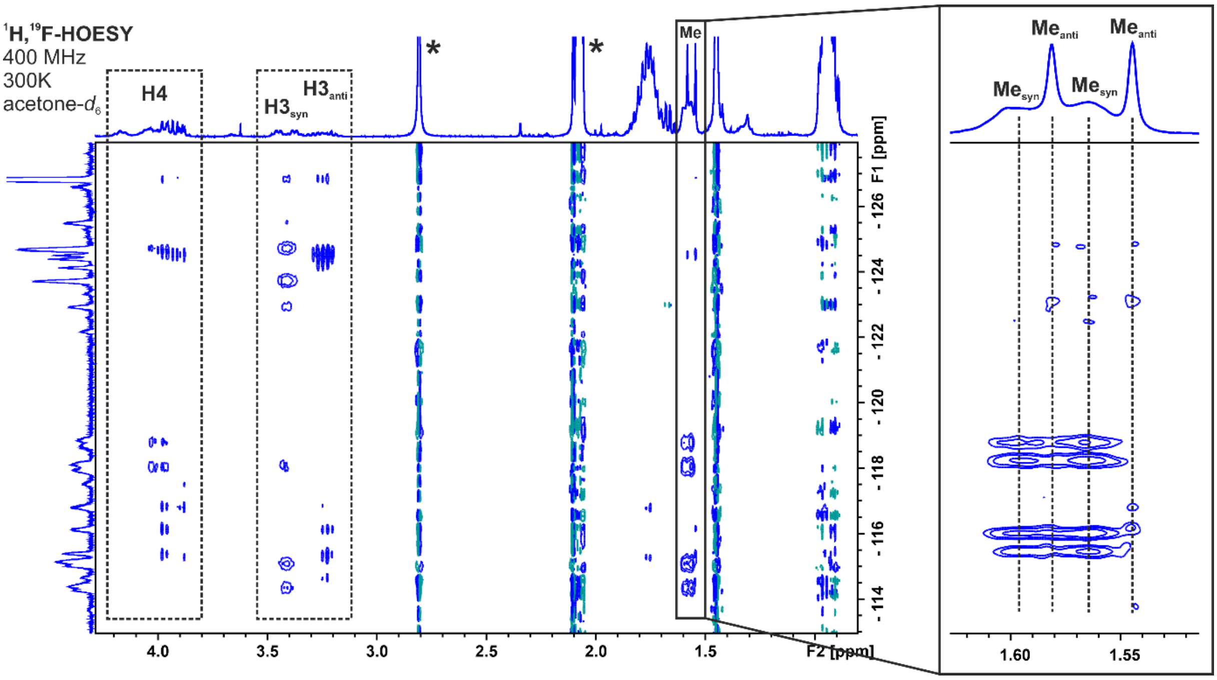

Supplementary Figure 8: 2D ^{1}H,^{19}F HOESY of compound **3e** in acetone-d_6 at 400 MHz and 300 K. The region where HOEs between the methyl groups and the perfluorinated side chains can be observed is highlighted (black box with solid line) and shown in more detail.

Intense HOEs are observed between the broad methyl groups Me_{syn} and the perfluorinated side chain, whereas no significant HOEs are observed between the sharp methyl groups Me_{anti} and the perfluorinated side chain (black box with solid lines and detailed view). Both findings match the expectations and results of the NOE analysis, allowing for an unambiguous assignment of the relative configuration by a combined analysis of NOESY and HOESY spectra. Additionally, the HOESY shows intense HOEs between the perfluorinated side chain and the protons H4 of both diastereomers, $H3_{anti}$ and $H3_{syn}$ (black boxes with dashed lines), which matches the expectation because of their close proximities in both diastereomers.

Investigations of compound 3d

No minor component is observed via ^{1}H NMR spectroscopy (figure SF-10) because of the excellent diastereoselectivity of the reaction. As for compound **3e**, two signal sets are visible because of the two Boc rotamers in slow exchange with each other. An example of the chemical exchange is demonstrated in figure SF-11 using selective 1D sel. NOE and 1D selective spin echo spectra. An assignment of all ^{1}H resonances that are relevant for the NOE analysis is shown in figure SF-9.

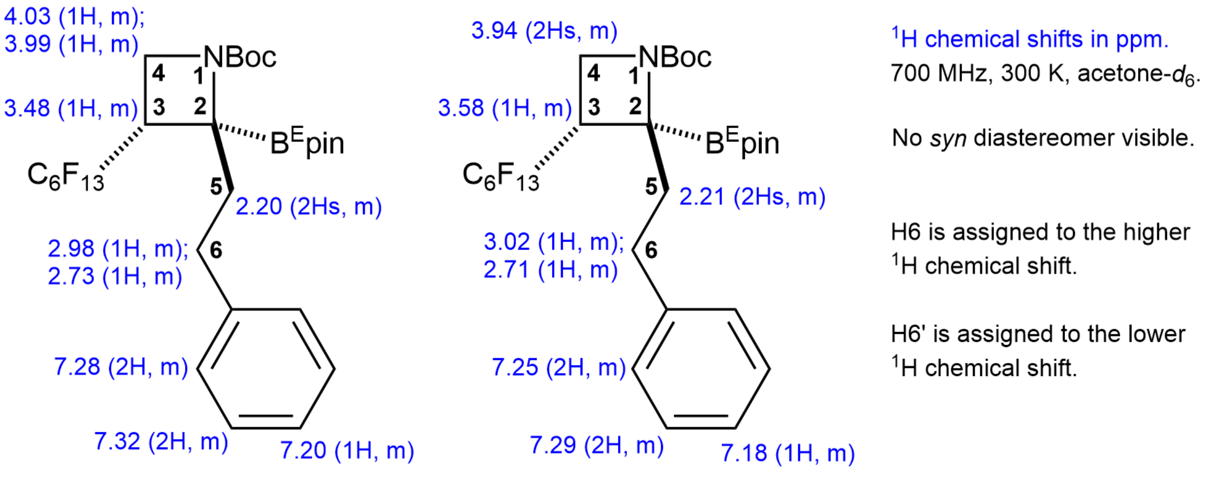

$3d_{anti}$ (rotamer 1) **$3d_{anti}$** (rotamer 2)

Supplementary Figure 9: Assignment of ^{1}H chemical shifts (acetone-d_6, 700 MHz, 300 K) to the two Boc rotamers present in compound **$3d_{anti}$**.

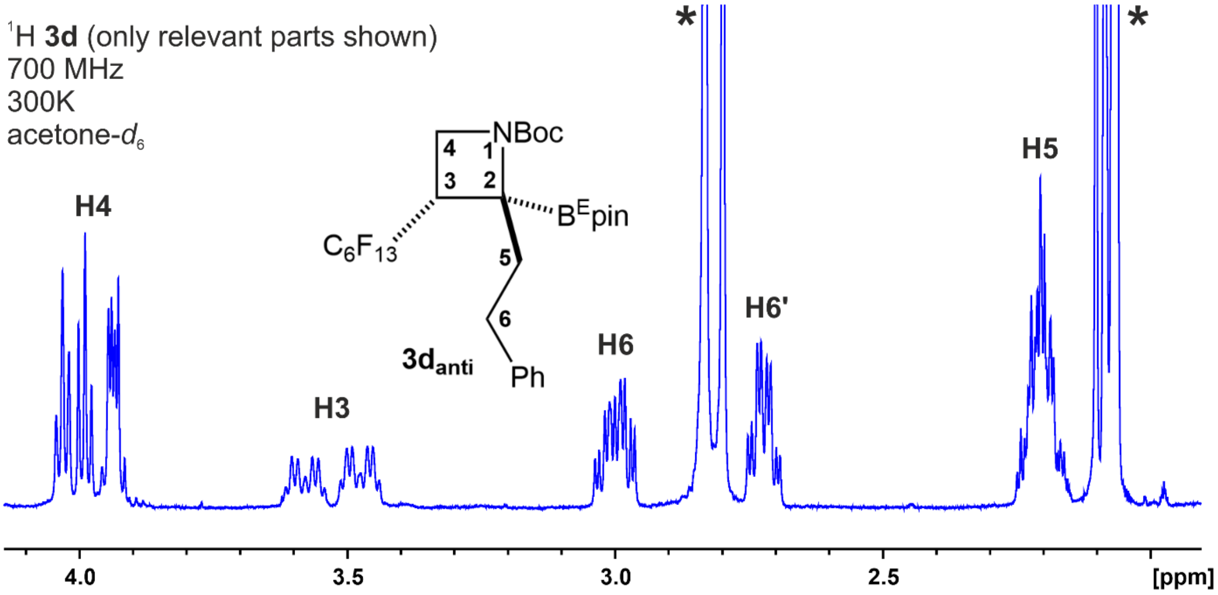

Supplementary Figure 10: ^{1}H NMR spectrum (acetone-d_6, 700 MHz, 300 K) of compound **3d**. Only the part relevant to this analysis is shown.

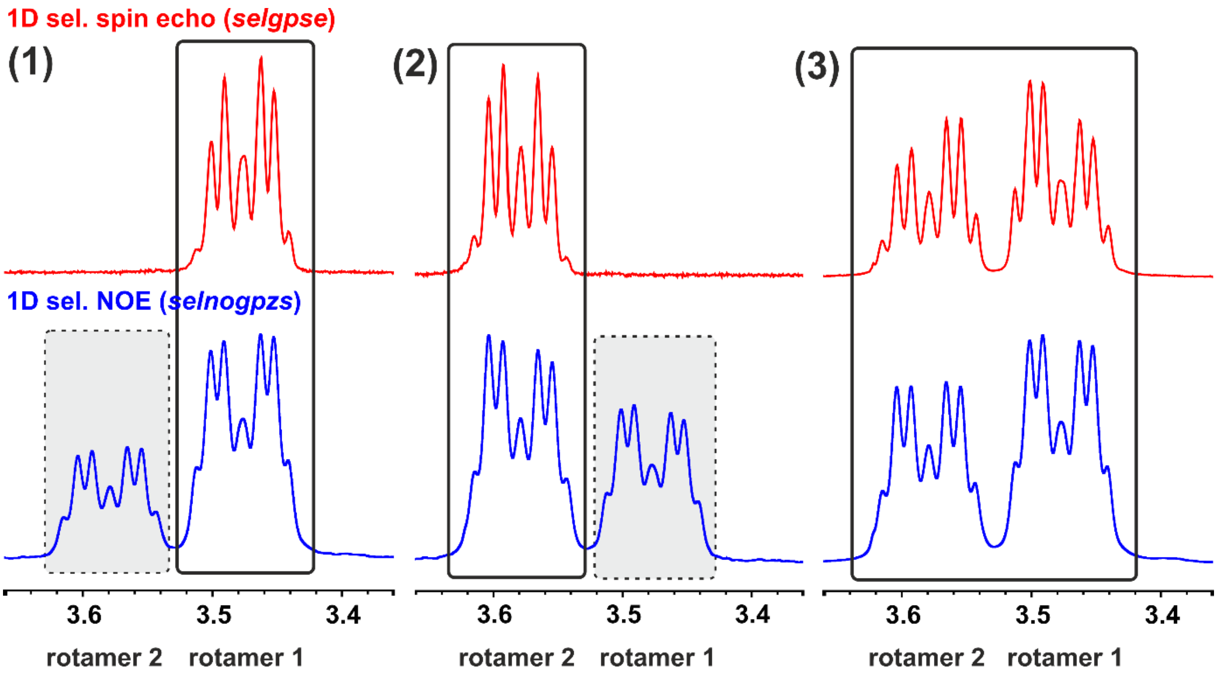

Supplementary Figure 11: 1D sel. NOE (blue, bottom) and 1D sel. spin echo (red, top) spectra of the two different H3s of compound **3d** (700 MHz, 300 K, acetone-d_6). The boxes with black solid lines show which proton (or combination of protons) is selectively refocused. The grey boxes symbolize signals caused by chemical exchange. In (1) $H3_{anti}$ of rotamer 1 and in (2) $H3_{anti}$ of rotamer 2 is refocused. In (3) $H3_{anti}$ of both rotamers are refocused simultaneously. Durations, bandwidths and offsets of the shaped pulses (RSnob) are given in table SF-6.

The comparison of the 1D sel. spin echo (red) and 1D sel. NOE spectra (blue) in figure SF-11 allows to clearly identify the two signals for H3 as caused by chemical exchange: The 1D sel. spin echo spectra show a clean selective refocusing of both different signals in (1) and (2). In the respective 1D sel. NOE spectra, where the same selective pulses were used, the non-refocused signals are visible as signals with the same sign as the refocused one, proofing that the existence of the second signal set is caused by chemical exchange. If the non-refocused signal would originate from a NOE contact, it would have the opposite phase/sign as the refocused signal.[16]

To determine the relative configuration of **3d** a 2D NOESY and a 2D HOESY are acquired. The NOESY is shown in figure SF-12.

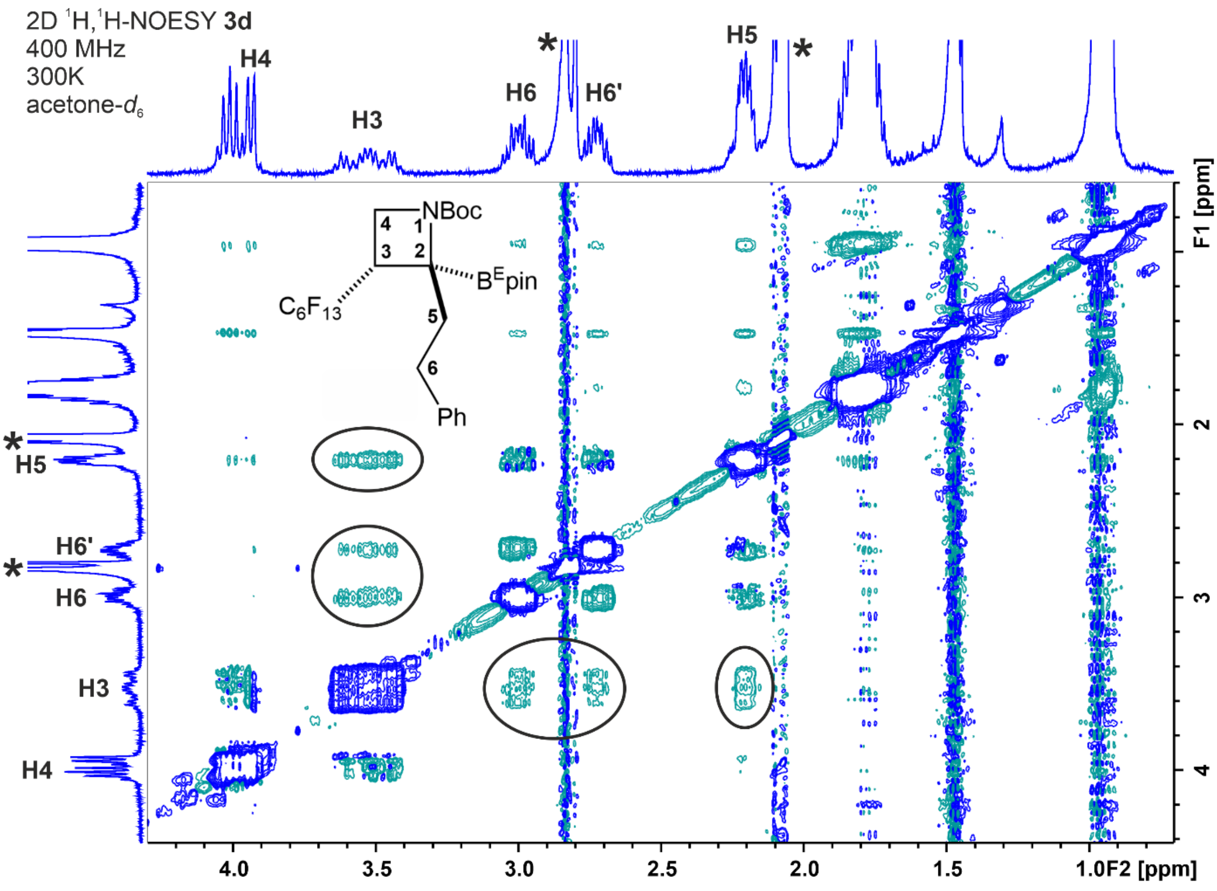

Supplementary Figure 12: 2D NOESY spectrum of compound **3d** in acetone-*d*6 at 400 MHz and 300 K. The NOE cross peaks between H3 and the phenethyl side chain (H5, H6, H6') are marked with black ellipses.

The NOESY spectrum (figure SF-12) shows strong NOEs between H3 and the protons H5, H6 and H6' of the phenethyl side chain indicating that H3 and the phenethyl side chain are located on the same side of the heterocycle. This implies that the phenethyl side chain and the perfluorinated side chain have to be on opposite sides, resulting in the *anti* diastereomer being the sole product of the reaction. This can be further supported by acquisition of a 2D $^{1}H,^{19}F$-HOESY spectrum which is shown in figure SF-13.

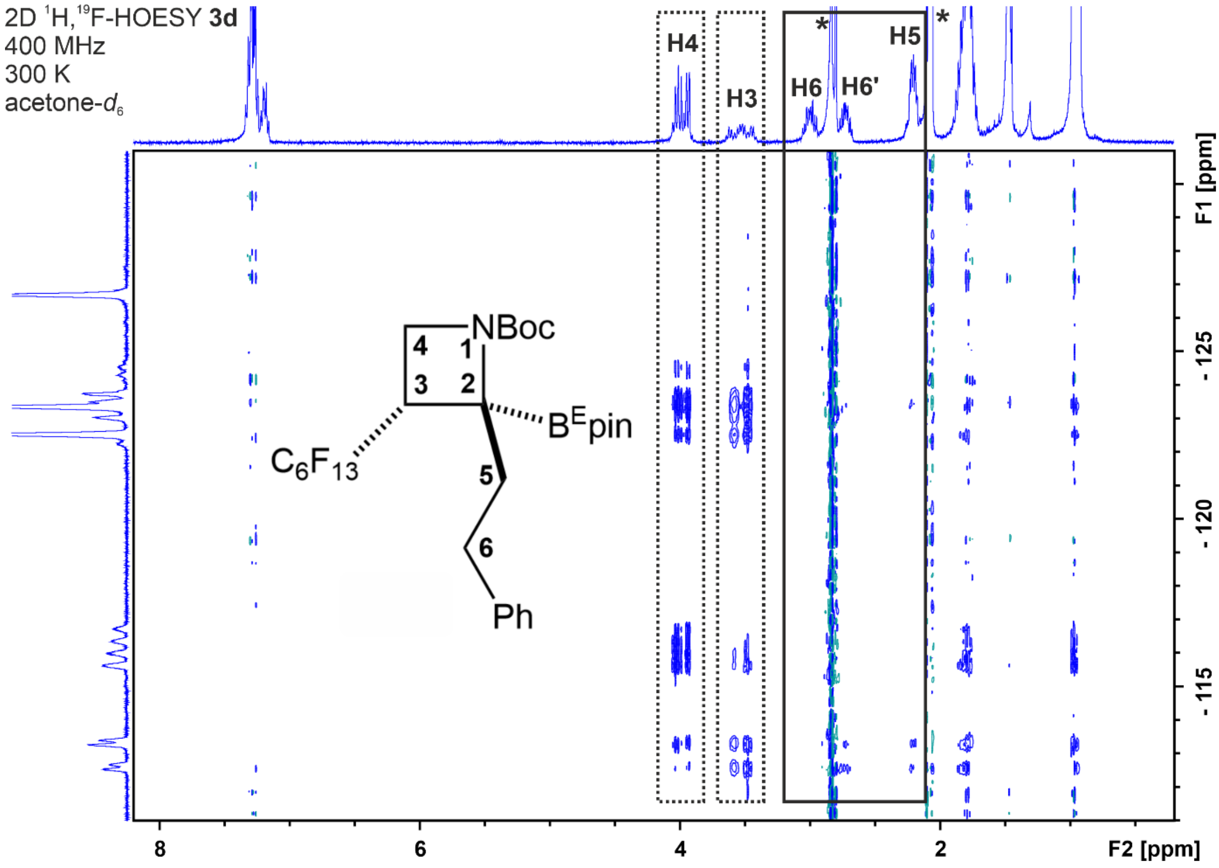

Supplementary Figure 13: 2D HOESY spectrum of compound **3d** in acetone-d_6 at 400 MHz and 300 K.

No significant HOE is observed between the perfluorinated side chain and the protons H5, H6 and H6' in the phenethyl side chain, allowing the unequivocal assignment of the relative configuration to be *anti* (phenethyl to perfluorinated side chain). This diastereomer is the sole/major diastereomer formed during the reaction.

Additionally, intense HOEs can be observed between the perfluorinated side chain and the protons H3 and H4 (black box with dashed line) which are located in close proximity.

7. References

[1] R. Rubio-Presa, S. Suárez-Pantiga, M. R. Pedrosa, R. Sanz, *Adv. Synth. Catal.* **2018**, *360*, 2216

[2] J. L. Wood, L. D. Marciasini, M. Vaultier, M. Pucheault, *Synlett* **2014**, *25*, 551-555.

[3] D. M. Hodgson, C. I. Pearson, M. Kazmi, *Org. Lett.* **2014**, *16*, 856-859.

[4] E. Cesarotti, P. Antognazza, M. Pallavicini, L. Villa, *Helv. Chim. Acta* **1993**, *76*, 2344-2349.

[5] J. A. Myhill, C. A. Wilhelmsen, L. Zhang, J. P. Morken, *J. Am. Chem. Soc.* **2018**, *140*, 15181-15185.

[6] F. Sladojevich, E. McNeill, J. Börgel, S.-L. Zheng, T. Ritter, *Angew. Chem. Int. Ed.* **2015**, *54*, 3712-3716.

[7] G. Yin, M. Gao, N. She, S. Hu, A. Wu, Y. Pan, *Synthesis* **2007**, *2007*, 3113-3116.

[8] L. P. Jayathilaka, M. Deb, R. F. Standaert, *Org. Lett.* **2004**, *6*, 3659-3662.

[9] H. E. Montenegro, P. Ramírez-López, M. C. de la Torre, M. Asenjo, M. A. Sierra, *Chem. - Eur. J.* **2010**, *16*, 3798-3814.

[10] B. Schweder, E. Uhlig, *J. Prakt. Chem.* **1991**, *333*, 223-228.

[11] P. Mayo, W. Tam, *Tetrahedron* **2002**, *58*, 9527-9540.

[12] K. Stott, J. Keeler, Q. N. Van, A. J. Shaka, *J. Magn. Reson.* **1997**, *125*, 302–324.

[13] M. J. Thrippleton, J. Keeler, *Angew. Chem. Int. Ed.* **2003**, *42*, 3938–3941.

[14] R. Wagner, S. Berger, *J. Magn. Reson. A* **1996**, *123*, 119–121.

[15] C. Yu, G. C. Levy, *J. Am. Chem. Soc.* **1984**, *106*, 6533–6537.

[16] D. X. Hu, P. Grice, S. V. Ley, *J. Org. Chem.* **2012**, *77*, 5198–5202.
