# Supplementary material for: Stereoselective polar radical crossover for the functionalization of strained-ring systems
Source: Commun Chem. 2024 Jun 19;7:139. doi: 10.1038/s42004-024-01221-3 (PMC11187220; doi:10.1038/s42004-024-01221-3)
Supplement: Supplementary file 3 — Description of Additional Supplementary Files [file 42004_2024_1221_MOESM3_ESM.pdf]

Description of Additional Supplementary Files

File name- Supplementary Data 1

File description- all 1H and 13C NMR spectra.
