# Supplementary material for: Stereoselective polar radical crossover for the functionalization of strained-ring systems
Source: Commun Chem. 2024 Jun 19;7:139. doi: 10.1038/s42004-024-01221-3 (PMC11187220; doi:10.1038/s42004-024-01221-3)
Supplement: Supplementary file 4 — Supplementary Data 1 [file 42004_2024_1221_MOESM4_ESM.pdf]

NMR Spectra

***tert*-Butyl 3-methoxyazetidine-1-carboxylate (Si-1)**

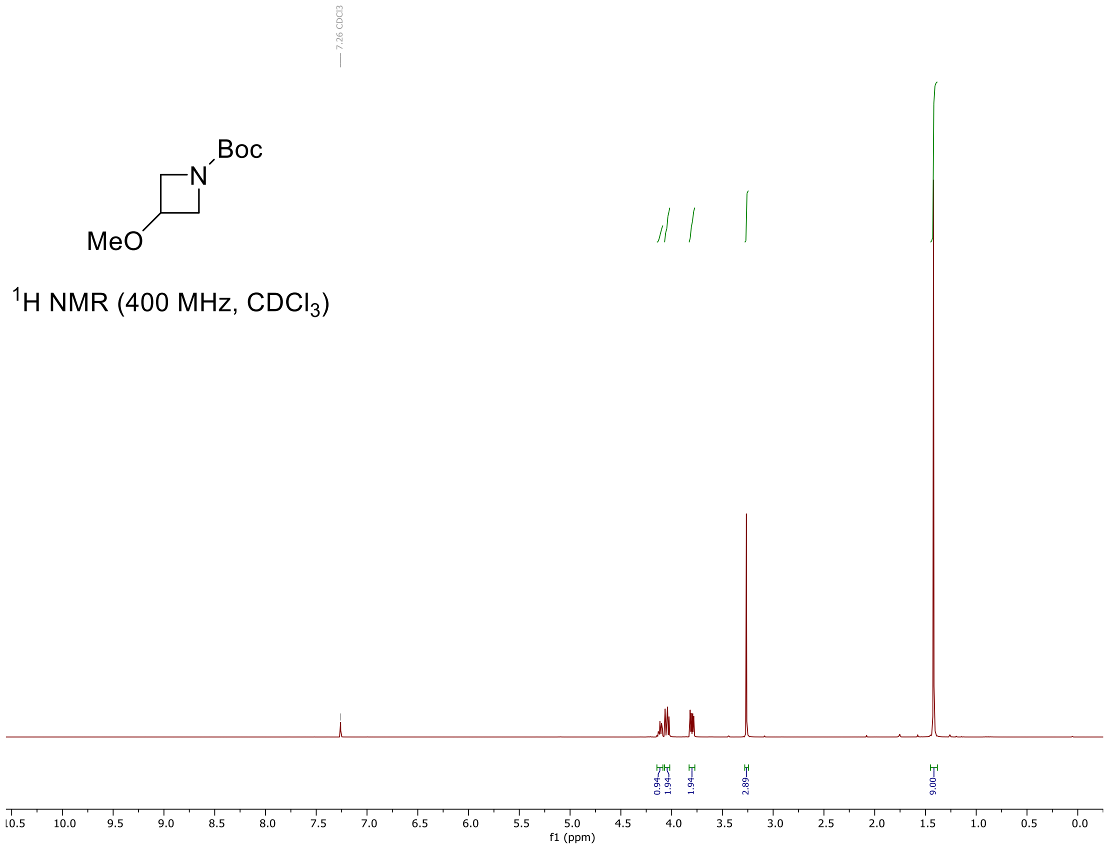

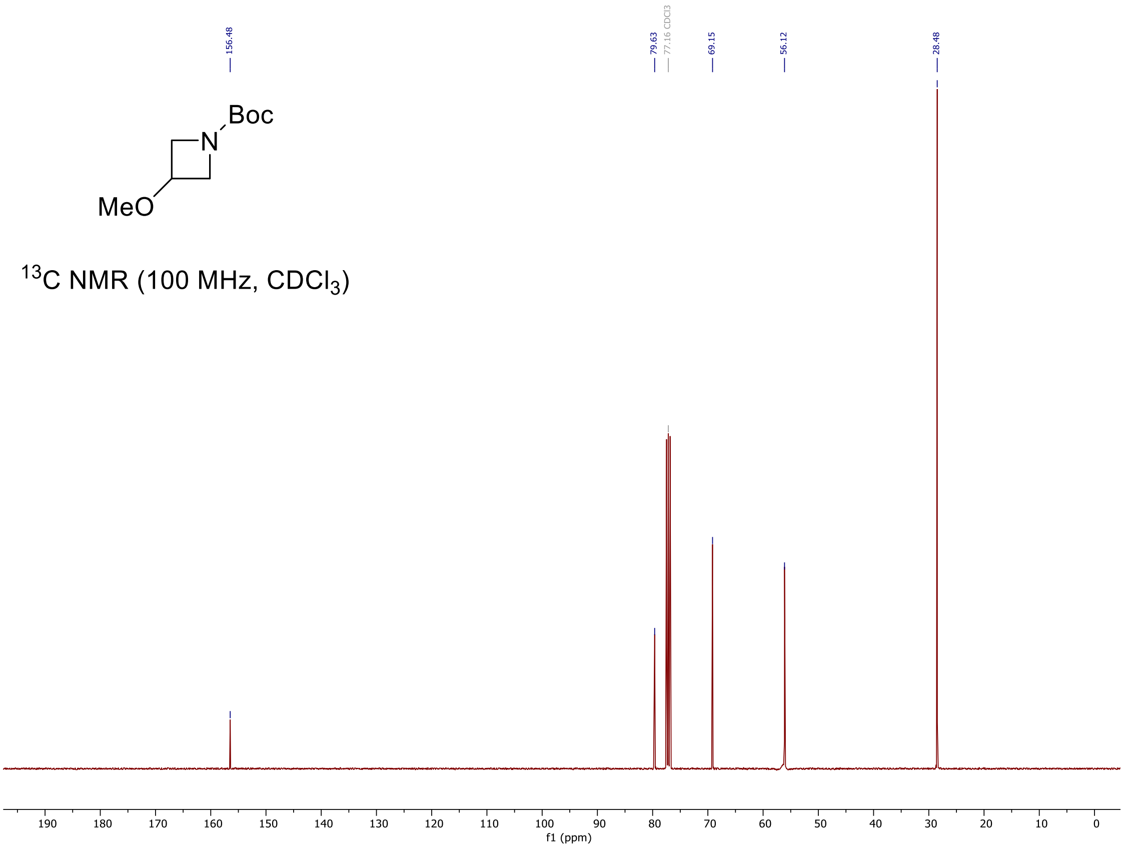

3,4-Diethylhexane-3,4-diol (L-2)

^{1}H NMR (400 MHz, $CDCl_3$)

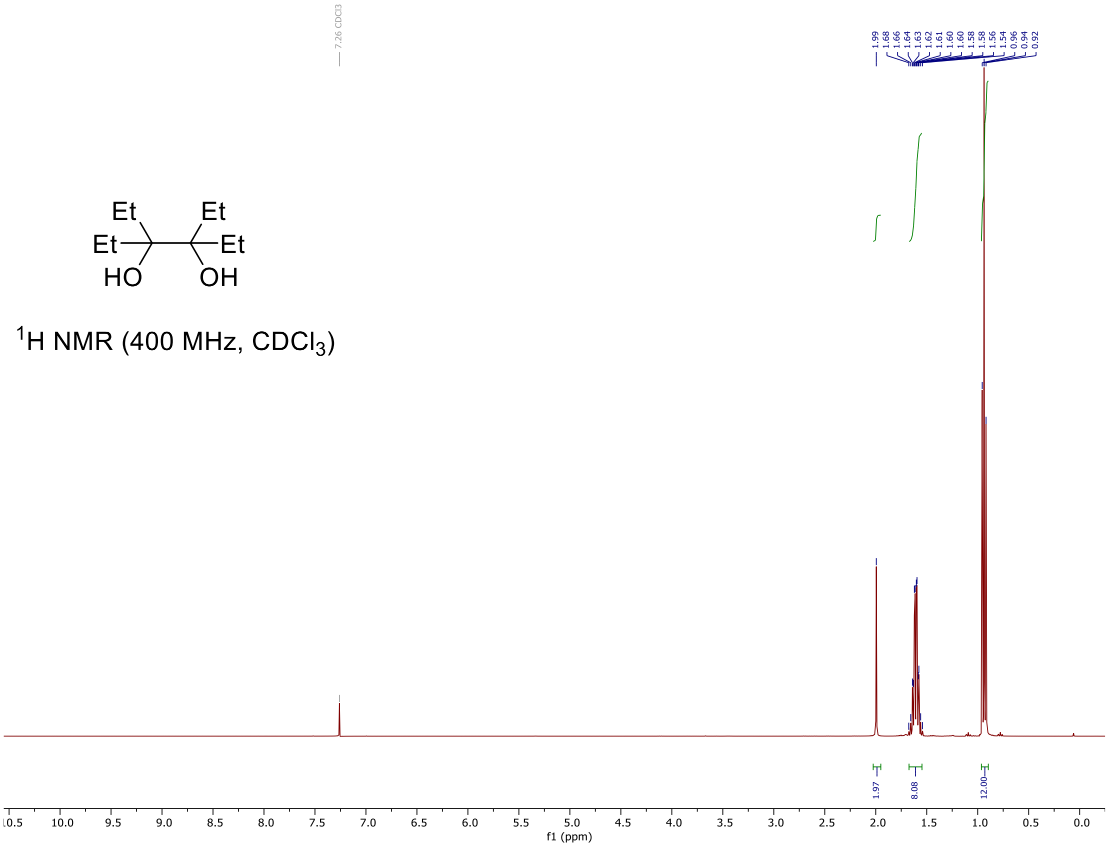

^{13}C NMR (100 MHz, $CDCl_3$)

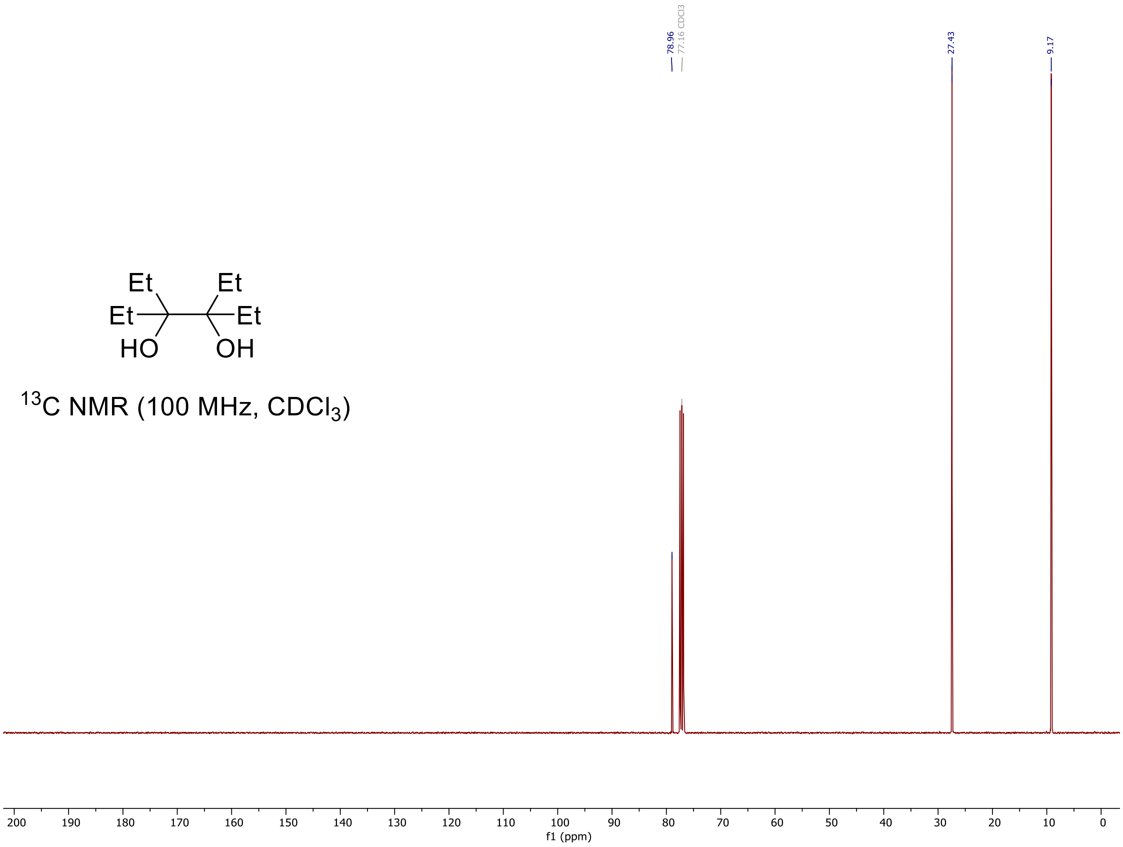

[1,1'-Bi(cyclopentane)]-1,1'-diol (L-3)

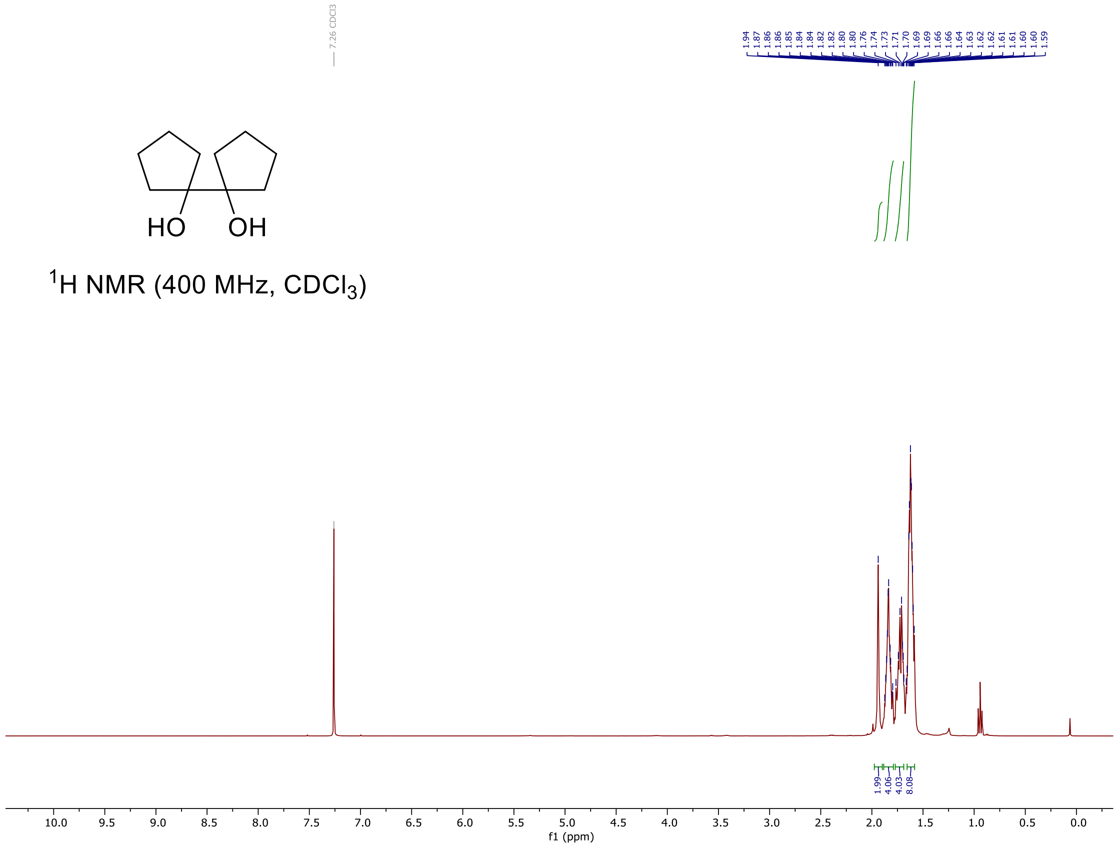

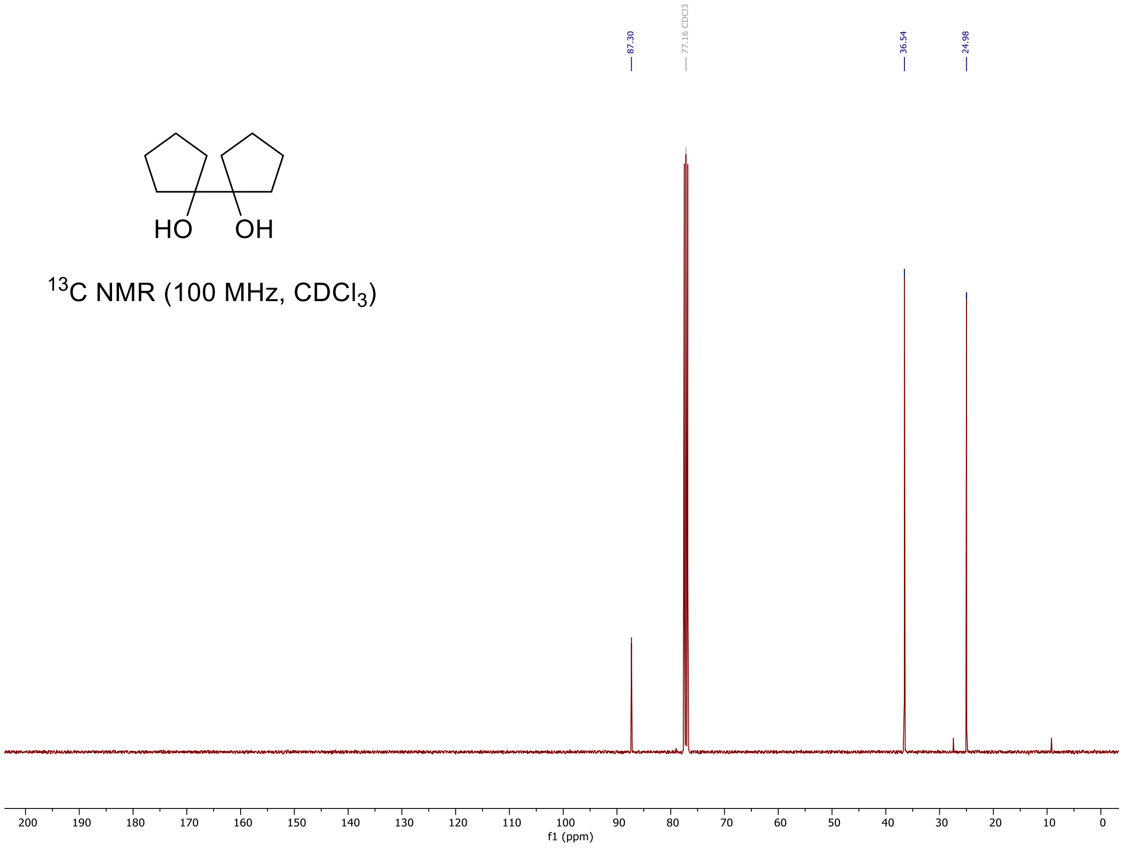

2,4-Dimethylpentane-2,4-diol (L-5)

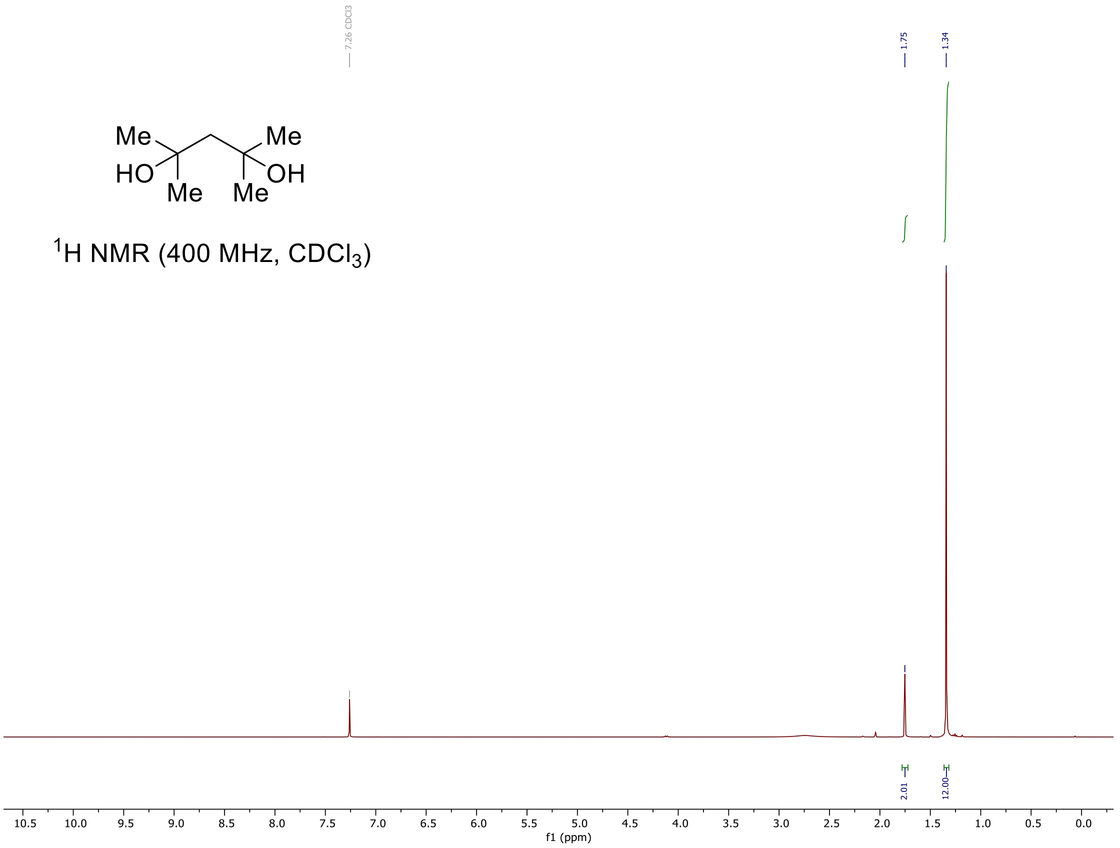

^{1}H NMR (400 MHz, $CDCl_3$)

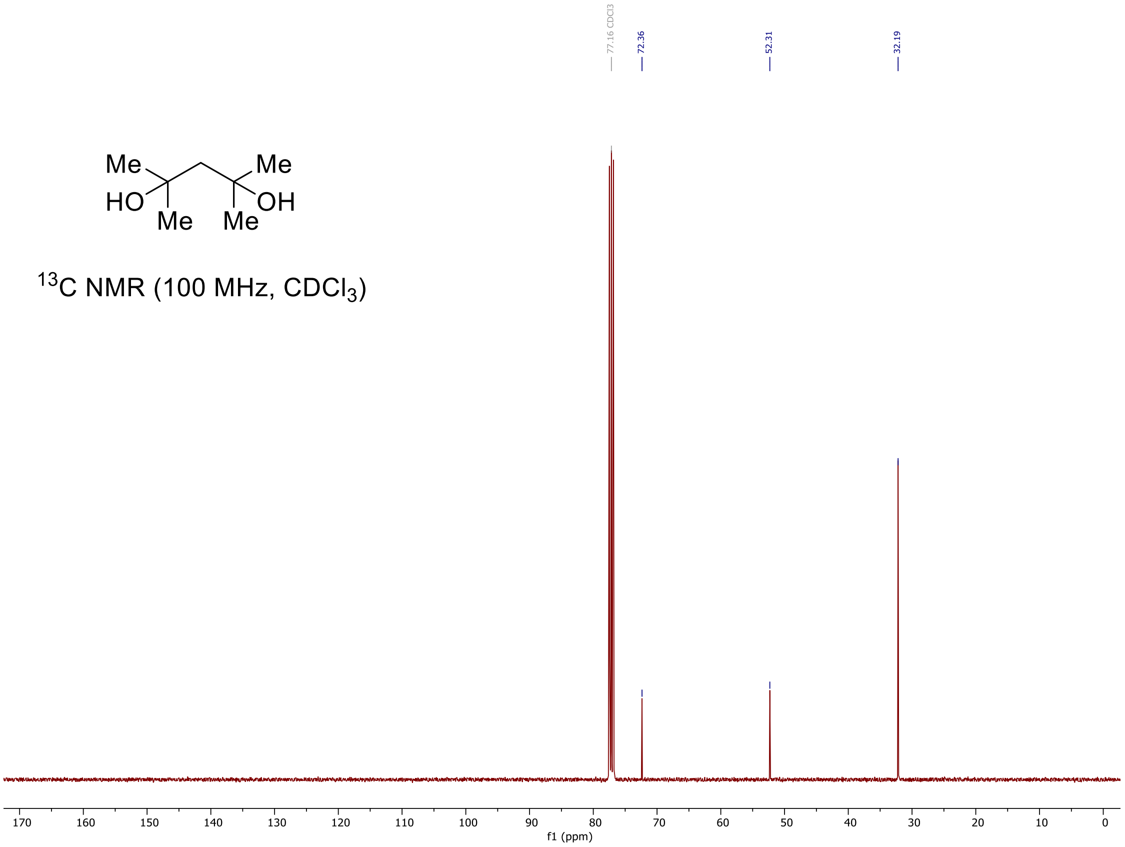

^{13}C NMR (100 MHz, $CDCl_3$)

2,2-Dimethoxypropane-1,3-diol (L-6)

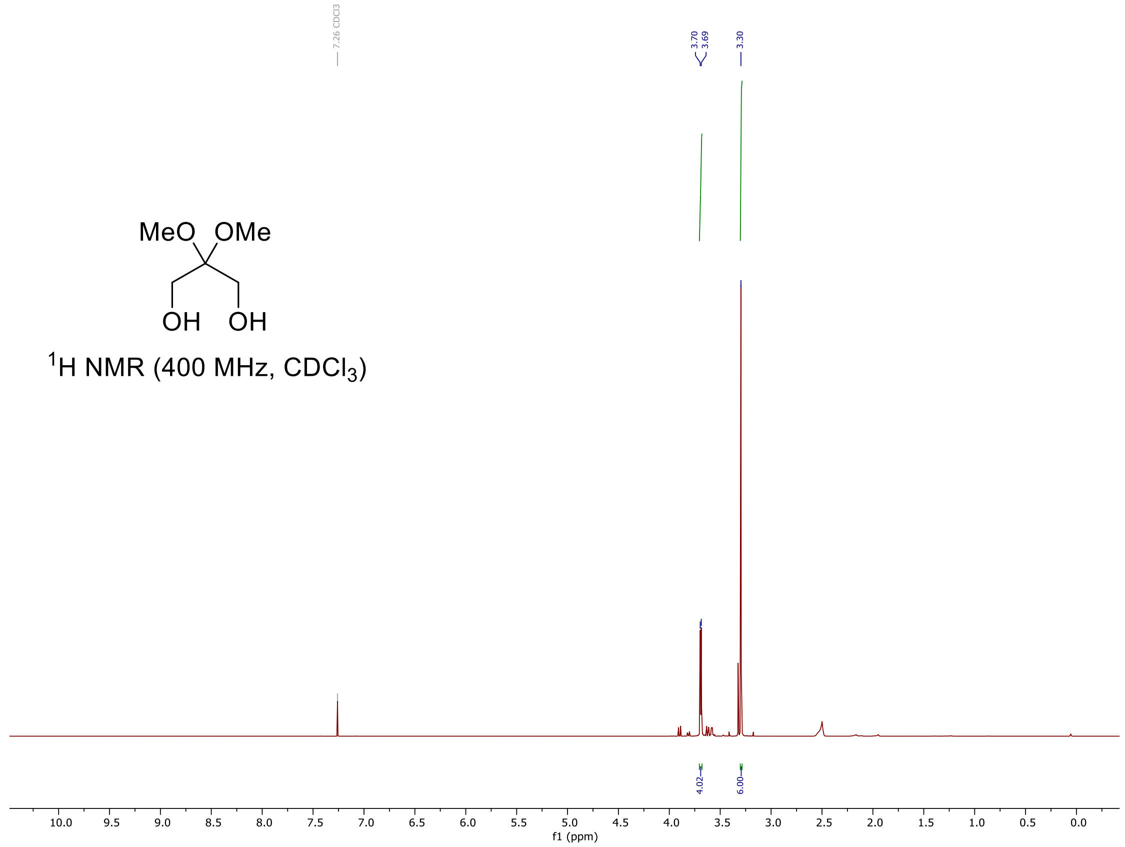

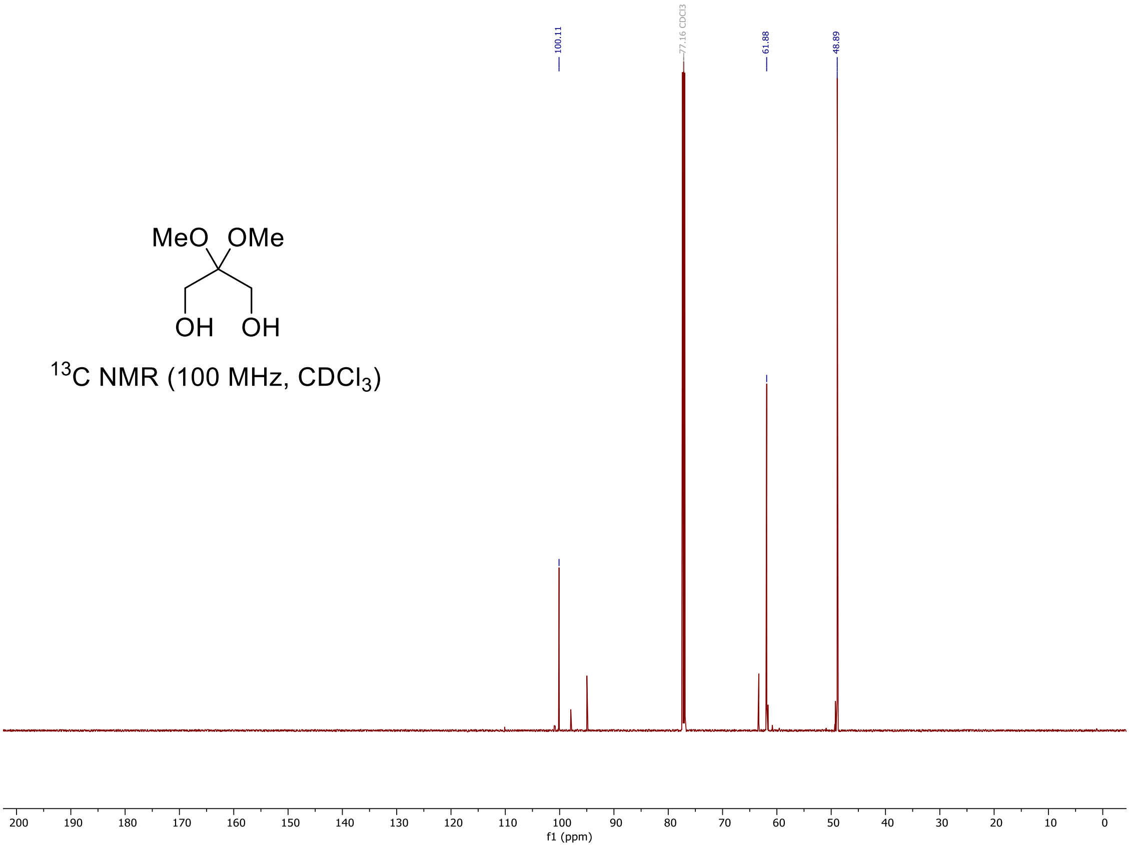

(1*R*,2*S*)-1,2-Dimethyl-1,2-dihydroacenaphthylene-1,2-diol (<u>L-7</u>)

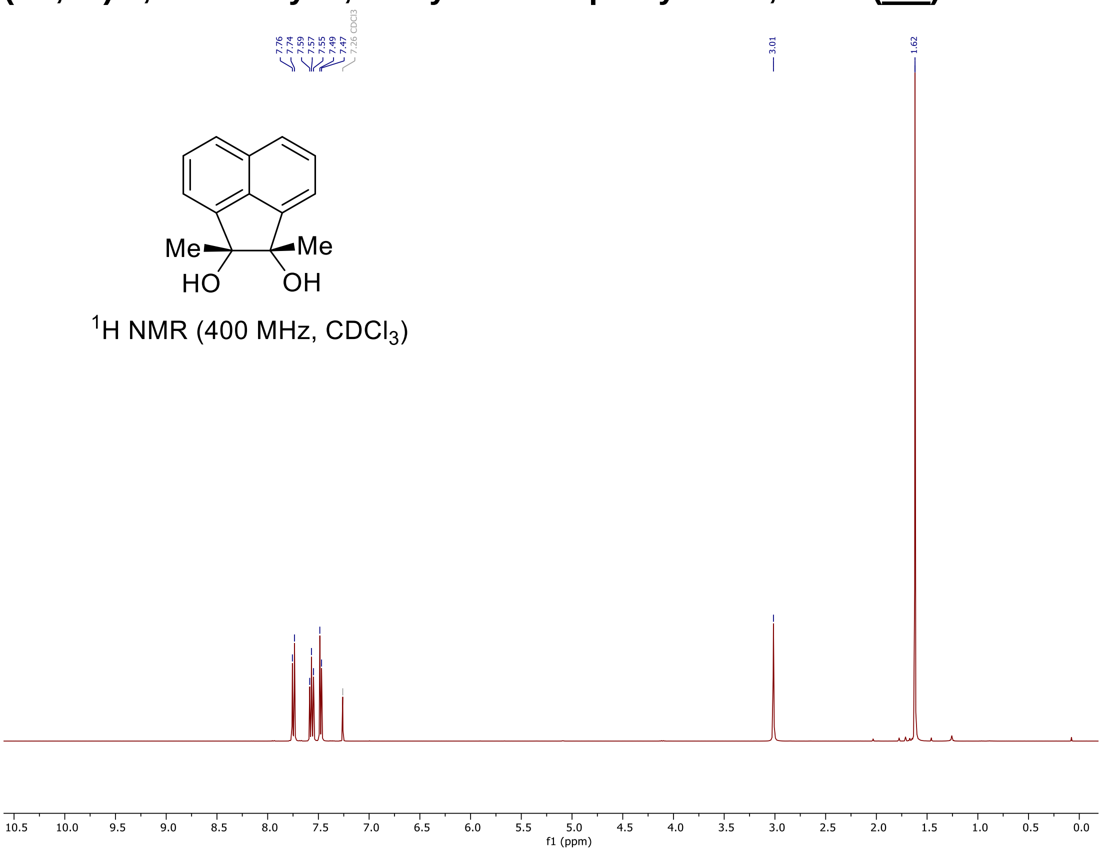

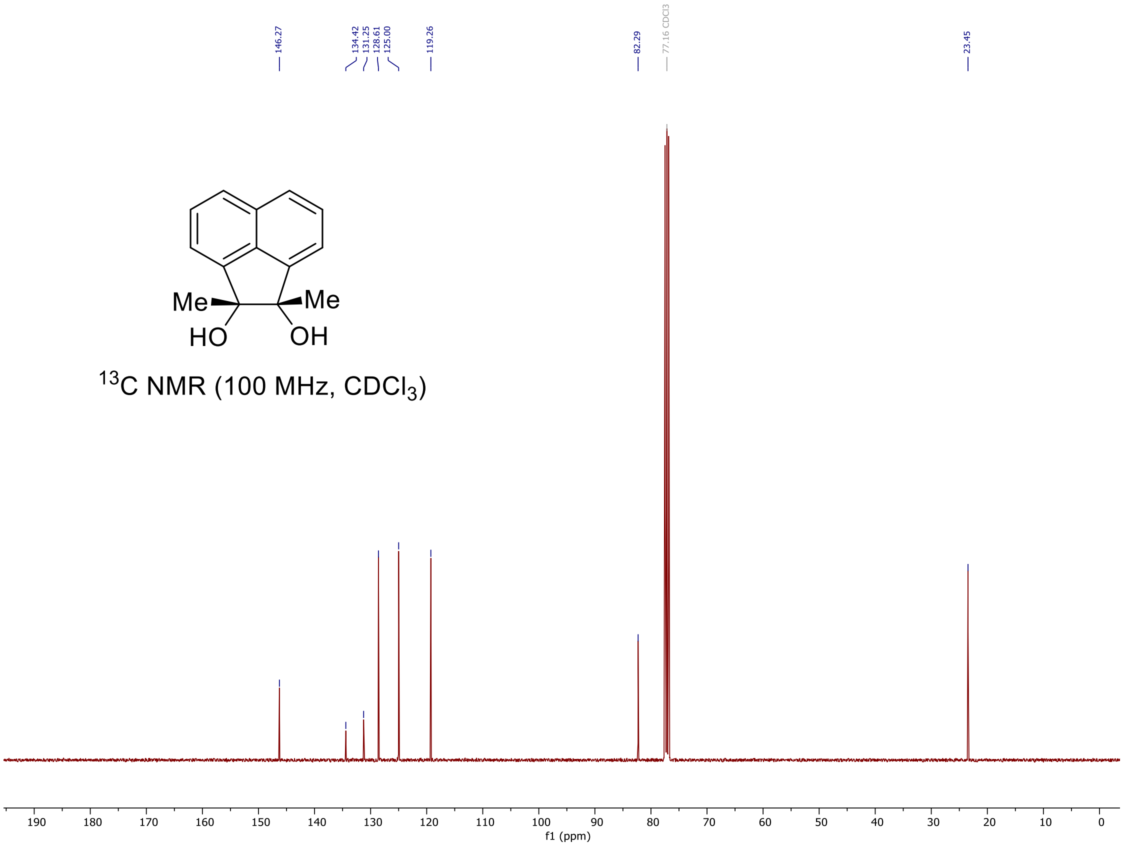

3,4-Diisopropyl-2,5-dimethylhexane-3,4-diol (<u>L-9</u>)

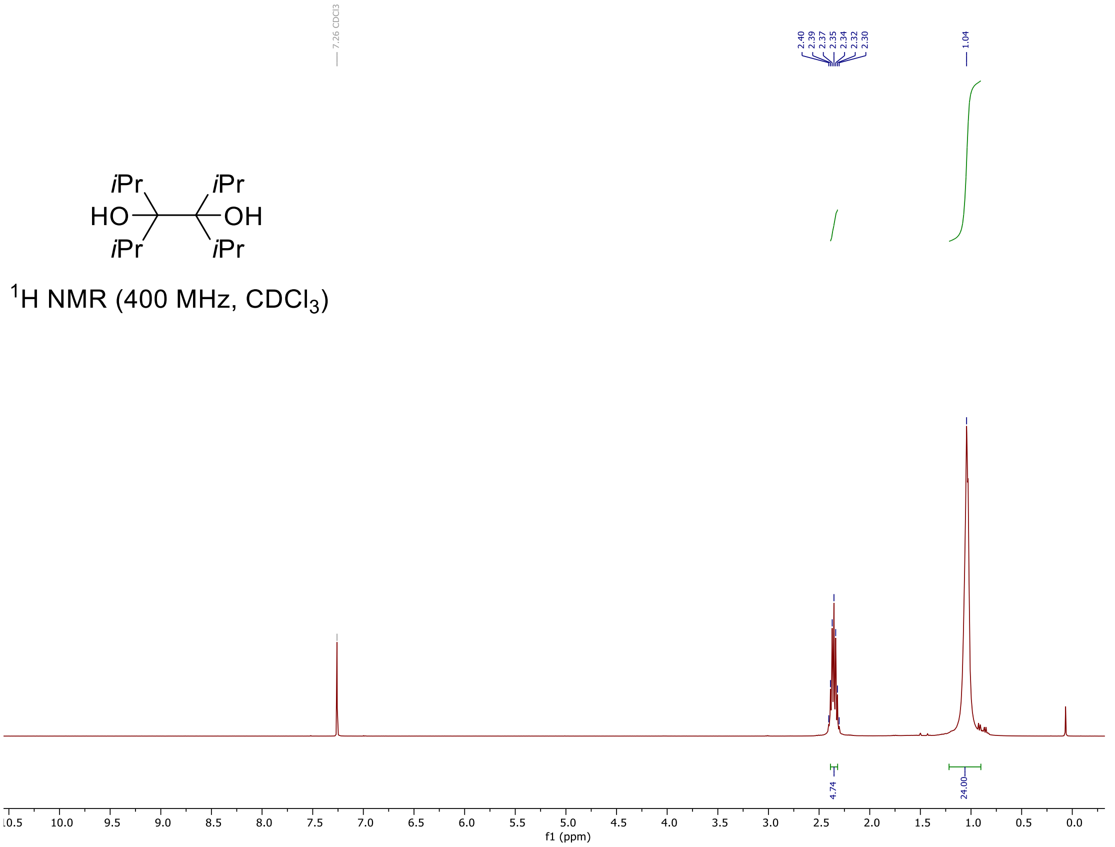

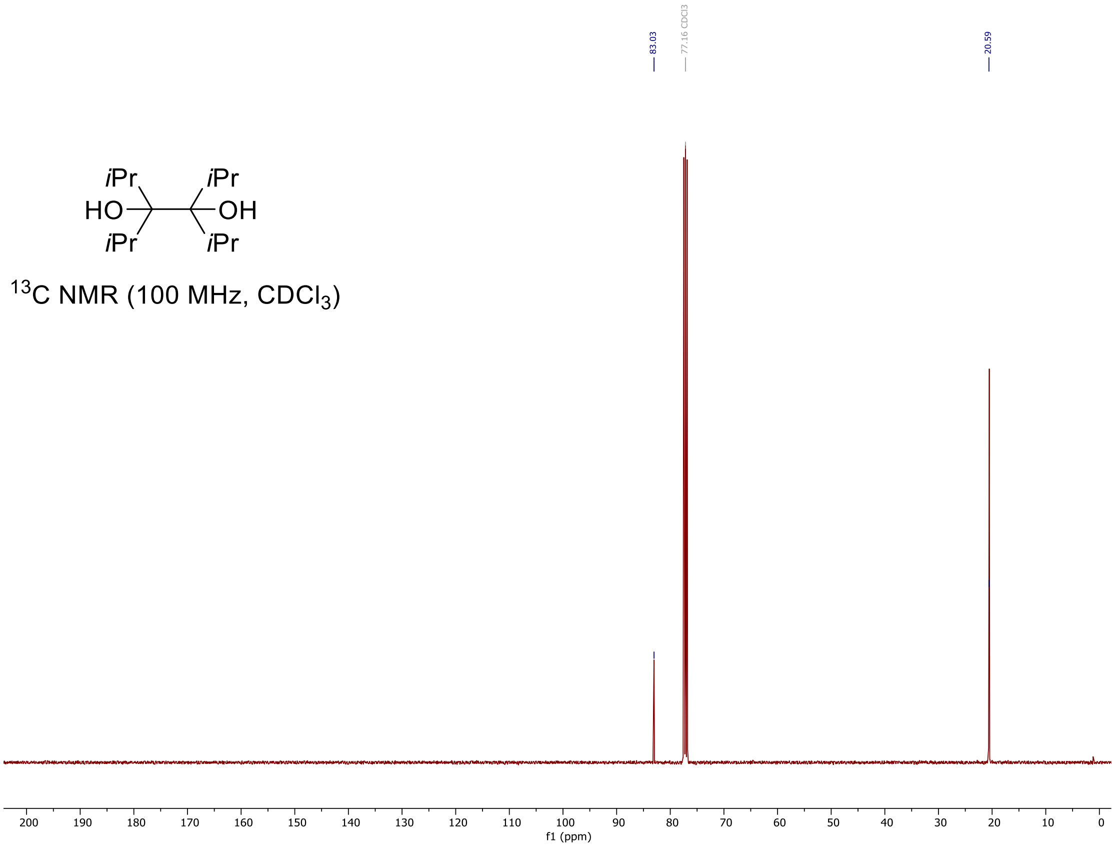

2-Butyl-4,4,5,5-tetramethyl-1,3,2-dioxaborolane (Si-2)

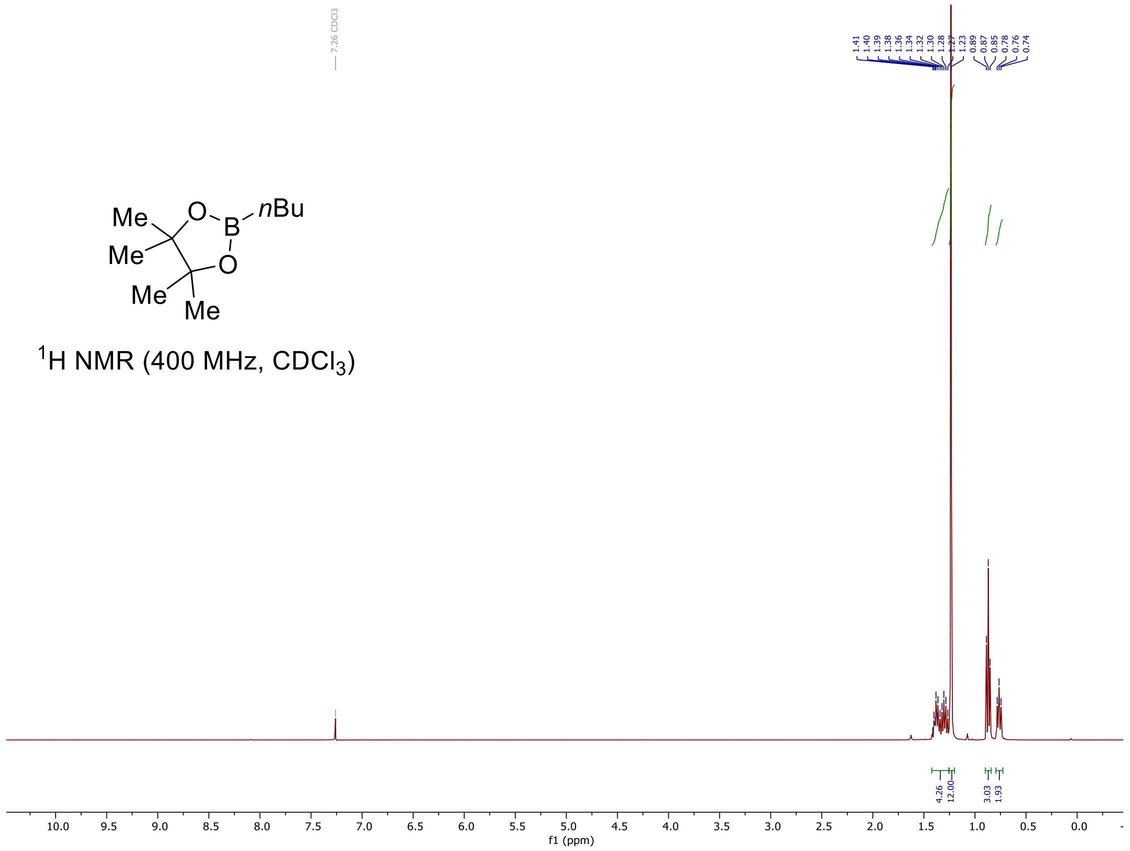

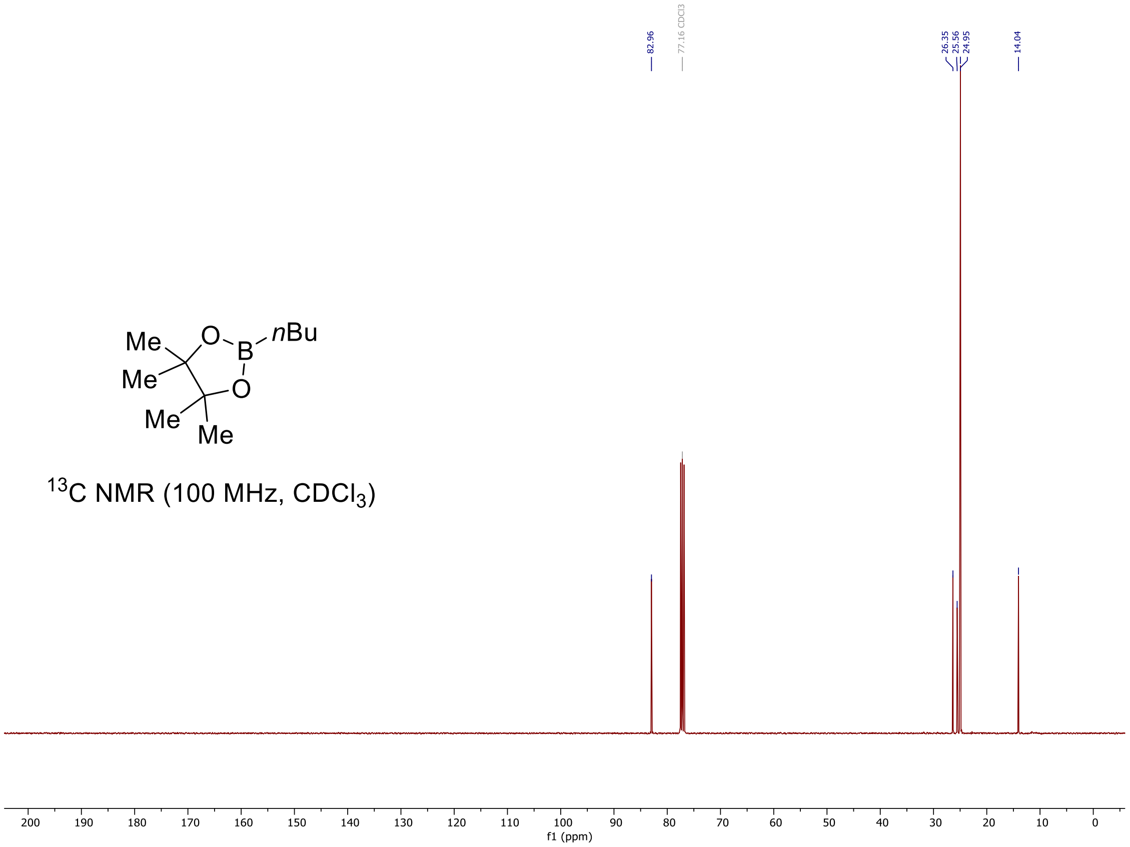

2-Butyl-4,4,5,5-tetraethyl-1,3,2-dioxaborolane (Si-3)

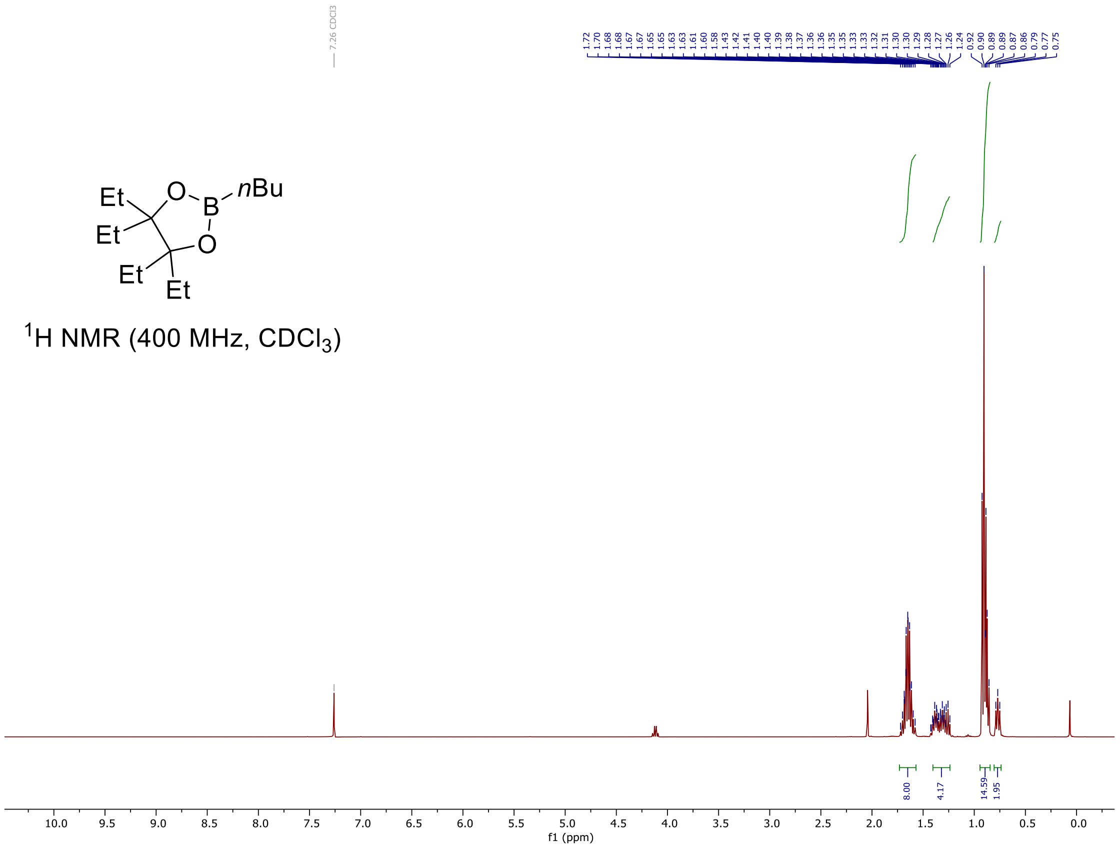

^{1}H NMR (400 MHz, $CDCl_3$)

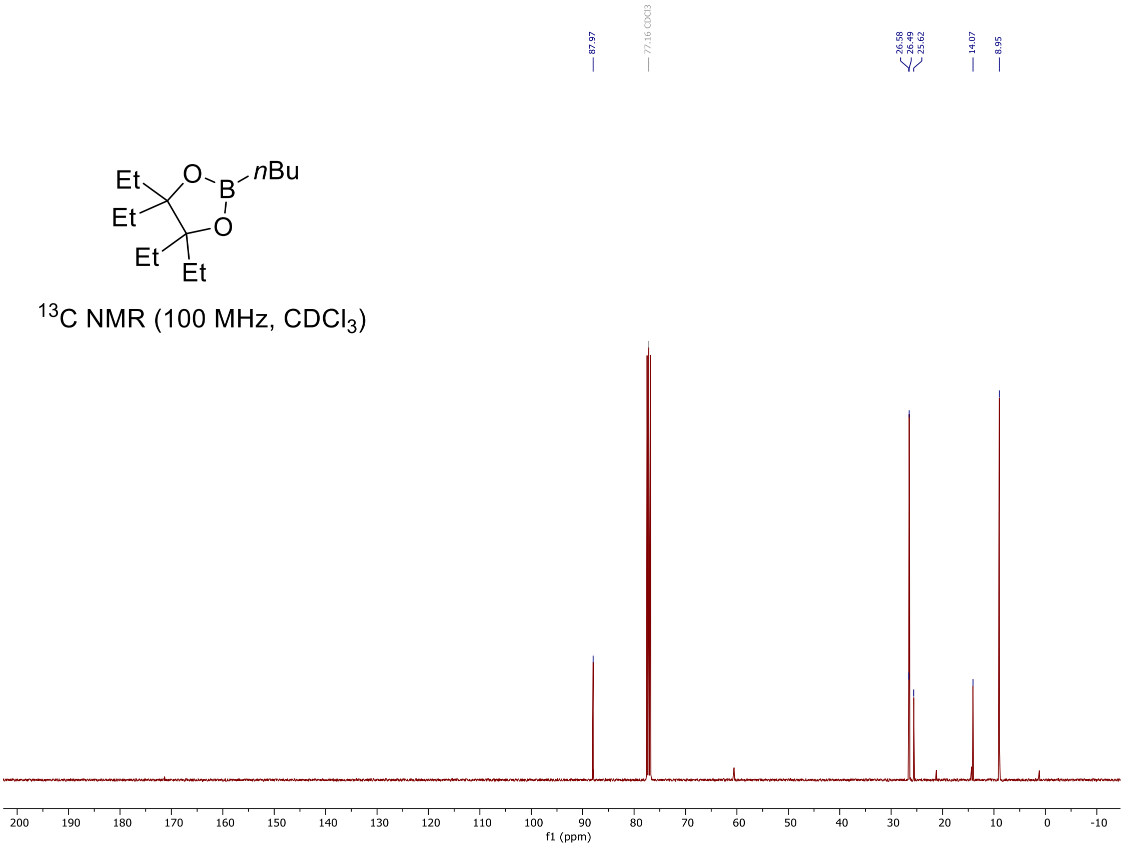

^{13}C NMR (100 MHz, $CDCl_3$)

12-Butyl-11,13-dioxa-12-boradispiro[4.0.46.35]tridecane (Si-4)

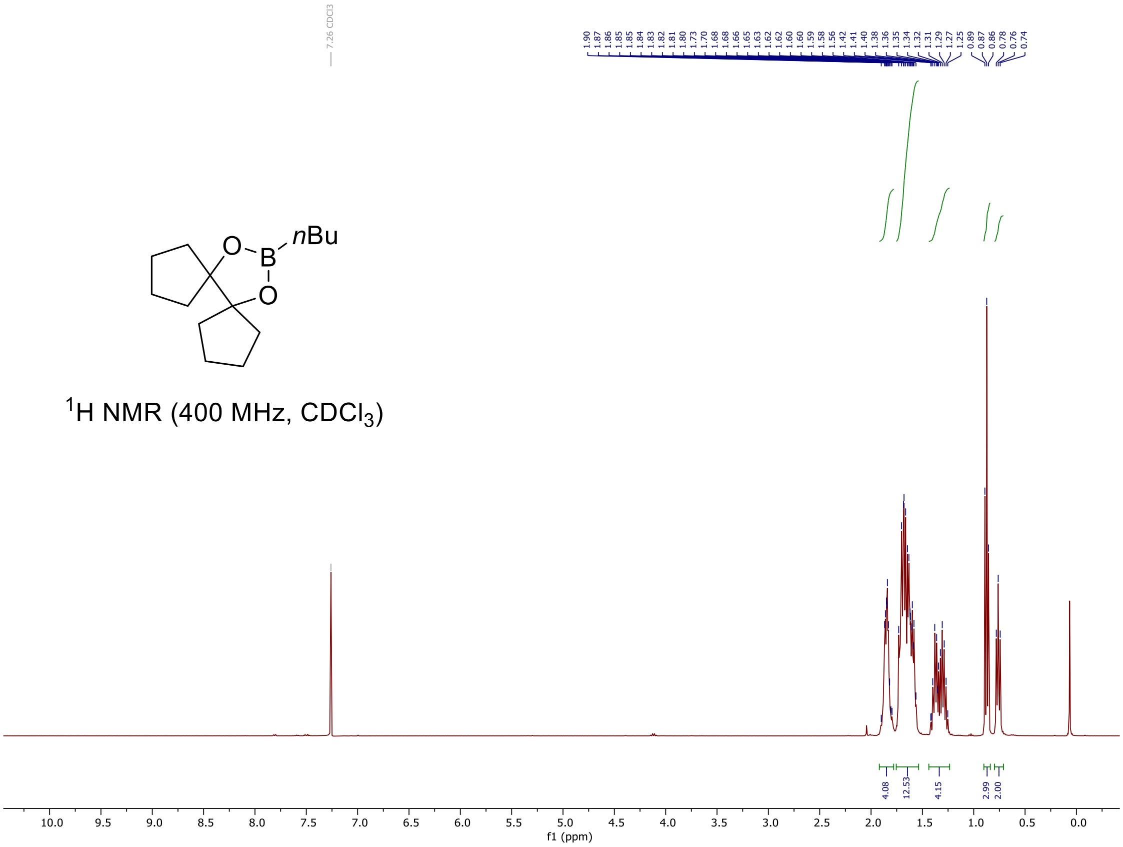

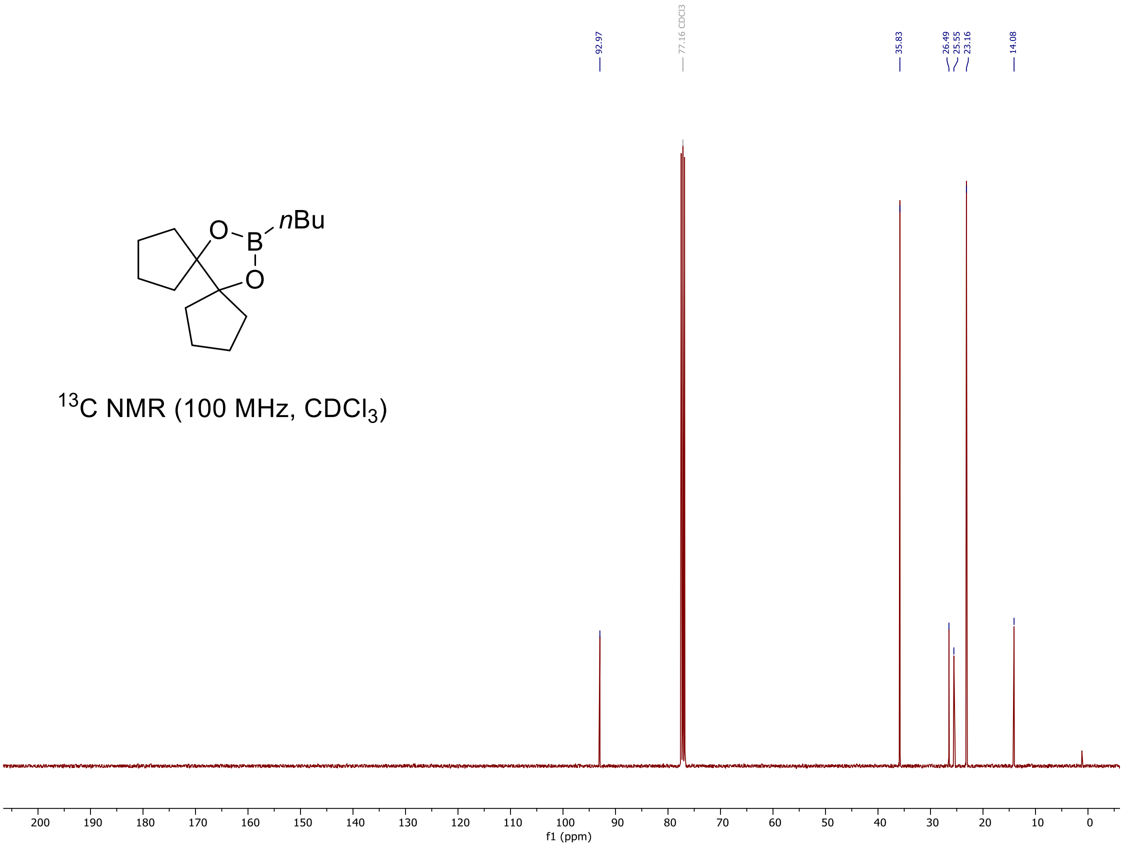

2-Butyl-5,5-dimethyl-1,3,2-dioxaborinane (Si-5)

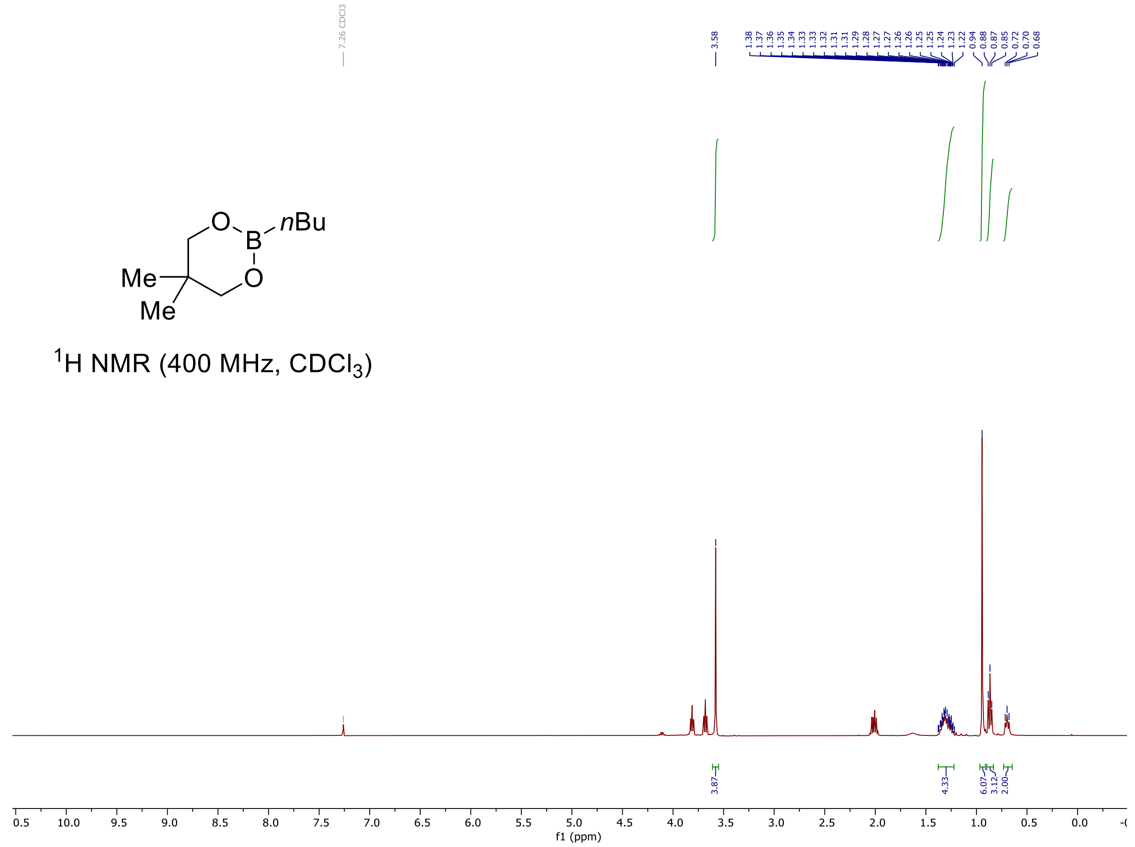

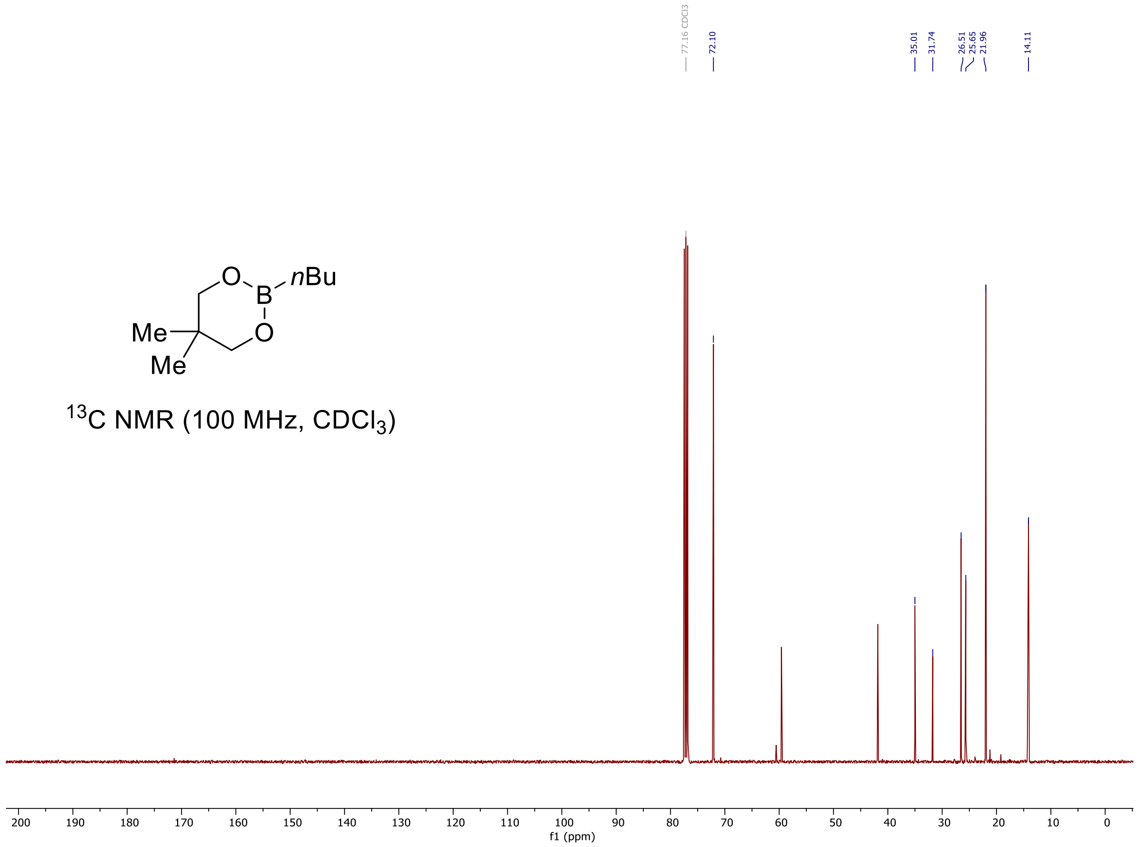

2-Butyl-4,4,6,6-tetramethyl-1,3,2-dioxaborinane (Si-6)

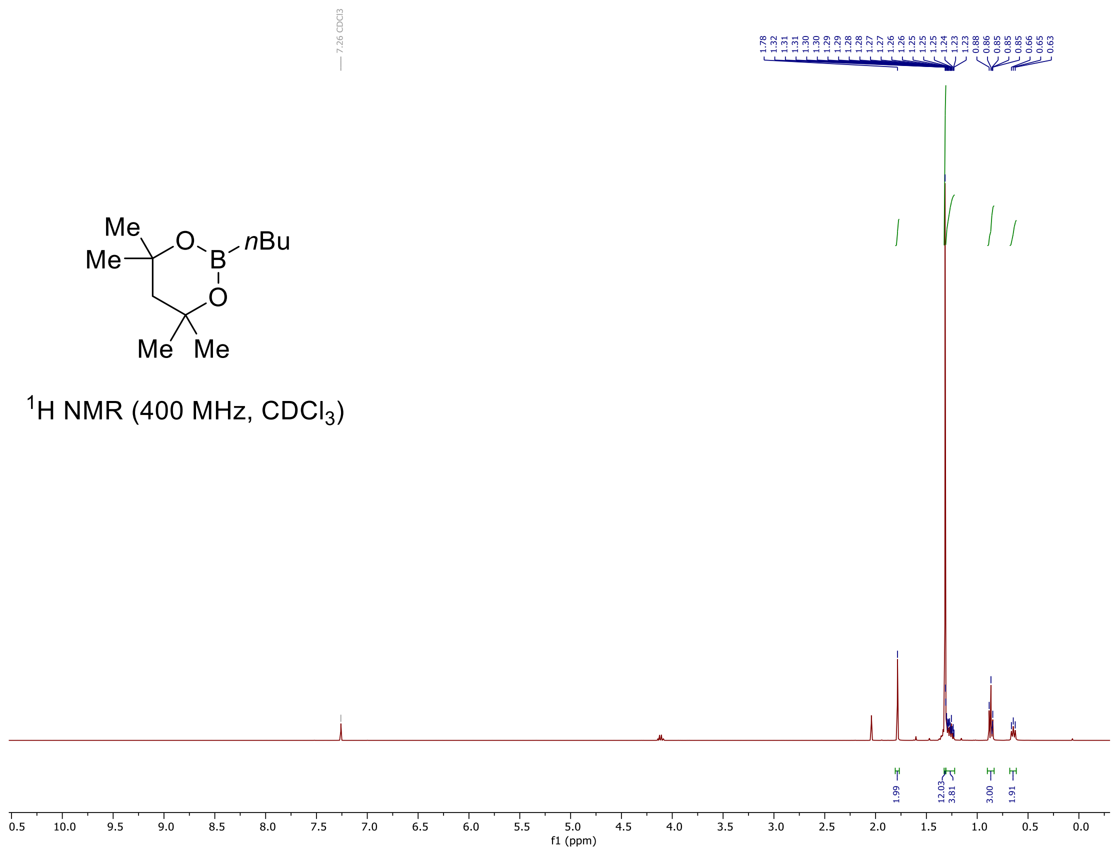

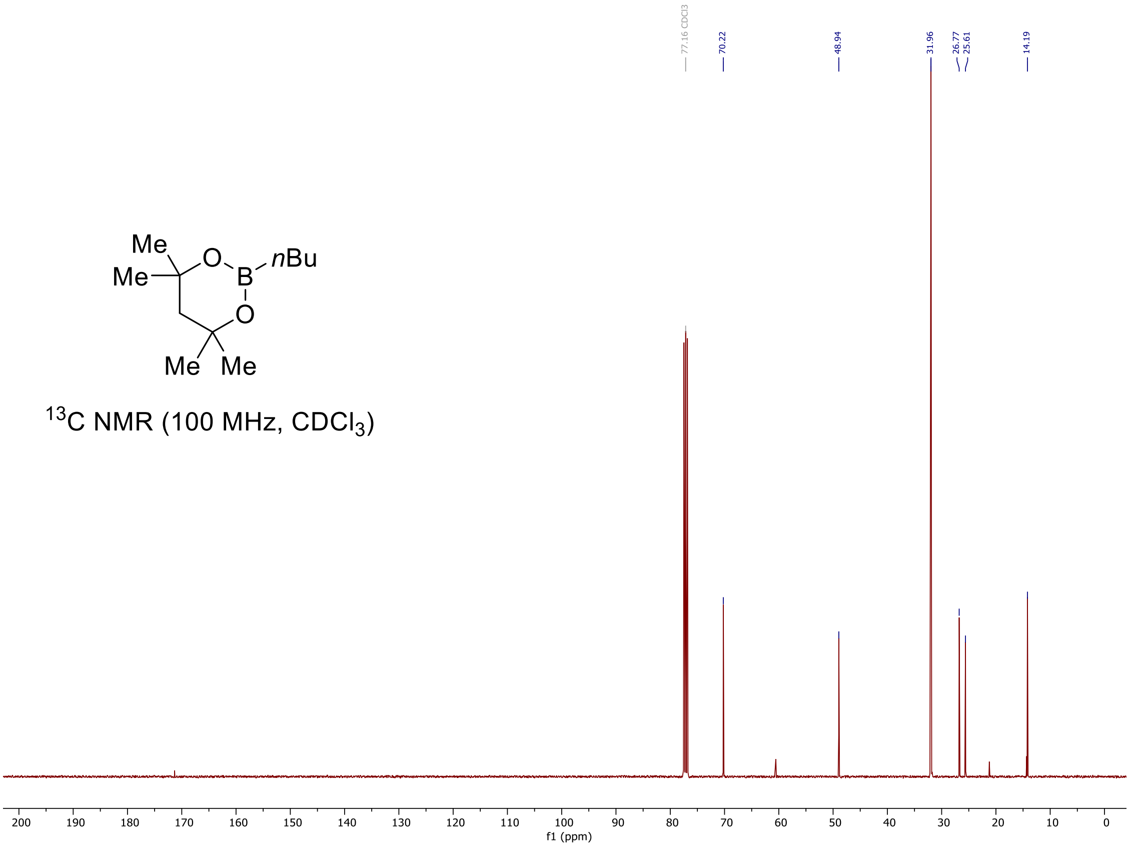

2-Butyl-5,5-dimethoxy-1,3,2-dioxaborinane (Si-7)

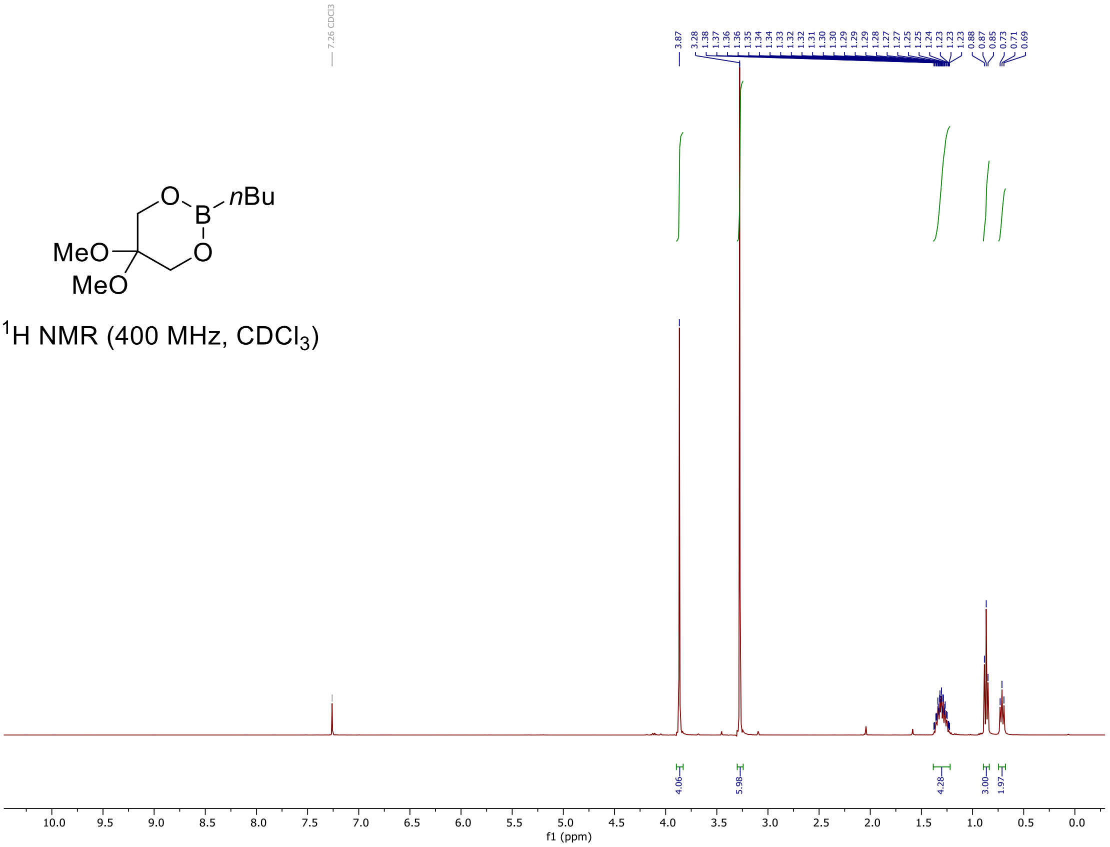

^{1}H NMR (400 MHz, $CDCl_3$)

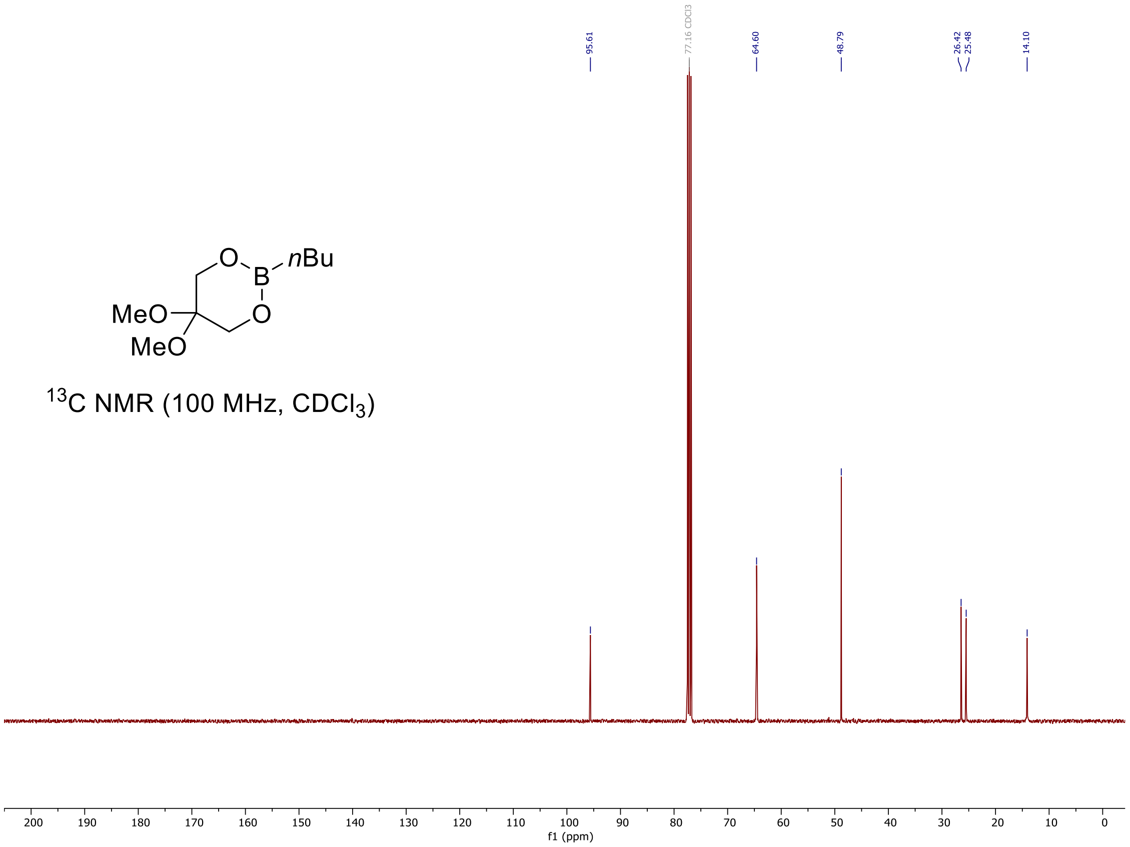

^{13}C NMR (100 MHz, $CDCl_3$)

(6b*R*,9a*S*)-8-Butyl-6b,9a-dimethyl-6b,9a-dihydroacenaphtho[1,2-d][1,3,2]dioxaborole (Si-8)

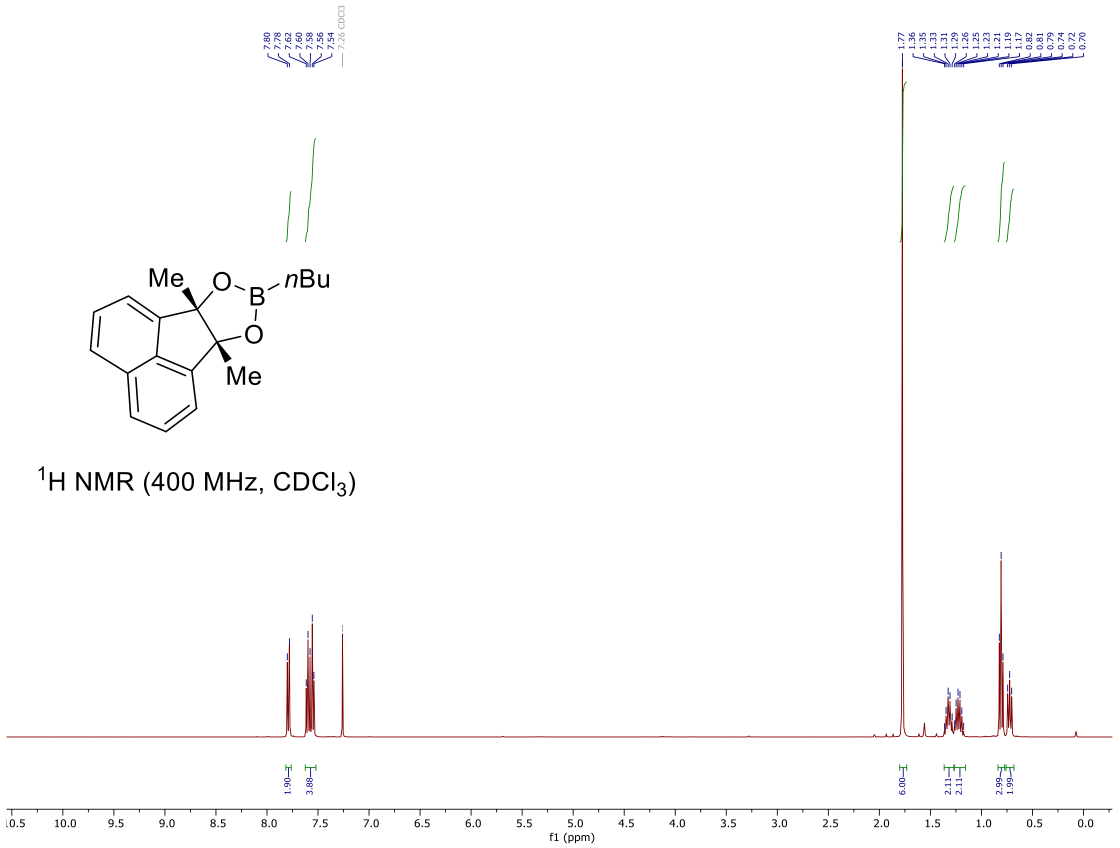

^{1}H NMR (400 MHz, $CDCl_3$)

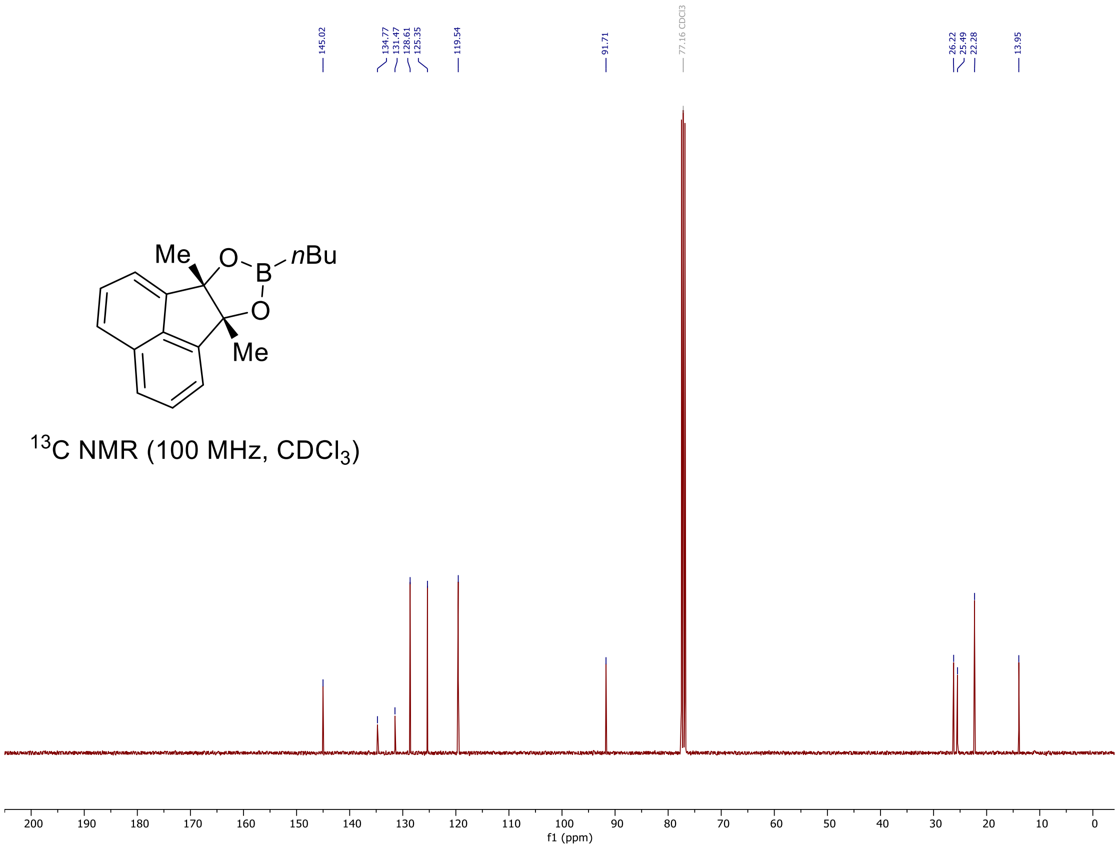

^{13}C NMR (100 MHz, $CDCl_3$)

2-Butyl-4,4,5,5-tetraphenyl-1,3,2-dioxaborolane (Si-9)

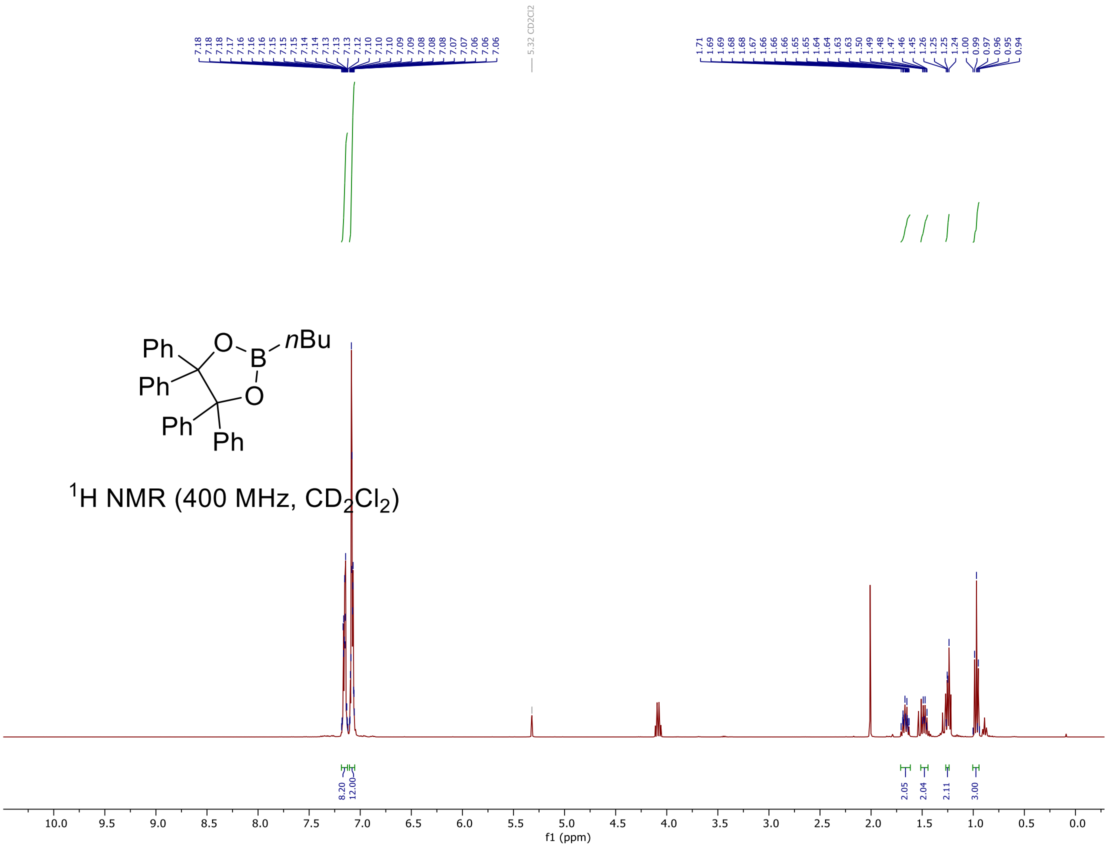

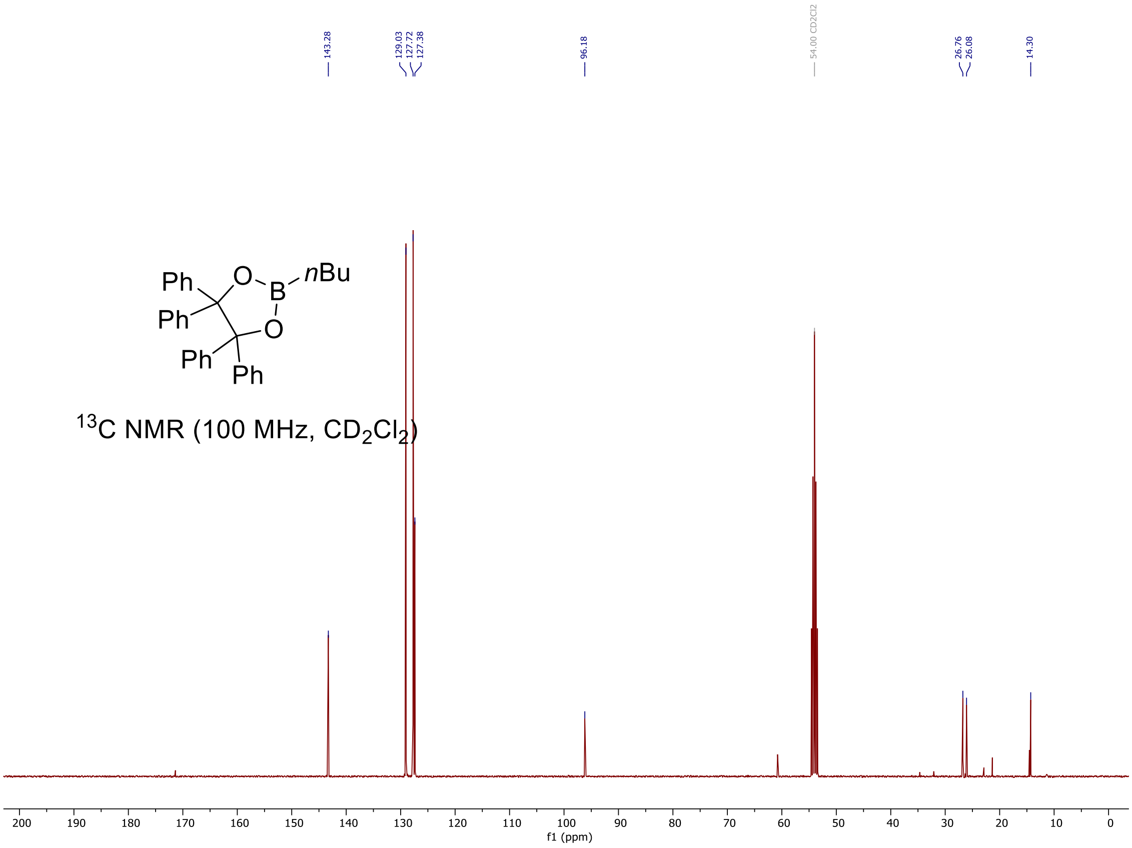

2-Butyl-4,4,5,5-tetraisopropyl-1,3,2-dioxaborolane (<u>Si-10</u>)

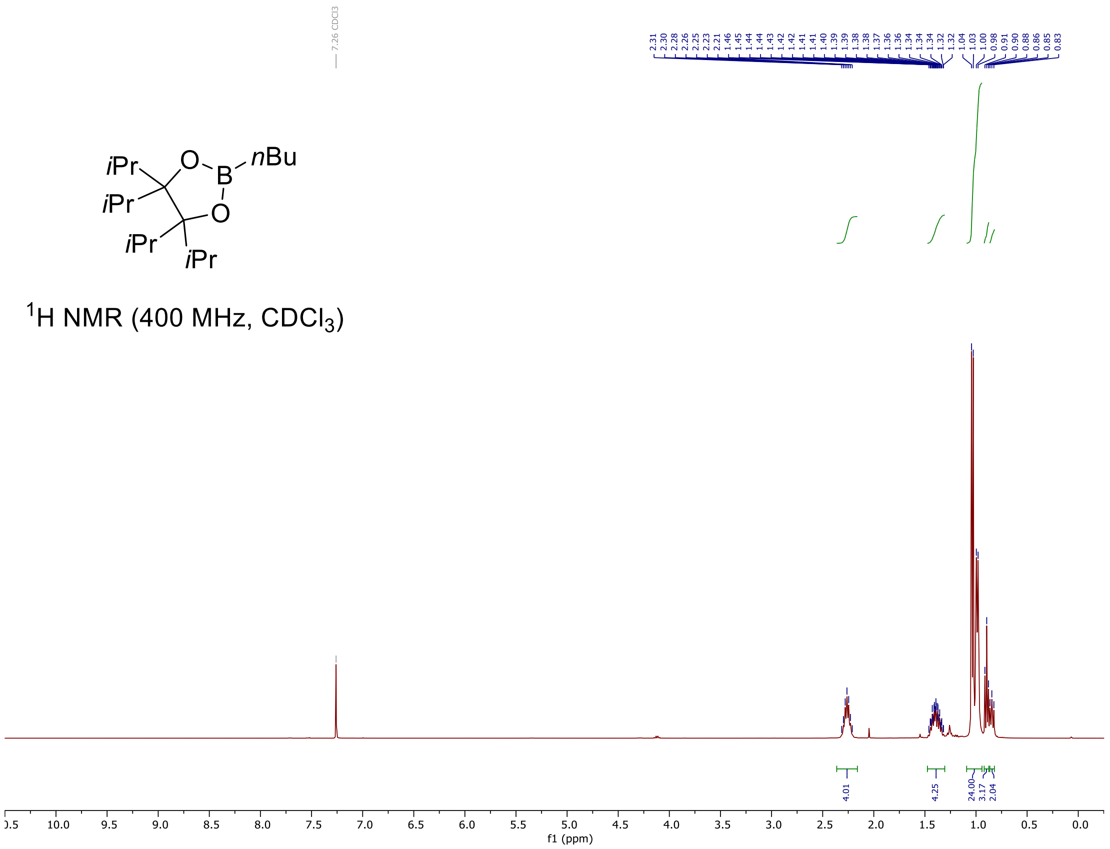

^{1}H NMR (400 MHz, $CDCl_3$)

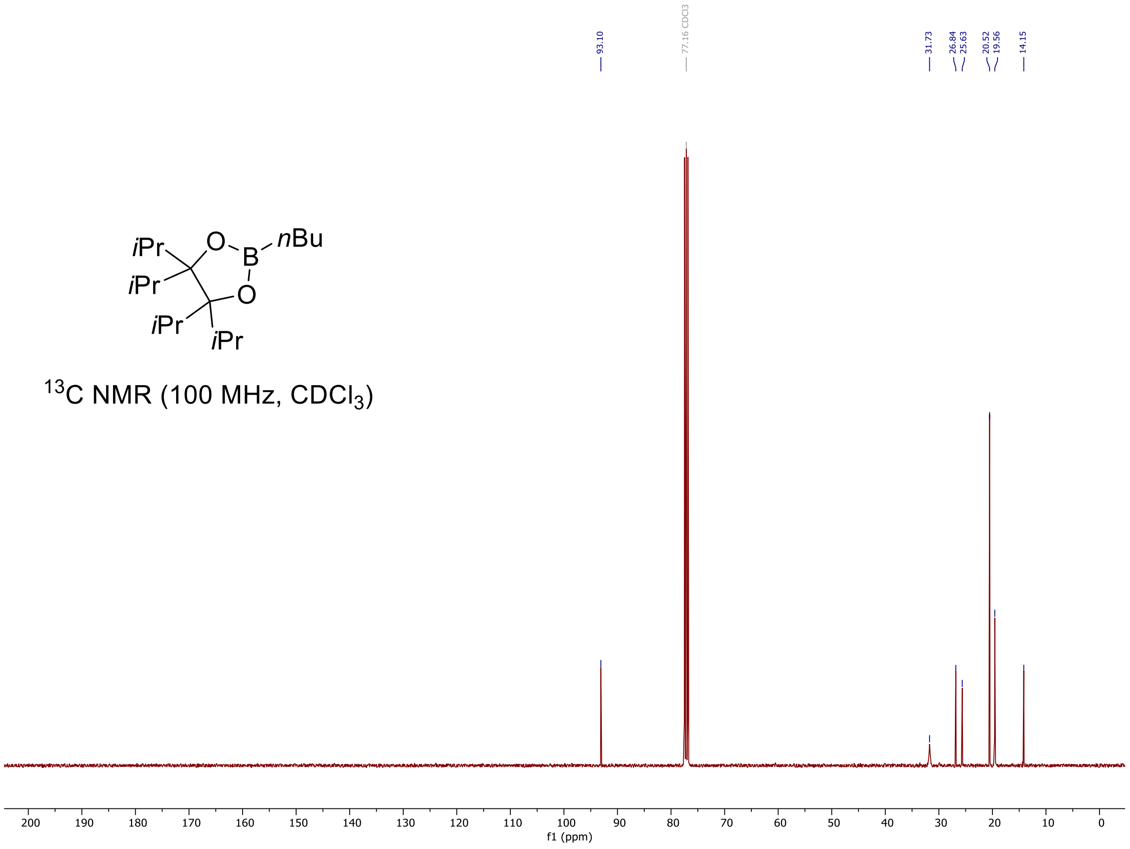

^{13}C NMR (100 MHz, $CDCl_3$)

2,4,4,5,5-Pentabutyl-1,3,2-dioxaborolane (Si-11)

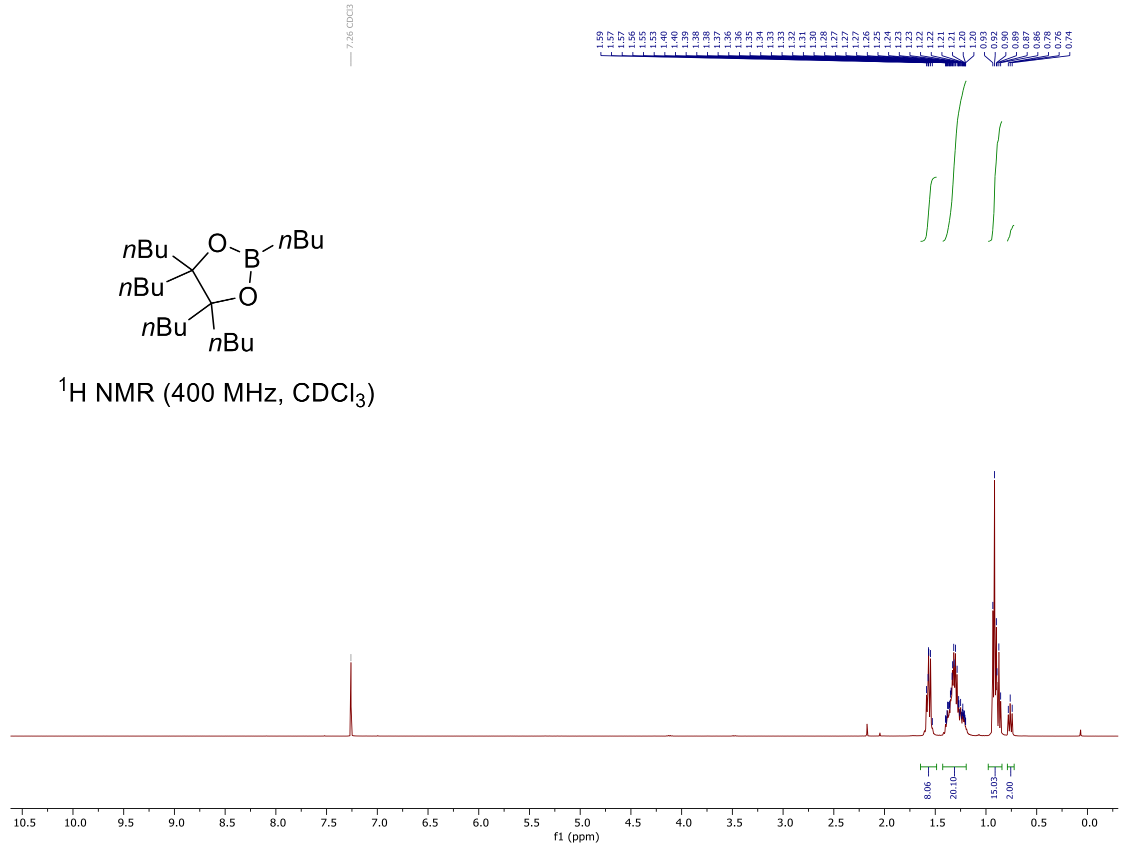

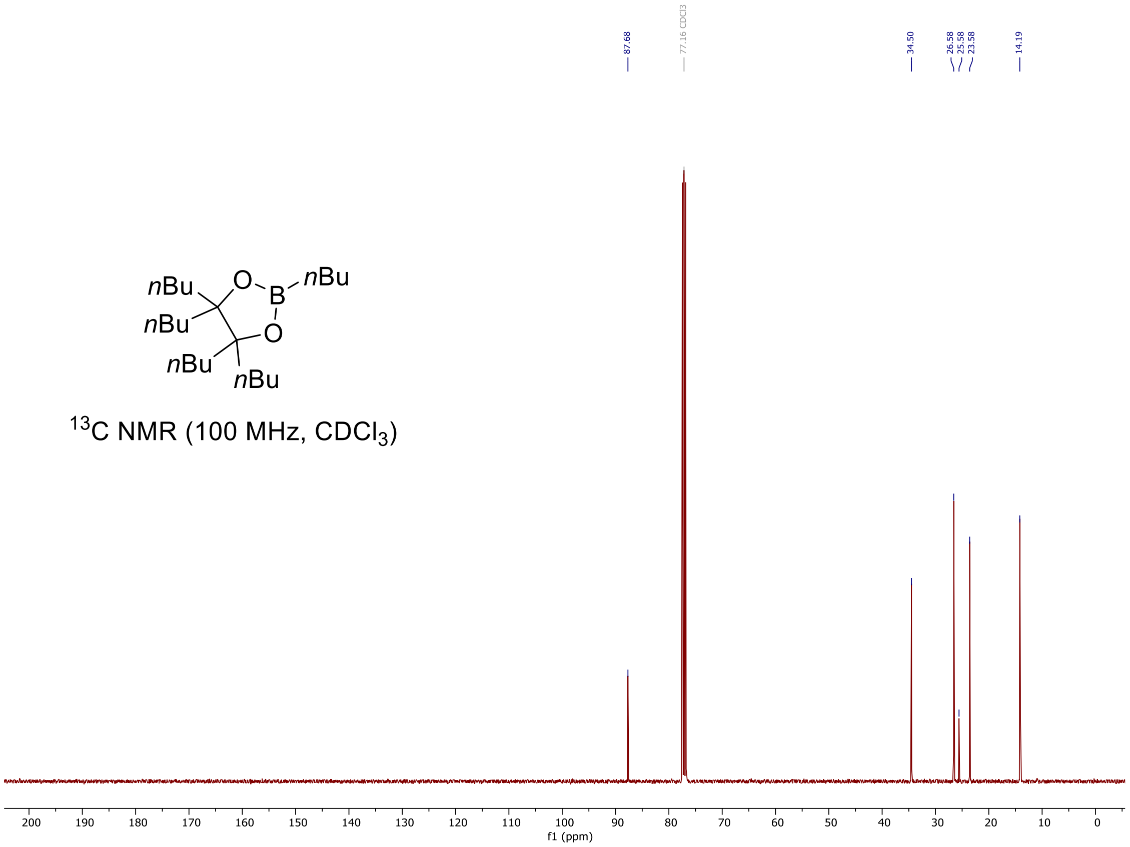

Butylboronic acid (1*S*,2*S*,3*R*,5*S*)-(+)-2,3-pinanediol ester (<u>Si-12</u>)

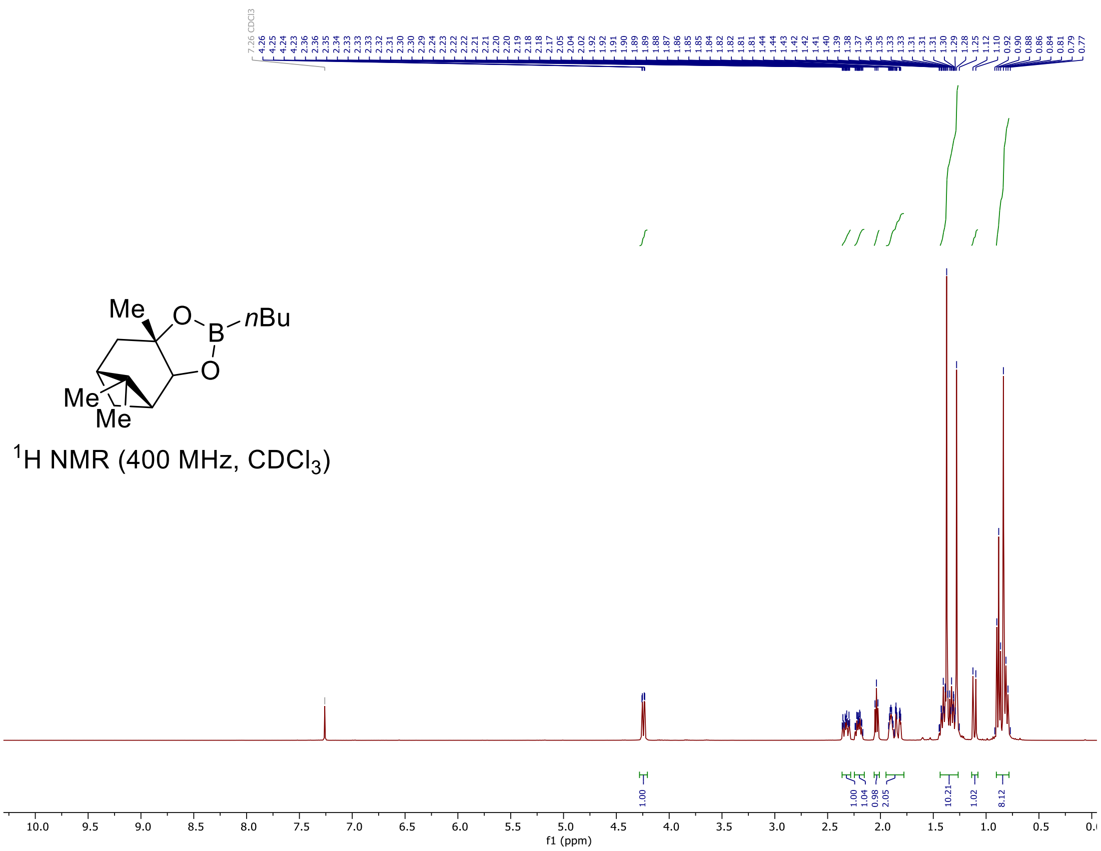

^{1}H NMR (400 MHz, $CDCl_3$)

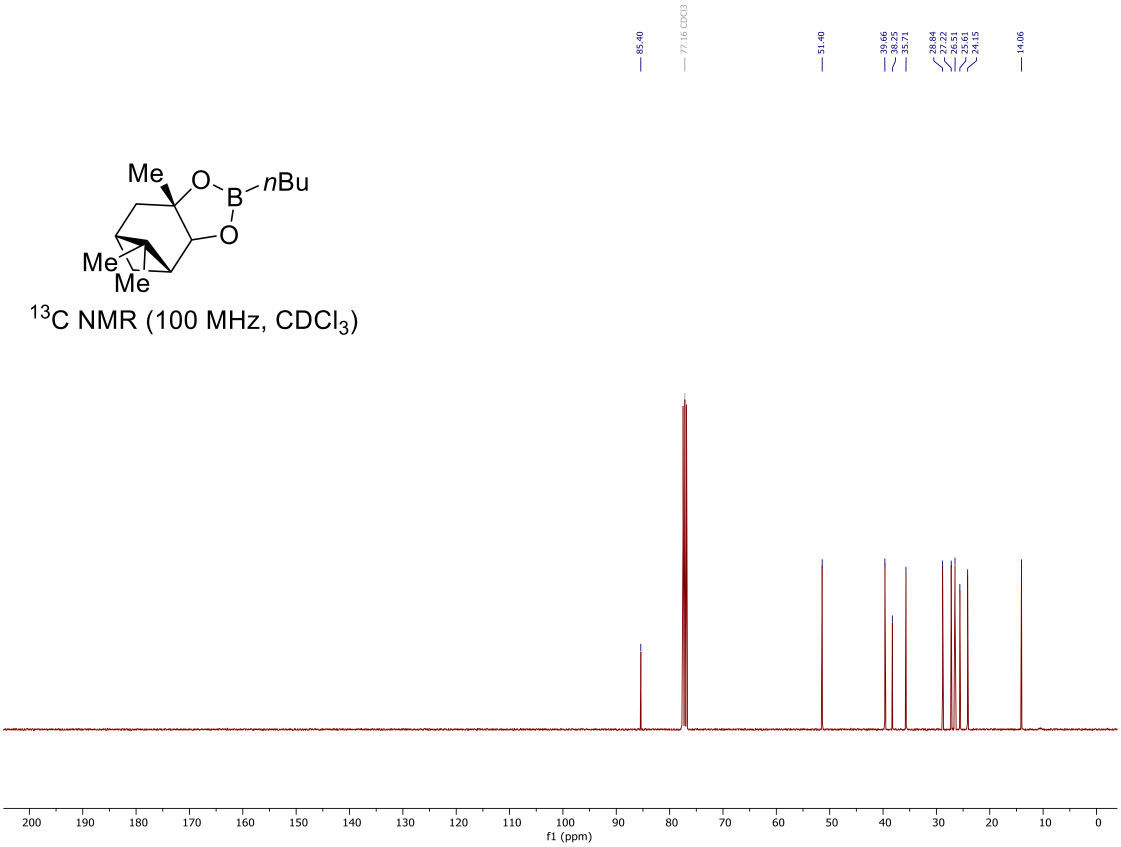

^{13}C NMR (100 MHz, $CDCl_3$)

Ritter's trifluoroiodomethane-DMSO complex (Si-13)

CF_3I • 2DMSO

^{1}H NMR (600 MHz, $CDCl_3$)

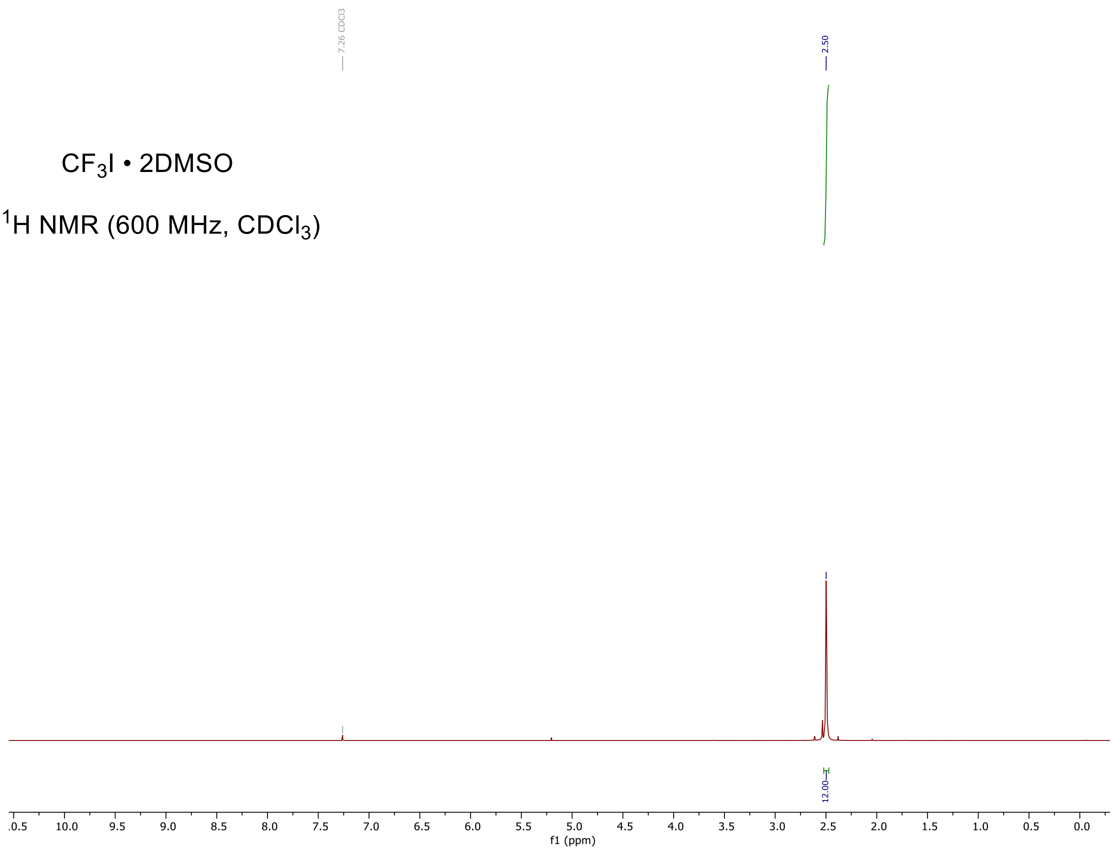

CF_3I • 2DMSO

^{13}C NMR (150 MHz, $CDCl_3$)

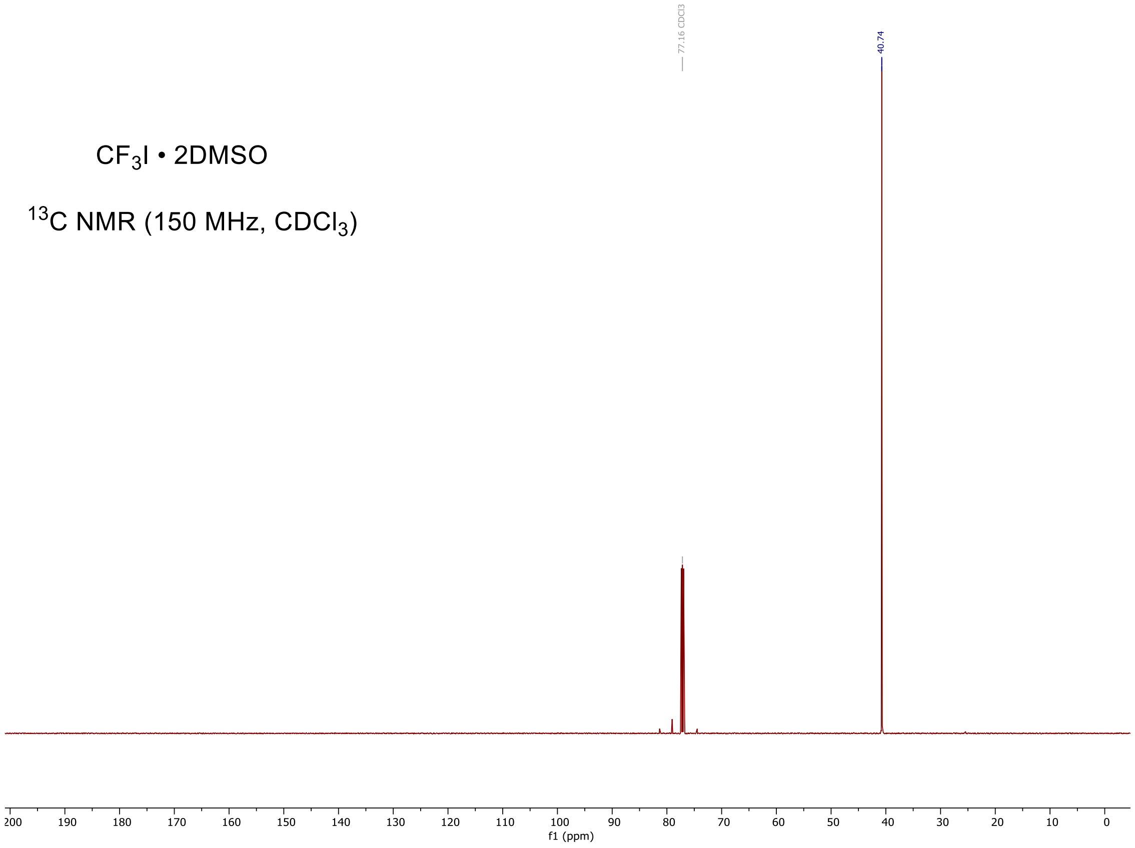

CF_3I • 2DMSO

^{19}F NMR (400 MHz, $CDCl_3$)

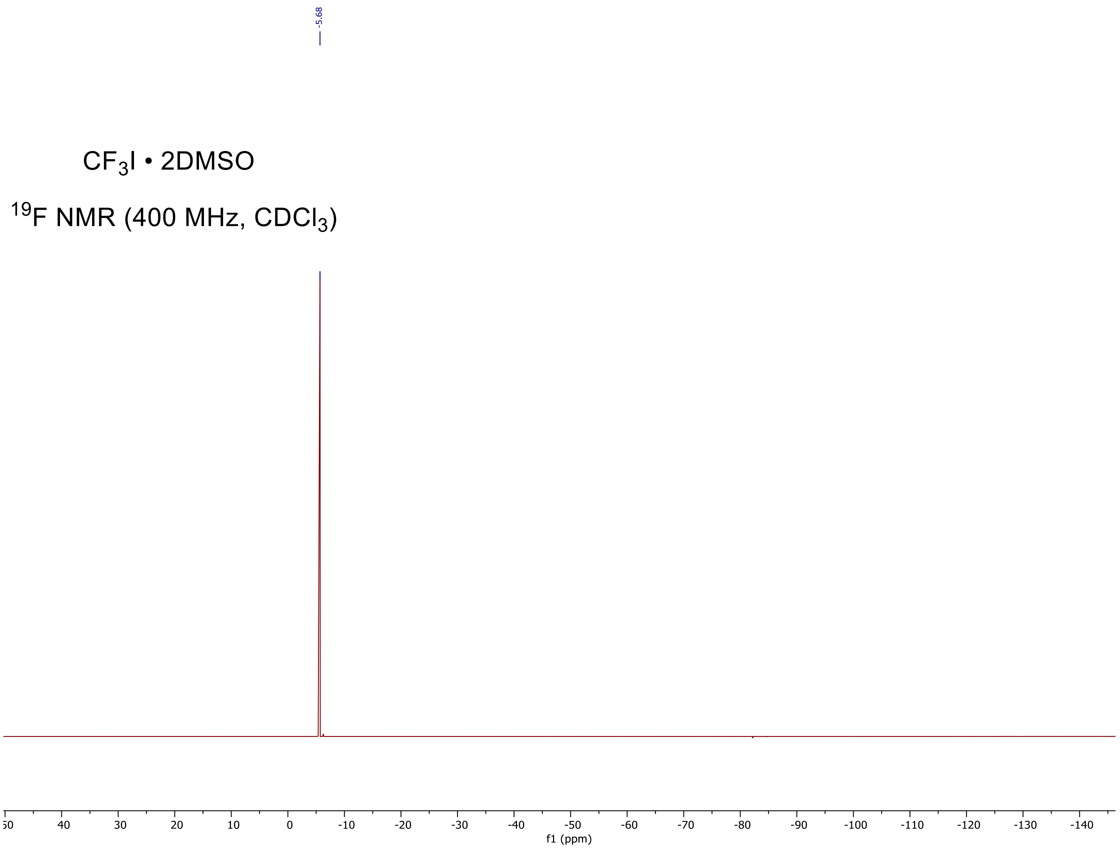

2-Iodo-1-phenylethan-1-one (Si-14)

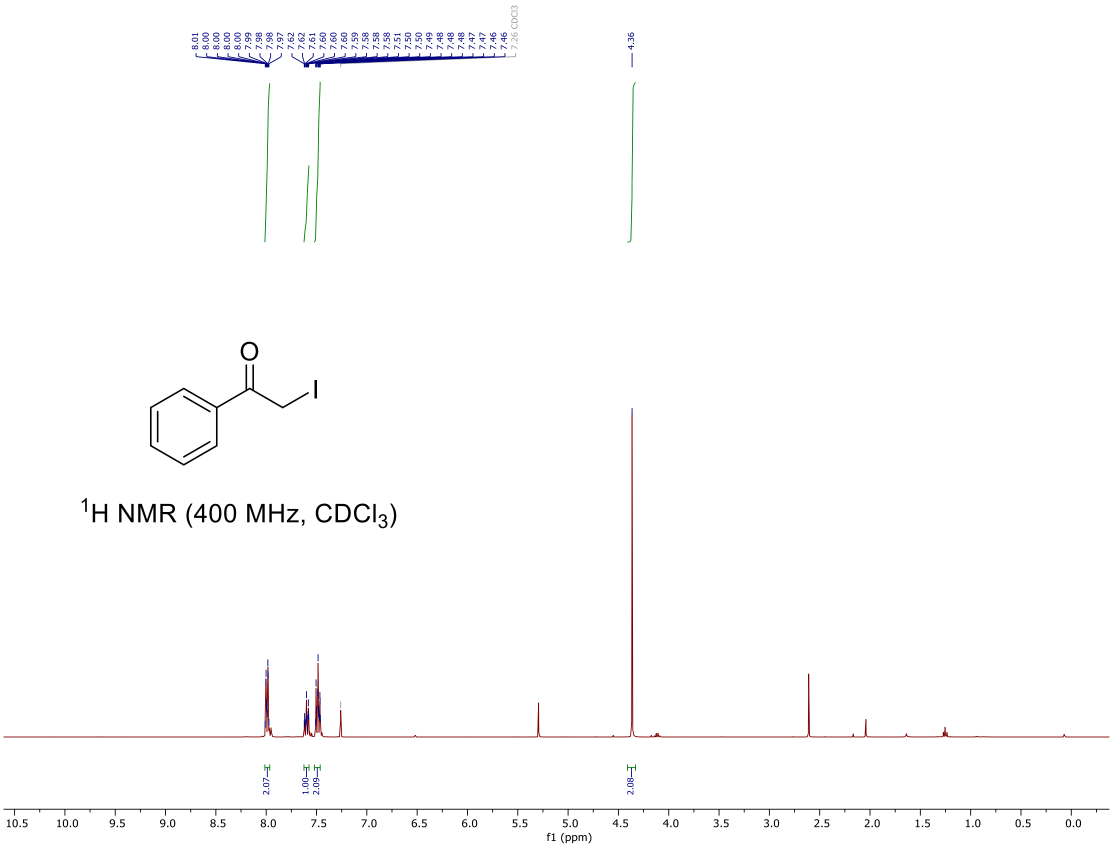

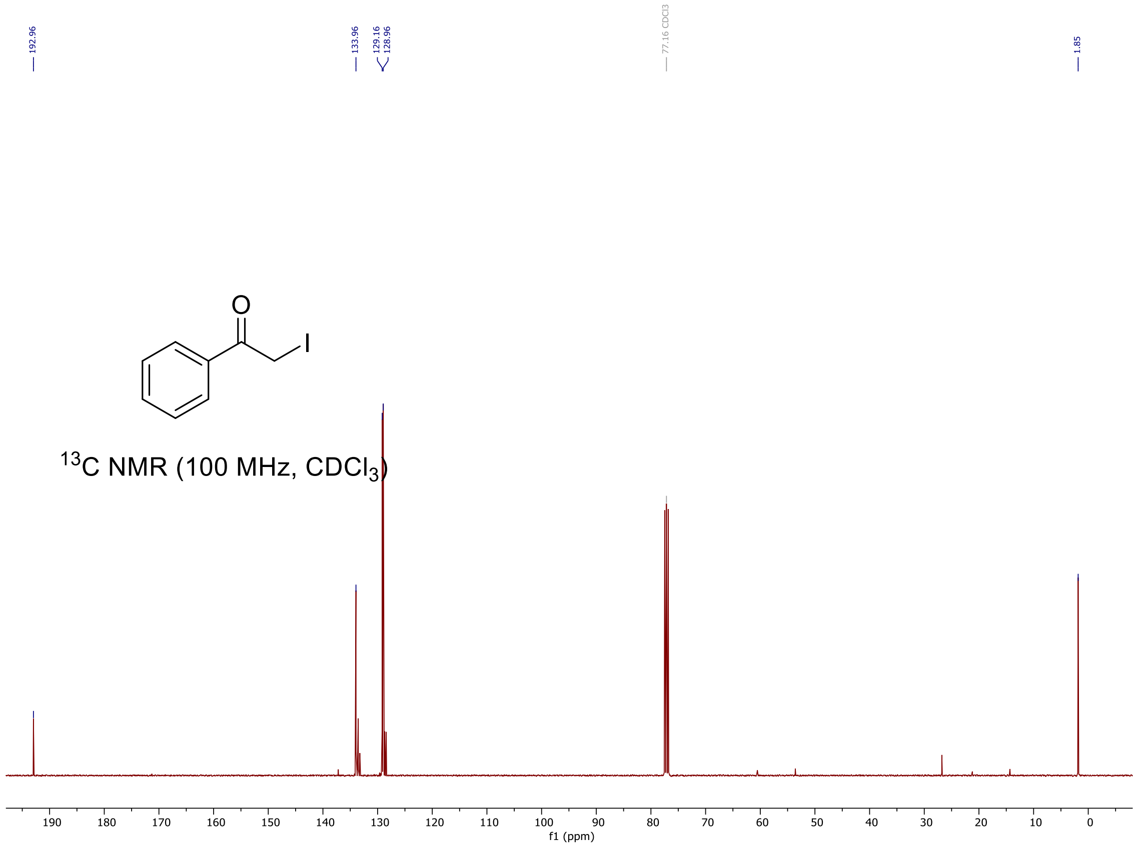

4,4,5,5-Tetraethyl-2-methoxy-1,3,2-dioxaborolane (Si-15)

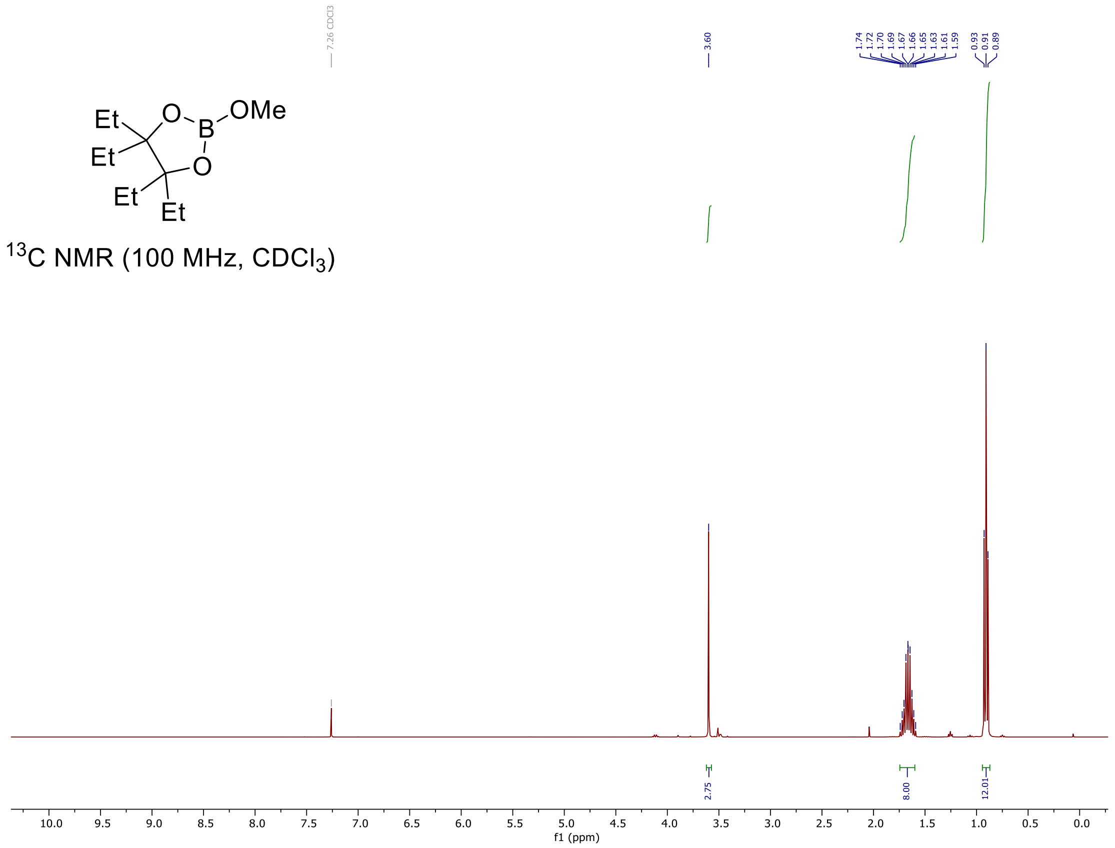

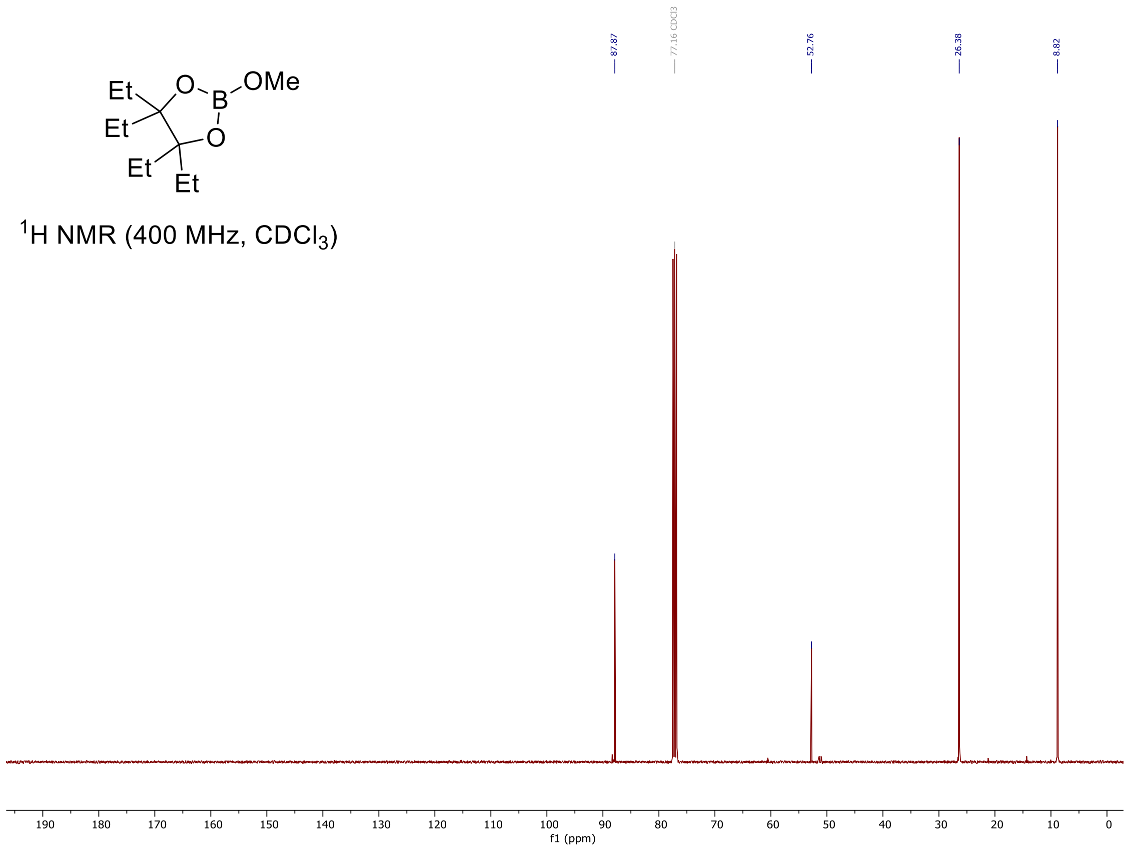

2-(2-Cyclohexylethyl)-4,4,5,5-tetraethyl-1,3,2-dioxaborolane (<u>Si-16</u>)

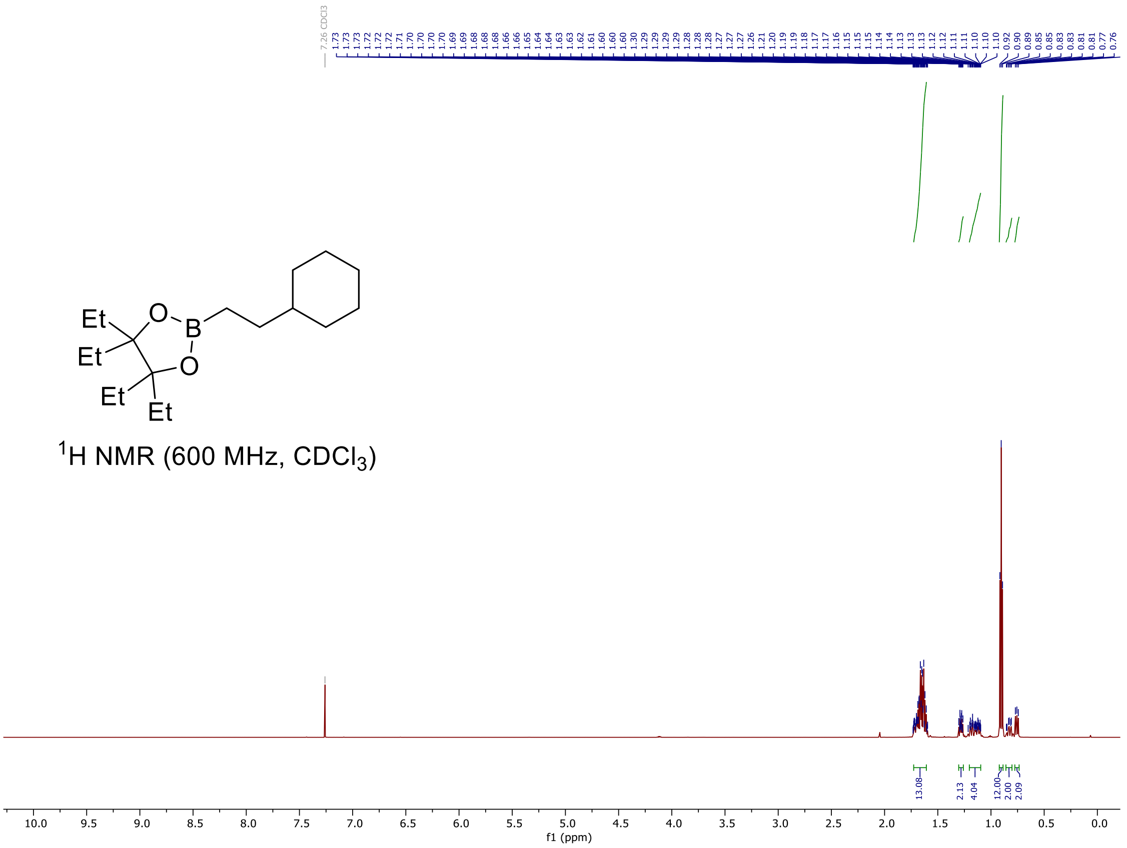

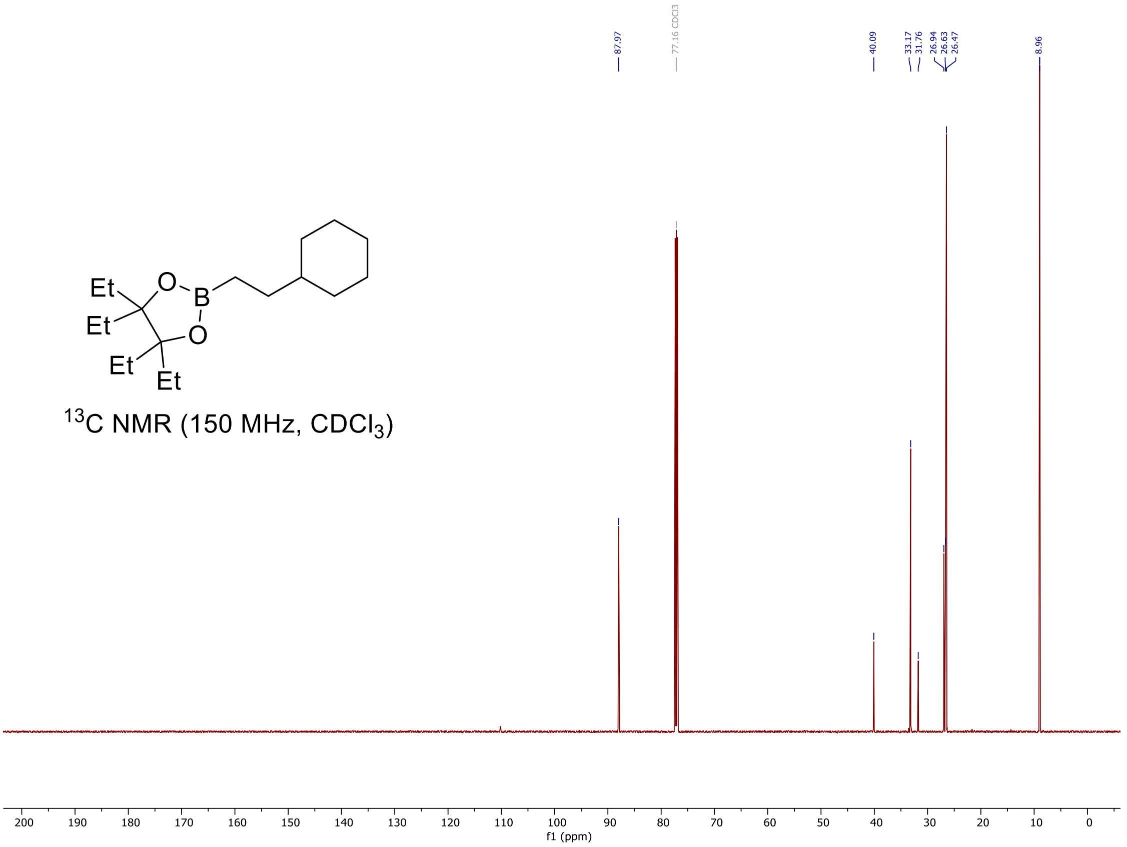

4,4,5,5-Tetraethyl-2-phenethyl-1,3,2-dioxaborolane (Si-17)

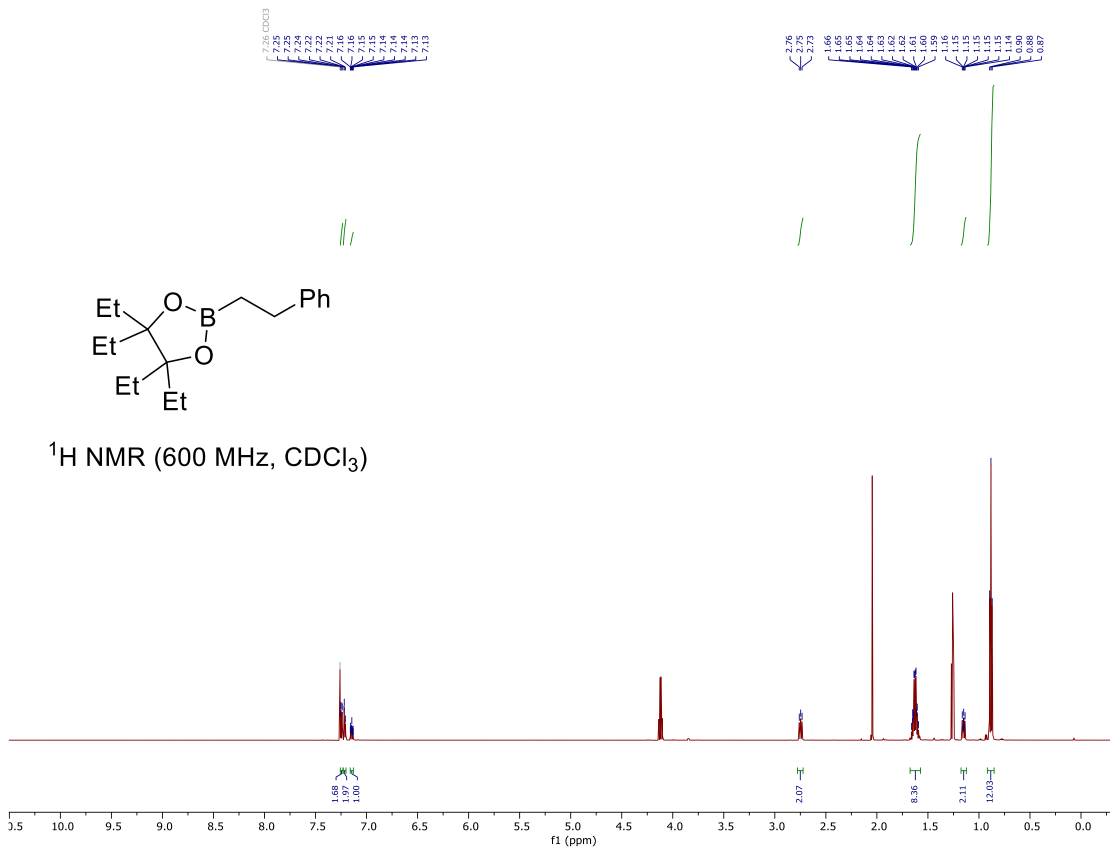

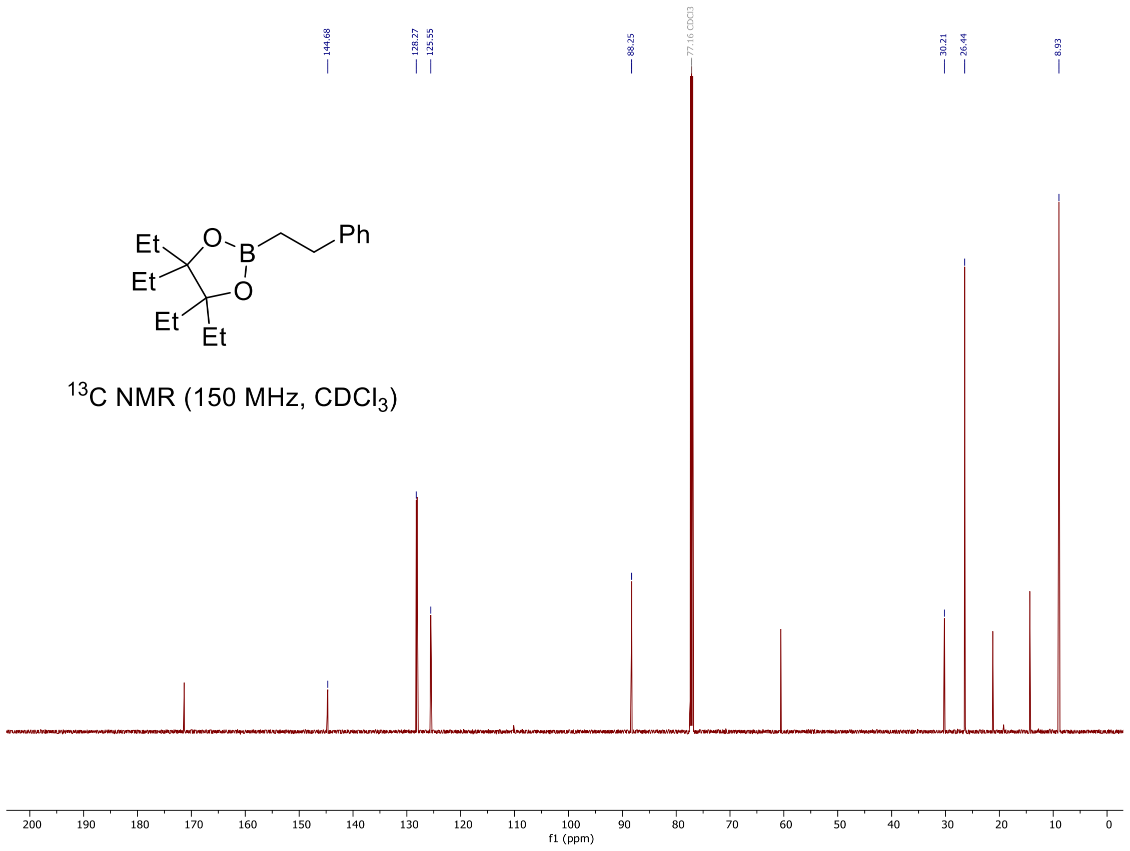

4,4,5,5-Tetraethyl-2-methyl-1,3,2-dioxaborolane (Si-18)

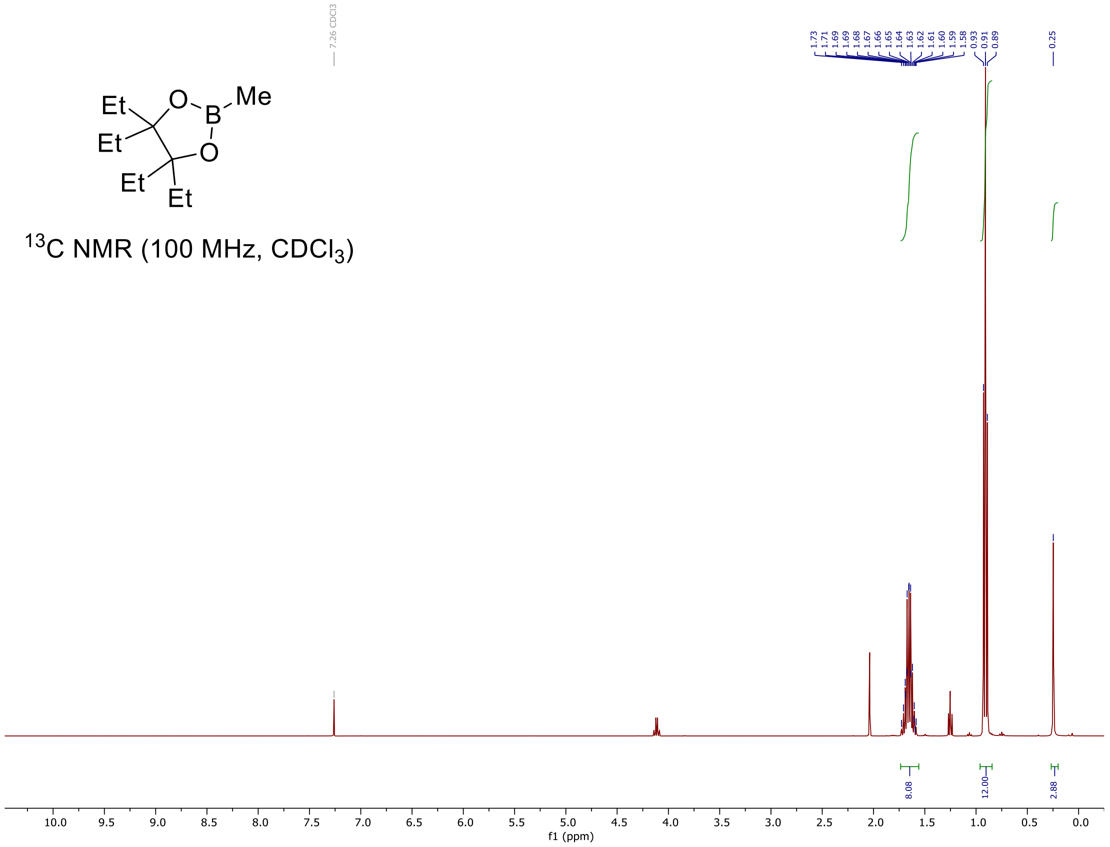

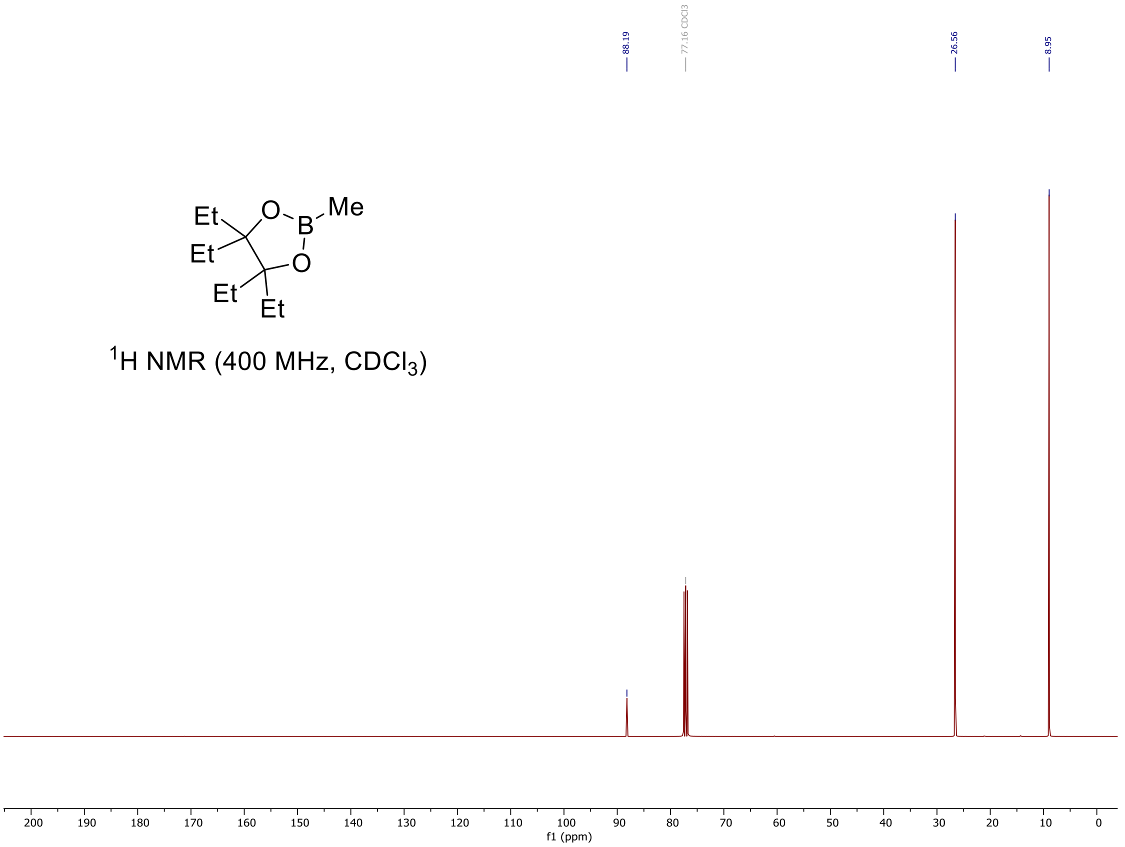

Trimethyl((4,4,5,5-tetraethyl-1,3,2-dioxaborolan-2-yl)methyl)silane (Si-19)

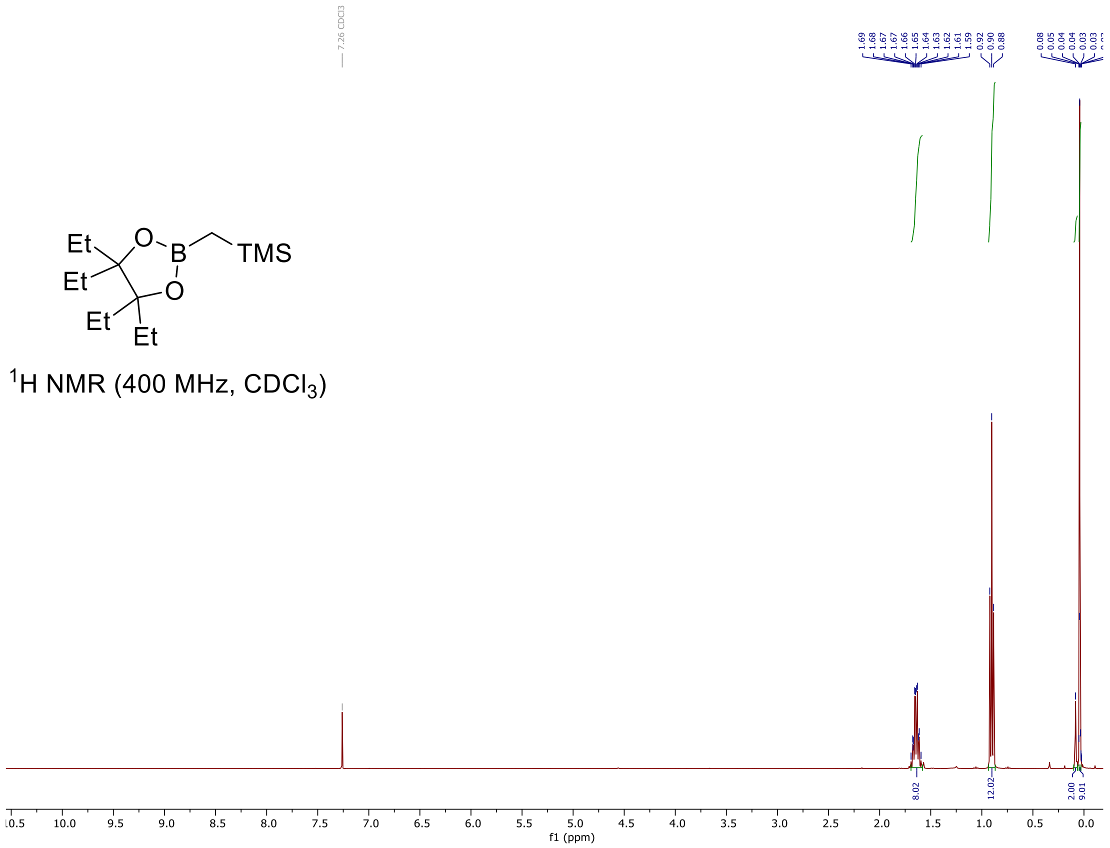

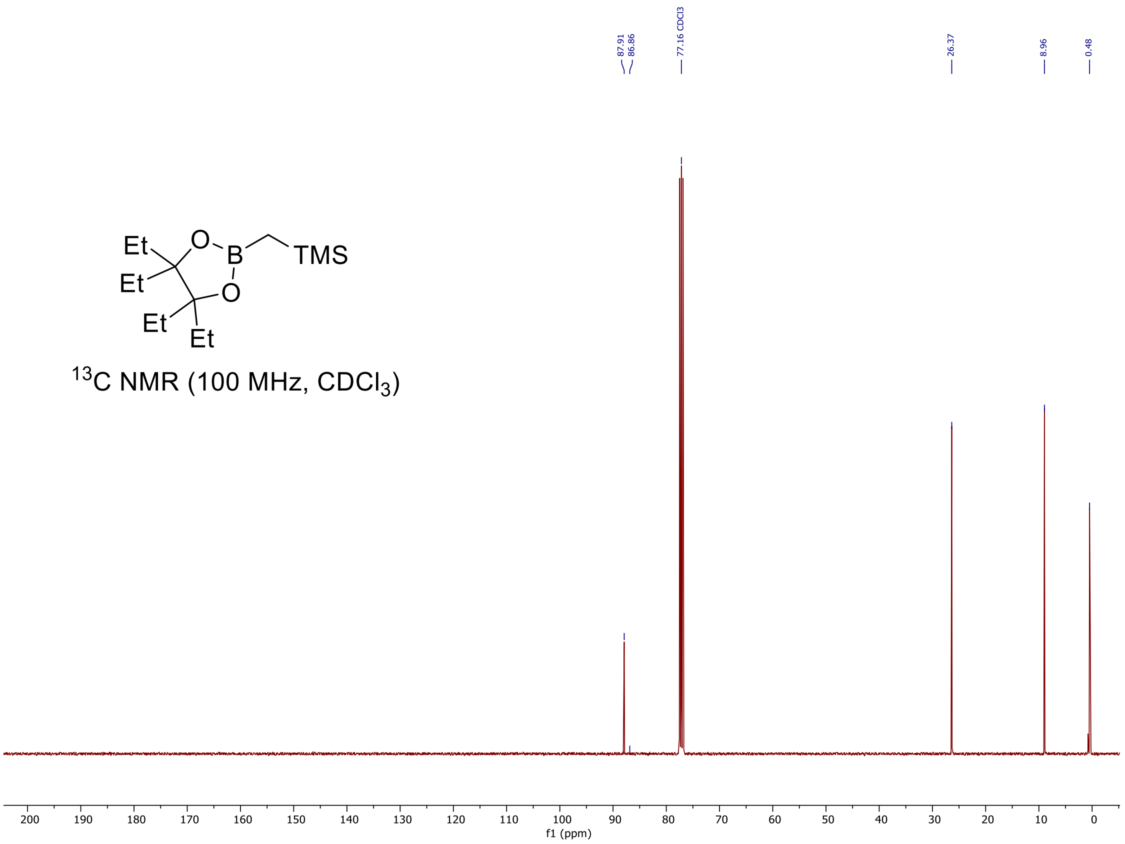

2-(4-chlorophenyl)-4,4,5,5-tetraethyl-1,3,2-dioxaborolane (<u>Si-20</u>)

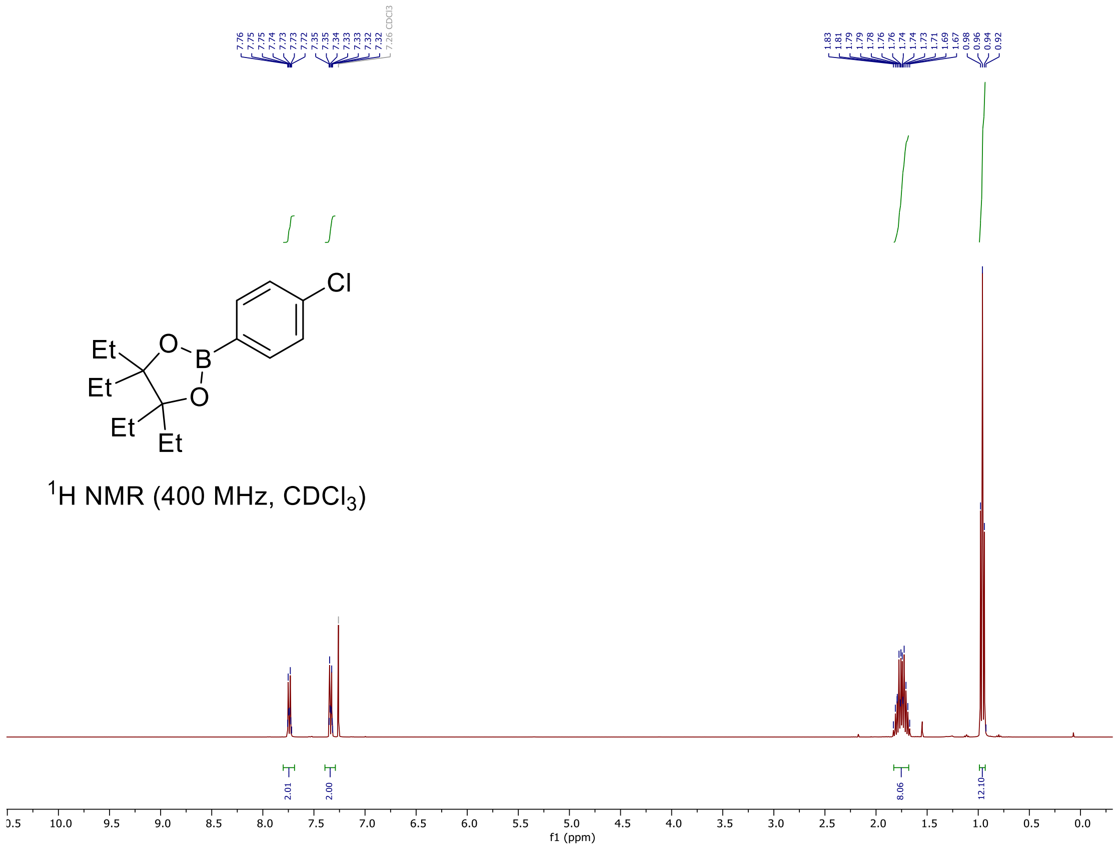

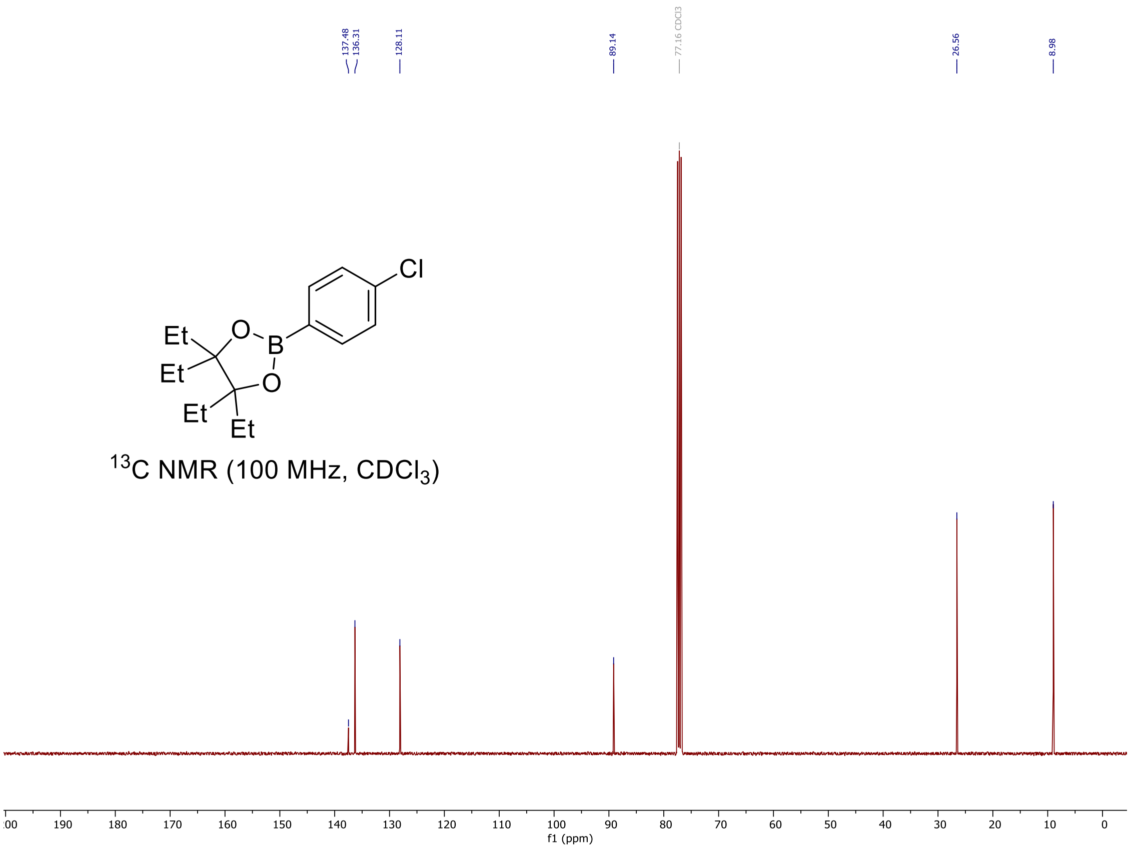

4,4,5,5-Tetraethyl-2-(4-fluorophenethyl)-1,3,2-dioxaborolane (Si-21)

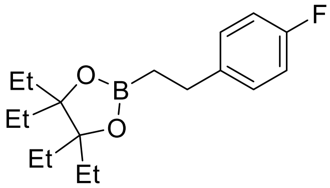

^{1}H NMR (400 MHz, $CDCl_3$)

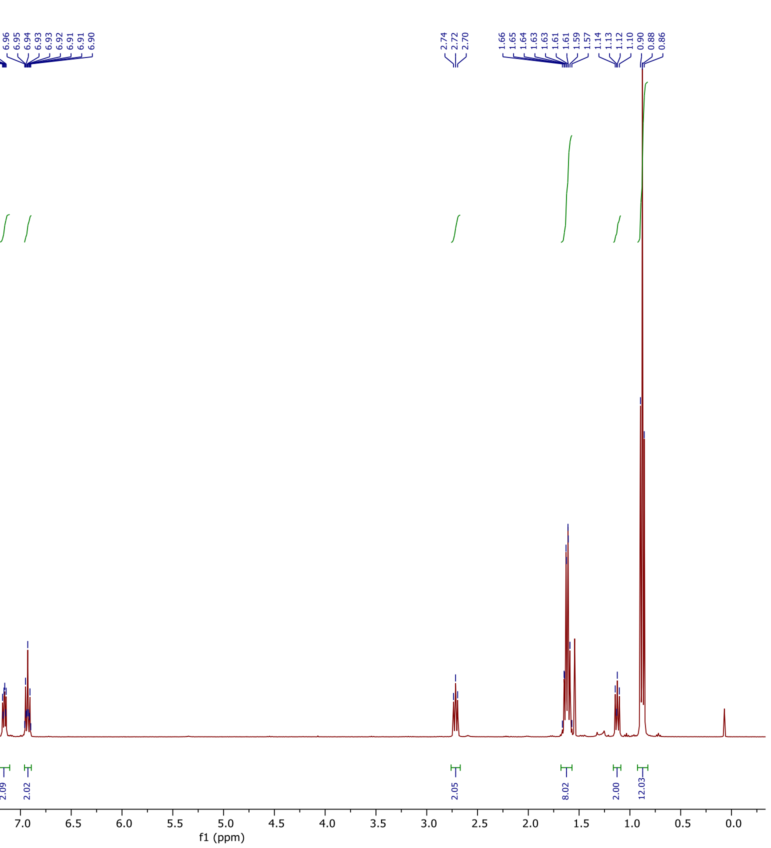

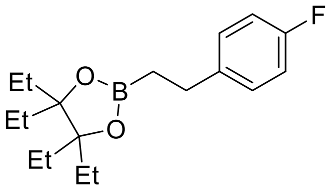

^{13}C NMR (100 MHz, $CDCl_3$)

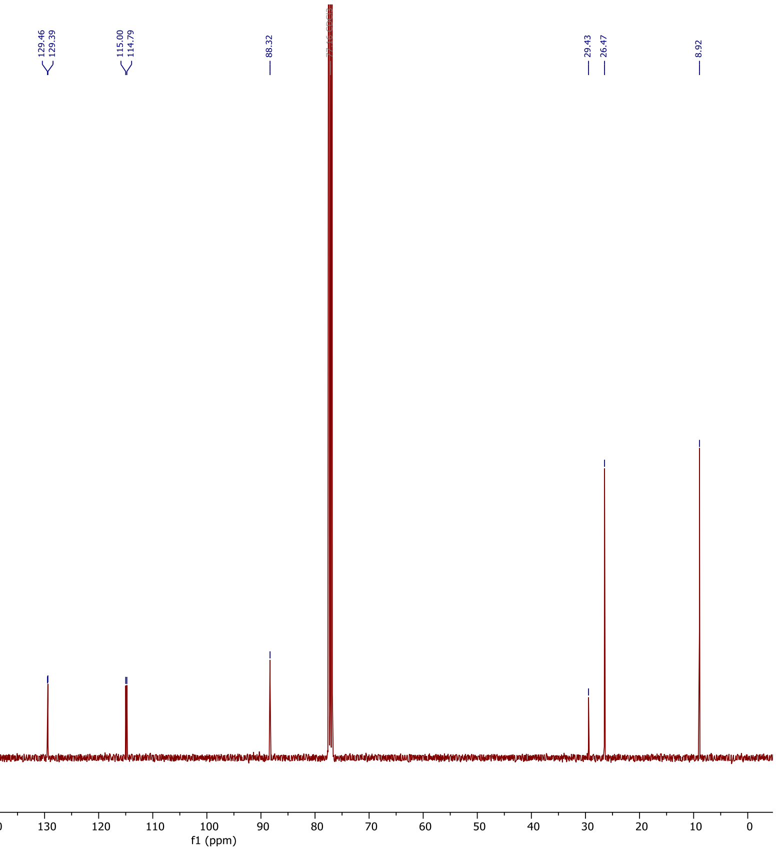

2-(Adamantan-1-yl)methyl)-4,4,5,5-tetraethyl-1,3,2-dioxaborolane (Si-22)

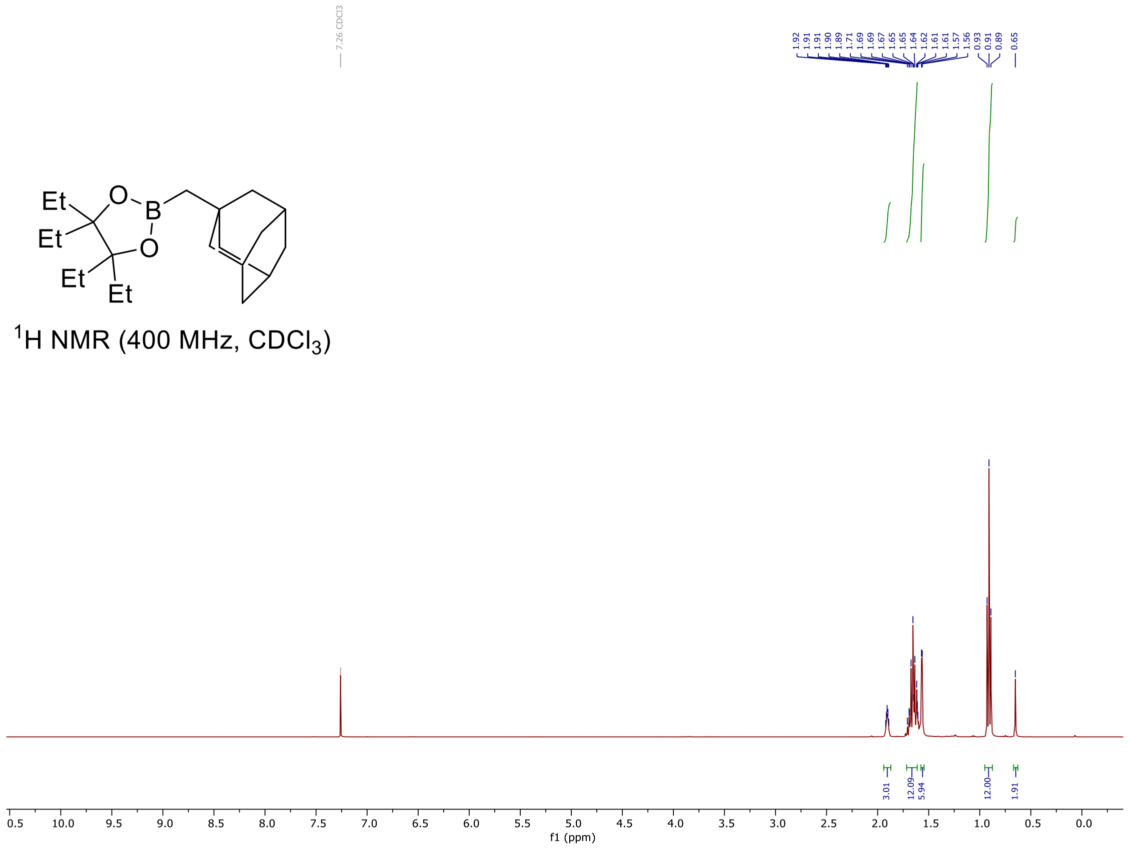

^{1}H NMR (400 MHz, $CDCl_3$)

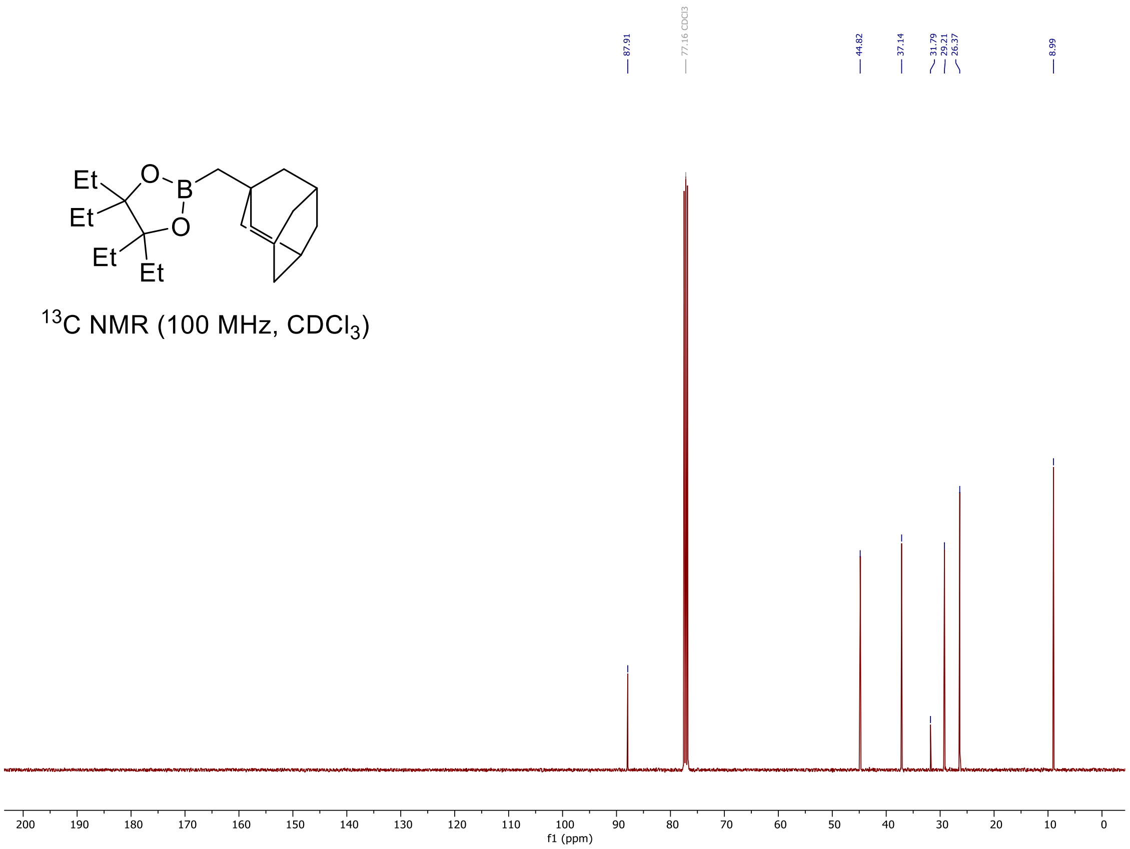

^{13}C NMR (100 MHz, $CDCl_3$)

4,4,5,5-Tetraethyl-2-neopentyl-1,3,2-dioxaborolane (Si-23)

^{1}H NMR (400 MHz, $CDCl_3$)

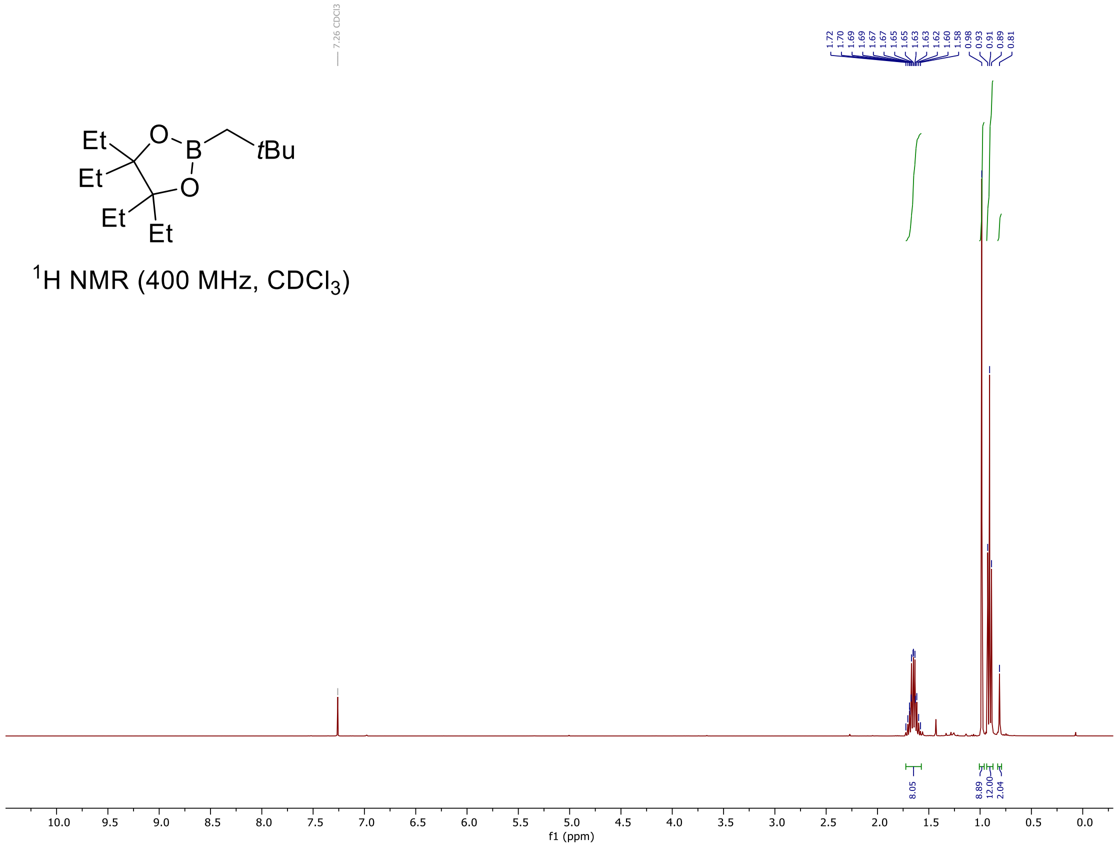

^{13}C NMR (100 MHz, $CDCl_3$)

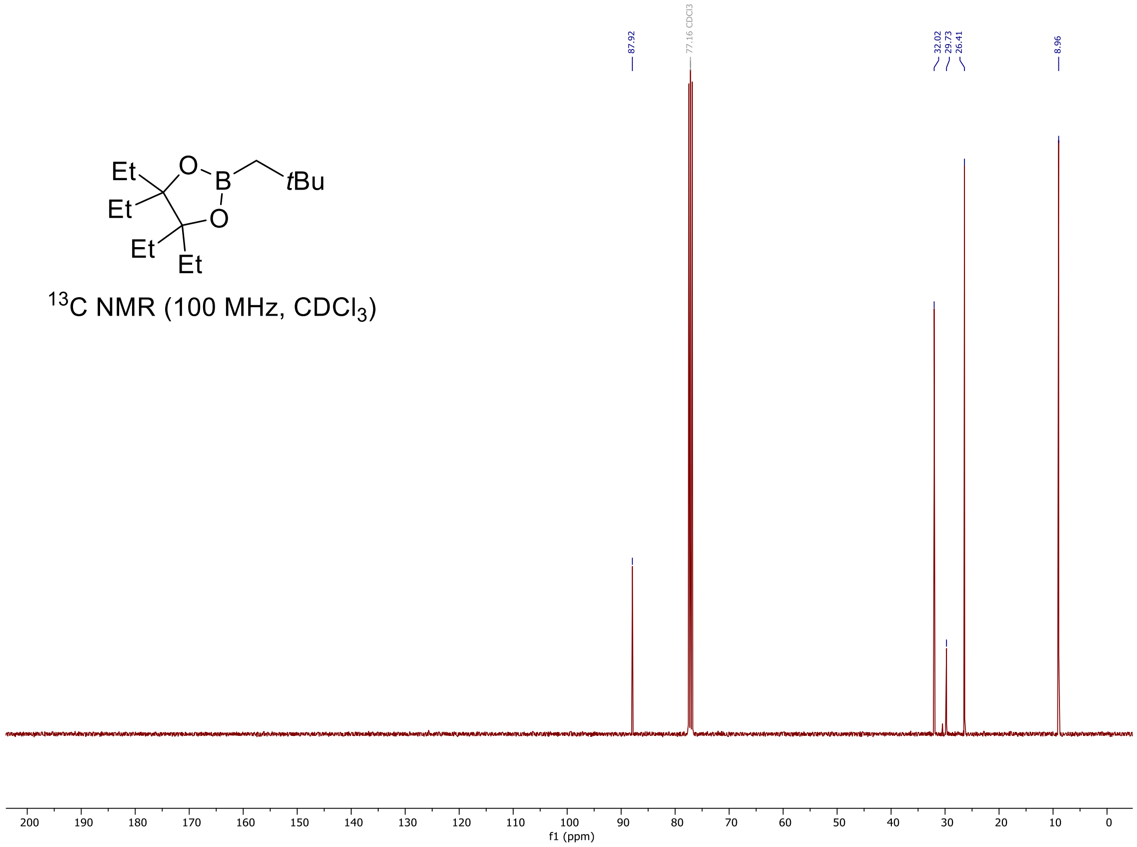

2-(3-(Adamantan-1-yl)-4-methoxyphenyl)-4,4,5,5-tetraethyl-1,3,2-dioxaborolane (Si-24)

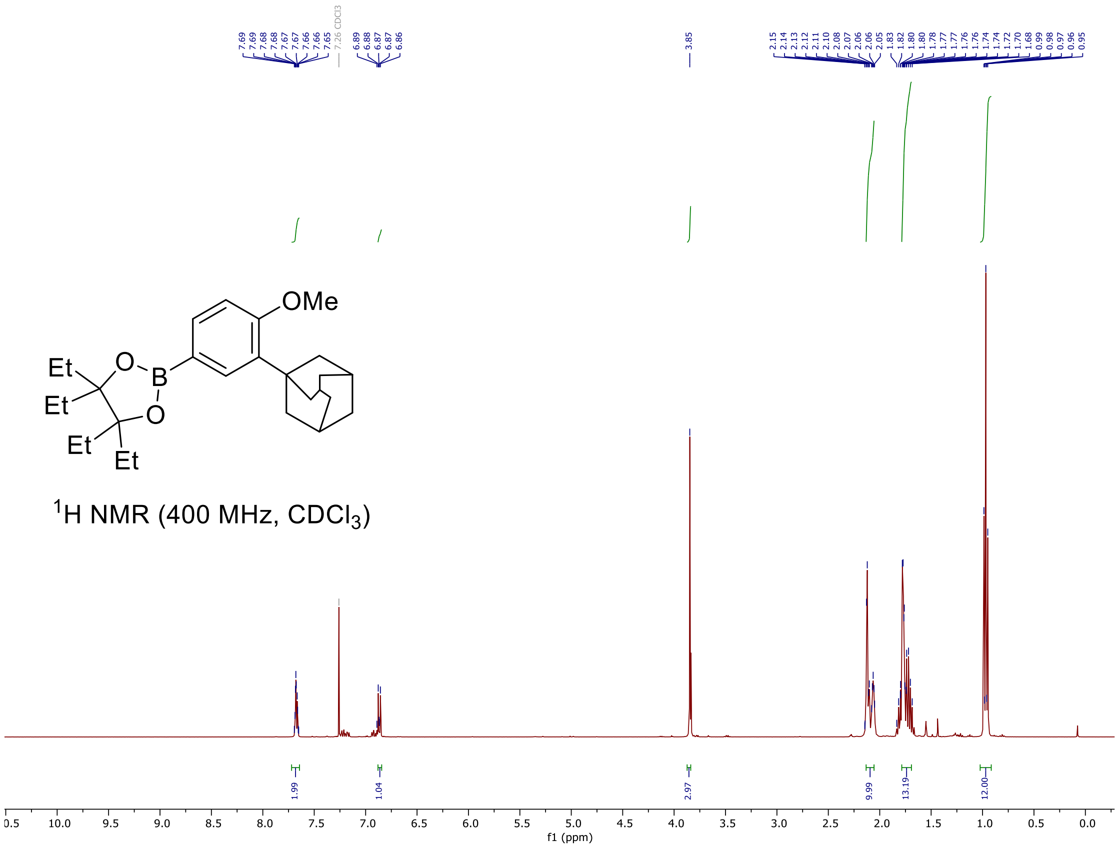

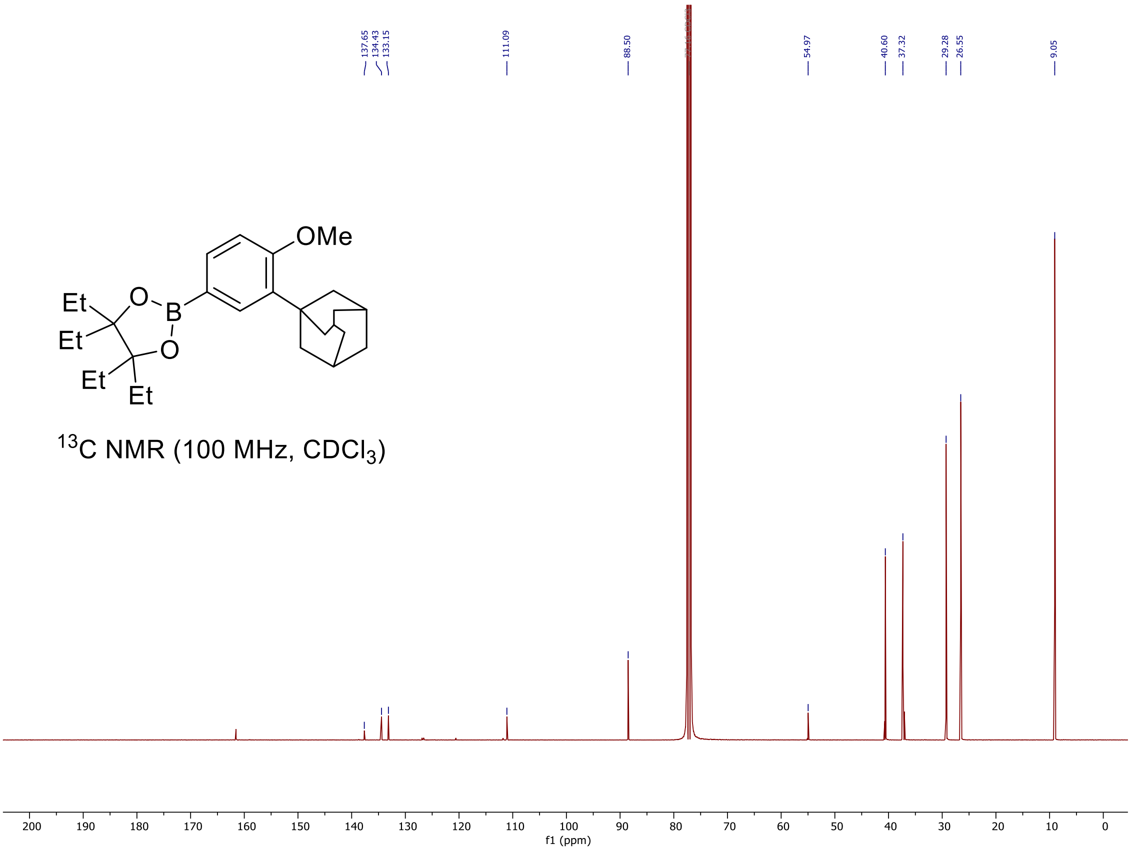

2-(Cyclohexylmethyl)-4,4,5,5-tetraethyl-1,3,2-dioxaborolane (Si-25)

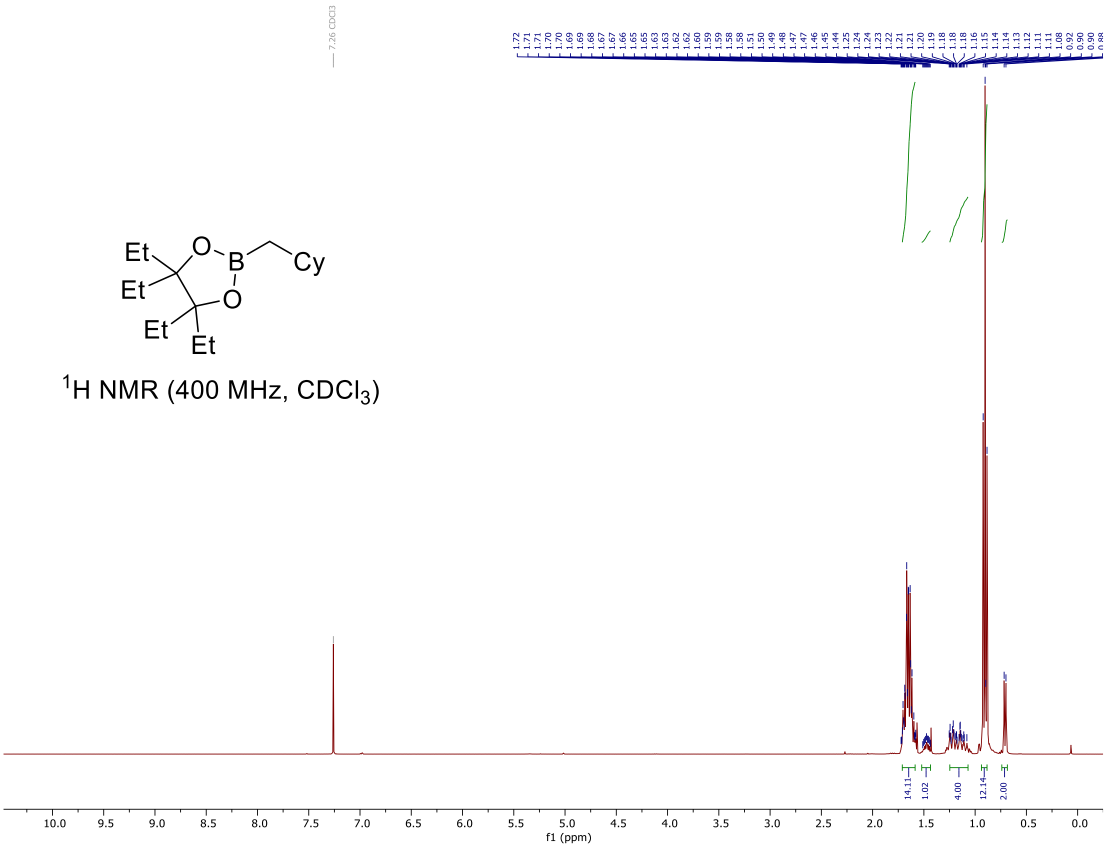

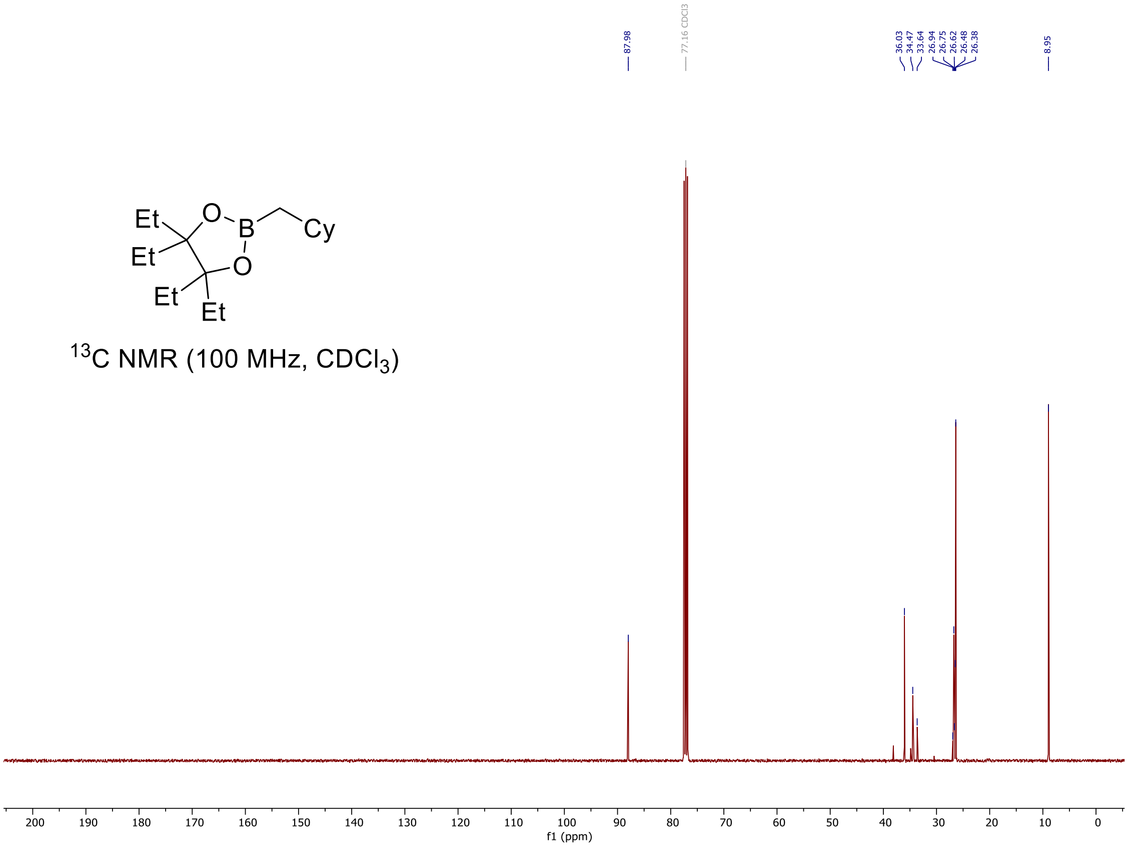

4,4,5,5-Tetraethyl-2-isobutyl-1,3,2-dioxaborolane (<u>Si-26</u>)

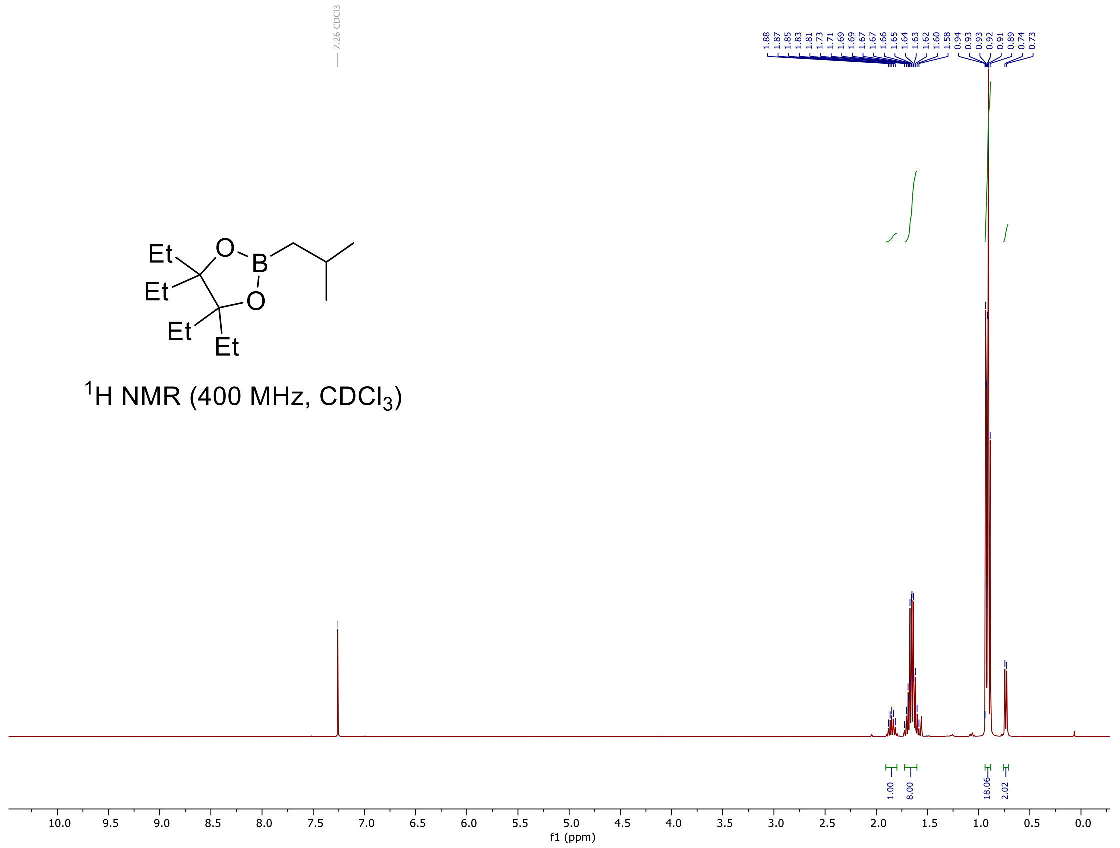

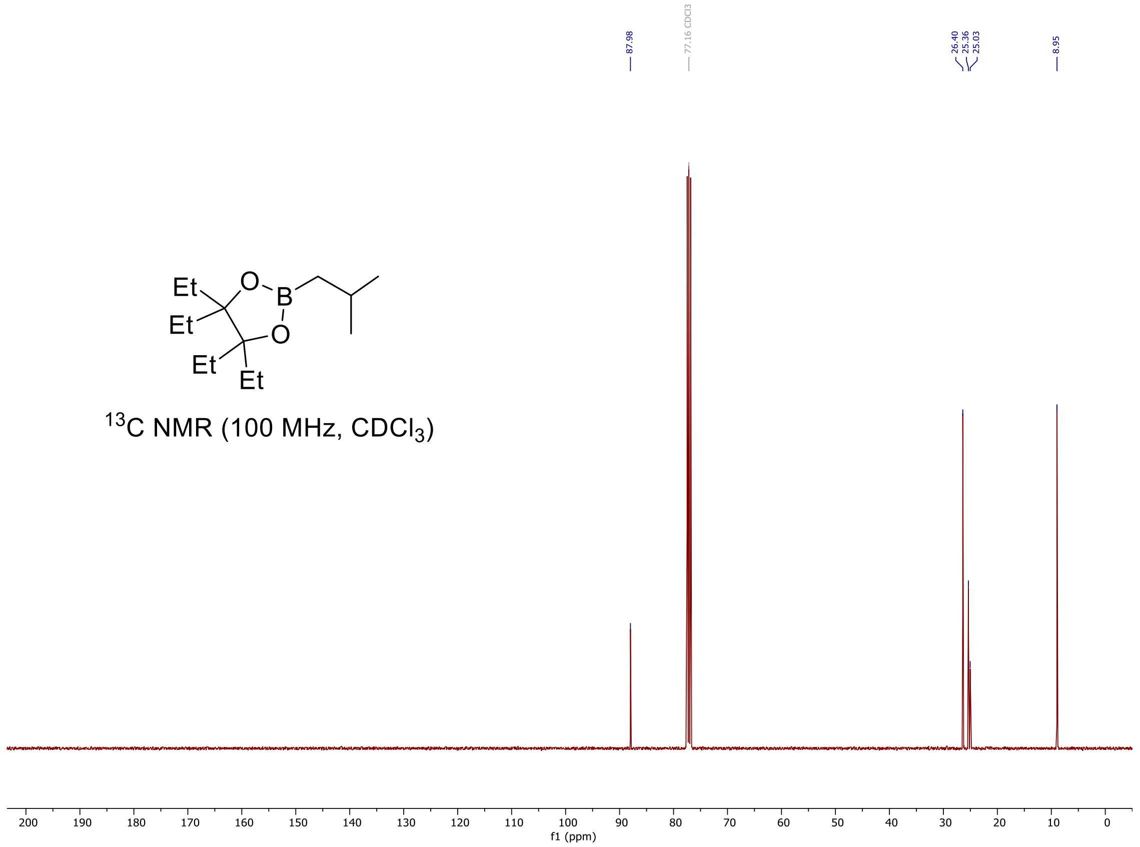

2-(Cyclobut-1-en-1-yl)-4,4,5,5-tetraethyl-1,3,2-dioxaborolane (4)

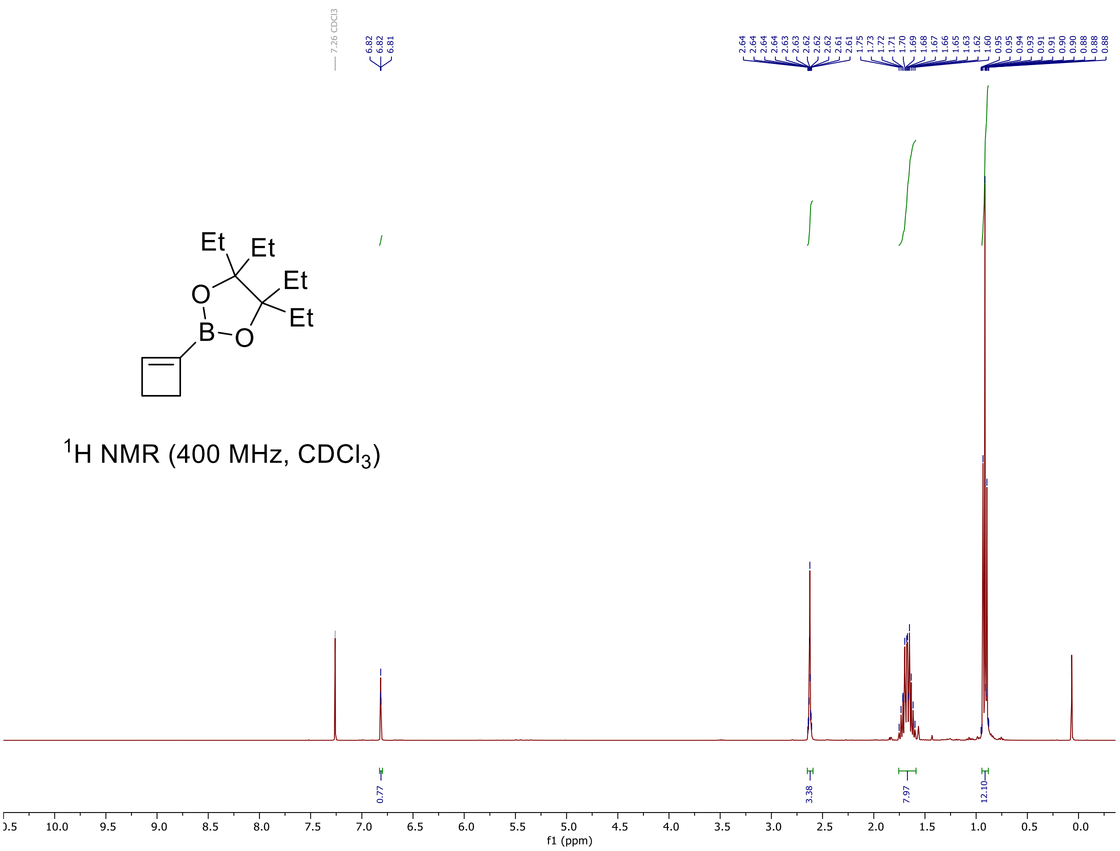

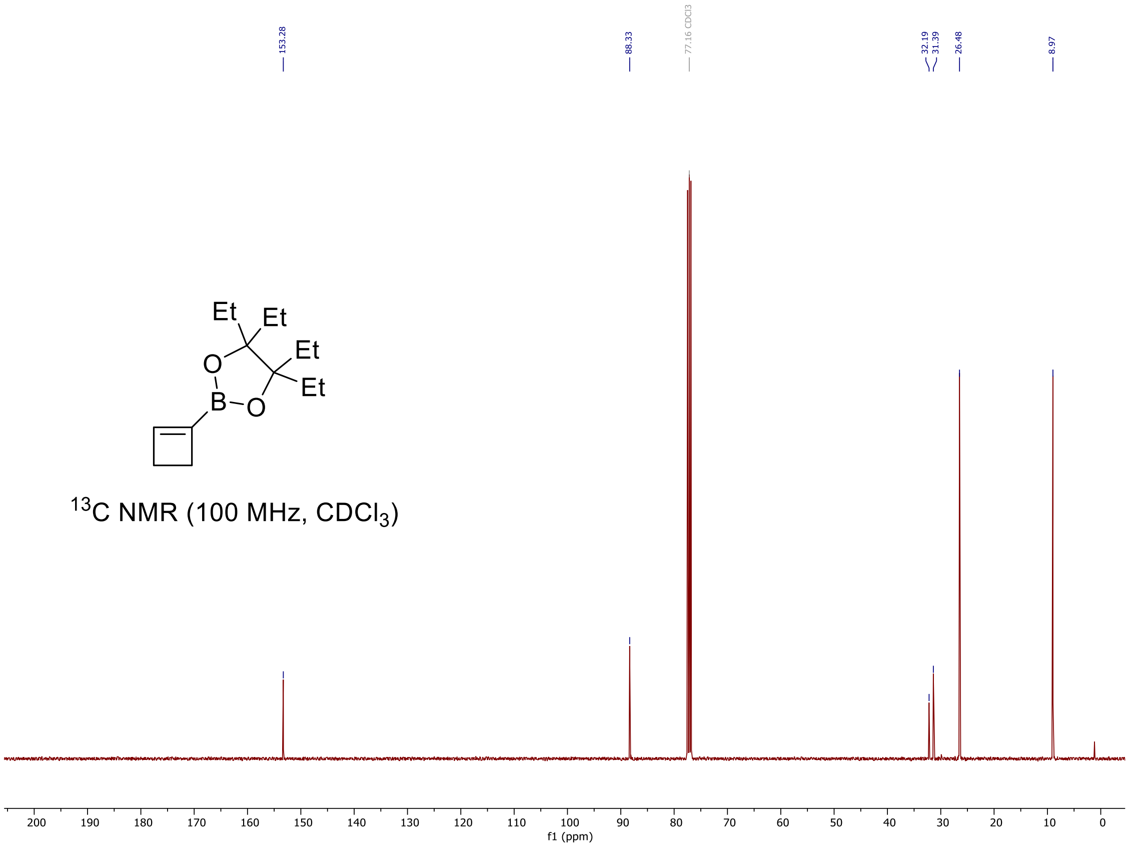

2-(cyclopent-1-en-1-yl)-4,4,5,5-tetraethyl-1,3,2-dioxaborolane (7)

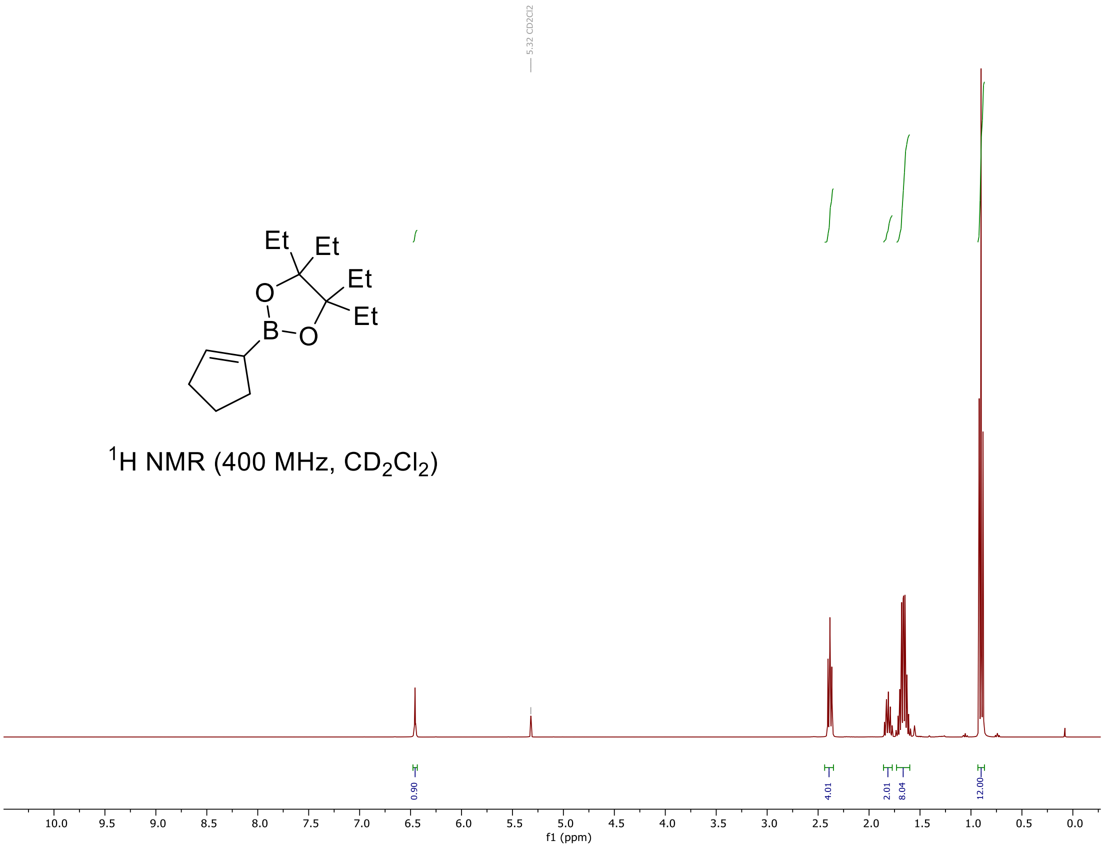

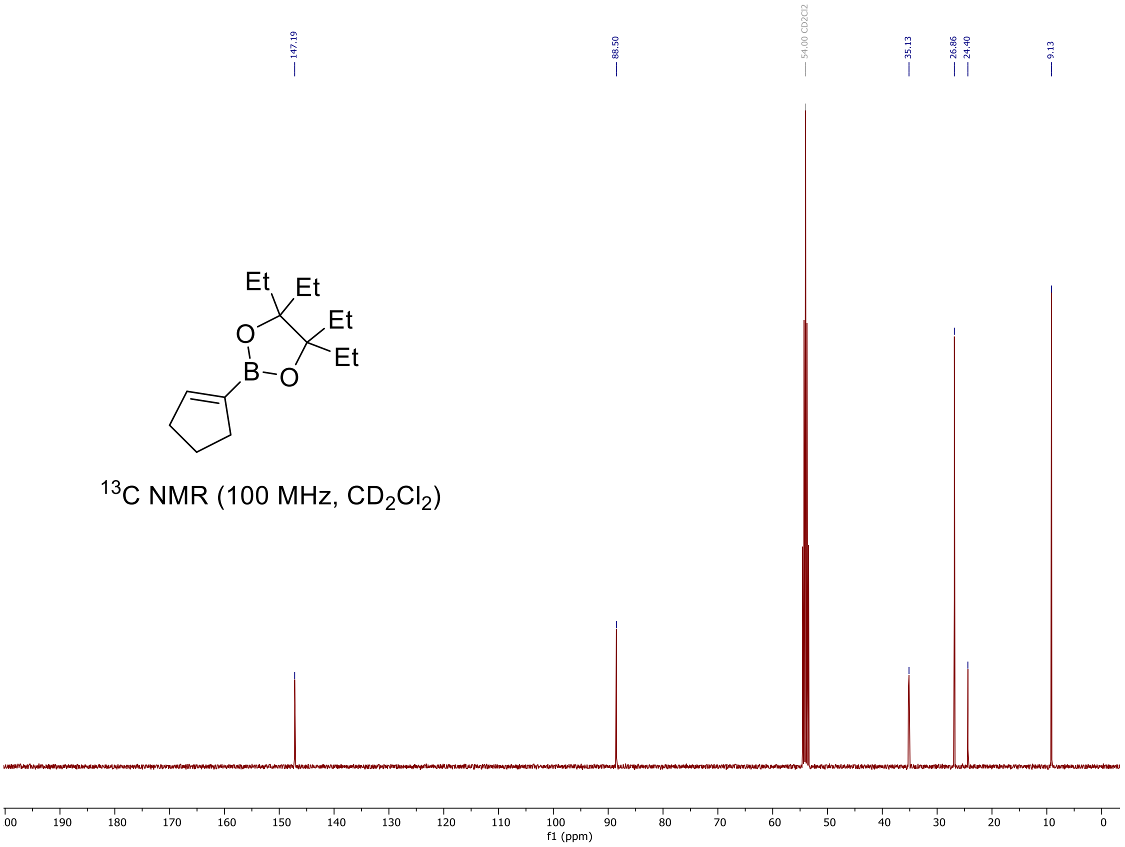

(8*R*,9*S*,13*S*,14*S*)-17-iodo-3-methoxy-13-methyl-7,8,9,11,12,13,14,15-octahydro-6H-cyclopenta[a]phenanthrene

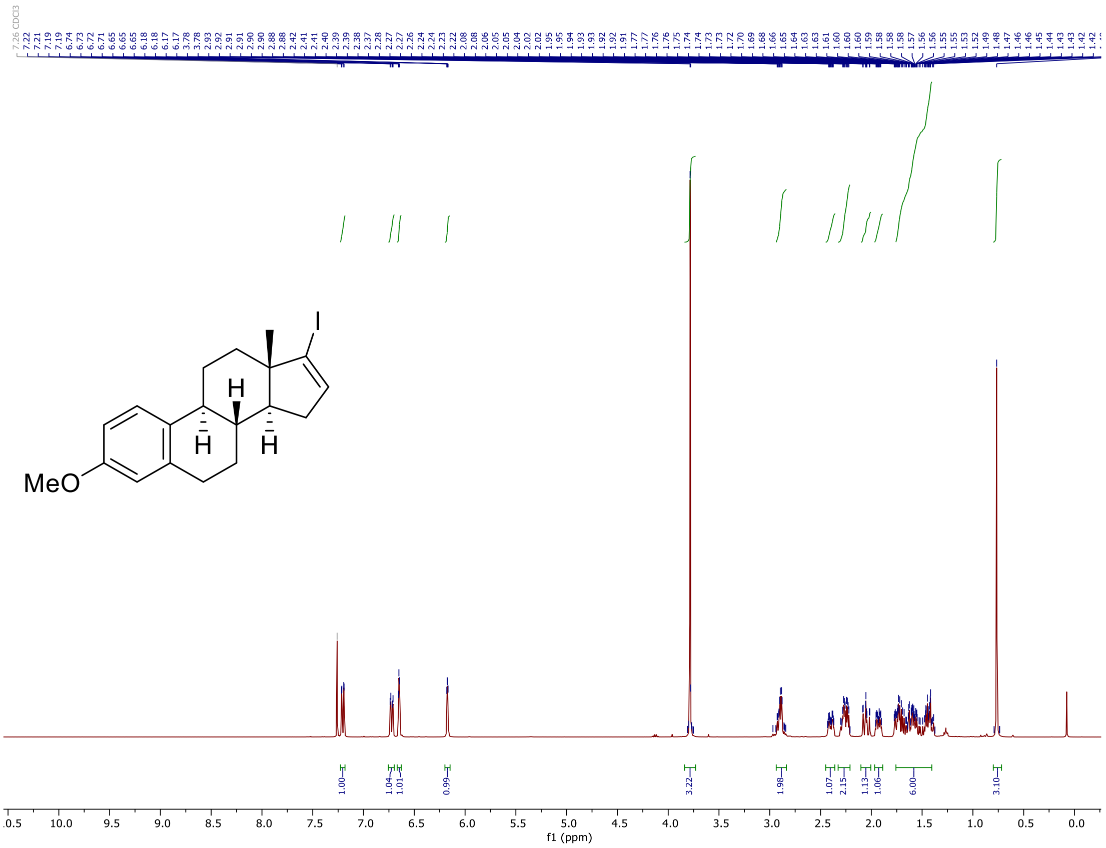

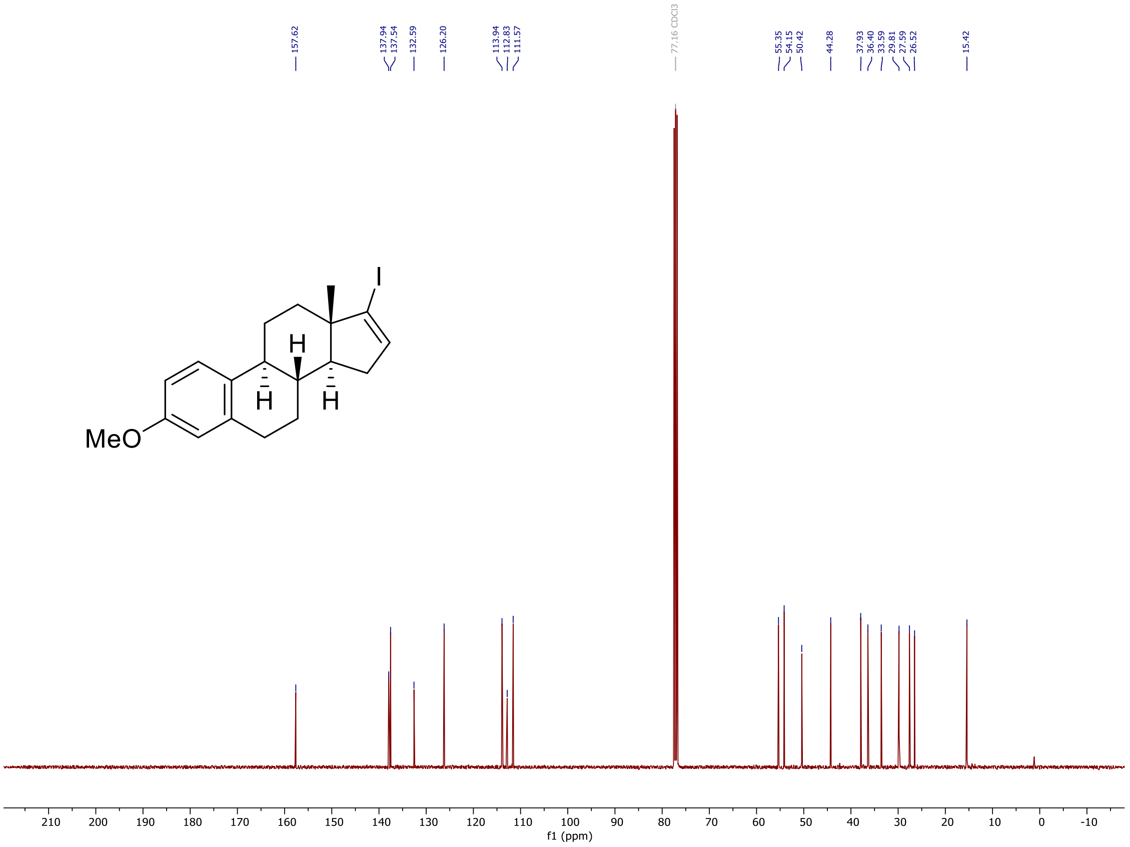

4,4,5,5-tetraethyl-2-((8S,9S,13S,14S)-3-methoxy-13-methyl-7,8,9,11,12,13,14,15-octahydro-6H-cyclopenta[a]phenanthren-17-yl)-1,3,2-dioxaborolane (Si-27)

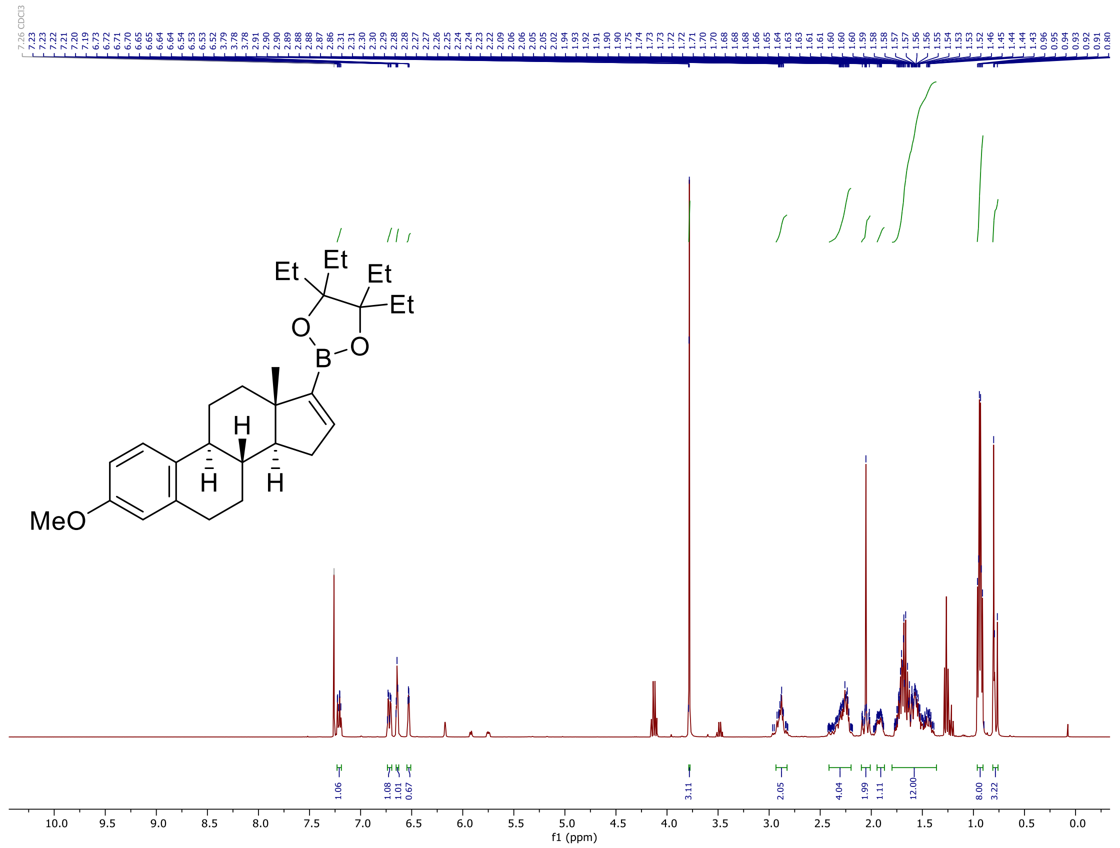

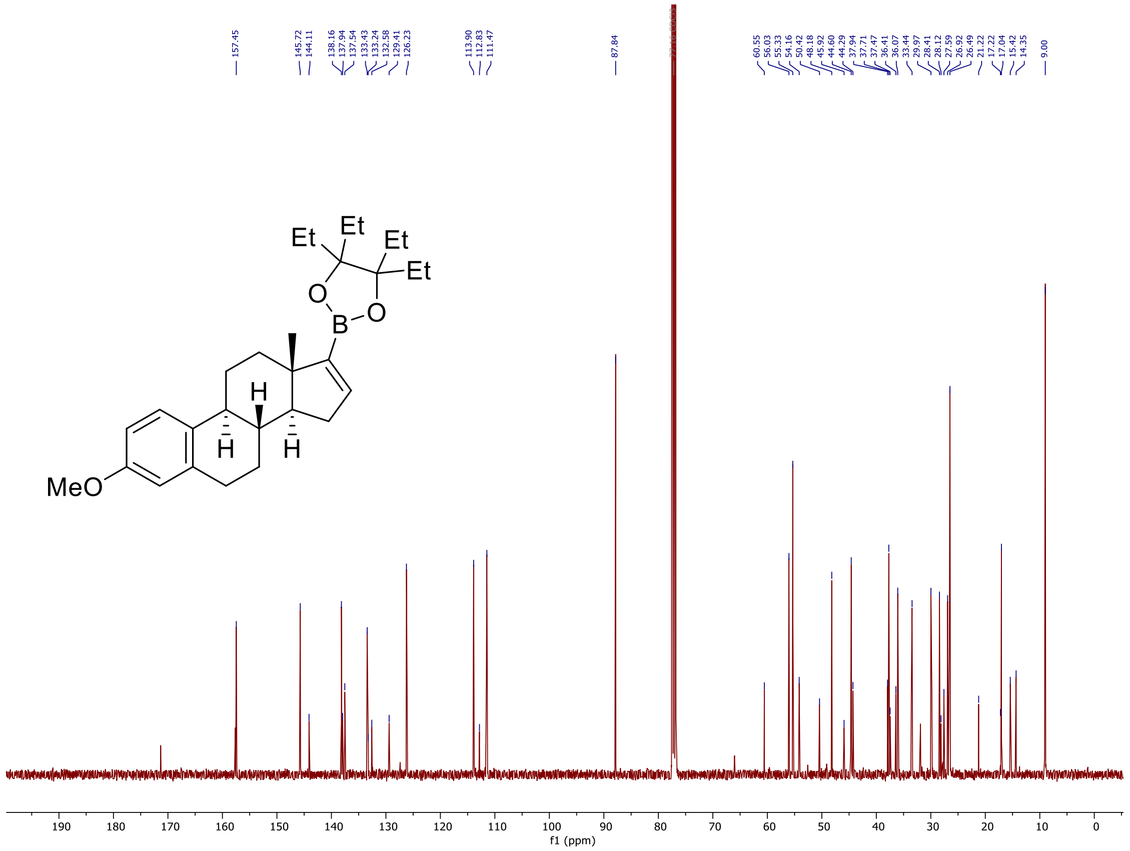

2-iodobicyclo[2.2.1]hept-2-ene

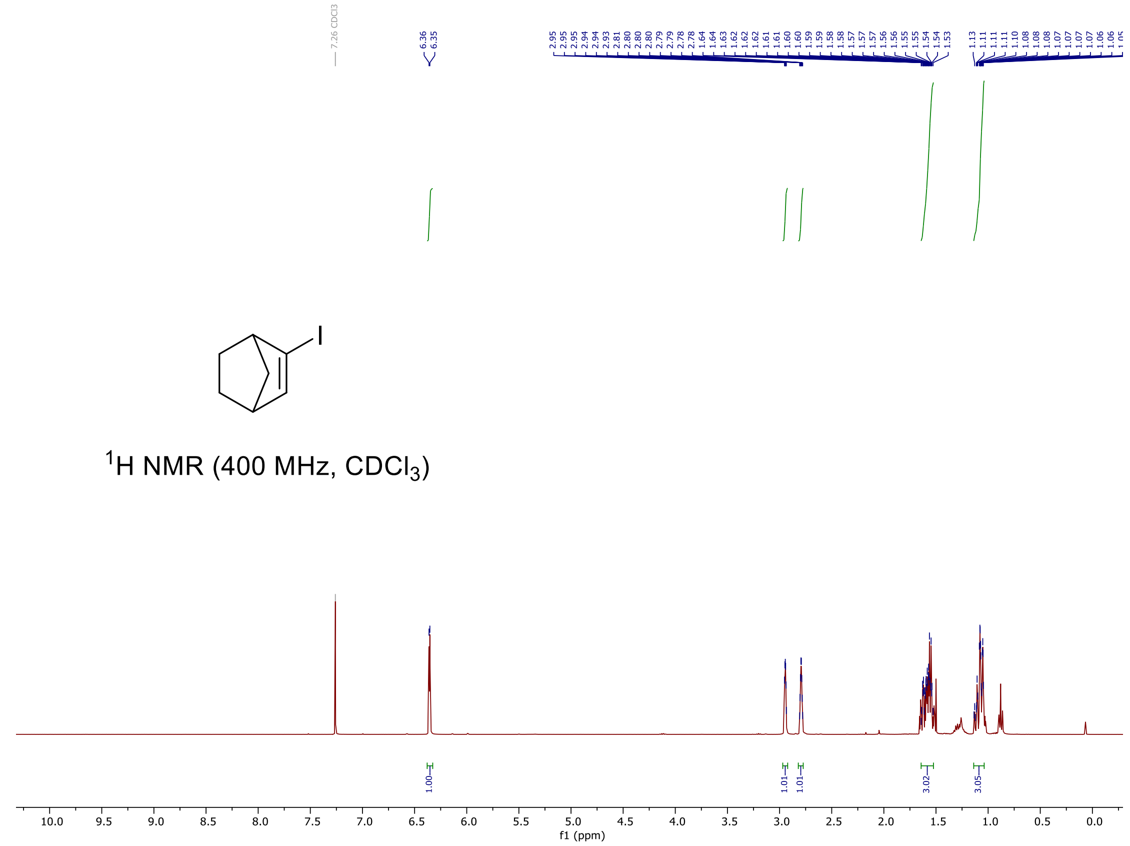

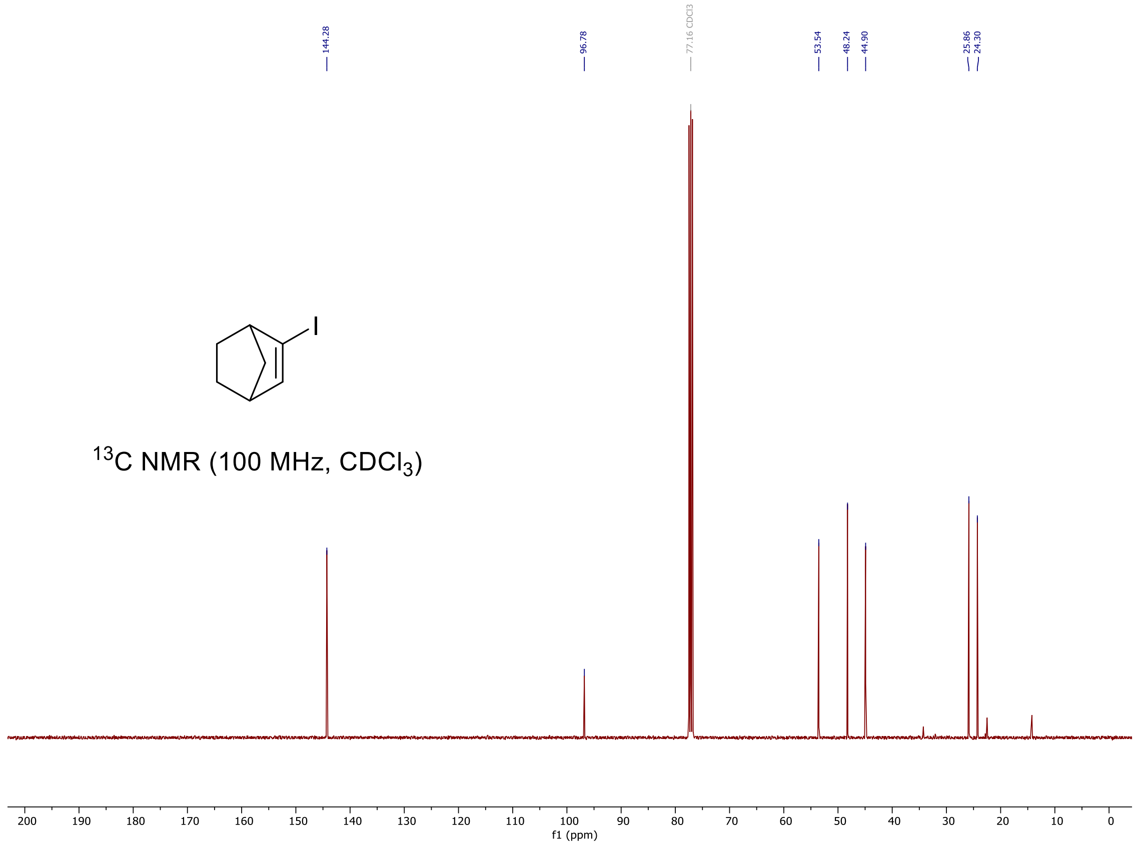

2-(Bicyclo[2.2.1]hept-2-en-2-yl)-4,4,5,5-tetraethyl-1,3,2-dioxaborolane (13)

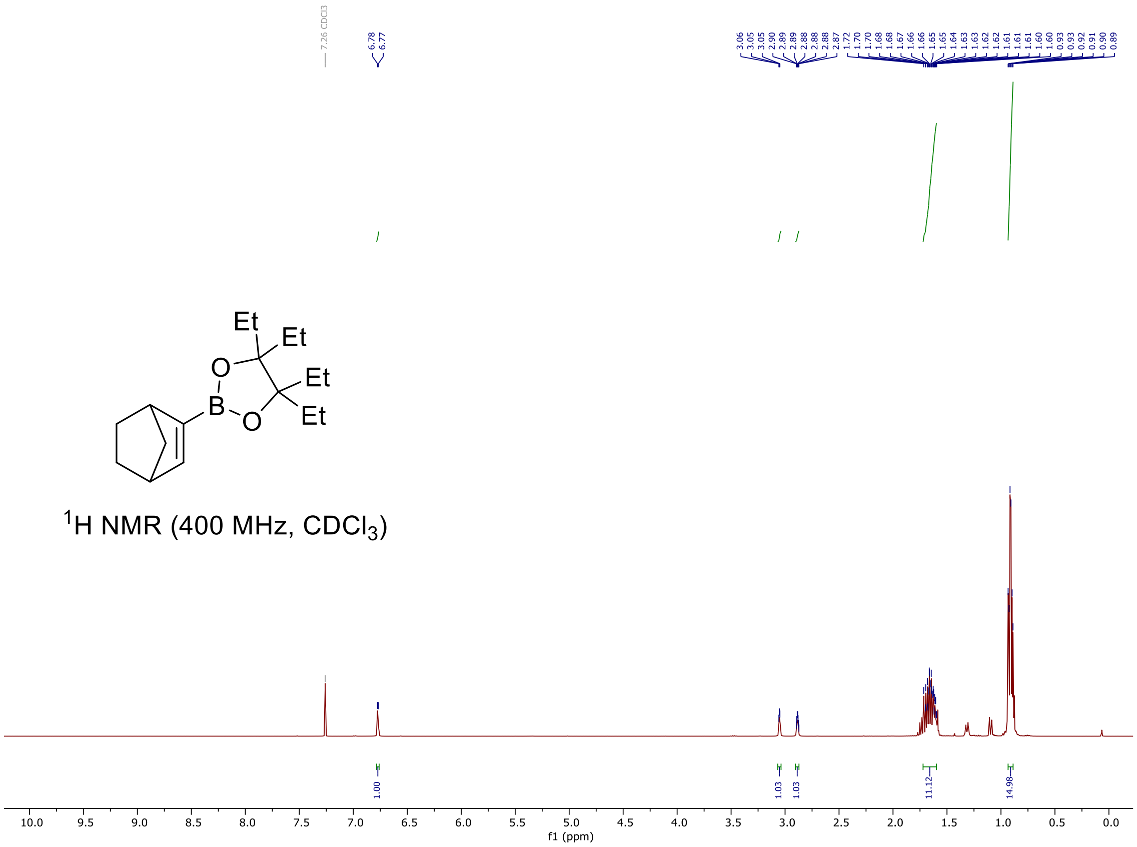

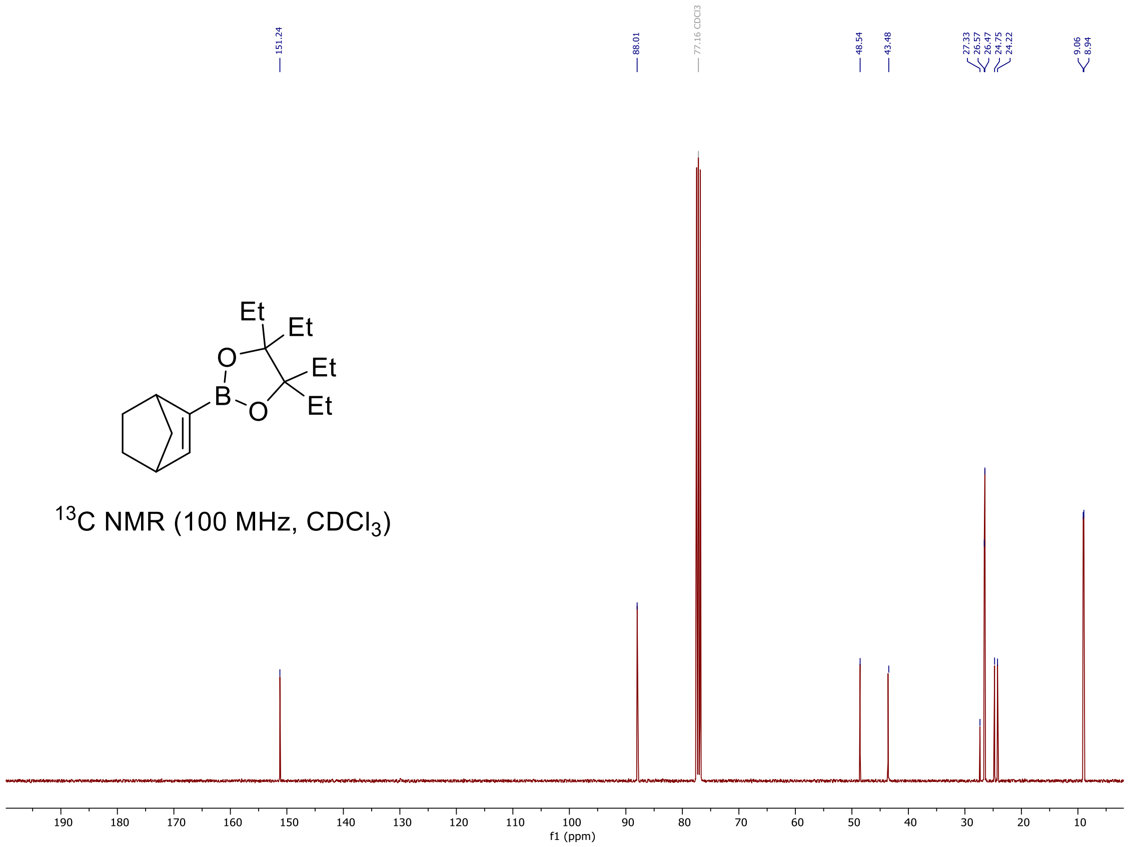

***tert*-Butyl (2*R*,3*S*)-2-butyl-3-(perfluorobutyl)-2-(4,4,5,5-tetraethyl-1,3,2-dioxaborolan-2-yl)azetidine-1-carboxylate (<u>3a</u>)**

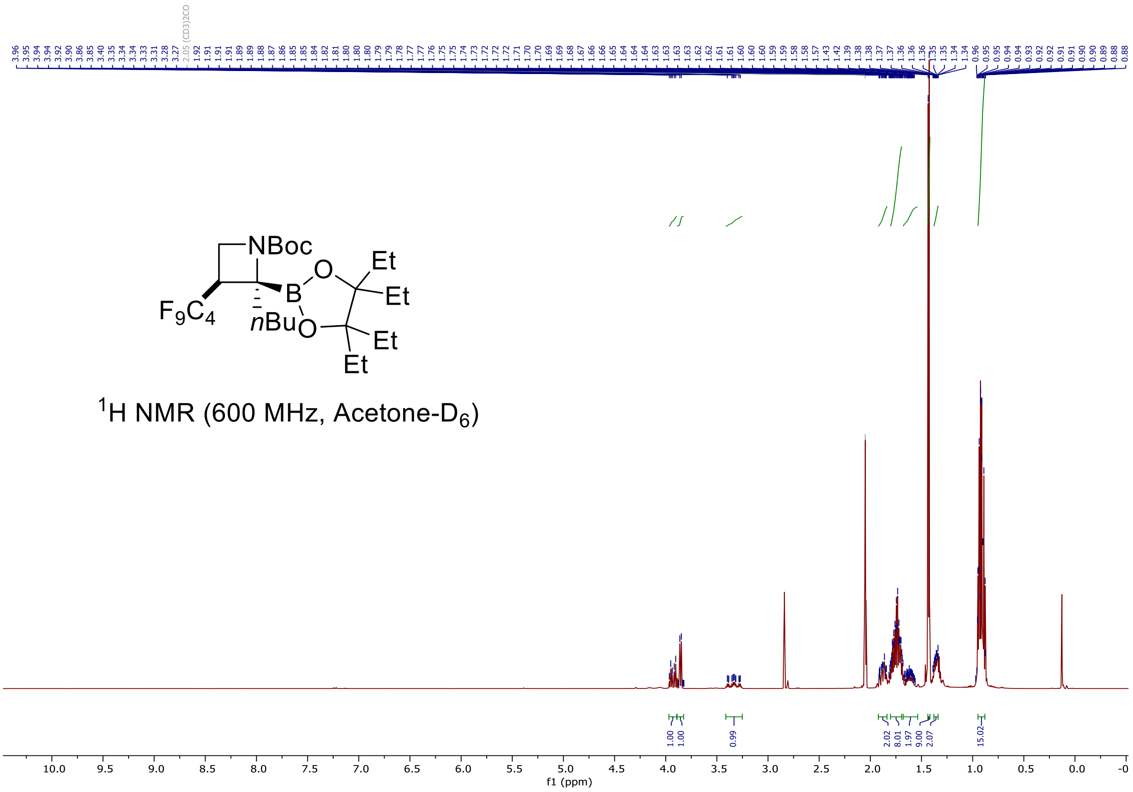

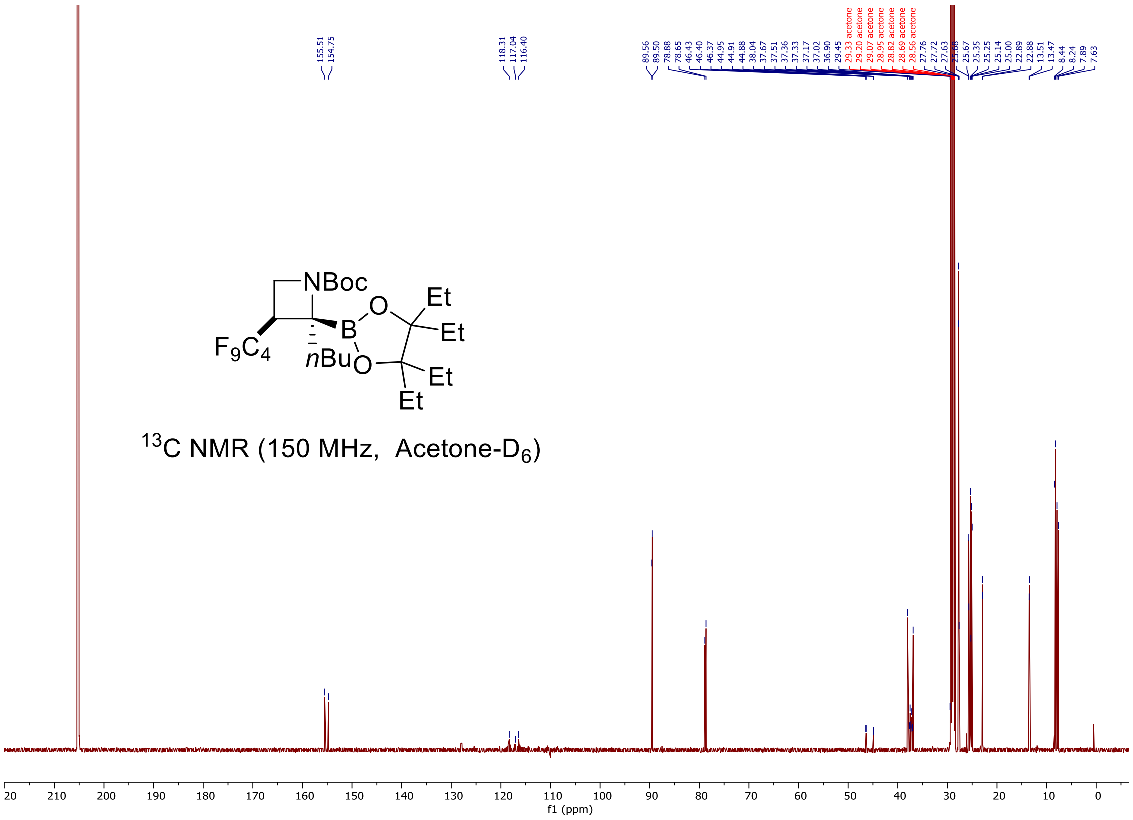

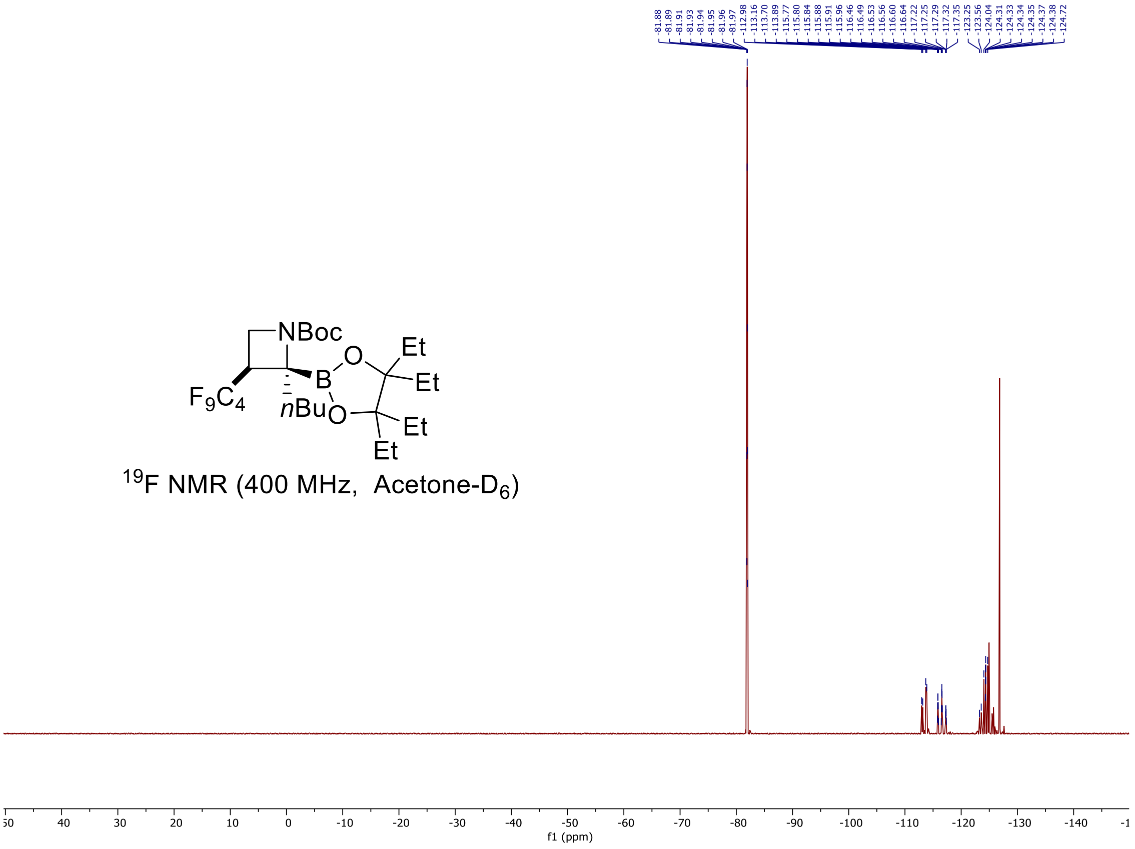

NBoc
Et
O
B
Et
F_9C_4
nBu
O
Et
Et
^{19}F NMR (400 MHz, Acetone-D$_6$)
f1 (ppm)

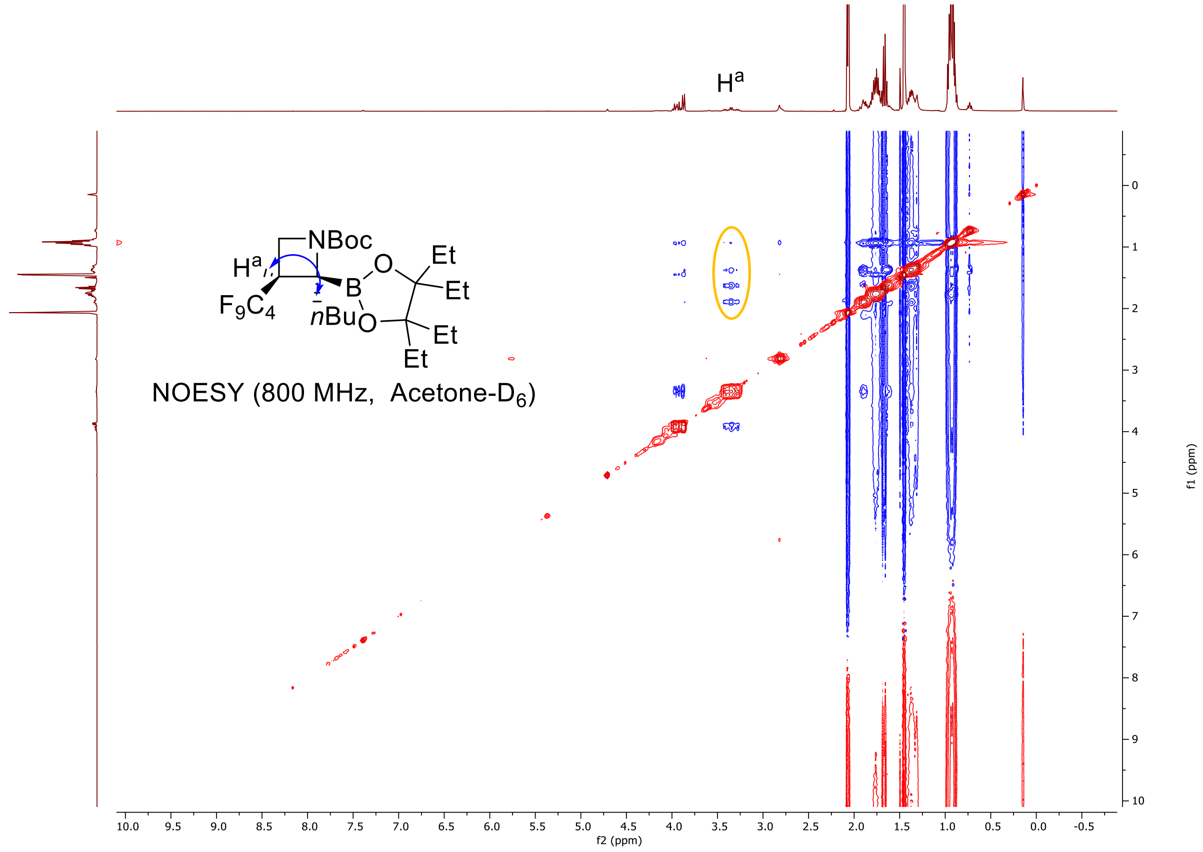

H^a
NBoc
Et
O
B
Et
F_9C_4
nBu
O
Et
Et
NOESY (800 MHz, Acetone-D$_6$)
f2 (ppm)
f1 (ppm)

tert-Butyl (2_R_,3_S_)-2-(2-cyclohexylethyl)-3-(perfluorobutyl)-2-(4,4,5,5-tetraethyl-1,3,2-dioxaborolan-2-yl)azetidine-1-carboxylate (3b)

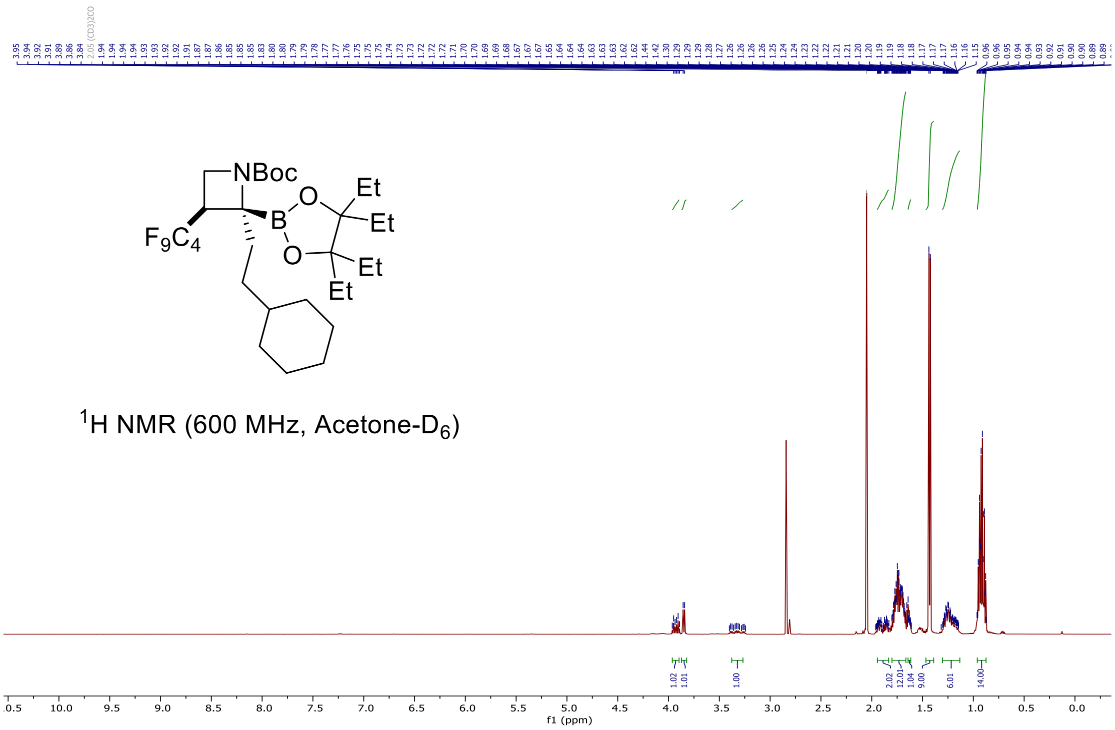

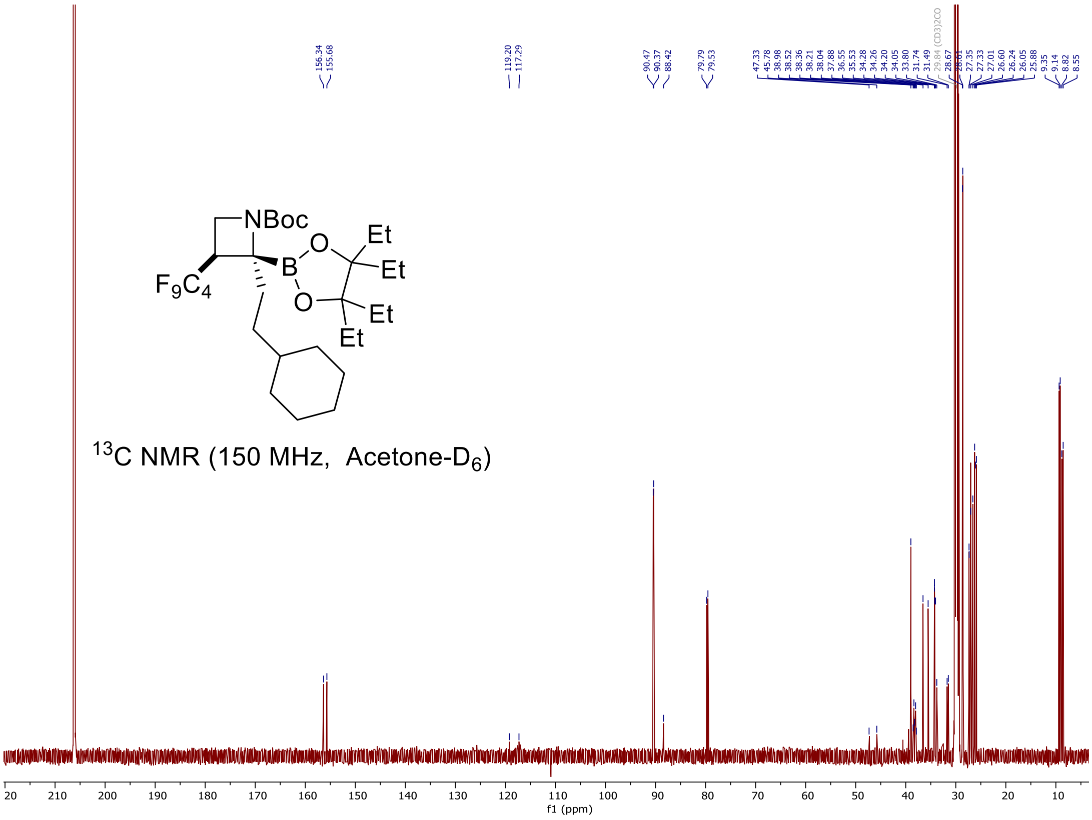

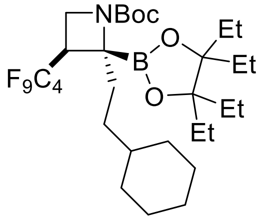

^{19}F NMR (400 MHz, Acetone-D$_6$)

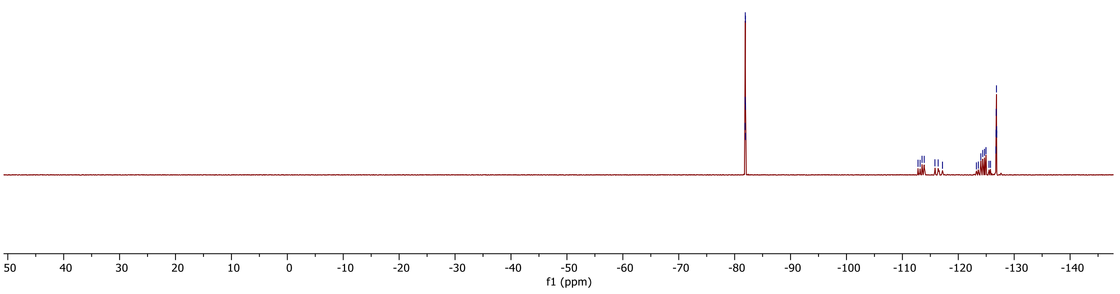

***tert*-Butyl (2*R*,3*S*)-3-(perfluorobutyl)-2-phenethyl-2-(4,4,5,5-tetraethyl-1,3,2-dioxaborolan-2-yl)azetidine-1-carboxylate (3c)**

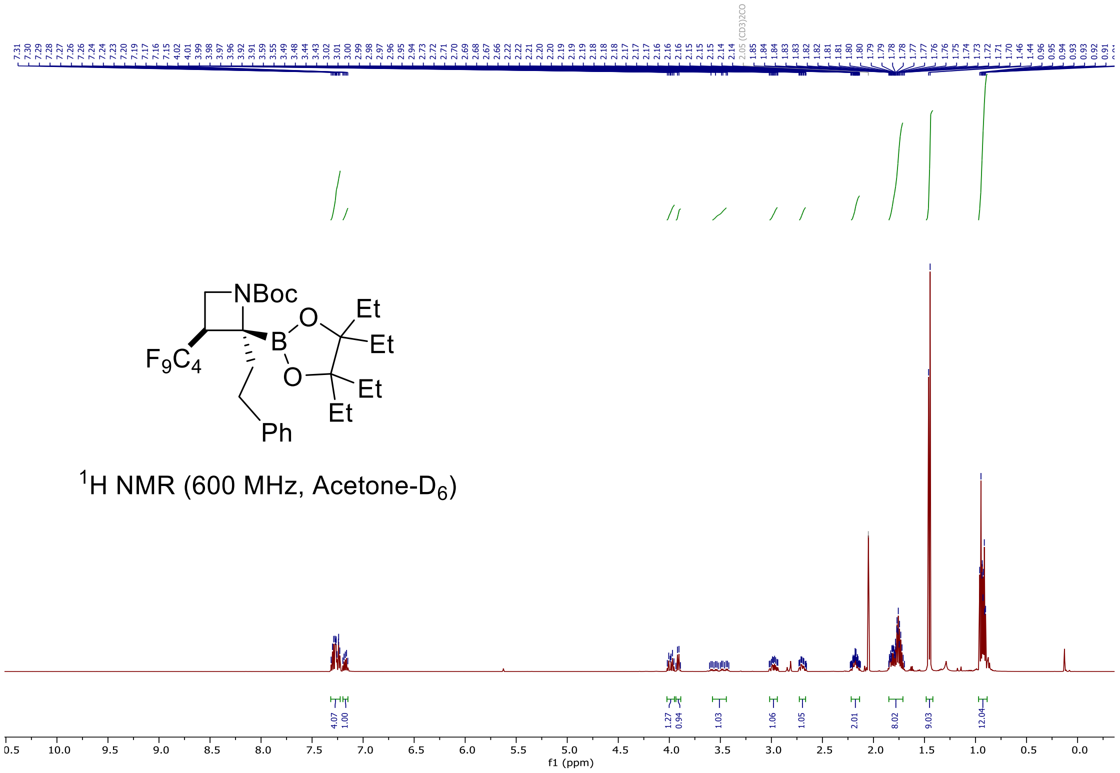

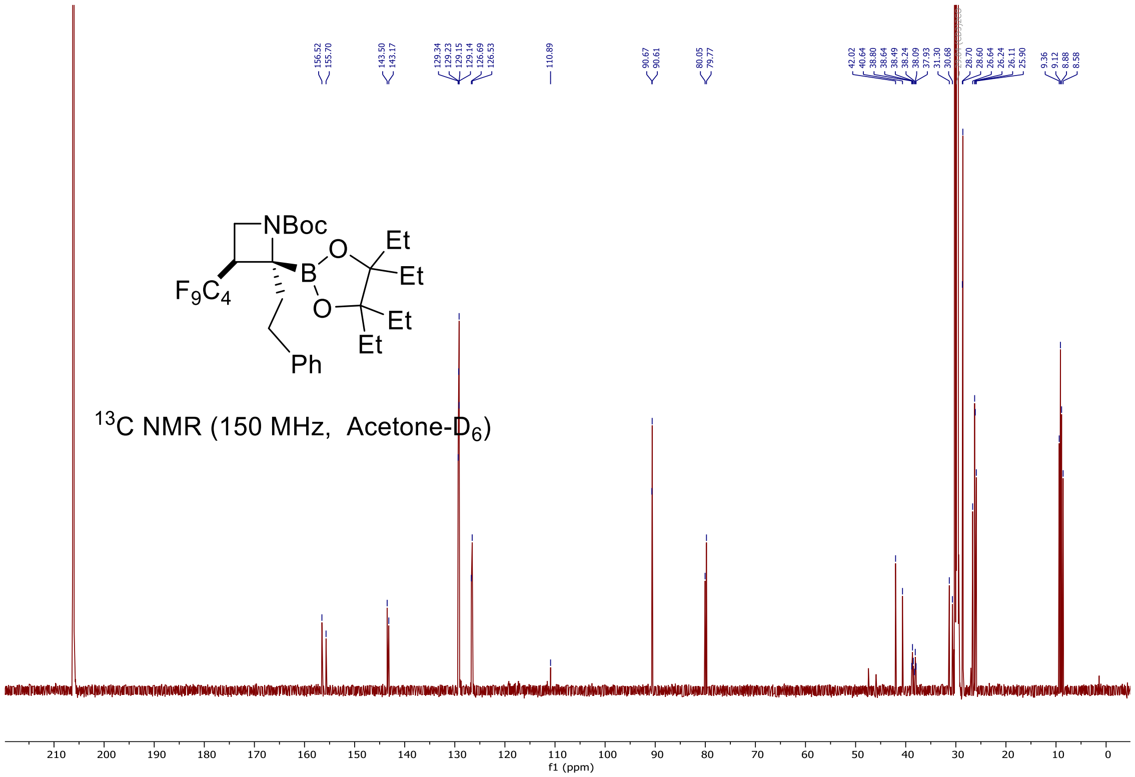

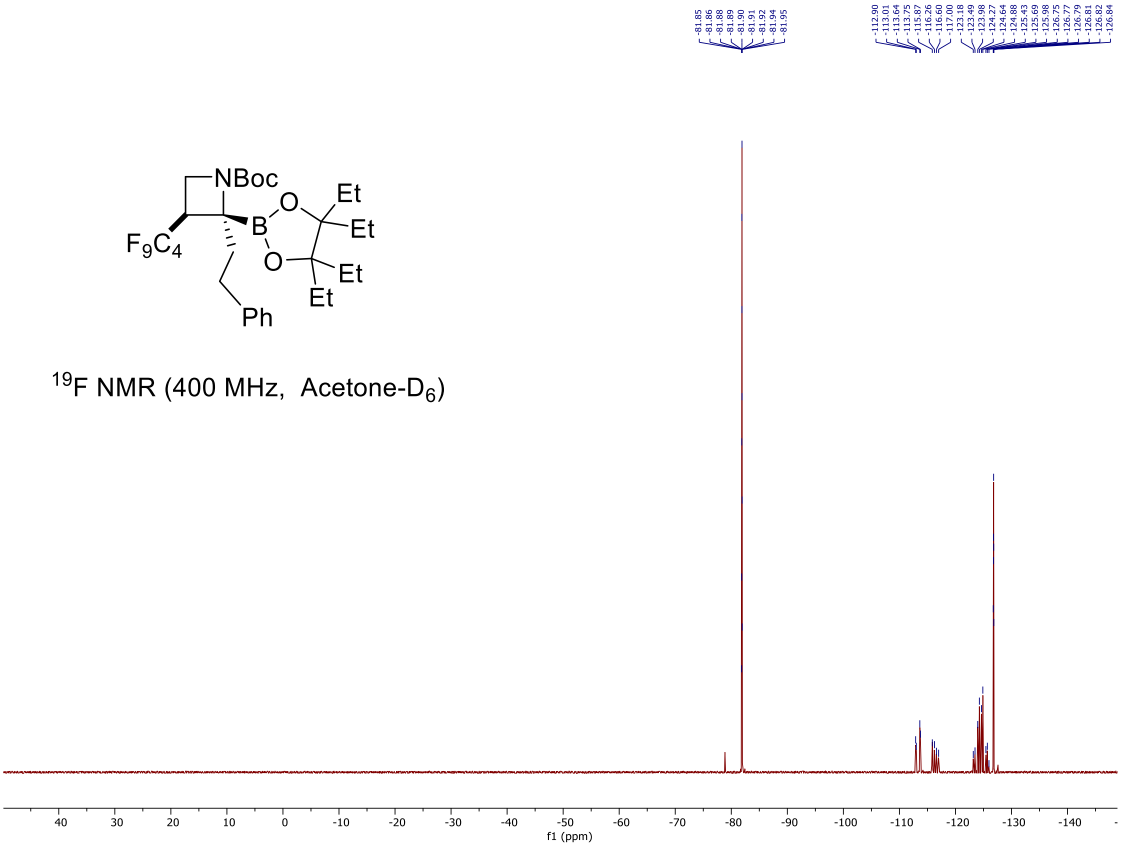

^{19}F NMR (400 MHz, Acetone-D$_6$)

***tert*-Butyl (2*R*,3*S*)-3-(perfluorohexyl)-2-phenethyl-2-(4,4,5,5-tetraethyl-1,3,2-dioxaborolan-2-yl)azetidine-1-carboxylate (3d)**

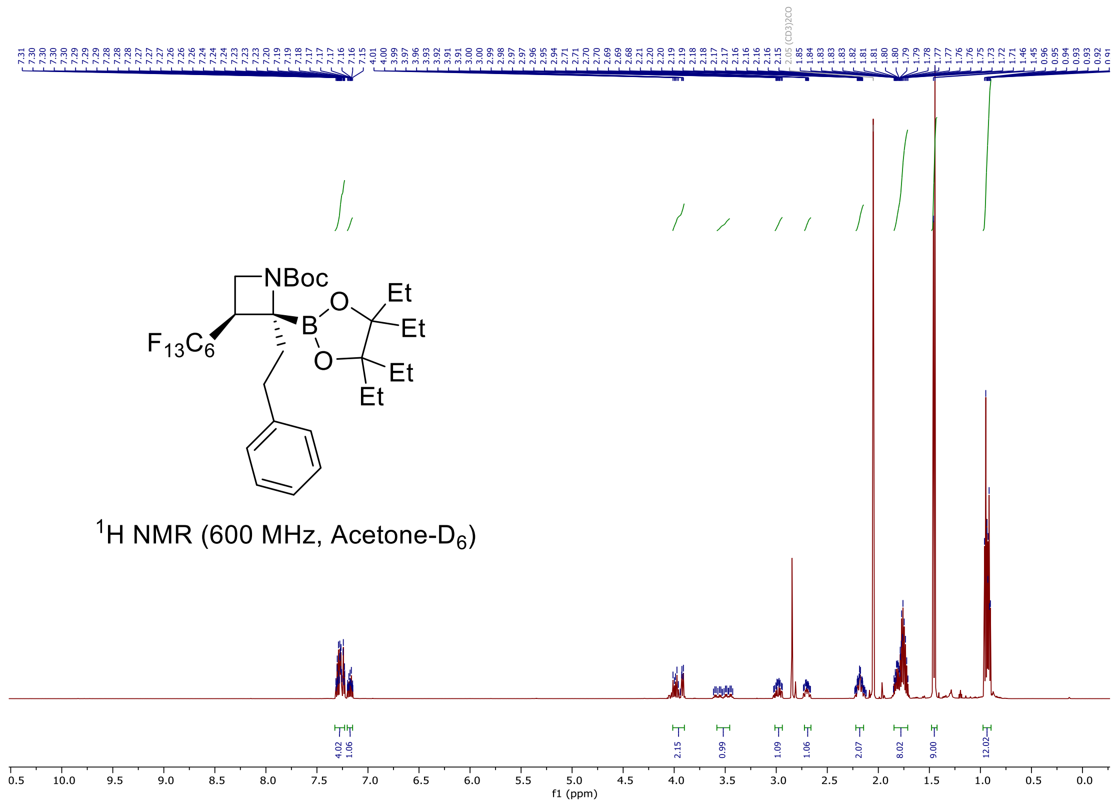

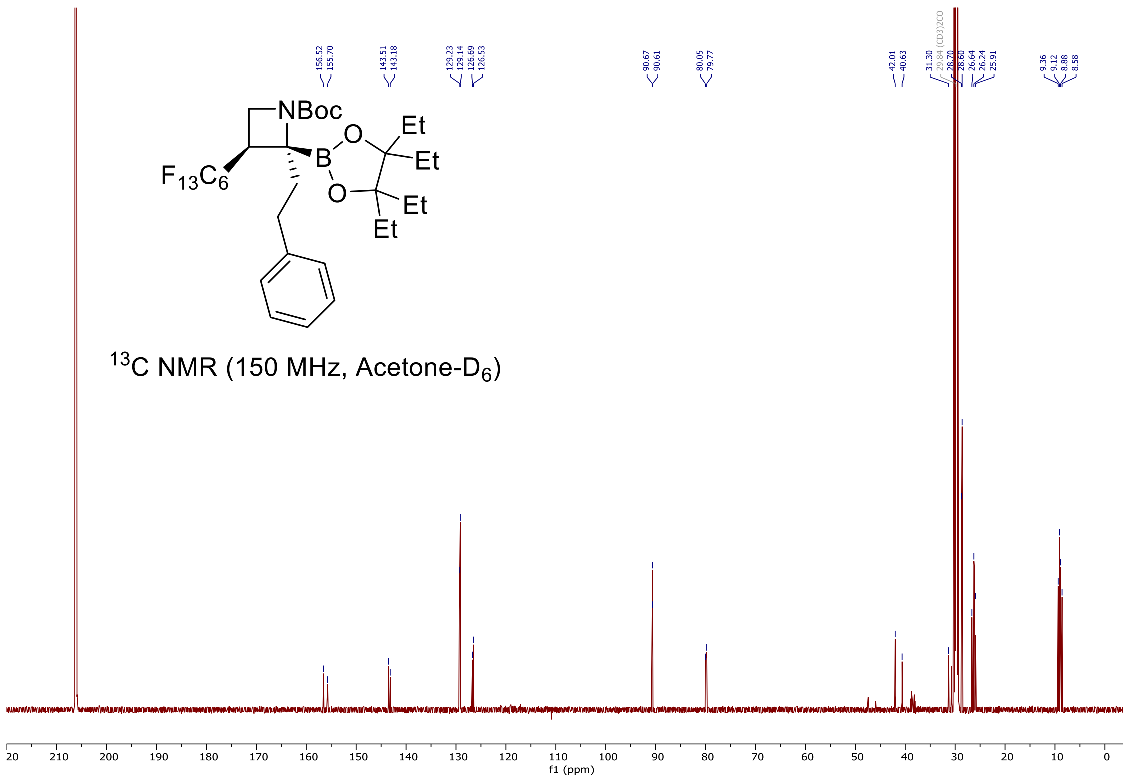

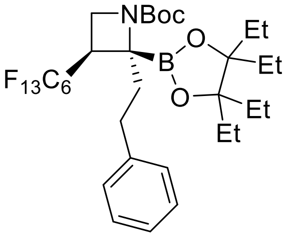

^{19}F NMR (400 MHz, Acetone-D_6)

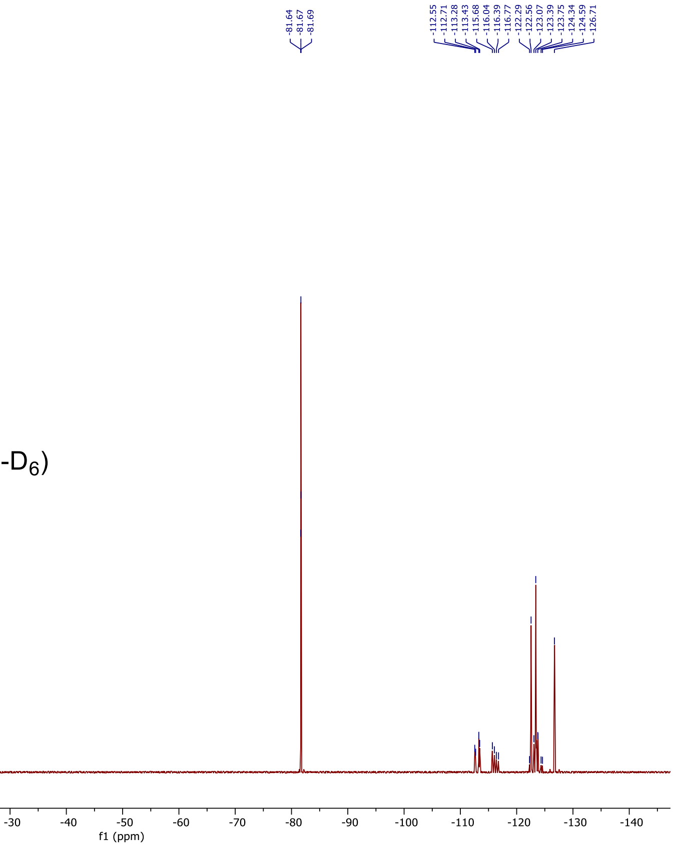

***tert*-Butyl (2*R*,3*S*)-2-methyl-3-(perfluorobutyl)-2-(4,4,5,5-tetraethyl-1,3,2-dioxaborolan-2-yl)azetidine-1-carboxylate (<u>3e</u>)**

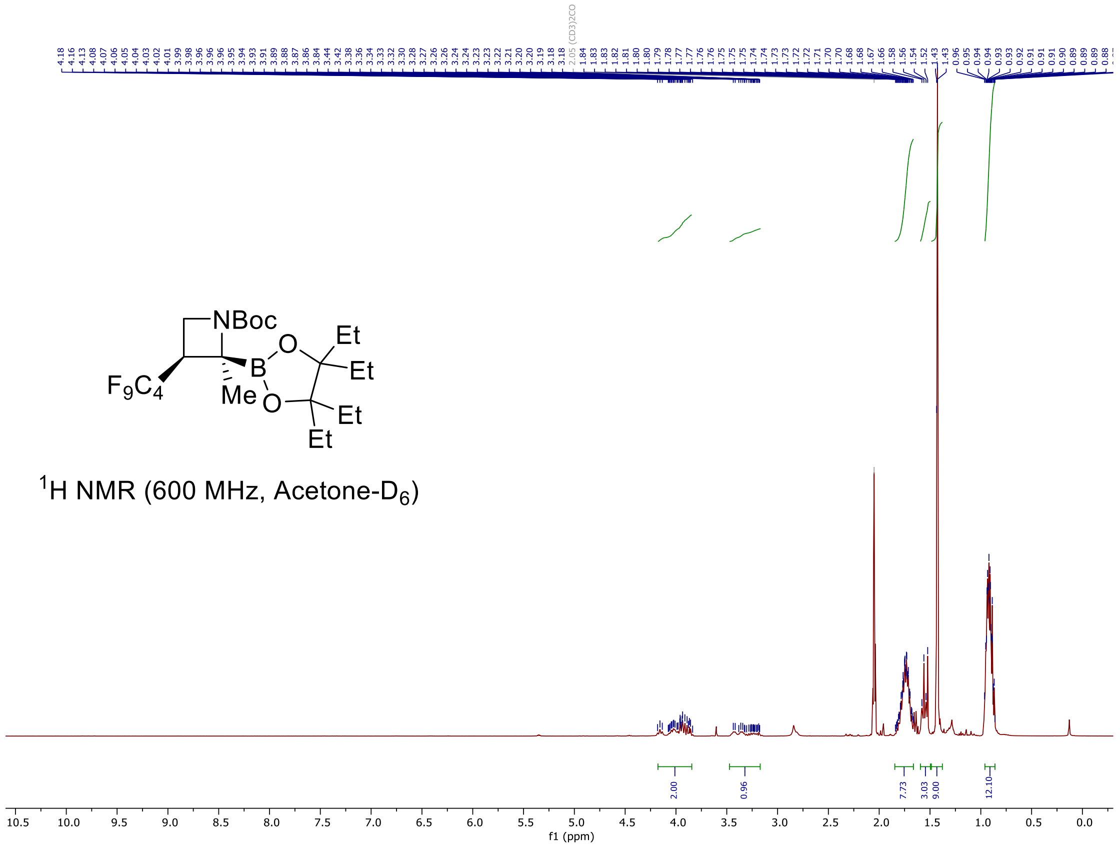

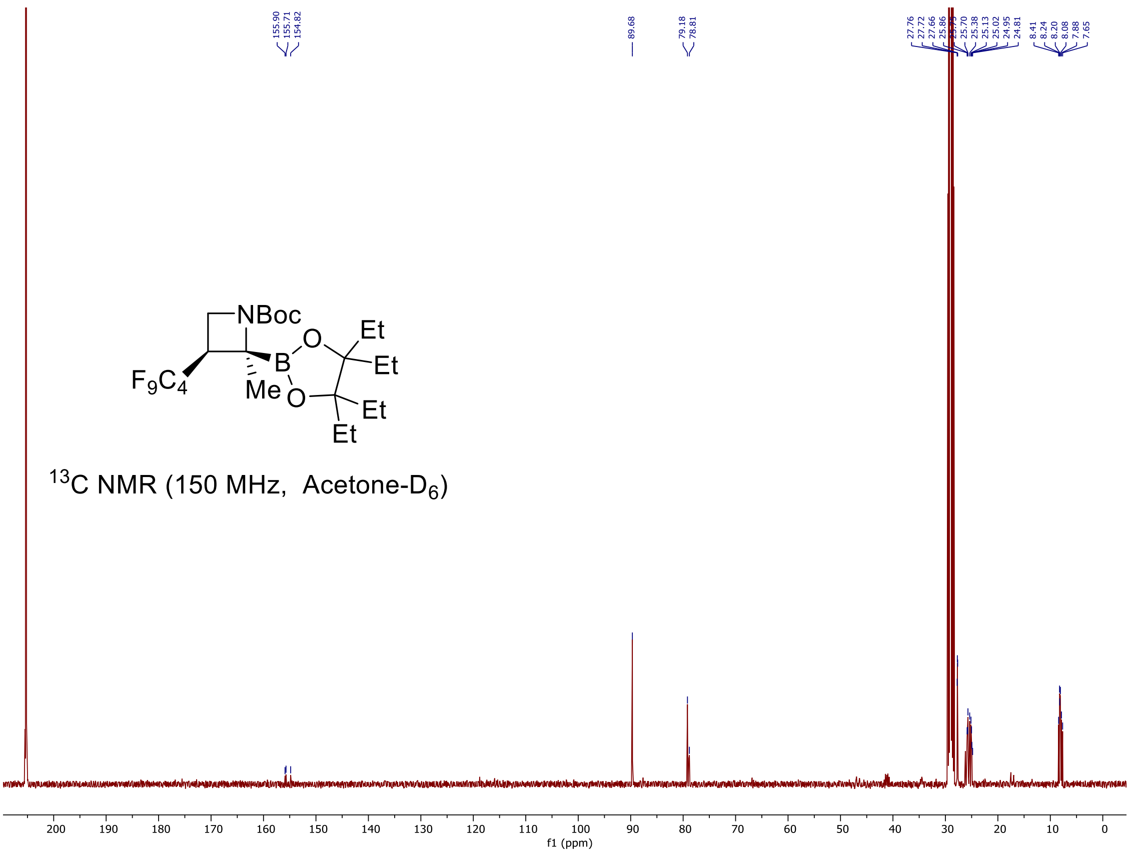

***tert*-Butyl (2*R*,3*S*)-3-(2-ethoxy-1,1-difluoro-2-oxoethyl)-2-phenethyl-2-(4,4,5,5-tetraethyl-1,3,2-dioxaborolan-2-yl)azetidine-1-carboxylate (3f)**

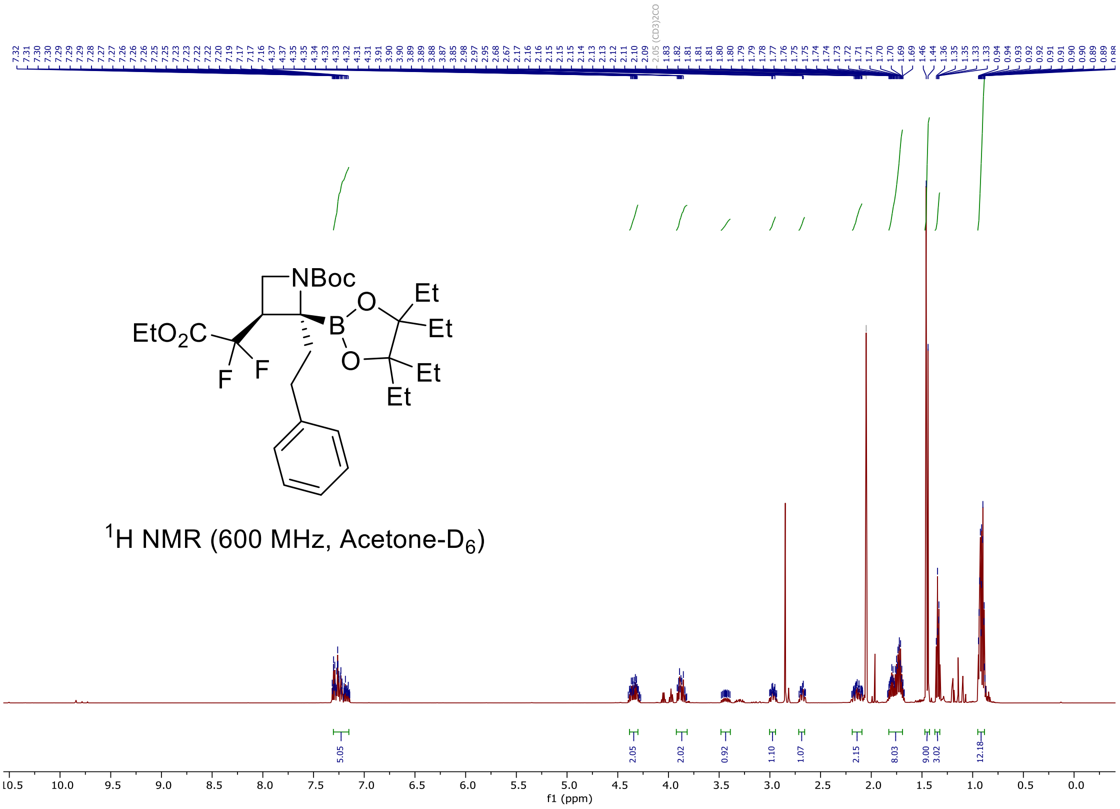

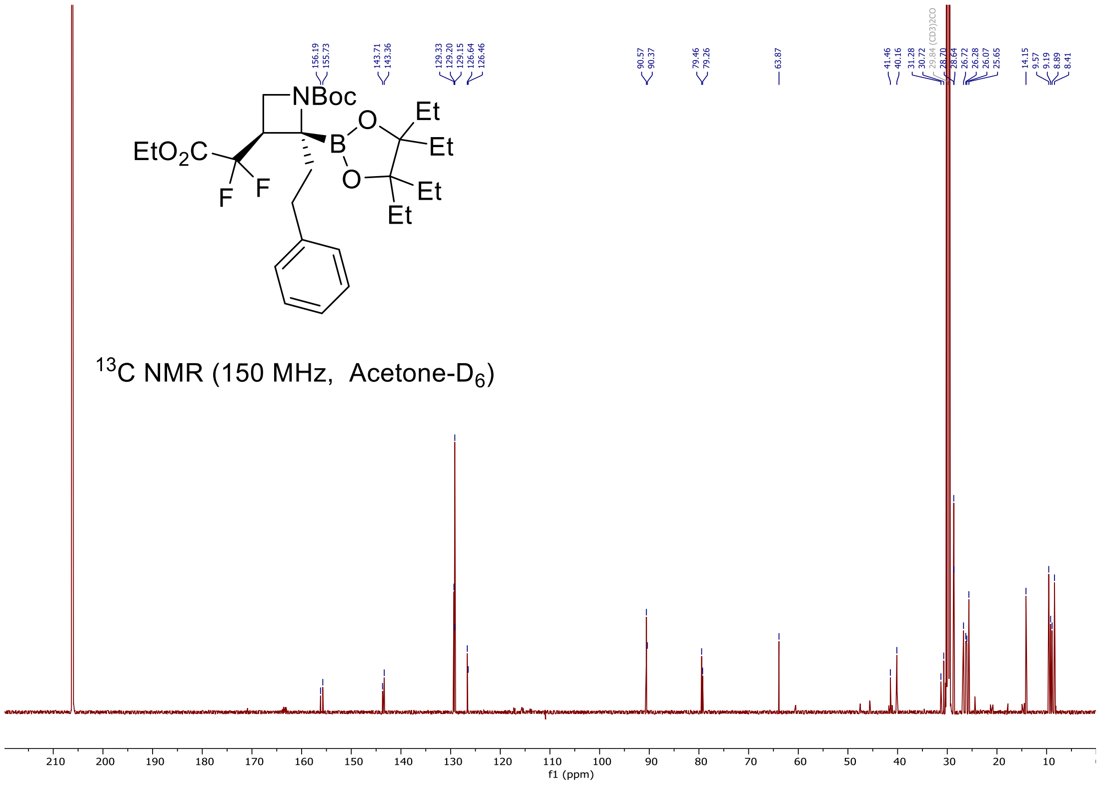

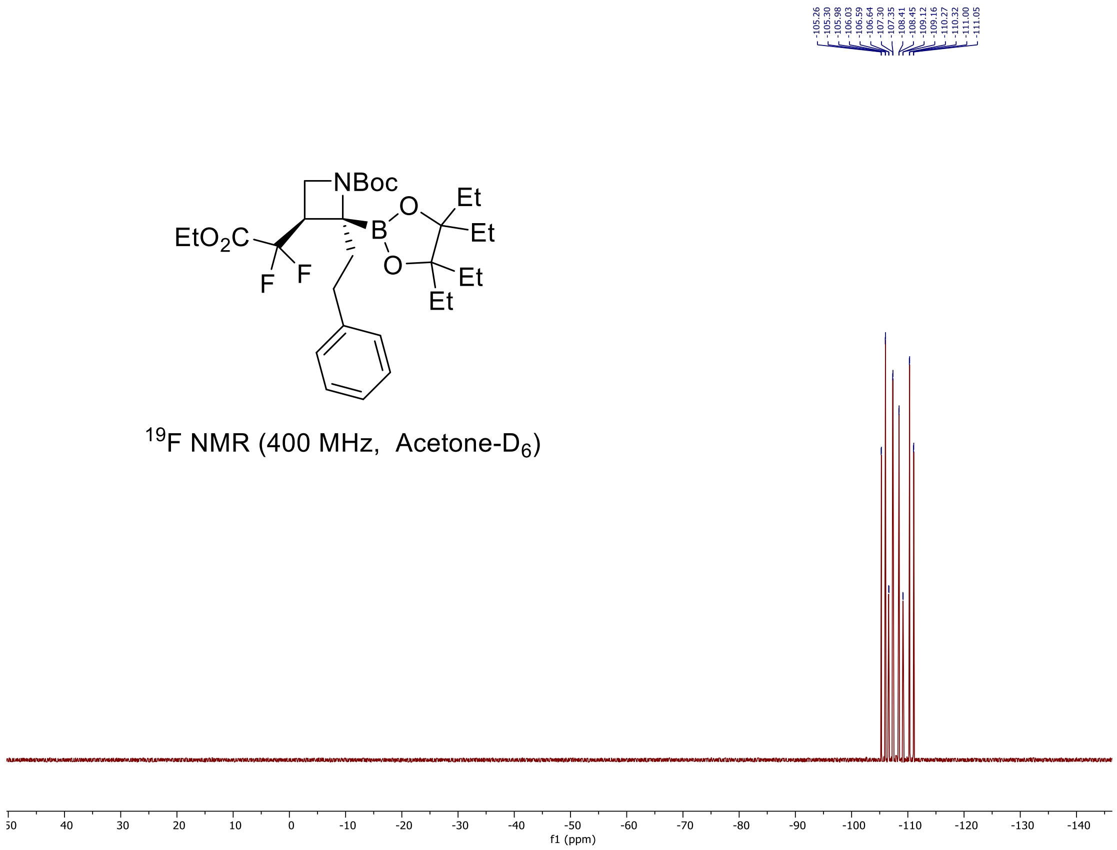

NBoc
EtO2C
F
F
B
O
O
Et
Et
Et
Et
19F NMR (400 MHz, Acetone-D6)
-105.26
-105.30
-105.98
-106.03
-106.59
-106.64
-107.30
-107.35
-108.41
-108.45
-109.12
-109.16
-110.27
-110.32
-111.00
-111.05
f1 (ppm)

tert-Butyl (2_S_,3_S_)-3-(2-ethoxy-1,1-difluoro-2-oxoethyl)-2-(4,4,5,5-tetraethyl-1,3,2-dioxaborolan-2-yl)-2-((trimethylsilyl)methyl)azetidine-1-carboxylate (3g)

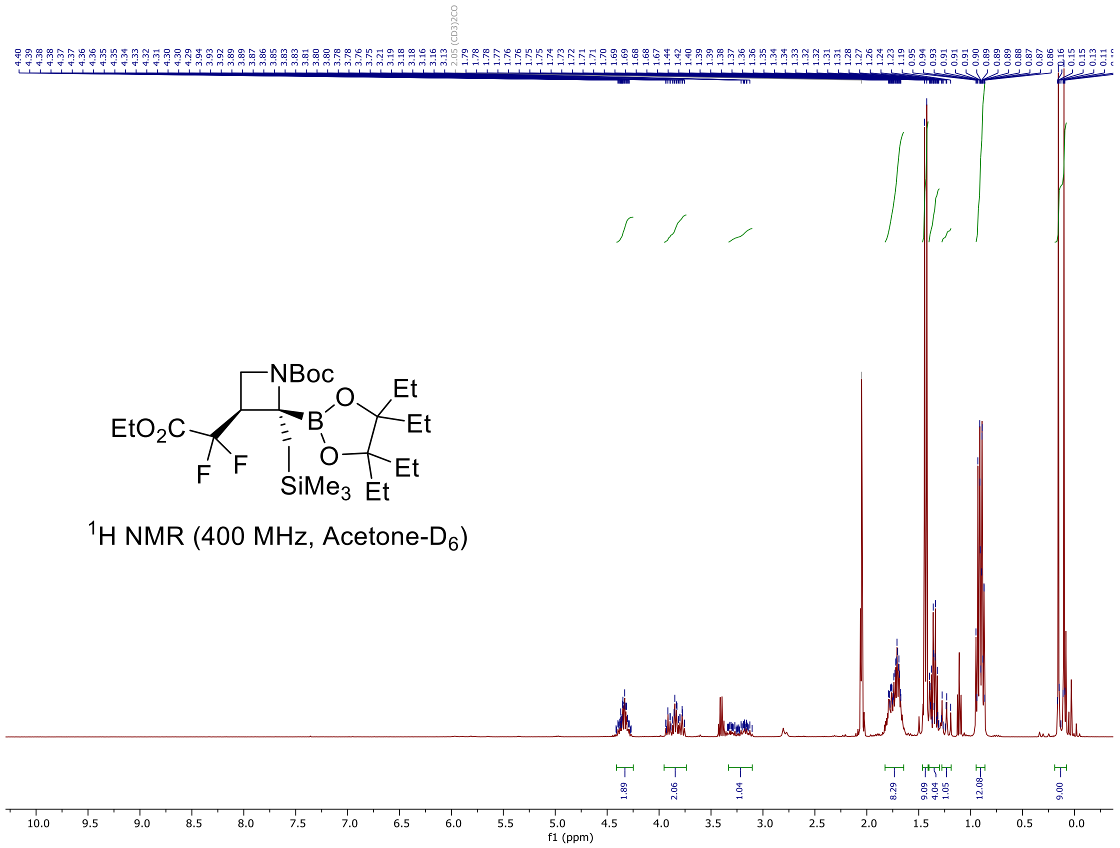

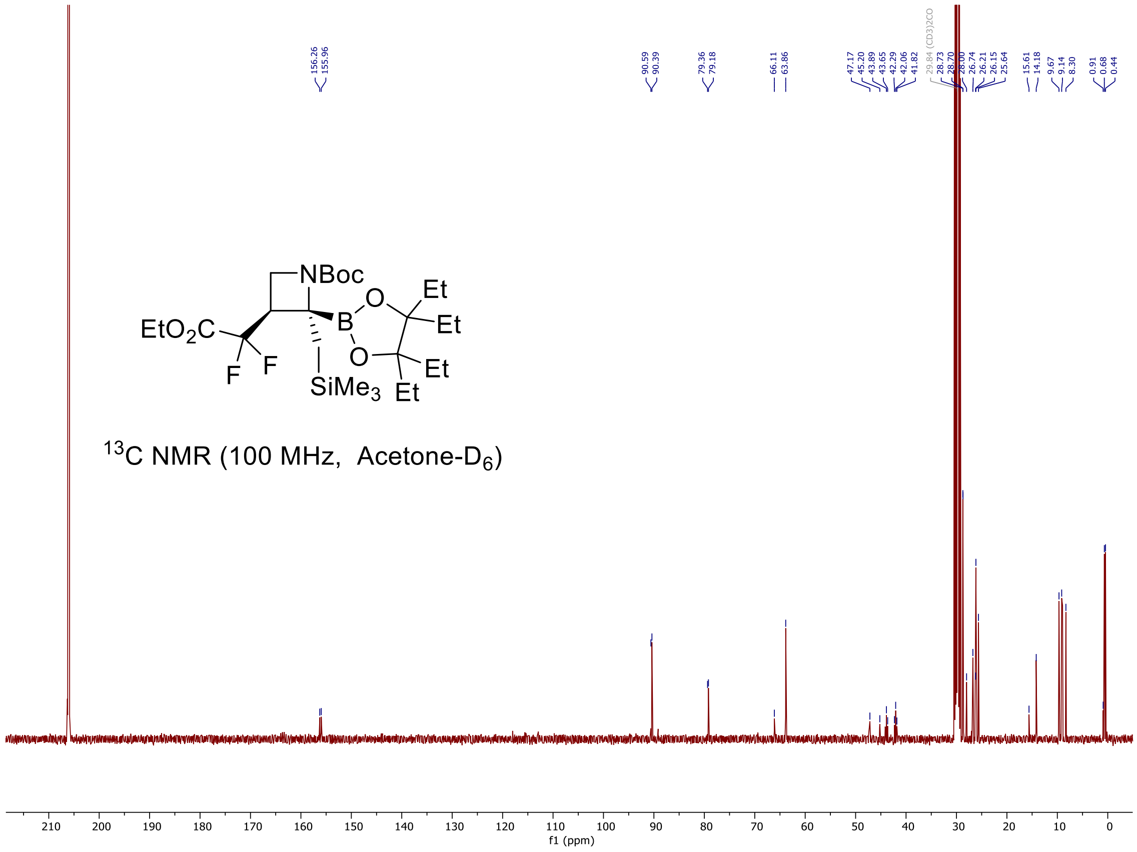

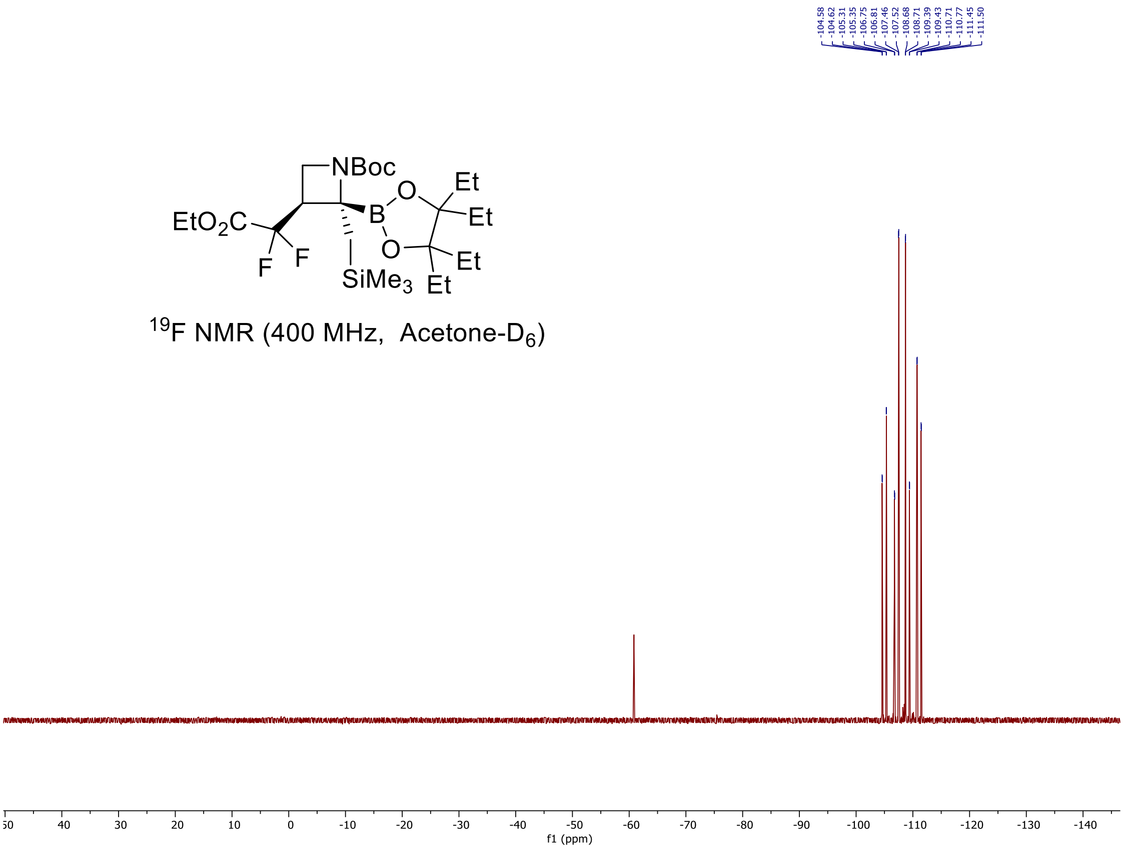

^{19}F NMR (400 MHz, Acetone-D_6)

***tert*-Butyl (2*R*,3*S*)-2-phenethyl-2-(4,4,5,5-tetraethyl-1,3,2-dioxaborolan-2-yl)-3-(2,2,2-trifluoroethyl)azetidine-1-carboxylate (3h)**

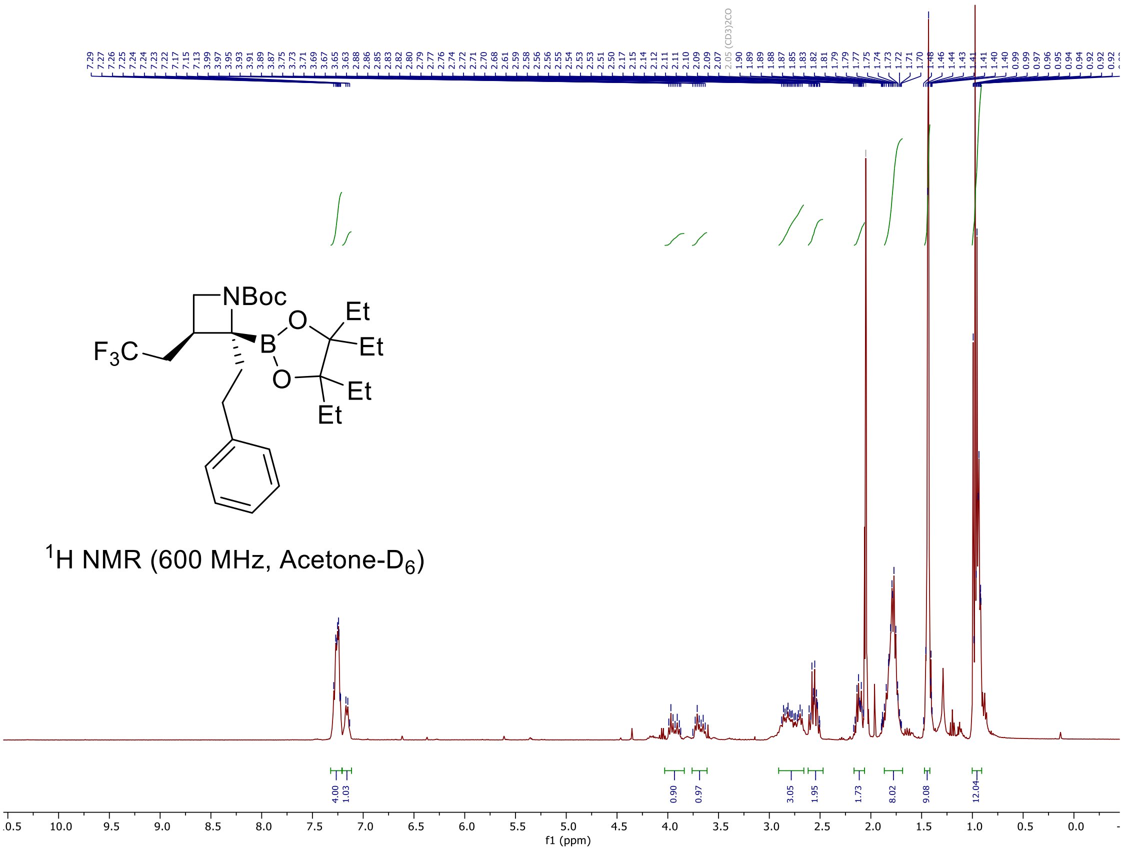

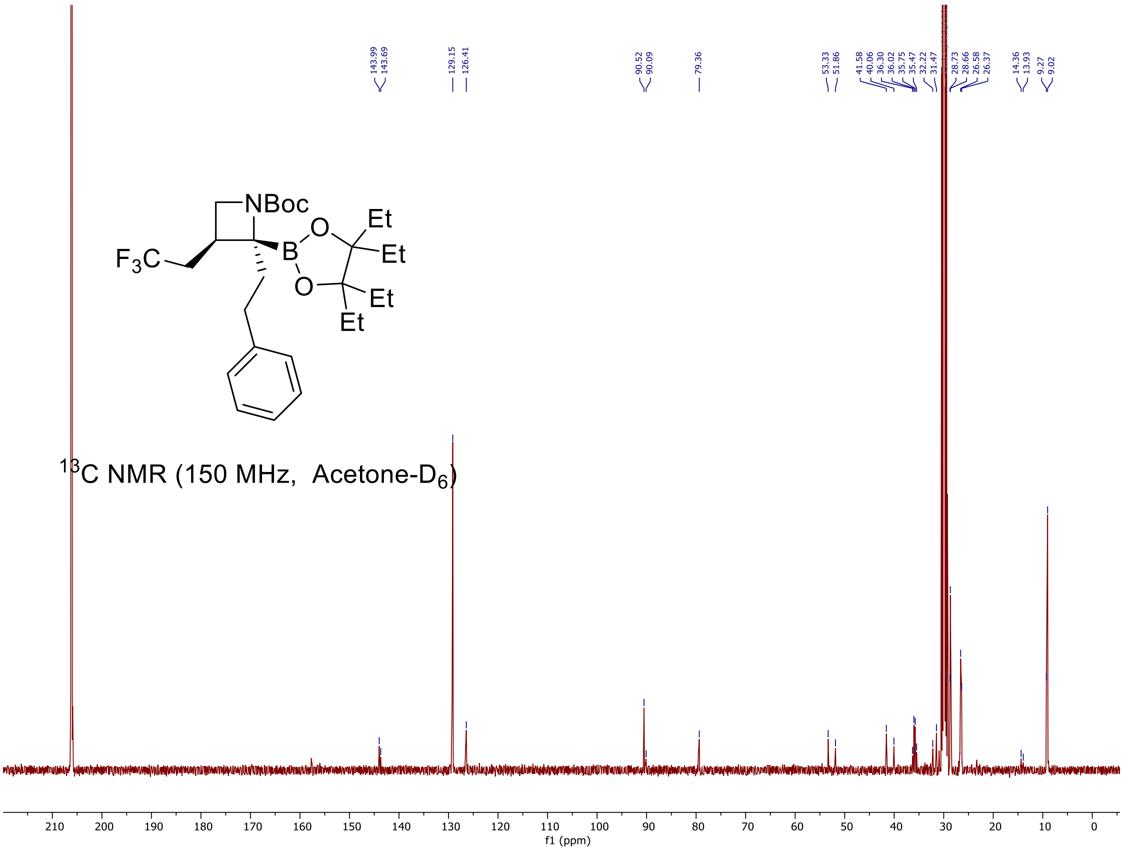

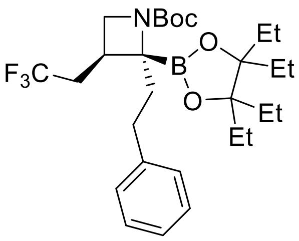

^{19}F NMR (400 MHz, Acetone-D_6)

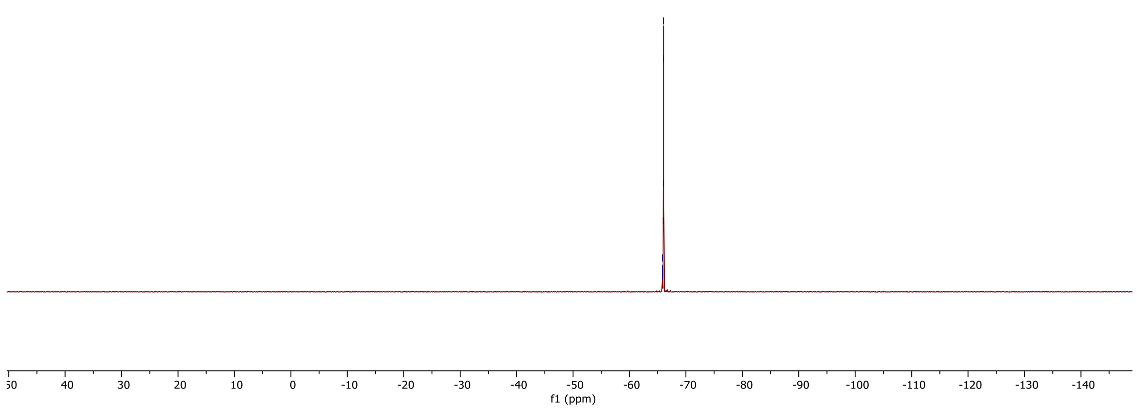

tert-Butyl (2_S_,3_S_)-2-(4-chlorophenyl)-2-(4,4,5,5-tetraethyl-1,3,2-dioxaborolan-2-yl)-3-(2,2,2-trifluoroethyl)azetidine-1-carboxylate (3i)

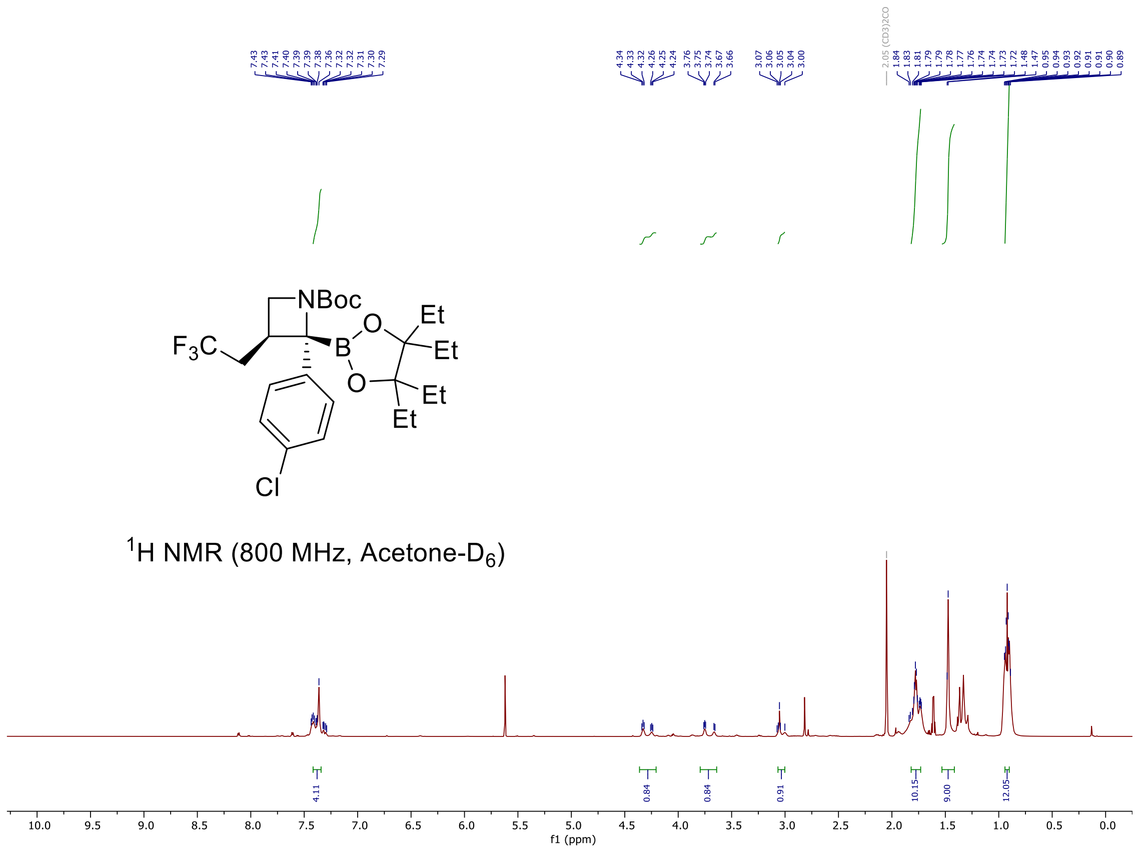

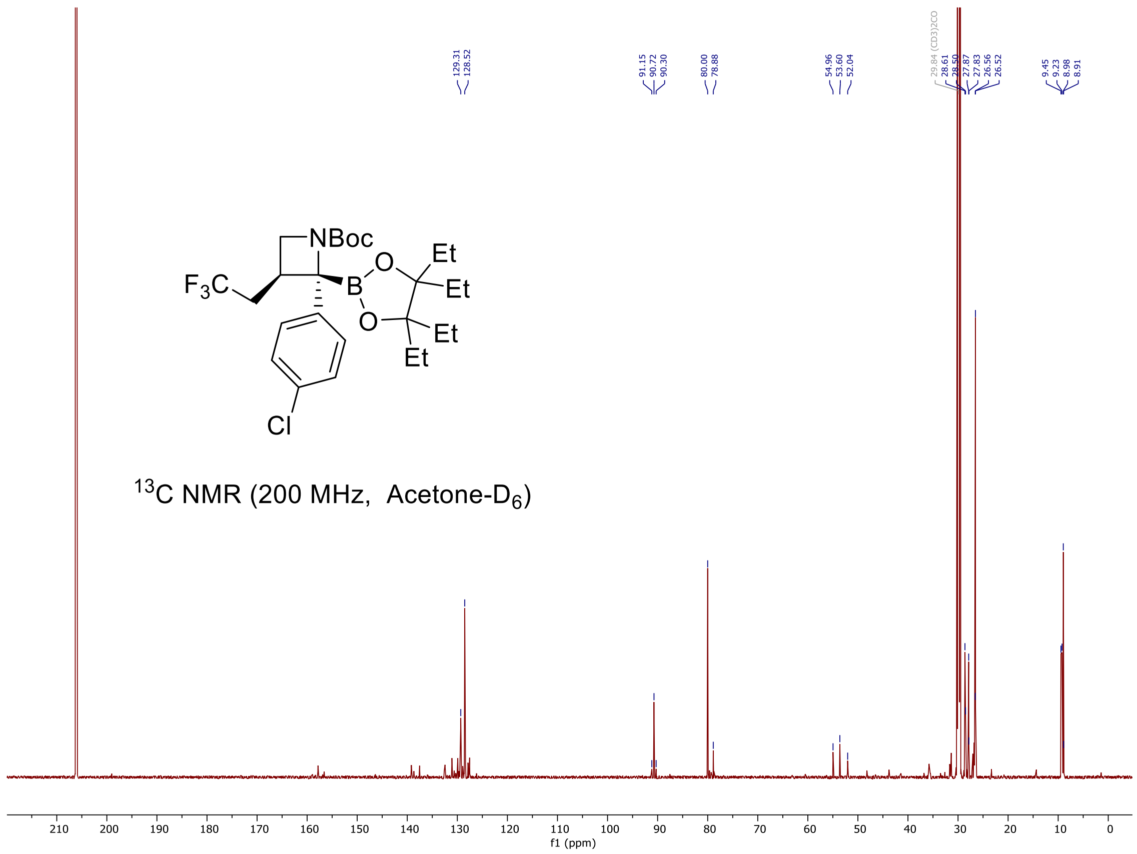

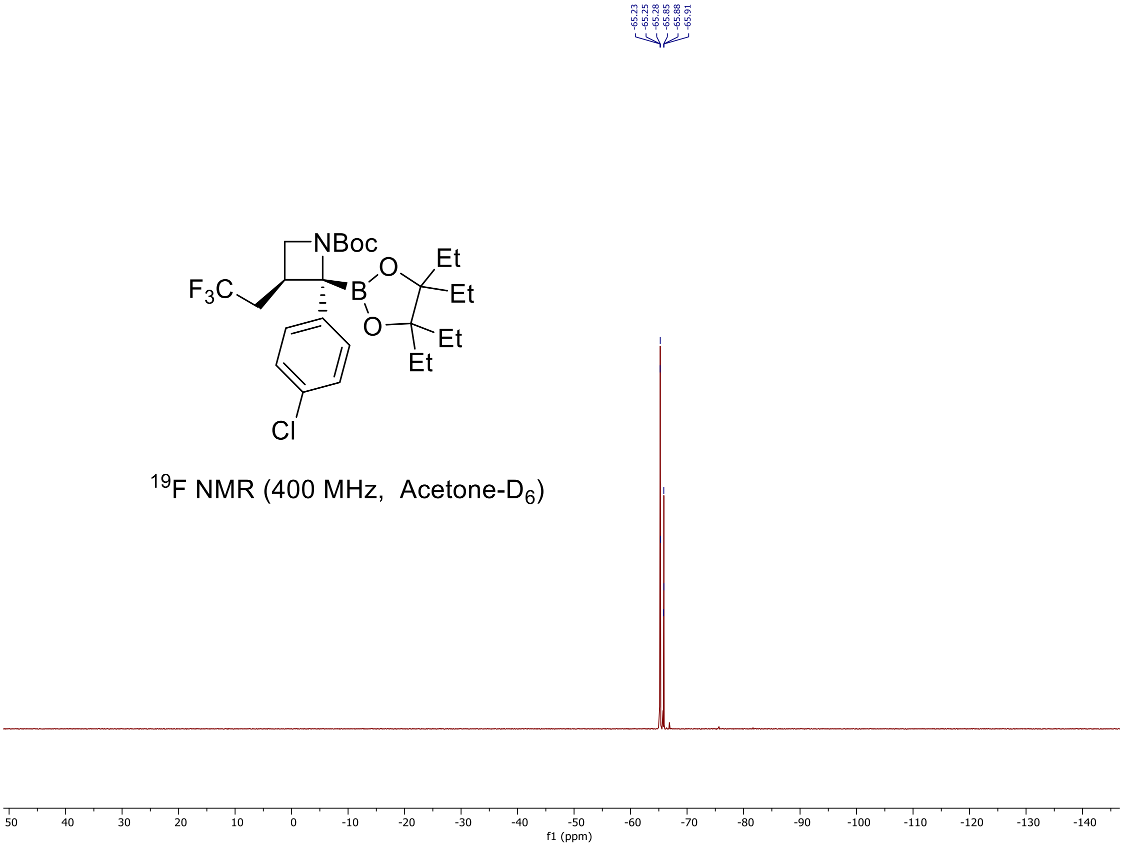

^{19}F NMR (400 MHz, Acetone-D_6)

***tert*-Butyl (2*R*,3*S*)-2-(4-fluorophenethyl)-2-(4,4,5,5-tetraethyl-1,3,2-dioxaborolan-2-yl)-3-(2,2,2-trifluoroethyl)azetidine-1-carboxylate (3j)**

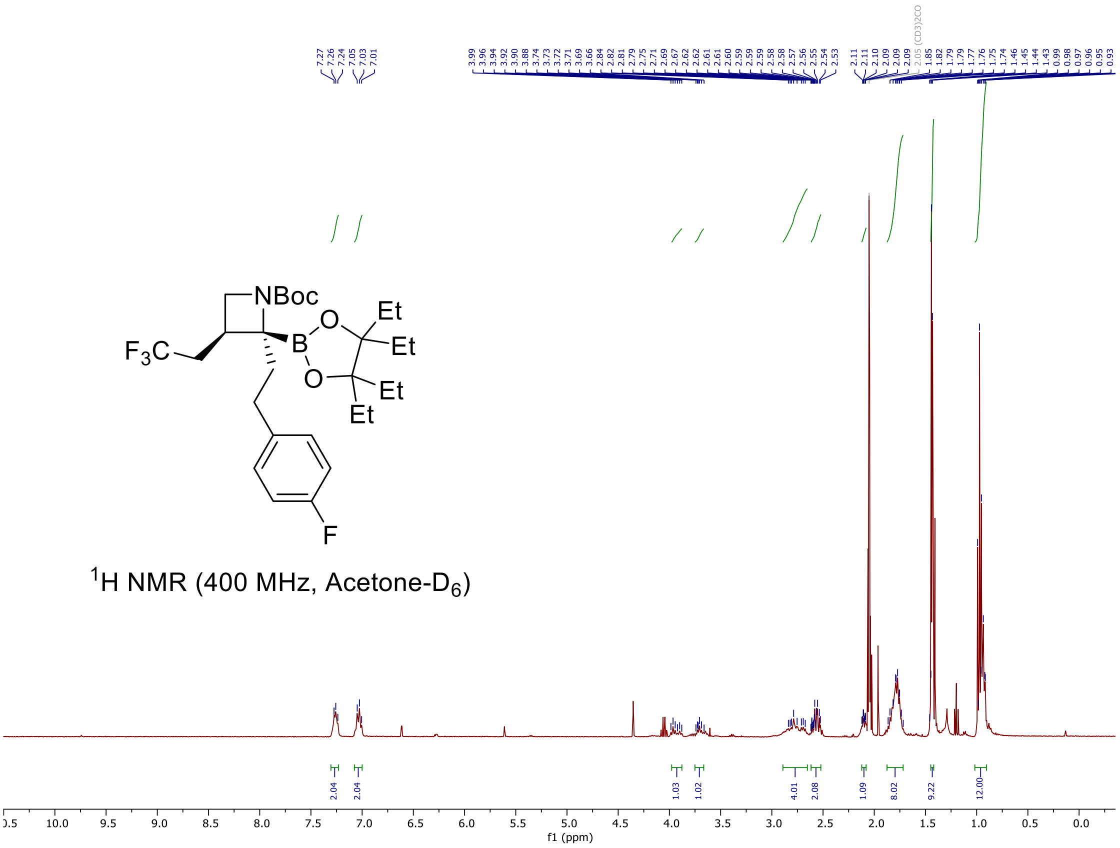

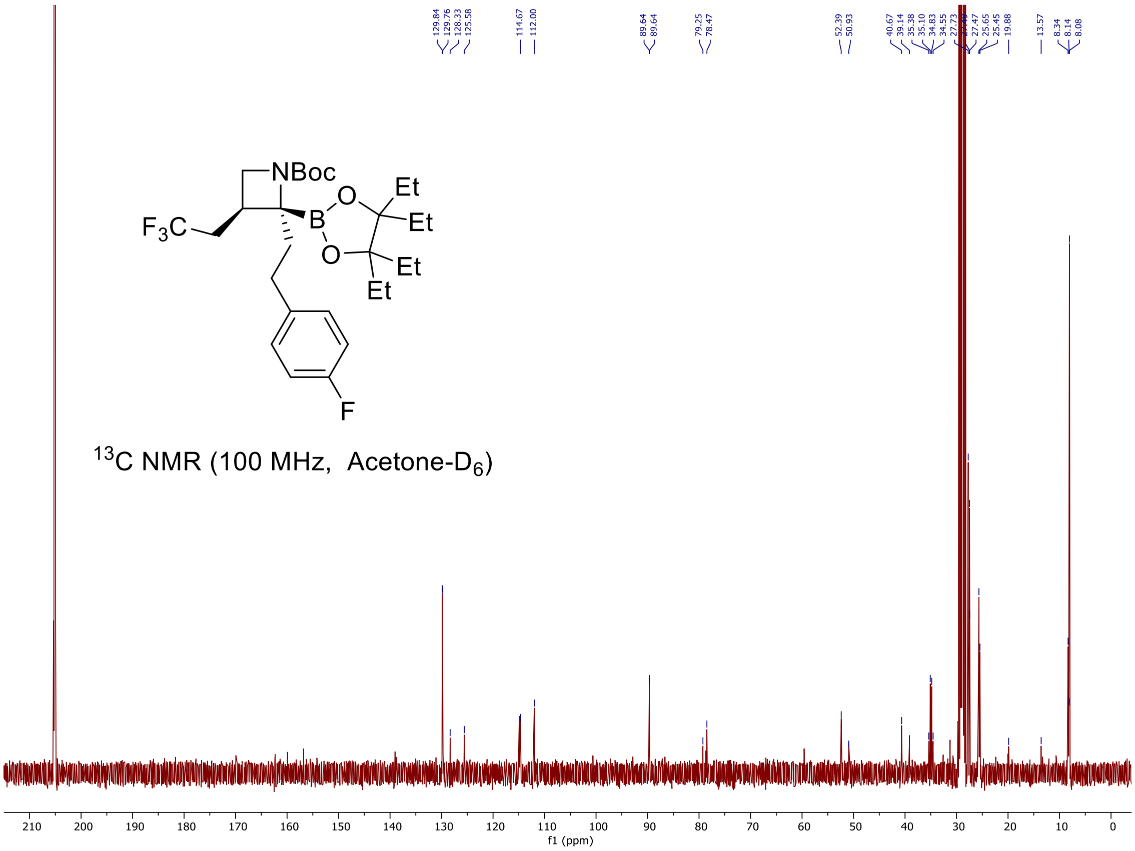

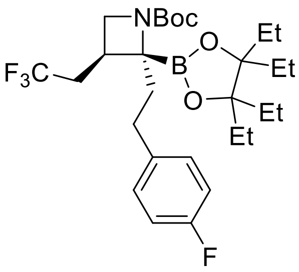

^{19}F NMR (400 MHz, Acetone-D_6)

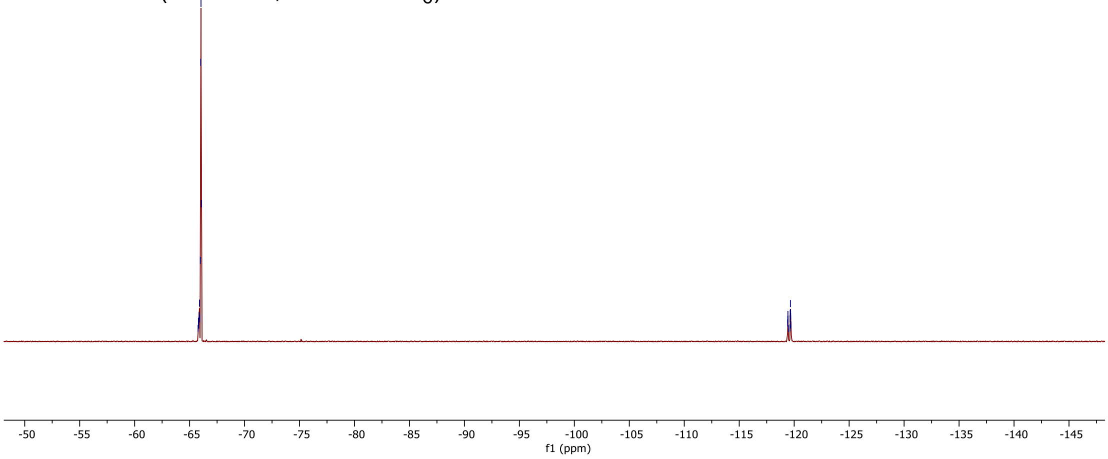

tert-Butyl (2_R_,3_S_)-2-(adamantan-1-ylmethyl)-3-(2-ethoxy-2-oxoethyl)-2-(4,4,5,5-tetraethyl-1,3,2-dioxaborolan-2-yl)azetidine-1-carboxylate (3k)

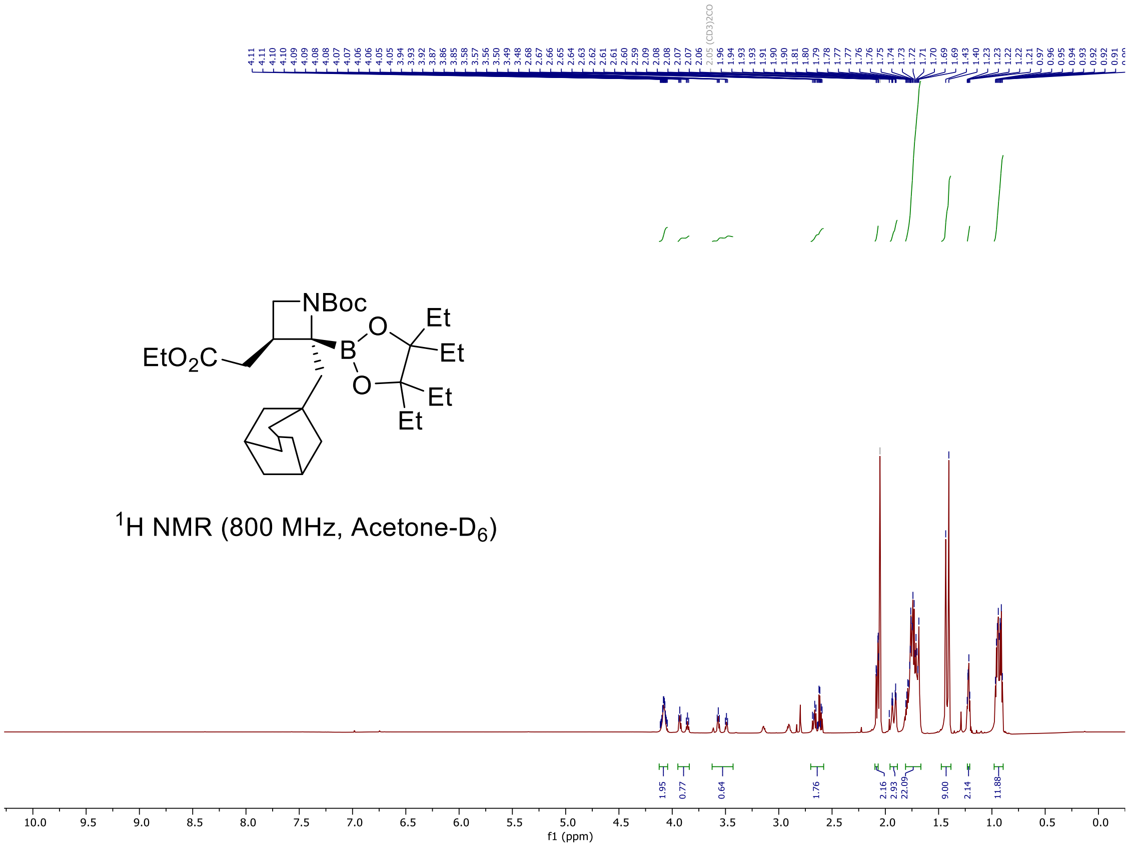

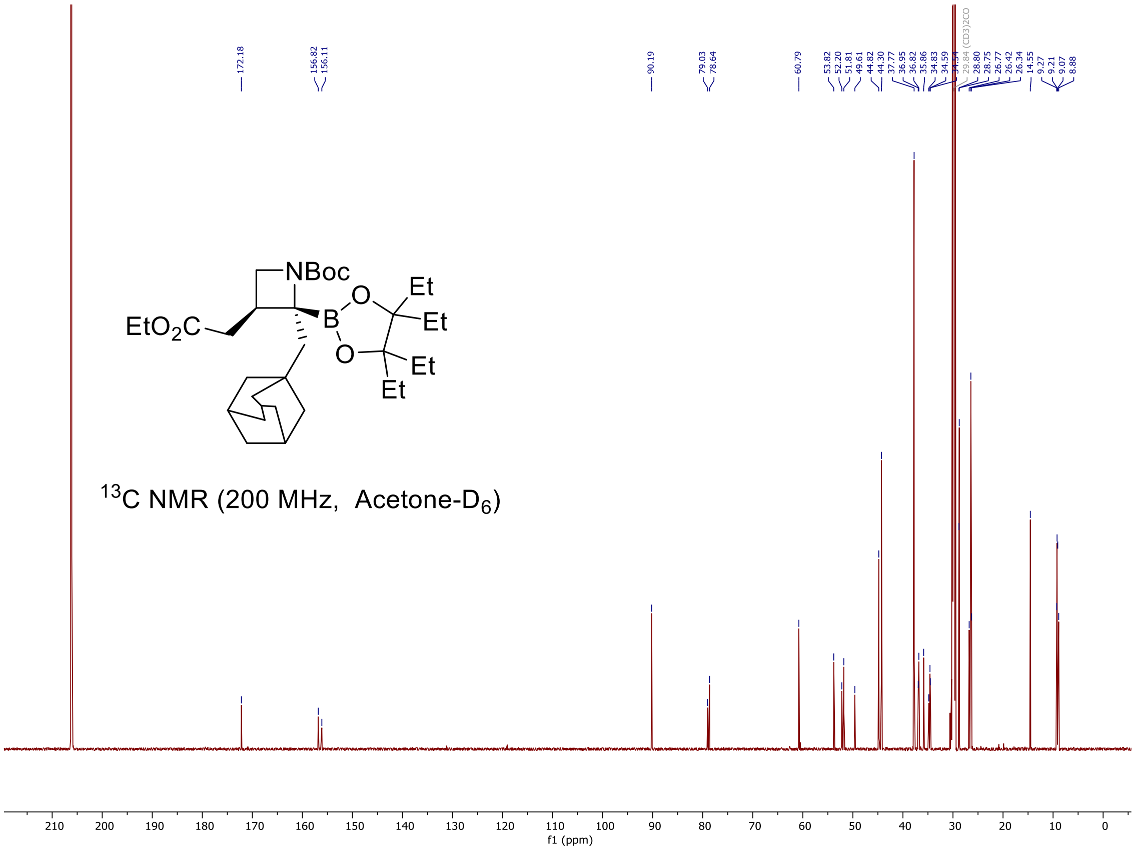

***tert*-Butyl (2*R*,3*S*)-3-(2-ethoxy-2-oxoethyl)-2-neopentyl-2-(4,4,5,5-tetraethyl-1,3,2-dioxaborolan-2-yl)azetidine-1-carboxylate (<u>3l</u>)**

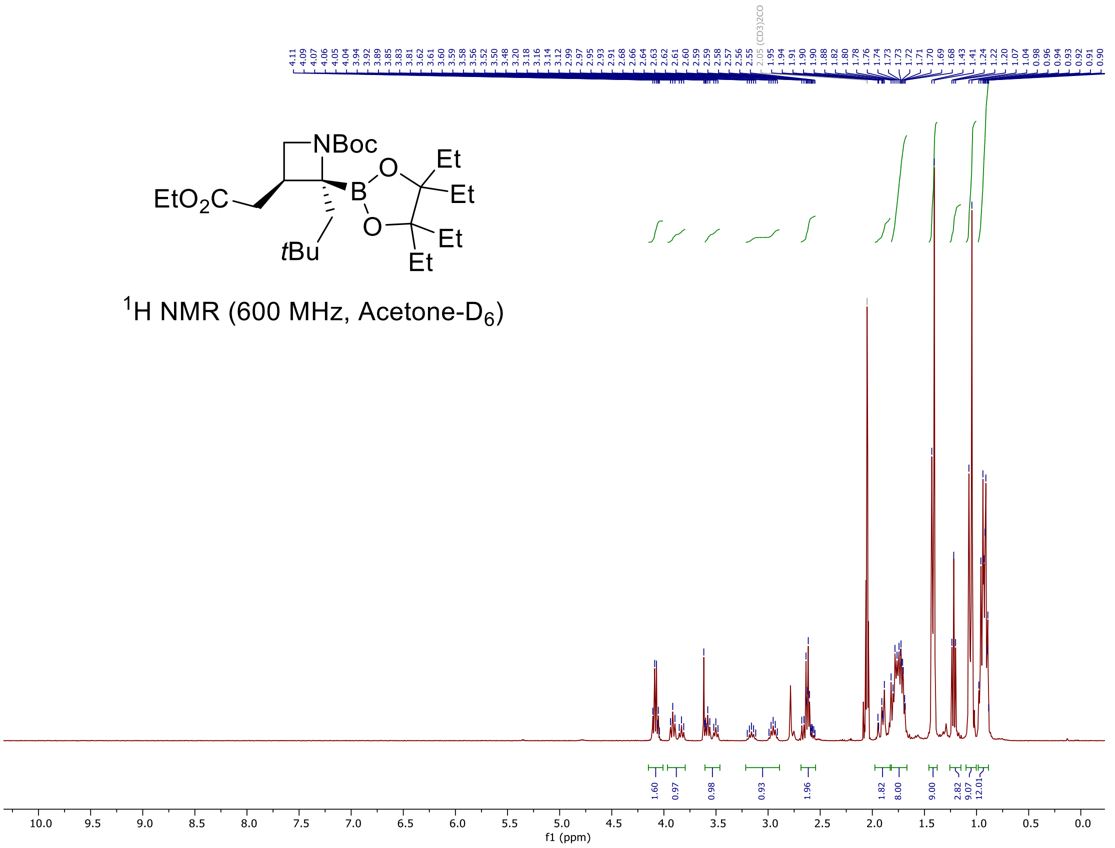

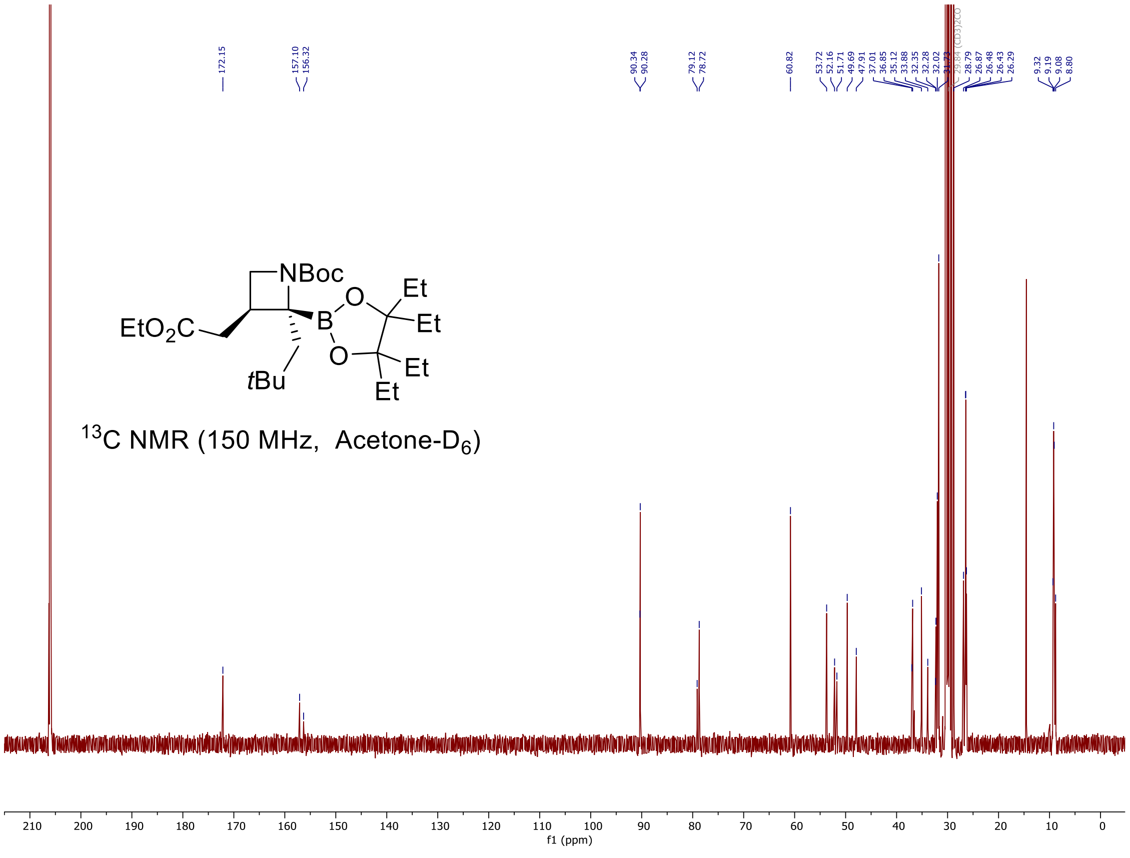

2-((2*R*,3*S*)-1-(*tert*-Butoxycarbonyl)-2-neopentyl-2-(4,4,5,5-tetraethyl-1,3,2-dioxaborolan-2-yl)azetidin-3-yl)acetic acid (3l')

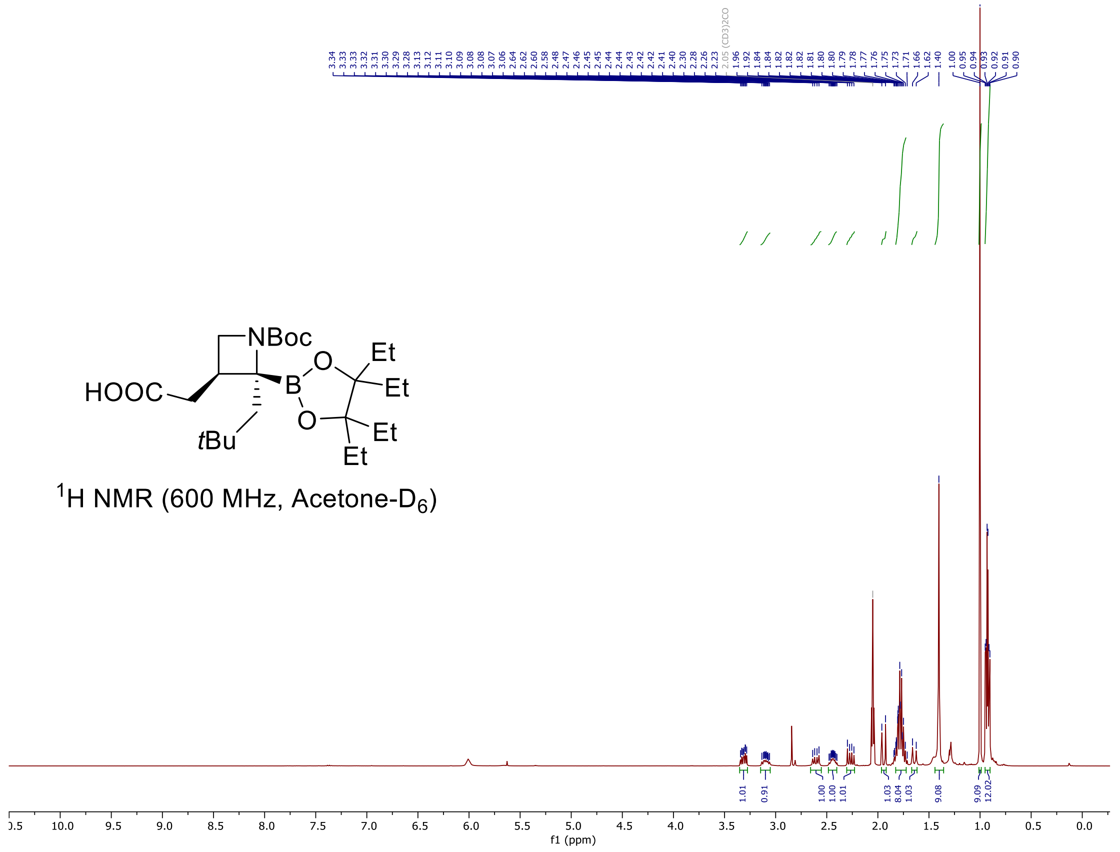

^{1}H NMR (600 MHz, Acetone-D$_6$)

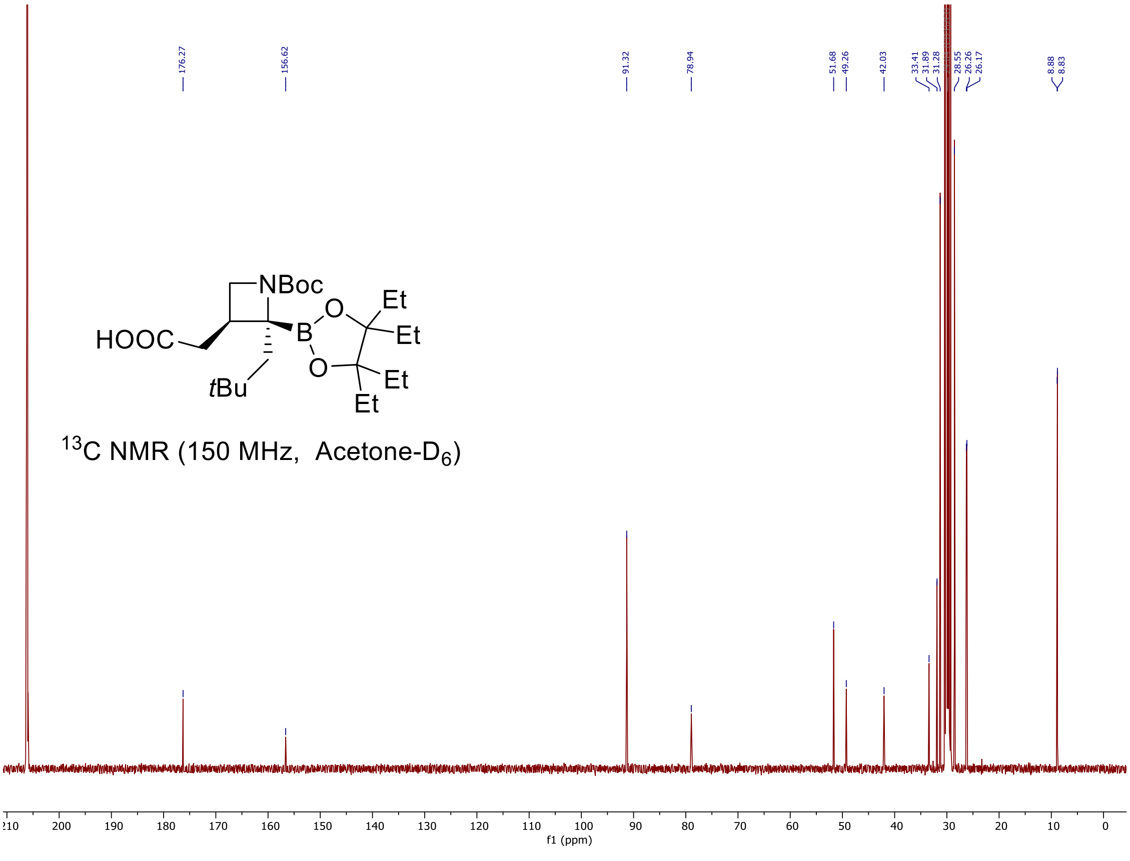

^{13}C NMR (150 MHz, Acetone-D$_6$)

tert-butyl (2_R_,3_S_)-3-(2-ethoxy-2-oxoethyl)-2-phenethyl-2-(4,4,5,5-tetraethyl-1,3,2-dioxaborolan-2-yl)azetidine-1-carboxylate (<u>3m</u>)

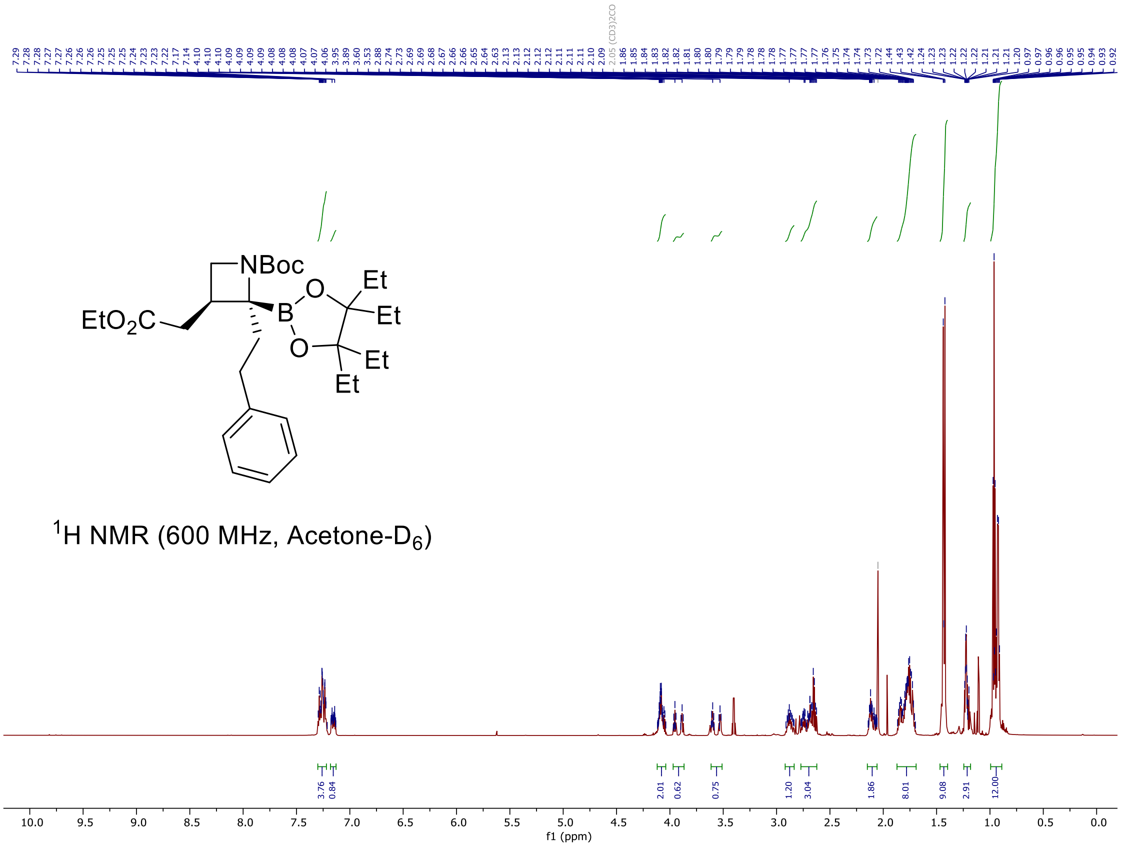

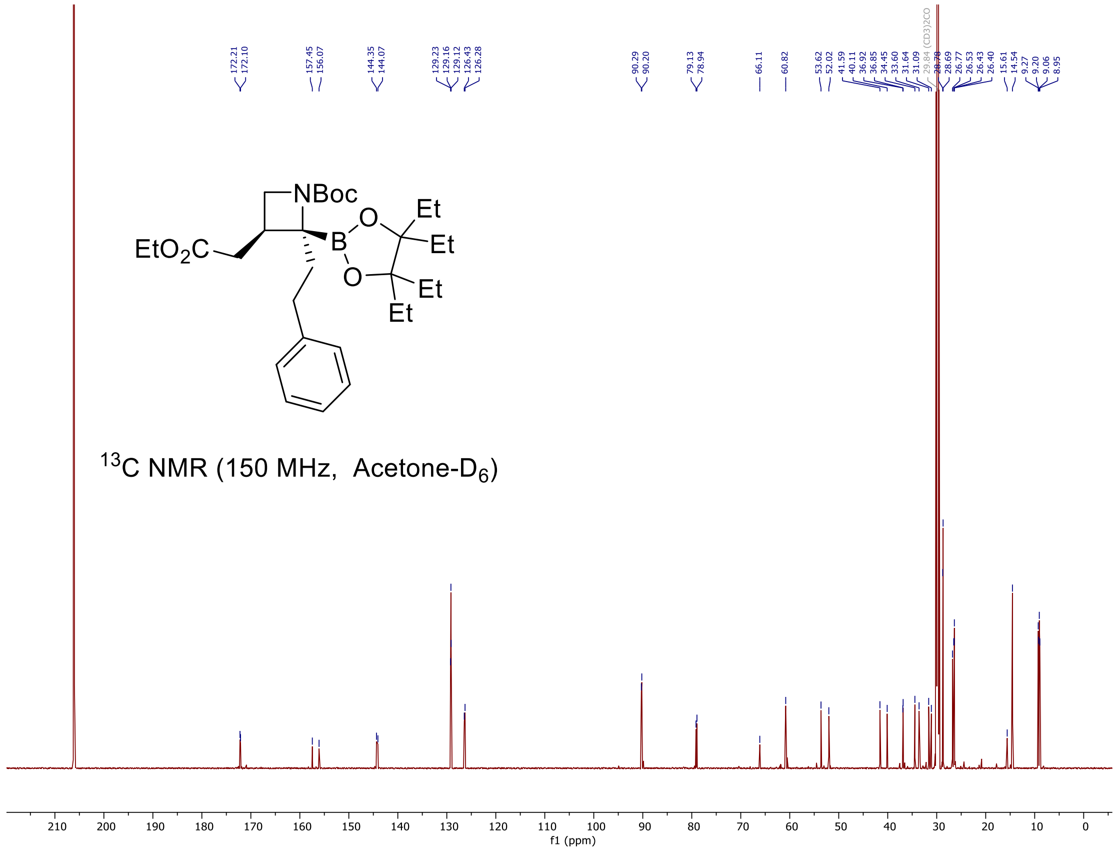

***tert*-Butyl (2*R*,3*S*)-3-(2-ethoxy-2-oxoethyl)-2-methyl-2-(4,4,5,5-tetraethyl-1,3,2-dioxaborolan-2-yl)azetidine-1-carboxylate (<u>3n</u>)**

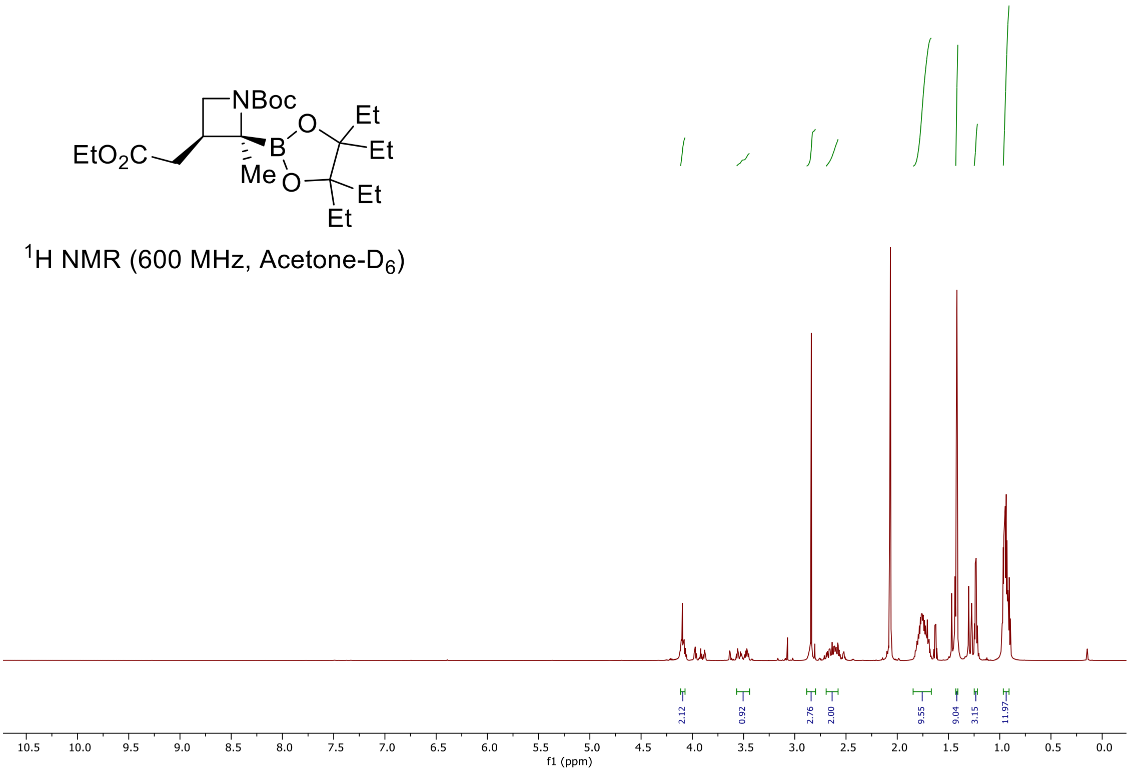

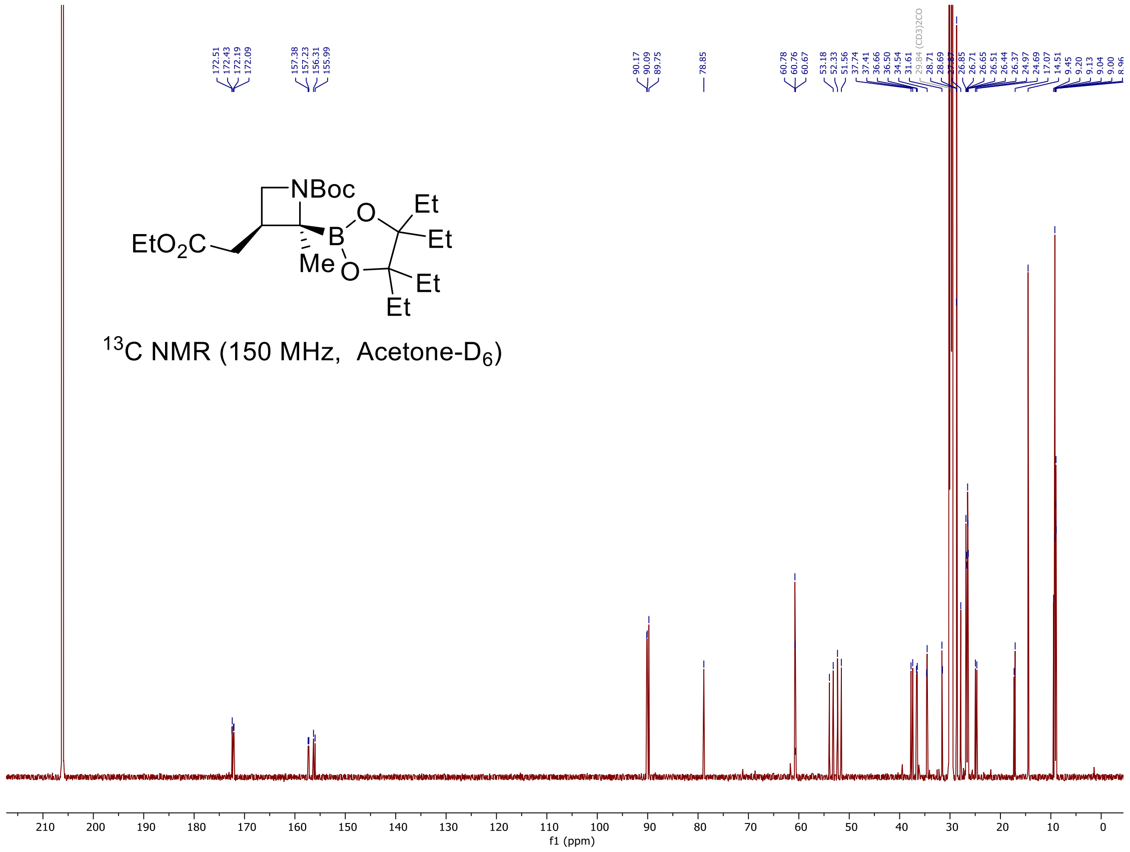

***tert*-Butyl (2*S*,3*S*)-2-(3-(adamantan-1-yl)-4-methoxyphenyl)-3-(2-ethoxy-2-oxoethyl)-2-(4,4,5,5-tetraethyl-1,3,2-dioxaborolan-2-yl)azetidine-1-carboxylate (3o)**

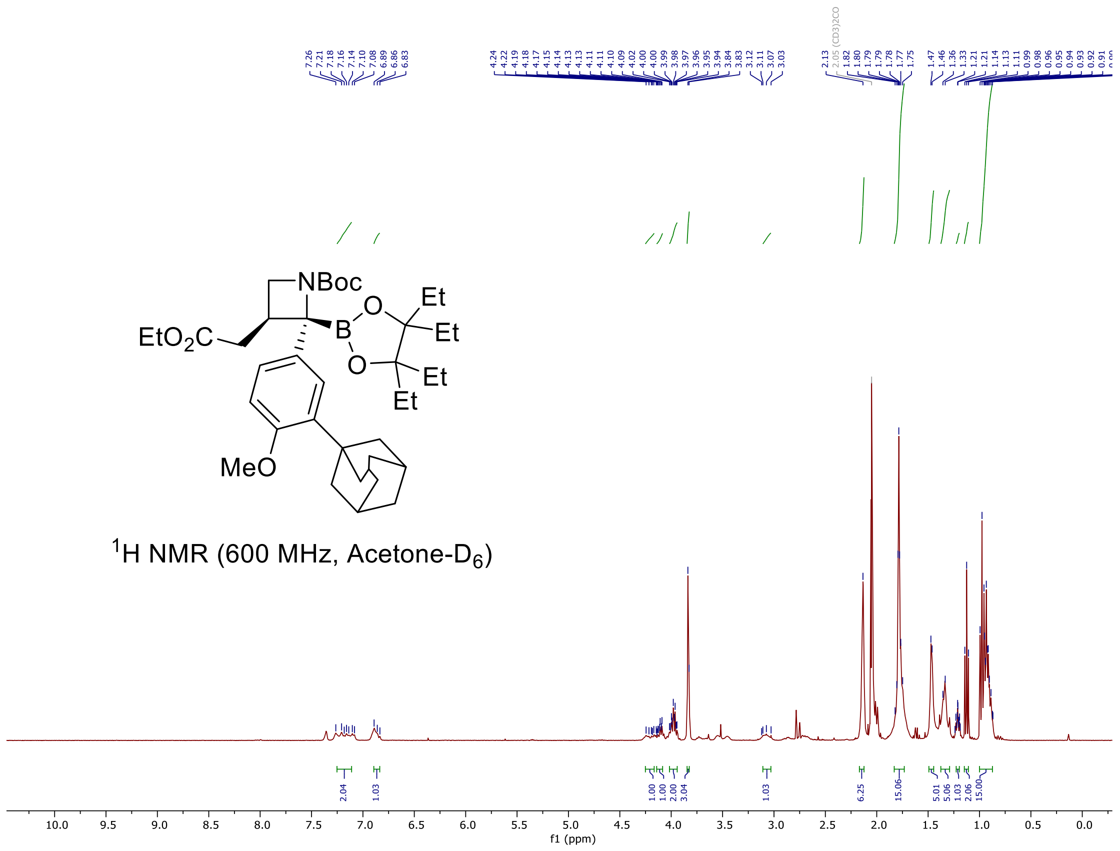

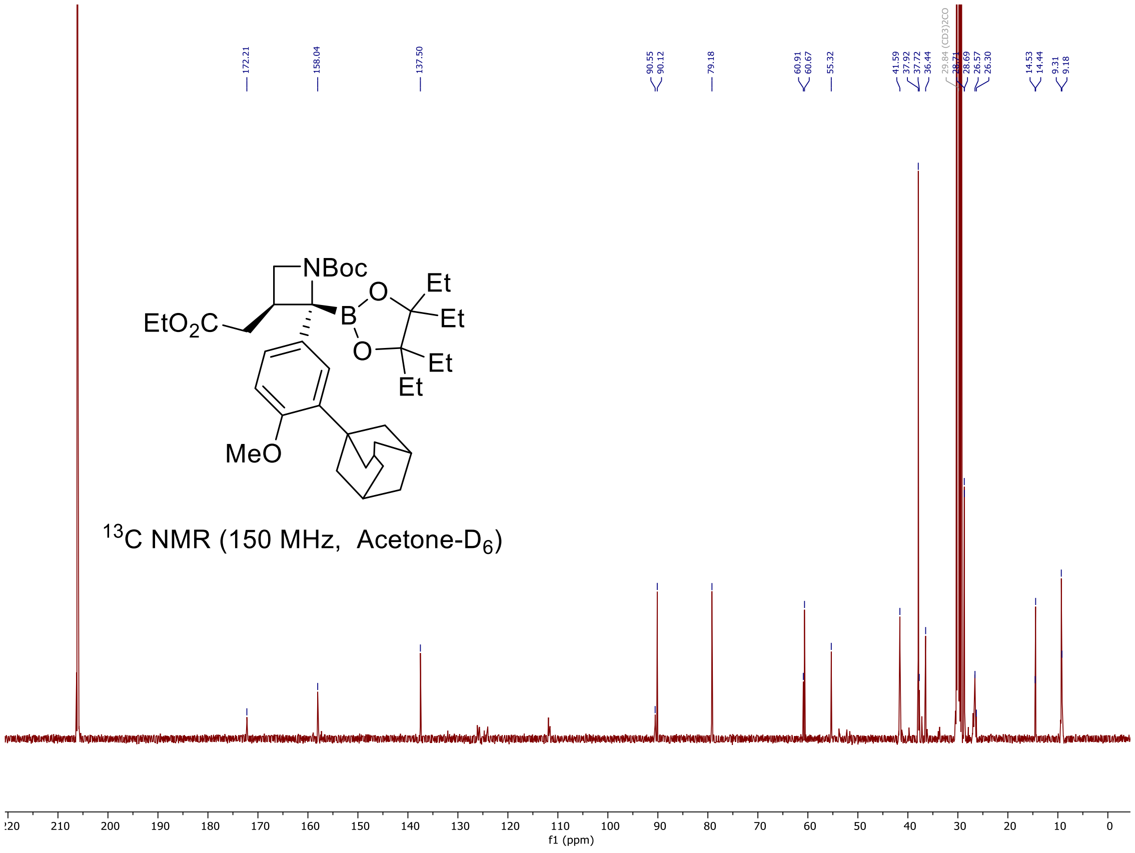

2-((1*S*,2*S*)-1-Butyl-2-(perfluorobutyl)cyclobutyl)-4,4,5,5-tetraethyl-1,3,2-dioxaborolane (<u>6a</u>)

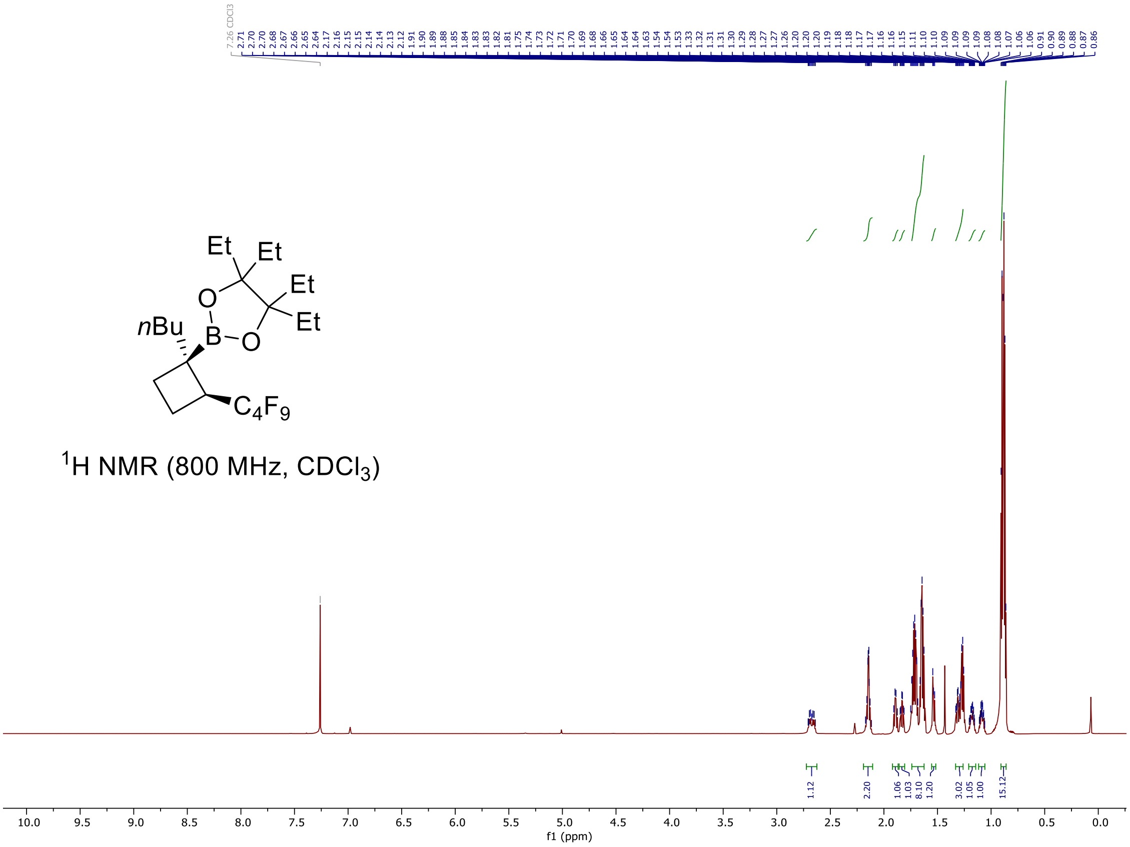

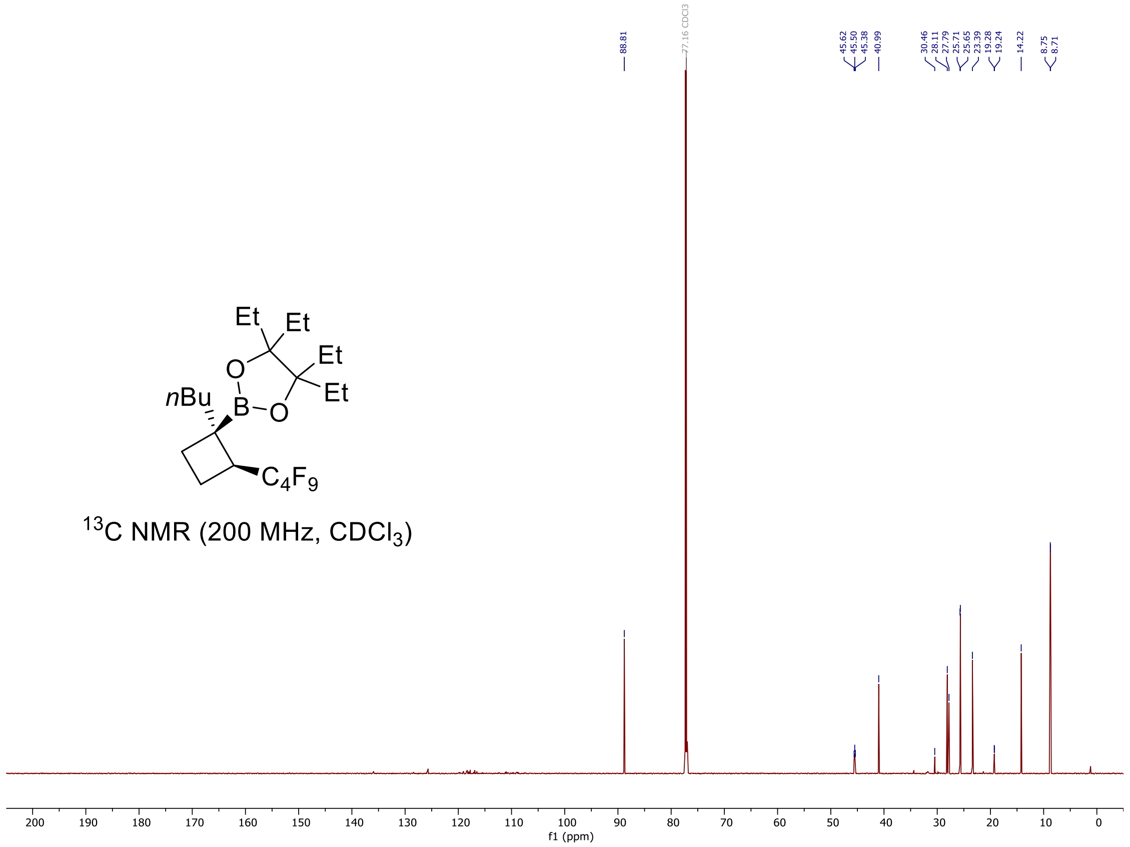

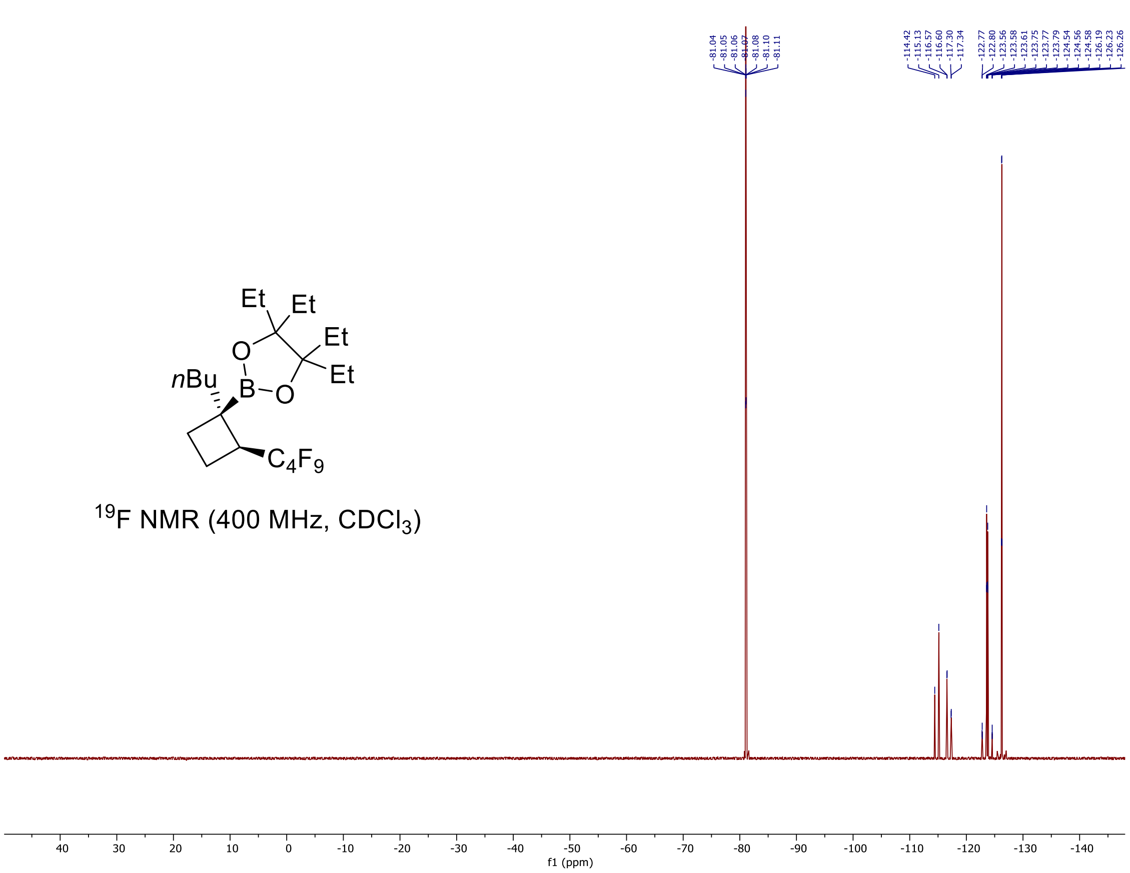

^{19}F NMR (400 MHz, $CDCl_3$)

2-((1*S*,2*S*)-1-(3-chloro-5-fluorophenyl)-2-(trifluoromethyl)cyclobutyl)-4,4,5,5-tetraethyl-1,3,2-dioxaborolane (6b)

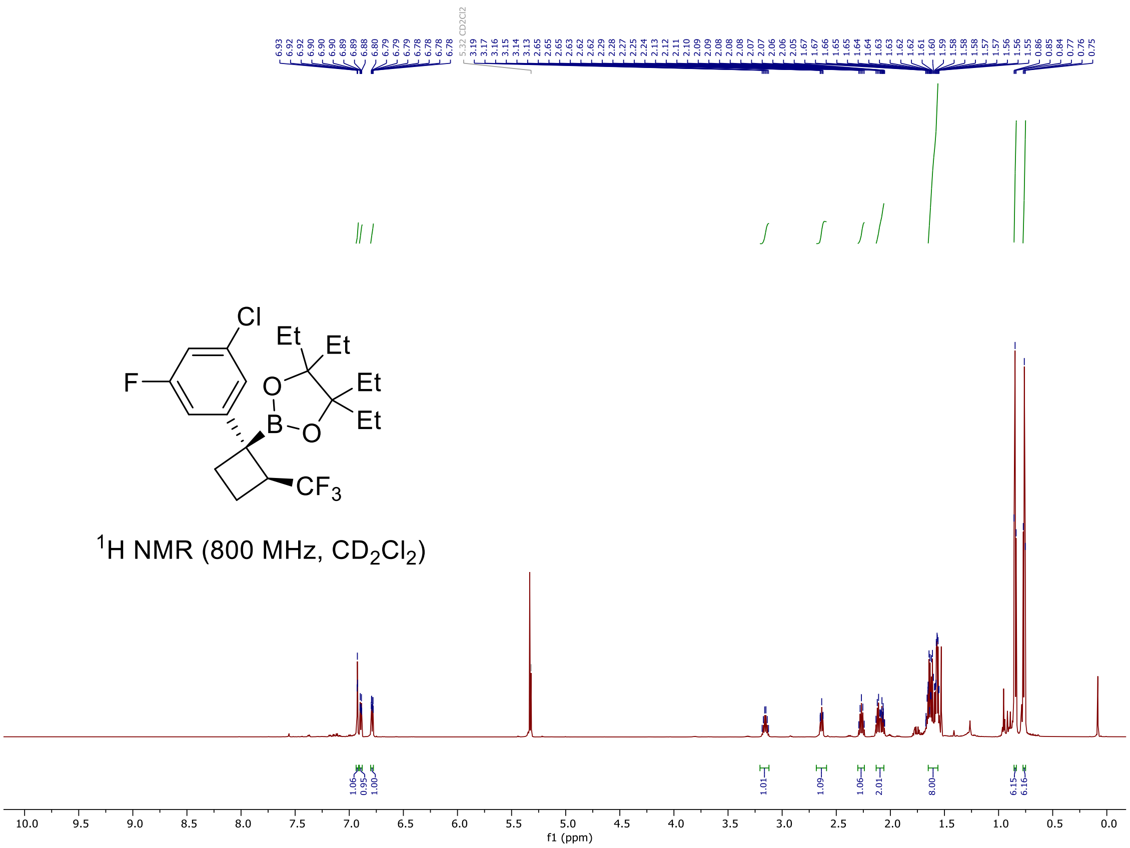

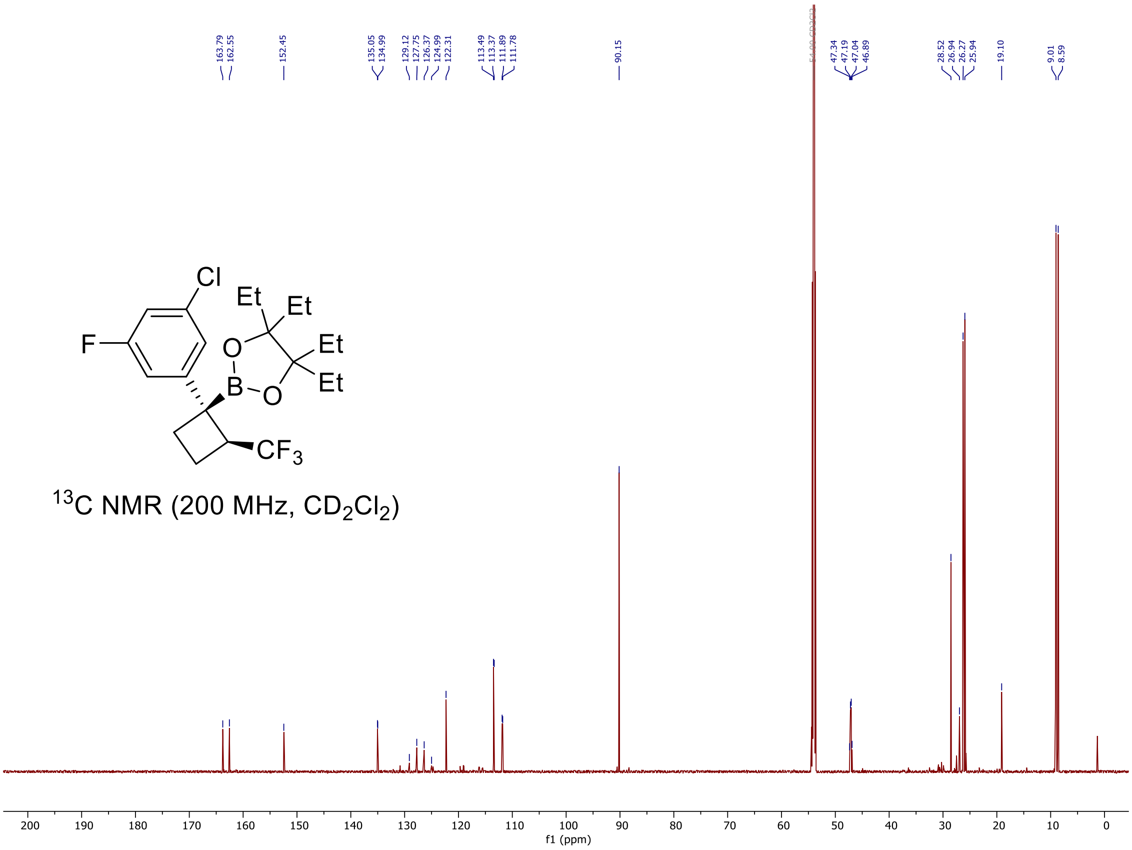

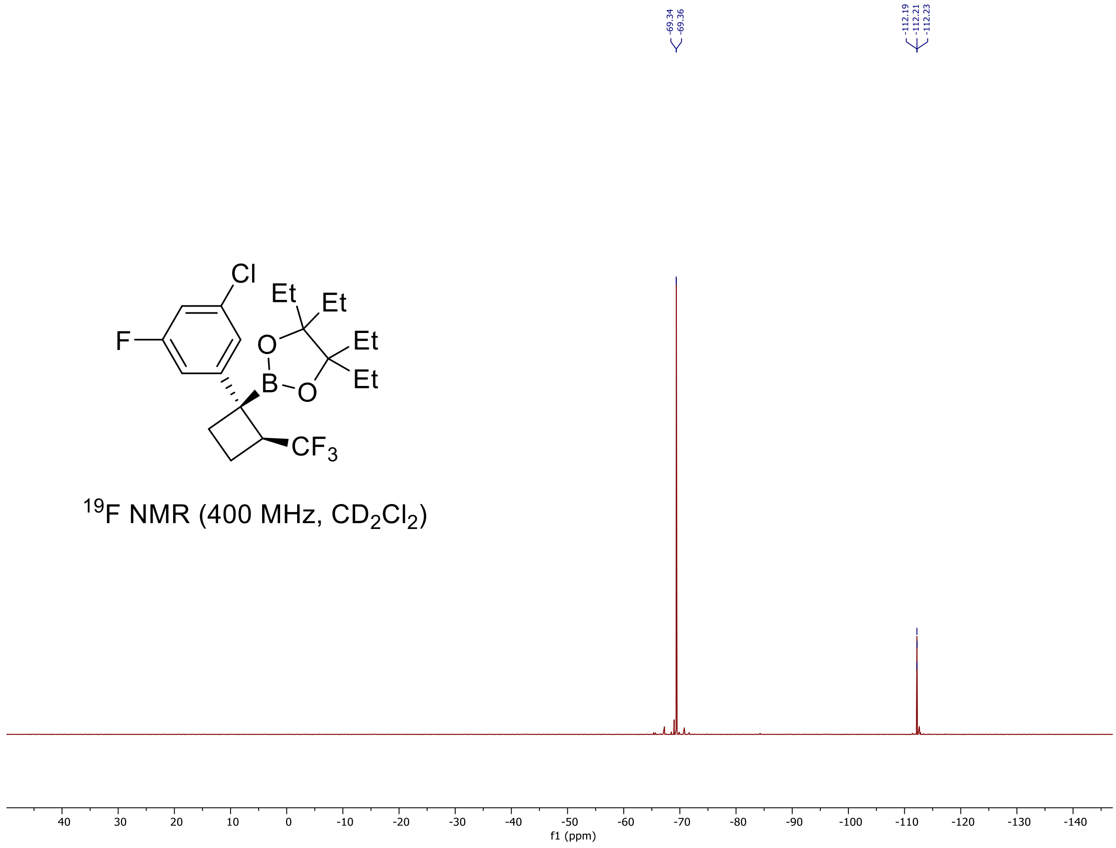

^{19}F NMR (400 MHz, CD_2Cl_2)

Ethyl 2-((1*R*,2*R*)-2-cyclopropyl-2-(4,4,5,5-tetraethyl-1,3,2-dioxaborolan-2-yl)cyclobutyl)acetate (6c)

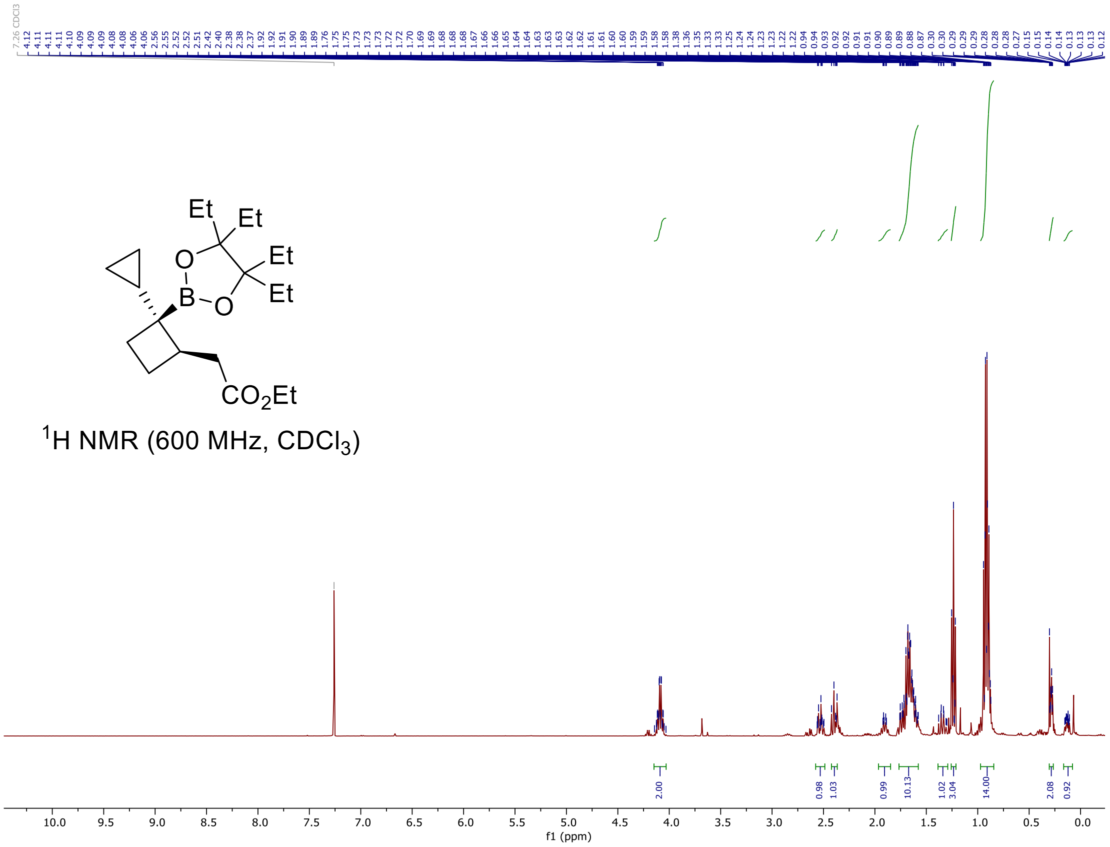

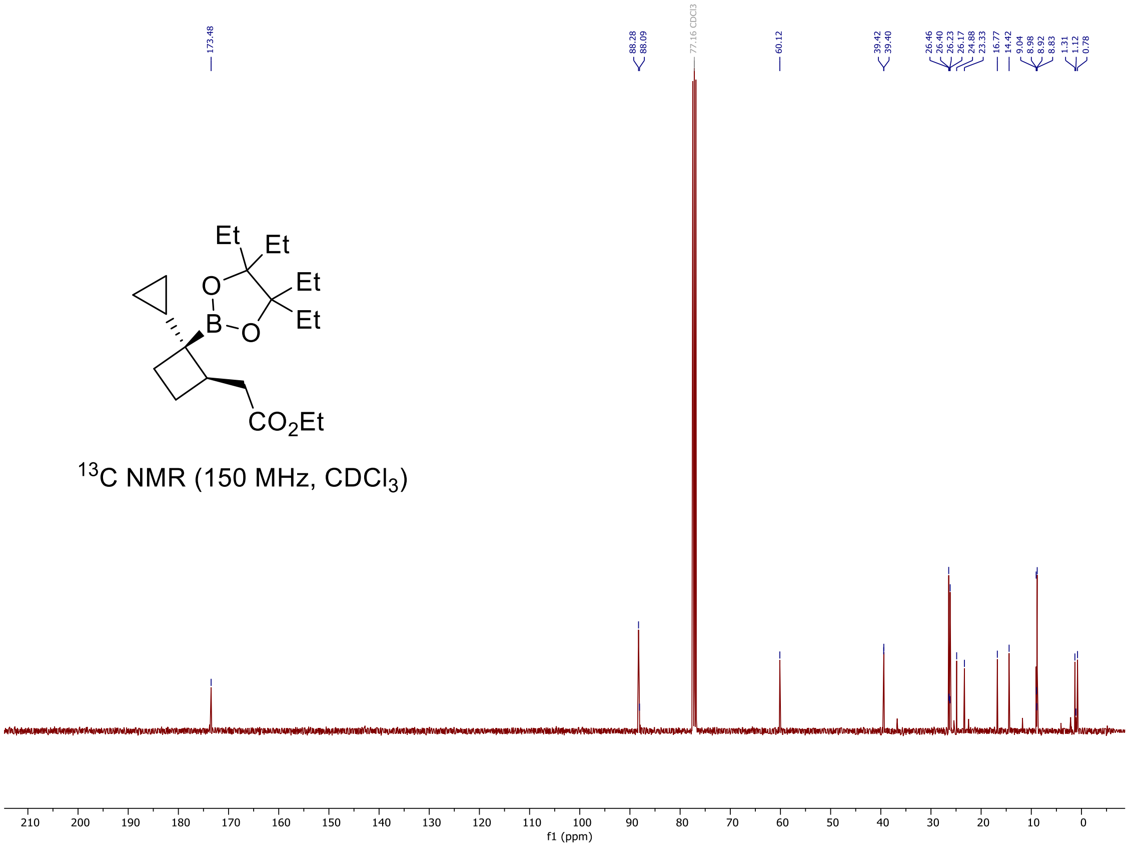

Ethyl 2-((1*R*,2*S*)-2-(4-fluorophenyl)-2-(4,4,5,5-tetraethyl-1,3,2-dioxaborolan-2-yl)cyclobutyl)acetate (6d)

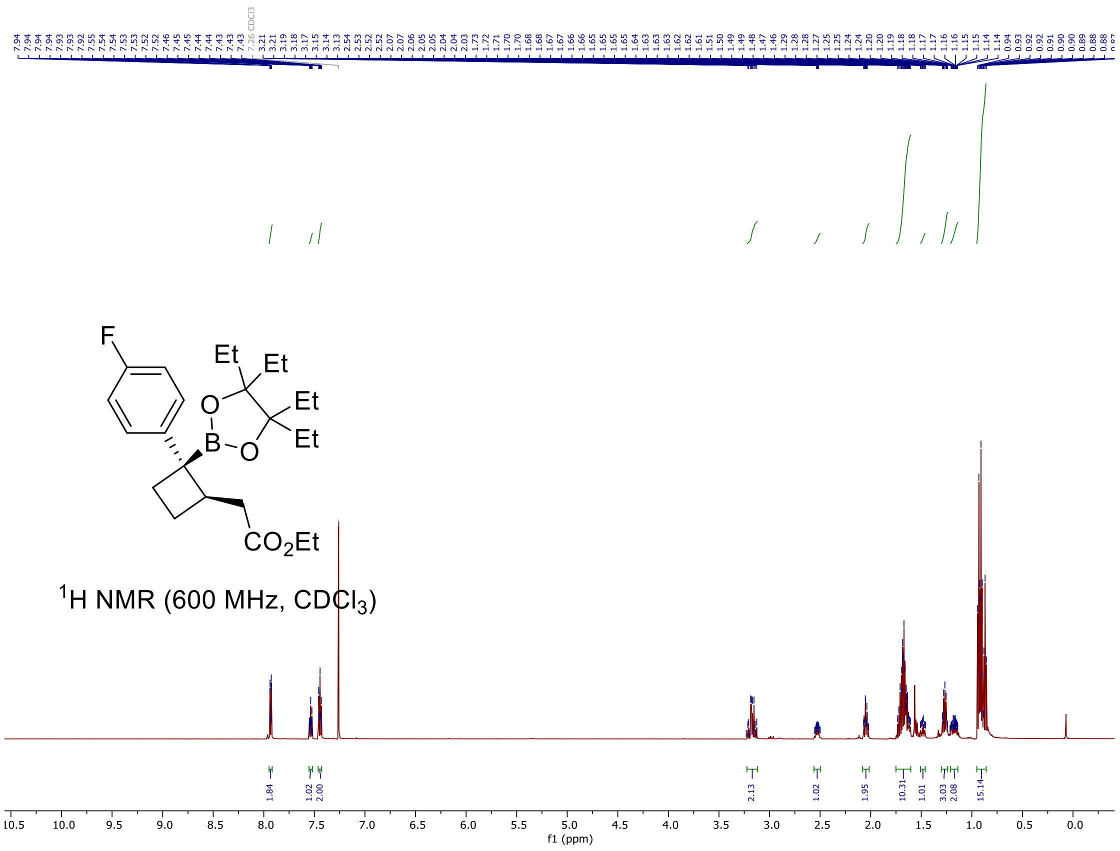

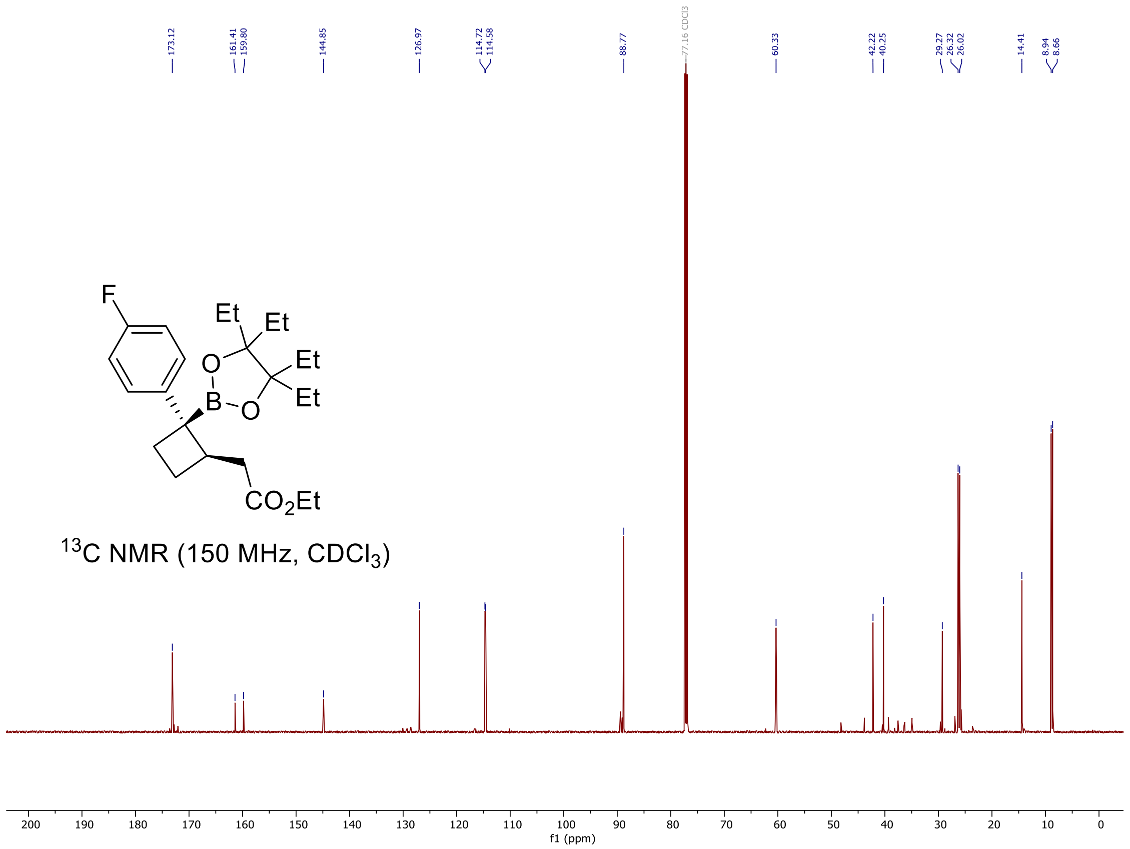

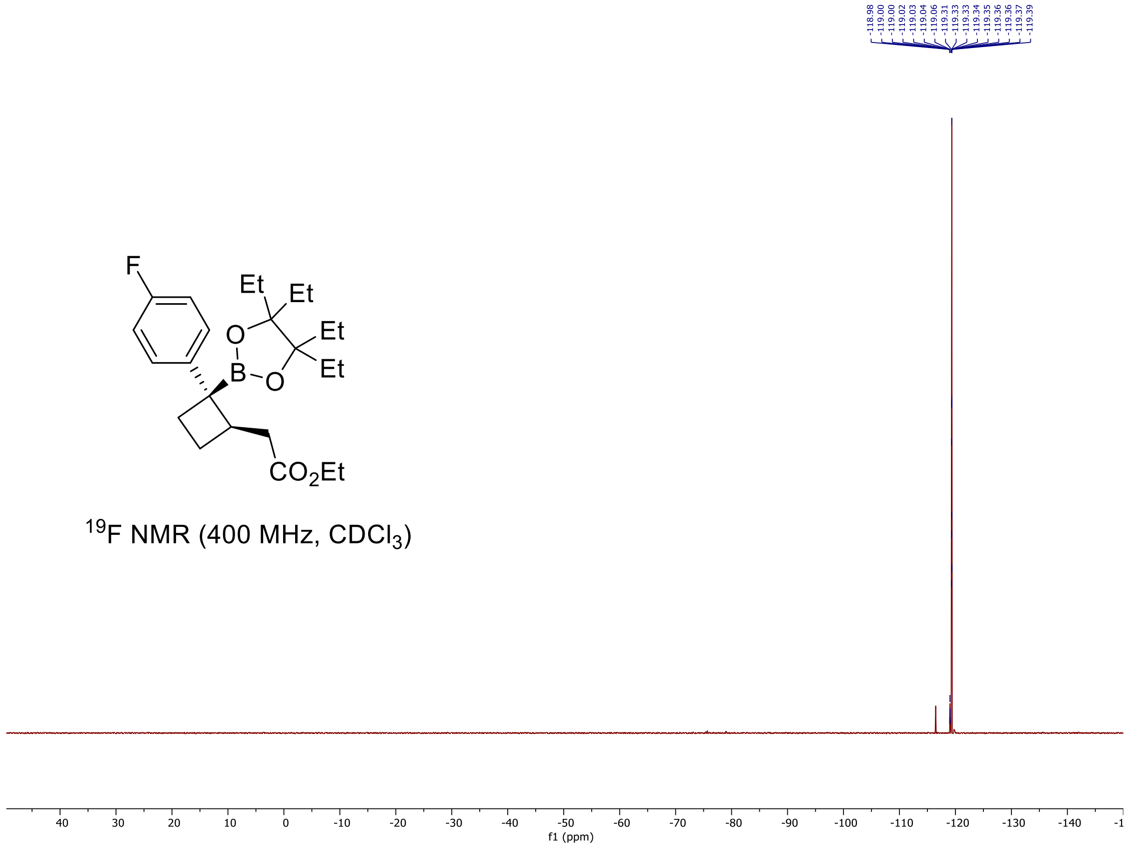

^{19}F NMR (400 MHz, $CDCl_3$)

2-((1*R*,2*S*)-2-Butyl-2-(4,4,5,5-tetraethyl-1,3,2-dioxaborolan-2-yl)cyclobutyl)-1-phenylethan-1-one (6e)

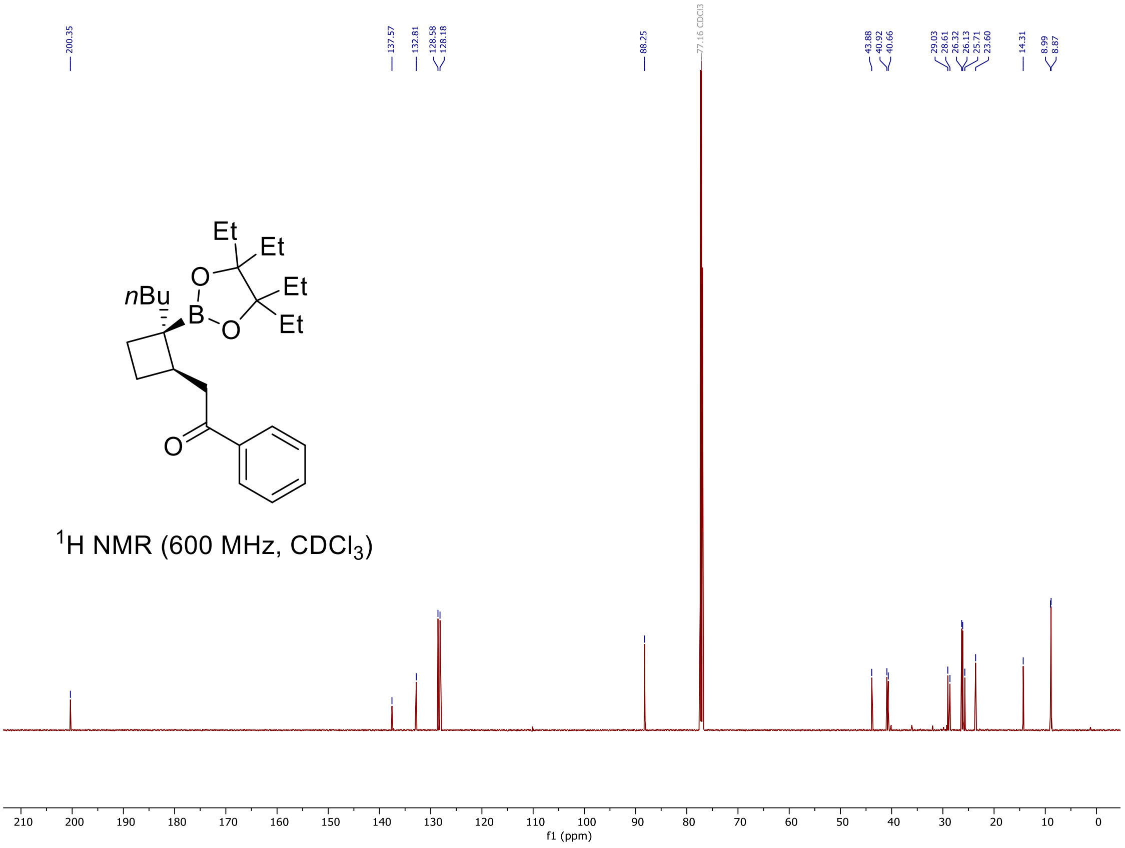

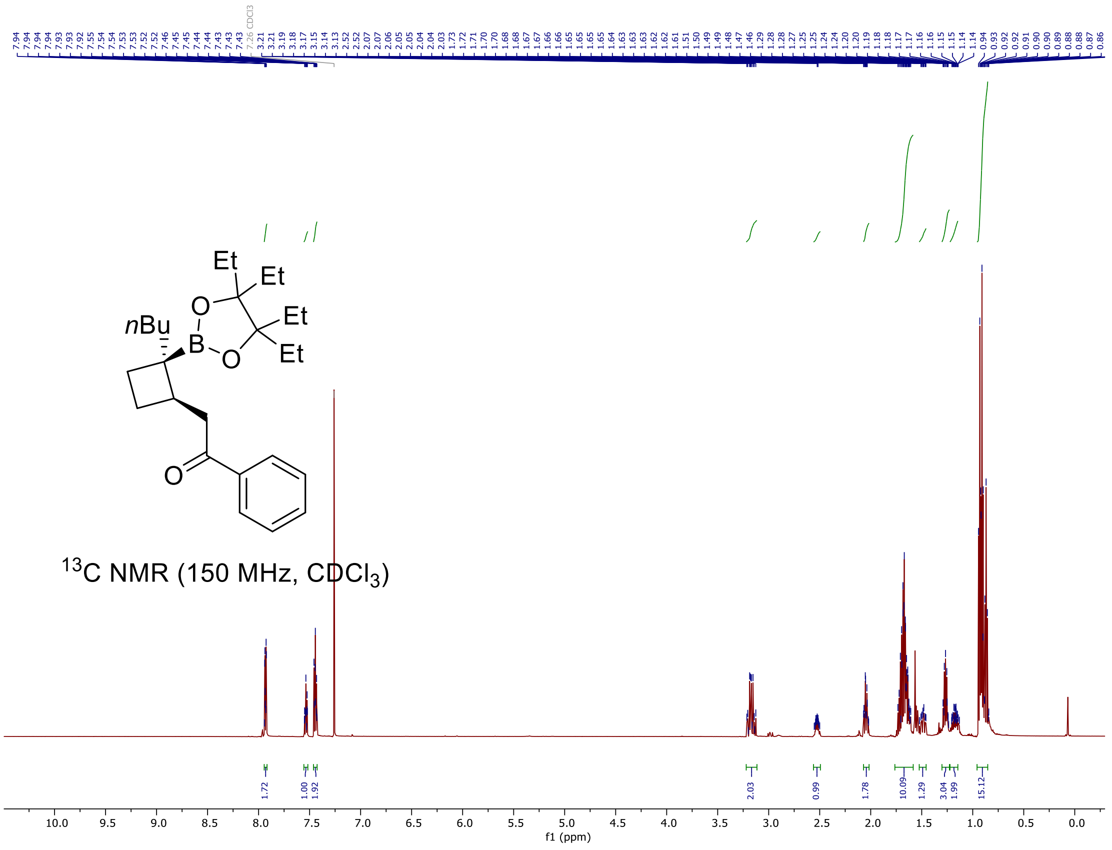

2-((1*R*,2*S*)-2-(4,4,5,5-tetraethyl-1,3,2-dioxaborolan-2-yl)-2-(4-(trifluoromethyl)phenyl)cyclobutyl)acetamide (6f)

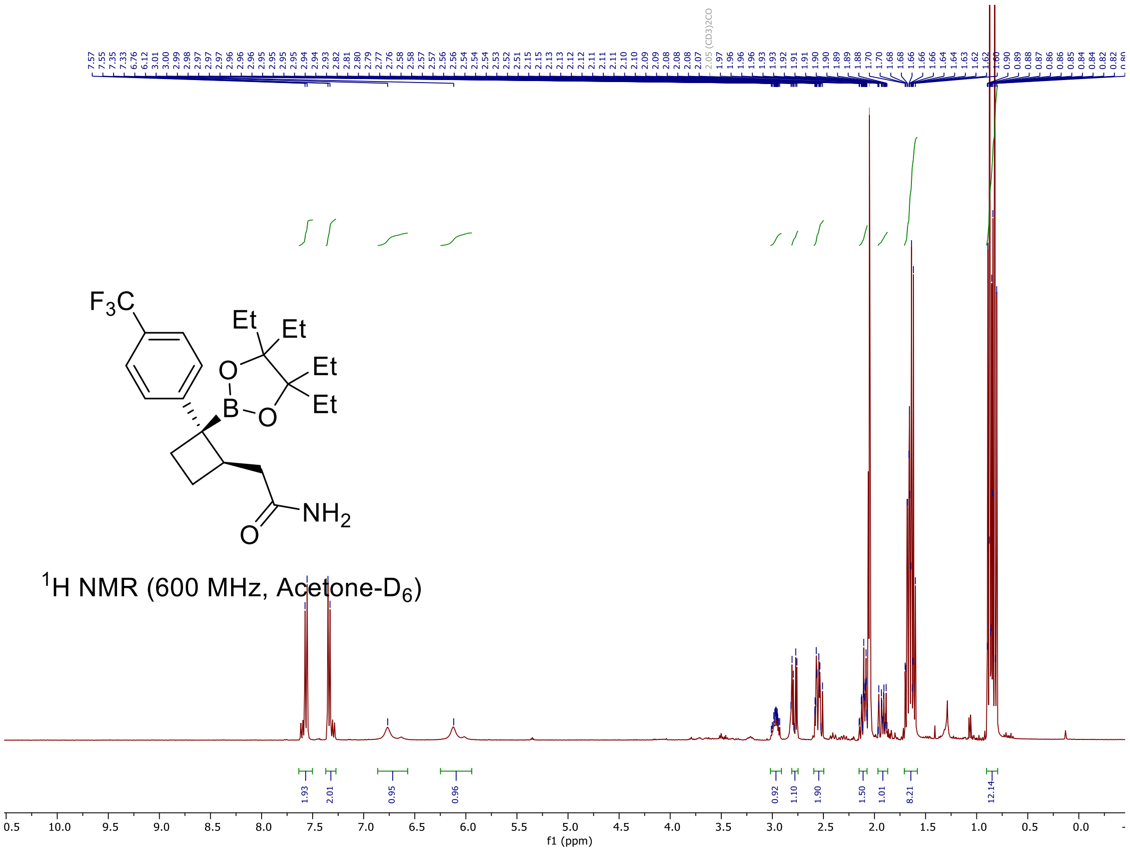

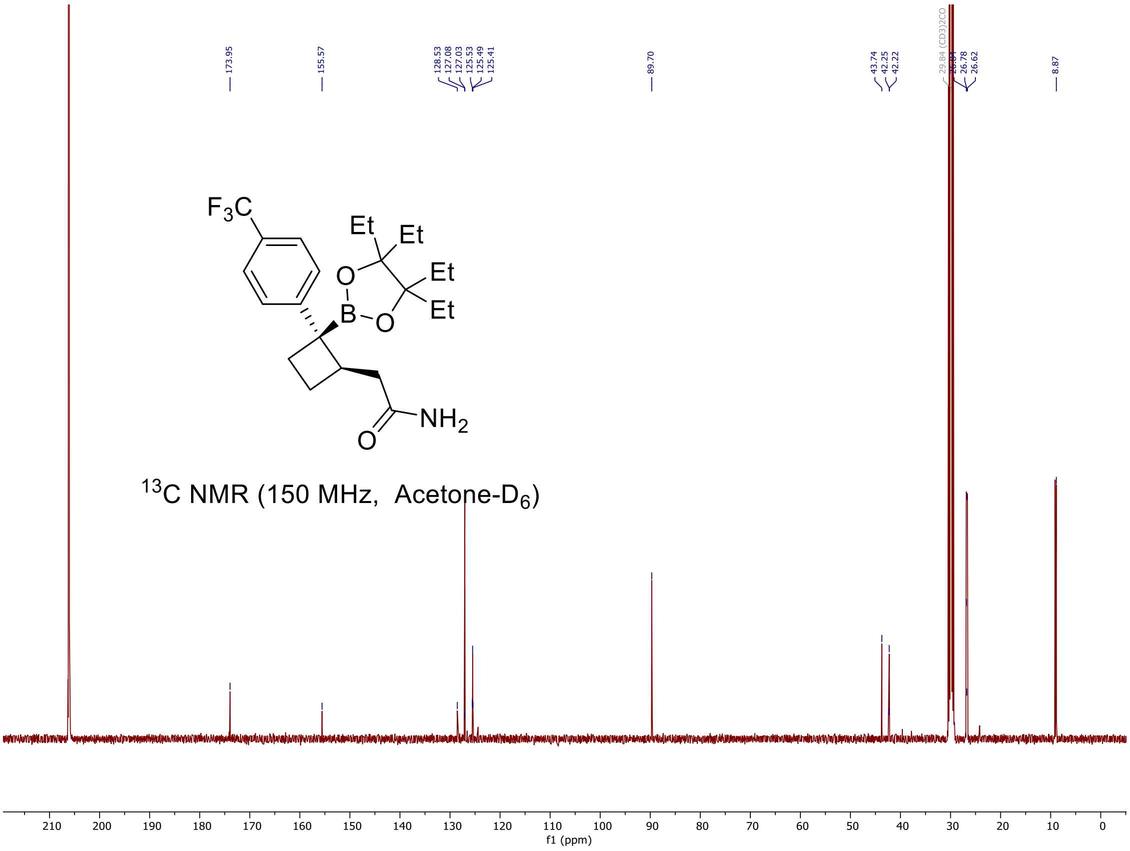

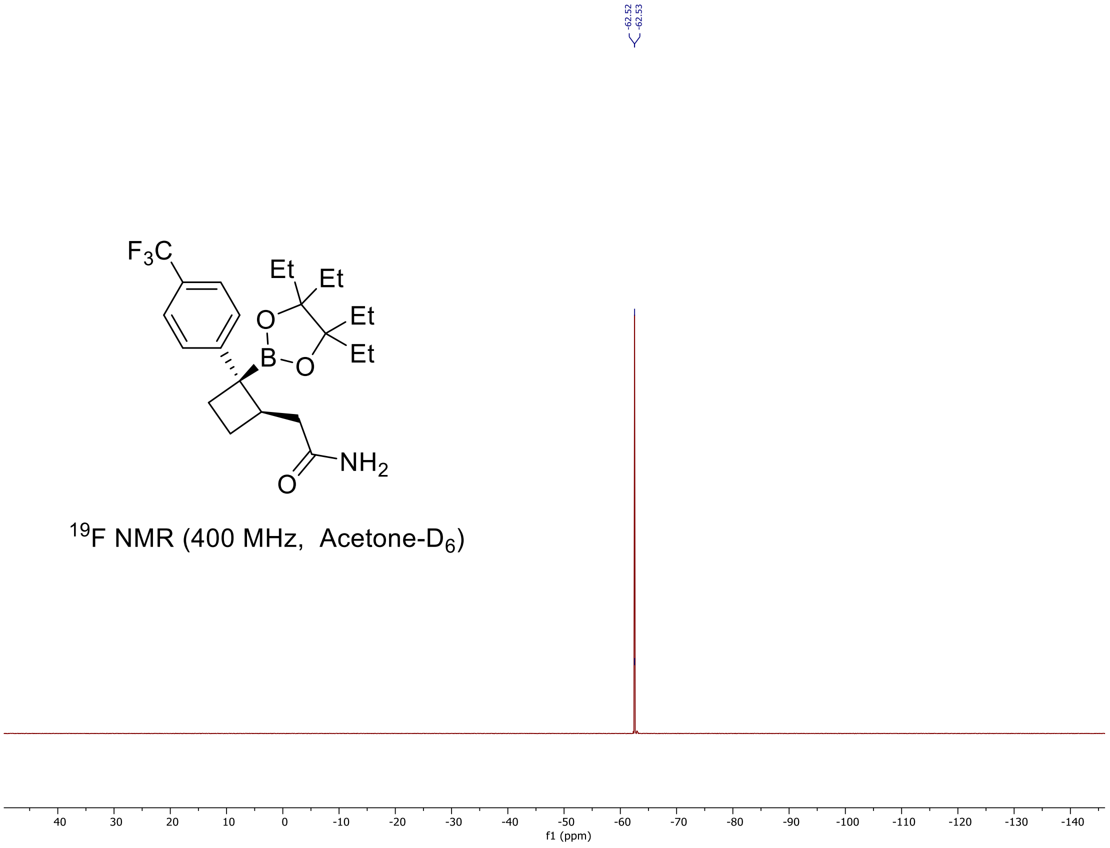

^{19}F NMR (400 MHz, Acetone-D_6)

Ethyl 2-((1*R*,2*S*)-2-(3-((5-(4-fluorophenyl)thiophen-2-yl)methyl)-4-methylphenyl)-2-(4,4,5,5-tetraethyl-1,3,2-dioxaborolan-2-yl)cyclobutyl)acetate (6g)

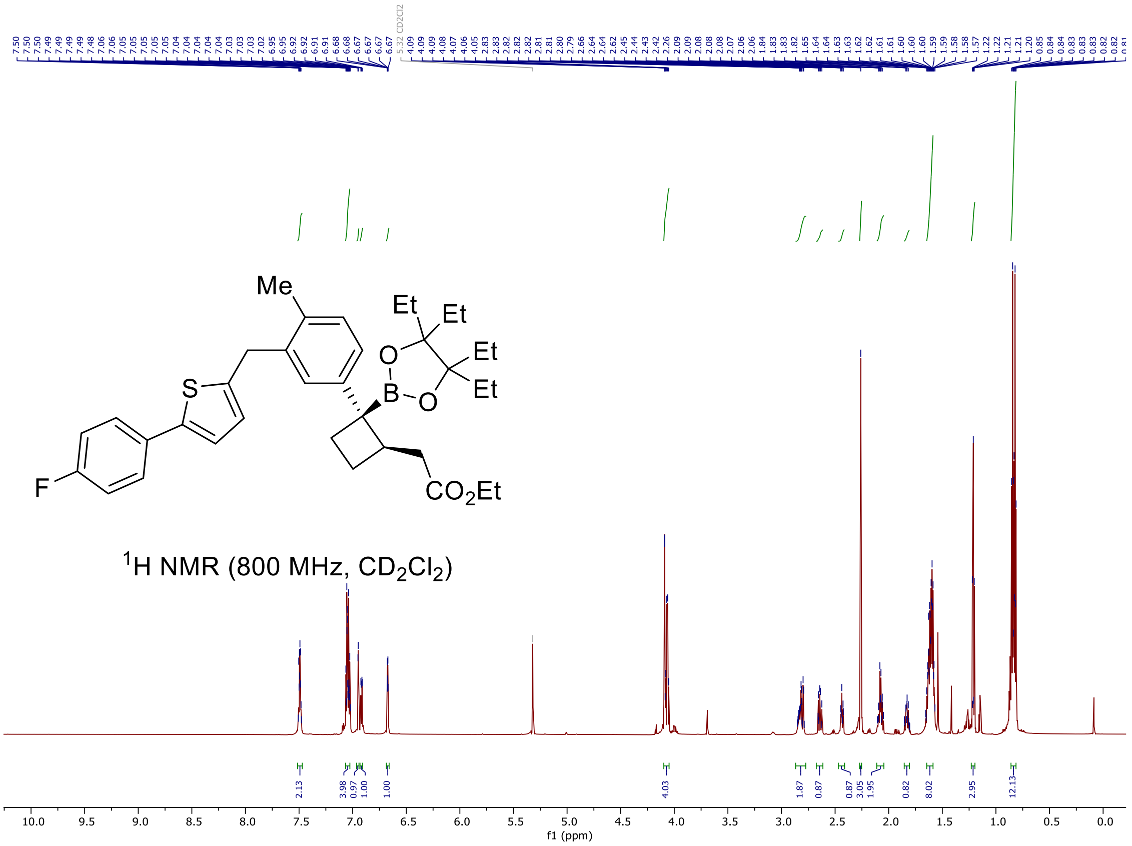

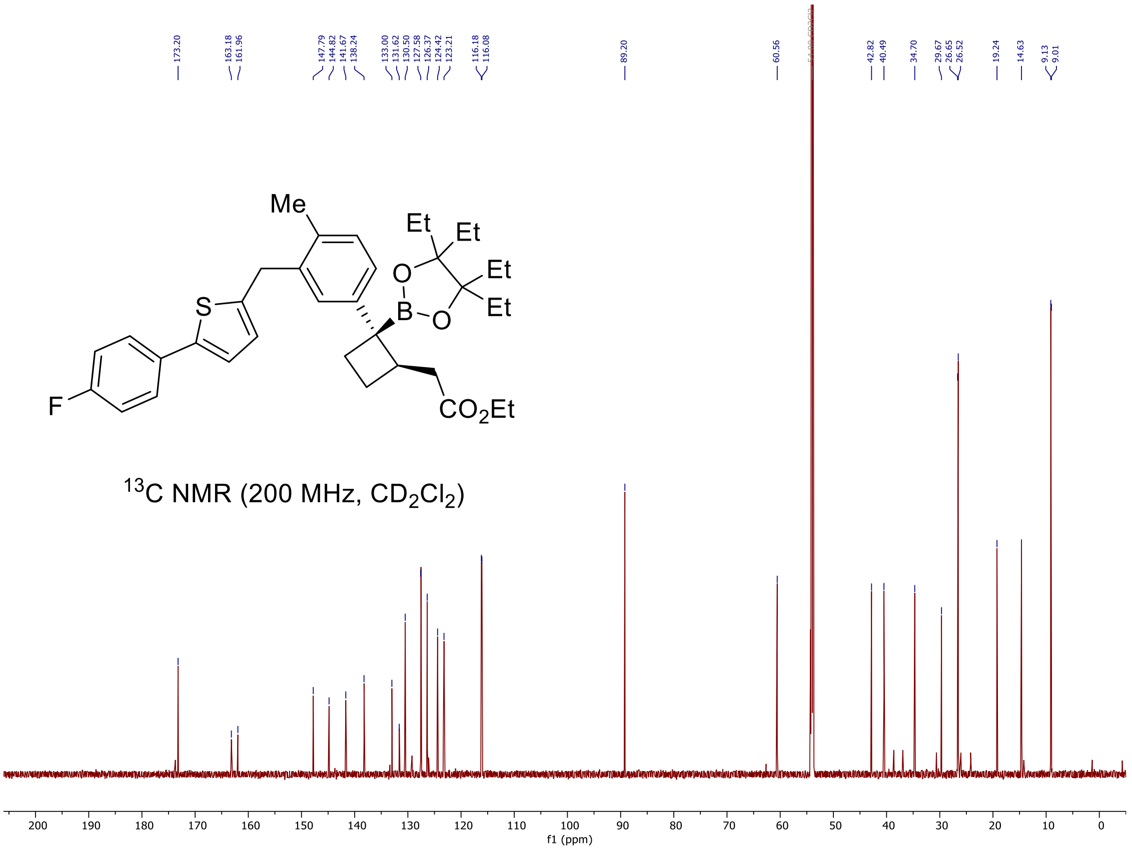

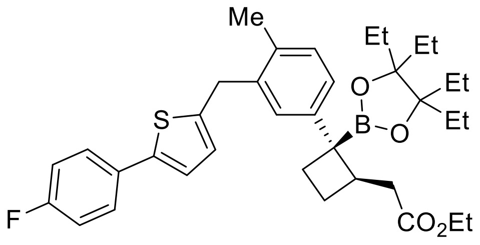

^{19}F NMR (400 MHz, CD_2Cl_2)

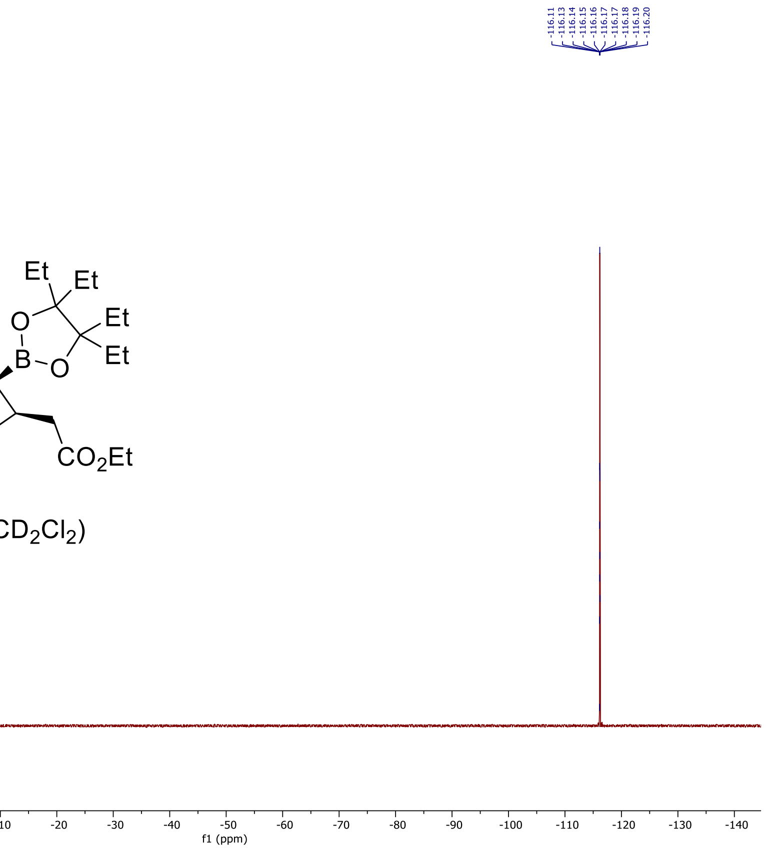

4,4,5,5-tetraethyl-2-((1*S*,2*S*)-1-(3-((5-(4-fluorophenyl)thiophen-2-yl)methyl)-4-methylphenyl)-2-(trifluoromethyl)cyclobutyl)-1,3,2-dioxaborolane (6h)

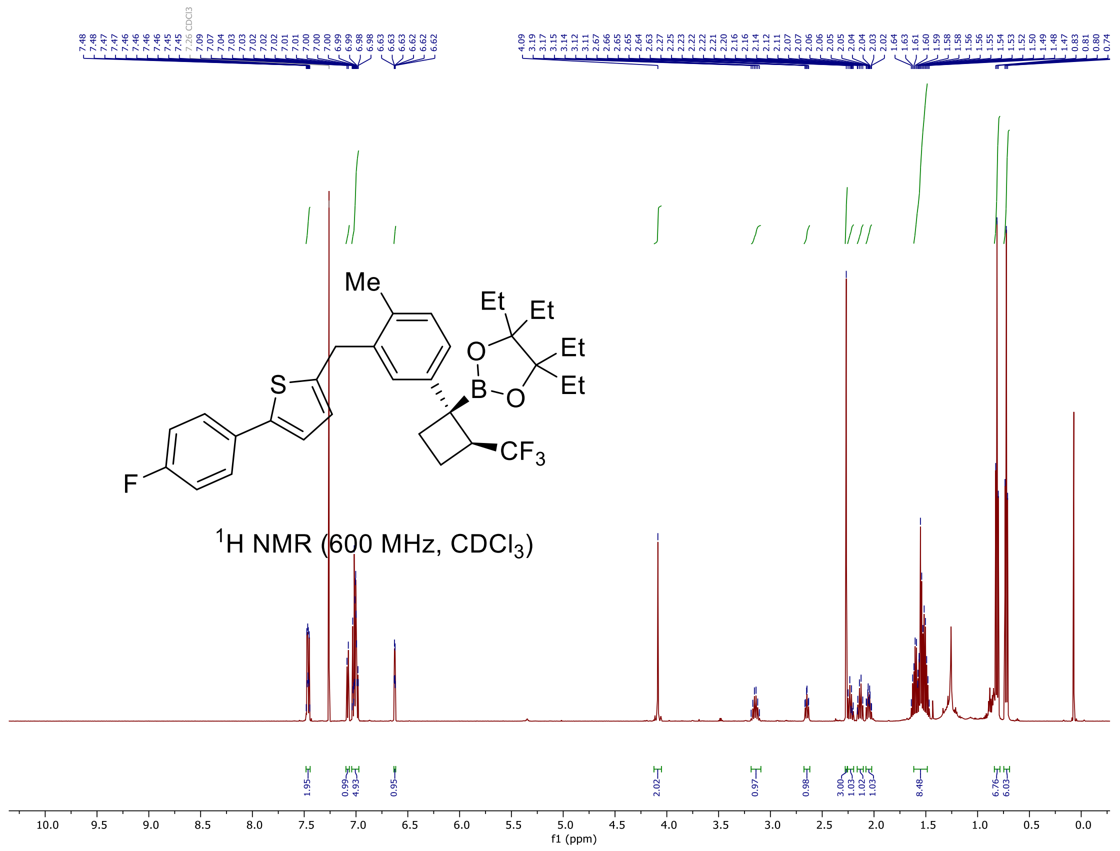

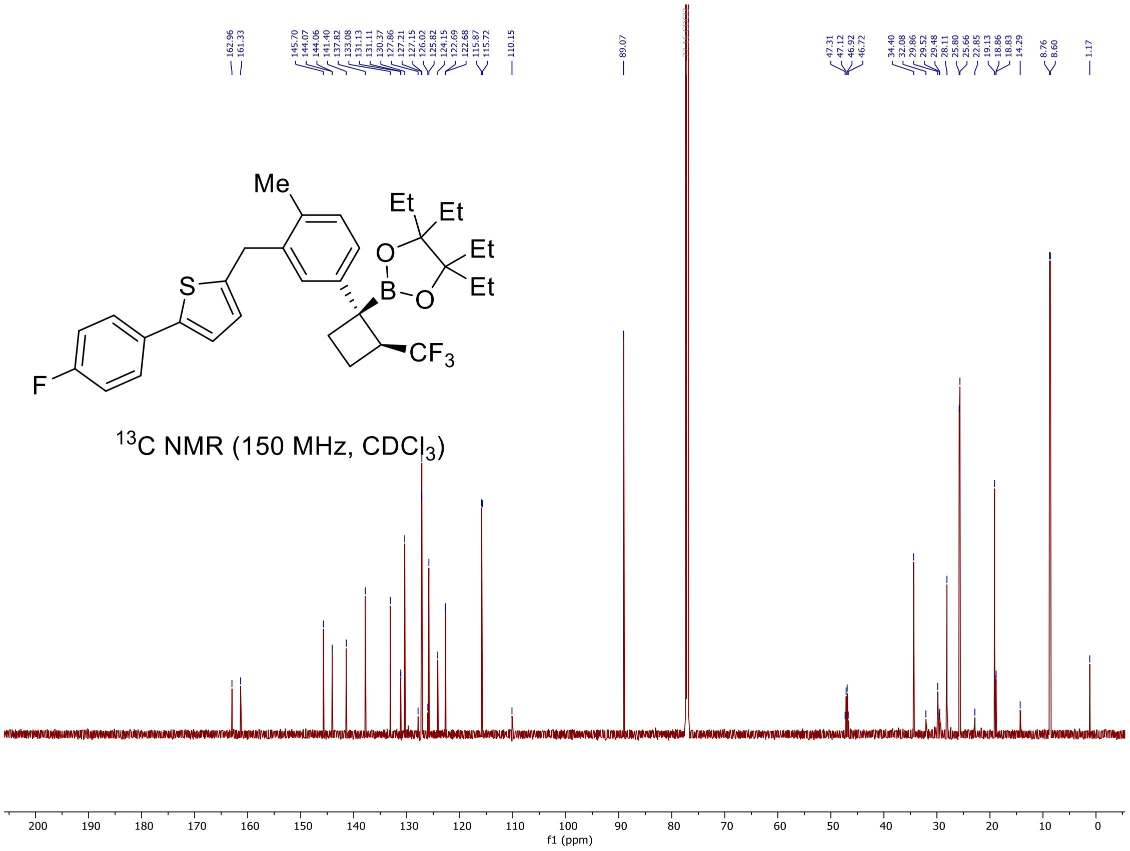

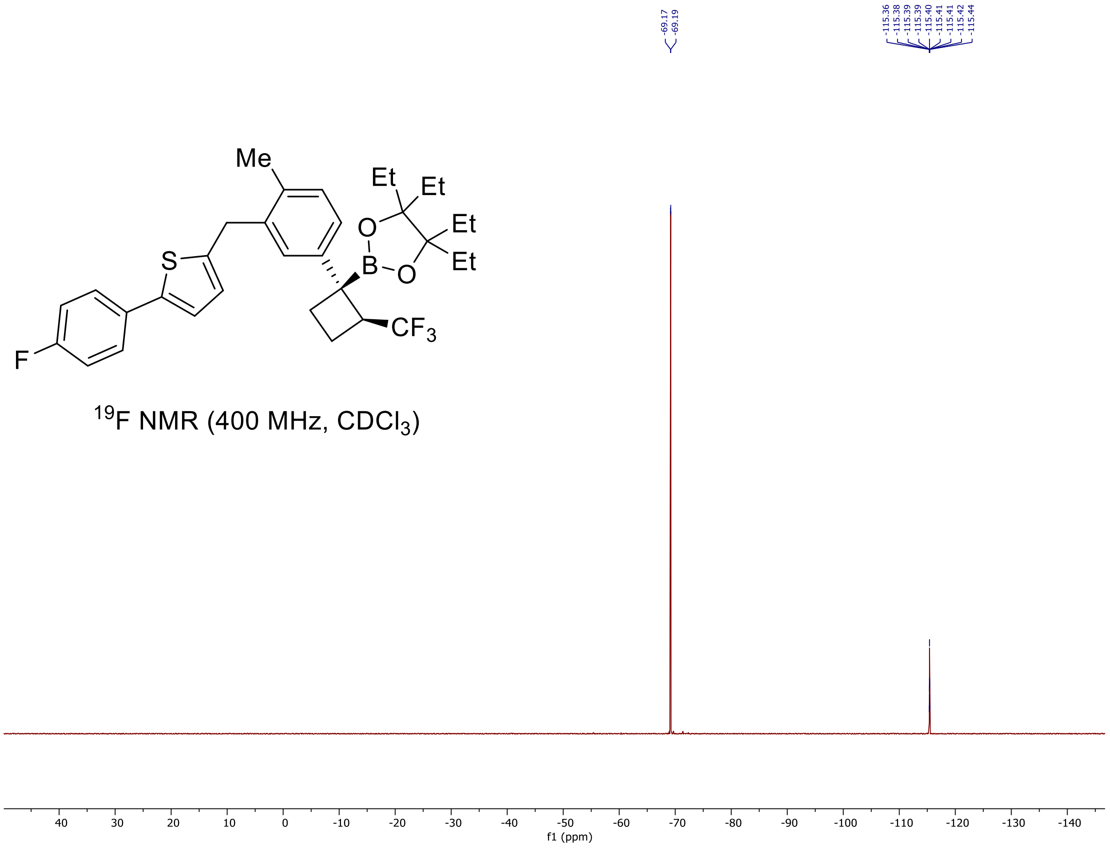

^{19}F NMR (400 MHz, $CDCl_3$)

Ethyl 2-((1*R*,2*S*)-2-butyl-2-(4,4,5,5-tetraethyl-1,3,2-dioxaborolan-2-yl)cyclopentyl)acetate (9a)

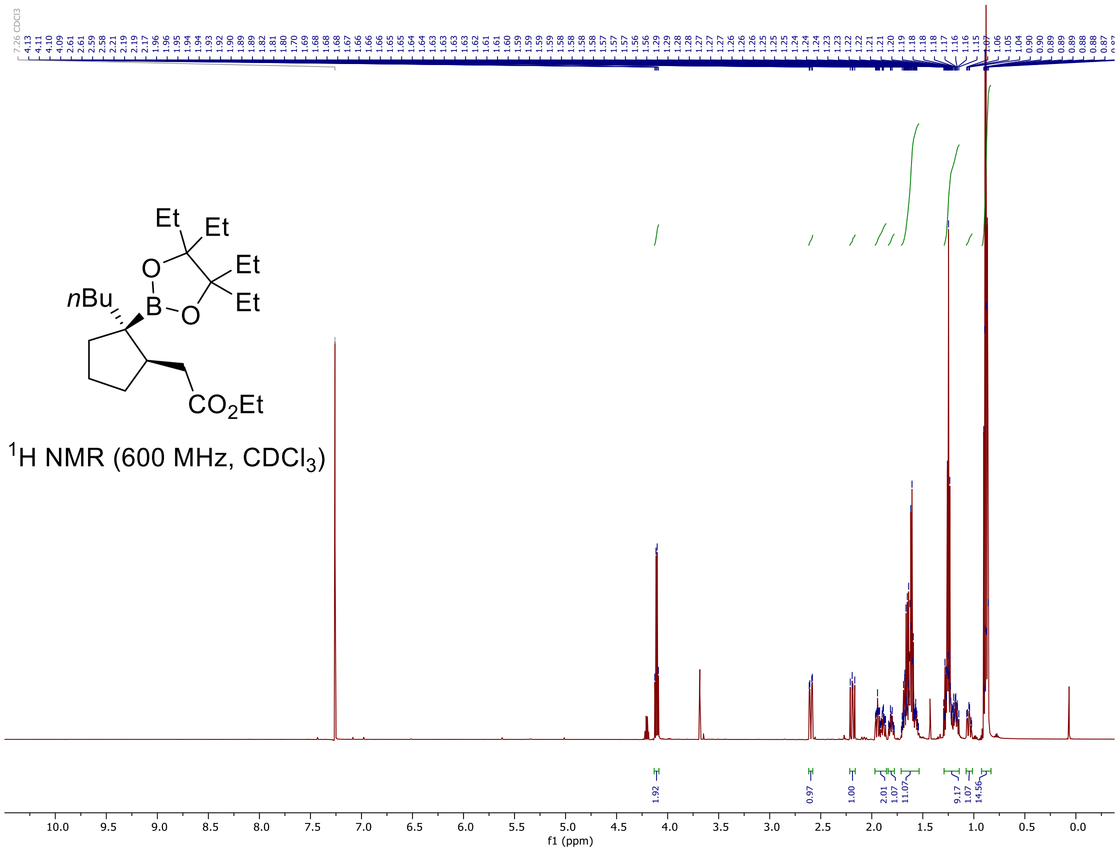

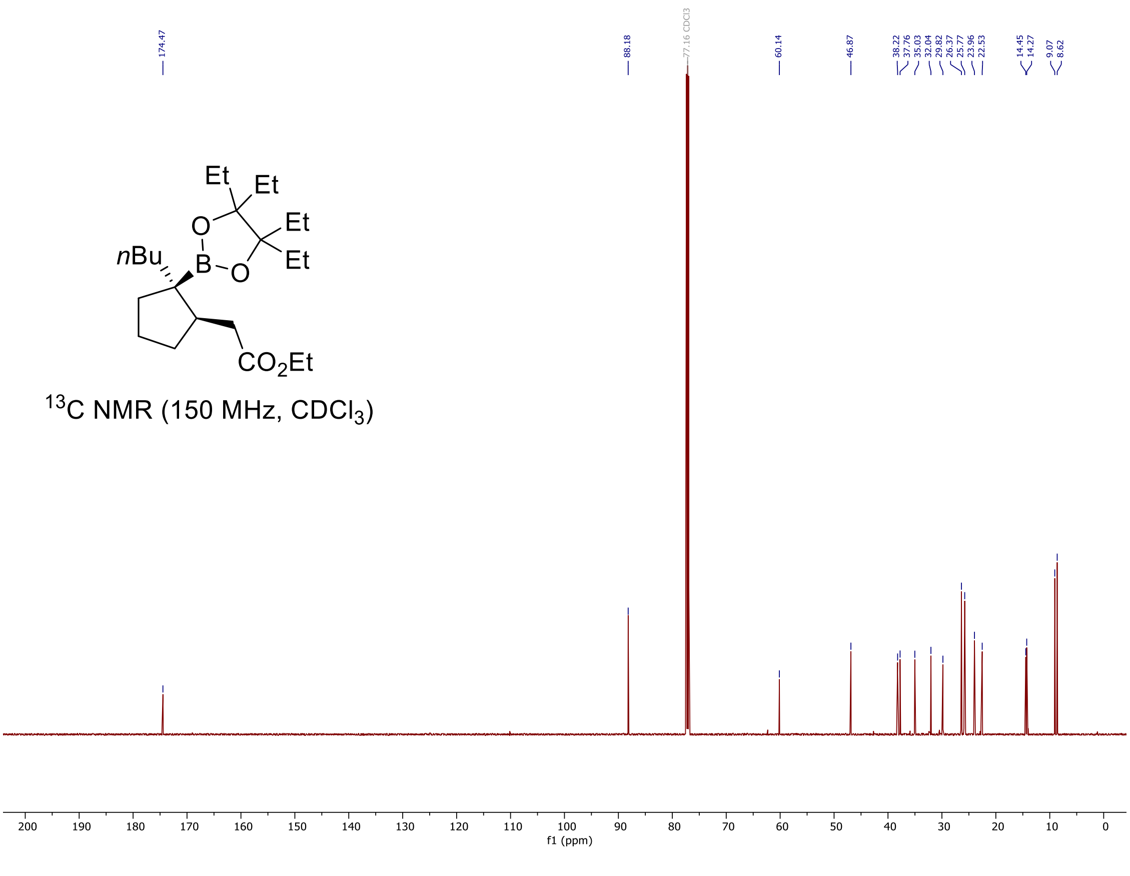

tert-Butyl 2-((1*R*,2*S*)-2-butyl-2-(4,4,5,5-tetraethyl-1,3,2-dioxaborolan-2-yl)cyclopentyl)acetate (<u>9a'</u>)

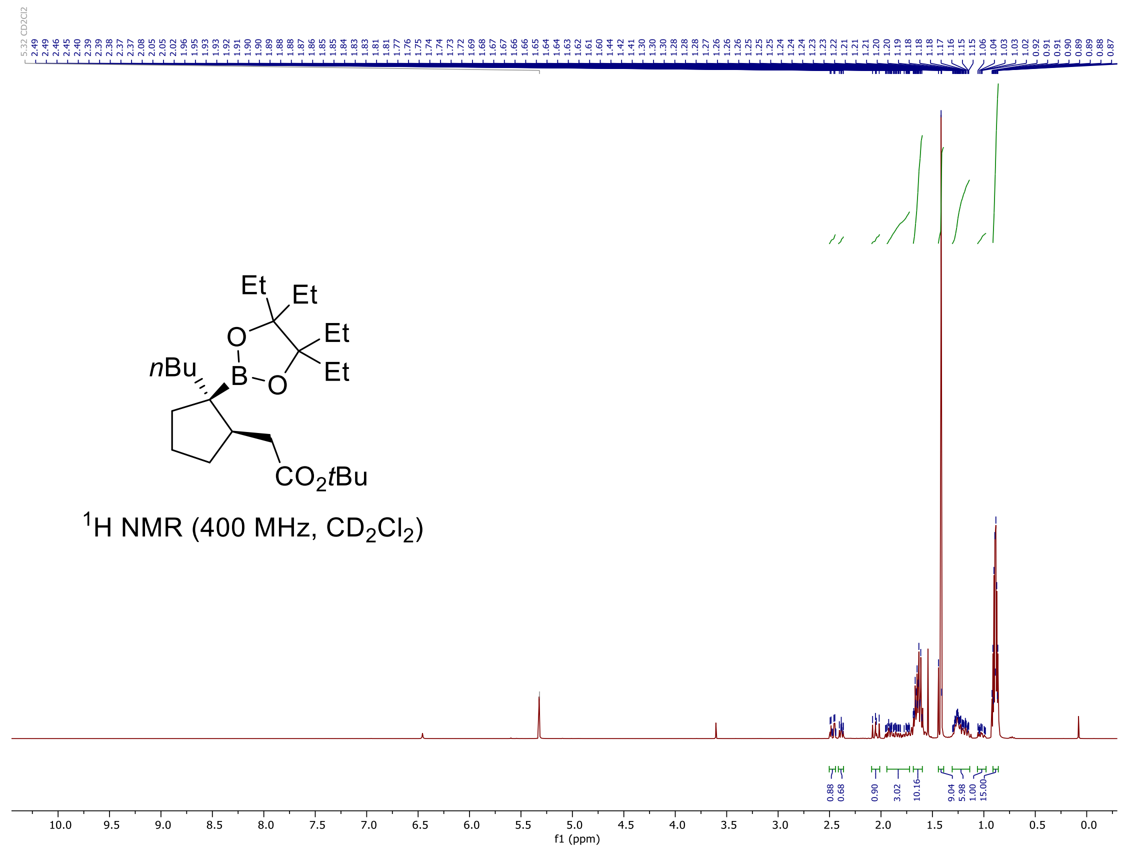

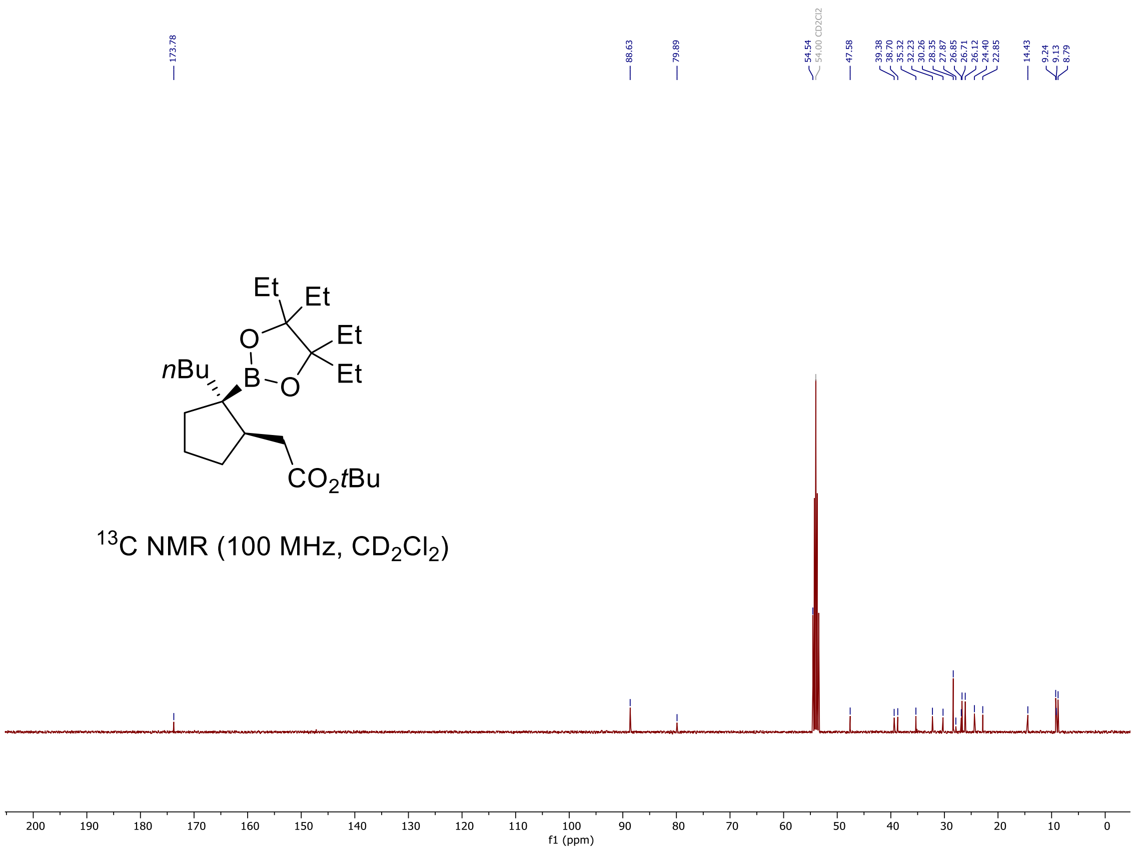

Ethyl 2-((1*R*,2*S*)-2-hexyl-2-(4,4,5,5-tetraethyl-1,3,2-dioxaborolan-2-yl)cyclopentyl)acetate (9b)

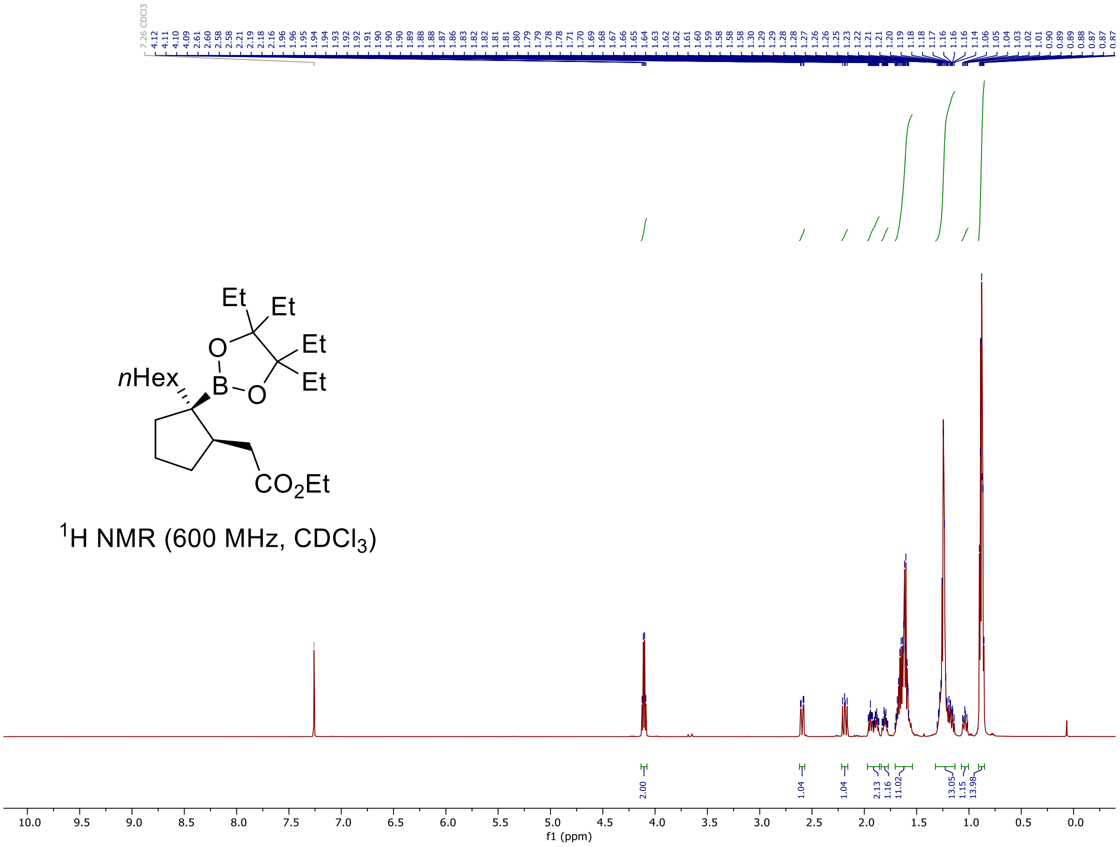

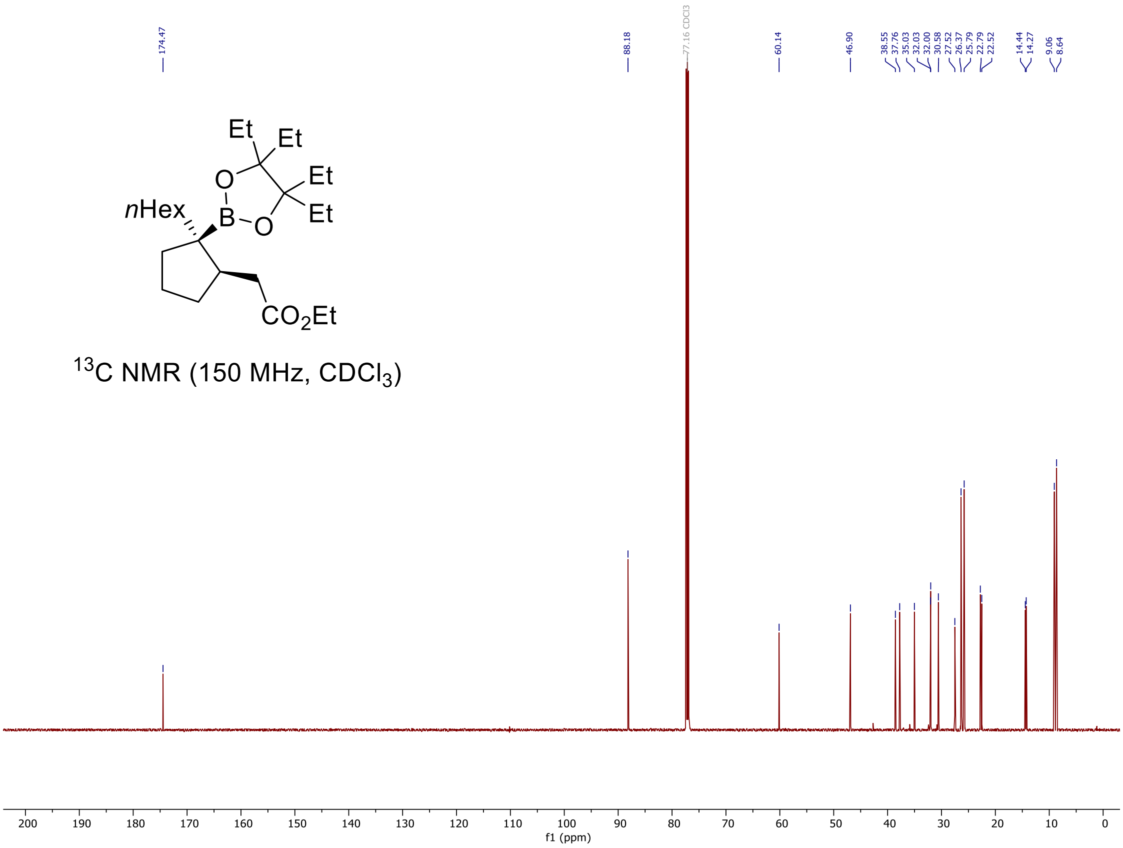

2-((1*R*,2*S*)-2-hexyl-2-(4,4,5,5-tetraethyl-1,3,2-dioxaborolan-2-yl)cyclopentyl)acetic acid (<u>9b'</u>)

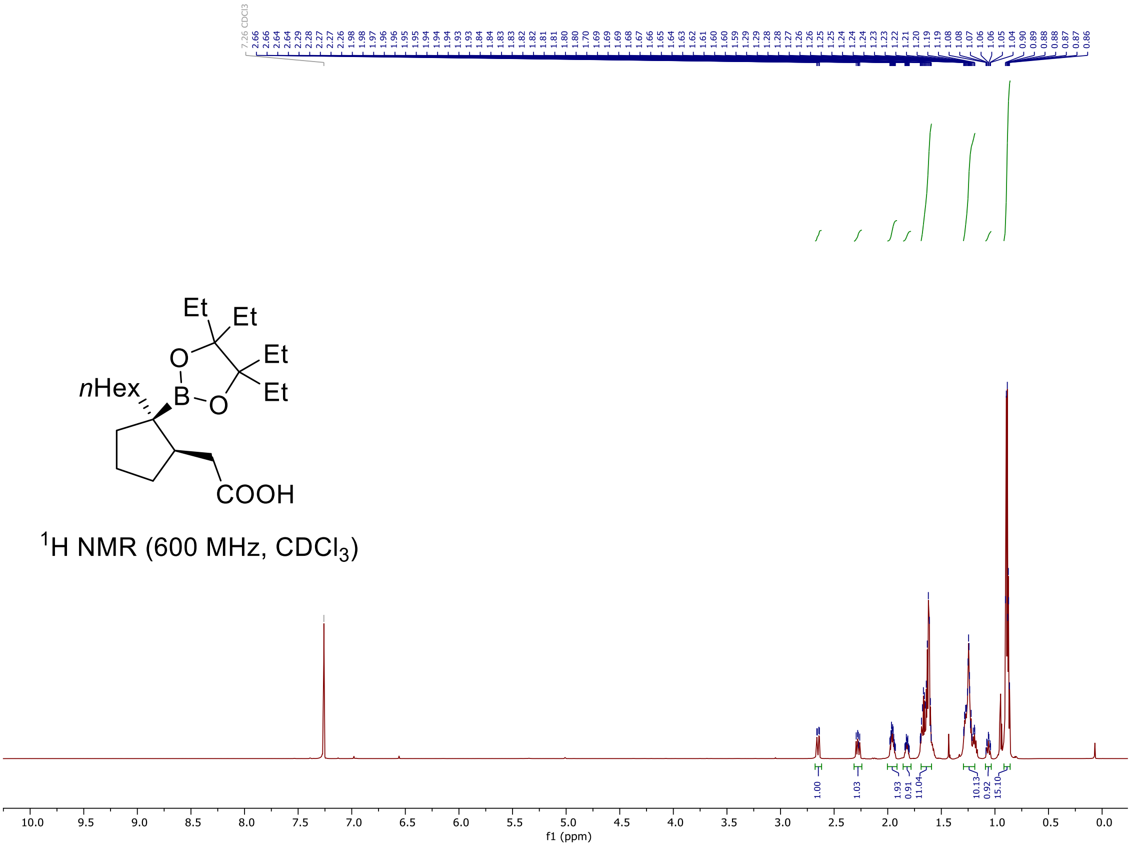

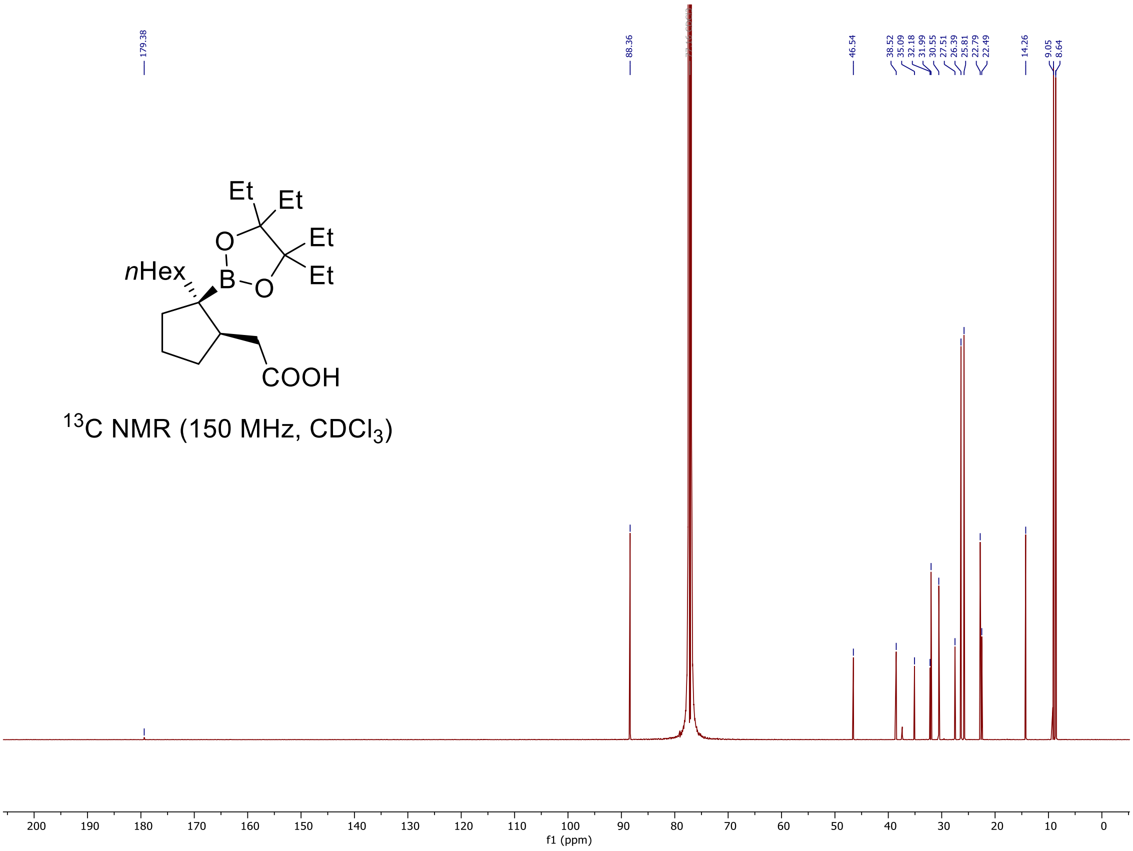

tert-Butyl 2-((1_R_,2S)-2-hexyl-2-(4,4,5,5-tetraethyl-1,3,2-dioxaborolan-2-yl)cyclopentyl)acetate (9b’’)

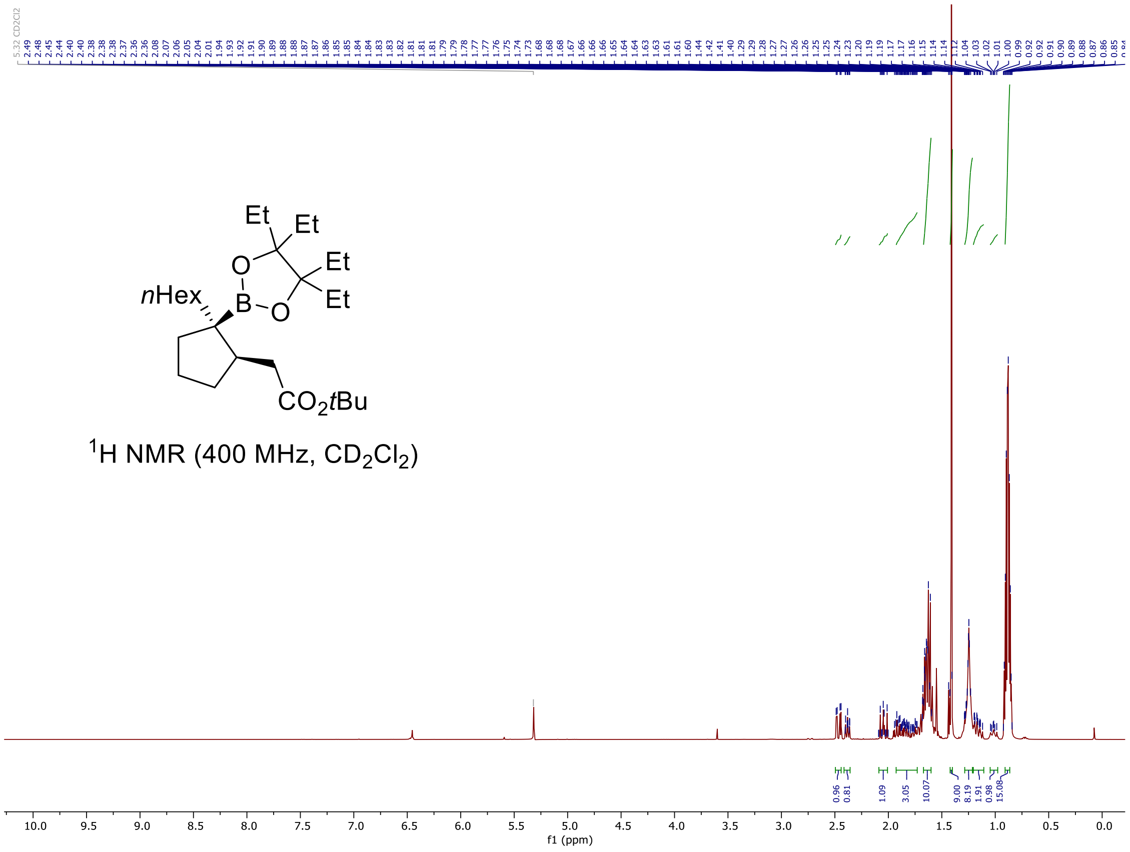

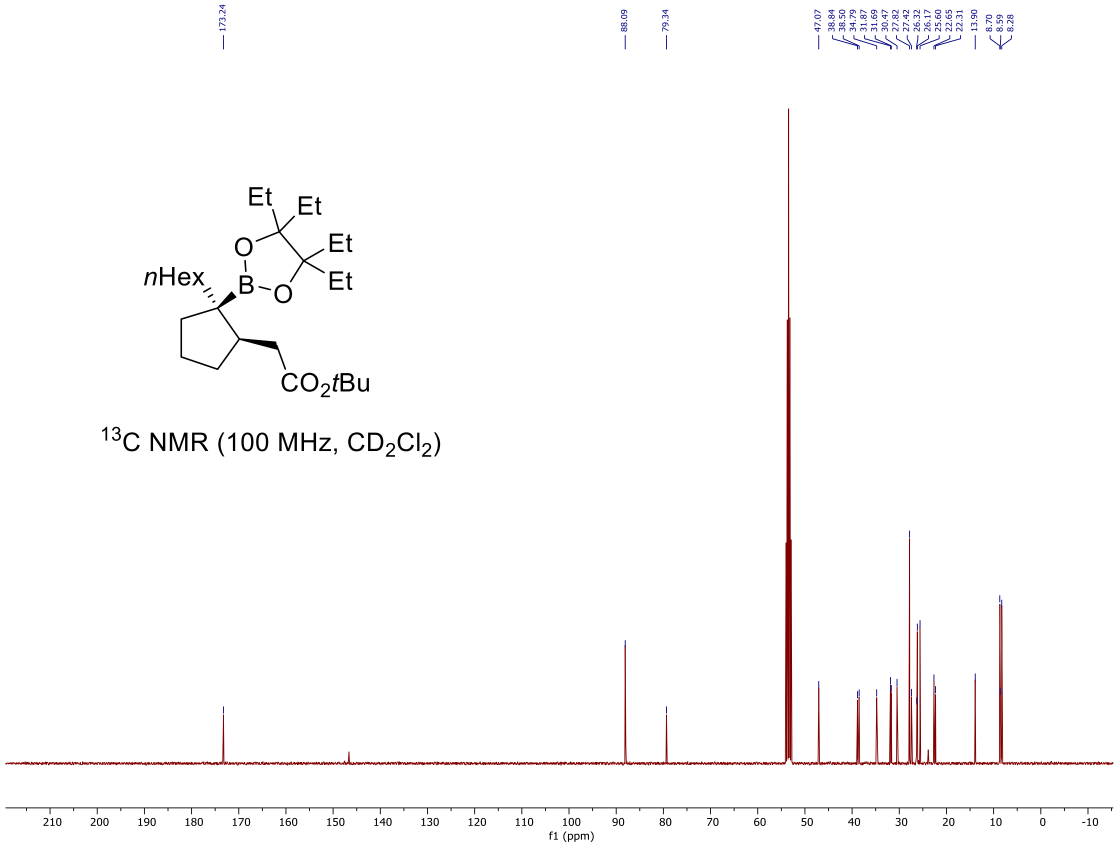

Ethyl 2-((1*R*,2*S*)-2-(4,4,5,5-tetraethyl-1,3,2-dioxaborolan-2-yl)-2-((trimethylsilyl)methyl)cyclopentyl)acetate (9c)

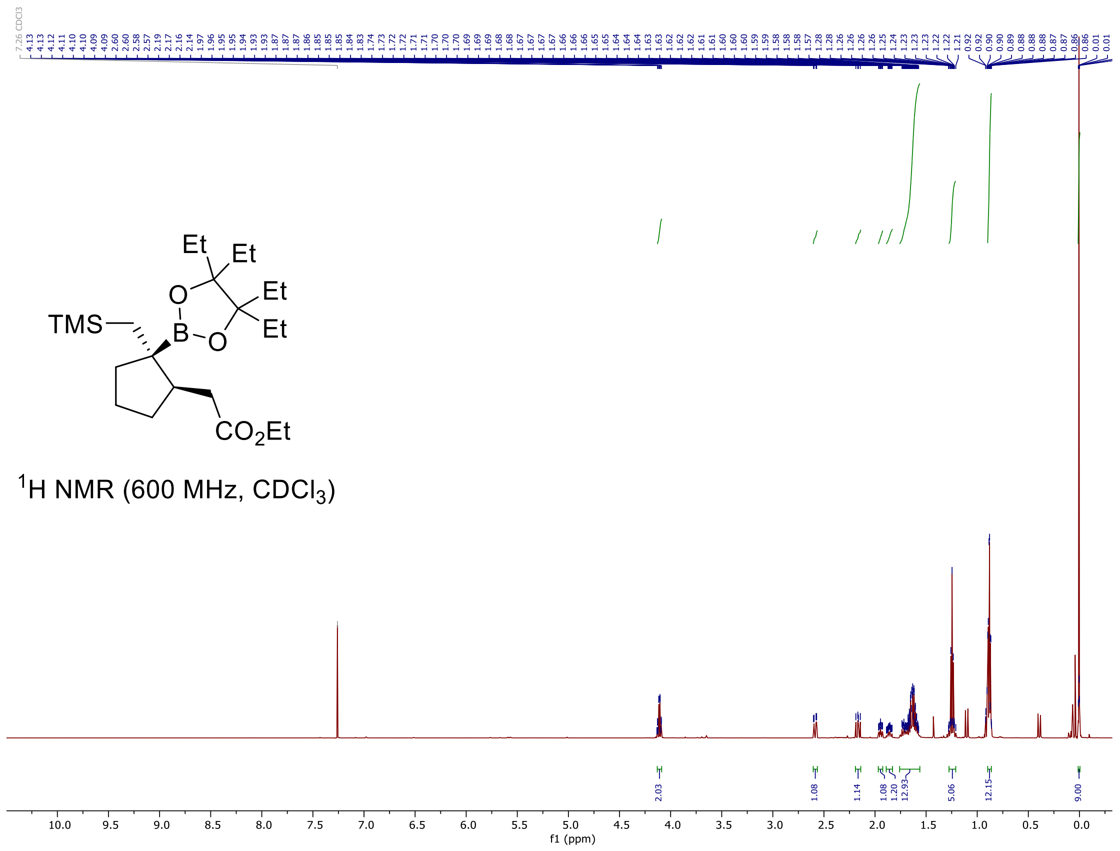

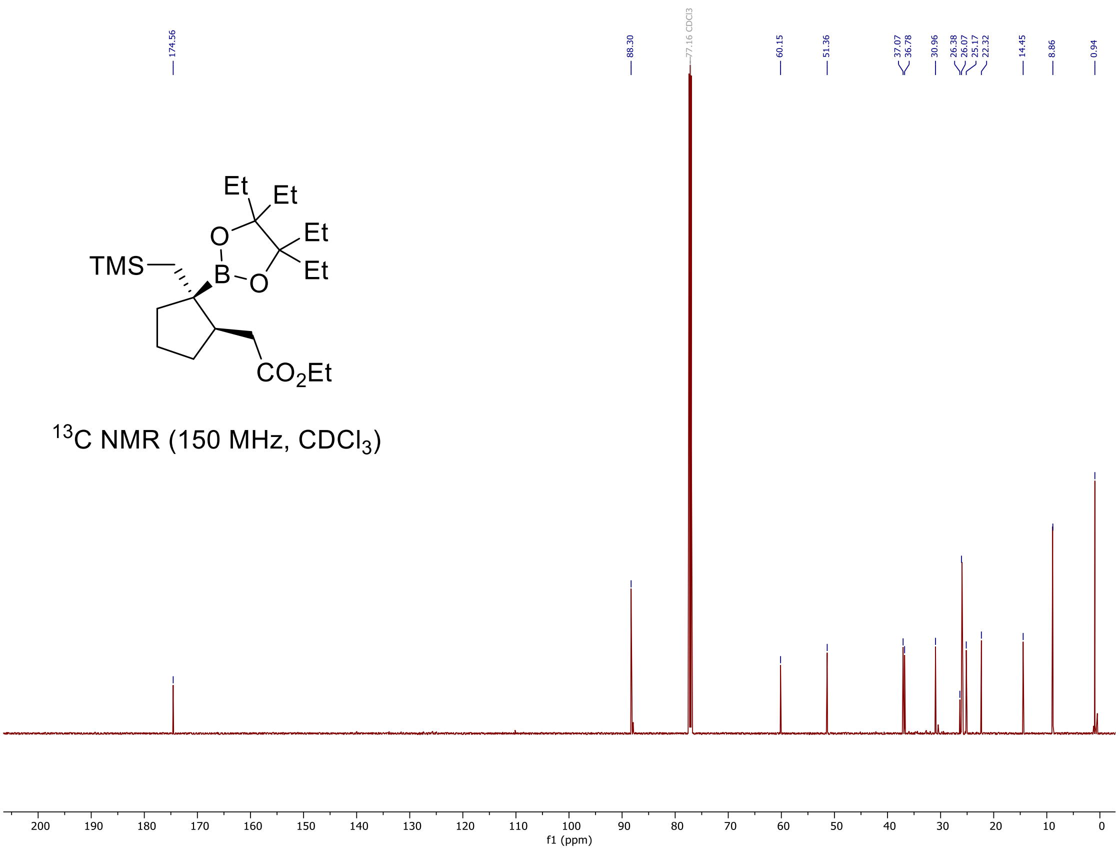

Ethyl 2-((1*R*,2*S*)-2-(4,4,5,5-tetraethyl-1,3,2-dioxaborolan-2-yl)-2-(3,4,5-trifluorophenyl)cyclopentyl)acetate (9d)

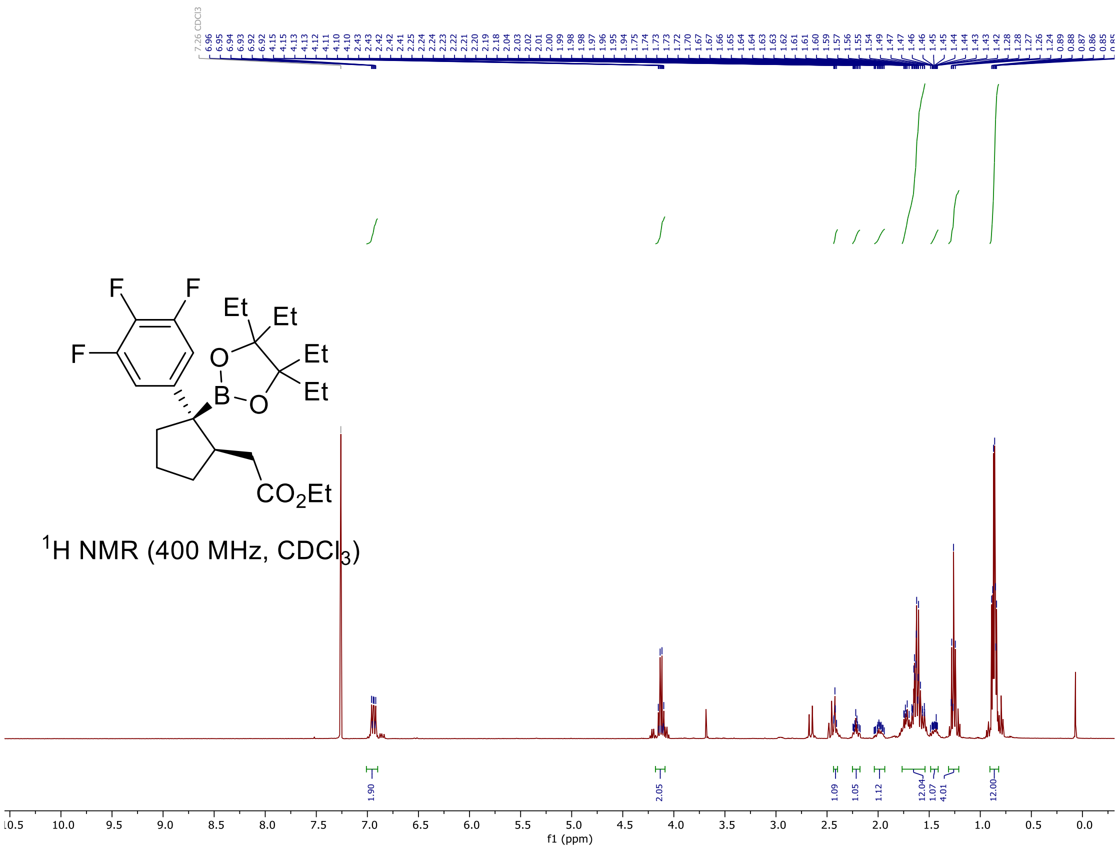

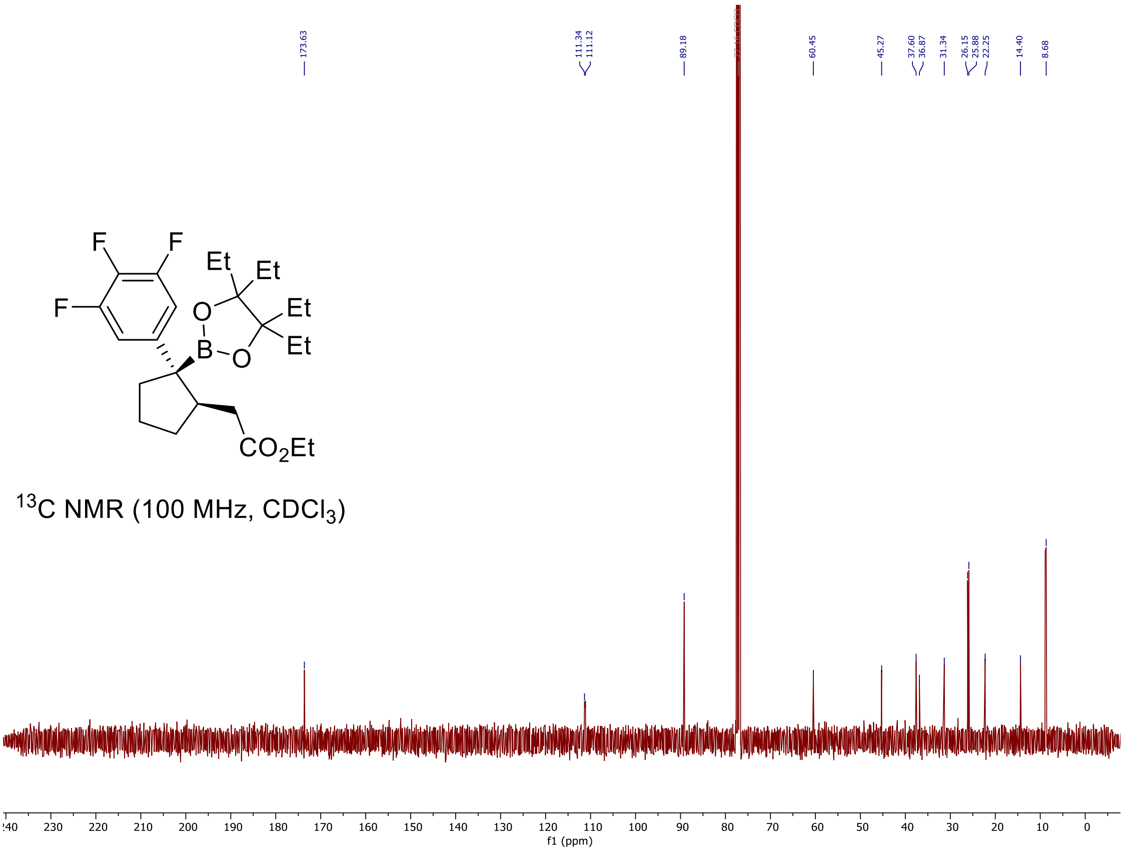

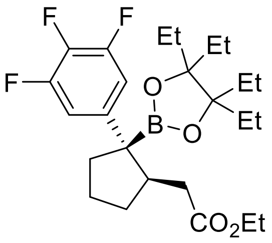

^{19}F NMR (400 MHz, $CDCl_3$)

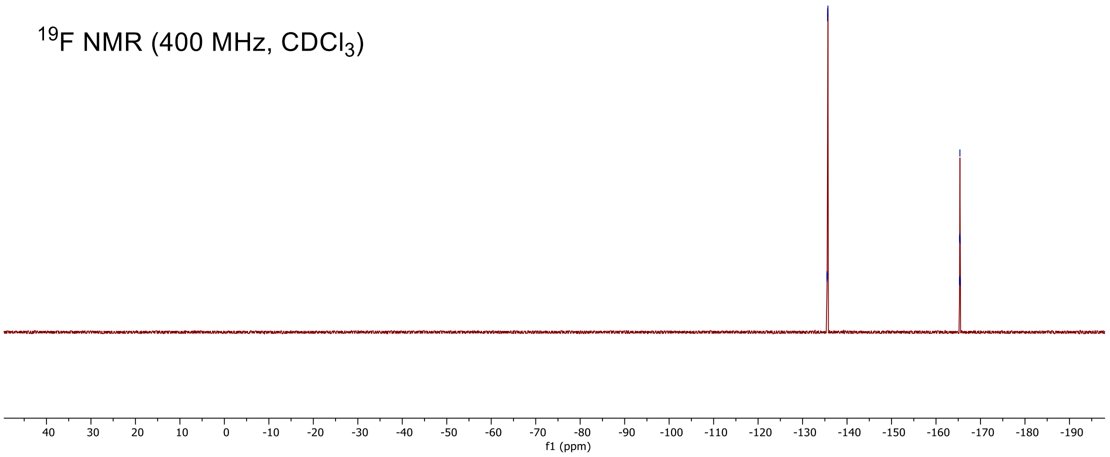

Trimethyl(((1*S*,2*S*)-1-(4,4,5,5-tetraethyl-1,3,2-dioxaborolan-2-yl)-2-(trifluoromethyl)cyclopentyl)methyl)silane (9e)

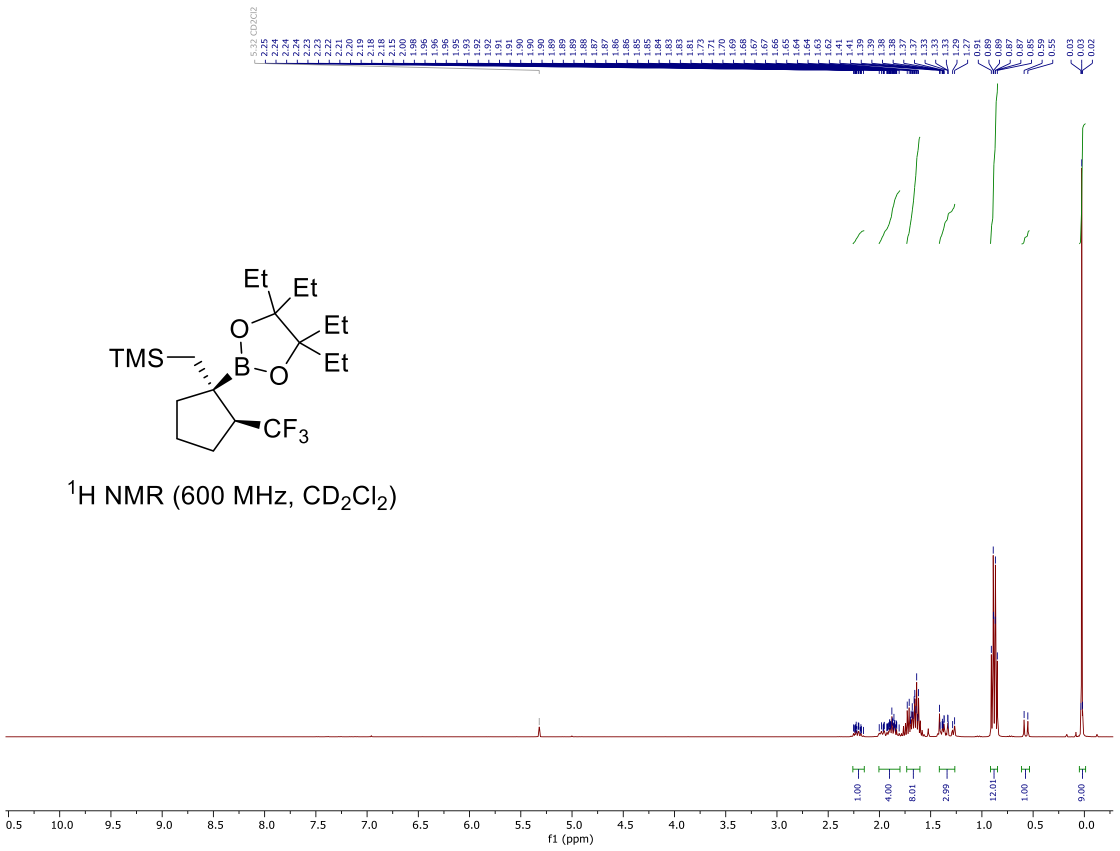

^{1}H NMR (600 MHz, CD_2Cl_2)

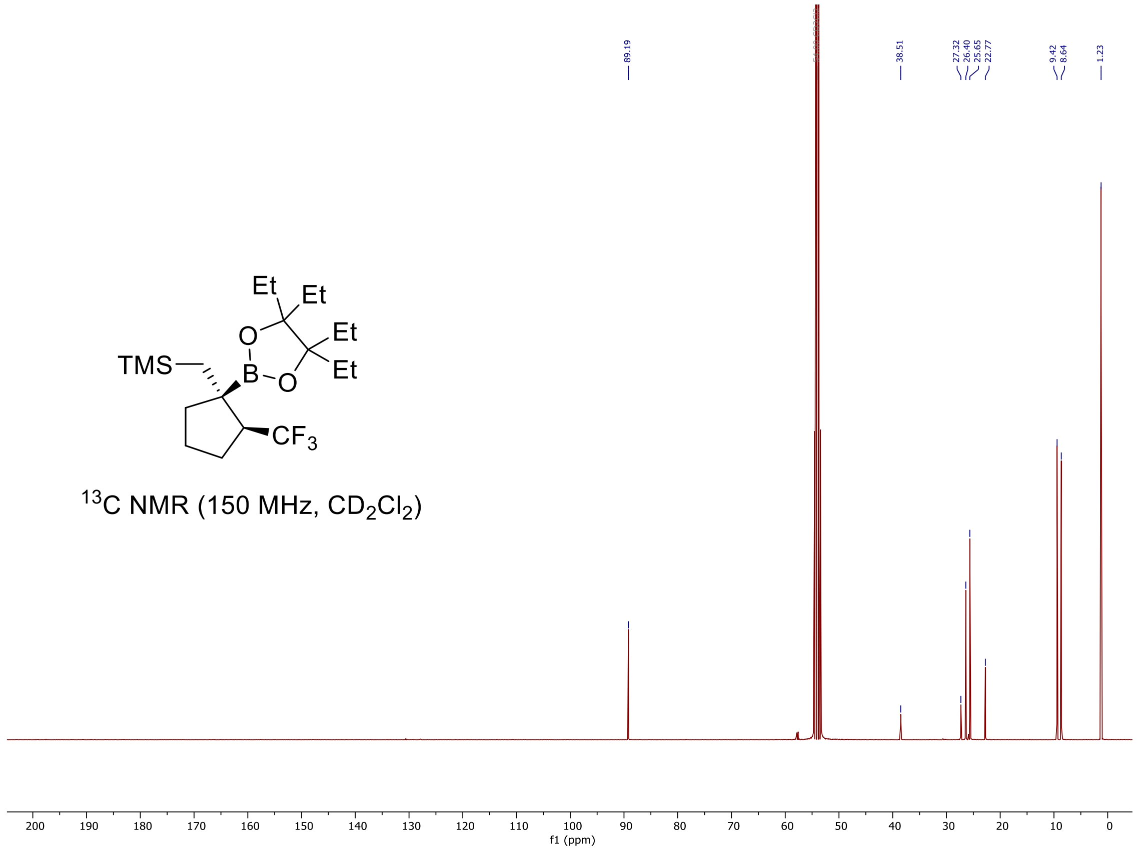

^{13}C NMR (150 MHz, CD_2Cl_2)

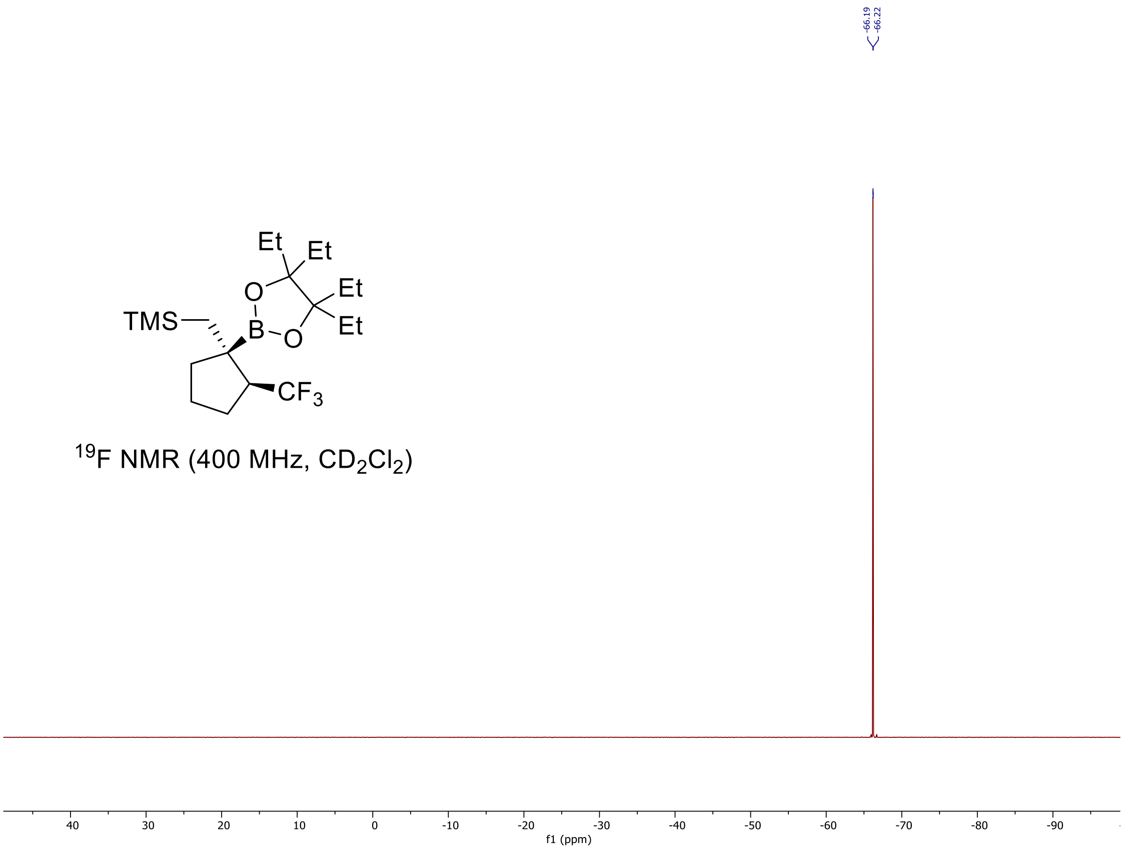

^{19}F NMR (400 MHz, CD_2Cl_2)

Ethyl 2-((8*S*,9*S*,13*S*,14*S*,16*R*,17*R*)-17-hexyl-3-methoxy-13-methyl-17-(4,4,5,5-tetraethyl-1,3,2-dioxaborolan-2-yl)-7,8,9,11,12,13,14,15,16,17-decahydro-6H-cyclopenta[a]phenanthren-16-yl)acetate (9f)

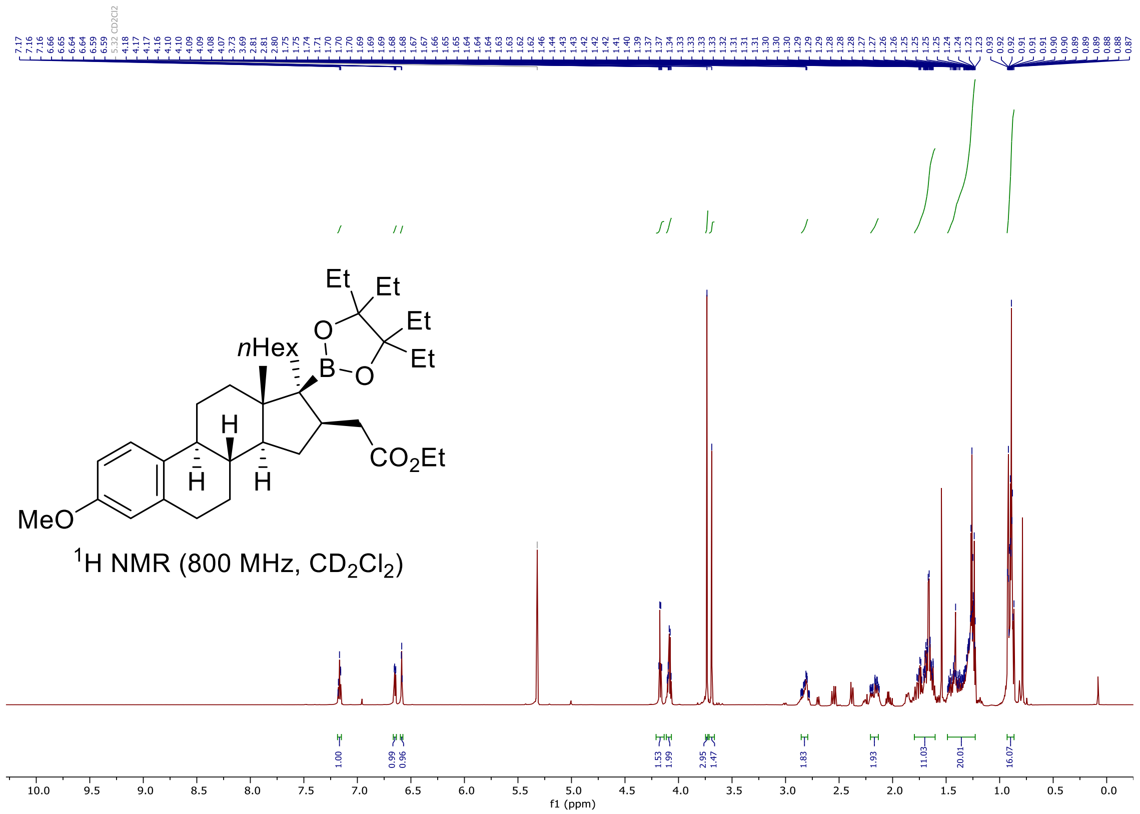

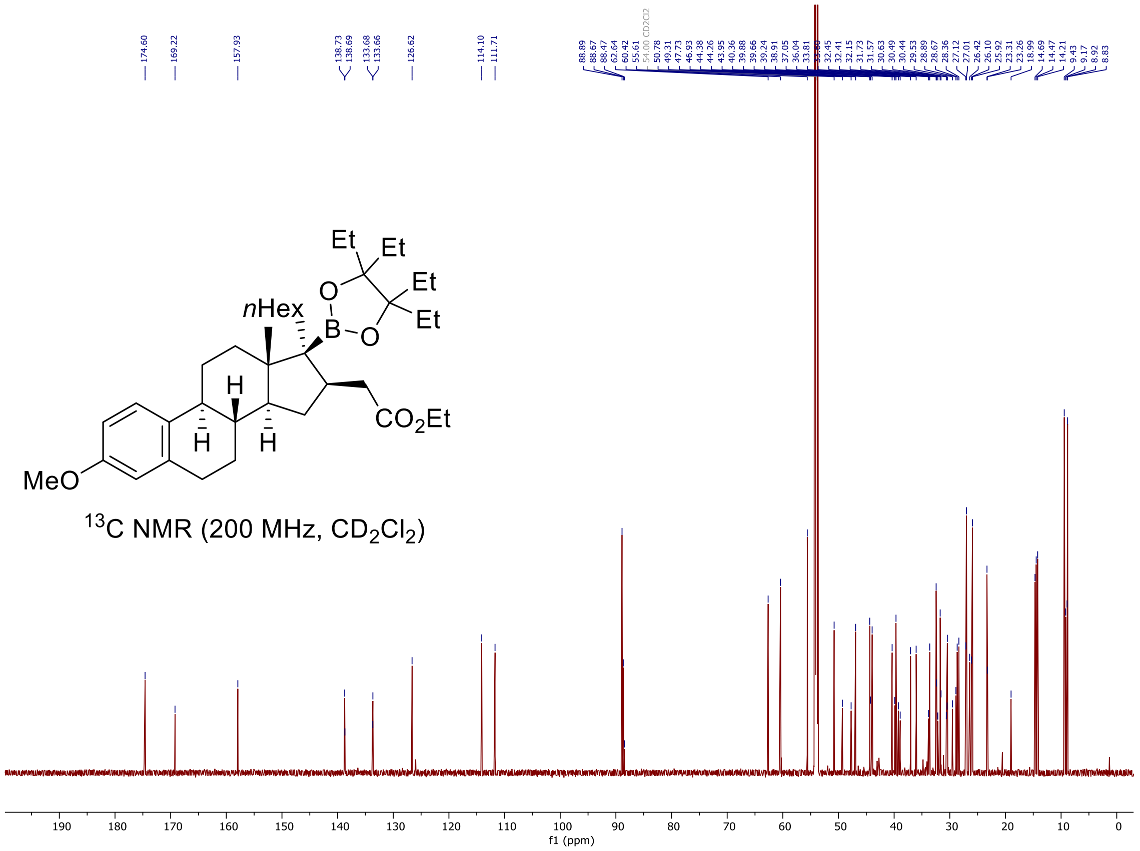

Ethyl 2-((8*S*,9*S*,13*S*,14*S*,16*R*,17*R*)-3-methoxy-13-methyl-17-(4,4,5,5-tetraethyl-1,3,2-dioxaborolan-2-yl)-17-((trimethylsilyl)methyl)-7,8,9,11,12,13,14,15,16,17-decahydro-6H-cyclopenta[a]phenanthren-16-yl)acetate (9g)

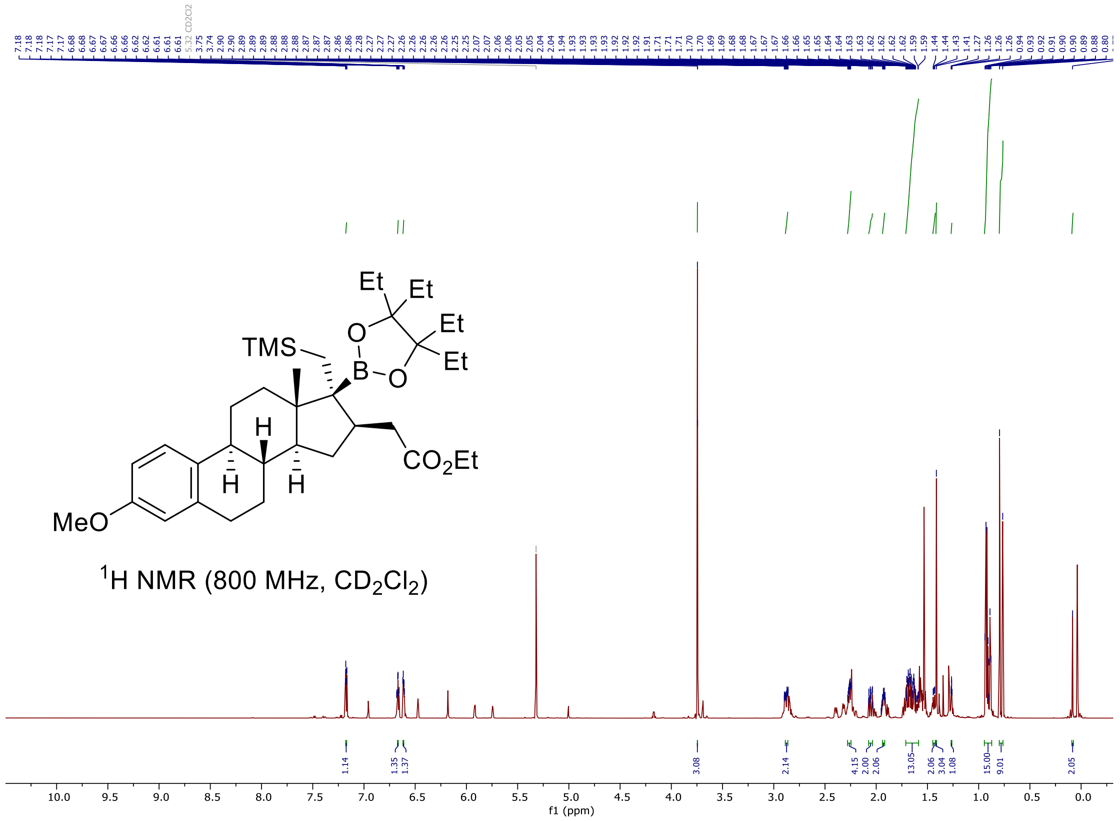

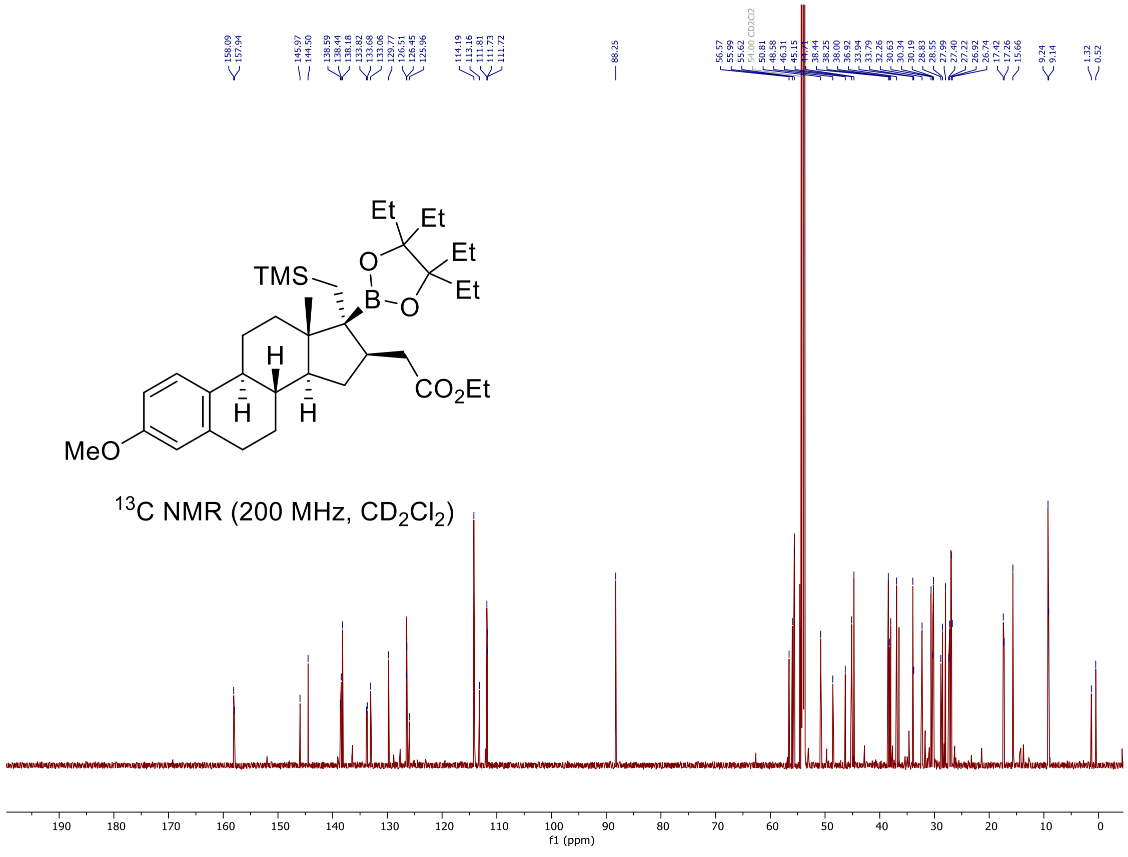

***tert*-Butyl (2*S*,3*R*)-3-(2-ethoxy-2-oxoethyl)-2-phenyl-2-(4,4,5,5-tetraethyl-1,3,2-dioxaborolan-2-yl)pyrrolidine-1-carboxylate (<u>12a</u>)**

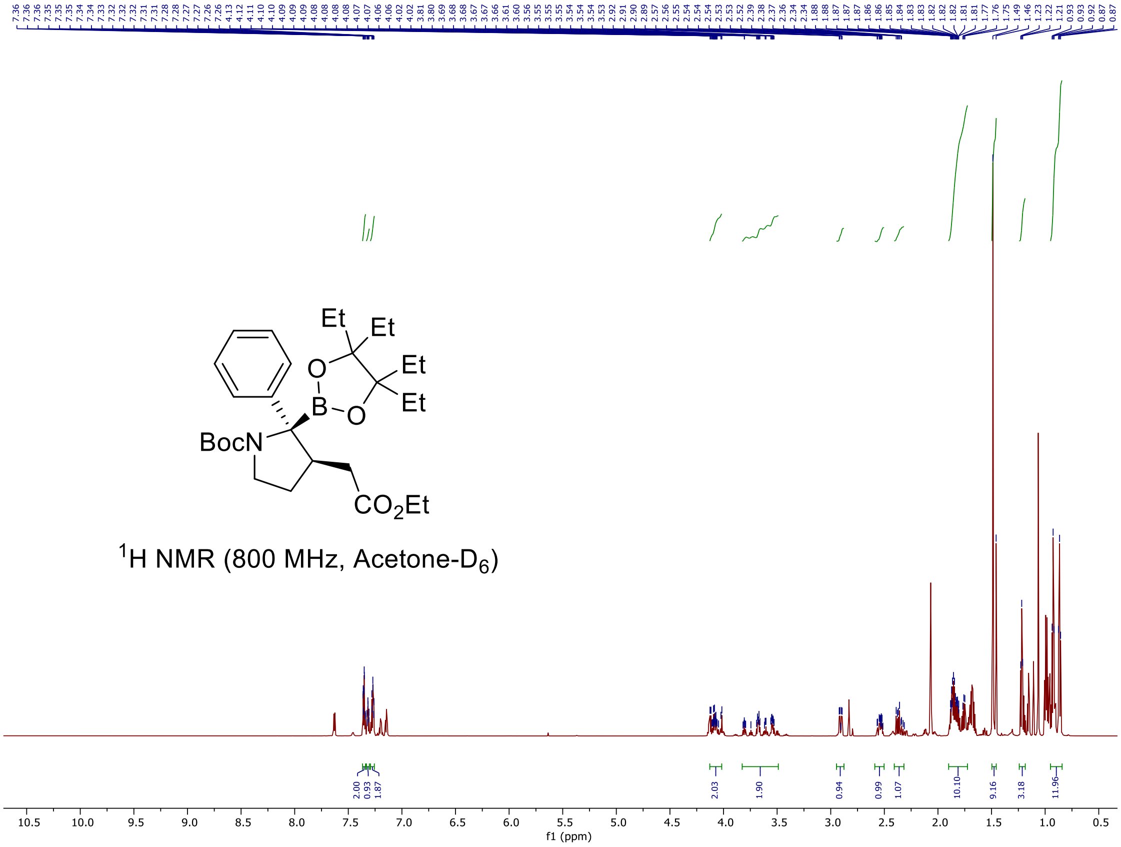

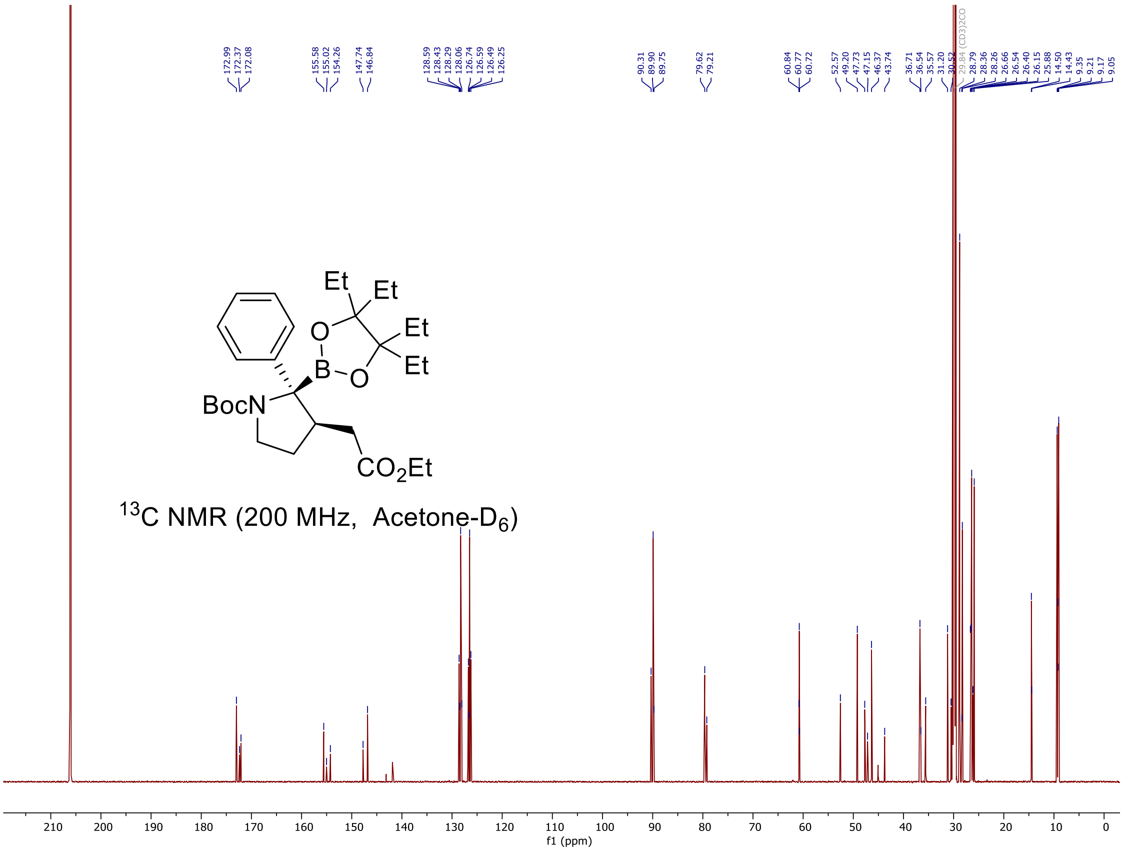

2-((2*R*,3*R*)-2-Isobutyl-2-(4,4,5,5-tetraethyl-1,3,2-dioxaborolan-2-yl)tetrahydrofuran-3-yl)acetamide (12b)

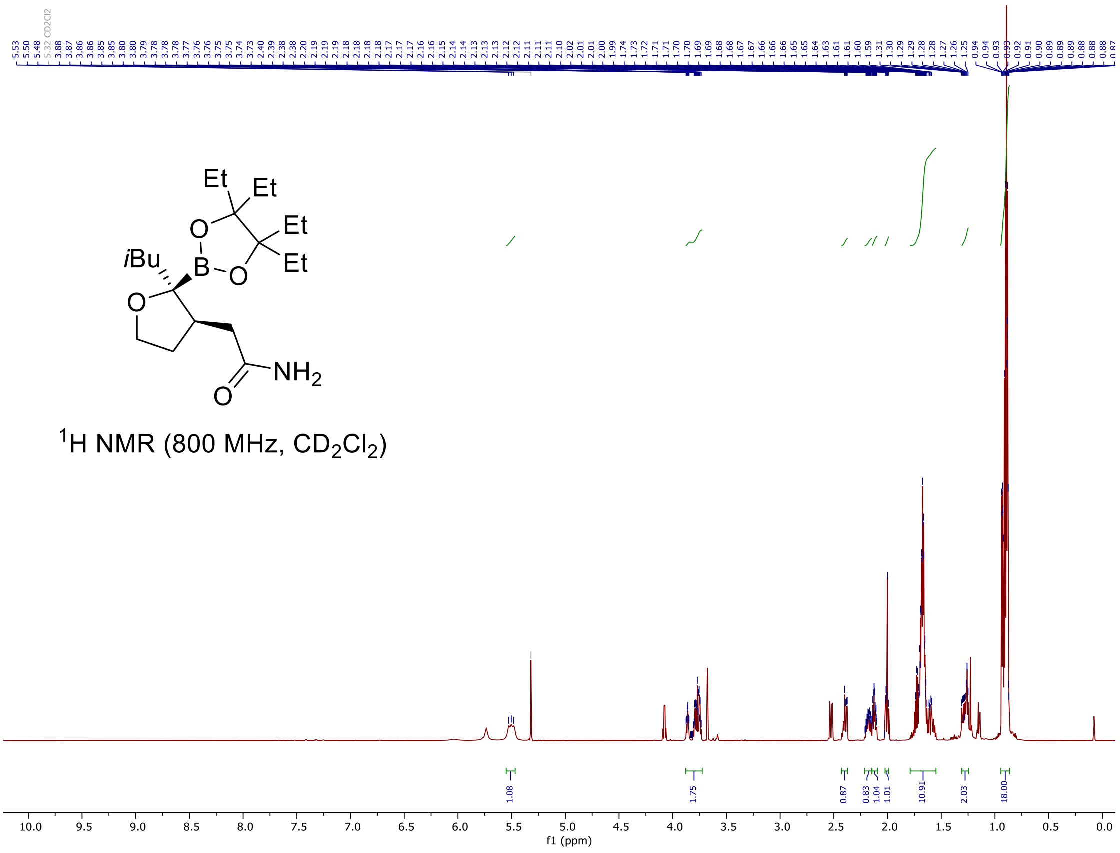

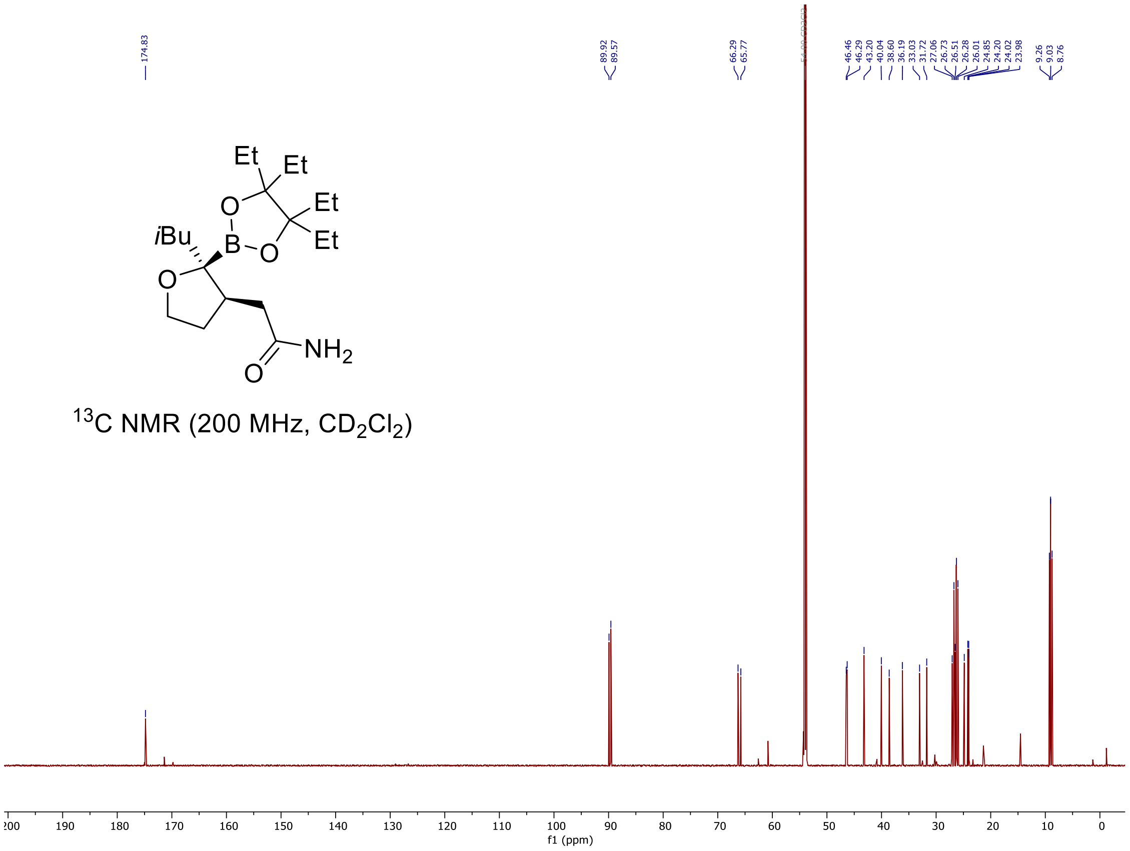

Ethyl 2-((2*R*,3*R*)-2-(cyclohexylmethyl)-2-(4,4,5,5-tetraethyl-1,3,2-dioxaborolan-2-yl)tetrahydrofuran-3-yl)acetate (12c)

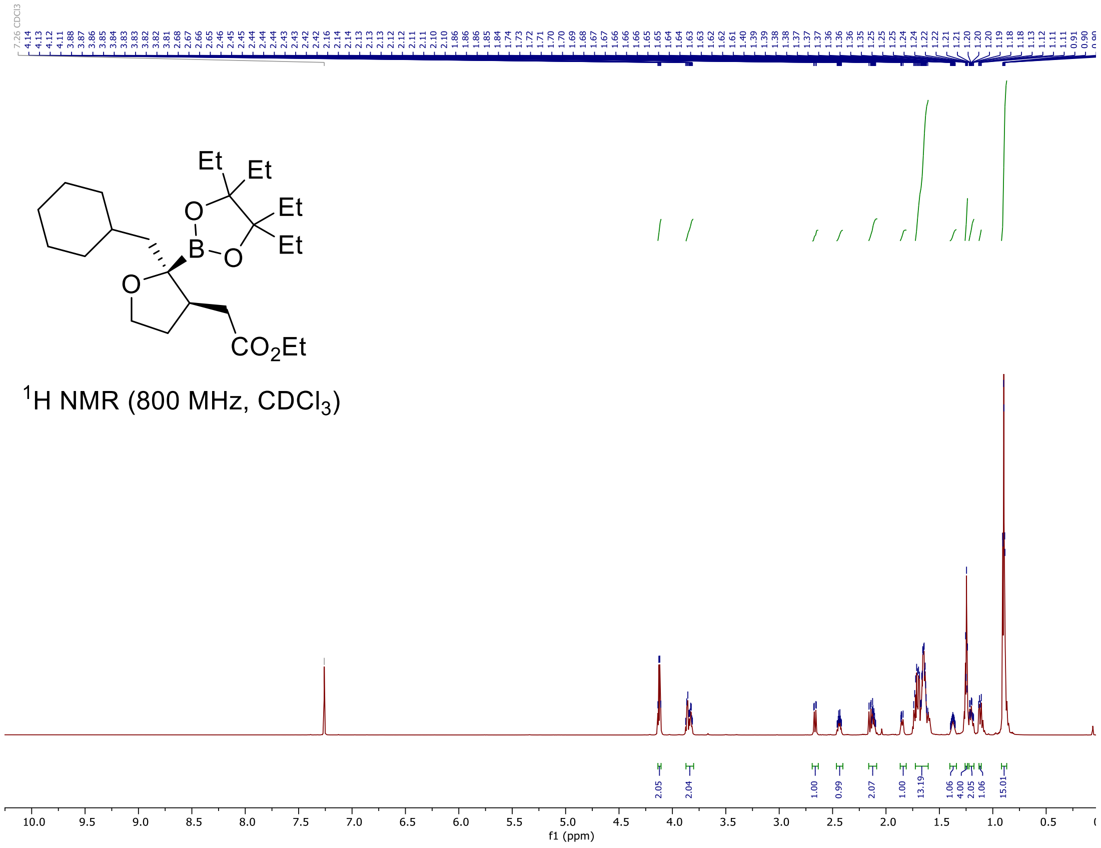

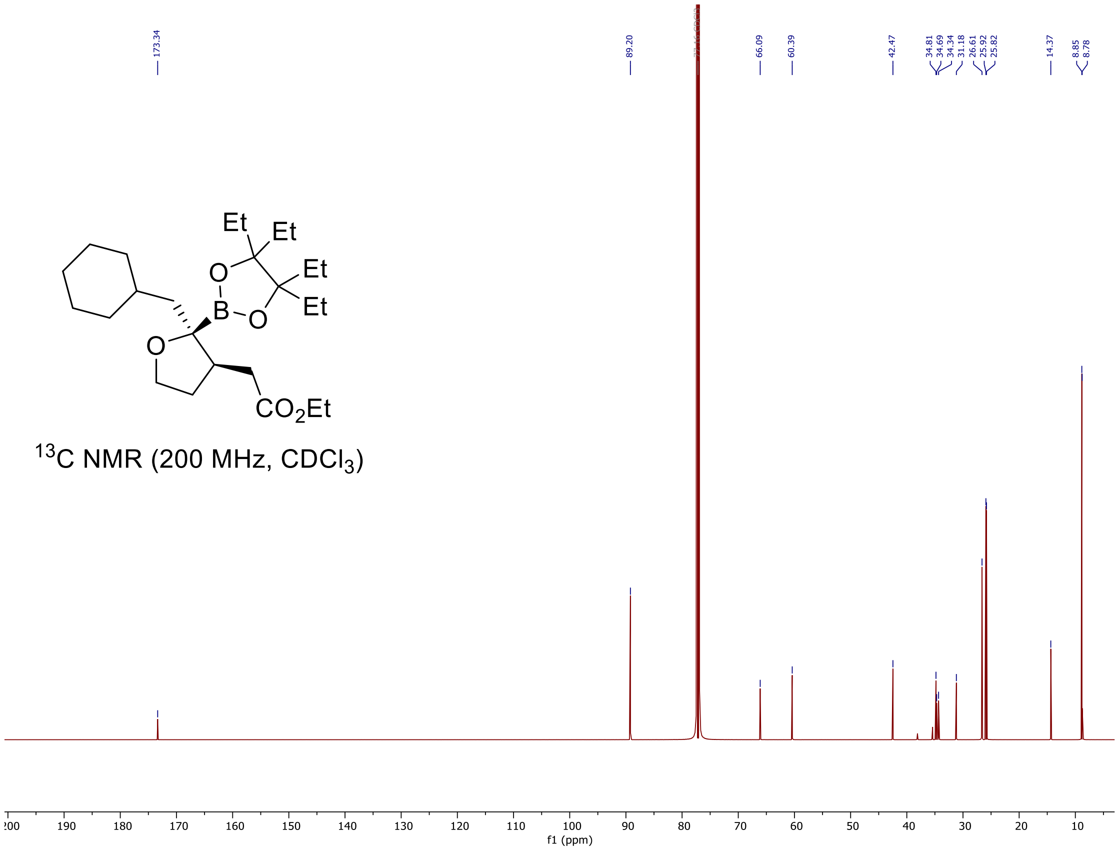

Ethyl 2-((2*S*,3*S*)-3-(4,4,5,5-tetraethyl-1,3,2-dioxaborolan-2-yl)-3-((trimethylsilyl)methyl)bicyclo[2.2.1]heptan-2-yl)acetate (15a)

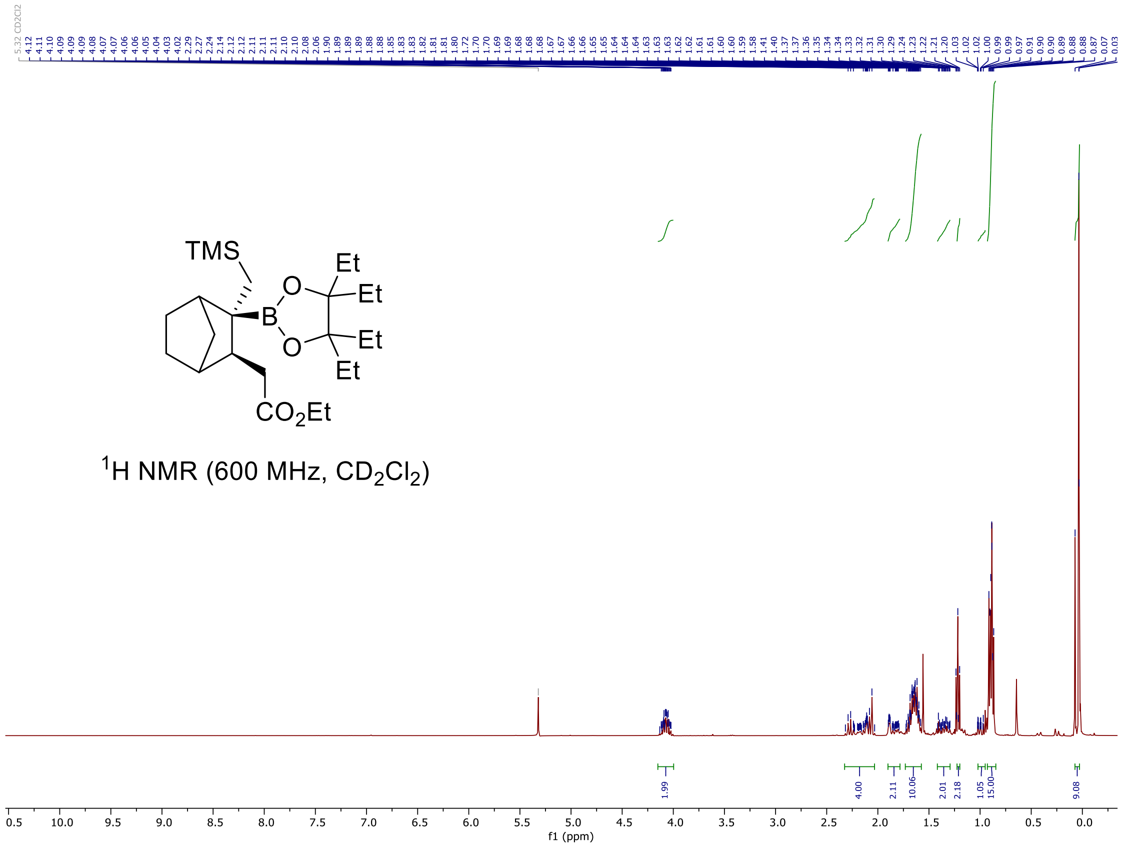

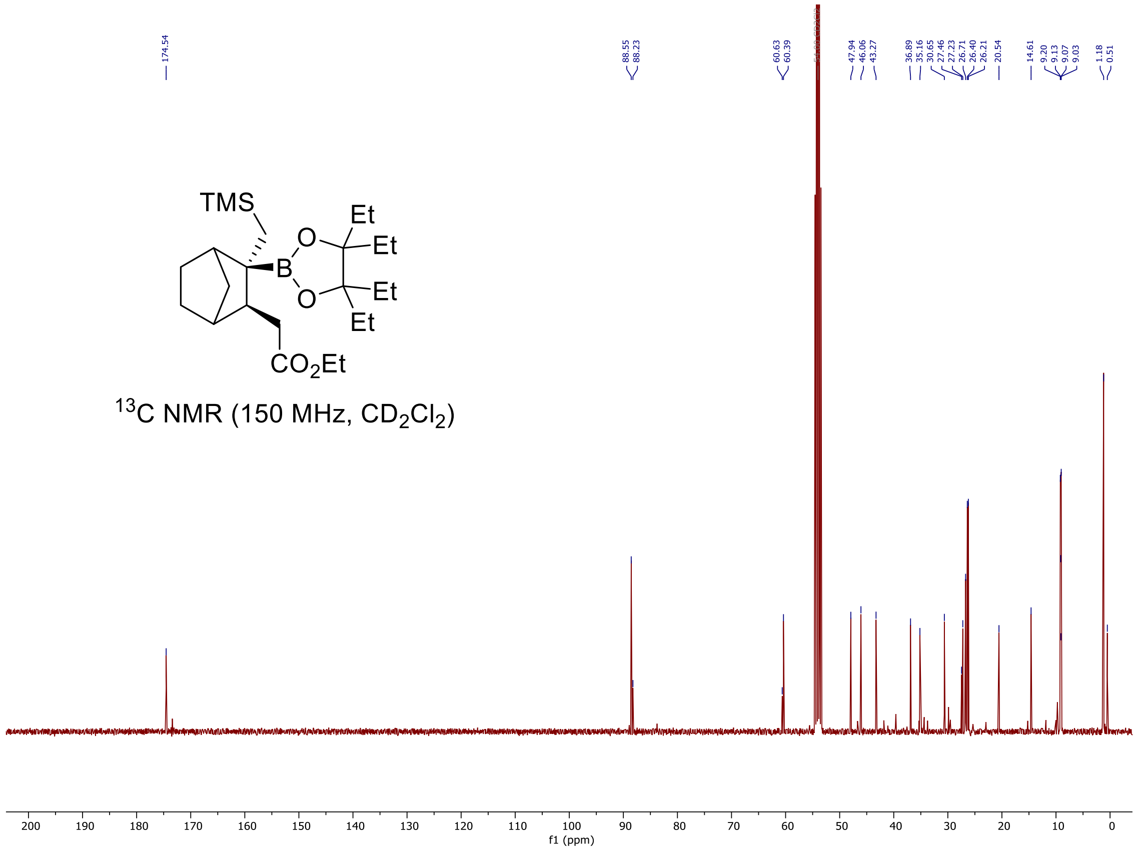

Ethyl 2-((2*S*,3*S*)-3-(4-(*tert*-butyl)phenyl)-3-(4,4,5,5-tetraethyl-1,3,2-dioxaborolan-2-yl)bicyclo[2.2.1]heptan-2-yl)acetate (15b)

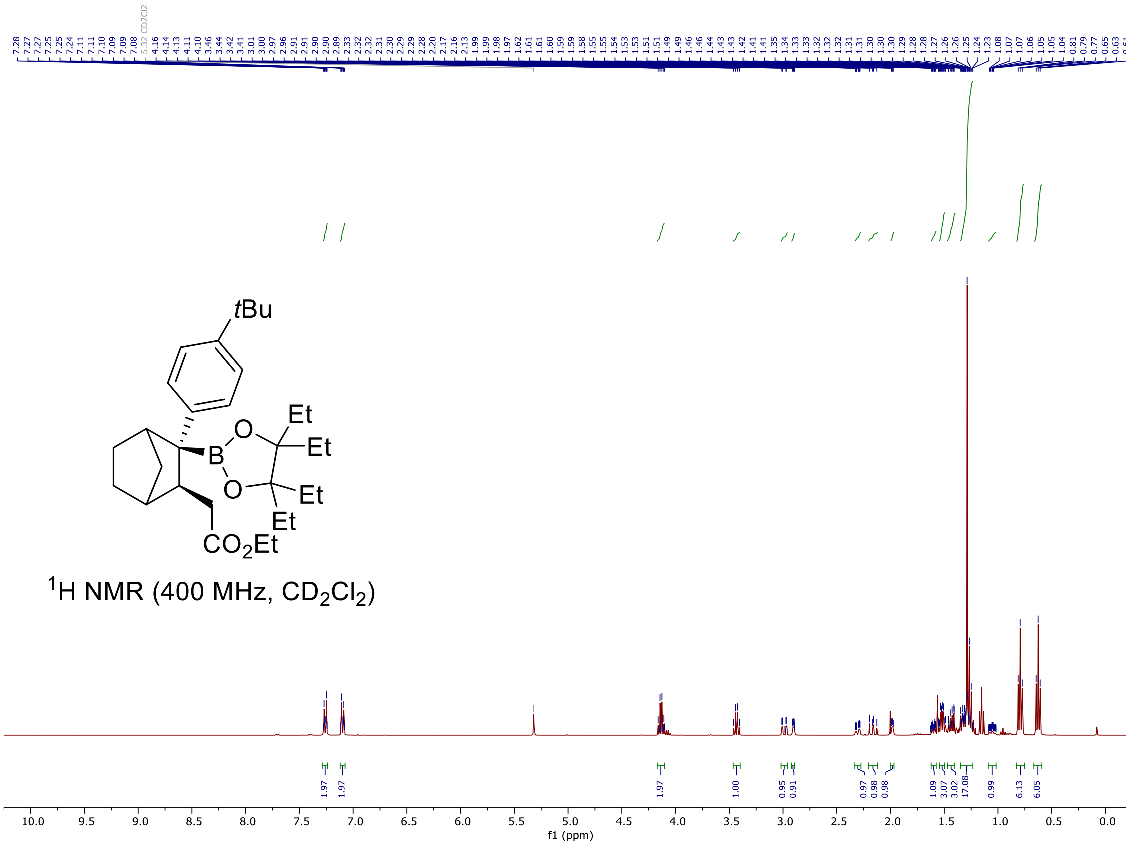

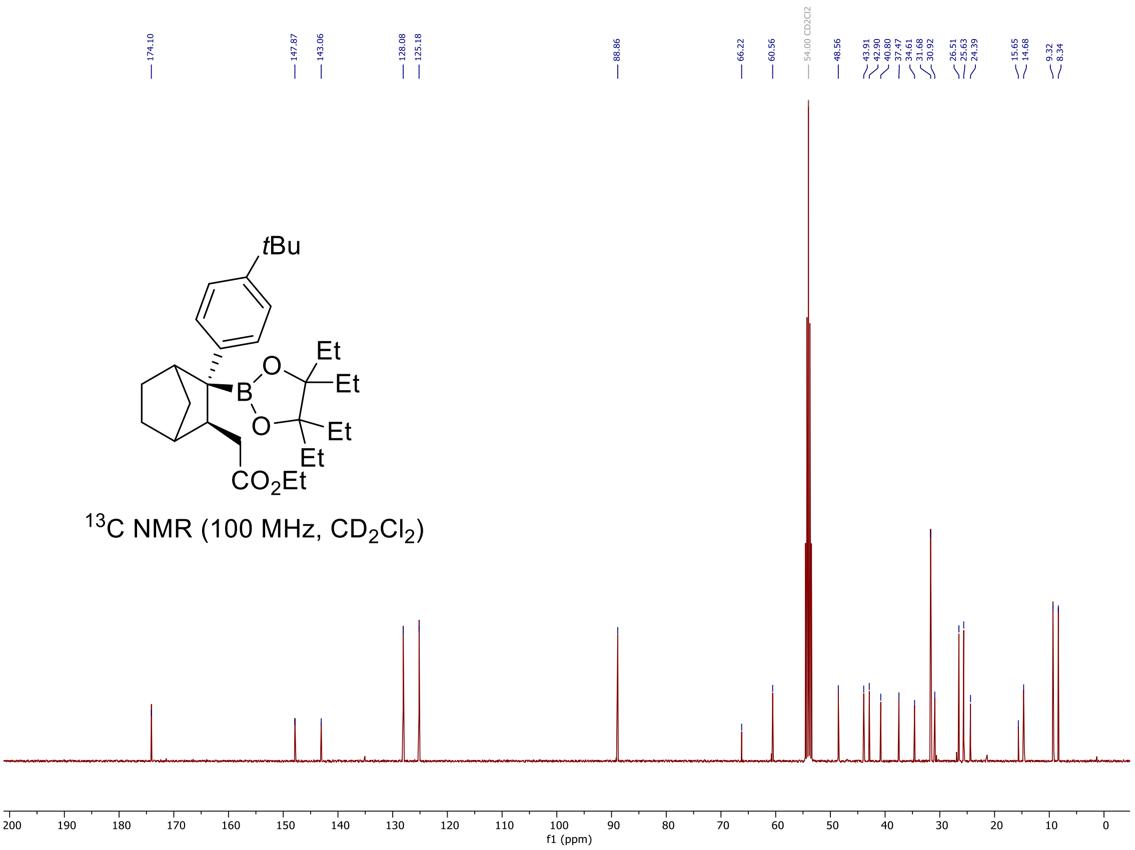

Ethyl 2-((2*S*,3*S*)-3-(benzo[d][1,3]dioxol-5-yl)-3-(4,4,5,5-tetraethyl-1,3,2-dioxaborolan-2-yl)bicyclo[2.2.1]heptan-2-yl)acetate (15c)

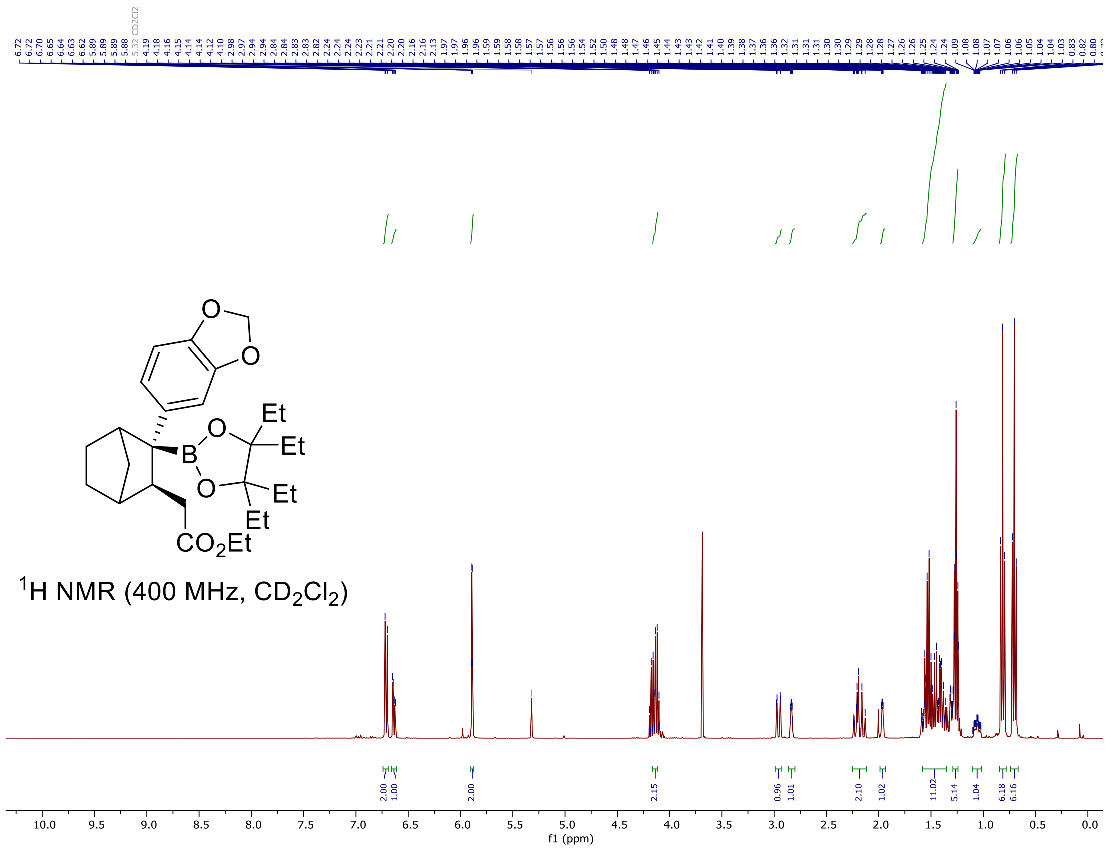

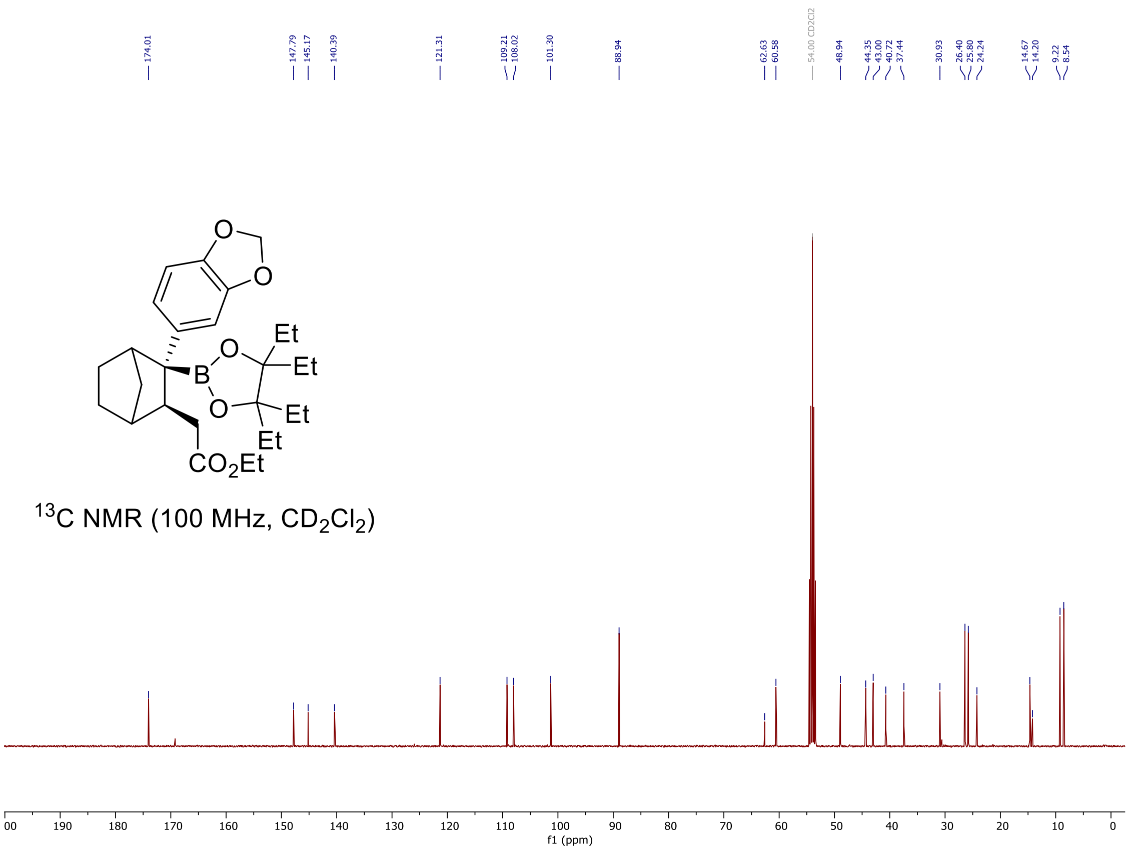

4-(4-((2*S*,3*S*)-2-(4,4,5,5-tetraethyl-1,3,2-dioxaborolan-2-yl)-3-(trifluoromethyl)bicyclo[2.2.1]heptan-2-yl)benzyl)morpholine (15d)

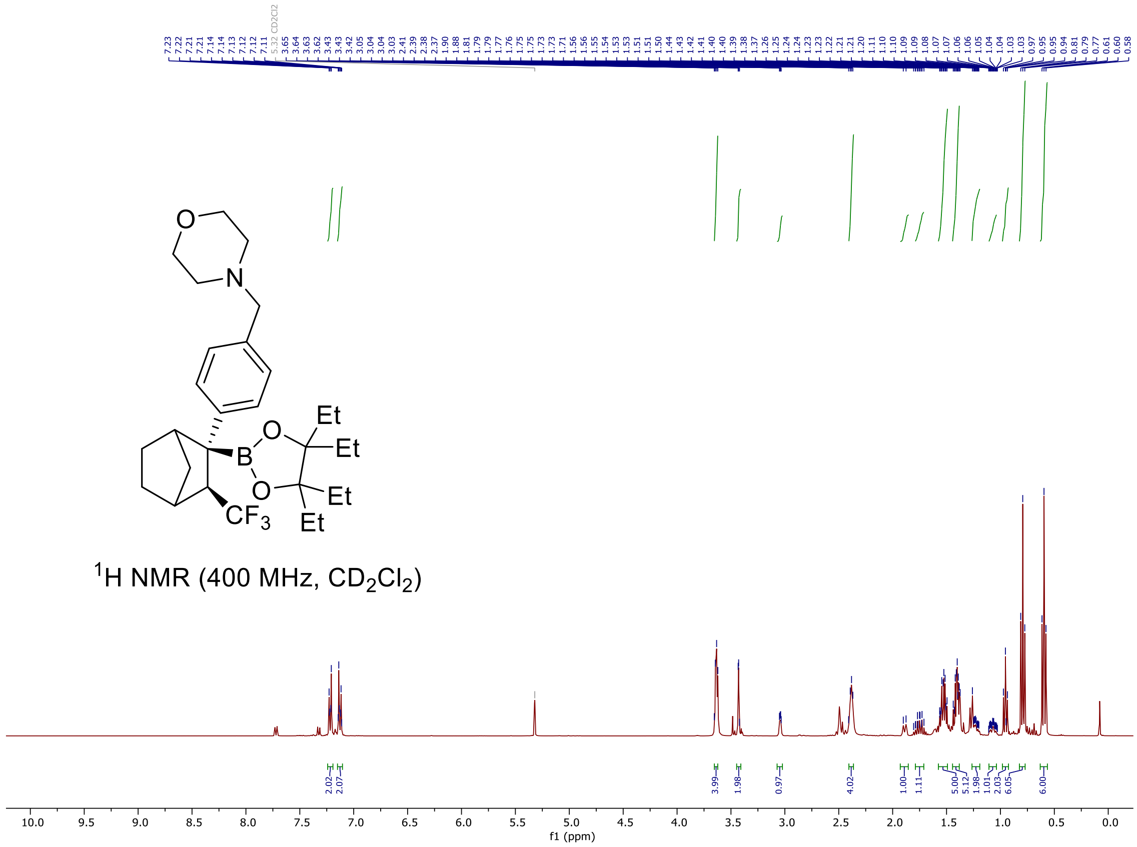

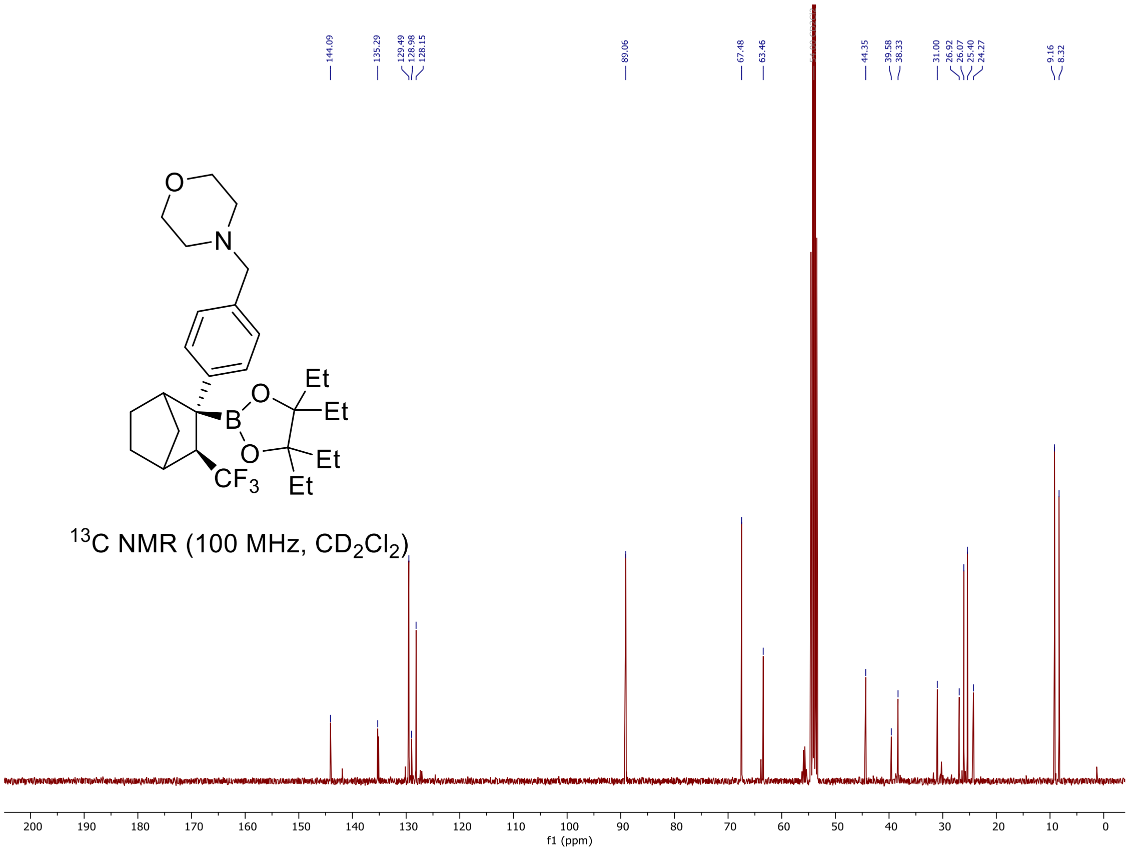

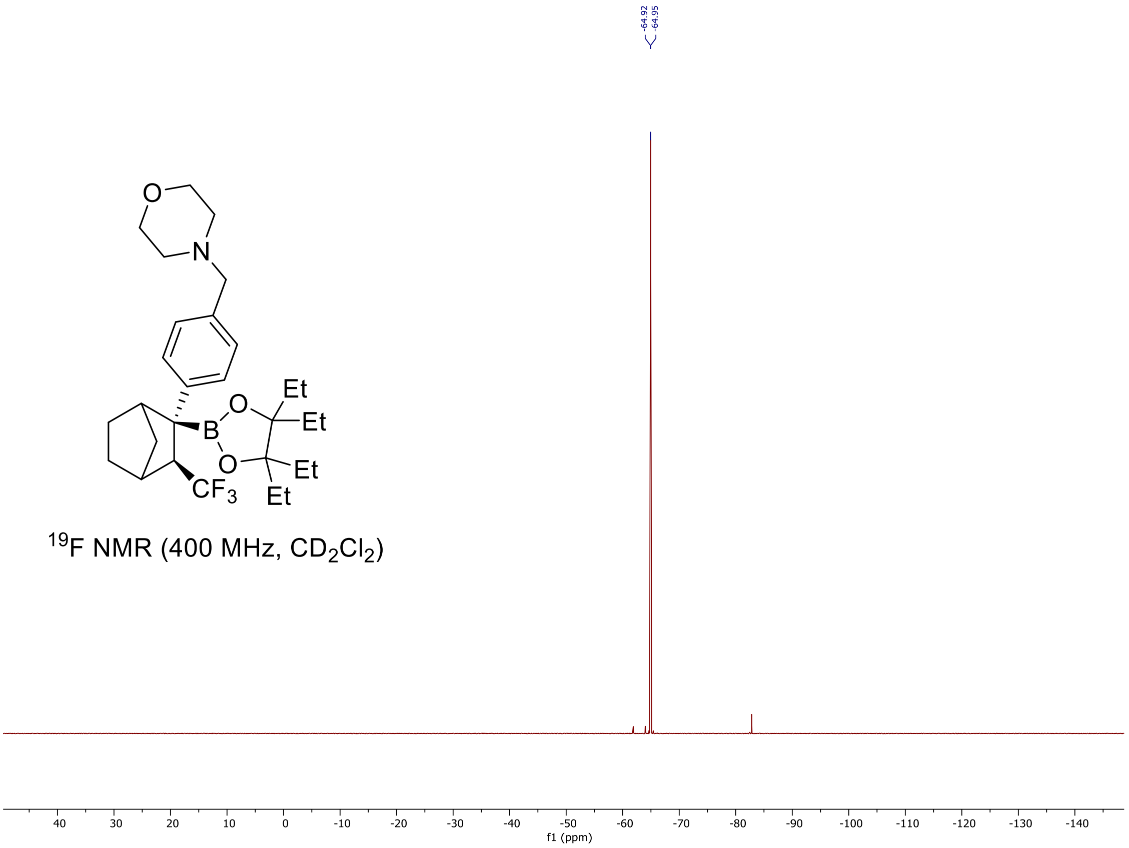

^{19}F NMR (400 MHz, CD_2Cl_2)

(3a*R*,6a*R*)-6a-hexylhexahydro-2H-cyclopenta[b]furan-2-one (<u>16</u>)

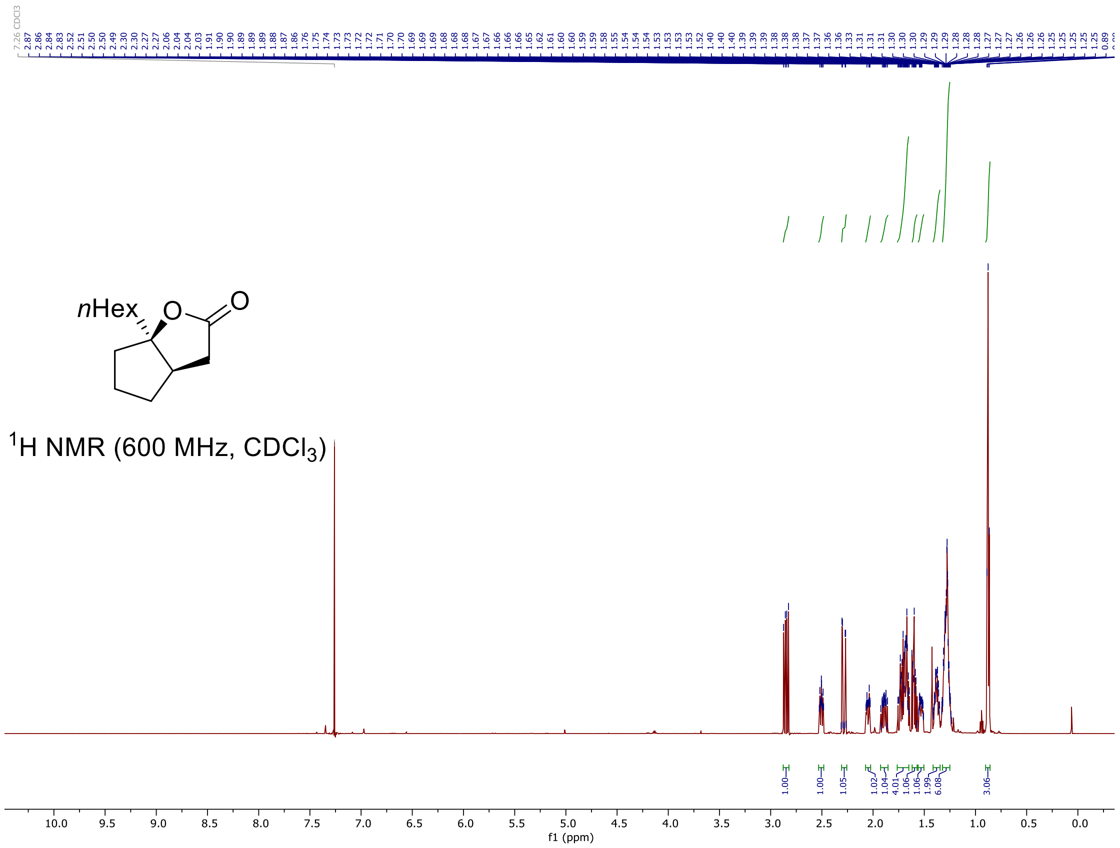

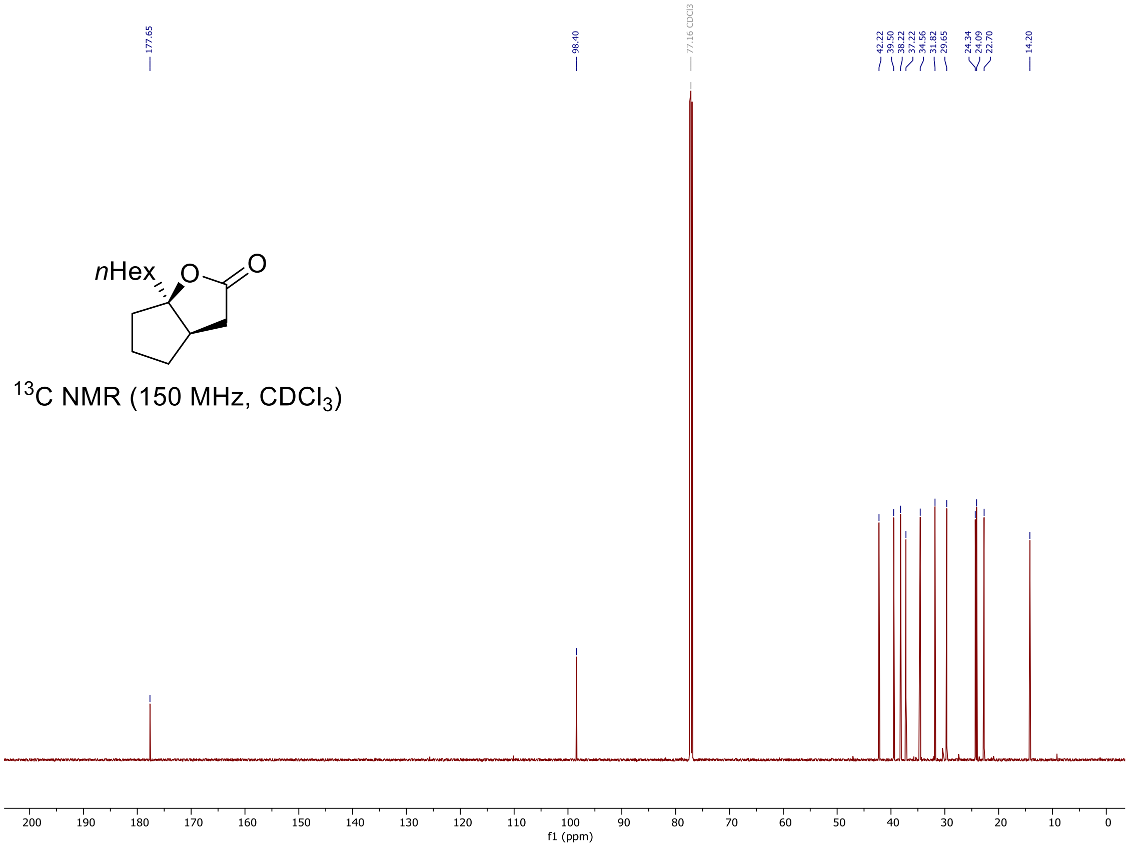

(2*R*,3*S*)-1-benzyl-2-butyl-3-(perfluorobutyl)-2-(4,4,5,5-tetraethyl-1,3,2-dioxaborolan-2-yl)azetidine (<u>17a</u>)

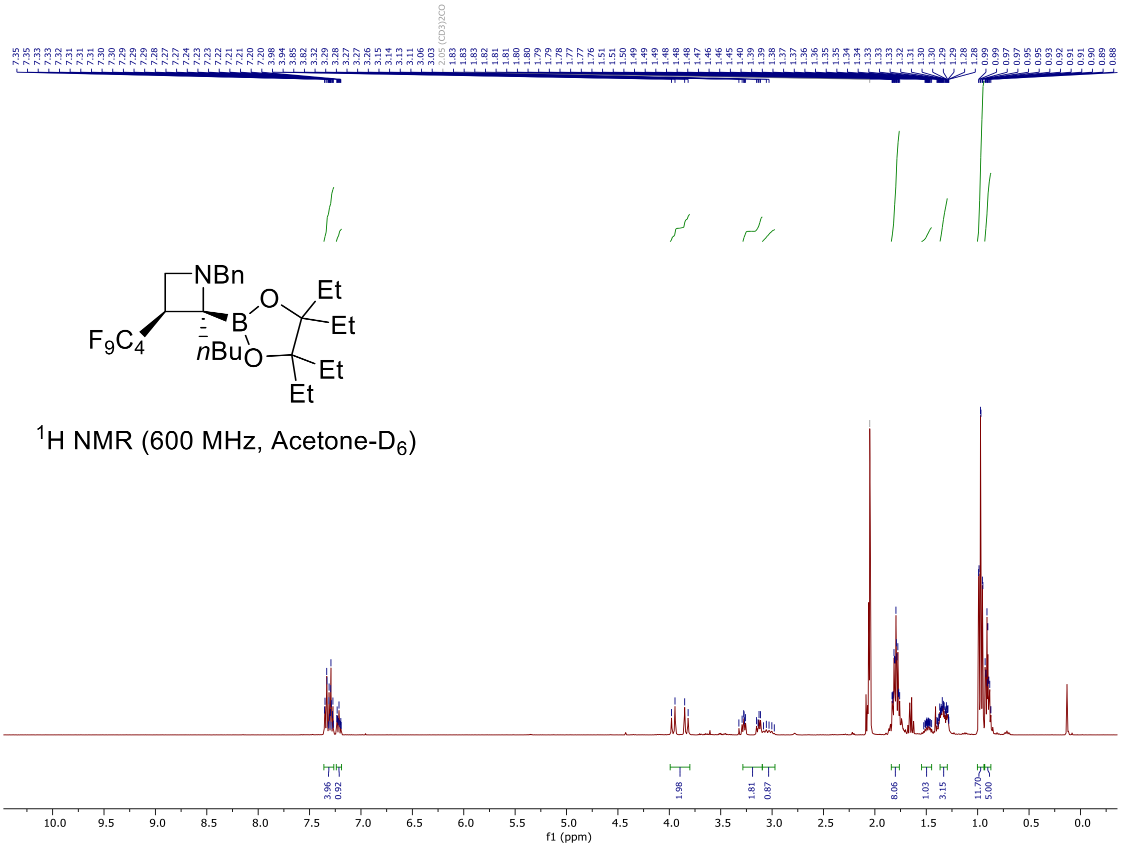

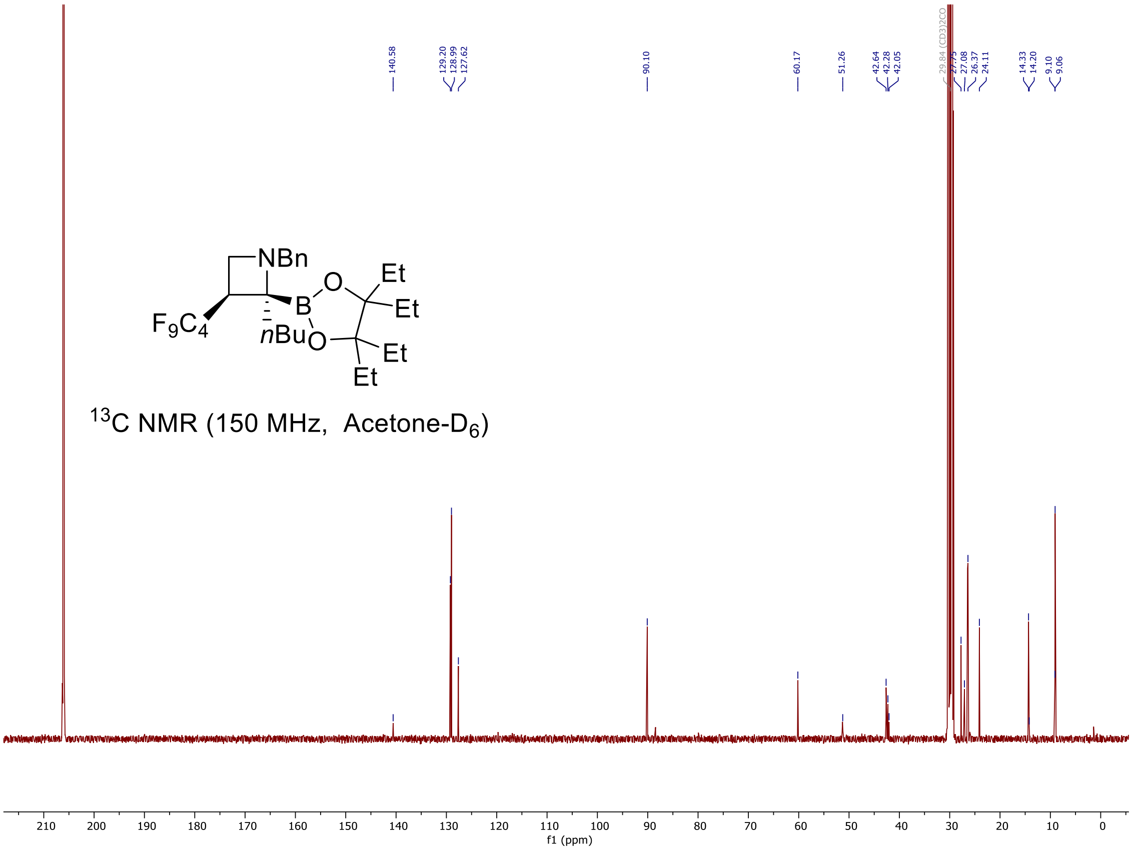

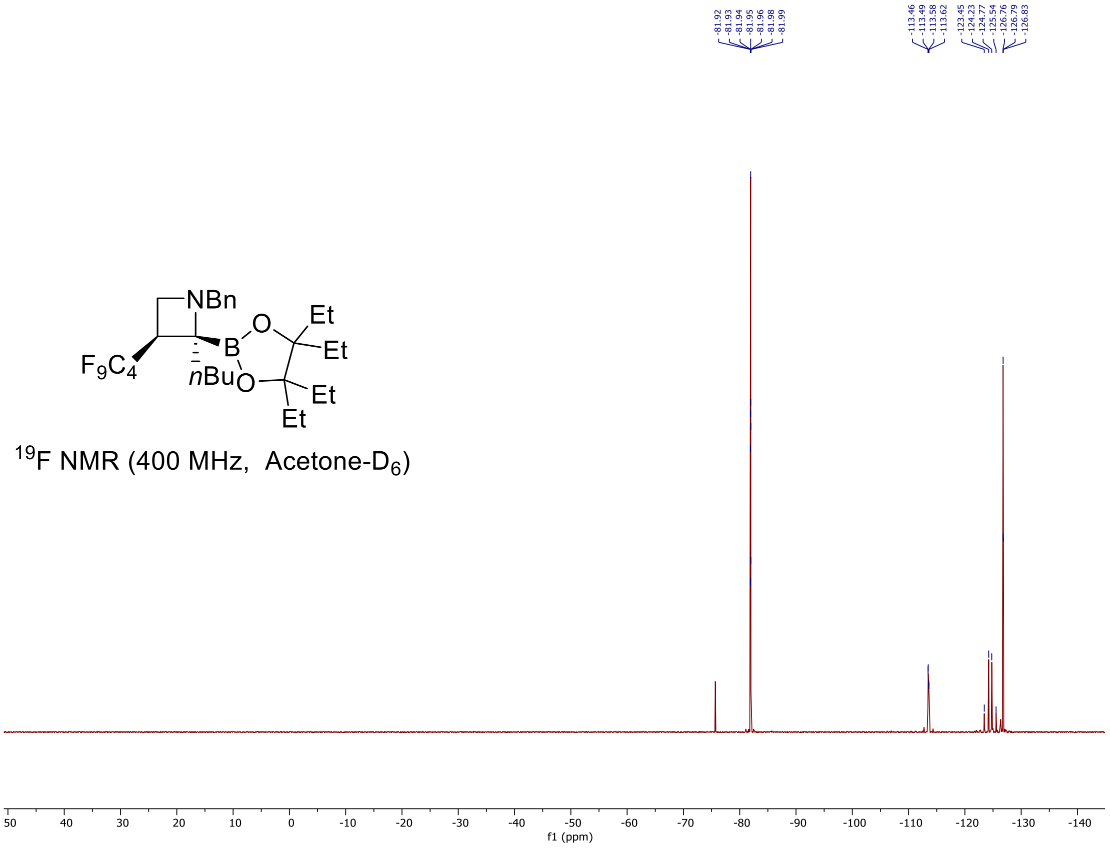
NBn
O
Et
B
Et
F9C4
nBu
O
Et
Et
19F NMR (400 MHz, Acetone-D6)
-81.92
-81.93
-81.94
-81.95
-81.96
-81.98
-81.99
-113.46
-113.49
-113.58
-113.62
-123.45
-124.23
-124.77
-125.54
-126.76
-126.79
-126.83
50 40 30 20 10 0 -10 -20 -30 -40 -50 -60 -70 -80 -90 -100 -110 -120 -130 -140
f1 (ppm)

Ethyl 2-((1*R*,2*S*)-2-(trifluoro-l4-boraneyl)-2-((trimethylsilyl)methyl)cyclopentyl)acetate, potassium salt (<u>17b</u>)

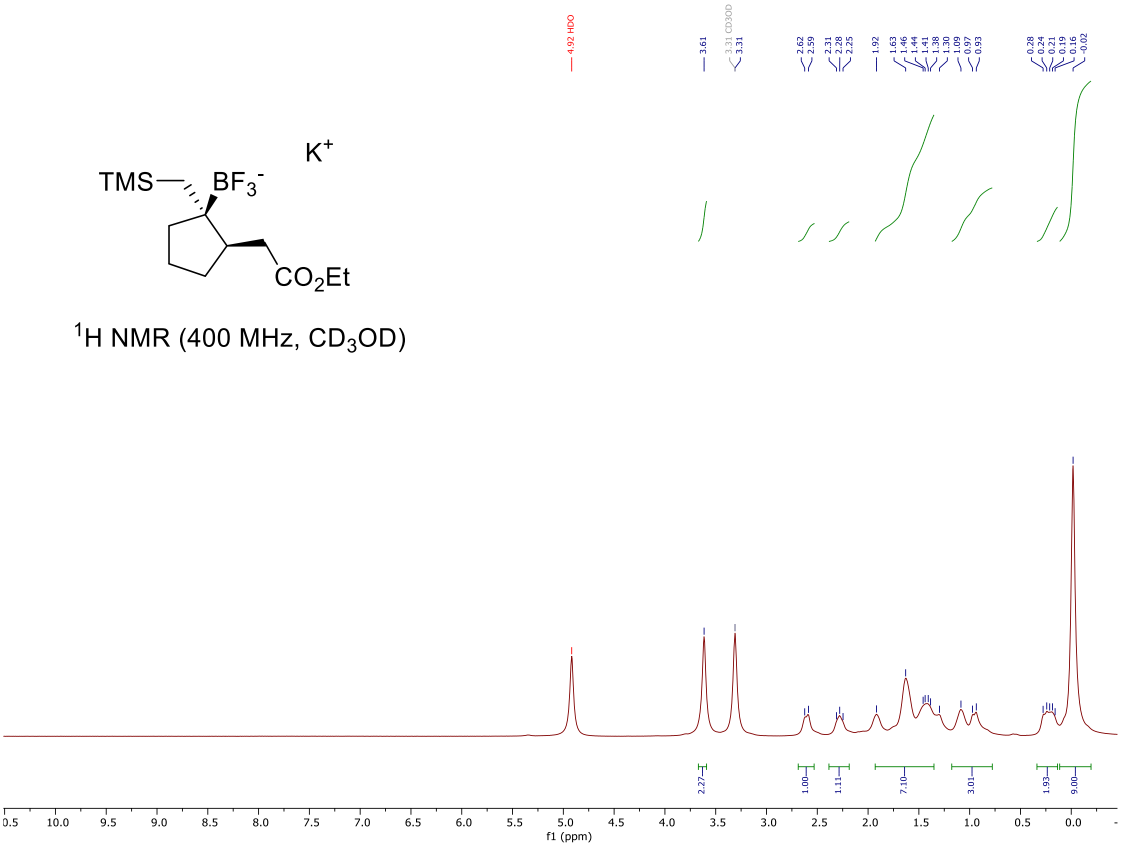

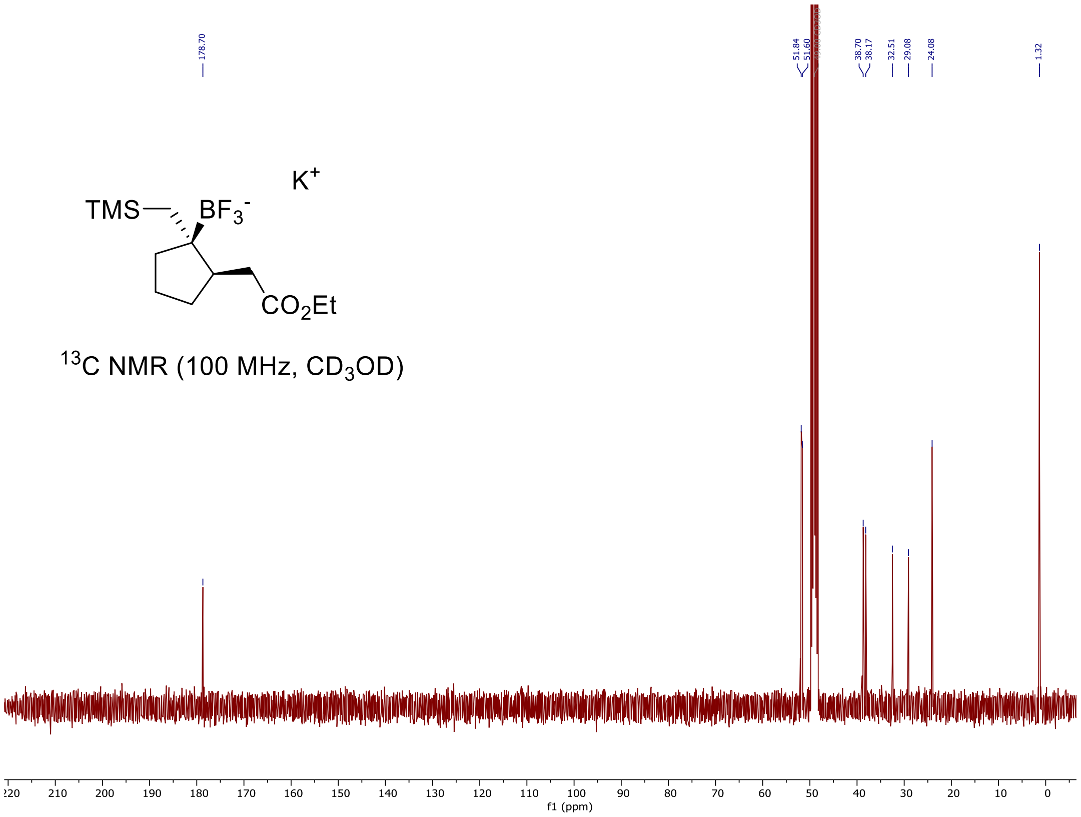

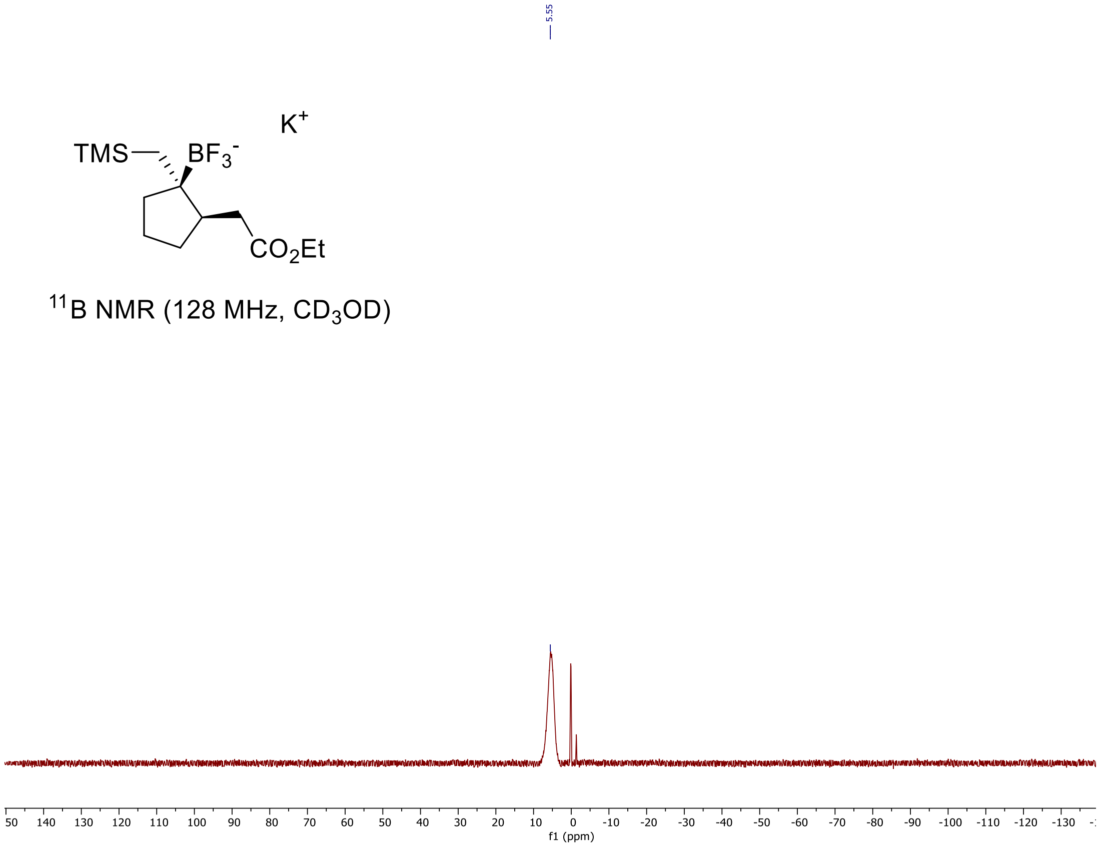
5.55
K+
TMS
BF3-
CO2Et
11B NMR (128 MHz, CD3OD)
f1 (ppm)

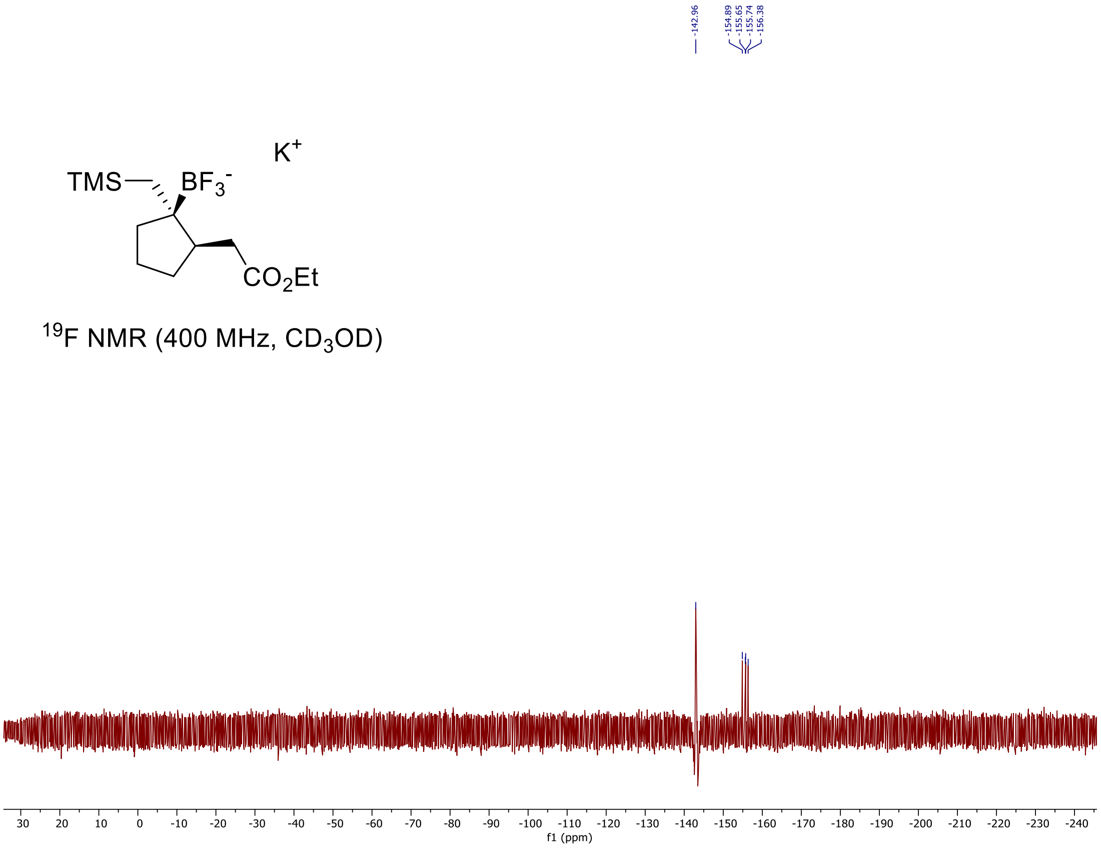
-142.96
-154.89
-155.65
-155.74
-156.38
K+
TMS
BF3-
CO2Et
19F NMR (400 MHz, CD3OD)
f1 (ppm)

(4a*R*,7a*S*)-7a-Butyl-1-hydroxyhexahydrocyclopenta[c][1,2]oxaborinin-3(1H)-one (17c)

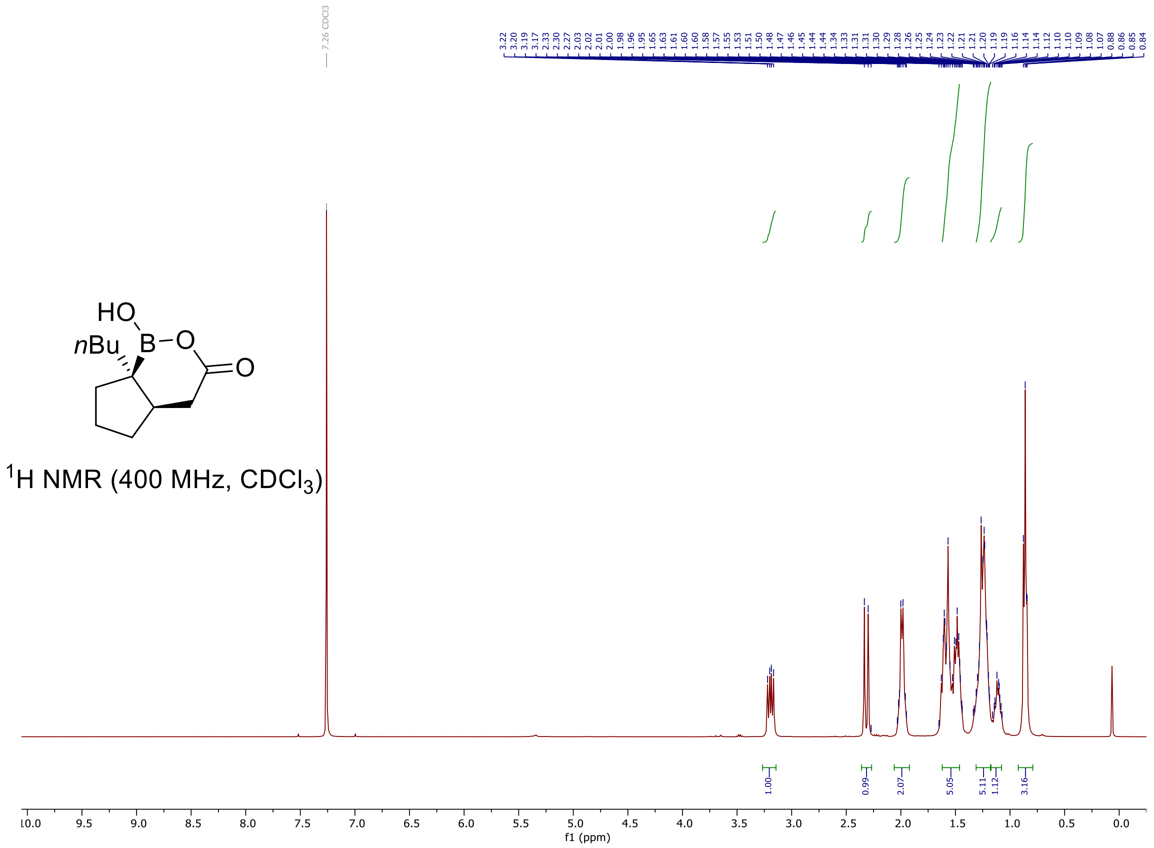

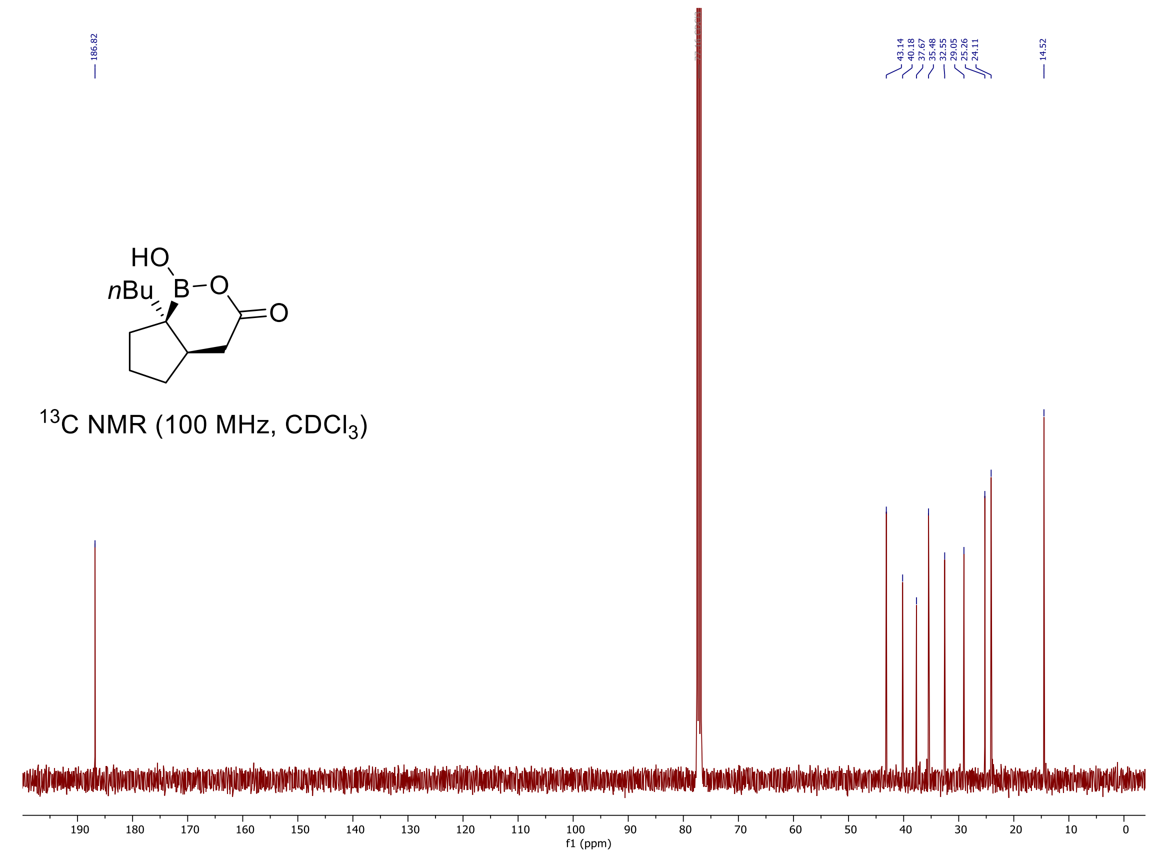

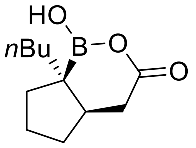

^{11}B NMR (128 MHz, $CDCl_3$)

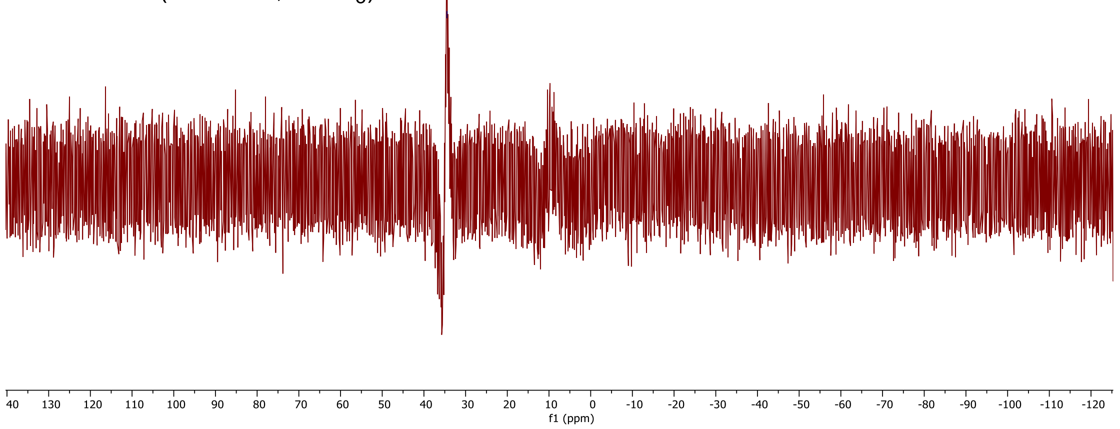

(2*R*,3*S*)-1-Benzyl-2-isobutyl-3-(perfluorobutyl)-2-(4,4,5,5-tetraethyl-1,3,2-dioxaborolan-2-yl)azetidine (<u>17b</u>)

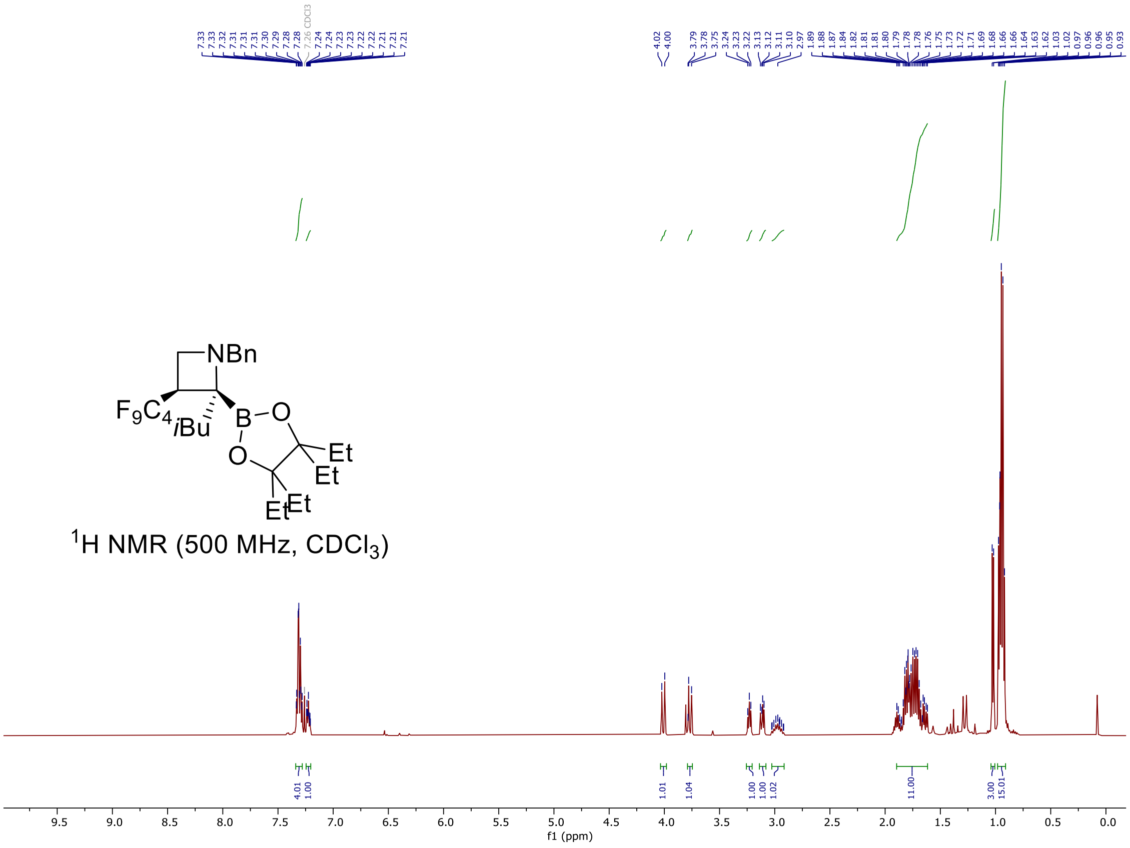

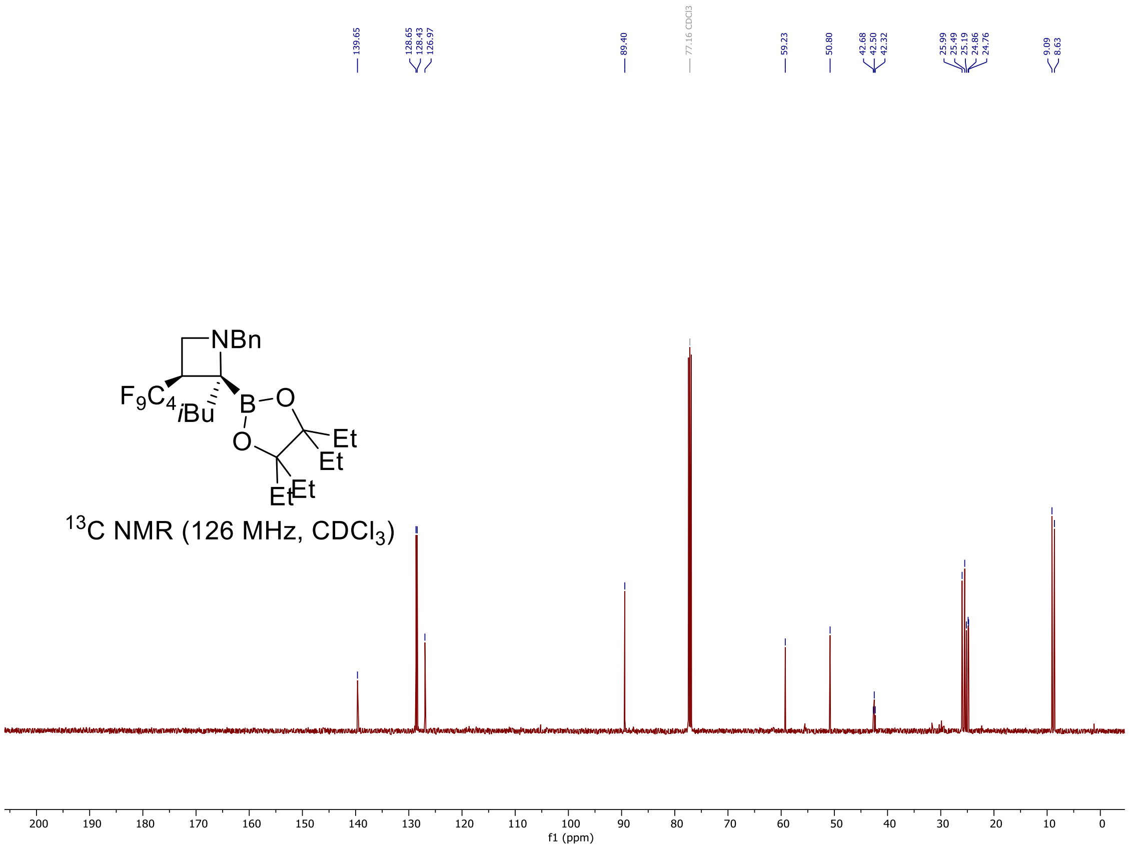

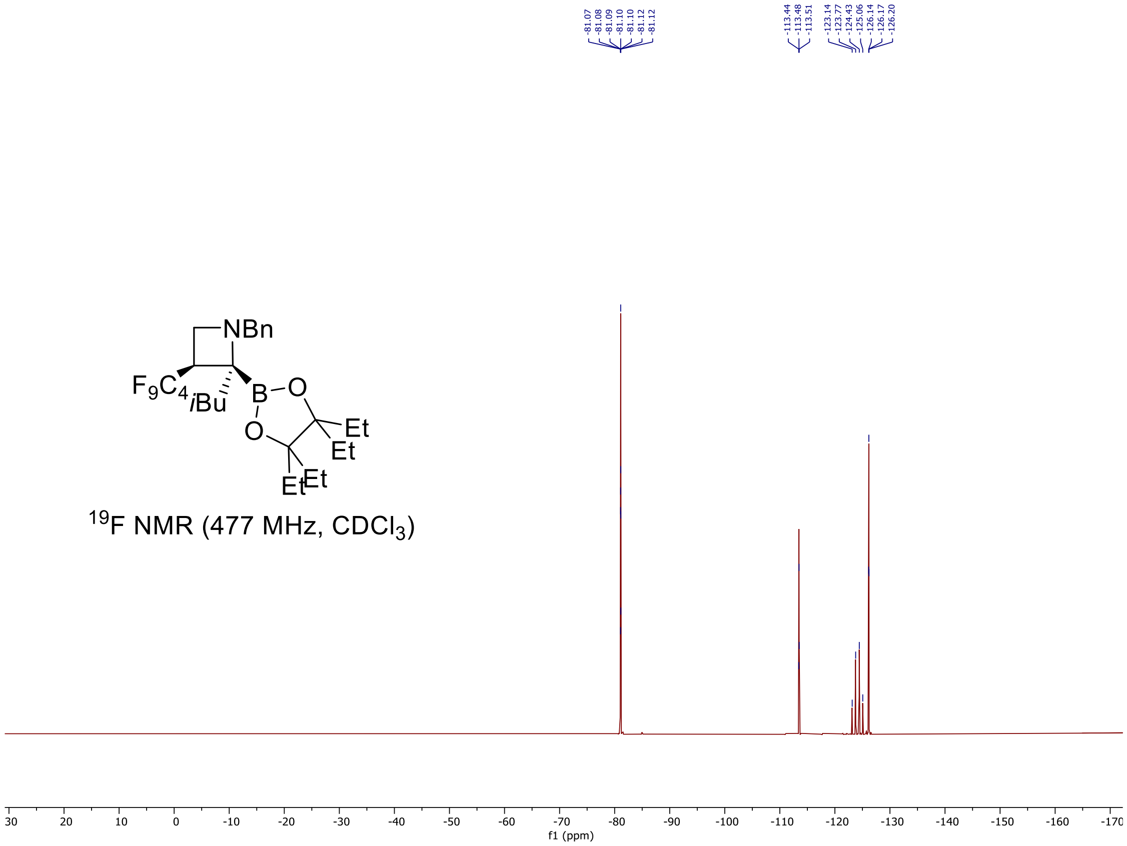

^{19}F NMR (477 MHz, $CDCl_3$)

Ethyl 2-((2*R*,3*S*)-1-benzyl-2-isobutyl-2-(4,4,5,5-tetraethyl-1,3,2-dioxaborolan-2-yl)azetidin-3-yl)acetate (18)

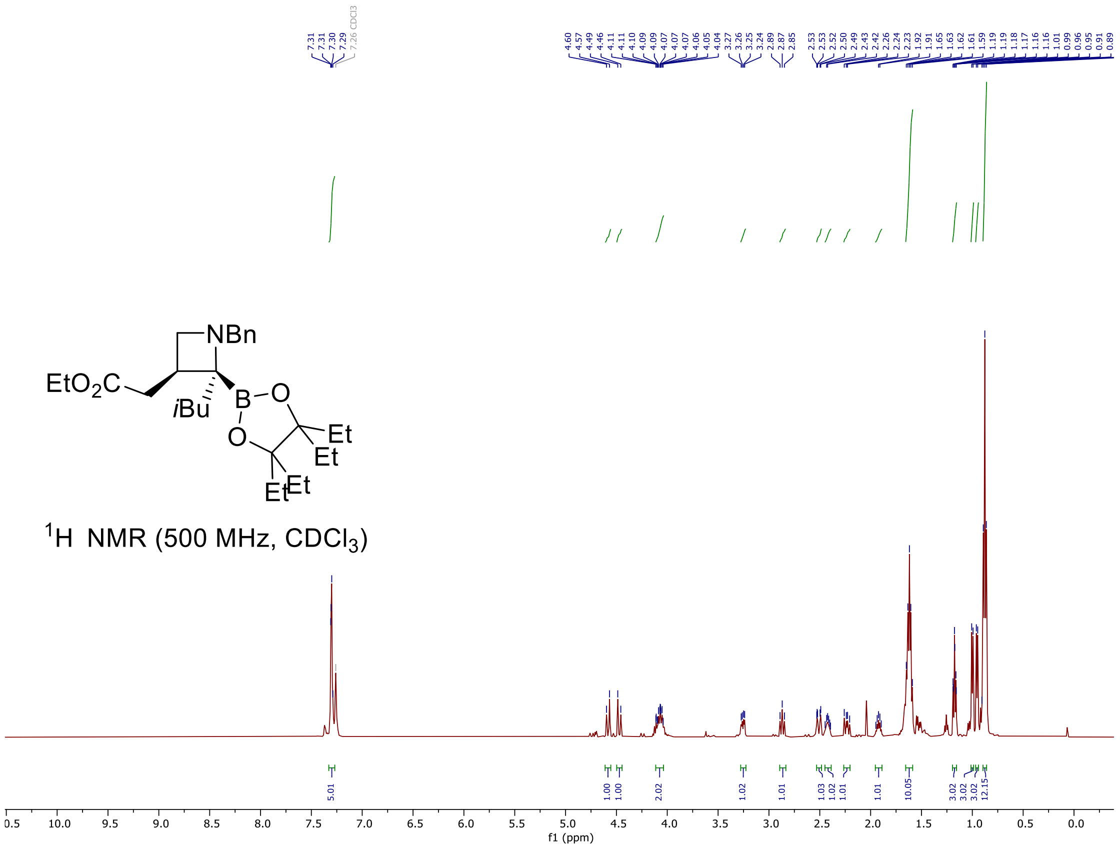

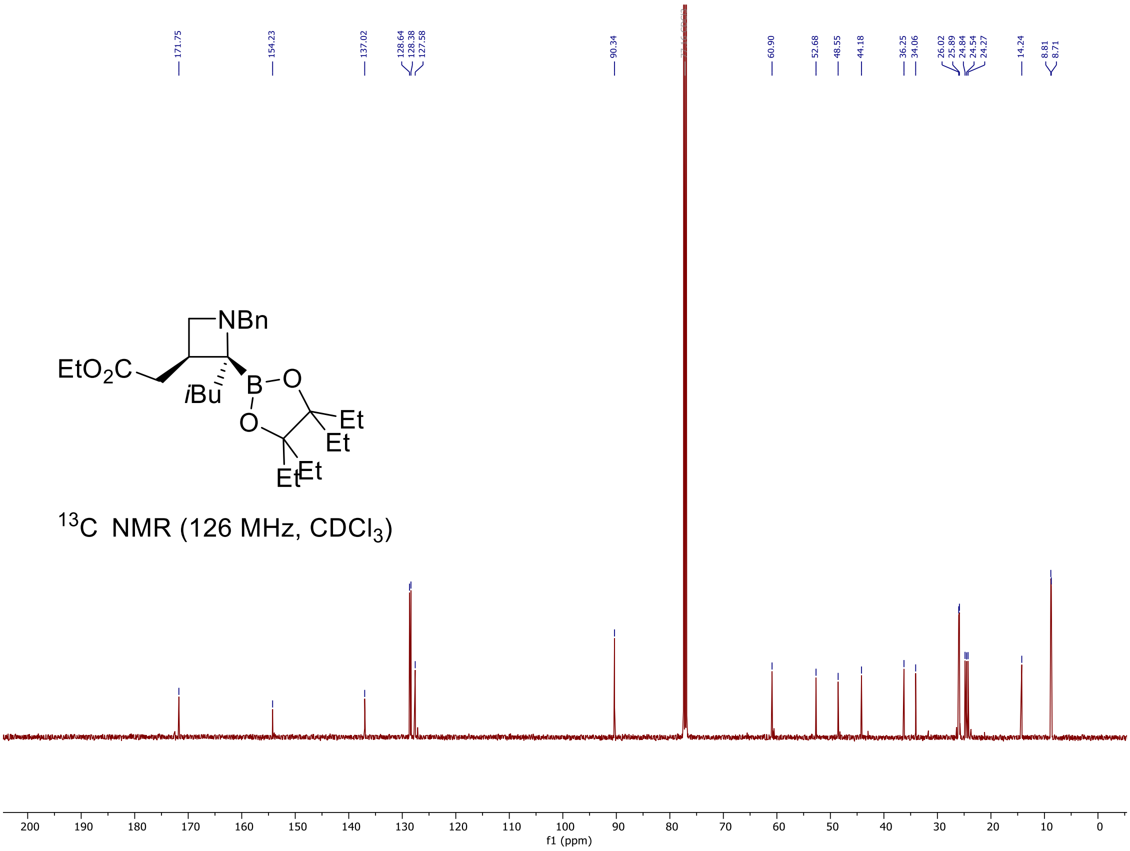

(1*R*,5*R*)-7-Benzyl-1-isobutyl-2-oxa-7-azabicyclo[3.2.0]heptan-3-one (<u>20</u>)

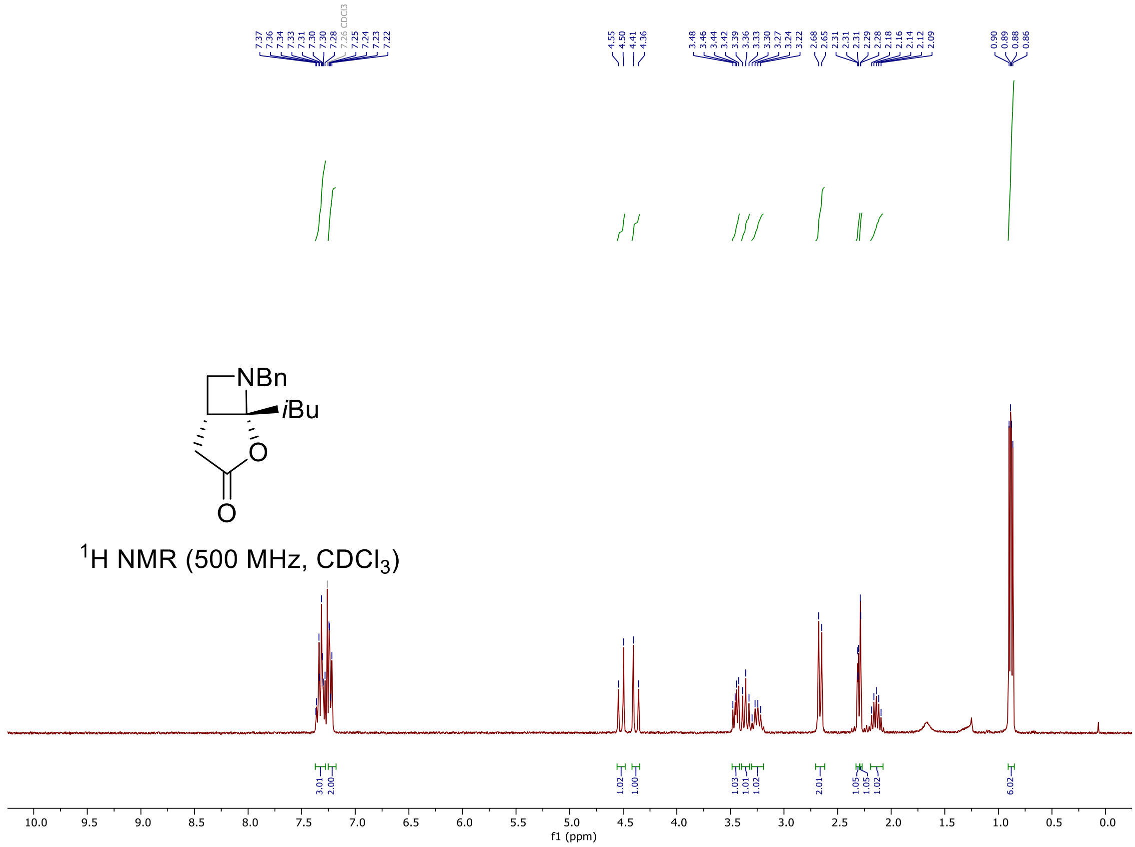

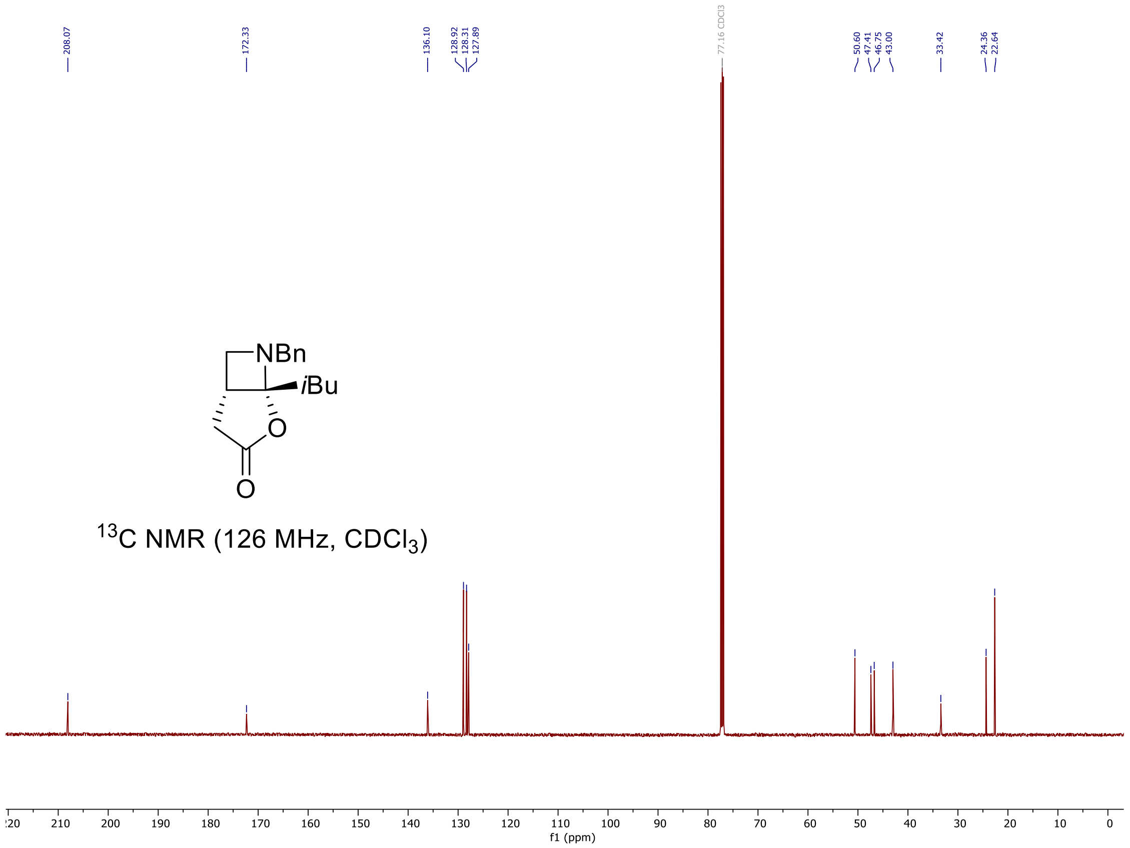

(2*R*,3*S*)-1-Benzyl-2-isobutyl-3-(perfluorobutyl)-2-(trifluoro-l4-boraneyl)azetidine, potassium salt (21)

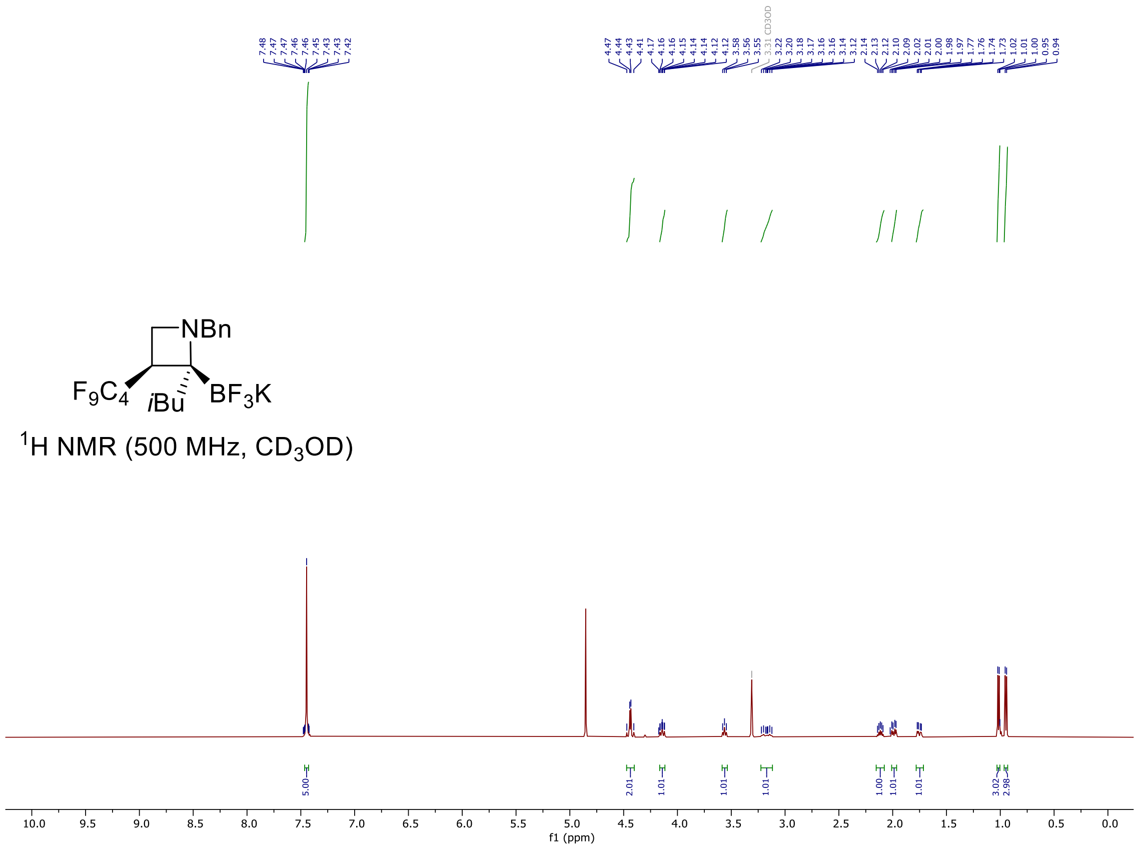

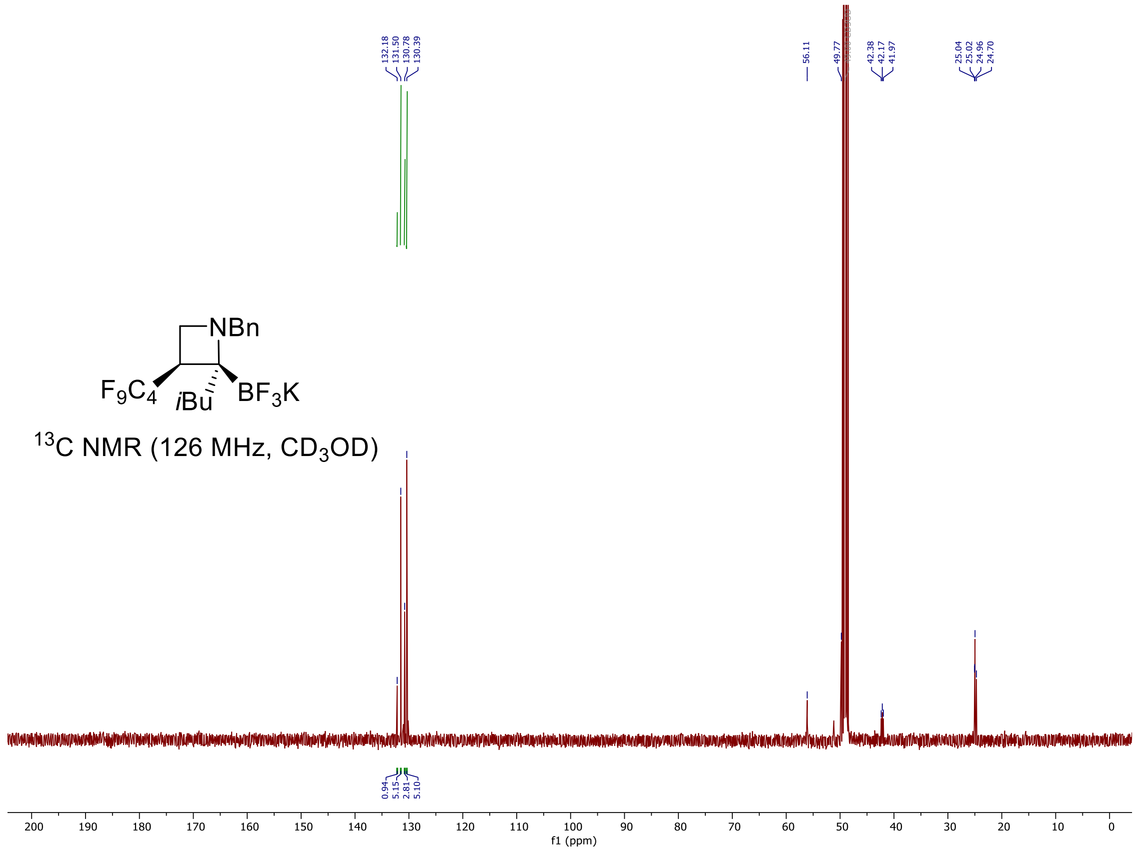

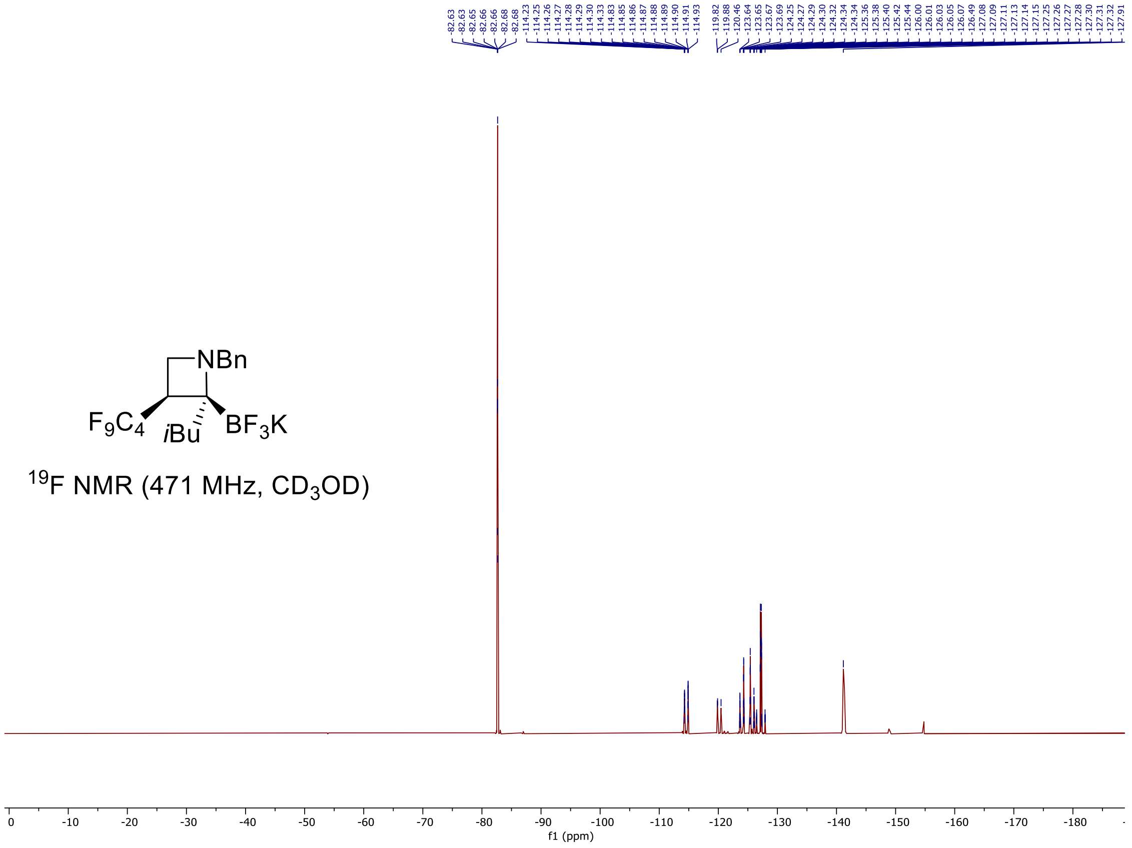

^{19}F NMR (471 MHz, CD_3OD)
